# Associations of Body Mass and Fat Indexes With Cardiometabolic Traits

**DOI:** 10.1016/j.jacc.2018.09.066

**Published:** 2018-12-18

**Authors:** Joshua A. Bell, David Carslake, Linda M. O’Keeffe, Monika Frysz, Laura D. Howe, Mark Hamer, Kaitlin H. Wade, Nicholas J. Timpson, George Davey Smith

**Affiliations:** a MRC Integrative Epidemiology Unit at the University of Bristol, Bristol, United Kingdom; b Population Health Sciences, Bristol Medical School, University of Bristol, Bristol, United Kingdom; c School of Sport, Exercise & Health Sciences, Loughborough University, Leicestershire, United Kingdom

**Keywords:** ALSPAC, body mass index, cardiometabolic traits, DXA, epidemiology, BMI, body mass index, CHD, coronary heart disease, CI, confidence interval, CRP, C-reactive protein, DBP, diastolic blood pressure, DXA, dual-energy x-ray absorptiometry, HDL, high-density lipoprotein, LDL, low-density lipoprotein, SBP, systolic blood pressure, VLDL, very-low-density lipoprotein

## Abstract

**Background:**

Body mass index (BMI) is criticized for not distinguishing fat from lean mass and ignoring fat distribution, leaving its ability to detect health effects unclear.

**Objectives:**

The aim of this study was to compare BMI with total and regional fat indexes from dual-energy x-ray absorptiometry in their associations with cardiometabolic traits. Duration of exposure to and change in each index across adolescence were examined in relation to detailed traits in young adulthood.

**Methods:**

BMI was examined alongside total, trunk, arm, and leg fat indexes (each in kilograms per square meter) from dual-energy x-ray absorptiometry at ages 10 and 18 years in relation to 230 traits from targeted metabolomics at age 18 years in 2,840 offspring from the Avon Longitudinal Study of Parents and Children.

**Results:**

Higher total fat mass index and BMI at age 10 years were similarly associated with cardiometabolic traits at age 18 years, including higher systolic and diastolic blood pressure, higher very low-density lipoprotein and low-density lipoprotein cholesterol, lower high-density lipoprotein cholesterol, higher triglycerides, and higher insulin and glycoprotein acetyls. Associations were stronger for both indexes measured at age 18 years and for gains in each index from age 10 to 18 years (e.g., 0.45 SDs [95% confidence interval: 0.38 to 0.53] in glycoprotein acetyls per SD unit gain in fat mass index vs. 0.38 SDs [95% confidence interval: 0.27 to 0.48] per SD unit gain in BMI). Associations resembled those for trunk fat index. Higher lean mass index was weakly associated with traits and was not protective against higher fat mass index.

**Conclusions:**

The results of this study support abdominal fatness as a primary driver of cardiometabolic dysfunction and BMI as a useful tool for detecting its effects.

Obesity is a public health crisis with unabating rates worldwide (1). Fatness is most commonly measured in populations using body mass index (BMI), a simple ratio of weight to squared height, because it is easily measured and highly correlated with more objective fat mass 2, 3. Causal analyses support effects of higher BMI on coronary heart disease (CHD) and its intermediates of low-density lipoprotein (LDL) and remnant cholesterol, blood pressure, and glucose 4, 5, 6. Despite this, BMI is often criticized for not distinguishing fat from lean mass and ignoring fat distribution 7, 8, which may limit its ability to reveal true health effects.

Moderate positive correlations exist between total fat and lean mass and between BMI and lean mass 3, 9, suggesting that true fat volume varies considerably among subjects with high BMI. This variation matters if body tissues have distinct effects on disease intermediates. For example, insulin resistance and dyslipidemia may depend most on excessive fat volume given adipocyte functions of lipolysis and nonesterified fatty acid release 10, 11, while hyperglycemia and inflammation may depend most on insufficient lean volume given skeletal muscle functions of glucose absorption and myokine release upon contraction 12, 13. These expectations are based on small laboratory-based human studies 10, 14 or epidemiological studies relating one-off measures of fat and lean mass to either few metabolic traits among many participants (15) or many metabolic traits among few participants (16). Gains in fat and lean mass are rarely examined, particularly in relation to detailed cholesterol and triglyceride subtypes and clinical trait precursors such as amino acids.

Fat stored centrally in the abdominal trunk region most closely reflects metabolically active visceral fat (17) and strongly influences CHD risk and its intermediates (18). Effects are often measured using waist circumference or waist-to-hip ratio, with scant evidence based on more objective trunk fat. Evidence is also limited on the effects of peripheral fat. Some studies suggest enhanced insulin sensitivity and reduced inflammation with higher leg fat 19, 20, but prospective evidence is lacking. Importantly, the extent to which more objective measures of total and regional fat offer insights into cardiometabolic effects that are not detectable by simple BMI is unknown.

In this study, we compared BMI with total and regional fat indexes from dual-energy x-ray absorptiometry (DXA), a scan that isolates fat and lean tissue from total body mass, in their associations with cardiometabolic traits relevant to CHD. We used data from a population-based birth cohort study to examine duration of exposure to and change in each index across adolescence in relation to detailed traits from targeted metabolomics in young adulthood. We also examined whether independent or interactive associations exist between fat and lean mass in relation to these traits.

## Methods

### Study population

**Figure 1:**
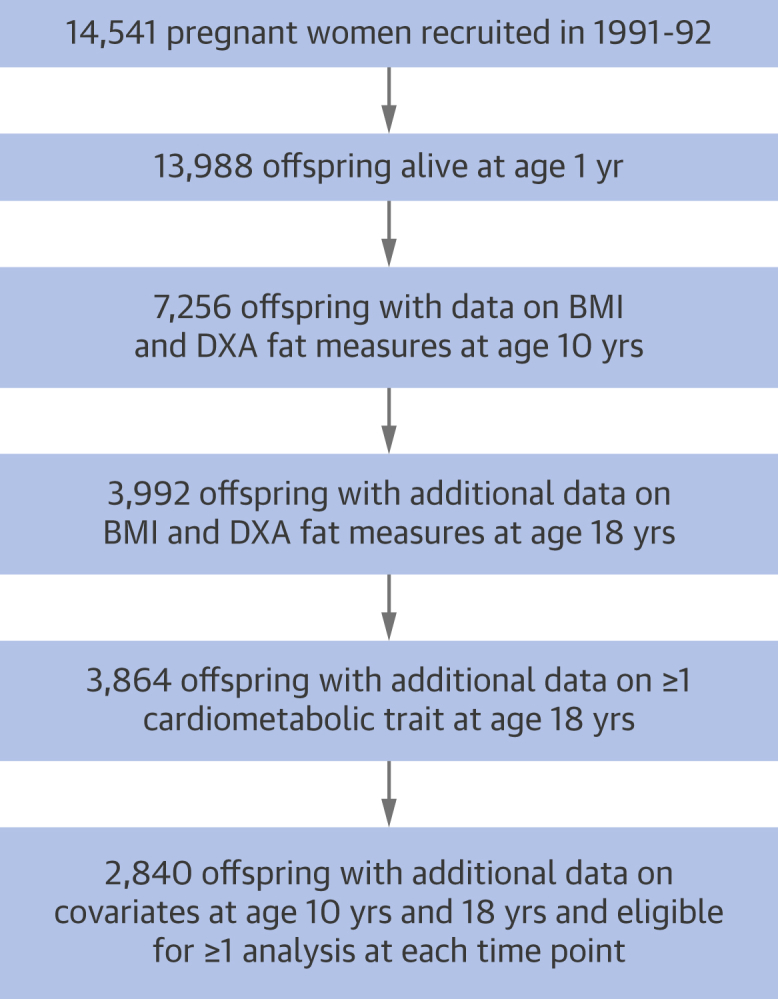
Selection of Participants From the Avon Longitudinal Study of Parents and Children Offspring Cohort Eligible for ≥1 Analysis at Each Time Point Participants described are those with data on body mass index (BMI) and dual-energy x-ray absorptiometry (DXA) fat indexes at 10 and 18 years of age, plus ≥1 cardiometabolic trait at age 18 years, plus covariates. Main analyses of duration (age at measurement comparisons) and change had varying sample sizes ranging from 1,997 to 3,583 participants and were repeated on a complete-case sample of 1,722 participants with data on all variables for comparison.

Data were from offspring participants of ALSPAC (Avon Longitudinal Study of Parents and Children), a population-based birth cohort study in which 14,541 pregnant women expected to deliver between April 1, 1991, and December 31, 1992, were recruited from southwestern England. Offspring who were alive at 1 year (n = 13,988) have since been followed with multiple assessments (21), with an additional 713 children enrolled over the course of the study. Participant selection for analyses is illustrated in Figure 1. Ethical approval was obtained from the ALSPAC law and ethics and local research ethics committees. Cohort details and data descriptions are available online (22).

### Assessment of BMI and DXA fat indexes

Data were collected on 2 occasions during clinical assessments when participants were approximately 10 and 18 years of age. Height was measured in light clothing without shoes to the nearest 0.1 cm using a Harpenden stadiometer. Weight was recorded to the nearest 0.1 kg using a Tanita scale. BMI was calculated as weight (in kilograms) divided by the square of height (in meters). Participants also underwent body scanning with DXA using a Lunar Prodigy narrow fan-beam densitometer from which total body fat mass (in kilograms, excluding lean and bone mass) was quantified. Trunk fat, arm fat, and leg fat (left and right body sides combined) were also quantified. Scans were screened for anomalies, motion, and material artifacts and were realigned when necessary as detailed elsewhere (23). Like BMI, fat indexes were calculated as kilograms per square meter. Lean mass index was also constructed in this way using total DXA-derived lean mass.

### Assessment of cardiometabolic traits

During the 18-year clinic visit, systolic blood pressure (SBP) and diastolic blood pressure (DBP) were examined twice in succession while seated with the arm supported using an appropriately sized cuff and a DINAMAP 9301 device. Mean levels were then calculated. Fasting blood samples were drawn, from which circulating insulin (milliunits per liter) and high-sensitivity C-reactive protein (CRP) (milligrams per liter) were quantified using routine chemistry assays. Targeted metabolomics (proton nuclear magnetic resonance spectroscopy [24]) was also performed to quantify 230 cardiometabolic traits (150 concentrations plus 80 ratios), including the concentration, diameter, and cholesterol and triglyceride content of lipoprotein subclass particles, plus apolipoproteins, fatty and amino acids, ketones, and factors related to glycolysis and inflammation.

### Assessment of covariates

Demographic covariates included sex, ethnicity (white vs. nonwhite), age (in months) at the time of fat index assessment, and highest level of education attained by the participant’s mother as reported shortly after delivery (Certificate of Secondary Education, vocational, O-level, A-level, or degree, using English standards) to indicate socioeconomic position at birth. Smoking at 18 years of age was recorded via questionnaire (grouped as never smoked an entire cigarette, smokes less than weekly, and smokes every week). Alcohol consumption at 18 years of age was also recorded (grouped as never/monthly/less than monthly, 2 or 4 times per month, and 2 or more times per week). Puberty timing was estimated through age at peak height velocity, which describes the age at which the greatest increase in height occurred on the basis of superimposition by translation and rotation growth curve modeling (25) of up to 10 (median 8) height assessments from 5 to 20 years of age (Online Appendix).

### Statistical approach

We examined Pearson correlation coefficients for each index pairing at age 10 years, age 18 years, and change from ages 10 to 18 years. Correlations between repeated measures of each index were examined to estimate the stability of each measure. All indexes were standardized within occasions to SD units for analyses using *z*-scores. Cardiometabolic traits were also standardized to allow effect size comparability.

In the first set of models, we examined associations of BMI and fat mass index measured at age 10 years with cardiometabolic traits at age 18 years using linear regression with robust standard errors, adjusting for demographics (age, sex, ethnicity, and maternal education). We then examined mutually adjusted associations of each regional fat index (trunk, arm, and leg) at age 10 years in relation to these cardiometabolic traits. Lean mass index at age 10 years was then examined in relation to traits as described, with additional adjustment for fat mass index. Evidence of interaction was assessed by including product terms of fat and lean mass indexes in linear models in relation to cardiometabolic traits and examining the direction of interaction coefficients and their p values.

Second, we examined BMI and fat mass index measured at age 18 years in relation to these same cardiometabolic traits at age 18 years adjusting for demographics plus smoking, alcohol, and puberty timing. Differences in effect size for fat measures at 18 versus 10 years of age were assumed to reflect differences in duration of exposure to fat levels if correlations between repeated fat measures were high. These were repeated using regional fat indexes and lean mass index at age 18 years, as described.

Third, we examined changes in BMI and fat mass index on the basis of differences in standardized values from 10 to 18 years of age in relation to cardiometabolic traits at age 18 years. These adjusted for demographics plus initial BMI/fat mass index at age 10 years. We then examined mutually adjusted change in each regional fat index from 10 to 18 years of age and change in lean mass index adjusted for change in fat mass index in relation to cardiometabolic traits, again with each adjusted for its value at age 10 years.

### Supplementary analyses

We repeated main analyses (which had varying sample sizes) on a complete-case sample of participants with data on all variables (all fat measures on both occasions, covariates, and all cardiometabolic traits).

To examine potential nonlinear associations of index change, we examined tertiles of SD change of each fat and lean measure from 10 to 18 years of age in relation to summary cardiometabolic traits at age 18 years.

To compare the explanatory power of current versus change values of total and regional fat indexes for cardiometabolic traits, we compared the proportion of variance in each trait explained by separate (not mutually adjusted) linear regression models of each fat index at age 18 years versus change in each fat index from 10 to 18 years of age. Models were adjusted for a common set of basic covariates (age at cardiometabolic trait assessment, sex, ethnicity, and maternal education).

Seventeen principal components explain 95% of the variance in these highly correlated cardiometabolic traits on the basis of previous ALSPAC analyses (26); this can be used to correct nominal significance thresholds for multiple testing using the Bonferroni method (e.g., alpha = 0.05/17). Here, we focus on effect size and precision 27, 28. Analyses were conducted using Stata version 15.1 (StataCorp, College Station, Texas).

## Results

### Sample characteristics

A total of 2,840 participants were eligible for inclusion in ≥1 analysis at each time point (Figure 1). Of these, 55.3% were female and 3.7% of nonwhite ethnicities (Table 1). Mean BMI was 17.5 ± 2.7 kg/m^2^ at age 10 years (mean 9.8 years); 12.7% overall had obesity using BMI ≥ 20.1 kg/m^2^ among male subjects and BMI ≥ 21.0 kg/m^2^ among female subjects on the basis of 95th percentile values from a World Health Organization 2007 preadult reference population (29). Mean BMI was 22.7 ± 0.4 kg/m^2^ at age 18 years (mean 17.7 years); 10.5% overall had obesity using BMI ≥27.3 kg/m^2^ among male subjects and BMI ≥27.6 kg/m^2^ among female subjects (29). Excluded versus included participants had slightly higher BMI and age at the age 18 years clinic visit, were less likely to be female, and were more likely to have low maternal education (Online Table 1).

**Table 1:** Characteristics of Participants From the Avon Longitudinal Study of Parents and Children Offspring Cohort

Demographics		
Exact age (yrs) at 18 yrs clinic	2,840	17.7 ± 0.4
Female	2,840	55.3 (1,570)
Nonwhite ethnicity	2,840	3.7 (104)
Low maternal education∗	2,840	49.2 (1,396)
Currently smokes at age 18 yrs	2,840	17.0 (483)
Drinks on ≥2 days/week at age 18 yrs	2,840	25.1 (714)
Age at peak height velocity, yrs	2,840	12.6 ± 1.2
Indexes at age 10 yrs		
BMI, kg/m^2^	2,840	17.5 ± 2.7
Has obesity†	2,840	12.7 (361)
Fat mass index, kg/m^2^	2,840	4.2 ± 2.3
Trunk fat index, kg/m^2^	2,840	1.7 ± 1.1
Arm fat index, kg/m^2^	2,840	0.4 ± 0.2
Leg fat index, kg/m^2^	2,840	1.9 ± 0.9
Lean mass index, kg/m^2^	2,840	12.5 ± 1.0
Indexes at age 18 yrs		
BMI, kg/m^2^	2,840	22.7 ± 4.0
Has obesity†	2,840	10.5 (299)
Fat mass index, kg/m^2^	2,840	6.2 ± 3.7
Trunk fat index, kg/m^2^	2,840	3.2 ± 2.0
Arm fat index, kg/m^2^	2,840	0.5 ± 0.3
Leg fat index, kg/m^2^	2,840	2.3 ± 1.3
Lean mass index, kg/m^2^	2,840	15.4 ± 2.1
Summary cardiometabolic traits at age 18 yrs		
Systolic blood pressure, mm Hg	2,744	114.3 ± 9.7
Diastolic blood pressure, mm Hg	2,744	64.2 ± 6.2
Total cholesterol, mmol/l	1,895	3.5 ± 0.7
LDL cholesterol, mmol/l	1,895	1.0 ± 0.4
HDL cholesterol, mmol/l	1,895	1.4 ± 0.2
Triglycerides, mmol/l	1,895	0.9 ± 0.3
Insulin, mU/l	1,928	6.7 (0.3–170.4)
Glucose, mmol/l	1,894	4.1 ± 0.4
Glycoprotein acetyls, mmol/l	1,894	1.2 ± 0.1
C-reactive protein, mg/l	1,950	0.6 (0.02–86.5)

Values are n, mean ± SD, % (n), or median (range). Participants included in ≥1 analysis at each time point (n = 2,840). Participants described are those with data on BMI and dual-energy x-ray absorptiometry fat measures at 10 and 18 years of age, plus ≥1 cardiometabolic trait at 18 years, plus covariates.

BMI = body mass index; HDL = high-density lipoprotein; LDL = low-density lipoprotein.

∗ Low maternal education defined as Certificate of Secondary Education, vocational, or O-level versus A-level or degree.

† Obesity defined on the basis of 95th percentile values of BMI from a World Health Organization 2007 preadult reference population (29); at mean age 9.8 years, these are BMI ≥20.1 kg/m^2^ among male subjects and BMI ≥21.0 kg/m^2^ among female subjects; at mean age 17.7 years, these are BMI ≥27.3 kg/m^2^ among male subjects and BMI ≥27.6 kg/m^2^ among female subjects.

### Correlations among BMI and DXA fat indexes

Correlations between repeated measures at 10 and 18 years of age were approximately 0.7 for BMI, fat mass index, and regional fat indexes. At age 10 years, BMI was correlated with total and regional fat indexes at 0.9; this was 0.5 between BMI and lean mass index. Correlations were similar at age 18 years (e.g., 0.8 between BMI and fat mass index and 0.9 between BMI and trunk fat index). Change in BMI was more strongly correlated with change in each fat index (e.g., 0.9 with change in fat mass index and 0.9 with change in trunk fat index) than with change in lean mass index (0.4). All had p values <0.0001 and are shown in Online Figures 1 to 4.

### Associations of BMI and DXA fat indexes at age 10 years with cardiometabolic traits at age 18 years

**Figure 2:**
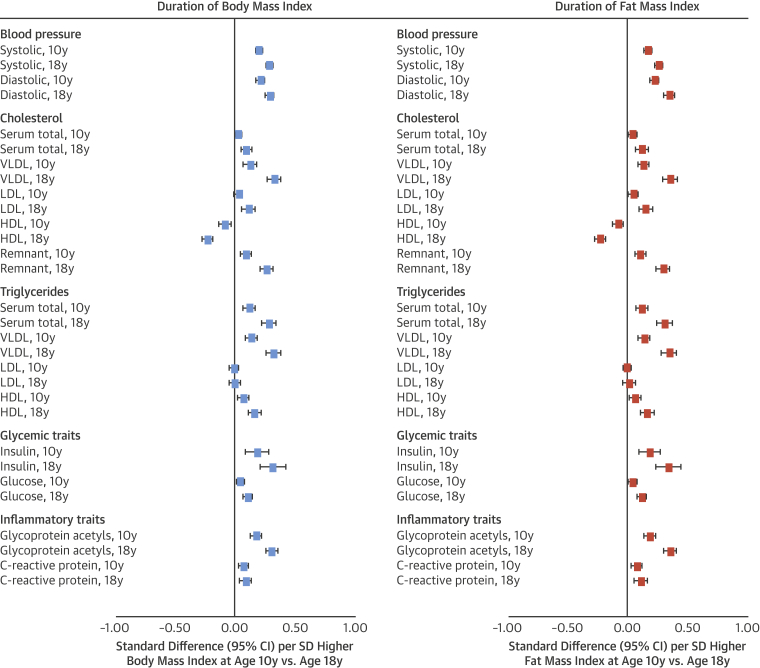
Duration of Exposure to Body Mass Index and Fat Mass Index in Relation to Blood Pressure, Cholesterol, Triglycerides, and Glycemic and Inflammatory Traits at Age 18 Years in the Avon Longitudinal Study of Parents and Children All outcomes are at age 18 years (“10y” and “18y” noted within outcome list refer to time of body mass index [BMI] or fat mass index measurement). Models for age 10 years exposures are adjusted for age (in months), sex, ethnicity, and maternal education. Models for age 18 years exposures are additionally adjusted for smoking, alcohol, and puberty timing. Estimates are standardized beta coefficients from linear regression models and are interpreted as the number of SDs from the mean of the outcome distribution per SD higher BMI or fat mass index. CI = confidence interval; HDL = high-density lipoprotein; LDL = low-density lipoprotein; VLDL = very-low-density lipoprotein.

Higher BMI at age 10 years was strongly associated with higher SBP, DBP, and cholesterol in very-low-density lipoprotein (VLDL), LDL, and remnant particles; with lower cholesterol in high-density lipoprotein (HDL) particles; and with higher triglycerides across all particles except LDL. Associations were strong with higher insulin, glucose, glycoprotein acetyls, and CRP. Associations of fat mass index with these traits closely resembled those of BMI (Figure 2). For example, each SD higher fat mass index was associated with 0.23 SD higher DBP (95% confidence interval [CI]: 0.20 to 0.27; p < 0.0001) versus 0.22 SD higher DBP (95% CI: 0.18 to 0.25; p < 0.0001) for BMI, with these measures explaining 7.1% versus 6.7% of trait variance, respectively.

**Figure 3:**
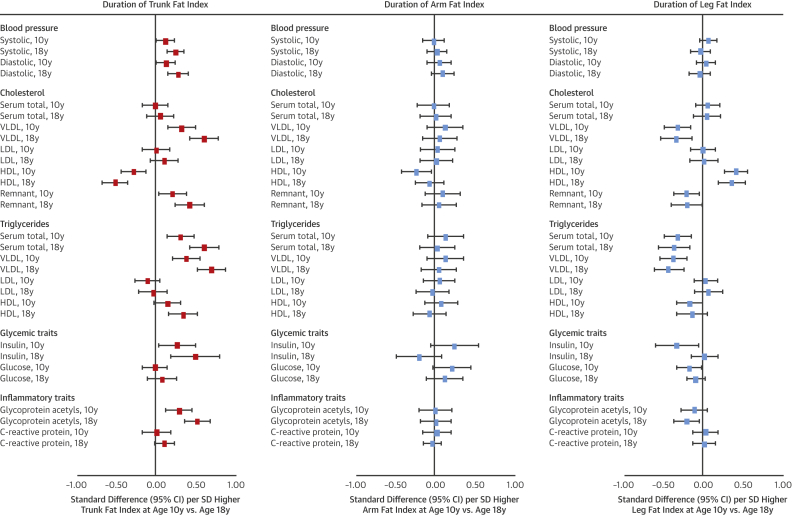
Duration of Exposure to Regional Fat Indexes in Relation to Blood Pressure, Cholesterol, Triglycerides, and Glycemic and Inflammatory Traits at Age 18 Years in the Avon Longitudinal Study of Parents and Children All outcomes are at age 18 years (“10y” and “18y” noted within outcome list refer to time of regional fat index measurement). Models for age 10 years exposures are adjusted for age (in months), sex, ethnicity, maternal education, and alternative regional fat measures. Models for age 18 years exposures are additionally adjusted for smoking, alcohol, and puberty timing. Estimates are standardized beta coefficients from linear regression models and are interpreted as the number of SDs from the mean of the outcome distribution per SD higher regional fat index. Abbreviations as in Figure 2.

Association patterns of trunk fat index at age 10 years with cardiometabolic traits closely resembled those of BMI and fat mass index (Figure 3). Effect sizes were double for many traits (e.g., 0.32 SD of total cholesterol in VLDL for trunk fat index vs. 0.14 SD for fat mass index). Effect sizes for arm fat index were smaller but directionally concordant with trunk fat index, while those for leg fat index were similarly large but directionally discordant (e.g., −0.31 SD; 95% CI: −0.47 to −0.15; p = 0.0002] of total cholesterol in VLDL per SD higher leg fat index). Evidence for heterogeneity across regional fat measures was strongest for VLDL- and HDL-related traits (typically p < 0.0001) and branched chain amino acids.

Higher lean mass index at age 10 years was most associated with higher SBP at 0.18 SD (95% CI: 0.14 to 0.21; p < 0.0001), with little attenuation upon adjustment for fat mass index at age 10 years. Attenuations were greater across lipid traits including cholesterol in VLDL. Evidence for interaction between fat and lean mass indexes at age 10 years in relation to most traits was weak (p = 0.001 to 0.99, median p = 0.31), with mostly positive interaction coefficients.

Comparable associations were seen between BMI and DXA fat indexes for lipoprotein subclasses, apolipoproteins, and clinical trait precursors including fatty acid ratios and branched chain and aromatic amino acids (full results in Online Tables 2, 3, 5, and 9).

### Associations of BMI and DXA fat indexes at age 18 years with cardiometabolic traits at age 18 years

BMI at age 18 years was more strongly associated with cardiometabolic traits at age 18 years than was BMI at age 10 years. For example, the coefficient rose from 0.13 to 0.33 SD for higher total cholesterol in VLDL and from 0.18 to 0.31 SD for glycoprotein acetyls. The pattern and magnitude of associations for fat mass index again closely resembled those of BMI (Figure 2), for example, 0.36 SD (95% CI: 0.31 to 0.41; p < 0.0001) of glycoprotein acetyls per SD higher fat mass index versus 0.31 SD (95% CI: 0.26 to 0.36; p < 0.0001) per SD higher BMI (full results in Online Table 2), with measures explaining 16.2% versus 15.4% of trait variance, respectively.

Among regional fat indexes, only trunk fat index at age 18 years showed larger effect sizes in relation to cardiometabolic traits at age 18 years versus its index at age 10 years (Figure 3). For example, the coefficients rose from 0.32 to 0.60 SD for total cholesterol in VLDL and from 0.38 to 0.69 SD for triglycerides in VLDL. Evidence for heterogeneity across regional fat measures was again strongest for VLDL- and HDL-related traits (typically p < 0.0001) and amino acids.

Effect sizes for lean mass index at age 18 years were often double those at age 10 years and showed attenuation upon adjustment for fat mass index (e.g., from 0.14 to 0.06 SD for total cholesterol in VLDL). Evidence for interaction between fat and lean mass indexes at age 18 years for most traits was strongest for VLDL-related traits (e.g., p = 0.0003 for triglycerides in VLDL), but interaction coefficients were positive, and evidence was weaker across wider traits (p < 0.0001 to 0.99, median p = 0.12).

Comparable associations were seen between BMI and DXA fat indexes for lipoprotein subclasses, apolipoproteins, and clinical trait precursors, including fatty acid ratios and branched chain and aromatic amino acids; these again tended to be higher in magnitude versus the earlier assessment (full results in Online Tables 2, 4, 5, and 9).

### Associations of change in BMI and DXA fat indexes from 10 to 18 years of age with cardiometabolic traits at age 18 years

**Figure 4:**
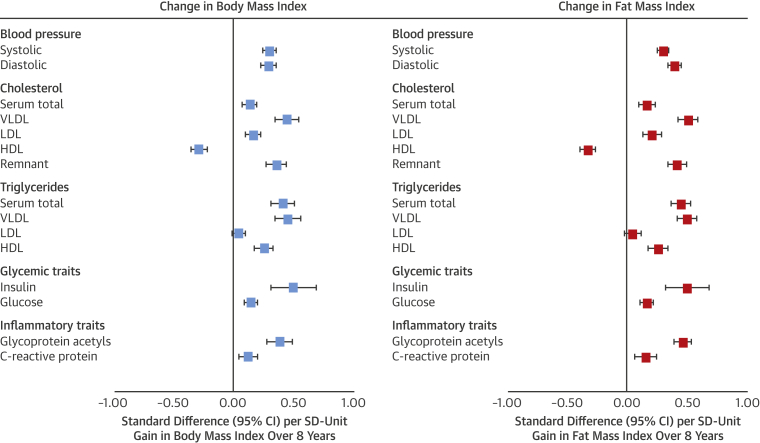
Change in Body Mass Index and Fat Mass Index From 10 to 18 Years of Age in Relation to Blood Pressure, Cholesterol, Triglycerides, and Glycemic and Inflammatory Traits at Age 18 Years in the Avon Longitudinal Study of Parents and Children Models are adjusted for age (in months) at age 10 years, sex, ethnicity, maternal education, and BMI or fat mass index at age 10 years. Estimates are standardized beta coefficients from linear regression models and are interpreted as number of SDs from the mean of the outcome distribution per SD unit gain in BMI or fat mass index. Abbreviations as in Figure 2.

Gains in BMI from 10 to 18 years of age were more strongly associated with higher SBP and DBP; higher total cholesterol in serum and in VLDL, LDL, and remnant particles; lower cholesterol in HDL particles; and higher triglycerides in all particle types besides LDL. Associations were also strong with higher insulin, glucose, and glycoprotein acetyls and CRP. The pattern and magnitude of associations for gains in fat mass index closely resembled those of gains in BMI (Figure 4), for example, 0.45 SD (95% CI: 0.38 to 0.53; p < 0.0001) of glycoprotein acetyls per SD unit gain in fat mass index versus 0.38 SD (95% CI: 0.27 to 0.48; p < 0.0001) per SD unit gain in BMI, with measures explaining 16.8% versus 15.7% of trait variance, respectively.

**Figure 5:**
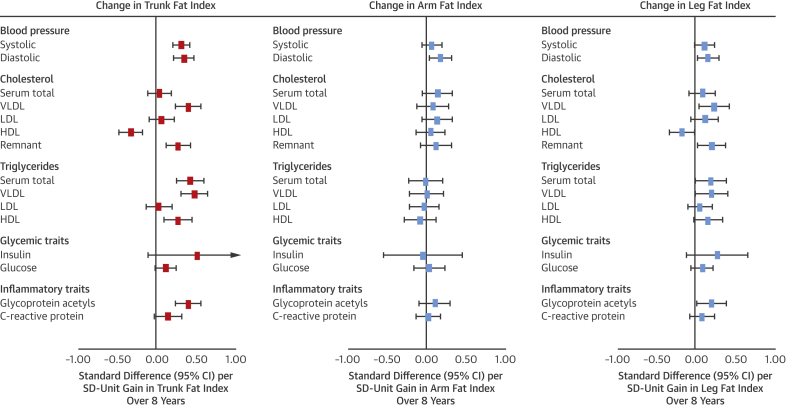
Change in Regional Fat Indexes From 10 to 18 Years of Age in Relation to Blood Pressure, Cholesterol, Triglycerides, and Glycemic and Inflammatory Traits at Age 18 Years in Avon Longitudinal Study of Parents and Children Models are adjusted for age (in months) at age 10 years, sex, ethnicity, maternal education, change in alternative regional fat indexes from 10 to 18 years of age, and regional fat index exposure at age 10 years. Estimates are standardized beta coefficients from linear regression models and are interpreted as the number of SDs from the mean of the outcome distribution per SD unit gain in regional fat index. Abbreviations as in Figure 2.

Among regional fat measures, associations of gains in trunk fat index most resembled associations of gains in BMI and fat mass index (Figure 5). Effect size for gains in trunk fat index was largest for insulin at 0.51 SD (95% CI: −0.09 to 1.10; p = 0.10), albeit with lower precision from higher skewedness. Associations for gains in leg fat index showed more directional concordance with gains in trunk fat index than gains in arm fat index for cholesterol and triglycerides across particle types. Evidence was weaker for heterogeneity across regional fat measures but remained strongest for VLDL- and HDL-related traits.

Effect sizes were also larger for change in lean mass index versus its cross-sectional assessments but were also substantially attenuated upon adjustment for change in fat mass index. Evidence for interaction between change in fat and lean mass indexes for traits was generally weak across traits (p = 0.002 to 0.99, median p = 0.22), with positive interaction coefficients.

Comparably strong associations were seen between gains in BMI and gains in DXA fat indexes in relation to lipoprotein subclasses, apolipoproteins, and clinical trait precursors, including fatty acid ratios and branched chain and aromatic amino acids (Online Figures 5 and 6; full results in Online Tables 6 to 9).

### Supplementary analyses

Repetition of analyses described previously in a complete-case sample of 1,722 participants gave similar association directions and magnitudes (Online Tables 2 to 9).

Higher tertiles of gains in BMI and fat indexes were comparably associated with summary cardiometabolic traits (Online Table 10). Associations for SBP, DBP, total cholesterol in HDL, triglycerides, and glycoprotein acetyls most reflected those of gains in trunk fat index; those for glucose reflected those of gains in leg fat index; those for total cholesterol in serum and LDL and CRP reflected gains in arm fat index; and those for insulin reflected gains in all fat indexes. Lean mass index change tertiles were less associated with traits.

Current values of BMI and fat mass index explained approximately double the variance in blood pressure, cholesterol, and triglycerides in VLDL, insulin, and glycoprotein acetyls than did change values of these indexes (Online Table 11). For example, variance explained by current BMI versus change in BMI was 8.8% versus 5.7% for total cholesterol in VLDL, 10.5% versus 5.7% for insulin, and 15.2% versus 9.8% for glycoprotein acetyls, respectively. In contrast, current and change values of these indexes explained similar amounts of variance in cholesterol and triglycerides in intermediate-density lipoprotein, LDL, and HDL; glucose; and fatty and amino acids. The same pattern was observed for regional fat indexes (Online Table 12).

## Discussion

In this study we compared BMI, the most widely used measure of body fatness, with more objective fat indexes in their associations with cardiometabolic traits relevant to CHD (Central Illustration). We examined duration of exposure to and change in BMI and DXA fat measures over 8 years in relation to detailed traits from targeted metabolomics. Our results suggest that higher fat mass index and BMI are similarly associated with higher blood pressure, higher VLDL and LDL cholesterol, lower HDL cholesterol, higher triglycerides, and higher glycemic and inflammatory traits, plus clinical trait precursors including branched chain and aromatic amino acids. These patterns closely resembled those seen for trunk fat index. Higher lean mass index was weakly associated with traits and did not appear to protect against effects of higher fatness. Altogether, the results support abdominal fatness as a primary driver of cardiometabolic dysfunction and BMI as a useful tool for detecting its effects.

**Central Illustration:**
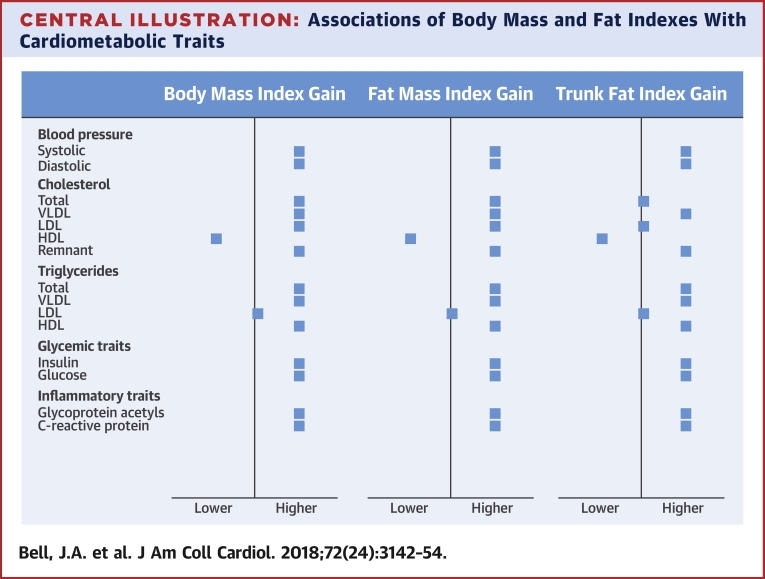
Associations of Body Mass and Fat Indexes With Cardiometabolic Traits Gains in body mass index and dual-energy x-ray absorptiometry–derived total and regional (trunk, arm, leg) fat indexes from 10 to 18 years of age were examined in relation to cardiometabolic traits from clinical assessments and targeted metabolomics at age 18 years among 2,840 offspring from the ALSPAC (Avon Longitudinal Study of Parents and Children) cohort. Models were adjusted for age, sex, ethnicity, maternal education, and index level at age 10 years. Trunk fat index gain was additionally adjusted for change in arm and leg fat indexes. Associations were similar in direction and magnitude for the 3 indexes shown and were weaker for gains in arm and leg fat indexes. HDL = high-density lipoprotein; LDL = low-density lipoprotein; VLDL = very-low-density lipoprotein.

Duration of fat exposure was assessed using a simple age comparison whereby fat measures were taken at 10 and 18 years of age, and effect sizes were compared in relation to cardiometabolic traits assessed at age 18 years, the latter fat measure assumed to capture more time exposed to tissue levels given sufficiently high correlations between repeated fat measures over time (0.7 here). Effect sizes increased with time for virtually all traits, especially for total VLDL cholesterol and triglyceride content. Trunk fat index was the regional measure most resembling these patterns and was the only one adversely influenced by longer exposure. Higher leg fat, in contrast, appeared beneficial for traits at each time point, particularly for cholesterol and triglyceride profiles.

Patterns for BMI and fat mass index were reinforced through analyses of gains over 8 years with strongly adverse associations with cardiometabolic traits at age 18 years, again with comparable effect sizes between measures. Among these were higher LDL and remnant cholesterol and higher blood pressure and glucose, which are likely causal for CHD (30) and which together mediate much of the CHD risk conferred by higher BMI (6). Gains in BMI and fat mass index were also similarly associated with higher inflammatory glycoprotein acetyls and fasting insulin, which marks insulin resistance, drives type 2 diabetes (11), and amplifies CHD and mortality risk (31). Once again, these associations closely resembled those for gains in trunk fat index, suggesting that effects are driven largely by abdominal fat gain. To a lesser extent, gains in leg fat were associated adversely with cholesterol and triglyceride content, suggesting that although higher leg fat appears beneficial using one-off measures, fat gain is not beneficial in any region.

The striking resemblance of BMI with total and trunk fat indexes in associations with cardiometabolic traits reflects the strong correlations between measures in this study (all about 0.9). The correlation between change in BMI was also highest with change in trunk fat index (at 0.9 vs. 0.8 for arm and leg fat indexes), indicating that although most fat gain occurs in the trunk, BMI is highly capable of detecting its effects, at least in young populations. Although current levels of total and regional fat indexes and their changes over time were both strongly associated with cardiometabolic traits, current levels showed greater explanatory power by way of more variance explained in several important traits, including blood pressure, cholesterol and triglycerides in VLDL, insulin, and inflammatory glycoprotein acetyls. Current index levels and their changes had similar explanatory power for other traits, including cholesterol and triglycerides in LDL and HDL, and glucose. This suggests that knowing one’s current level of fatness is at least as informative as knowing one’s change in fatness over time for intermediates of CHD. This has welcome implications given the relative ease of measuring current fatness versus change in fatness in clinical settings.

Higher lean mass index was less associated with cardiometabolic traits. Irrespective of change in fat mass index, gains in lean mass index were associated most strongly with higher SBP and creatinine, a protein-like marker of muscle mass and kidney function. Findings support those of a previous cross-sectional study of adolescents suggesting paradoxically adverse associations of higher lean mass index with lower HDL cholesterol, higher blood pressure, and higher insulin (15). Attenuations were substantial upon adjustment for fat mass index, however, indicating confounding by accompanying fat gain. Effect sizes were also smaller than for fat mass index. Lower grip strength, an indirect measure of lower lean mass, has been associated with higher cardiovascular- and all-cause mortality risk among adults independent of fat mass 32, 33. One recent study using UK Biobank data suggested independent associations of one-off measures of higher fat mass and lower grip strength with mortality (34), supporting both fat loss and muscle gain as priorities for longevity. Our results suggest that priorities are unlikely equal with respect to type 2 diabetes and CHD risk, because causal intermediates track more closely alongside total fat than lean mass, and higher leanness did not appear protective against higher fatness. Evidence for interaction was generally weak, and importantly, effects operated in the same direction; that is, higher leanness appeared to amplify, not reduce, effects of higher fatness. Such positive interactions are, in any case, sensitive to measurement scaling and considered less informative than crossover interactions 35, 36. An important caveat of this study with regard to lean mass is its focus on total “resting” mass and not dynamic properties of lean tissue in response to physical activity. This is important because key benefits of skeletal muscle are transient, such as anti-inflammatory myokine release through contractile functions 12, 13. Moderate correlations do exist between muscle mass, quality, and strength (37), however, and so higher resting lean mass may reflect more regular contractile activity. Correlations between fat and lean mass indexes here were low to moderately positive, possibly reflecting low overall fatness in the study population (12.7% and 10.5% had obesity at 10 and 18 years of age, respectively), and downward pressure by fat on muscle volume beyond the minimum needed for mobility. Higher fatness also reduces habitual physical activity 38, 39, which may in turn reduce muscle mass.

Patterns of fat distribution are known to differ by sex, with men tending to store fat more abdominally and women more peripherally, resulting in higher visceral-to-subcutaneous fat volumes and insulin resistance in men (17). Higher fatness is therefore expected to affect cardiometabolic traits such as insulin and LDL cholesterol more strongly among men. Importantly, sex differences are also expected to be directionally concordant, referring to magnitude, not existence, of associations.

### Study strengths

Strengths of this study include measures of fat and lean mass from DXA scans, which correlate well with those based on more resonant (and expensive) computed tomography (40). We had the rare opportunity of comparing the magnitude of associations of BMI and DXA-based indexes measured in childhood versus young adulthood with traits from targeted metabolomics, which allowed us to distinguish their associations with cholesterol and triglyceride content and clinical trait precursors in greater detail than previously possible. This also allowed us to address questions of both duration of exposure to and change in each tissue type. Participants were young and generally lean, thus minimizing distortion of associations by pre-existing chronic diseases and behaviors such as smoking and alcohol use, which are more common at older ages. This is important given complex bidirectional relations between smoking and adiposity, with smoking leading to decreased BMI (41) and higher BMI leading to increased smoking (42). Investigations among adult populations should consider how this important confounder influences fat mass. Post-pubertal analyses were adjusted for an objective height-based measure of puberty timing because earlier puberty onset is associated with higher subsequent fatness and cardiometabolic traits (43). This had little effect on results, possibly because puberty timing effects are largely confounded by pre-pubertal adiposity (44) and would thus overlap with fat measures examined here as exposures.

### Study limitations

Limitations of this study include the observational nature of the data, which are subject to residual confounding and reverse causation bias; robust genetic proxies for more objective fat and lean mass are not currently available for Mendelian randomization analyses. Change scores of fat indexes may be more prone to measurement error than one-off measures; this could partly explain inferior explanatory power of change versus current values of fat indexes for some cardiometabolic traits. Participants were also predominantly white and European, limiting inference to other ethnic groups. DXA measures do not distinguish histological subtypes of fat (e.g., visceral from subcutaneous). Visceral fat is known to drive cardiometabolic effects 10, 11, but visceral fat volume does vary alongside BMI in populations and so is likely captured by indirect measures (45). We did not adjust for physical activity because instrumental variable analyses suggest that BMI influences activity more than activity influences BMI 38, 39, positioning physical activity as a mediator, not a confounder, of associations. Cardiorespiratory fitness would also be a mediator because it is measured as a function of weight (e.g., maximum oxygen intake in milliliters per kilogram per minute). “Fitness versus fatness” debates are likely misguided.

## Conclusions

Our results suggest that BMI and DXA fat mass index are similarly associated with cardiometabolic traits relevant to CHD. These include blood pressure, VLDL and LDL cholesterol, triglycerides, and glycemic and inflammatory traits, plus clinical trait precursors such as branched chain amino acids. Patterns closely resemble those for trunk fat index, as reinforced through analyses of duration of exposure to and change in each fat measure across adolescence. Higher leanness is less associated with these traits and does not appear to protect against higher fatness. Altogether, the results support abdominal fatness as a primary driver of cardiometabolic dysfunction and BMI as a useful tool for detecting its effects.

Perspectives **COMPETENCY IN MEDICAL KNOWLEDGE:** Although BMI does not address fat distribution or distinguish fat from lean mass, it can be used to detect subclinical cardiometabolic abnormalities, which are closely related to abdominal obesity. Higher lean mass is not likely to protect against the cardiometabolic consequences of obesity.

**TRANSLATIONAL OUTLOOK:** It is reasonable to rely on BMI as an indirect measure of total body and abdominal fatness in future investigations of disease etiology including large-scale Mendelian randomization studies.
