# Supplementary material for: Associations of Body Mass and Fat Indexes With Cardiometabolic Traits
Source: J Am Coll Cardiol. 2018 Dec 18;72(24):3142–54. doi: 10.1016/j.jacc.2018.09.066 (PMC6290112; doi:10.1016/j.jacc.2018.09.066)
Supplement: Online Data [file mmc1.pdf]

Online Appendix for *Associations of Body Mass and Fat Indexes With Cardiometabolic Traits*

Estimating age at peak height velocity (PHV)

A random subsample of 10% of ALSPAC participants were invited to clinics between birth and age 5 years, and all children were invited to clinics at ages 7, 9, 10, 11, 13, 15 and 17 years. Height was measured in light clothing without shoes to the nearest 0.1 cm by a trained fieldworker. For the current analysis height measurements collected between age 5 to 20 years were included. These data were further restricted to include individuals with at least one height measurement for the following time points: 5 to <10 years, 10 to < 15 years and 15 to 20 years. A total of 46,246 height measurements for 5,707 individuals (3,019 females, 2,688 males) were available for analysis.

Super-Imposition by Translation And Rotation (SITAR) analysis was implemented in R version 3.4.1 in order to estimate age at PHV. SITAR is a shape-invariant growth model used to summarize pubertal growth by fitting a random effects model to a set of height growth curves. The estimated parameters: size, tempo (based on age at PHV) and velocity describe how each individual differs from the mean curve. This method has been described in detail previously [1]. For the purposes of this paper only the estimate of age at PHV was used.

Data were uploaded in R for initial model fitting and outliers with velocity exceeding 4 SDs and standardized residuals exceeding 4 in absolute value, were removed. A total of 40,037 height measurements for 5,707 individuals with an average of 8 (and up to 10) measurements per participant were available for analysis. The final SITAR model was fitted with 5 degrees of freedom, for males and females separately, explaining 98% and 98.3% of variance in males and females, respectively. Mean (SD) age at PHV was 13.6 (0.9) years among males (**Figure 1**) and 11.7 (0.8) years among females (**Figure 2**).

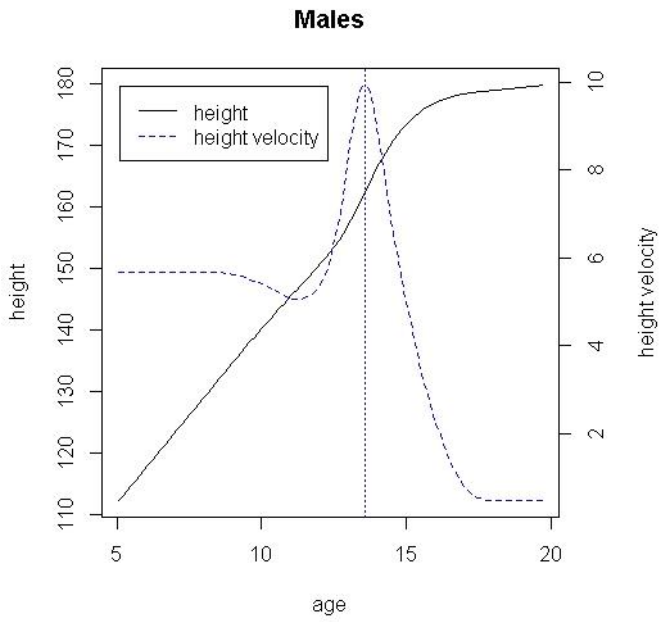

Figure 1 Mean growth curve (solid line) and velocity (dashed line) plots estimated by SITAR for males. Vertical dotted line represents age at peak height velocity.

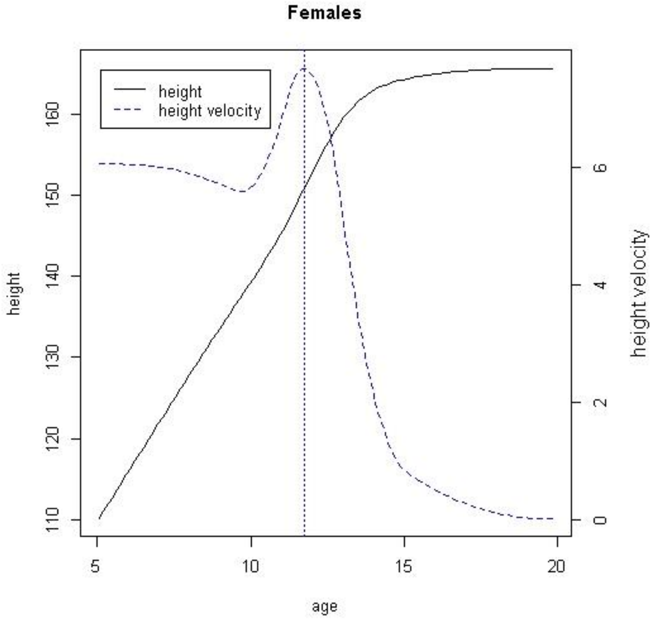

Figure 2 Mean growth curve (solid line) and velocity (dashed line) plots estimated by SITAR for females. Vertical dotted line represents age at peak height velocity.

References

1. Cole TJ, Donaldson MD, Ben-Shlomo Y. SITAR—a useful instrument for growth curve analysis. Int J Epidemiol. 2010;39(6):1558-66.

Online Table 1 Characteristics of included and excluded participants in ALSPAC

	n	**Included in ≥ 1 analysis at each time point***	*n*	**Excluded from analyses**	*P-value for difference*
Demographics					
Exact age (years) at 18y clinic – mean (SD)	2840	17.7 (0.4)	2376	17.9 (0.5)	<0.0001
Female – n (%)	2840	1570 (55.3)	12014	5649 (47.0)	<0.0001
Non-white ethnicity – n (%)	2840	104 (3.7)	9310	509 (5.5)	0.0001
Low maternal education – n (%)	2840	1396 (49.2)	9653	6687 (69.3)	<0.0001
Currently smokes at age 18y - n (%)	2840	483 (17.0)	1359	233 (17.1)	0.912
Drinks on 2+ days/week at age 18y - n (%)	2840	714 (25.1)	1131	298 (26.4)	0.431
Age at peak height velocity - mean (SD)	2840	12.6 (1.2)	2864	12.6 (1.3)	0.004
Indexes at age 10y					
Body mass index (kg/m^2) – mean (SD)	2840	17.5 (2.7)	4795	17.8 (3.0)	<0.0001
Fat mass index (kg/m^2) – mean (SD)	2840	4.2 (2.3)	4424	4.4 (2.5)	0.012
Trunk fat index (kg/m^2) - mean (SD)	2840	1.7 (1.1)	4424	1.8 (1.2)	0.005
Arm fat index (kg/m^2) - mean (SD)	2840	0.4 (0.2)	4424	0.4 (0.3)	0.016
Leg fat index (kg/m^2) - mean (SD)	2840	1.9 (0.9)	4424	2.0 (1.0)	0.033
Lean mass index (kg/m^2) – mean (SD)	2840	12.5 (1.0)	4424	12.6 (1.0)	<0.0001
Indexes at age 18y					
Body mass index (kg/m^2) – mean (SD)	2840	22.7 (4.0)	2223	23.1 (4.5)	0.0001
Fat mass index (kg/m^2) – mean (SD)	2840	6.2 (3.7)	1988	6.6 (3.9)	0.002
Trunk fat index (kg/m^2) - mean (SD)	2840	3.2 (2.0)	1988	3.4 (2.1)	0.002
Arm fat index (kg/m^2) - mean (SD)	2840	0.5 (0.3)	1988	0.5 (0.3)	0.006
Leg fat index (kg/m^2) - mean (SD)	2840	2.3 (1.3)	1988	2.4 (1.4)	0.002
Lean mass index (kg/m^2) – mean (SD)	2840	15.4 (2.1)	1988	15.4 (2.2)	0.975

*Participants described are those with data on BMI and DXA fat measures at age 10y and 18y, plus ≥ 1 cardiometabolic trait at 18y, plus all covariates
P-values are based on linear or logistic regression models.

Online Table 2 Associations of body mass index (BMI) and fat mass index at age 10y and 18y with cardiometabolic traits at age 18y in ALSPAC

	At age 10y										At age 18y									
	BMI (per 2.7 kg/m^2 higher)					Fat mass index (per 2.3 kg/m^2 higher)					BMI (per 4.0 kg/m^2 higher)					Fat mass index (per 3.7 kg/m^2 higher)				
	Adj. for age, sex, ethnicity, maternal education					*Adj. for age, sex, ethnicity, maternal education*					*Adj. for age, sex, ethnicity, maternal education, smoking, alcohol, puberty timing*					*Adj. for age, sex, ethnicity, maternal education, smoking, alcohol, puberty timing*				
**Standardized outcome at age 18y**	**N**	**Beta**	**LCL**	**UCL**	**P-value**	**N**	**Beta**	**LCL**	**UCL**	**P-value**	**N**	**Beta**	**LCL**	**UCL**	**P-value**	**N**	**Beta**	**LCL**	**UCL**	**P-value**
Systolic blood pressure (mmHg)	3583	0.20	0.17	0.23	<0.0001	3548	0.17	0.14	0.21	<0.0001	3069	0.29	0.25	0.32	<0.0001	3016	0.26	0.23	0.30	<0.0001
Diastolic blood pressure (mmHg)	3583	0.22	0.18	0.25	<0.0001	3548	0.23	0.20	0.27	<0.0001	3069	0.29	0.26	0.33	<0.0001	3016	0.35	0.31	0.39	<0.0001
Concentration of chylomicrons and extremely large VLDL particles (mol/l)	2456	0.15	0.10	0.20	<0.0001	2440	0.16	0.11	0.21	<0.0001	2093	0.34	0.29	0.40	<0.0001	2058	0.39	0.32	0.45	<0.0001
Total lipids in chylomicrons and extremely large VLDL (mmol/l)	2456	0.15	0.10	0.20	<0.0001	2440	0.16	0.11	0.20	<0.0001	2093	0.34	0.28	0.40	<0.0001	2058	0.38	0.31	0.44	<0.0001
Phospholipids in chylomicrons and extremely large VLDL (mmol/l)	2456	0.15	0.10	0.20	<0.0001	2440	0.15	0.11	0.20	<0.0001	2093	0.33	0.27	0.39	<0.0001	2058	0.37	0.31	0.44	<0.0001
Total cholesterol in chylomicrons and extremely large VLDL (mmol/l)	2456	0.14	0.10	0.19	<0.0001	2440	0.15	0.10	0.20	<0.0001	2093	0.34	0.28	0.40	<0.0001	2058	0.37	0.31	0.44	<0.0001
Cholesterol esters in chylomicrons and extremely large VLDL (mmol/l)	2456	0.14	0.09	0.19	<0.0001	2440	0.14	0.09	0.19	<0.0001	2093	0.33	0.28	0.39	<0.0001	2058	0.36	0.30	0.43	<0.0001
Free cholesterol in chylomicrons and extremely large VLDL (mmol/l)	2456	0.15	0.10	0.20	<0.0001	2440	0.15	0.10	0.20	<0.0001	2093	0.33	0.27	0.39	<0.0001	2058	0.37	0.31	0.43	<0.0001
Triglycerides in chylomicrons and extremely large VLDL (mmol/l)	2456	0.15	0.10	0.20	<0.0001	2440	0.16	0.11	0.20	<0.0001	2093	0.34	0.28	0.40	<0.0001	2058	0.38	0.31	0.44	<0.0001
Concentration of very large VLDL particles (mol/l)	2456	0.15	0.10	0.20	<0.0001	2440	0.15	0.10	0.20	<0.0001	2093	0.34	0.28	0.40	<0.0001	2058	0.37	0.31	0.44	<0.0001
Total lipids in very large VLDL (mmol/l)	2456	0.15	0.10	0.20	<0.0001	2440	0.15	0.10	0.20	<0.0001	2093	0.33	0.28	0.39	<0.0001	2058	0.37	0.30	0.43	<0.0001
Phospholipids in very large VLDL (mmol/l)	2456	0.14	0.10	0.19	<0.0001	2440	0.15	0.10	0.20	<0.0001	2093	0.33	0.27	0.39	<0.0001	2058	0.37	0.30	0.43	<0.0001
Total cholesterol in very large VLDL (mmol/l)	2456	0.15	0.10	0.20	<0.0001	2440	0.16	0.11	0.20	<0.0001	2093	0.35	0.29	0.41	<0.0001	2058	0.38	0.32	0.45	<0.0001
Cholesterol esters in very large VLDL (mmol/l)	2456	0.15	0.10	0.20	<0.0001	2440	0.16	0.11	0.21	<0.0001	2093	0.36	0.30	0.41	<0.0001	2058	0.39	0.32	0.45	<0.0001
Free cholesterol in very large VLDL (mmol/l)	2456	0.15	0.10	0.20	<0.0001	2440	0.15	0.10	0.20	<0.0001	2093	0.34	0.28	0.40	<0.0001	2058	0.38	0.31	0.44	<0.0001
Triglycerides in very large VLDL (mmol/l)	2456	0.15	0.10	0.19	<0.0001	2440	0.15	0.10	0.20	<0.0001	2093	0.33	0.27	0.39	<0.0001	2058	0.36	0.30	0.43	<0.0001
Concentration of large VLDL particles (mol/l)	2456	0.14	0.10	0.19	<0.0001	2440	0.15	0.10	0.20	<0.0001	2093	0.33	0.27	0.39	<0.0001	2058	0.36	0.29	0.42	<0.0001
Total lipids in large VLDL (mmol/l)	2456	0.15	0.10	0.19	<0.0001	2440	0.15	0.10	0.20	<0.0001	2093	0.33	0.27	0.39	<0.0001	2058	0.36	0.29	0.42	<0.0001
Phospholipids in large VLDL (mmol/l)	2456	0.14	0.09	0.19	<0.0001	2440	0.15	0.10	0.20	<0.0001	2093	0.33	0.27	0.39	<0.0001	2058	0.36	0.29	0.42	<0.0001
Total cholesterol in large VLDL (mmol/l)	2456	0.14	0.10	0.19	<0.0001	2440	0.15	0.10	0.20	<0.0001	2093	0.34	0.28	0.40	<0.0001	2058	0.37	0.30	0.43	<0.0001
Cholesterol esters in large VLDL (mmol/l)	2456	0.15	0.10	0.20	<0.0001	2440	0.15	0.10	0.20	<0.0001	2093	0.34	0.29	0.40	<0.0001	2058	0.37	0.31	0.44	<0.0001
Free cholesterol in large VLDL (mmol/l)	2456	0.14	0.09	0.19	<0.0001	2440	0.15	0.10	0.20	<0.0001	2093	0.33	0.27	0.39	<0.0001	2058	0.36	0.29	0.42	<0.0001
Triglycerides in large VLDL (mmol/l)	2456	0.14	0.10	0.19	<0.0001	2440	0.15	0.10	0.20	<0.0001	2093	0.32	0.27	0.38	<0.0001	2058	0.35	0.29	0.42	<0.0001
Concentration of medium VLDL particles (mol/l)	2456	0.15	0.10	0.20	<0.0001	2440	0.15	0.10	0.20	<0.0001	2093	0.34	0.28	0.39	<0.0001	2058	0.37	0.30	0.43	<0.0001
Total lipids in medium VLDL (mmol/l)	2456	0.15	0.10	0.20	<0.0001	2440	0.15	0.10	0.20	<0.0001	2093	0.34	0.28	0.40	<0.0001	2058	0.37	0.30	0.43	<0.0001
Phospholipids in medium VLDL (mmol/l)	2456	0.14	0.09	0.19	<0.0001	2440	0.15	0.10	0.20	<0.0001	2093	0.33	0.27	0.39	<0.0001	2058	0.36	0.29	0.42	<0.0001
Total cholesterol in medium VLDL (mmol/l)	2456	0.14	0.09	0.19	<0.0001	2440	0.14	0.09	0.19	<0.0001	2093	0.33	0.28	0.39	<0.0001	2058	0.36	0.30	0.42	<0.0001
Cholesterol esters in medium VLDL (mmol/l)	2456	0.13	0.08	0.18	<0.0001	2440	0.13	0.09	0.18	<0.0001	2093	0.33	0.28	0.39	<0.0001	2058	0.35	0.29	0.42	<0.0001
Free cholesterol in medium VLDL (mmol/l)	2456	0.14	0.09	0.19	<0.0001	2440	0.14	0.09	0.19	<0.0001	2093	0.32	0.26	0.38	<0.0001	2058	0.35	0.28	0.41	<0.0001
Triglycerides in medium VLDL (mmol/l)	2456	0.15	0.10	0.20	<0.0001	2440	0.15	0.10	0.20	<0.0001	2093	0.34	0.28	0.39	<0.0001	2058	0.37	0.30	0.43	<0.0001
Concentration of small VLDL particles (mol/l)	2456	0.12	0.07	0.17	<0.0001	2440	0.13	0.08	0.18	<0.0001	2093	0.30	0.25	0.36	<0.0001	2058	0.33	0.27	0.39	<0.0001
Total lipids in small VLDL (mmol/l)	2456	0.12	0.07	0.17	<0.0001	2440	0.13	0.08	0.18	<0.0001	2093	0.31	0.25	0.36	<0.0001	2058	0.34	0.28	0.40	<0.0001
Phospholipids in small VLDL (mmol/l)	2456	0.11	0.06	0.16	<0.0001	2440	0.12	0.07	0.16	<0.0001	2093	0.28	0.23	0.33	<0.0001	2058	0.31	0.25	0.37	<0.0001
Total cholesterol in small VLDL (mmol/l)	2456	0.11	0.06	0.16	<0.0001	2440	0.12	0.07	0.17	<0.0001	2093	0.29	0.24	0.35	<0.0001	2058	0.33	0.27	0.39	<0.0001
Cholesterol esters in small VLDL (mmol/l)	2456	0.11	0.06	0.15	<0.0001	2440	0.12	0.07	0.16	<0.0001	2093	0.29	0.24	0.34	<0.0001	2058	0.32	0.27	0.38	<0.0001
Free cholesterol in small VLDL (mmol/l)	2456	0.10	0.06	0.15	<0.0001	2440	0.11	0.07	0.16	<0.0001	2093	0.27	0.22	0.33	<0.0001	2058	0.30	0.24	0.36	<0.0001
Triglycerides in small VLDL (mmol/l)	2456	0.12	0.07	0.17	<0.0001	2440	0.13	0.08	0.17	<0.0001	2093	0.29	0.24	0.35	<0.0001	2058	0.32	0.26	0.38	<0.0001
Concentration of very small VLDL particles (mol/l)	2456	0.05	0.01	0.10	0.013	2440	0.06	0.02	0.11	0.005	2093	0.17	0.12	0.22	<0.0001	2058	0.19	0.14	0.25	<0.0001
Total lipids in very small VLDL (mmol/l)	2456	0.07	0.02	0.11	0.003	2440	0.08	0.03	0.12	0.001	2093	0.20	0.15	0.25	<0.0001	2058	0.23	0.17	0.29	<0.0001
Phospholipids in very small VLDL (mmol/l)	2456	0.04	0.00	0.08	0.060	2440	0.05	0.01	0.09	0.020	2093	0.14	0.09	0.19	<0.0001	2058	0.17	0.11	0.23	<0.0001
Total cholesterol in very small VLDL (mmol/l)	2456	0.07	0.02	0.11	0.003	2440	0.08	0.03	0.13	0.001	2093	0.20	0.14	0.25	<0.0001	2058	0.22	0.16	0.28	<0.0001
Cholesterol esters in very small VLDL (mmol/l)	2456	0.08	0.04	0.13	0.001	2440	0.09	0.05	0.14	0.0001	2093	0.22	0.17	0.27	<0.0001	2058	0.25	0.19	0.31	<0.0001
Free cholesterol in very small VLDL (mmol/l)	2456	0.03	-0.01	0.07	0.163	2440	0.04	0.00	0.08	0.063	2093	0.12	0.06	0.17	<0.0001	2058	0.13	0.07	0.19	<0.0001
Triglycerides in very small VLDL (mmol/l)	2456	0.07	0.02	0.11	0.003	2440	0.07	0.03	0.11	0.001	2093	0.18	0.13	0.24	<0.0001	2058	0.21	0.15	0.26	<0.0001
Concentration of IDL particles (mol/l)	2456	0.02	-0.02	0.07	0.256	2440	0.03	-0.01	0.08	0.127	2093	0.10	0.05	0.15	<0.0001	2058	0.13	0.07	0.18	<0.0001
Total lipids in IDL (mmol/l)	2456	0.03	-0.01	0.07	0.136	2440	0.04	0.00	0.09	0.052	2093	0.12	0.07	0.17	<0.0001	2058	0.14	0.09	0.20	<0.0001
Phospholipids in IDL (mmol/l)	2456	0.02	-0.02	0.06	0.332	2440	0.03	-0.01	0.07	0.157	2093	0.09	0.04	0.14	0.001	2058	0.11	0.06	0.17	<0.0001
Total cholesterol in IDL (mmol/l)	2456	0.04	0.00	0.08	0.065	2440	0.05	0.01	0.10	0.020	2093	0.14	0.09	0.19	<0.0001	2058	0.17	0.11	0.23	<0.0001
Cholesterol esters in IDL (mmol/l)	2456	0.05	0.01	0.10	0.018	2440	0.06	0.02	0.11	0.004	2093	0.17	0.12	0.22	<0.0001	2058	0.20	0.14	0.26	<0.0001
Free cholesterol in IDL (mmol/l)	2456	0.01	-0.03	0.05	0.640	2440	0.02	-0.02	0.06	0.356	2093	0.06	0.01	0.11	0.014	2058	0.09	0.03	0.14	0.003
Triglycerides in IDL (mmol/l)	2456	0.00	-0.04	0.04	0.920	2440	0.00	-0.04	0.04	0.974	2093	0.02	-0.03	0.07	0.373	2058	0.03	-0.02	0.08	0.257
Concentration of large LDL particles (mol/l)	2456	0.03	-0.01	0.07	0.129	2440	0.04	0.00	0.08	0.041	2093	0.10	0.06	0.15	<0.0001	2058	0.14	0.08	0.19	<0.0001
Total lipids in large LDL (mmol/l)	2456	0.03	-0.01	0.08	0.105	2440	0.05	0.00	0.09	0.031	2093	0.11	0.06	0.16	<0.0001	2058	0.14	0.09	0.20	<0.0001
Phospholipids in large LDL (mmol/l)	2456	0.04	0.00	0.08	0.039	2440	0.06	0.01	0.10	0.009	2093	0.13	0.08	0.18	<0.0001	2058	0.17	0.11	0.22	<0.0001
Total cholesterol in large LDL (mmol/l)	2456	0.04	-0.01	0.08	0.088	2440	0.05	0.01	0.09	0.023	2093	0.12	0.07	0.17	<0.0001	2058	0.15	0.10	0.21	<0.0001
Cholesterol esters in large LDL (mmol/l)	2456	0.04	0.00	0.08	0.048	2440	0.06	0.01	0.10	0.011	2093	0.13	0.08	0.18	<0.0001	2058	0.17	0.11	0.22	<0.0001
Free cholesterol in large LDL (mmol/l)	2456	0.02	-0.02	0.06	0.399	2440	0.03	-0.01	0.07	0.164	2093	0.08	0.03	0.13	0.002	2058	0.11	0.05	0.16	0.0002
Triglycerides in large LDL (mmol/l)	2456	-0.01	-0.05	0.03	0.606	2440	-0.01	-0.05	0.03	0.650	2093	-0.01	-0.06	0.04	0.657	2058	0.00	-0.05	0.05	0.912
Concentration of medium LDL particles (mol/l)	2456	0.04	0.00	0.08	0.048	2440	0.05	0.01	0.09	0.011	2093	0.12	0.07	0.17	<0.0001	2058	0.16	0.10	0.21	<0.0001

Online Table 2 Associations of body mass index (BMI) and fat mass index at age 10y and 18y with cardiometabolic traits at age 18y in ALSPAC

	**At age 10y**										**At age 18y**									
	**BMI (per 2.7 kg/m^2 higher)**					**Fat mass index (per 2.3 kg/m^2 higher)**					**BMI (per 4.0 kg/m^2 higher)**					**Fat mass index (per 3.7 kg/m^2 higher)**				
	Adj. for age, sex, ethnicity, maternal education					*Adj. for age, sex, ethnicity, maternal education*					*Adj. for age, sex, ethnicity, maternal education, smoking, alcohol, puberty timing*					*Adj. for age, sex, ethnicity, maternal education, smoking, alcohol, puberty timing*				
**Standardized outcome at age 18y**	**N**	**Beta**	**LCL**	**UCL**	**P-value**	**N**	**Beta**	**LCL**	**UCL**	**P-value**	**N**	**Beta**	**LCL**	**UCL**	**P-value**	**N**	**Beta**	**LCL**	**UCL**	**P-value**
Total lipids in medium LDL (mmol/l)	2456	0.04	0.00	0.08	0.055	2440	0.05	0.01	0.10	0.012	2093	0.12	0.07	0.17	<0.0001	2058	0.16	0.10	0.21	<0.0001
Phospholipids in medium LDL (mmol/l)	2456	0.06	0.02	0.10	0.004	2440	0.08	0.03	0.12	0.0004	2093	0.16	0.12	0.21	<0.0001	2058	0.20	0.15	0.26	<0.0001
Total cholesterol in medium LDL (mmol/l)	2456	0.04	0.00	0.08	0.058	2440	0.05	0.01	0.10	0.014	2093	0.13	0.08	0.18	<0.0001	2058	0.16	0.10	0.22	<0.0001
Cholesterol esters in medium LDL (mmol/l)	2456	0.04	0.00	0.08	0.054	2440	0.05	0.01	0.10	0.014	2093	0.13	0.08	0.18	<0.0001	2058	0.16	0.10	0.22	<0.0001
Free cholesterol in medium LDL (mmol/l)	2456	0.04	-0.01	0.08	0.092	2440	0.05	0.01	0.09	0.017	2093	0.11	0.06	0.16	<0.0001	2058	0.15	0.09	0.21	<0.0001
Triglycerides in medium LDL (mmol/l)	2456	-0.01	-0.05	0.03	0.533	2440	-0.01	-0.05	0.03	0.645	2093	-0.02	-0.06	0.03	0.457	2058	0.00	-0.06	0.05	0.850
Concentration of small LDL particles (mol/l)	2456	0.04	0.00	0.08	0.039	2440	0.06	0.02	0.10	0.006	2093	0.12	0.07	0.17	<0.0001	2058	0.16	0.10	0.21	<0.0001
Total lipids in small LDL (mmol/l)	2456	0.04	0.00	0.09	0.038	2440	0.06	0.02	0.10	0.006	2093	0.13	0.08	0.18	<0.0001	2058	0.16	0.11	0.22	<0.0001
Phospholipids in small LDL (mmol/l)	2456	0.06	0.01	0.10	0.008	2440	0.07	0.03	0.11	0.001	2093	0.15	0.10	0.20	<0.0001	2058	0.18	0.13	0.24	<0.0001
Total cholesterol in small LDL (mmol/l)	2456	0.04	0.00	0.08	0.065	2440	0.05	0.01	0.10	0.013	2093	0.12	0.07	0.17	<0.0001	2058	0.16	0.10	0.21	<0.0001
Cholesterol esters in small LDL (mmol/l)	2456	0.04	0.00	0.08	0.065	2440	0.05	0.01	0.10	0.015	2093	0.12	0.07	0.17	<0.0001	2058	0.16	0.10	0.21	<0.0001
Free cholesterol in small LDL (mmol/l)	2456	0.04	0.00	0.08	0.076	2440	0.06	0.01	0.10	0.009	2093	0.12	0.07	0.17	<0.0001	2058	0.15	0.09	0.21	<0.0001
Triglycerides in small LDL (mmol/l)	2456	0.03	0.00	0.07	0.086	2440	0.04	0.00	0.08	0.049	2093	0.09	0.04	0.14	0.0003	2058	0.11	0.06	0.17	<0.0001
Concentration of very large HDL particles (mol/l)	2456	-0.09	-0.13	-0.05	<0.0001	2440	-0.09	-0.13	-0.05	<0.0001	2093	-0.22	-0.27	-0.18	<0.0001	2058	-0.23	-0.28	-0.19	<0.0001
Total lipids in very large HDL (mmol/l)	2456	-0.09	-0.13	-0.04	<0.0001	2440	-0.08	-0.13	-0.04	0.0001	2093	-0.22	-0.26	-0.17	<0.0001	2058	-0.22	-0.27	-0.18	<0.0001
Phospholipids in very large HDL (mmol/l)	2456	-0.10	-0.14	-0.06	<0.0001	2440	-0.10	-0.14	-0.06	<0.0001	2093	-0.24	-0.29	-0.20	<0.0001	2058	-0.26	-0.30	-0.21	<0.0001
Total cholesterol in very large HDL (mmol/l)	2456	-0.07	-0.11	-0.02	0.002	2440	-0.06	-0.10	-0.02	0.006	2093	-0.18	-0.22	-0.13	<0.0001	2058	-0.17	-0.22	-0.13	<0.0001
Cholesterol esters in very large HDL (mmol/l)	2456	-0.06	-0.10	-0.02	0.006	2440	-0.05	-0.09	-0.01	0.019	2093	-0.16	-0.21	-0.12	<0.0001	2058	-0.16	-0.21	-0.11	<0.0001
Free cholesterol in very large HDL (mmol/l)	2456	-0.08	-0.13	-0.04	0.0001	2440	-0.08	-0.12	-0.04	0.0003	2093	-0.21	-0.26	-0.17	<0.0001	2058	-0.21	-0.26	-0.17	<0.0001
Triglycerides in very large HDL (mmol/l)	2456	0.00	-0.05	0.04	0.867	2440	-0.01	-0.05	0.03	0.612	2093	0.01	-0.04	0.06	0.682	2058	0.00	-0.05	0.06	0.917
Concentration of large HDL particles (mol/l)	2456	-0.10	-0.14	-0.05	<0.0001	2440	-0.10	-0.15	-0.06	<0.0001	2093	-0.26	-0.30	-0.22	<0.0001	2058	-0.28	-0.33	-0.24	<0.0001
Total lipids in large HDL (mmol/l)	2456	-0.10	-0.14	-0.06	<0.0001	2440	-0.10	-0.15	-0.06	<0.0001	2093	-0.26	-0.30	-0.22	<0.0001	2058	-0.28	-0.33	-0.23	<0.0001
Phospholipids in large HDL (mmol/l)	2456	-0.09	-0.13	-0.04	<0.0001	2440	-0.09	-0.13	-0.04	<0.0001	2093	-0.24	-0.28	-0.19	<0.0001	2058	-0.25	-0.30	-0.21	<0.0001
Total cholesterol in large HDL (mmol/l)	2456	-0.11	-0.16	-0.07	<0.0001	2440	-0.12	-0.16	-0.07	<0.0001	2093	-0.28	-0.33	-0.24	<0.0001	2058	-0.31	-0.36	-0.26	<0.0001
Cholesterol esters in large HDL (mmol/l)	2456	-0.11	-0.16	-0.07	<0.0001	2440	-0.12	-0.16	-0.07	<0.0001	2093	-0.29	-0.33	-0.24	<0.0001	2058	-0.31	-0.36	-0.26	<0.0001
Free cholesterol in large HDL (mmol/l)	2456	-0.11	-0.15	-0.06	<0.0001	2440	-0.11	-0.15	-0.06	<0.0001	2093	-0.27	-0.32	-0.23	<0.0001	2058	-0.29	-0.34	-0.25	<0.0001
Triglycerides in large HDL (mmol/l)	2456	0.00	-0.04	0.04	0.833	2440	-0.01	-0.05	0.03	0.663	2093	-0.01	-0.05	0.04	0.804	2058	-0.03	-0.08	0.02	0.268
Concentration of medium HDL particles (mol/l)	2456	0.00	-0.04	0.04	0.991	2440	0.00	-0.04	0.05	0.839	2093	-0.06	-0.10	-0.01	0.019	2058	-0.06	-0.11	-0.01	0.020
Total lipids in medium HDL (mmol/l)	2456	-0.01	-0.05	0.04	0.735	2440	0.00	-0.05	0.04	0.899	2093	-0.07	-0.12	-0.03	0.002	2058	-0.08	-0.13	-0.03	0.003
Phospholipids in medium HDL (mmol/l)	2456	-0.02	-0.06	0.03	0.479	2440	-0.01	-0.06	0.03	0.624	2093	-0.08	-0.13	-0.04	0.0004	2058	-0.09	-0.14	-0.04	0.001
Total cholesterol in medium HDL (mmol/l)	2456	-0.02	-0.06	0.03	0.481	2440	-0.01	-0.06	0.03	0.638	2093	-0.10	-0.15	-0.05	<0.0001	2058	-0.10	-0.15	-0.05	0.0001
Cholesterol esters in medium HDL (mmol/l)	2456	-0.02	-0.06	0.03	0.418	2440	-0.01	-0.06	0.03	0.563	2093	-0.11	-0.16	-0.06	<0.0001	2058	-0.11	-0.16	-0.06	<0.0001
Free cholesterol in medium HDL (mmol/l)	2456	0.00	-0.04	0.04	0.947	2440	0.00	-0.04	0.05	0.835	2093	-0.06	-0.10	-0.01	0.017	2058	-0.06	-0.11	-0.01	0.031
Triglycerides in medium HDL (mmol/l)	2456	0.11	0.07	0.16	<0.0001	2440	0.11	0.06	0.15	<0.0001	2093	0.25	0.20	0.30	<0.0001	2058	0.26	0.20	0.31	<0.0001
Concentration of small HDL particles (mol/l)	2456	0.05	0.01	0.10	0.018	2440	0.06	0.02	0.11	0.008	2093	0.06	0.01	0.11	0.015	2058	0.07	0.02	0.13	0.009
Total lipids in small HDL (mmol/l)	2456	0.02	-0.02	0.06	0.363	2440	0.03	-0.02	0.07	0.209	2093	0.00	-0.04	0.05	0.881	2058	0.01	-0.04	0.06	0.657
Phospholipids in small HDL (mmol/l)	2456	0.05	0.00	0.09	0.049	2440	0.05	0.00	0.09	0.038	2093	0.03	-0.02	0.09	0.191	2058	0.04	-0.02	0.10	0.181
Total cholesterol in small HDL (mmol/l)	2456	-0.02	-0.06	0.02	0.266	2440	-0.01	-0.05	0.03	0.642	2093	-0.06	-0.10	-0.02	0.008	2058	-0.05	-0.10	0.00	0.043
Cholesterol esters in small HDL (mmol/l)	2456	-0.02	-0.06	0.02	0.233	2440	-0.01	-0.05	0.03	0.663	2093	-0.05	-0.09	0.00	0.029	2058	-0.03	-0.08	0.01	0.160
Free cholesterol in small HDL (mmol/l)	2456	-0.01	-0.06	0.03	0.560	2440	-0.01	-0.06	0.04	0.641	2093	-0.08	-0.13	-0.03	0.002	2058	-0.09	-0.14	-0.03	0.002
Triglycerides in small HDL (mmol/l)	2456	0.08	0.03	0.12	0.001	2440	0.08	0.03	0.12	0.001	2093	0.18	0.13	0.23	<0.0001	2058	0.19	0.13	0.24	<0.0001
Phospholipids to total lipds ratio in chylomicrons and extremely large VLDL (%)	2456	0.04	0.02	0.06	<0.0001	2440	0.04	0.02	0.07	0.001	2093	0.02	0.00	0.04	0.132	2058	0.03	0.00	0.05	0.027
Total cholesterol to total lipids ratio in chylomicrons and extremely large VLDL (%)	2456	0.03	-0.01	0.07	0.186	2440	0.02	-0.02	0.07	0.258	2093	0.13	0.09	0.17	<0.0001	2058	0.13	0.08	0.18	<0.0001
Cholesterol esters to total lipids ratio in chylomicrons and extremely large VLDL (%)	2456	0.00	-0.05	0.04	0.846	2440	-0.01	-0.05	0.03	0.653	2093	0.09	0.05	0.14	<0.0001	2058	0.09	0.04	0.13	0.0004
Free cholesterol to total lipids ratio in chylomicrons and extremely large VLDL (%)	2456	0.11	0.07	0.15	<0.0001	2440	0.11	0.07	0.15	<0.0001	2093	0.15	0.10	0.19	<0.0001	2058	0.17	0.12	0.21	<0.0001
Triglycerides to total lipids ratio in chylomicrons and extremely large VLDL (%)	2456	-0.05	-0.08	-0.01	0.021	2440	-0.04	-0.08	0.00	0.082	2093	-0.12	-0.16	-0.08	<0.0001	2058	-0.12	-0.16	-0.07	<0.0001
Phospholipids to total lipds ratio in very large VLDL (%)	2456	0.11	0.07	0.15	<0.0001	2440	0.12	0.08	0.16	<0.0001	2093	0.20	0.16	0.24	<0.0001	2058	0.23	0.19	0.28	<0.0001
Total cholesterol to total lipids ratio in very large VLDL (%)	2456	-0.03	-0.07	0.01	0.134	2440	-0.04	-0.08	0.00	0.062	2093	-0.03	-0.07	0.01	0.087	2058	-0.05	-0.10	-0.01	0.024
Cholesterol esters to total lipids ratio in very large VLDL (%)	2456	-0.02	-0.08	0.04	0.565	2440	-0.03	-0.08	0.02	0.290	2093	-0.03	-0.06	0.01	0.111	2058	-0.04	-0.08	0.00	0.032
Free cholesterol to total lipids ratio in very large VLDL (%)	2456	0.03	-0.03	0.09	0.346	2440	0.03	-0.03	0.08	0.323	2093	0.01	-0.03	0.04	0.636	2058	0.01	-0.03	0.05	0.744
Triglycerides to total lipids ratio in very large VLDL (%)	2456	0.02	-0.02	0.06	0.316	2440	0.02	-0.02	0.06	0.338	2093	-0.03	-0.08	0.01	0.105	2058	-0.03	-0.07	0.02	0.239
Phospholipids to total lipds ratio in large VLDL (%)	2456	0.08	0.04	0.12	<0.0001	2440	0.09	0.05	0.13	<0.0001	2093	0.15	0.11	0.19	<0.0001	2058	0.17	0.13	0.22	<0.0001
Total cholesterol to total lipids ratio in large VLDL (%)	2456	0.06	0.02	0.10	0.003	2440	0.05	0.01	0.09	0.009	2093	0.18	0.14	0.22	<0.0001	2058	0.18	0.13	0.23	<0.0001
Cholesterol esters to total lipids ratio in large VLDL (%)	2456	0.02	-0.02	0.06	0.317	2440	0.01	-0.03	0.05	0.769	2093	0.09	0.05	0.13	<0.0001	2058	0.07	0.02	0.11	0.002
Free cholesterol to total lipids ratio in large VLDL (%)	2456	0.03	0.00	0.06	0.025	2440	0.04	0.02	0.06	0.001	2093	0.09	0.07	0.11	<0.0001	2058	0.10	0.08	0.12	<0.0001
Triglycerides to total lipids ratio in large VLDL (%)	2456	-0.08	-0.12	-0.03	0.001	2440	-0.07	-0.11	-0.03	0.001	2093	-0.18	-0.23	-0.14	<0.0001	2058	-0.19	-0.24	-0.14	<0.0001
Phospholipids to total lipids ratio in medium VLDL (%)	2456	-0.06	-0.11	-0.02	0.003	2440	-0.07	-0.11	-0.02	0.002	2093	-0.18	-0.23	-0.14	<0.0001	2058	-0.20	-0.24	-0.15	<0.0001
Total cholesterol to total lipids ratio in medium VLDL (%)	2456	0.03	-0.02	0.07	0.218	2440	0.02	-0.02	0.06	0.281	2093	0.11	0.07	0.16	<0.0001	2058	0.11	0.06	0.16	<0.0001
Cholesterol esters to total lipids ratio in medium VLDL (%)	2456	0.01	-0.03	0.05	0.563	2440	0.01	-0.03	0.05	0.711	2093	0.10	0.06	0.15	<0.0001	2058	0.09	0.04	0.14	0.0003

Online Table 2 Associations of body mass index (BMI) and fat mass index at age 10y and 18y with cardiometabolic traits at age 18y in ALSPAC

	At age 10y										At age 18y									
	**BMI (per 2.7 kg/m^2 higher)**					**Fat mass index (per 2.3 kg/m^2 higher)**					**BMI (per 4.0 kg/m^2 higher)**					**Fat mass index (per 3.7 kg/m^2 higher)**				
	Adj. for age, sex, ethnicity, maternal education					*Adj. for age, sex, ethnicity, maternal education*					*Adj. for age, sex, ethnicity, maternal education, smoking, alcohol, puberty timing*					*Adj. for age, sex, ethnicity, maternal education, smoking, alcohol, puberty timing*				
**Standardized outcome at age 18y**	**N**	**Beta**	**LCL**	**UCL**	**P-value**	**N**	**Beta**	**LCL**	**UCL**	**P-value**	**N**	**Beta**	**LCL**	**UCL**	**P-value**	**N**	**Beta**	**LCL**	**UCL**	**P-value**
Free cholesterol to total lipids ratio in medium VLDL (%)	2456	0.05	0.01	0.09	0.013	2440	0.05	0.01	0.09	0.008	2093	0.09	0.05	0.14	<0.0001	2058	0.11	0.06	0.16	<0.0001
Triglycerides to total lipids ratio in medium VLDL (%)	2456	-0.01	-0.05	0.03	0.532	2440	-0.01	-0.05	0.03	0.660	2093	-0.07	-0.11	-0.02	0.002	2058	-0.06	-0.11	-0.01	0.011
Phospholipids to total lipds ratio in small VLDL (%)	2456	-0.09	-0.14	-0.05	0.0001	2440	-0.10	-0.15	-0.05	<0.0001	2093	-0.26	-0.31	-0.21	<0.0001	2058	-0.29	-0.34	-0.24	<0.0001
Total cholesterol to total lipids ratio in small VLDL (%)	2456	-0.01	-0.06	0.03	0.567	2440	-0.01	-0.05	0.04	0.746	2093	0.02	-0.03	0.07	0.504	2058	0.03	-0.03	0.08	0.336
Cholesterol esters to total lipids ratio in small VLDL (%)	2456	0.00	-0.04	0.05	0.941	2440	0.01	-0.04	0.05	0.743	2093	0.05	0.00	0.10	0.054	2058	0.06	0.01	0.12	0.025
Free cholesterol to total lipids ratio in small VLDL (%)	2456	-0.09	-0.13	-0.05	<0.0001	2440	-0.09	-0.14	-0.05	<0.0001	2093	-0.21	-0.25	-0.17	<0.0001	2058	-0.23	-0.28	-0.19	<0.0001
Triglycerides to total lipids ratio in small VLDL (%)	2456	0.05	0.00	0.09	0.029	2440	0.04	0.00	0.09	0.043	2093	0.07	0.02	0.12	0.006	2058	0.07	0.02	0.13	0.012
Phospholipids to total lipds ratio in very small VLDL (%)	2456	-0.02	-0.06	0.02	0.332	2440	-0.02	-0.06	0.02	0.446	2093	-0.02	-0.07	0.02	0.328	2058	-0.01	-0.06	0.04	0.692
Total cholesterol to total lipids ratio in very small VLDL (%)	2456	0.00	-0.04	0.05	0.840	2440	0.01	-0.03	0.05	0.754	2093	0.00	-0.05	0.05	0.919	2058	-0.01	-0.06	0.05	0.752
Cholesterol esters to total lipids ratio in very small VLDL (%)	2456	0.03	-0.01	0.07	0.136	2440	0.03	-0.01	0.08	0.106	2093	0.06	0.01	0.11	0.014	2058	0.06	0.01	0.12	0.017
Free cholesterol to total lipids ratio in very small VLDL (%)	2456	-0.09	-0.13	-0.05	<0.0001	2440	-0.09	-0.13	-0.05	<0.0001	2093	-0.21	-0.27	-0.15	<0.0001	2058	-0.25	-0.31	-0.19	<0.0001
Triglycerides to total lipids ratio in very small VLDL (%)	2456	0.01	-0.03	0.05	0.649	2440	0.00	-0.04	0.05	0.854	2093	0.02	-0.03	0.07	0.485	2058	0.02	-0.04	0.08	0.542
Phospholipids to total lipds ratio in IDL (%)	2456	-0.09	-0.14	-0.05	<0.0001	2440	-0.10	-0.15	-0.05	<0.0001	2093	-0.25	-0.30	-0.20	<0.0001	2058	-0.27	-0.32	-0.21	<0.0001
Total cholesterol to total lipids ratio in IDL (%)	2456	0.07	0.02	0.11	0.003	2440	0.08	0.03	0.13	0.001	2093	0.18	0.12	0.23	<0.0001	2058	0.19	0.14	0.25	<0.0001
Cholesterol esters to total lipids ratio in IDL (%)	2456	0.11	0.06	0.15	<0.0001	2440	0.11	0.07	0.16	<0.0001	2093	0.26	0.21	0.31	<0.0001	2058	0.28	0.23	0.33	<0.0001
Free cholesterol to total lipids ratio in IDL (%)	2456	-0.08	-0.12	-0.03	0.001	2440	-0.08	-0.12	-0.03	0.001	2093	-0.19	-0.24	-0.13	<0.0001	2058	-0.19	-0.25	-0.13	<0.0001
Triglycerides to total lipids ratio in IDL (%)	2456	-0.04	-0.09	0.00	0.049	2440	-0.05	-0.10	-0.01	0.020	2093	-0.11	-0.16	-0.05	<0.0001	2058	-0.12	-0.18	-0.06	<0.0001
Phospholipids to total lipds ratio in large LDL (%)	2456	-0.02	-0.06	0.03	0.437	2440	-0.02	-0.07	0.02	0.257	2093	-0.06	-0.11	-0.01	0.016	2058	-0.08	-0.13	-0.03	0.003
Total cholesterol to total lipids ratio in large LDL (%)	2456	0.05	0.00	0.10	0.032	2440	0.06	0.02	0.11	0.010	2093	0.14	0.08	0.19	<0.0001	2058	0.16	0.11	0.22	<0.0001
Cholesterol esters to total lipids ratio in large LDL (%)	2456	0.08	0.03	0.12	0.001	2440	0.09	0.04	0.14	0.0002	2093	0.19	0.14	0.24	<0.0001	2058	0.22	0.17	0.28	<0.0001
Free cholesterol to total lipids ratio in large LDL (%)	2456	-0.10	-0.14	-0.05	<0.0001	2440	-0.11	-0.15	-0.06	<0.0001	2093	-0.23	-0.28	-0.18	<0.0001	2058	-0.26	-0.31	-0.20	<0.0001
Triglycerides to total lipids ratio in large LDL (%)	2456	-0.06	-0.11	-0.01	0.010	2440	-0.07	-0.12	-0.02	0.003	2093	-0.15	-0.21	-0.10	<0.0001	2058	-0.17	-0.23	-0.12	<0.0001
Phospholipids to total lipds ratio in medium LDL (%)	2456	-0.02	-0.06	0.03	0.439	2440	-0.02	-0.07	0.02	0.328	2093	-0.06	-0.10	-0.01	0.025	2058	-0.07	-0.13	-0.02	0.009
Total cholesterol to total lipids ratio in medium LDL (%)	2456	0.04	0.00	0.09	0.060	2440	0.05	0.00	0.10	0.035	2093	0.11	0.06	0.16	<0.0001	2058	0.13	0.07	0.18	<0.0001
Cholesterol esters to total lipids ratio in medium LDL (%)	2456	0.05	0.01	0.10	0.023	2440	0.06	0.01	0.10	0.012	2093	0.13	0.08	0.18	<0.0001	2058	0.15	0.10	0.21	<0.0001
Free cholesterol to total lipids ratio in medium LDL (%)	2456	-0.06	-0.10	-0.01	0.009	2440	-0.06	-0.10	-0.02	0.004	2093	-0.14	-0.18	-0.09	<0.0001	2058	-0.16	-0.21	-0.11	<0.0001
Triglycerides to total lipids ratio in medium LDL (%)	2456	-0.06	-0.10	-0.01	0.008	2440	-0.06	-0.11	-0.02	0.004	2093	-0.13	-0.18	-0.09	<0.0001	2058	-0.14	-0.19	-0.09	<0.0001
Phospholipids to total lipds ratio in small LDL (%)	2456	-0.03	-0.08	0.01	0.127	2440	-0.04	-0.09	0.00	0.062	2093	-0.10	-0.14	-0.05	<0.0001	2058	-0.12	-0.17	-0.07	<0.0001
Total cholesterol to total lipids ratio in small LDL (%)	2456	0.03	-0.02	0.07	0.234	2440	0.04	-0.01	0.08	0.116	2093	0.08	0.03	0.13	0.001	2058	0.10	0.05	0.16	0.0004
Cholesterol esters to total lipids ratio in small LDL (%)	2456	0.04	0.00	0.08	0.067	2440	0.05	0.01	0.10	0.029	2093	0.11	0.06	0.16	<0.0001	2058	0.13	0.08	0.19	<0.0001
Free cholesterol to total lipids ratio in small LDL (%)	2456	-0.06	-0.10	-0.01	0.008	2440	-0.06	-0.10	-0.02	0.004	2093	-0.13	-0.18	-0.09	<0.0001	2058	-0.16	-0.21	-0.11	<0.0001
Triglycerides to total lipids ratio in small LDL (%)	2456	0.01	-0.04	0.05	0.674	2440	0.00	-0.04	0.05	0.847	2093	0.02	-0.03	0.07	0.468	2058	0.02	-0.04	0.08	0.438
Phospholipids to total lipds ratio in very large HDL (%)	2456	-0.12	-0.16	-0.08	<0.0001	2440	-0.13	-0.17	-0.08	<0.0001	2093	-0.26	-0.31	-0.21	<0.0001	2058	-0.28	-0.33	-0.23	<0.0001
Total cholesterol to total lipids ratio in very large HDL (%)	2456	0.11	0.06	0.15	<0.0001	2440	0.11	0.07	0.16	<0.0001	2093	0.23	0.18	0.27	<0.0001	2058	0.24	0.19	0.29	<0.0001
Cholesterol esters to total lipids ratio in very large HDL (%)	2456	0.10	0.06	0.15	<0.0001	2440	0.11	0.07	0.15	<0.0001	2093	0.22	0.18	0.27	<0.0001	2058	0.24	0.19	0.29	<0.0001
Free cholesterol to total lipids ratio in very large HDL (%)	2456	-0.02	-0.06	0.02	0.330	2440	-0.01	-0.05	0.03	0.574	2093	-0.06	-0.11	-0.01	0.032	2058	-0.04	-0.10	0.02	0.169
Triglycerides to total lipids ratio in very large HDL (%)	2456	0.10	0.04	0.15	0.0004	2440	0.09	0.03	0.14	0.001	2093	0.24	0.17	0.31	<0.0001	2058	0.24	0.17	0.31	<0.0001
Phospholipids to total lipds ratio in large HDL (%)	2456	0.17	0.12	0.22	<0.0001	2440	0.18	0.13	0.22	<0.0001	2093	0.34	0.28	0.39	<0.0001	2058	0.38	0.32	0.44	<0.0001
Total cholesterol to total lipids ratio in large HDL (%)	2456	-0.17	-0.22	-0.12	<0.0001	2440	-0.17	-0.22	-0.12	<0.0001	2093	-0.36	-0.42	-0.30	<0.0001	2058	-0.39	-0.45	-0.33	<0.0001
Cholesterol esters to total lipids ratio in large HDL (%)	2456	-0.17	-0.23	-0.12	<0.0001	2440	-0.18	-0.23	-0.13	<0.0001	2093	-0.36	-0.42	-0.31	<0.0001	2058	-0.40	-0.46	-0.33	<0.0001
Free cholesterol to total lipids ratio in large HDL (%)	2456	-0.13	-0.17	-0.08	<0.0001	2440	-0.12	-0.17	-0.08	<0.0001	2093	-0.26	-0.32	-0.21	<0.0001	2058	-0.27	-0.33	-0.21	<0.0001
Triglycerides to total lipids ratio in large HDL (%)	2456	0.14	0.09	0.20	<0.0001	2440	0.13	0.08	0.19	<0.0001	2093	0.32	0.26	0.38	<0.0001	2058	0.33	0.26	0.39	<0.0001
Phospholipids to total lipds ratio in medium HDL (%)	2456	-0.04	-0.08	0.00	0.045	2440	-0.04	-0.08	0.00	0.082	2093	-0.08	-0.13	-0.04	0.001	2058	-0.09	-0.14	-0.04	0.001
Total cholesterol to total lipids ratio in medium HDL (%)	2456	-0.03	-0.07	0.01	0.128	2440	-0.03	-0.08	0.01	0.125	2093	-0.09	-0.13	-0.04	0.001	2058	-0.09	-0.14	-0.04	0.001
Cholesterol esters to total lipids ratio in medium HDL (%)	2456	-0.04	-0.08	0.00	0.075	2440	-0.04	-0.08	0.00	0.070	2093	-0.10	-0.15	-0.05	<0.0001	2058	-0.11	-0.16	-0.05	<0.0001
Free cholesterol to total lipids ratio in medium HDL (%)	2456	0.02	-0.02	0.06	0.283	2440	0.02	-0.01	0.06	0.222	2093	0.05	0.01	0.10	0.030	2058	0.06	0.01	0.12	0.024
Triglycerides to total lipids ratio in medium HDL (%)	2456	0.13	0.09	0.18	<0.0001	2440	0.13	0.08	0.18	<0.0001	2093	0.32	0.26	0.38	<0.0001	2058	0.33	0.27	0.40	<0.0001
Phospholipids to total lipds ratio in small HDL (%)	2456	0.05	0.01	0.09	0.014	2440	0.04	0.00	0.07	0.073	2093	0.06	0.01	0.10	0.010	2058	0.05	0.00	0.09	0.056
Total cholesterol to total lipids ratio in small HDL (%)	2456	-0.07	-0.11	-0.03	0.000	2440	-0.06	-0.10	-0.02	0.007	2093	-0.11	-0.16	-0.07	<0.0001	2058	-0.10	-0.15	-0.05	<0.0001
Cholesterol esters to total lipids ratio in small HDL (%)	2456	-0.05	-0.09	-0.01	0.010	2440	-0.04	-0.08	0.00	0.080	2093	-0.07	-0.11	-0.02	0.003	2058	-0.05	-0.10	0.00	0.036
Free cholesterol to total lipids ratio in small HDL (%)	2456	-0.10	-0.14	-0.05	<0.0001	2440	-0.11	-0.15	-0.06	<0.0001	2093	-0.24	-0.30	-0.19	<0.0001	2058	-0.29	-0.34	-0.23	<0.0001
Triglycerides to total lipids ratio in small HDL (%)	2456	0.08	0.04	0.13	0.001	2440	0.08	0.03	0.12	0.002	2093	0.20	0.14	0.26	<0.0001	2058	0.21	0.14	0.27	<0.0001
Mean diameter for VLDL particles (nm)	2456	0.14	0.09	0.18	<0.0001	2440	0.14	0.09	0.18	<0.0001	2093	0.30	0.25	0.35	<0.0001	2058	0.32	0.27	0.38	<0.0001
Mean diameter for LDL particles (nm)	2456	-0.07	-0.11	-0.03	0.001	2440	-0.09	-0.13	-0.05	<0.0001	2093	-0.12	-0.16	-0.07	<0.0001	2058	-0.16	-0.21	-0.11	<0.0001
Mean diameter for HDL particles (nm)	2456	-0.12	-0.16	-0.07	<0.0001	2440	-0.12	-0.16	-0.07	<0.0001	2093	-0.27	-0.31	-0.22	<0.0001	2058	-0.28	-0.33	-0.24	<0.0001
Serum total cholesterol (mmol/l)	2456	0.03	-0.01	0.07	0.109	2440	0.05	0.01	0.09	0.028	2093	0.10	0.05	0.15	<0.0001	2058	0.13	0.07	0.18	<0.0001
Total cholesterol in VLDL (mmol/l)	2456	0.13	0.08	0.18	<0.0001	2440	0.14	0.09	0.19	<0.0001	2093	0.33	0.27	0.38	<0.0001	2058	0.36	0.30	0.42	<0.0001
Remnant cholesterol (non-HDL, non-LDL -cholesterol) (mmol/l)	2456	0.10	0.05	0.14	<0.0001	2440	0.11	0.06	0.16	<0.0001	2093	0.27	0.21	0.32	<0.0001	2058	0.30	0.24	0.36	<0.0001
Total cholesterol in LDL (mmol/l)	2456	0.04	0.00	0.08	0.073	2440	0.05	0.01	0.09	0.018	2093	0.12	0.07	0.17	<0.0001	2058	0.16	0.10	0.21	<0.0001

Online Table 2 Associations of body mass index (BMI) and fat mass index at age 10y and 18y with cardiometabolic traits at age 18y in ALSPAC

	At age 10y										At age 18y									
	**BMI (per 2.7 kg/m^2 higher)**					**Fat mass index (per 2.3 kg/m^2 higher)**					**BMI (per 4.0 kg/m^2 higher)**					**Fat mass index (per 3.7 kg/m^2 higher)**				
	Adj. for age, sex, ethnicity, maternal education					*Adj. for age, sex, ethnicity, maternal education*					*Adj. for age, sex, ethnicity, maternal education, smoking, alcohol, puberty timing*					*Adj. for age, sex, ethnicity, maternal education, smoking, alcohol, puberty timing*				
**Standardized outcome at age 18y**	**N**	**Beta**	**LCL**	**UCL**	**P-value**	**N**	**Beta**	**LCL**	**UCL**	**P-value**	**N**	**Beta**	**LCL**	**UCL**	**P-value**	**N**	**Beta**	**LCL**	**UCL**	**P-value**
Total cholesterol in HDL (mmol/l)	2456	-0.08	-0.12	-0.03	0.0005	2440	-0.07	-0.12	-0.03	0.001	2093	-0.22	-0.26	-0.17	<0.0001	2058	-0.22	-0.27	-0.17	<0.0001
Total cholesterol in HDL2 (mmol/l)	2456	-0.09	-0.14	-0.05	<0.0001	2440	-0.09	-0.13	-0.04	0.0002	2093	-0.25	-0.29	-0.20	<0.0001	2058	-0.26	-0.31	-0.21	<0.0001
Total cholesterol in HDL3 (mmol/l)	2456	-0.05	-0.09	-0.01	0.017	2440	-0.05	-0.09	0.00	0.038	2093	-0.15	-0.19	-0.10	<0.0001	2058	-0.15	-0.20	-0.10	<0.0001
Esterified cholesterol (mmol/l)	2446	0.03	-0.01	0.07	0.130	2430	0.04	0.00	0.09	0.035	2086	0.10	0.05	0.15	<0.0001	2051	0.13	0.07	0.18	<0.0001
Free cholesterol (mmol/l)	2444	0.03	0.00	0.07	0.085	2428	0.05	0.01	0.09	0.026	2084	0.10	0.05	0.15	<0.0001	2049	0.13	0.07	0.18	<0.0001
Serum total triglycerides (mmol/l)	2456	0.12	0.08	0.17	<0.0001	2440	0.13	0.08	0.17	<0.0001	2093	0.29	0.23	0.34	<0.0001	2058	0.31	0.25	0.38	<0.0001
Triglycerides in VLDL (mmol/l)	2456	0.14	0.09	0.19	<0.0001	2440	0.15	0.10	0.19	<0.0001	2093	0.32	0.27	0.38	<0.0001	2058	0.35	0.29	0.42	<0.0001
Triglycerides in LDL (mmol/l)	2456	0.00	-0.04	0.04	0.887	2440	0.00	-0.04	0.04	0.986	2093	0.01	-0.04	0.05	0.827	2058	0.02	-0.03	0.07	0.510
Triglycerides in HDL (mmol/l)	2456	0.08	0.03	0.12	0.001	2440	0.07	0.02	0.11	0.002	2093	0.17	0.12	0.22	<0.0001	2058	0.17	0.11	0.23	<0.0001
Diacylglycerol (mmol/l)	2386	0.11	0.06	0.15	<0.0001	2373	0.11	0.06	0.15	<0.0001	2031	0.22	0.17	0.28	<0.0001	1997	0.23	0.17	0.29	<0.0001
Ratio of diacylglycerol to triglycerides	2387	0.07	0.03	0.12	0.001	2374	0.08	0.03	0.12	0.001	2032	0.10	0.05	0.15	<0.0001	1998	0.10	0.05	0.16	0.0001
Total phosphoglycerides (mmol/l)	2444	0.01	-0.03	0.05	0.632	2428	0.01	-0.03	0.05	0.598	2084	-0.01	-0.06	0.03	0.602	2049	-0.01	-0.06	0.04	0.630
Ratio of triglycerides to phosphoglycerides	2444	0.14	0.09	0.19	<0.0001	2428	0.13	0.09	0.18	<0.0001	2084	0.31	0.25	0.36	<0.0001	2049	0.33	0.26	0.39	<0.0001
Phosphatidylcholine and other cholines (mmol/l)	2425	-0.01	-0.05	0.03	0.565	2409	-0.01	-0.05	0.03	0.505	2068	-0.02	-0.07	0.02	0.307	2034	-0.03	-0.09	0.02	0.207
Total cholines (mmol/l)	2446	0.00	-0.04	0.04	0.877	2430	0.00	-0.04	0.04	0.963	2086	-0.01	-0.06	0.03	0.634	2051	-0.01	-0.06	0.04	0.626
Apolipoprotein A-I (g/l)	2456	-0.04	-0.08	0.00	0.085	2440	-0.03	-0.07	0.01	0.190	2093	-0.11	-0.15	-0.07	<0.0001	2058	-0.11	-0.15	-0.06	<0.0001
Apolipoprotein B (g/l)	2456	0.10	0.06	0.15	<0.0001	2440	0.11	0.07	0.16	<0.0001	2093	0.27	0.22	0.33	<0.0001	2058	0.31	0.25	0.37	<0.0001
Ratio of apolipoprotein B to apolipoprotein A-I	2456	0.13	0.08	0.18	<0.0001	2440	0.13	0.08	0.18	<0.0001	2093	0.33	0.27	0.38	<0.0001	2058	0.36	0.30	0.42	<0.0001
Total fatty acids (mmol/l)	2446	0.07	0.03	0.11	0.001	2430	0.08	0.04	0.12	0.0003	2086	0.16	0.11	0.21	<0.0001	2051	0.19	0.13	0.24	<0.0001
Estimated description of fatty acid chain length, not actual carbon number	2446	0.03	-0.01	0.08	0.152	2430	0.03	-0.02	0.07	0.239	2087	0.00	-0.05	0.05	0.980	2052	-0.01	-0.07	0.05	0.830
Estimated degree of unsaturation	2446	-0.01	-0.05	0.03	0.713	2430	-0.01	-0.05	0.03	0.613	2086	-0.07	-0.12	-0.02	0.004	2051	-0.08	-0.14	-0.03	0.003
22:6, docosahexaenoic acid (mmol/l)	2446	0.02	-0.02	0.06	0.235	2430	0.02	-0.02	0.06	0.273	2086	0.04	-0.01	0.08	0.090	2051	0.05	0.00	0.09	0.068
18:2, linoleic acid (mmol/l)	2445	0.02	-0.02	0.06	0.376	2429	0.03	-0.01	0.07	0.175	2086	0.07	0.02	0.12	0.004	2051	0.08	0.02	0.14	0.005
Conjugated linoleic acid (mmol/l)	2445	0.05	0.02	0.09	0.006	2429	0.05	0.01	0.09	0.018	2085	0.10	0.05	0.15	<0.0001	2050	0.11	0.06	0.16	<0.0001
Omega-3 fatty acids (mmol/l)	2446	0.06	0.02	0.10	0.003	2430	0.07	0.02	0.11	0.003	2086	0.11	0.07	0.16	<0.0001	2051	0.13	0.08	0.19	<0.0001
Omega-6 fatty acids (mmol/l)	2446	0.03	-0.01	0.07	0.109	2430	0.04	0.00	0.08	0.041	2086	0.09	0.05	0.14	0.0001	2051	0.11	0.06	0.17	<0.0001
Polyunsaturated fatty acids (mmol/l)	2445	0.04	0.00	0.08	0.061	2429	0.05	0.01	0.09	0.024	2086	0.10	0.05	0.15	<0.0001	2051	0.12	0.06	0.17	<0.0001
Monounsaturated fatty acids; 16:1, 18:1 (mmol/l)	2446	0.09	0.04	0.13	<0.0001	2430	0.10	0.05	0.14	<0.0001	2086	0.20	0.14	0.25	<0.0001	2051	0.23	0.18	0.29	<0.0001
Saturated fatty acids (mmol/l)	2445	0.07	0.02	0.11	0.003	2429	0.07	0.03	0.11	0.002	2085	0.15	0.10	0.20	<0.0001	2050	0.16	0.10	0.22	<0.0001
Ratio of 22:6 docosahexaenoic acid to total fatty acids (%)	2447	0.00	-0.04	0.04	0.996	2431	-0.01	-0.05	0.04	0.765	2087	-0.04	-0.09	0.01	0.082	2052	-0.04	-0.09	0.00	0.069
Ratio of 18:2 linoleic acid to total fatty acids (%)	2446	-0.10	-0.14	-0.05	<0.0001	2430	-0.10	-0.14	-0.05	<0.0001	2087	-0.17	-0.22	-0.12	<0.0001	2052	-0.20	-0.26	-0.15	<0.0001
Ratio of conjugated linoleic acid to total fatty acids (%)	2446	0.04	0.00	0.08	0.036	2430	0.03	-0.01	0.07	0.127	2086	0.08	0.03	0.12	0.001	2051	0.08	0.03	0.13	0.001
Ratio of omega-3 fatty acids to total fatty acids (%)	2447	0.02	-0.02	0.07	0.256	2431	0.02	-0.02	0.06	0.370	2087	0.00	-0.04	0.05	0.915	2052	0.00	-0.05	0.05	0.901
Ratio of omega-6 fatty acids to total fatty acids (%)	2447	-0.09	-0.14	-0.04	0.0001	2431	-0.09	-0.14	-0.04	0.0002	2087	-0.16	-0.21	-0.11	<0.0001	2052	-0.18	-0.24	-0.13	<0.0001
Ratio of polyunsaturated fatty acids to total fatty acids (%)	2446	-0.08	-0.13	-0.03	0.001	2430	-0.08	-0.13	-0.03	0.001	2087	-0.15	-0.20	-0.10	<0.0001	2052	-0.17	-0.23	-0.12	<0.0001
Ratio of monounsaturated fatty acids to total fatty acids (%)	2447	0.08	0.04	0.12	0.0004	2431	0.09	0.04	0.13	0.0002	2087	0.16	0.11	0.21	<0.0001	2052	0.20	0.15	0.26	<0.0001
Ratio of saturated fatty acids to total fatty acids (%)	2446	-0.01	-0.06	0.03	0.604	2430	-0.02	-0.07	0.03	0.349	2086	-0.03	-0.08	0.02	0.219	2051	-0.06	-0.12	-0.01	0.021
Insulin (mu/l)	2494	0.19	0.09	0.28	<0.0001	2478	0.19	0.10	0.28	<0.0001	2133	0.32	0.21	0.42	<0.0001	2097	0.35	0.24	0.45	<0.0001
Glucose (mmol/l)	2455	0.05	0.01	0.09	0.007	2439	0.04	0.00	0.08	0.029	2092	0.11	0.08	0.15	<0.0001	2057	0.12	0.09	0.16	<0.0001
Lactate (mmol/l)	2455	0.03	-0.01	0.07	0.144	2439	0.05	0.00	0.09	0.031	2092	0.01	-0.04	0.05	0.706	2057	0.03	-0.02	0.08	0.210
Pyruvate (mmol/l)	2455	0.11	0.06	0.15	<0.0001	2439	0.11	0.06	0.16	<0.0001	2092	0.13	0.09	0.18	<0.0001	2057	0.16	0.11	0.21	<0.0001
Citrate (mmol/l)	2455	-0.15	-0.19	-0.10	<0.0001	2439	-0.14	-0.19	-0.10	<0.0001	2092	-0.16	-0.21	-0.11	<0.0001	2057	-0.16	-0.21	-0.11	<0.0001
Alanine (mmol/l)	2455	0.04	-0.01	0.08	0.087	2439	0.03	-0.02	0.07	0.207	2092	0.12	0.07	0.17	<0.0001	2057	0.12	0.07	0.17	<0.0001
Glutamine (mmol/l)	2455	-0.05	-0.09	-0.01	0.024	2439	-0.04	-0.08	0.00	0.046	2092	-0.03	-0.08	0.02	0.193	2057	-0.03	-0.08	0.02	0.302
Histidine (mmol/l)	2455	0.02	-0.02	0.06	0.275	2439	0.02	-0.02	0.06	0.362	2092	0.05	0.00	0.10	0.041	2057	0.04	-0.01	0.10	0.103
Isoleucine (mmol/l)	2455	0.13	0.08	0.17	<0.0001	2439	0.12	0.07	0.16	<0.0001	2092	0.24	0.19	0.29	<0.0001	2057	0.23	0.18	0.28	<0.0001
Leucine (mmol/l)	2455	0.09	0.05	0.13	<0.0001	2439	0.07	0.03	0.11	0.001	2092	0.18	0.13	0.22	<0.0001	2057	0.14	0.09	0.19	<0.0001
Valine (mmol/l)	2455	0.15	0.11	0.19	<0.0001	2439	0.13	0.09	0.18	<0.0001	2092	0.24	0.20	0.29	<0.0001	2057	0.23	0.18	0.28	<0.0001
Phenylalanine (mmol/l)	2454	0.11	0.07	0.16	<0.0001	2438	0.10	0.05	0.15	<0.0001	2091	0.20	0.15	0.24	<0.0001	2057	0.19	0.14	0.24	<0.0001
Tyrosine (mmol/l)	2455	0.13	0.09	0.18	<0.0001	2439	0.13	0.08	0.17	<0.0001	2092	0.28	0.23	0.33	<0.0001	2057	0.29	0.23	0.35	<0.0001
Acetate (mmol/l)	2454	-0.05	-0.08	-0.02	0.001	2438	-0.05	-0.07	-0.02	0.001	2092	-0.09	-0.13	-0.04	<0.0001	2057	-0.08	-0.13	-0.04	<0.0001
Acetoacetate (mmol/l)	2455	-0.02	-0.06	0.02	0.293	2439	0.00	-0.04	0.05	0.871	2092	-0.10	-0.15	-0.05	0.0002	2057	-0.07	-0.11	-0.02	0.003
3-hydroxybutyrate (mmol/l)	2452	-0.03	-0.07	0.01	0.103	2436	0.00	-0.05	0.04	0.888	2089	-0.11	-0.16	-0.06	<0.0001	2054	-0.08	-0.13	-0.03	0.001
Creatinine (mmol/l)	2455	0.05	0.02	0.09	0.006	2439	0.01	-0.03	0.04	0.723	2092	0.08	0.04	0.12	0.0001	2057	-0.01	-0.05	0.04	0.803
Albumin (signal area)	2456	-0.05	-0.09	-0.01	0.020	2440	-0.05	-0.10	-0.01	0.009	2093	-0.09	-0.14	-0.04	0.0002	2058	-0.09	-0.14	-0.04	0.0004
Glycoprotein acetyls, mainly a1-acid glycoprotein (mmol/l)	2455	0.18	0.14	0.23	<0.0001	2439	0.19	0.15	0.24	<0.0001	2092	0.31	0.26	0.36	<0.0001	2057	0.36	0.31	0.41	<0.0001
C-reactive protein (mg/l)	2536	0.08	0.04	0.11	<0.0001	2519	0.08	0.04	0.12	0.0001	2160	0.09	0.04	0.15	0.0002	2124	0.12	0.06	0.17	0.0001

Online Table 2 Associations of body mass index (BMI) and fat mass index at age 10y and 18y with cardiometabolic traits at age 18y in ALSPAC

	At age 10y										At age 18y									
	**BMI (per 2.7 kg/m^2 higher)**					**Fat mass index (per 2.3 kg/m^2 higher)**					**BMI (per 4.0 kg/m^2 higher)**					**Fat mass index (per 3.7 kg/m^2 higher)**				
	Adj. for age, sex, ethnicity, maternal education					*Adj. for age, sex, ethnicity, maternal education*					*Adj. for age, sex, ethnicity, maternal education, smoking, alcohol, puberty timing*					*Adj. for age, sex, ethnicity, maternal education, smoking, alcohol, puberty timing*				
**Standardized outcome at age 18y**	**N**	**Beta**	**LCL**	**UCL**	**P-value**	**N**	**Beta**	**LCL**	**UCL**	**P-value**	**N**	**Beta**	**LCL**	**UCL**	**P-value**	**N**	**Beta**	**LCL**	**UCL**	**P-value**

Complete case sample

	At age 10y										At age 18y									
	**BMI (per 2.6 kg/m^2 higher)**					**Fat mass index (per 2.2 kg/m^2 higher)**					**BMI (per 3.8 kg/m^2 higher)**					**Fat mass index (per 3.6 kg/m^2 higher)**				
	Adj. for age, sex, ethnicity, maternal education					*Adj. for age, sex, ethnicity, maternal education*					*Adj. for age, sex, ethnicity, maternal education, smoking, alcohol, puberty timing*					*Adj. for age, sex, ethnicity, maternal education, smoking, alcohol, puberty timing*				
**Standardized outcome at age 18y**	**N**	**Beta**	**LCL**	**UCL**	**P-value**	**N**	**Beta**	**LCL**	**UCL**	**P-value**	**N**	**Beta**	**LCL**	**UCL**	**P-value**	**N**	**Beta**	**LCL**	**UCL**	**P-value**
Systolic blood pressure (mmHg)	1722	0.21	0.16	0.26	<0.0001	1722	0.18	0.13	0.23	<0.0001	1722	0.28	0.23	0.33	<0.0001	1722	0.24	0.19	0.29	<0.0001
Diastolic blood pressure (mmHg)	1722	0.22	0.17	0.27	<0.0001	1722	0.23	0.18	0.29	<0.0001	1722	0.28	0.23	0.34	<0.0001	1722	0.34	0.28	0.40	<0.0001
Concentration of chylomicrons and extremely large VLDL particles (mol/l)	1722	0.16	0.11	0.22	<0.0001	1722	0.17	0.11	0.23	<0.0001	1722	0.36	0.29	0.42	<0.0001	1722	0.40	0.33	0.47	<0.0001
Total lipids in chylomicrons and extremely large VLDL (mmol/l)	1722	0.16	0.10	0.22	<0.0001	1722	0.17	0.11	0.23	<0.0001	1722	0.35	0.28	0.42	<0.0001	1722	0.39	0.32	0.46	<0.0001
Phospholipids in chylomicrons and extremely large VLDL (mmol/l)	1722	0.16	0.10	0.22	<0.0001	1722	0.17	0.11	0.22	<0.0001	1722	0.34	0.28	0.41	<0.0001	1722	0.38	0.31	0.45	<0.0001
Total cholesterol in chylomicrons and extremely large VLDL (mmol/l)	1722	0.15	0.10	0.21	<0.0001	1722	0.16	0.10	0.22	<0.0001	1722	0.35	0.28	0.42	<0.0001	1722	0.38	0.31	0.46	<0.0001
Cholesterol esters in chylomicrons and extremely large VLDL (mmol/l)	1722	0.15	0.09	0.20	<0.0001	1722	0.15	0.09	0.21	<0.0001	1722	0.34	0.28	0.41	<0.0001	1722	0.38	0.30	0.45	<0.0001
Free cholesterol in chylomicrons and extremely large VLDL (mmol/l)	1722	0.16	0.10	0.22	<0.0001	1722	0.16	0.11	0.22	<0.0001	1722	0.34	0.28	0.41	<0.0001	1722	0.38	0.31	0.45	<0.0001
Triglycerides in chylomicrons and extremely large VLDL (mmol/l)	1722	0.16	0.11	0.22	<0.0001	1722	0.17	0.11	0.23	<0.0001	1722	0.35	0.28	0.41	<0.0001	1722	0.39	0.32	0.46	<0.0001
Concentration of very large VLDL particles (mol/l)	1722	0.16	0.10	0.22	<0.0001	1722	0.16	0.11	0.22	<0.0001	1722	0.35	0.28	0.41	<0.0001	1722	0.38	0.31	0.45	<0.0001
Total lipids in very large VLDL (mmol/l)	1722	0.16	0.10	0.22	<0.0001	1722	0.16	0.11	0.22	<0.0001	1722	0.34	0.28	0.41	<0.0001	1722	0.38	0.31	0.45	<0.0001
Phospholipids in very large VLDL (mmol/l)	1722	0.15	0.10	0.21	<0.0001	1722	0.16	0.10	0.22	<0.0001	1722	0.34	0.27	0.41	<0.0001	1722	0.38	0.31	0.45	<0.0001
Total cholesterol in very large VLDL (mmol/l)	1722	0.16	0.10	0.22	<0.0001	1722	0.17	0.11	0.22	<0.0001	1722	0.36	0.29	0.43	<0.0001	1722	0.40	0.32	0.47	<0.0001
Cholesterol esters in very large VLDL (mmol/l)	1722	0.16	0.10	0.22	<0.0001	1722	0.17	0.11	0.23	<0.0001	1722	0.37	0.30	0.43	<0.0001	1722	0.40	0.33	0.47	<0.0001
Free cholesterol in very large VLDL (mmol/l)	1722	0.16	0.10	0.22	<0.0001	1722	0.16	0.11	0.22	<0.0001	1722	0.35	0.28	0.42	<0.0001	1722	0.39	0.32	0.46	<0.0001
Triglycerides in very large VLDL (mmol/l)	1722	0.16	0.10	0.21	<0.0001	1722	0.16	0.10	0.22	<0.0001	1722	0.34	0.27	0.40	<0.0001	1722	0.37	0.30	0.44	<0.0001
Concentration of large VLDL particles (mol/l)	1722	0.15	0.10	0.21	<0.0001	1722	0.16	0.10	0.22	<0.0001	1722	0.33	0.27	0.40	<0.0001	1722	0.36	0.29	0.43	<0.0001
Total lipids in large VLDL (mmol/l)	1722	0.15	0.10	0.21	<0.0001	1722	0.16	0.10	0.22	<0.0001	1722	0.33	0.27	0.40	<0.0001	1722	0.37	0.30	0.44	<0.0001
Phospholipids in large VLDL (mmol/l)	1722	0.15	0.10	0.21	<0.0001	1722	0.16	0.10	0.22	<0.0001	1722	0.33	0.27	0.40	<0.0001	1722	0.36	0.29	0.44	<0.0001
Total cholesterol in large VLDL (mmol/l)	1722	0.15	0.10	0.21	<0.0001	1722	0.16	0.10	0.22	<0.0001	1722	0.34	0.28	0.41	<0.0001	1722	0.38	0.31	0.45	<0.0001
Cholesterol esters in large VLDL (mmol/l)	1722	0.15	0.10	0.21	<0.0001	1722	0.16	0.10	0.22	<0.0001	1722	0.35	0.29	0.42	<0.0001	1722	0.38	0.31	0.45	<0.0001
Free cholesterol in large VLDL (mmol/l)	1722	0.15	0.09	0.21	<0.0001	1722	0.16	0.10	0.22	<0.0001	1722	0.33	0.27	0.40	<0.0001	1722	0.37	0.30	0.44	<0.0001
Triglycerides in large VLDL (mmol/l)	1722	0.15	0.10	0.21	<0.0001	1722	0.16	0.10	0.22	<0.0001	1722	0.33	0.26	0.39	<0.0001	1722	0.36	0.29	0.43	<0.0001
Concentration of medium VLDL particles (mol/l)	1722	0.16	0.10	0.21	<0.0001	1722	0.16	0.10	0.22	<0.0001	1722	0.34	0.28	0.41	<0.0001	1722	0.37	0.30	0.44	<0.0001
Total lipids in medium VLDL (mmol/l)	1722	0.16	0.10	0.21	<0.0001	1722	0.16	0.10	0.22	<0.0001	1722	0.35	0.28	0.41	<0.0001	1722	0.38	0.31	0.45	<0.0001
Phospholipids in medium VLDL (mmol/l)	1722	0.15	0.09	0.21	<0.0001	1722	0.16	0.10	0.21	<0.0001	1722	0.34	0.27	0.40	<0.0001	1722	0.37	0.30	0.44	<0.0001
Total cholesterol in medium VLDL (mmol/l)	1722	0.14	0.09	0.20	<0.0001	1722	0.15	0.09	0.21	<0.0001	1722	0.34	0.28	0.40	<0.0001	1722	0.37	0.30	0.44	<0.0001
Cholesterol esters in medium VLDL (mmol/l)	1722	0.14	0.08	0.19	<0.0001	1722	0.14	0.08	0.20	<0.0001	1722	0.34	0.28	0.40	<0.0001	1722	0.36	0.29	0.43	<0.0001
Free cholesterol in medium VLDL (mmol/l)	1722	0.15	0.09	0.20	<0.0001	1722	0.15	0.09	0.21	<0.0001	1722	0.33	0.26	0.39	<0.0001	1722	0.36	0.29	0.43	<0.0001
Triglycerides in medium VLDL (mmol/l)	1722	0.16	0.10	0.22	<0.0001	1722	0.16	0.11	0.22	<0.0001	1722	0.34	0.28	0.40	<0.0001	1722	0.37	0.30	0.44	<0.0001
Concentration of small VLDL particles (mol/l)	1722	0.13	0.08	0.19	<0.0001	1722	0.14	0.08	0.20	<0.0001	1722	0.31	0.25	0.37	<0.0001	1722	0.34	0.28	0.41	<0.0001
Total lipids in small VLDL (mmol/l)	1722	0.13	0.07	0.19	<0.0001	1722	0.14	0.08	0.20	<0.0001	1722	0.32	0.26	0.38	<0.0001	1722	0.35	0.29	0.42	<0.0001
Phospholipids in small VLDL (mmol/l)	1722	0.12	0.06	0.17	<0.0001	1722	0.13	0.07	0.18	<0.0001	1722	0.29	0.23	0.34	<0.0001	1722	0.32	0.25	0.38	<0.0001
Total cholesterol in small VLDL (mmol/l)	1722	0.12	0.06	0.17	<0.0001	1722	0.12	0.06	0.18	<0.0001	1722	0.31	0.25	0.36	<0.0001	1722	0.34	0.27	0.41	<0.0001
Cholesterol esters in small VLDL (mmol/l)	1722	0.11	0.05	0.17	0.0001	1722	0.12	0.06	0.18	0.0001	1722	0.30	0.24	0.36	<0.0001	1722	0.34	0.27	0.40	<0.0001
Free cholesterol in small VLDL (mmol/l)	1722	0.11	0.06	0.17	<0.0001	1722	0.12	0.07	0.18	<0.0001	1722	0.28	0.22	0.34	<0.0001	1722	0.31	0.25	0.38	<0.0001
Triglycerides in small VLDL (mmol/l)	1722	0.13	0.08	0.19	<0.0001	1722	0.14	0.08	0.19	<0.0001	1722	0.30	0.24	0.36	<0.0001	1722	0.33	0.26	0.40	<0.0001
Concentration of very small VLDL particles (mol/l)	1722	0.06	0.01	0.12	0.016	1722	0.07	0.02	0.12	0.011	1722	0.19	0.13	0.24	<0.0001	1722	0.21	0.15	0.27	<0.0001
Total lipids in very small VLDL (mmol/l)	1722	0.08	0.02	0.13	0.006	1722	0.08	0.03	0.14	0.004	1722	0.22	0.16	0.28	<0.0001	1722	0.24	0.18	0.31	<0.0001
Phospholipids in very small VLDL (mmol/l)	1722	0.05	0.00	0.10	0.044	1722	0.06	0.01	0.11	0.030	1722	0.16	0.11	0.22	<0.0001	1722	0.19	0.12	0.25	<0.0001
Total cholesterol in very small VLDL (mmol/l)	1722	0.07	0.02	0.13	0.013	1722	0.08	0.02	0.13	0.011	1722	0.21	0.15	0.27	<0.0001	1722	0.23	0.17	0.30	<0.0001
Cholesterol esters in very small VLDL (mmol/l)	1722	0.08	0.03	0.14	0.004	1722	0.09	0.03	0.15	0.004	1722	0.23	0.17	0.29	<0.0001	1722	0.26	0.19	0.33	<0.0001
Free cholesterol in very small VLDL (mmol/l)	1722	0.03	-0.02	0.09	0.197	1722	0.04	-0.01	0.09	0.145	1722	0.13	0.07	0.18	<0.0001	1722	0.14	0.07	0.21	<0.0001
Triglycerides in very small VLDL (mmol/l)	1722	0.08	0.03	0.13	0.003	1722	0.08	0.03	0.14	0.001	1722	0.20	0.14	0.25	<0.0001	1722	0.22	0.16	0.28	<0.0001
Concentration of IDL particles (mol/l)	1722	0.04	-0.01	0.09	0.118	1722	0.04	-0.01	0.09	0.093	1722	0.13	0.07	0.18	<0.0001	1722	0.15	0.08	0.21	<0.0001
Total lipids in IDL (mmol/l)	1722	0.05	-0.01	0.10	0.082	1722	0.05	0.00	0.10	0.057	1722	0.14	0.08	0.20	<0.0001	1722	0.16	0.10	0.23	<0.0001
Phospholipids in IDL (mmol/l)	1722	0.04	-0.01	0.08	0.164	1722	0.04	-0.01	0.09	0.114	1722	0.11	0.06	0.17	<0.0001	1722	0.13	0.07	0.19	<0.0001
Total cholesterol in IDL (mmol/l)	1722	0.05	0.00	0.11	0.051	1722	0.06	0.00	0.11	0.034	1722	0.16	0.10	0.22	<0.0001	1722	0.18	0.12	0.25	<0.0001
Cholesterol esters in IDL (mmol/l)	1722	0.06	0.01	0.12	0.019	1722	0.07	0.02	0.12	0.012	1722	0.19	0.13	0.25	<0.0001	1722	0.21	0.15	0.28	<0.0001

Online Table 2 Associations of body mass index (BMI) and fat mass index at age 10y and 18y with cardiometabolic traits at age 18y in ALSPAC

	At age 10y										At age 18y									
	BMI (per 2.7 kg/m^2 higher)					Fat mass index (per 2.3 kg/m^2 higher)					BMI (per 4.0 kg/m^2 higher)					Fat mass index (per 3.7 kg/m^2 higher)				
	Adj. for age, sex, ethnicity, maternal education					*Adj. for age, sex, ethnicity, maternal education*					*Adj. for age, sex, ethnicity, maternal education, smoking, alcohol, puberty timing*					*Adj. for age, sex, ethnicity, maternal education, smoking, alcohol, puberty timing*				
**Standardized outcome at age 18y**	**N**	**Beta**	**LCL**	**UCL**	**P-value**	**N**	**Beta**	**LCL**	**UCL**	**P-value**	**N**	**Beta**	**LCL**	**UCL**	**P-value**	**N**	**Beta**	**LCL**	**UCL**	**P-value**
Free cholesterol in IDL (mmol/l)	1722	0.02	-0.03	0.07	0.374	1722	0.03	-0.02	0.08	0.292	1722	0.09	0.03	0.14	0.002	1722	0.11	0.04	0.17	0.001
Triglycerides in IDL (mmol/l)	1722	0.01	-0.04	0.06	0.652	1722	0.01	-0.04	0.06	0.642	1722	0.04	-0.01	0.09	0.159	1722	0.05	-0.01	0.10	0.099
Concentration of large LDL particles (mol/l)	1722	0.05	0.00	0.10	0.055	1722	0.06	0.01	0.11	0.025	1722	0.13	0.08	0.19	<0.0001	1722	0.16	0.10	0.22	<0.0001
Total lipids in large LDL (mmol/l)	1722	0.05	0.00	0.10	0.054	1722	0.06	0.01	0.11	0.026	1722	0.14	0.08	0.19	<0.0001	1722	0.16	0.10	0.22	<0.0001
Phospholipids in large LDL (mmol/l)	1722	0.06	0.01	0.11	0.024	1722	0.07	0.02	0.12	0.010	1722	0.16	0.10	0.21	<0.0001	1722	0.18	0.12	0.25	<0.0001
Total cholesterol in large LDL (mmol/l)	1722	0.05	0.00	0.10	0.048	1722	0.06	0.01	0.11	0.022	1722	0.14	0.09	0.20	<0.0001	1722	0.17	0.11	0.23	<0.0001
Cholesterol esters in large LDL (mmol/l)	1722	0.06	0.01	0.11	0.028	1722	0.07	0.01	0.12	0.012	1722	0.16	0.10	0.21	<0.0001	1722	0.19	0.12	0.25	<0.0001
Free cholesterol in large LDL (mmol/l)	1722	0.03	-0.02	0.08	0.210	1722	0.04	-0.01	0.09	0.125	1722	0.10	0.05	0.16	0.0003	1722	0.13	0.06	0.19	<0.0001
Triglycerides in large LDL (mmol/l)	1722	0.00	-0.04	0.05	0.899	1722	0.00	-0.04	0.05	0.851	1722	0.01	-0.04	0.06	0.836	1722	0.01	-0.04	0.07	0.597
Concentration of medium LDL particles (mol/l)	1722	0.06	0.01	0.11	0.019	1722	0.07	0.02	0.12	0.006	1722	0.15	0.10	0.20	<0.0001	1722	0.18	0.12	0.24	<0.0001
Total lipids in medium LDL (mmol/l)	1722	0.06	0.01	0.11	0.027	1722	0.07	0.02	0.12	0.009	1722	0.15	0.09	0.20	<0.0001	1722	0.18	0.12	0.24	<0.0001
Phospholipids in medium LDL (mmol/l)	1722	0.07	0.02	0.13	0.004	1722	0.09	0.04	0.14	0.001	1722	0.19	0.13	0.24	<0.0001	1722	0.22	0.16	0.28	<0.0001
Total cholesterol in medium LDL (mmol/l)	1722	0.06	0.01	0.11	0.029	1722	0.07	0.01	0.12	0.012	1722	0.15	0.10	0.21	<0.0001	1722	0.18	0.12	0.24	<0.0001
Cholesterol esters in medium LDL (mmol/l)	1722	0.06	0.01	0.11	0.027	1722	0.07	0.02	0.12	0.011	1722	0.15	0.10	0.21	<0.0001	1722	0.18	0.12	0.24	<0.0001
Free cholesterol in medium LDL (mmol/l)	1722	0.05	0.00	0.10	0.045	1722	0.07	0.01	0.12	0.014	1722	0.14	0.08	0.20	<0.0001	1722	0.17	0.11	0.23	<0.0001
Triglycerides in medium LDL (mmol/l)	1722	0.00	-0.04	0.05	0.942	1722	0.01	-0.04	0.05	0.786	1722	0.00	-0.05	0.05	0.994	1722	0.01	-0.04	0.07	0.604
Concentration of small LDL particles (mol/l)	1722	0.06	0.01	0.11	0.015	1722	0.08	0.03	0.13	0.003	1722	0.15	0.09	0.20	<0.0001	1722	0.18	0.12	0.24	<0.0001
Total lipids in small LDL (mmol/l)	1722	0.06	0.01	0.11	0.018	1722	0.07	0.02	0.13	0.005	1722	0.15	0.10	0.21	<0.0001	1722	0.18	0.12	0.25	<0.0001
Phospholipids in small LDL (mmol/l)	1722	0.07	0.02	0.12	0.006	1722	0.09	0.04	0.14	0.001	1722	0.17	0.12	0.22	<0.0001	1722	0.20	0.14	0.27	<0.0001
Total cholesterol in small LDL (mmol/l)	1722	0.06	0.01	0.11	0.031	1722	0.07	0.02	0.12	0.010	1722	0.15	0.09	0.20	<0.0001	1722	0.18	0.11	0.24	<0.0001
Cholesterol esters in small LDL (mmol/l)	1722	0.06	0.01	0.11	0.030	1722	0.07	0.02	0.12	0.011	1722	0.15	0.09	0.21	<0.0001	1722	0.18	0.12	0.24	<0.0001
Free cholesterol in small LDL (mmol/l)	1722	0.05	0.00	0.10	0.047	1722	0.07	0.02	0.12	0.010	1722	0.14	0.08	0.19	<0.0001	1722	0.17	0.11	0.23	<0.0001
Triglycerides in small LDL (mmol/l)	1722	0.05	0.00	0.10	0.038	1722	0.06	0.01	0.10	0.017	1722	0.11	0.05	0.16	<0.0001	1722	0.13	0.07	0.19	<0.0001
Concentration of very large HDL particles (mol/l)	1722	-0.09	-0.14	-0.04	0.001	1722	-0.09	-0.14	-0.03	0.001	1722	-0.21	-0.26	-0.16	<0.0001	1722	-0.22	-0.27	-0.16	<0.0001
Total lipids in very large HDL (mmol/l)	1722	-0.08	-0.13	-0.03	0.002	1722	-0.08	-0.13	-0.03	0.003	1722	-0.20	-0.25	-0.15	<0.0001	1722	-0.21	-0.26	-0.15	<0.0001
Phospholipids in very large HDL (mmol/l)	1722	-0.10	-0.15	-0.05	0.0002	1722	-0.10	-0.15	-0.05	0.0002	1722	-0.23	-0.28	-0.18	<0.0001	1722	-0.24	-0.29	-0.19	<0.0001
Total cholesterol in very large HDL (mmol/l)	1722	-0.06	-0.11	-0.01	0.022	1722	-0.05	-0.10	0.00	0.055	1722	-0.16	-0.21	-0.11	<0.0001	1722	-0.16	-0.21	-0.10	<0.0001
Cholesterol esters in very large HDL (mmol/l)	1722	-0.05	-0.10	0.00	0.046	1722	-0.04	-0.09	0.01	0.114	1722	-0.14	-0.20	-0.09	<0.0001	1722	-0.14	-0.19	-0.08	<0.0001
Free cholesterol in very large HDL (mmol/l)	1722	-0.08	-0.13	-0.03	0.003	1722	-0.07	-0.13	-0.02	0.006	1722	-0.19	-0.24	-0.14	<0.0001	1722	-0.19	-0.25	-0.14	<0.0001
Triglycerides in very large HDL (mmol/l)	1722	0.01	-0.05	0.06	0.821	1722	-0.01	-0.06	0.05	0.828	1722	0.03	-0.03	0.08	0.377	1722	0.02	-0.04	0.08	0.529
Concentration of large HDL particles (mol/l)	1722	-0.10	-0.16	-0.05	0.0001	1722	-0.10	-0.16	-0.05	0.0002	1722	-0.26	-0.31	-0.21	<0.0001	1722	-0.28	-0.33	-0.23	<0.0001
Total lipids in large HDL (mmol/l)	1722	-0.11	-0.16	-0.05	<0.0001	1722	-0.10	-0.16	-0.05	0.0002	1722	-0.26	-0.30	-0.21	<0.0001	1722	-0.28	-0.33	-0.22	<0.0001
Phospholipids in large HDL (mmol/l)	1722	-0.09	-0.14	-0.04	0.0005	1722	-0.09	-0.14	-0.04	0.001	1722	-0.23	-0.28	-0.18	<0.0001	1722	-0.25	-0.30	-0.20	<0.0001
Total cholesterol in large HDL (mmol/l)	1722	-0.12	-0.17	-0.06	<0.0001	1722	-0.12	-0.17	-0.06	<0.0001	1722	-0.28	-0.33	-0.23	<0.0001	1722	-0.30	-0.35	-0.25	<0.0001
Cholesterol esters in large HDL (mmol/l)	1722	-0.12	-0.17	-0.06	<0.0001	1722	-0.12	-0.17	-0.06	<0.0001	1722	-0.28	-0.33	-0.23	<0.0001	1722	-0.30	-0.36	-0.25	<0.0001
Free cholesterol in large HDL (mmol/l)	1722	-0.11	-0.17	-0.06	<0.0001	1722	-0.11	-0.17	-0.06	<0.0001	1722	-0.27	-0.32	-0.22	<0.0001	1722	-0.29	-0.34	-0.23	<0.0001
Triglycerides in large HDL (mmol/l)	1722	-0.01	-0.05	0.04	0.810	1722	-0.02	-0.07	0.03	0.438	1722	0.00	-0.05	0.05	0.937	1722	-0.02	-0.08	0.03	0.446
Concentration of medium HDL particles (mol/l)	1722	-0.01	-0.06	0.04	0.660	1722	0.00	-0.05	0.06	0.883	1722	-0.07	-0.12	-0.02	0.009	1722	-0.07	-0.12	-0.01	0.020
Total lipids in medium HDL (mmol/l)	1722	-0.02	-0.07	0.03	0.455	1722	0.00	-0.06	0.05	0.878	1722	-0.09	-0.14	-0.03	0.001	1722	-0.08	-0.14	-0.03	0.004
Phospholipids in medium HDL (mmol/l)	1722	-0.03	-0.08	0.03	0.349	1722	-0.01	-0.06	0.04	0.723	1722	-0.10	-0.15	-0.05	0.0002	1722	-0.09	-0.15	-0.04	0.001
Total cholesterol in medium HDL (mmol/l)	1722	-0.03	-0.08	0.02	0.251	1722	-0.02	-0.07	0.04	0.582	1722	-0.11	-0.16	-0.05	<0.0001	1722	-0.11	-0.17	-0.05	0.0002
Cholesterol esters in medium HDL (mmol/l)	1722	-0.03	-0.09	0.02	0.212	1722	-0.02	-0.07	0.04	0.515	1722	-0.12	-0.17	-0.06	<0.0001	1722	-0.12	-0.18	-0.06	<0.0001
Free cholesterol in medium HDL (mmol/l)	1722	-0.01	-0.06	0.04	0.599	1722	0.00	-0.05	0.05	0.931	1722	-0.06	-0.11	-0.01	0.021	1722	-0.06	-0.12	0.00	0.039
Triglycerides in medium HDL (mmol/l)	1722	0.11	0.06	0.16	<0.0001	1722	0.11	0.06	0.16	<0.0001	1722	0.24	0.19	0.30	<0.0001	1722	0.26	0.20	0.32	<0.0001
Concentration of small HDL particles (mol/l)	1722	0.05	-0.01	0.10	0.095	1722	0.07	0.01	0.12	0.018	1722	0.04	-0.01	0.10	0.115	1722	0.06	0.00	0.12	0.038
Total lipids in small HDL (mmol/l)	1722	0.01	-0.04	0.07	0.598	1722	0.03	-0.02	0.09	0.210	1722	-0.01	-0.06	0.04	0.708	1722	0.01	-0.05	0.06	0.840
Phospholipids in small HDL (mmol/l)	1722	0.04	-0.02	0.09	0.177	1722	0.06	0.00	0.11	0.053	1722	0.01	-0.04	0.07	0.641	1722	0.02	-0.04	0.08	0.455
Total cholesterol in small HDL (mmol/l)	1722	-0.03	-0.08	0.02	0.264	1722	-0.01	-0.06	0.04	0.774	1722	-0.06	-0.11	-0.01	0.011	1722	-0.04	-0.10	0.01	0.097
Cholesterol esters in small HDL (mmol/l)	1722	-0.03	-0.07	0.02	0.243	1722	-0.01	-0.06	0.04	0.736	1722	-0.05	-0.09	0.00	0.055	1722	-0.02	-0.08	0.03	0.333
Free cholesterol in small HDL (mmol/l)	1722	-0.02	-0.08	0.04	0.495	1722	0.00	-0.06	0.06	0.915	1722	-0.10	-0.15	-0.04	0.001	1722	-0.10	-0.16	-0.03	0.002
Triglycerides in small HDL (mmol/l)	1722	0.09	0.04	0.14	0.001	1722	0.09	0.04	0.14	0.001	1722	0.18	0.12	0.23	<0.0001	1722	0.19	0.13	0.25	<0.0001
Phospholipids to total lipds ratio in chylomicrons and extremely large VLDL (%)	1722	0.05	0.02	0.07	0.0003	1722	0.05	0.03	0.08	<0.0001	1722	0.02	0.00	0.04	0.118	1722	0.03	0.00	0.06	0.027
Total cholesterol to total lipids ratio in chylomicrons and extremely large VLDL (%)	1722	0.04	-0.01	0.09	0.098	1722	0.03	-0.02	0.08	0.196	1722	0.14	0.09	0.18	<0.0001	1722	0.13	0.08	0.18	<0.0001
Cholesterol esters to total lipids ratio in chylomicrons and extremely large VLDL (%)	1722	0.01	-0.04	0.06	0.807	1722	-0.01	-0.06	0.05	0.836	1722	0.10	0.05	0.15	<0.0001	1722	0.09	0.04	0.14	0.001
Free cholesterol to total lipids ratio in chylomicrons and extremely large VLDL (%)	1722	0.12	0.07	0.17	<0.0001	1722	0.13	0.08	0.17	<0.0001	1722	0.15	0.10	0.19	<0.0001	1722	0.16	0.11	0.21	<0.0001
Triglycerides to total lipids ratio in chylomicrons and extremely large VLDL (%)	1722	-0.06	-0.11	-0.01	0.019	1722	-0.05	-0.10	0.01	0.084	1722	-0.13	-0.17	-0.08	<0.0001	1722	-0.12	-0.17	-0.07	<0.0001
Phospholipids to total lipds ratio in very large VLDL (%)	1722	0.12	0.07	0.16	<0.0001	1722	0.13	0.08	0.17	<0.0001	1722	0.21	0.16	0.25	<0.0001	1722	0.23	0.18	0.28	<0.0001
Total cholesterol to total lipids ratio in very large VLDL (%)	1722	-0.03	-0.08	0.02	0.195	1722	-0.04	-0.09	0.00	0.076	1722	-0.03	-0.07	0.02	0.239	1722	-0.05	-0.10	0.00	0.055
Cholesterol esters to total lipids ratio in very large VLDL (%)	1722	-0.04	-0.08	0.00	0.030	1722	-0.05	-0.08	-0.01	0.007	1722	-0.03	-0.07	0.00	0.078	1722	-0.05	-0.09	-0.01	0.006

Online Table 2 Associations of body mass index (BMI) and fat mass index at age 10y and 18y with cardiometabolic traits at age 18y in ALSPAC

	At age 10y										At age 18y									
	BMI (per 2.7 kg/m^2 higher)					Fat mass index (per 2.3 kg/m^2 higher)					BMI (per 4.0 kg/m^2 higher)					Fat mass index (per 3.7 kg/m^2 higher)				
	Adj. for age, sex, ethnicity, maternal education					*Adj. for age, sex, ethnicity, maternal education*					*Adj. for age, sex, ethnicity, maternal education, smoking, alcohol, puberty timing*					*Adj. for age, sex, ethnicity, maternal education, smoking, alcohol, puberty timing*				
**Standardized outcome at age 18y**	**N**	**Beta**	**LCL**	**UCL**	**P-value**	**N**	**Beta**	**LCL**	**UCL**	**P-value**	**N**	**Beta**	**LCL**	**UCL**	**P-value**	**N**	**Beta**	**LCL**	**UCL**	**P-value**
Free cholesterol to total lipids ratio in very large VLDL (%)	1722	0.00	-0.03	0.03	0.975	1722	0.00	-0.03	0.03	0.943	1722	0.01	-0.02	0.04	0.598	1722	0.00	-0.04	0.04	0.972
Triglycerides to total lipids ratio in very large VLDL (%)	1722	0.01	-0.04	0.05	0.732	1722	0.01	-0.03	0.06	0.643	1722	-0.05	-0.10	0.00	0.041	1722	-0.04	-0.09	0.02	0.173
Phospholipids to total lipds ratio in large VLDL (%)	1722	0.09	0.05	0.14	0.0001	1722	0.09	0.05	0.14	<0.0001	1722	0.15	0.10	0.19	<0.0001	1722	0.17	0.12	0.22	<0.0001
Total cholesterol to total lipids ratio in large VLDL (%)	1722	0.07	0.03	0.12	0.002	1722	0.06	0.01	0.11	0.019	1722	0.18	0.14	0.23	<0.0001	1722	0.17	0.12	0.23	<0.0001
Cholesterol esters to total lipids ratio in large VLDL (%)	1722	0.01	-0.02	0.05	0.458	1722	-0.01	-0.05	0.03	0.645	1722	0.08	0.04	0.13	<0.0001	1722	0.06	0.01	0.10	0.021
Free cholesterol to total lipids ratio in large VLDL (%)	1722	0.05	0.03	0.07	<0.0001	1722	0.05	0.03	0.07	<0.0001	1722	0.09	0.07	0.11	<0.0001	1722	0.10	0.08	0.12	<0.0001
Triglycerides to total lipids ratio in large VLDL (%)	1722	-0.08	-0.13	-0.03	0.001	1722	-0.07	-0.12	-0.02	0.006	1722	-0.18	-0.23	-0.13	<0.0001	1722	-0.18	-0.24	-0.13	<0.0001
Phospholipids to total lipds ratio in medium VLDL (%)	1722	-0.07	-0.11	-0.02	0.009	1722	-0.07	-0.12	-0.02	0.009	1722	-0.17	-0.21	-0.12	<0.0001	1722	-0.19	-0.24	-0.14	<0.0001
Total cholesterol to total lipids ratio in medium VLDL (%)	1722	0.03	-0.02	0.08	0.199	1722	0.02	-0.03	0.07	0.359	1722	0.11	0.06	0.16	<0.0001	1722	0.10	0.05	0.16	0.0002
Cholesterol esters to total lipids ratio in medium VLDL (%)	1722	0.01	-0.03	0.06	0.577	1722	0.00	-0.05	0.05	0.919	1722	0.10	0.05	0.15	0.0001	1722	0.09	0.03	0.14	0.002
Free cholesterol to total lipids ratio in medium VLDL (%)	1722	0.07	0.02	0.11	0.007	1722	0.07	0.02	0.12	0.004	1722	0.10	0.05	0.15	<0.0001	1722	0.11	0.06	0.17	<0.0001
Triglycerides to total lipids ratio in medium VLDL (%)	1722	-0.02	-0.06	0.03	0.521	1722	-0.01	-0.05	0.04	0.773	1722	-0.07	-0.12	-0.02	0.005	1722	-0.06	-0.11	-0.01	0.029
Phospholipids to total lipds ratio in small VLDL (%)	1722	-0.11	-0.17	-0.05	0.0003	1722	-0.11	-0.17	-0.04	0.001	1722	-0.27	-0.33	-0.22	<0.0001	1722	-0.30	-0.36	-0.24	<0.0001
Total cholesterol to total lipids ratio in small VLDL (%)	1722	-0.01	-0.07	0.04	0.586	1722	-0.02	-0.07	0.03	0.447	1722	0.02	-0.03	0.08	0.439	1722	0.02	-0.04	0.09	0.451
Cholesterol esters to total lipids ratio in small VLDL (%)	1722	0.00	-0.05	0.05	0.988	1722	-0.01	-0.06	0.05	0.791	1722	0.05	0.00	0.11	0.059	1722	0.06	0.00	0.12	0.062
Free cholesterol to total lipids ratio in small VLDL (%)	1722	-0.09	-0.15	-0.04	0.0005	1722	-0.09	-0.14	-0.04	0.001	1722	-0.21	-0.26	-0.16	<0.0001	1722	-0.23	-0.28	-0.18	<0.0001
Triglycerides to total lipids ratio in small VLDL (%)	1722	0.05	0.00	0.10	0.034	1722	0.06	0.01	0.11	0.023	1722	0.07	0.02	0.13	0.013	1722	0.08	0.02	0.14	0.014
Phospholipids to total lipds ratio in very small VLDL (%)	1722	0.00	-0.05	0.04	0.912	1722	0.00	-0.04	0.05	0.953	1722	0.00	-0.05	0.05	0.976	1722	0.01	-0.05	0.06	0.837
Total cholesterol to total lipids ratio in very small VLDL (%)	1722	-0.01	-0.06	0.04	0.674	1722	-0.01	-0.06	0.03	0.555	1722	-0.01	-0.07	0.04	0.645	1722	-0.02	-0.08	0.04	0.505
Cholesterol esters to total lipids ratio in very small VLDL (%)	1722	0.02	-0.03	0.07	0.454	1722	0.01	-0.04	0.07	0.585	1722	0.05	0.00	0.10	0.047	1722	0.05	0.00	0.11	0.060
Free cholesterol to total lipids ratio in very small VLDL (%)	1722	-0.10	-0.15	-0.05	<0.0001	1722	-0.10	-0.15	-0.06	<0.0001	1722	-0.22	-0.28	-0.16	<0.0001	1722	-0.25	-0.32	-0.19	<0.0001
Triglycerides to total lipids ratio in very small VLDL (%)	1722	0.02	-0.04	0.07	0.555	1722	0.02	-0.03	0.07	0.499	1722	0.01	-0.04	0.07	0.612	1722	0.02	-0.04	0.08	0.526
Phospholipids to total lipds ratio in IDL (%)	1722	-0.09	-0.14	-0.03	0.003	1722	-0.09	-0.14	-0.03	0.004	1722	-0.25	-0.31	-0.19	<0.0001	1722	-0.26	-0.32	-0.19	<0.0001
Total cholesterol to total lipids ratio in IDL (%)	1722	0.07	0.01	0.13	0.025	1722	0.07	0.01	0.13	0.025	1722	0.18	0.13	0.24	<0.0001	1722	0.19	0.13	0.25	<0.0001
Cholesterol esters to total lipids ratio in IDL (%)	1722	0.10	0.04	0.16	0.001	1722	0.10	0.04	0.16	0.001	1722	0.26	0.21	0.31	<0.0001	1722	0.27	0.21	0.33	<0.0001
Free cholesterol to total lipids ratio in IDL (%)	1722	-0.07	-0.12	-0.02	0.007	1722	-0.07	-0.12	-0.02	0.011	1722	-0.17	-0.23	-0.11	<0.0001	1722	-0.18	-0.25	-0.11	<0.0001
Triglycerides to total lipids ratio in IDL (%)	1722	-0.04	-0.10	0.01	0.128	1722	-0.05	-0.11	0.01	0.113	1722	-0.11	-0.17	-0.06	<0.0001	1722	-0.12	-0.18	-0.06	0.0001
Phospholipids to total lipds ratio in large LDL (%)	1722	-0.04	-0.09	0.01	0.102	1722	-0.05	-0.10	0.00	0.053	1722	-0.08	-0.13	-0.03	0.004	1722	-0.09	-0.15	-0.03	0.002
Total cholesterol to total lipids ratio in large LDL (%)	1722	0.07	0.01	0.12	0.023	1722	0.08	0.02	0.14	0.013	1722	0.15	0.10	0.21	<0.0001	1722	0.17	0.11	0.23	<0.0001
Cholesterol esters to total lipids ratio in large LDL (%)	1722	0.10	0.04	0.15	0.001	1722	0.11	0.05	0.16	0.0003	1722	0.21	0.15	0.26	<0.0001	1722	0.23	0.17	0.29	<0.0001
Free cholesterol to total lipids ratio in large LDL (%)	1722	-0.12	-0.17	-0.07	<0.0001	1722	-0.13	-0.18	-0.08	<0.0001	1722	-0.23	-0.29	-0.18	<0.0001	1722	-0.26	-0.32	-0.20	<0.0001
Triglycerides to total lipids ratio in large LDL (%)	1722	-0.06	-0.12	0.00	0.049	1722	-0.07	-0.13	0.00	0.040	1722	-0.16	-0.22	-0.10	<0.0001	1722	-0.17	-0.23	-0.11	<0.0001
Phospholipids to total lipds ratio in medium LDL (%)	1722	-0.04	-0.10	0.01	0.082	1722	-0.05	-0.10	0.00	0.059	1722	-0.08	-0.13	-0.02	0.005	1722	-0.08	-0.14	-0.02	0.006
Total cholesterol to total lipids ratio in medium LDL (%)	1722	0.06	0.01	0.12	0.022	1722	0.07	0.01	0.12	0.021	1722	0.13	0.07	0.19	<0.0001	1722	0.13	0.07	0.20	<0.0001
Cholesterol esters to total lipids ratio in medium LDL (%)	1722	0.08	0.02	0.13	0.005	1722	0.08	0.03	0.14	0.003	1722	0.15	0.10	0.20	<0.0001	1722	0.16	0.10	0.22	<0.0001
Free cholesterol to total lipids ratio in medium LDL (%)	1722	-0.08	-0.13	-0.03	0.001	1722	-0.09	-0.14	-0.04	0.0003	1722	-0.15	-0.20	-0.10	<0.0001	1722	-0.17	-0.23	-0.12	<0.0001
Triglycerides to total lipids ratio in medium LDL (%)	1722	-0.05	-0.10	0.00	0.073	1722	-0.05	-0.11	0.01	0.088	1722	-0.13	-0.18	-0.08	<0.0001	1722	-0.13	-0.18	-0.08	<0.0001
Phospholipids to total lipds ratio in small LDL (%)	1722	-0.06	-0.11	-0.01	0.022	1722	-0.07	-0.12	-0.01	0.012	1722	-0.12	-0.17	-0.06	<0.0001	1722	-0.13	-0.19	-0.07	<0.0001
Total cholesterol to total lipids ratio in small LDL (%)	1722	0.05	-0.01	0.10	0.078	1722	0.05	0.00	0.11	0.057	1722	0.10	0.04	0.16	0.000	1722	0.11	0.05	0.17	0.001
Cholesterol esters to total lipids ratio in small LDL (%)	1722	0.07	0.01	0.12	0.014	1722	0.07	0.02	0.13	0.007	1722	0.13	0.08	0.19	<0.0001	1722	0.14	0.09	0.20	<0.0001
Free cholesterol to total lipids ratio in small LDL (%)	1722	-0.08	-0.13	-0.03	0.001	1722	-0.09	-0.14	-0.04	0.0002	1722	-0.15	-0.20	-0.10	<0.0001	1722	-0.17	-0.23	-0.12	<0.0001
Triglycerides to total lipids ratio in small LDL (%)	1722	0.02	-0.04	0.07	0.550	1722	0.02	-0.04	0.08	0.502	1722	0.02	-0.04	0.08	0.536	1722	0.03	-0.03	0.09	0.354
Phospholipids to total lipids ratio in very large HDL (%)	1722	-0.13	-0.18	-0.08	<0.0001	1722	-0.13	-0.19	-0.08	<0.0001	1722	-0.26	-0.31	-0.21	<0.0001	1722	-0.28	-0.34	-0.22	<0.0001
Total cholesterol to total lipids ratio in very large HDL (%)	1722	0.11	0.07	0.16	<0.0001	1722	0.12	0.07	0.17	<0.0001	1722	0.22	0.17	0.27	<0.0001	1722	0.24	0.19	0.30	<0.0001
Cholesterol esters to total lipids ratio in very large HDL (%)	1722	0.11	0.06	0.16	<0.0001	1722	0.12	0.07	0.17	<0.0001	1722	0.22	0.17	0.27	<0.0001	1722	0.24	0.18	0.29	<0.0001
Free cholesterol to total lipids ratio in very large HDL (%)	1722	-0.01	-0.06	0.04	0.695	1722	0.01	-0.05	0.06	0.825	1722	-0.04	-0.10	0.02	0.222	1722	-0.02	-0.08	0.04	0.538
Triglycerides to total lipids ratio in very large HDL (%)	1722	0.10	0.04	0.17	0.002	1722	0.09	0.03	0.15	0.006	1722	0.25	0.17	0.32	<0.0001	1722	0.25	0.17	0.33	<0.0001
Phospholipids to total lipids ratio in large HDL (%)	1722	0.17	0.11	0.23	<0.0001	1722	0.18	0.12	0.24	<0.0001	1722	0.34	0.28	0.39	<0.0001	1722	0.37	0.31	0.44	<0.0001
Total cholesterol to total lipids ratio in large HDL (%)	1722	-0.18	-0.24	-0.11	<0.0001	1722	-0.18	-0.24	-0.12	<0.0001	1722	-0.36	-0.43	-0.30	<0.0001	1722	-0.39	-0.46	-0.32	<0.0001
Cholesterol esters to total lipids ratio in large HDL (%)	1722	-0.18	-0.24	-0.12	<0.0001	1722	-0.19	-0.25	-0.12	<0.0001	1722	-0.37	-0.44	-0.31	<0.0001	1722	-0.40	-0.48	-0.33	<0.0001
Free cholesterol to total lipids ratio in large HDL (%)	1722	-0.13	-0.18	-0.07	<0.0001	1722	-0.13	-0.18	-0.07	<0.0001	1722	-0.25	-0.31	-0.19	<0.0001	1722	-0.26	-0.33	-0.20	<0.0001
Triglycerides to total lipids ratio in large HDL (%)	1722	0.15	0.08	0.21	<0.0001	1722	0.13	0.07	0.20	<0.0001	1722	0.32	0.25	0.39	<0.0001	1722	0.33	0.26	0.41	<0.0001
Phospholipids to total lipds ratio in medium HDL (%)	1722	-0.04	-0.09	0.01	0.149	1722	-0.03	-0.08	0.02	0.290	1722	-0.09	-0.15	-0.04	0.001	1722	-0.08	-0.14	-0.03	0.004
Total cholesterol to total lipids ratio in medium HDL (%)	1722	-0.04	-0.09	0.01	0.128	1722	-0.04	-0.09	0.01	0.098	1722	-0.08	-0.14	-0.02	0.006	1722	-0.10	-0.15	-0.04	0.001
Cholesterol esters to total lipids ratio in medium HDL (%)	1722	-0.05	-0.09	0.00	0.072	1722	-0.05	-0.10	0.00	0.054	1722	-0.10	-0.15	-0.04	0.0005	1722	-0.12	-0.17	-0.06	<0.0001
Free cholesterol to total lipids ratio in medium HDL (%)	1722	0.02	-0.02	0.07	0.295	1722	0.03	-0.02	0.07	0.294	1722	0.07	0.02	0.13	0.008	1722	0.07	0.02	0.13	0.013
Triglycerides to total lipids ratio in medium HDL (%)	1722	0.14	0.08	0.20	<0.0001	1722	0.13	0.08	0.19	<0.0001	1722	0.32	0.26	0.39	<0.0001	1722	0.34	0.27	0.41	<0.0001
Phospholipids to total lipds ratio in small HDL (%)	1722	0.05	0.00	0.09	0.045	1722	0.04	-0.01	0.08	0.120	1722	0.04	0.00	0.09	0.073	1722	0.03	-0.03	0.08	0.309
Total cholesterol to total lipids ratio in small HDL (%)	1722	-0.07	-0.12	-0.03	0.002	1722	-0.06	-0.11	-0.01	0.014	1722	-0.10	-0.15	-0.05	<0.0001	1722	-0.09	-0.14	-0.03	0.001

Online Table 2 Associations of body mass index (BMI) and fat mass index at age 10y and 18y with cardiometabolic traits at age 18y in ALSPAC

	**At age 10y**										**At age 18y**									
	**BMI (per 2.7 kg/m^2 higher)**					**Fat mass index (per 2.3 kg/m^2 higher)**					**BMI (per 4.0 kg/m^2 higher)**					**Fat mass index (per 3.7 kg/m^2 higher)**				
	Adj. for age, sex, ethnicity, maternal education					*Adj. for age, sex, ethnicity, maternal education*					*Adj. for age, sex, ethnicity, maternal education, smoking, alcohol, puberty timing*					*Adj. for age, sex, ethnicity, maternal education, smoking, alcohol, puberty timing*				
**Standardized outcome at age 18y**	**N**	**Beta**	**LCL**	**UCL**	**P-value**	**N**	**Beta**	**LCL**	**UCL**	**P-value**	**N**	**Beta**	**LCL**	**UCL**	**P-value**	**N**	**Beta**	**LCL**	**UCL**	**P-value**
Cholesterol esters to total lipids ratio in small HDL (%)	1722	-0.05	-0.10	-0.01	0.025	1722	-0.04	-0.09	0.01	0.099	1722	-0.06	-0.11	-0.01	0.029	1722	-0.04	-0.09	0.02	0.189
Free cholesterol to total lipids ratio in small HDL (%)	1722	-0.10	-0.15	-0.04	0.0004	1722	-0.10	-0.16	-0.05	0.0002	1722	-0.26	-0.31	-0.20	<0.0001	1722	-0.30	-0.36	-0.23	<0.0001
Triglycerides to total lipids ratio in small HDL (%)	1722	0.10	0.04	0.16	0.001	1722	0.09	0.03	0.14	0.003	1722	0.21	0.14	0.28	<0.0001	1722	0.22	0.15	0.29	<0.0001
Mean diameter for VLDL particles (nm)	1722	0.14	0.09	0.20	<0.0001	1722	0.15	0.10	0.21	<0.0001	1722	0.29	0.24	0.35	<0.0001	1722	0.32	0.26	0.38	<0.0001
Mean diameter for LDL particles (nm)	1722	-0.09	-0.14	-0.04	0.0004	1722	-0.13	-0.18	-0.08	<0.0001	1722	-0.13	-0.18	-0.07	<0.0001	1722	-0.17	-0.22	-0.11	<0.0001
Mean diameter for HDL particles (nm)	1722	-0.12	-0.17	-0.06	<0.0001	1722	-0.12	-0.17	-0.07	<0.0001	1722	-0.25	-0.30	-0.21	<0.0001	1722	-0.27	-0.32	-0.22	<0.0001
Serum total cholesterol (mmol/l)	1722	0.04	0.00	0.09	0.077	1722	0.06	0.01	0.10	0.028	1722	0.12	0.07	0.17	<0.0001	1722	0.15	0.09	0.21	<0.0001
Total cholesterol in VLDL (mmol/l)	1722	0.14	0.08	0.20	<0.0001	1722	0.14	0.08	0.20	<0.0001	1722	0.34	0.28	0.40	<0.0001	1722	0.37	0.31	0.44	<0.0001
Remnant cholesterol (non-HDL, non-LDL -cholesterol) (mmol/l)	1722	0.11	0.05	0.17	0.0002	1722	0.11	0.06	0.17	0.0001	1722	0.28	0.22	0.34	<0.0001	1722	0.32	0.25	0.38	<0.0001
Total cholesterol in LDL (mmol/l)	1722	0.05	0.00	0.11	0.038	1722	0.06	0.01	0.12	0.016	1722	0.15	0.09	0.20	<0.0001	1722	0.18	0.11	0.24	<0.0001
Total cholesterol in HDL (mmol/l)	1722	-0.08	-0.14	-0.03	0.002	1722	-0.07	-0.13	-0.02	0.008	1722	-0.21	-0.26	-0.16	<0.0001	1722	-0.22	-0.27	-0.16	<0.0001
Total cholesterol in HDL2 (mmol/l)	1722	-0.10	-0.15	-0.04	0.0003	1722	-0.09	-0.14	-0.03	0.002	1722	-0.24	-0.30	-0.19	<0.0001	1722	-0.25	-0.31	-0.20	<0.0001
Total cholesterol in HDL3 (mmol/l)	1722	-0.05	-0.10	0.00	0.036	1722	-0.04	-0.09	0.01	0.115	1722	-0.14	-0.19	-0.09	<0.0001	1722	-0.14	-0.19	-0.09	<0.0001
Esterified cholesterol (mmol/l)	1722	0.04	-0.01	0.09	0.117	1722	0.05	0.00	0.10	0.046	1722	0.12	0.06	0.17	<0.0001	1722	0.14	0.08	0.20	<0.0001
Free cholesterol (mmol/l)	1722	0.05	0.00	0.10	0.035	1722	0.06	0.01	0.11	0.013	1722	0.12	0.07	0.17	<0.0001	1722	0.14	0.08	0.20	<0.0001
Serum total triglycerides (mmol/l)	1722	0.13	0.08	0.19	<0.0001	1722	0.14	0.08	0.20	<0.0001	1722	0.29	0.23	0.36	<0.0001	1722	0.33	0.26	0.39	<0.0001
Triglycerides in VLDL (mmol/l)	1722	0.15	0.09	0.21	<0.0001	1722	0.16	0.10	0.21	<0.0001	1722	0.33	0.27	0.39	<0.0001	1722	0.36	0.29	0.43	<0.0001
Triglycerides in LDL (mmol/l)	1722	0.01	-0.04	0.06	0.639	1722	0.01	-0.03	0.06	0.538	1722	0.02	-0.03	0.07	0.392	1722	0.04	-0.02	0.09	0.202
Triglycerides in HDL (mmol/l)	1722	0.08	0.03	0.13	0.003	1722	0.07	0.02	0.13	0.005	1722	0.17	0.11	0.23	<0.0001	1722	0.18	0.12	0.24	<0.0001
Diacylglycerol (mmol/l)	1722	0.11	0.06	0.16	<0.0001	1722	0.10	0.05	0.16	0.0001	1722	0.24	0.18	0.29	<0.0001	1722	0.24	0.18	0.31	<0.0001
Ratio of diacylglycerol to triglycerides	1722	0.07	0.02	0.12	0.008	1722	0.07	0.02	0.12	0.011	1722	0.11	0.06	0.16	<0.0001	1722	0.11	0.05	0.16	0.0001
Total phosphoglycerides (mmol/l)	1722	0.01	-0.04	0.05	0.784	1722	0.01	-0.04	0.06	0.676	1722	0.00	-0.05	0.05	0.945	1722	0.00	-0.05	0.05	0.997
Ratio of triglycerides to phosphoglycerides	1722	0.14	0.08	0.20	<0.0001	1722	0.14	0.08	0.20	<0.0001	1722	0.31	0.24	0.37	<0.0001	1722	0.33	0.26	0.40	<0.0001
Phosphatidylcholine and other cholines (mmol/l)	1722	0.00	-0.05	0.04	0.873	1722	0.00	-0.05	0.05	0.961	1722	-0.01	-0.06	0.04	0.614	1722	-0.02	-0.07	0.04	0.511
Total cholines (mmol/l)	1722	-0.01	-0.05	0.04	0.779	1722	0.00	-0.05	0.05	0.948	1722	-0.01	-0.06	0.04	0.798	1722	0.00	-0.06	0.05	0.885
Apolipoprotein A-I (g/l)	1722	-0.04	-0.09	0.01	0.122	1722	-0.02	-0.07	0.03	0.341	1722	-0.10	-0.15	-0.05	<0.0001	1722	-0.09	-0.14	-0.04	0.0004
Apolipoprotein B (g/l)	1722	0.12	0.06	0.17	<0.0001	1722	0.12	0.07	0.18	<0.0001	1722	0.29	0.23	0.35	<0.0001	1722	0.33	0.26	0.39	<0.0001
Ratio of apolipoprotein B to apolipoprotein A-I	1722	0.14	0.08	0.20	<0.0001	1722	0.14	0.08	0.20	<0.0001	1722	0.34	0.28	0.41	<0.0001	1722	0.37	0.31	0.44	<0.0001
Total fatty acids (mmol/l)	1722	0.07	0.02	0.12	0.003	1722	0.08	0.03	0.13	0.001	1722	0.17	0.12	0.23	<0.0001	1722	0.20	0.14	0.26	<0.0001
Estimated description of fatty acid chain length, not actual carbon number	1722	0.01	-0.03	0.06	0.544	1722	0.01	-0.04	0.06	0.649	1722	0.01	-0.04	0.06	0.743	1722	0.02	-0.04	0.08	0.556
Estimated degree of unsaturation	1722	-0.03	-0.08	0.02	0.211	1722	-0.03	-0.08	0.02	0.260	1722	-0.06	-0.12	-0.01	0.016	1722	-0.07	-0.12	-0.01	0.019
22:6, docosahexaenoic acid (mmol/l)	1722	0.01	-0.04	0.05	0.692	1722	0.01	-0.04	0.05	0.749	1722	0.05	0.00	0.09	0.065	1722	0.05	0.00	0.10	0.059
18:2, linoleic acid (mmol/l)	1722	0.02	-0.03	0.07	0.359	1722	0.03	-0.02	0.08	0.214	1722	0.09	0.03	0.14	0.001	1722	0.10	0.04	0.16	0.001
Conjugated linoleic acid (mmol/l)	1722	0.06	0.02	0.10	0.006	1722	0.07	0.02	0.11	0.002	1722	0.11	0.06	0.16	<0.0001	1722	0.14	0.09	0.20	<0.0001
Omega-3 fatty acids (mmol/l)	1722	0.04	-0.01	0.09	0.118	1722	0.05	0.00	0.10	0.064	1722	0.12	0.07	0.17	<0.0001	1722	0.14	0.08	0.19	<0.0001
Omega-6 fatty acids (mmol/l)	1722	0.04	-0.01	0.08	0.138	1722	0.05	0.00	0.09	0.066	1722	0.11	0.06	0.16	<0.0001	1722	0.12	0.07	0.18	<0.0001
Polyunsaturated fatty acids (mmol/l)	1722	0.04	-0.01	0.09	0.119	1722	0.05	0.00	0.10	0.054	1722	0.11	0.06	0.17	<0.0001	1722	0.13	0.07	0.19	<0.0001
Monounsaturated fatty acids; 16:1, 18:1 (mmol/l)	1722	0.10	0.05	0.15	0.0002	1722	0.10	0.05	0.16	<0.0001	1722	0.21	0.15	0.26	<0.0001	1722	0.25	0.19	0.31	<0.0001
Saturated fatty acids (mmol/l)	1722	0.07	0.02	0.12	0.006	1722	0.08	0.03	0.13	0.003	1722	0.15	0.10	0.21	<0.0001	1722	0.17	0.11	0.23	<0.0001
Ratio of 22:6 docosahexaenoic acid to total fatty acids (%)	1722	-0.02	-0.07	0.02	0.361	1722	-0.03	-0.07	0.02	0.265	1722	-0.04	-0.09	0.01	0.110	1722	-0.05	-0.10	0.01	0.081
Ratio of 18:2 linoleic acid to total fatty acids (%)	1722	-0.10	-0.15	-0.05	0.0001	1722	-0.10	-0.16	-0.05	0.0001	1722	-0.16	-0.21	-0.10	<0.0001	1722	-0.19	-0.25	-0.13	<0.0001
Ratio of conjugated linoleic acid to total fatty acids (%)	1722	0.05	0.01	0.09	0.018	1722	0.05	0.01	0.10	0.009	1722	0.08	0.04	0.13	0.0004	1722	0.11	0.06	0.16	<0.0001
Ratio of omega-3 fatty acids to total fatty acids (%)	1722	-0.01	-0.06	0.04	0.581	1722	-0.01	-0.06	0.04	0.702	1722	0.00	-0.05	0.05	0.926	1722	0.00	-0.06	0.05	0.880
Ratio of omega-6 fatty acids to total fatty acids (%)	1722	-0.10	-0.15	-0.04	0.0003	1722	-0.10	-0.15	-0.04	0.0003	1722	-0.15	-0.21	-0.10	<0.0001	1722	-0.18	-0.24	-0.12	<0.0001
Ratio of polyunsaturated fatty acids to total fatty acids (%)	1722	-0.09	-0.15	-0.04	0.0004	1722	-0.10	-0.15	-0.04	0.0005	1722	-0.15	-0.20	-0.09	<0.0001	1722	-0.17	-0.23	-0.11	<0.0001
Ratio of monounsaturated fatty acids to total fatty acids (%)	1722	0.09	0.04	0.14	0.0004	1722	0.10	0.05	0.15	0.0002	1722	0.16	0.11	0.22	<0.0001	1722	0.21	0.15	0.27	<0.0001
Ratio of saturated fatty acids to total fatty acids (%)	1722	-0.01	-0.06	0.04	0.664	1722	-0.02	-0.07	0.03	0.445	1722	-0.04	-0.09	0.01	0.131	1722	-0.08	-0.14	-0.02	0.006
Insulin (mu/l)	1722	0.16	0.10	0.22	<0.0001	1722	0.16	0.11	0.21	<0.0001	1722	0.33	0.21	0.46	<0.0001	1722	0.36	0.23	0.48	<0.0001
Glucose (mmol/l)	1722	0.03	0.00	0.07	0.083	1722	0.03	-0.01	0.07	0.105	1722	0.10	0.07	0.14	<0.0001	1722	0.11	0.07	0.15	<0.0001
Lactate (mmol/l)	1722	0.03	-0.02	0.08	0.290	1722	0.05	0.00	0.10	0.074	1722	0.02	-0.03	0.07	0.542	1722	0.04	-0.01	0.10	0.147
Pyruvate (mmol/l)	1722	0.10	0.05	0.15	0.0002	1722	0.11	0.06	0.17	<0.0001	1722	0.12	0.07	0.17	<0.0001	1722	0.15	0.09	0.21	<0.0001
Citrate (mmol/l)	1722	-0.13	-0.18	-0.07	<0.0001	1722	-0.13	-0.18	-0.08	<0.0001	1722	-0.14	-0.19	-0.09	<0.0001	1722	-0.14	-0.20	-0.08	<0.0001
Alanine (mmol/l)	1722	0.05	0.01	0.10	0.028	1722	0.05	0.00	0.11	0.034	1722	0.11	0.06	0.16	<0.0001	1722	0.11	0.05	0.17	0.0003
Glutamine (mmol/l)	1722	-0.04	-0.08	0.01	0.114	1722	-0.04	-0.09	0.01	0.109	1722	-0.03	-0.08	0.02	0.298	1722	-0.02	-0.08	0.03	0.406
Histidine (mmol/l)	1722	0.02	-0.03	0.07	0.509	1722	0.02	-0.03	0.07	0.479	1722	0.05	-0.01	0.10	0.076	1722	0.04	-0.02	0.10	0.183
Isoleucine (mmol/l)	1722	0.12	0.07	0.17	<0.0001	1722	0.11	0.06	0.16	<0.0001	1722	0.24	0.19	0.29	<0.0001	1722	0.24	0.18	0.29	<0.0001
Leucine (mmol/l)	1722	0.09	0.04	0.13	0.0002	1722	0.07	0.03	0.12	0.002	1722	0.18	0.13	0.22	<0.0001	1722	0.15	0.10	0.20	<0.0001
Valine (mmol/l)	1722	0.14	0.09	0.19	<0.0001	1722	0.13	0.08	0.18	<0.0001	1722	0.24	0.19	0.29	<0.0001	1722	0.22	0.17	0.28	<0.0001
Phenylalanine (mmol/l)	1722	0.11	0.06	0.16	<0.0001	1722	0.11	0.05	0.16	<0.0001	1722	0.20	0.15	0.25	<0.0001	1722	0.20	0.14	0.26	<0.0001

Online Table 2 Associations of body mass index (BMI) and fat mass index at age 10y and 18y with cardiometabolic traits at age 18y in ALSPAC

	**At age 10y**										**At age 18y**									
	**BMI (per 2.7 kg/m^2 higher)**					**Fat mass index (per 2.3 kg/m^2 higher)**					**BMI (per 4.0 kg/m^2 higher)**					**Fat mass index (per 3.7 kg/m^2 higher)**				
	Adj. for age, sex, ethnicity, maternal education					*Adj. for age, sex, ethnicity, maternal education*					*Adj. for age, sex, ethnicity, maternal education, smoking, alcohol, puberty timing*					*Adj. for age, sex, ethnicity, maternal education, smoking, alcohol, puberty timing*				
**Standardized outcome at age 18y**	**N**	**Beta**	**LCL**	**UCL**	**P-value**	**N**	**Beta**	**LCL**	**UCL**	**P-value**	**N**	**Beta**	**LCL**	**UCL**	**P-value**	**N**	**Beta**	**LCL**	**UCL**	**P-value**
Tyrosine (mmol/l)	1722	0.13	0.08	0.18	<0.0001	1722	0.14	0.08	0.19	<0.0001	1722	0.28	0.22	0.33	<0.0001	1722	0.30	0.24	0.36	<0.0001
Acetate (mmol/l)	1722	-0.06	-0.08	-0.03	<0.0001	1722	-0.05	-0.07	-0.03	<0.0001	1722	-0.06	-0.09	-0.03	<0.0001	1722	-0.06	-0.08	-0.03	<0.0001
Acetoacetate (mmol/l)	1722	-0.01	-0.06	0.04	0.769	1722	0.02	-0.04	0.07	0.543	1722	-0.08	-0.13	-0.03	0.001	1722	-0.06	-0.11	-0.01	0.014
3-hydroxybutyrate (mmol/l)	1722	-0.03	-0.07	0.02	0.316	1722	0.00	-0.05	0.05	0.993	1722	-0.11	-0.16	-0.05	0.0001	1722	-0.08	-0.13	-0.02	0.005
Creatinine (mmol/l)	1722	0.05	0.01	0.09	0.013	1722	0.01	-0.03	0.05	0.602	1722	0.07	0.03	0.11	0.002	1722	-0.01	-0.06	0.04	0.743
Albumin (signal area)	1722	-0.05	-0.10	0.00	0.039	1722	-0.07	-0.12	-0.02	0.006	1722	-0.07	-0.12	-0.01	0.013	1722	-0.08	-0.14	-0.03	0.003
Glycoprotein acetyls, mainly a1-acid glycoprotein (mmol/l)	1722	0.15	0.10	0.21	<0.0001	1722	0.16	0.11	0.22	<0.0001	1722	0.30	0.25	0.35	<0.0001	1722	0.35	0.29	0.40	<0.0001
C-reactive protein (mg/l)	1722	0.06	0.01	0.10	0.013	1722	0.06	0.01	0.10	0.022	1722	0.08	0.02	0.13	0.007	1722	0.09	0.03	0.16	0.006

Online Table 3 Associations of regional fat indexes at age 10y with cardiometabolic traits at age 18y in ALSPAC

	At age 10y															
	**Trunk fat index (per 1.1 kg/m^2 higher)**					**Arm fat index (per 0.2 kg/m^2 higher)**					**Leg fat index (per 0.9 kg/m^2 higher)**					
	Adj. for age, sex, ethnicity, maternal education, arm fat index, leg fat index					*Adj. for age, sex, ethnicity, maternal education, trunk fat index, leg fat index*					*Adj. for age, sex, ethnicity, maternal education, trunk fat index, arm fat index*					***P-value for regional heterogeneity***
**Standardized outcome at age 18y**	**N**	**Beta**	**LCL**	**UCL**	**P-value**	**N**	**Beta**	**LCL**	**UCL**	**P-value**	**N**	**Beta**	**LCL**	**UCL**	**P-value**	
Systolic blood pressure (mmHg)	3548	0.12	0.01	0.23	0.039	3548	-0.01	-0.15	0.12	0.843	3548	0.07	-0.03	0.18	0.186	0.515
Diastolic blood pressure (mmHg)	3548	0.13	0.01	0.25	0.033	3548	0.06	-0.09	0.21	0.447	3548	0.05	-0.07	0.17	0.447	0.654
Concentration of chylomicrons and extremely large VLDL particles (mol/l)	2440	0.37	0.20	0.54	<0.0001	2440	0.13	-0.10	0.35	0.283	2440	-0.33	-0.49	-0.16	<0.0001	<0.0001
Total lipids in chylomicrons and extremely large VLDL (mmol/l)	2440	0.37	0.20	0.55	<0.0001	2440	0.12	-0.11	0.35	0.307	2440	-0.33	-0.49	-0.16	<0.0001	<0.0001
Phospholipids in chylomicrons and extremely large VLDL (mmol/l)	2440	0.37	0.19	0.54	<0.0001	2440	0.12	-0.11	0.35	0.312	2440	-0.32	-0.49	-0.16	0.0001	<0.0001
Total cholesterol in chylomicrons and extremely large VLDL (mmol/l)	2440	0.37	0.20	0.54	<0.0001	2440	0.10	-0.13	0.33	0.408	2440	-0.31	-0.48	-0.14	0.0003	<0.0001
Cholesterol esters in chylomicrons and extremely large VLDL (mmol/l)	2440	0.36	0.19	0.54	<0.0001	2440	0.08	-0.15	0.30	0.511	2440	-0.29	-0.46	-0.12	0.001	<0.0001
Free cholesterol in chylomicrons and extremely large VLDL (mmol/l)	2440	0.36	0.19	0.53	<0.0001	2440	0.12	-0.11	0.35	0.310	2440	-0.32	-0.49	-0.15	0.0002	<0.0001
Triglycerides in chylomicrons and extremely large VLDL (mmol/l)	2440	0.37	0.20	0.55	<0.0001	2440	0.12	-0.10	0.35	0.285	2440	-0.33	-0.50	-0.17	<0.0001	<0.0001
Concentration of very large VLDL particles (mol/l)	2440	0.38	0.21	0.55	<0.0001	2440	0.12	-0.11	0.36	0.295	2440	-0.34	-0.51	-0.17	<0.0001	<0.0001
Total lipids in very large VLDL (mmol/l)	2440	0.38	0.21	0.55	<0.0001	2440	0.12	-0.11	0.35	0.322	2440	-0.34	-0.51	-0.17	<0.0001	<0.0001
Phospholipids in very large VLDL (mmol/l)	2440	0.37	0.20	0.54	<0.0001	2440	0.11	-0.12	0.34	0.359	2440	-0.32	-0.49	-0.15	0.0002	<0.0001
Total cholesterol in very large VLDL (mmol/l)	2440	0.38	0.21	0.55	<0.0001	2440	0.11	-0.12	0.35	0.330	2440	-0.33	-0.50	-0.16	0.0001	<0.0001
Cholesterol esters in very large VLDL (mmol/l)	2440	0.39	0.22	0.56	<0.0001	2440	0.12	-0.11	0.35	0.317	2440	-0.34	-0.51	-0.17	<0.0001	<0.0001
Free cholesterol in very large VLDL (mmol/l)	2440	0.37	0.19	0.54	<0.0001	2440	0.11	-0.12	0.34	0.350	2440	-0.32	-0.48	-0.15	0.0002	<0.0001
Triglycerides in very large VLDL (mmol/l)	2440	0.38	0.21	0.55	<0.0001	2440	0.12	-0.11	0.35	0.313	2440	-0.34	-0.51	-0.18	<0.0001	<0.0001
Concentration of large VLDL particles (mol/l)	2440	0.39	0.22	0.56	<0.0001	2440	0.12	-0.11	0.35	0.298	2440	-0.35	-0.52	-0.18	<0.0001	<0.0001
Total lipids in large VLDL (mmol/l)	2440	0.39	0.22	0.56	<0.0001	2440	0.12	-0.11	0.35	0.312	2440	-0.35	-0.52	-0.18	<0.0001	<0.0001
Phospholipids in large VLDL (mmol/l)	2440	0.38	0.21	0.55	<0.0001	2440	0.12	-0.11	0.35	0.324	2440	-0.34	-0.51	-0.17	<0.0001	<0.0001
Total cholesterol in large VLDL (mmol/l)	2440	0.38	0.21	0.55	<0.0001	2440	0.11	-0.12	0.34	0.348	2440	-0.33	-0.50	-0.16	0.0001	<0.0001
Cholesterol esters in large VLDL (mmol/l)	2440	0.38	0.21	0.55	<0.0001	2440	0.11	-0.12	0.34	0.356	2440	-0.33	-0.50	-0.16	0.0002	<0.0001
Free cholesterol in large VLDL (mmol/l)	2440	0.37	0.20	0.54	<0.0001	2440	0.11	-0.12	0.34	0.344	2440	-0.33	-0.50	-0.16	0.0001	<0.0001
Triglycerides in large VLDL (mmol/l)	2440	0.39	0.22	0.56	<0.0001	2440	0.12	-0.11	0.35	0.296	2440	-0.36	-0.53	-0.19	<0.0001	<0.0001
Concentration of medium VLDL particles (mol/l)	2440	0.39	0.22	0.56	<0.0001	2440	0.14	-0.09	0.37	0.248	2440	-0.37	-0.54	-0.20	<0.0001	<0.0001
Total lipids in medium VLDL (mmol/l)	2440	0.39	0.22	0.56	<0.0001	2440	0.13	-0.10	0.36	0.260	2440	-0.36	-0.53	-0.20	<0.0001	<0.0001
Phospholipids in medium VLDL (mmol/l)	2440	0.37	0.20	0.54	<0.0001	2440	0.13	-0.10	0.36	0.258	2440	-0.35	-0.52	-0.18	<0.0001	<0.0001
Total cholesterol in medium VLDL (mmol/l)	2440	0.34	0.17	0.51	<0.0001	2440	0.12	-0.10	0.35	0.286	2440	-0.32	-0.48	-0.15	0.0002	<0.0001
Cholesterol esters in medium VLDL (mmol/l)	2440	0.31	0.14	0.48	0.0004	2440	0.11	-0.11	0.33	0.321	2440	-0.28	-0.45	-0.12	0.001	<0.0001
Free cholesterol in medium VLDL (mmol/l)	2440	0.36	0.19	0.53	<0.0001	2440	0.13	-0.10	0.36	0.265	2440	-0.34	-0.51	-0.17	<0.0001	<0.0001
Triglycerides in medium VLDL (mmol/l)	2440	0.41	0.24	0.58	<0.0001	2440	0.13	-0.10	0.36	0.257	2440	-0.38	-0.55	-0.21	<0.0001	<0.0001
Concentration of small VLDL particles (mol/l)	2440	0.33	0.16	0.49	0.0001	2440	0.15	-0.07	0.37	0.181	2440	-0.34	-0.50	-0.18	<0.0001	<0.0001
Total lipids in small VLDL (mmol/l)	2440	0.32	0.15	0.48	0.0002	2440	0.15	-0.06	0.37	0.169	2440	-0.33	-0.49	-0.17	<0.0001	<0.0001
Phospholipids in small VLDL (mmol/l)	2440	0.29	0.12	0.45	0.001	2440	0.14	-0.07	0.35	0.195	2440	-0.30	-0.46	-0.14	0.0002	<0.0001
Total cholesterol in small VLDL (mmol/l)	2440	0.27	0.10	0.44	0.002	2440	0.14	-0.08	0.35	0.214	2440	-0.28	-0.44	-0.13	0.0004	<0.0001
Cholesterol esters in small VLDL (mmol/l)	2440	0.26	0.09	0.43	0.003	2440	0.13	-0.09	0.35	0.248	2440	-0.27	-0.42	-0.11	0.001	<0.0001
Free cholesterol in small VLDL (mmol/l)	2440	0.26	0.10	0.43	0.001	2440	0.14	-0.07	0.35	0.198	2440	-0.29	-0.44	-0.13	0.0004	<0.0001
Triglycerides in small VLDL (mmol/l)	2440	0.33	0.16	0.50	<0.0001	2440	0.15	-0.07	0.37	0.185	2440	-0.35	-0.51	-0.18	<0.0001	<0.0001
Concentration of very small VLDL particles (mol/l)	2440	0.07	-0.09	0.24	0.369	2440	0.10	-0.10	0.31	0.327	2440	-0.11	-0.27	0.04	0.154	0.217
Total lipids in very small VLDL (mmol/l)	2440	0.13	-0.04	0.30	0.138	2440	0.11	-0.10	0.33	0.304	2440	-0.16	-0.32	0.00	0.048	0.045
Phospholipids in very small VLDL (mmol/l)	2440	0.03	-0.14	0.20	0.721	2440	0.07	-0.14	0.28	0.498	2440	-0.05	-0.21	0.11	0.525	0.692
Total cholesterol in very small VLDL (mmol/l)	2440	0.16	-0.02	0.33	0.081	2440	0.09	-0.13	0.31	0.409	2440	-0.17	-0.32	-0.01	0.040	0.028
Cholesterol esters in very small VLDL (mmol/l)	2440	0.19	0.02	0.37	0.033	2440	0.11	-0.11	0.33	0.338	2440	-0.20	-0.36	-0.05	0.011	0.004
Free cholesterol in very small VLDL (mmol/l)	2440	0.05	-0.12	0.23	0.538	2440	0.05	-0.16	0.27	0.644	2440	-0.06	-0.22	0.10	0.436	0.604
Triglycerides in very small VLDL (mmol/l)	2440	0.13	-0.03	0.29	0.106	2440	0.15	-0.05	0.36	0.151	2440	-0.21	-0.36	-0.05	0.009	0.011
Concentration of IDL particles (mol/l)	2440	-0.02	-0.18	0.15	0.831	2440	0.06	-0.15	0.26	0.598	2440	-0.01	-0.16	0.15	0.948	0.912
Total lipids in IDL (mmol/l)	2440	0.00	-0.17	0.16	0.967	2440	0.05	-0.16	0.26	0.644	2440	0.00	-0.16	0.15	0.964	0.947
Phospholipids in IDL (mmol/l)	2440	-0.04	-0.21	0.12	0.616	2440	0.04	-0.17	0.24	0.741	2440	0.04	-0.12	0.19	0.644	0.796
Total cholesterol in IDL (mmol/l)	2440	0.03	-0.14	0.20	0.744	2440	0.04	-0.17	0.26	0.686	2440	-0.02	-0.18	0.14	0.803	0.894
Cholesterol esters in IDL (mmol/l)	2440	0.07	-0.11	0.24	0.452	2440	0.05	-0.16	0.27	0.623	2440	-0.05	-0.21	0.10	0.498	0.588
Free cholesterol in IDL (mmol/l)	2440	-0.06	-0.23	0.10	0.459	2440	0.02	-0.19	0.23	0.864	2440	0.06	-0.09	0.22	0.430	0.593

Online Table 3 Associations of regional fat indexes at age 10y with cardiometabolic traits at age 18y in ALSPAC

	At age 10y															
	**Trunk fat index (per 1.1 kg/m^2 higher)**					**Arm fat index (per 0.2 kg/m^2 higher)**					**Leg fat index (per 0.9 kg/m^2 higher)**					
	Adj. for age, sex, ethnicity, maternal education, arm fat index, leg fat index					*Adj. for age, sex, ethnicity, maternal education, trunk fat index, leg fat index*					*Adj. for age, sex, ethnicity, maternal education, trunk fat index, arm fat index*					***P-value for regional heterogeneity***
**Standardized outcome at age 18y**	**N**	**Beta**	**LCL**	**UCL**	**P-value**	**N**	**Beta**	**LCL**	**UCL**	**P-value**	**N**	**Beta**	**LCL**	**UCL**	**P-value**	
Triglycerides in IDL (mmol/l)	2440	-0.10	-0.25	0.05	0.198	2440	0.09	-0.10	0.28	0.360	2440	0.01	-0.14	0.15	0.937	0.434
Concentration of large LDL particles (mol/l)	2440	-0.02	-0.18	0.15	0.851	2440	0.04	-0.16	0.25	0.680	2440	0.02	-0.14	0.17	0.847	0.936
Total lipids in large LDL (mmol/l)	2440	-0.01	-0.18	0.15	0.867	2440	0.04	-0.16	0.25	0.681	2440	0.02	-0.14	0.17	0.836	0.939
Phospholipids in large LDL (mmol/l)	2440	0.01	-0.15	0.18	0.904	2440	0.04	-0.16	0.25	0.688	2440	0.00	-0.15	0.16	0.963	0.973
Total cholesterol in large LDL (mmol/l)	2440	0.00	-0.17	0.16	0.962	2440	0.04	-0.17	0.25	0.713	2440	0.01	-0.14	0.17	0.861	0.969
Cholesterol esters in large LDL (mmol/l)	2440	0.01	-0.15	0.18	0.884	2440	0.04	-0.17	0.25	0.686	2440	0.00	-0.15	0.15	1.000	0.967
Free cholesterol in large LDL (mmol/l)	2440	-0.05	-0.22	0.11	0.519	2440	0.03	-0.18	0.23	0.805	2440	0.06	-0.10	0.21	0.474	0.657
Triglycerides in large LDL (mmol/l)	2440	-0.13	-0.28	0.02	0.096	2440	0.06	-0.13	0.26	0.529	2440	0.06	-0.09	0.20	0.456	0.231
Concentration of medium LDL particles (mol/l)	2440	0.01	-0.15	0.18	0.895	2440	0.06	-0.15	0.26	0.602	2440	-0.01	-0.17	0.14	0.874	0.918
Total lipids in medium LDL (mmol/l)	2440	0.01	-0.16	0.17	0.925	2440	0.05	-0.16	0.26	0.645	2440	0.00	-0.16	0.15	0.970	0.953
Phospholipids in medium LDL (mmol/l)	2440	0.05	-0.11	0.21	0.539	2440	0.05	-0.16	0.26	0.632	2440	-0.02	-0.18	0.13	0.759	0.792
Total cholesterol in medium LDL (mmol/l)	2440	0.01	-0.15	0.18	0.874	2440	0.05	-0.16	0.26	0.664	2440	-0.01	-0.16	0.15	0.935	0.950
Cholesterol esters in medium LDL (mmol/l)	2440	0.02	-0.15	0.19	0.835	2440	0.05	-0.16	0.26	0.648	2440	-0.01	-0.17	0.14	0.870	0.925
Free cholesterol in medium LDL (mmol/l)	2440	-0.01	-0.17	0.16	0.935	2440	0.04	-0.17	0.25	0.717	2440	0.02	-0.13	0.17	0.803	0.959
Triglycerides in medium LDL (mmol/l)	2440	-0.13	-0.28	0.03	0.110	2440	0.04	-0.15	0.24	0.651	2440	0.07	-0.07	0.21	0.326	0.217
Concentration of small LDL particles (mol/l)	2440	0.03	-0.14	0.20	0.731	2440	0.04	-0.17	0.25	0.705	2440	-0.01	-0.16	0.14	0.888	0.924
Total lipids in small LDL (mmol/l)	2440	0.03	-0.14	0.19	0.734	2440	0.04	-0.17	0.25	0.712	2440	-0.01	-0.16	0.14	0.907	0.932
Phospholipids in small LDL (mmol/l)	2440	0.07	-0.09	0.23	0.407	2440	0.02	-0.19	0.22	0.873	2440	-0.01	-0.16	0.14	0.873	0.793
Total cholesterol in small LDL (mmol/l)	2440	0.02	-0.15	0.18	0.839	2440	0.04	-0.17	0.25	0.717	2440	0.00	-0.16	0.15	0.978	0.967
Cholesterol esters in small LDL (mmol/l)	2440	0.01	-0.16	0.18	0.880	2440	0.04	-0.17	0.25	0.692	2440	0.00	-0.16	0.15	0.974	0.964
Free cholesterol in small LDL (mmol/l)	2440	0.04	-0.13	0.20	0.677	2440	0.02	-0.19	0.23	0.836	2440	0.00	-0.15	0.15	0.996	0.956
Triglycerides in small LDL (mmol/l)	2440	0.01	-0.15	0.18	0.859	2440	0.08	-0.12	0.28	0.436	2440	-0.05	-0.20	0.10	0.486	0.676
Concentration of very large HDL particles (mol/l)	2440	-0.31	-0.46	-0.16	<0.0001	2440	-0.20	-0.38	-0.01	0.035	2440	0.41	0.27	0.56	<0.0001	<0.0001
Total lipids in very large HDL (mmol/l)	2440	-0.29	-0.44	-0.14	0.0002	2440	-0.20	-0.38	-0.02	0.034	2440	0.40	0.25	0.54	<0.0001	<0.0001
Phospholipids in very large HDL (mmol/l)	2440	-0.36	-0.50	-0.21	<0.0001	2440	-0.20	-0.38	-0.01	0.034	2440	0.44	0.30	0.59	<0.0001	<0.0001
Total cholesterol in very large HDL (mmol/l)	2440	-0.19	-0.35	-0.03	0.017	2440	-0.19	-0.38	0.00	0.045	2440	0.32	0.18	0.47	<0.0001	<0.0001
Cholesterol esters in very large HDL (mmol/l)	2440	-0.15	-0.31	0.01	0.059	2440	-0.18	-0.37	0.01	0.062	2440	0.28	0.13	0.43	0.0002	0.0002
Free cholesterol in very large HDL (mmol/l)	2440	-0.29	-0.44	-0.14	0.0002	2440	-0.21	-0.39	-0.02	0.030	2440	0.41	0.26	0.55	<0.0001	<0.0001
Triglycerides in very large HDL (mmol/l)	2440	-0.06	-0.23	0.10	0.430	2440	0.01	-0.20	0.22	0.922	2440	0.04	-0.12	0.20	0.603	0.690
Concentration of large HDL particles (mol/l)	2440	-0.34	-0.49	-0.18	<0.0001	2440	-0.21	-0.41	-0.02	0.031	2440	0.44	0.30	0.58	<0.0001	<0.0001
Total lipids in large HDL (mmol/l)	2440	-0.34	-0.50	-0.19	<0.0001	2440	-0.21	-0.41	-0.02	0.029	2440	0.45	0.31	0.59	<0.0001	<0.0001
Phospholipids in large HDL (mmol/l)	2440	-0.32	-0.47	-0.18	<0.0001	2440	-0.20	-0.39	-0.01	0.039	2440	0.43	0.29	0.57	<0.0001	<0.0001
Total cholesterol in large HDL (mmol/l)	2440	-0.36	-0.51	-0.21	<0.0001	2440	-0.23	-0.42	-0.03	0.022	2440	0.47	0.32	0.61	<0.0001	<0.0001
Cholesterol esters in large HDL (mmol/l)	2440	-0.36	-0.51	-0.21	<0.0001	2440	-0.23	-0.42	-0.03	0.022	2440	0.46	0.32	0.61	<0.0001	<0.0001
Free cholesterol in large HDL (mmol/l)	2440	-0.36	-0.51	-0.21	<0.0001	2440	-0.22	-0.41	-0.03	0.023	2440	0.46	0.32	0.61	<0.0001	<0.0001
Triglycerides in large HDL (mmol/l)	2440	-0.04	-0.20	0.11	0.592	2440	-0.09	-0.28	0.10	0.364	2440	0.12	-0.03	0.27	0.116	0.266
Concentration of medium HDL particles (mol/l)	2440	-0.08	-0.25	0.09	0.349	2440	-0.14	-0.33	0.06	0.183	2440	0.22	0.07	0.37	0.005	0.020
Total lipids in medium HDL (mmol/l)	2440	-0.10	-0.27	0.07	0.251	2440	-0.15	-0.35	0.05	0.133	2440	0.24	0.09	0.40	0.002	0.006
Phospholipids in medium HDL (mmol/l)	2440	-0.11	-0.28	0.06	0.186	2440	-0.14	-0.34	0.06	0.172	2440	0.24	0.09	0.39	0.002	0.006
Total cholesterol in medium HDL (mmol/l)	2440	-0.12	-0.28	0.05	0.170	2440	-0.18	-0.38	0.02	0.077	2440	0.28	0.13	0.43	0.0003	0.001
Cholesterol esters in medium HDL (mmol/l)	2440	-0.12	-0.28	0.05	0.160	2440	-0.18	-0.38	0.01	0.069	2440	0.29	0.13	0.44	0.0003	0.001
Free cholesterol in medium HDL (mmol/l)	2440	-0.10	-0.26	0.07	0.247	2440	-0.15	-0.34	0.04	0.129	2440	0.25	0.10	0.39	0.001	0.005
Triglycerides in medium HDL (mmol/l)	2440	0.24	0.07	0.41	0.005	2440	0.09	-0.11	0.29	0.395	2440	-0.22	-0.38	-0.06	0.007	0.002
Concentration of small HDL particles (mol/l)	2440	0.09	-0.08	0.27	0.293	2440	-0.03	-0.23	0.17	0.764	2440	0.00	-0.16	0.16	0.978	0.706
Total lipids in small HDL (mmol/l)	2440	0.00	-0.17	0.17	0.997	2440	-0.06	-0.26	0.14	0.570	2440	0.09	-0.07	0.24	0.274	0.622
Phospholipids in small HDL (mmol/l)	2440	0.08	-0.10	0.26	0.392	2440	-0.03	-0.24	0.17	0.743	2440	0.00	-0.16	0.16	0.953	0.790
Total cholesterol in small HDL (mmol/l)	2440	-0.12	-0.27	0.04	0.139	2440	-0.10	-0.29	0.09	0.323	2440	0.20	0.06	0.34	0.006	0.014
Cholesterol esters in small HDL (mmol/l)	2440	-0.12	-0.27	0.02	0.093	2440	-0.08	-0.27	0.10	0.387	2440	0.19	0.06	0.33	0.006	0.010
Free cholesterol in small HDL (mmol/l)	2440	-0.04	-0.23	0.14	0.631	2440	-0.11	-0.32	0.10	0.285	2440	0.15	-0.01	0.31	0.071	0.183
Triglycerides in small HDL (mmol/l)	2440	0.19	0.02	0.35	0.026	2440	0.16	-0.05	0.36	0.136	2440	-0.26	-0.42	-0.10	0.001	0.001

Online Table 3 Associations of regional fat indexes at age 10y with cardiometabolic traits at age 18y in ALSPAC

	At age 10y															
	**Trunk fat index (per 1.1 kg/m^2 higher)** *Adj. for age, sex, ethnicity, maternal education, arm fat index, leg fat index*					**Arm fat index (per 0.2 kg/m^2 higher)** *Adj. for age, sex, ethnicity, maternal education, trunk fat index, leg fat index*					**Leg fat index (per 0.9 kg/m^2 higher)** *Adj. for age, sex, ethnicity, maternal education, trunk fat index, arm fat index*					***P-value for regional heterogeneity***
**Standardized outcome at age 18y**	**N**	**Beta**	**LCL**	**UCL**	**P-value**	**N**	**Beta**	**LCL**	**UCL**	**P-value**	**N**	**Beta**	**LCL**	**UCL**	**P-value**	
Phospholipids to total lipids ratio in chylomicrons and extremely large VLDL (%)	2440	0.02	-0.06	0.09	0.620	2440	-0.10	-0.27	0.08	0.281	2440	0.12	-0.02	0.26	0.103	0.369
Total cholesterol to total lipids ratio in chylomicrons and extremely large VLDL (%)	2440	0.08	-0.07	0.23	0.288	2440	0.02	-0.17	0.20	0.871	2440	-0.07	-0.23	0.09	0.372	0.460
Cholesterol esters to total lipids ratio in chylomicrons and extremely large VLDL (%)	2440	0.07	-0.07	0.21	0.327	2440	-0.01	-0.20	0.17	0.875	2440	-0.06	-0.22	0.09	0.422	0.511
Free cholesterol to total lipids ratio in chylomicrons and extremely large VLDL (%)	2440	0.08	-0.07	0.23	0.285	2440	0.12	-0.07	0.31	0.217	2440	-0.09	-0.23	0.06	0.234	0.208
Triglycerides to total lipids ratio in chylomicrons and extremely large VLDL (%)	2440	-0.12	-0.27	0.03	0.125	2440	0.05	-0.13	0.23	0.576	2440	0.03	-0.12	0.18	0.691	0.398
Phospholipids to total lipds ratio in very large VLDL (%)	2440	0.18	0.04	0.32	0.014	2440	0.00	-0.20	0.20	0.996	2440	-0.06	-0.21	0.10	0.470	0.097
Total cholesterol to total lipids ratio in very large VLDL (%)	2440	-0.20	-0.42	0.02	0.071	2440	-0.02	-0.23	0.19	0.848	2440	0.18	0.02	0.33	0.023	0.033
Cholesterol esters to total lipids ratio in very large VLDL (%)	2440	-0.04	-0.32	0.24	0.779	2440	-0.23	-0.74	0.28	0.372	2440	0.24	-0.07	0.55	0.132	0.056
Free cholesterol to total lipids ratio in very large VLDL (%)	2440	-0.01	-0.27	0.24	0.921	2440	-0.26	-0.76	0.24	0.305	2440	0.30	-0.01	0.61	0.060	0.003
Triglycerides to total lipids ratio in very large VLDL (%)	2440	0.13	-0.02	0.29	0.083	2440	-0.01	-0.21	0.18	0.898	2440	-0.10	-0.24	0.04	0.162	0.106
Phospholipids to total lipds ratio in large VLDL (%)	2440	0.19	0.04	0.34	0.011	2440	0.10	-0.09	0.29	0.285	2440	-0.21	-0.35	-0.06	0.004	0.001
Total cholesterol to total lipids ratio in large VLDL (%)	2440	0.05	-0.11	0.21	0.532	2440	0.13	-0.07	0.33	0.199	2440	-0.12	-0.27	0.02	0.100	0.159
Cholesterol esters to total lipids ratio in large VLDL (%)	2440	-0.12	-0.27	0.03	0.108	2440	0.11	-0.07	0.29	0.245	2440	0.02	-0.12	0.16	0.793	0.262
Free cholesterol to total lipids ratio in large VLDL (%)	2440	0.11	0.04	0.18	0.003	2440	0.02	-0.07	0.10	0.657	2440	-0.08	-0.15	-0.02	0.017	0.003
Triglycerides to total lipids ratio in large VLDL (%)	2440	-0.14	-0.31	0.03	0.100	2440	-0.11	-0.32	0.09	0.277	2440	0.18	0.03	0.33	0.019	0.015
Phospholipids to total lipids ratio in medium VLDL (%)	2440	-0.32	-0.47	-0.17	<0.0001	2440	-0.03	-0.23	0.17	0.769	2440	0.28	0.13	0.43	0.0003	<0.0001
Total cholesterol to total lipids ratio in medium VLDL (%)	2440	-0.04	-0.20	0.12	0.610	2440	0.12	-0.08	0.32	0.244	2440	-0.06	-0.20	0.09	0.461	0.540
Cholesterol esters to total lipids ratio in medium VLDL (%)	2440	-0.07	-0.23	0.08	0.365	2440	0.10	-0.09	0.30	0.305	2440	-0.02	-0.17	0.12	0.763	0.570
Free cholesterol to total lipids ratio in medium VLDL (%)	2440	0.07	-0.09	0.24	0.382	2440	0.12	-0.09	0.34	0.271	2440	-0.14	-0.29	0.01	0.075	0.105
Triglycerides to total lipids ratio in medium VLDL (%)	2440	0.09	-0.07	0.25	0.259	2440	-0.09	-0.29	0.11	0.379	2440	-0.01	-0.16	0.14	0.891	0.504
Phospholipids to total lipds ratio in small VLDL (%)	2440	-0.28	-0.45	-0.11	0.002	2440	-0.17	-0.39	0.06	0.139	2440	0.34	0.18	0.50	<0.0001	<0.0001
Total cholesterol to total lipids ratio in small VLDL (%)	2440	-0.10	-0.26	0.07	0.246	2440	0.04	-0.17	0.25	0.714	2440	0.05	-0.10	0.20	0.530	0.456
Cholesterol esters to total lipids ratio in small VLDL (%)	2440	-0.05	-0.22	0.12	0.561	2440	0.05	-0.16	0.26	0.644	2440	0.01	-0.15	0.16	0.946	0.829
Free cholesterol to total lipids ratio in small VLDL (%)	2440	-0.33	-0.50	-0.16	0.0001	2440	-0.08	-0.29	0.12	0.441	2440	0.31	0.16	0.46	<0.0001	<0.0001
Triglycerides to total lipids ratio in small VLDL (%)	2440	0.20	0.04	0.36	0.015	2440	0.01	-0.20	0.21	0.942	2440	-0.16	-0.31	-0.01	0.040	0.012
Phospholipids to total lipds ratio in very small VLDL (%)	2440	-0.20	-0.35	-0.05	0.009	2440	0.01	-0.17	0.20	0.878	2440	0.17	0.03	0.31	0.017	0.004
Total cholesterol to total lipids ratio in very small VLDL (%)	2440	0.07	-0.09	0.24	0.396	2440	-0.05	-0.26	0.15	0.605	2440	-0.01	-0.16	0.14	0.889	0.711
Cholesterol esters to total lipids ratio in very small VLDL (%)	2440	0.14	-0.02	0.30	0.084	2440	-0.01	-0.21	0.19	0.951	2440	-0.10	-0.24	0.04	0.174	0.123
Free cholesterol to total lipids ratio in very small VLDL (%)	2440	-0.22	-0.41	-0.02	0.028	2440	-0.16	-0.38	0.06	0.147	2440	0.28	0.12	0.43	0.0004	0.0004
Triglycerides to total lipids ratio in very small VLDL (%)	2440	0.05	-0.11	0.22	0.529	2440	0.05	-0.16	0.26	0.659	2440	-0.10	-0.25	0.06	0.220	0.422
Phospholipids to total lipds ratio in IDL (%)	2440	-0.25	-0.42	-0.08	0.003	2440	-0.17	-0.39	0.05	0.132	2440	0.32	0.16	0.47	<0.0001	<0.0001
Total cholesterol to total lipids ratio in IDL (%)	2440	0.17	0.01	0.34	0.040	2440	0.03	-0.18	0.24	0.797	2440	-0.12	-0.26	0.03	0.112	0.048
Cholesterol esters to total lipids ratio in IDL (%)	2440	0.31	0.15	0.47	0.0001	2440	0.06	-0.14	0.27	0.557	2440	-0.25	-0.39	-0.11	0.0004	<0.0001
Free cholesterol to total lipids ratio in IDL (%)	2440	-0.31	-0.47	-0.15	0.0001	2440	-0.08	-0.30	0.14	0.476	2440	0.31	0.14	0.47	0.0002	<0.0001
Triglycerides to total lipids ratio in IDL (%)	2440	-0.10	-0.26	0.06	0.209	2440	0.03	-0.17	0.24	0.740	2440	0.01	-0.13	0.16	0.875	0.566
Phospholipids to total lipids ratio in large LDL (%)	2440	0.12	-0.04	0.28	0.140	2440	-0.16	-0.35	0.04	0.118	2440	0.01	-0.13	0.16	0.864	0.245
Total cholesterol to total lipids ratio in large LDL (%)	2440	0.00	-0.16	0.17	0.976	2440	0.11	-0.10	0.31	0.309	2440	-0.05	-0.20	0.10	0.548	0.641
Cholesterol esters to total lipids ratio in large LDL (%)	2440	0.05	-0.11	0.22	0.515	2440	0.14	-0.06	0.35	0.161	2440	-0.11	-0.26	0.04	0.152	0.179
Free cholesterol to total lipids ratio in large LDL (%)	2440	-0.15	-0.32	0.01	0.070	2440	-0.17	-0.38	0.05	0.126	2440	0.21	0.05	0.37	0.008	0.005
Triglycerides to total lipids ratio in large LDL (%)	2440	-0.14	-0.30	0.03	0.098	2440	0.00	-0.21	0.21	0.978	2440	0.06	-0.08	0.21	0.389	0.231
Phospholipids to total lipids ratio in medium LDL (%)	2440	0.11	-0.05	0.27	0.169	2440	-0.17	-0.36	0.03	0.102	2440	0.03	-0.12	0.18	0.676	0.256
Total cholesterol to total lipids ratio in medium LDL (%)	2440	-0.03	-0.19	0.13	0.720	2440	0.14	-0.06	0.35	0.164	2440	-0.06	-0.21	0.08	0.393	0.438
Cholesterol esters to total lipids ratio in medium LDL (%)	2440	-0.03	-0.19	0.14	0.752	2440	0.17	-0.03	0.37	0.098	2440	-0.09	-0.24	0.06	0.261	0.284
Free cholesterol to total lipids ratio in medium LDL (%)	2440	0.01	-0.15	0.18	0.885	2440	-0.17	-0.37	0.03	0.089	2440	0.10	-0.05	0.24	0.197	0.224
Triglycerides to total lipids ratio in medium LDL (%)	2440	-0.16	-0.31	-0.01	0.031	2440	0.01	-0.18	0.19	0.958	2440	0.09	-0.04	0.22	0.173	0.060
Phospholipids to total lipds ratio in small LDL (%)	2440	0.08	-0.08	0.24	0.340	2440	-0.18	-0.38	0.02	0.077	2440	0.06	-0.09	0.21	0.422	0.269
Total cholesterol to total lipids ratio in small LDL (%)	2440	-0.08	-0.24	0.08	0.348	2440	0.14	-0.07	0.35	0.187	2440	-0.03	-0.18	0.13	0.729	0.451
Cholesterol esters to total lipids ratio in small LDL (%)	2440	-0.06	-0.22	0.10	0.488	2440	0.15	-0.05	0.35	0.141	2440	-0.04	-0.19	0.11	0.561	0.428
Free cholesterol to total lipids ratio in small LDL (%)	2440	0.01	-0.15	0.18	0.887	2440	-0.14	-0.34	0.05	0.159	2440	0.07	-0.08	0.21	0.374	0.407
Triglycerides to total lipids ratio in small LDL (%)	2440	0.01	-0.15	0.18	0.860	2440	0.08	-0.14	0.29	0.491	2440	-0.08	-0.24	0.07	0.281	0.551

Online Table 3 Associations of regional fat indexes at age 10y with cardiometabolic traits at age 18y in ALSPAC

	**At age 10y**															
	**Trunk fat index (per 1.1 kg/m^2 higher)** *Adj. for age, sex, ethnicity, maternal education, arm fat index, leg fat index*					**Arm fat index (per 0.2 kg/m^2 higher)** *Adj. for age, sex, ethnicity, maternal education, trunk fat index, leg fat index*					**Leg fat index (per 0.9 kg/m^2 higher)** *Adj. for age, sex, ethnicity, maternal education, trunk fat index, arm fat index*					
**Standardized outcome at age 18y**	**N**	**Beta**	**LCL**	**UCL**	**P-value**	**N**	**Beta**	**LCL**	**UCL**	**P-value**	**N**	**Beta**	**LCL**	**UCL**	**P-value**	***P-value for regional heterogeneity***
Phospholipids to total lipds ratio in very large HDL (%)	2440	-0.38	-0.53	-0.24	<0.0001	2440	-0.15	-0.33	0.04	0.115	2440	0.40	0.26	0.54	<0.0001	<0.0001
Total cholesterol to total lipids ratio in very large HDL (%)	2440	0.36	0.22	0.51	<0.0001	2440	0.11	-0.06	0.29	0.206	2440	-0.36	-0.49	-0.22	<0.0001	<0.0001
Cholesterol esters to total lipids ratio in very large HDL (%)	2440	0.36	0.22	0.50	<0.0001	2440	0.13	-0.05	0.30	0.158	2440	-0.37	-0.51	-0.23	<0.0001	<0.0001
Free cholesterol to total lipids ratio in very large HDL (%)	2440	-0.12	-0.27	0.04	0.145	2440	-0.15	-0.33	0.04	0.127	2440	0.25	0.10	0.40	0.001	0.003
Triglycerides to total lipids ratio in very large HDL (%)	2440	0.20	0.02	0.38	0.028	2440	0.20	-0.03	0.42	0.096	2440	-0.31	-0.48	-0.13	0.001	0.001
Phospholipids to total lipds ratio in large HDL (%)	2440	0.31	0.14	0.49	0.0004	2440	0.18	-0.02	0.39	0.072	2440	-0.32	-0.47	-0.16	<0.0001	<0.0001
Total cholesterol to total lipids ratio in large HDL (%)	2440	-0.34	-0.51	-0.16	0.0002	2440	-0.22	-0.43	0.00	0.049	2440	0.37	0.21	0.53	<0.0001	<0.0001
Cholesterol esters to total lipids ratio in large HDL (%)	2440	-0.33	-0.51	-0.15	0.0003	2440	-0.22	-0.43	0.00	0.052	2440	0.36	0.20	0.52	<0.0001	<0.0001
Free cholesterol to total lipids ratio in large HDL (%)	2440	-0.28	-0.45	-0.11	0.002	2440	-0.17	-0.37	0.04	0.110	2440	0.32	0.16	0.48	0.0001	<0.0001
Triglycerides to total lipids ratio in large HDL (%)	2440	0.32	0.15	0.49	0.0002	2440	0.19	-0.04	0.43	0.106	2440	-0.38	-0.55	-0.21	<0.0001	<0.0001
Phospholipids to total lipds ratio in medium HDL (%)	2440	-0.14	-0.30	0.03	0.113	2440	-0.01	-0.21	0.18	0.899	2440	0.11	-0.03	0.25	0.126	0.127
Total cholesterol to total lipids ratio in medium HDL (%)	2440	-0.02	-0.18	0.15	0.825	2440	-0.08	-0.28	0.11	0.408	2440	0.07	-0.08	0.21	0.370	0.571
Cholesterol esters to total lipids ratio in medium HDL (%)	2440	-0.02	-0.18	0.14	0.806	2440	-0.09	-0.28	0.10	0.361	2440	0.07	-0.08	0.22	0.341	0.522
Free cholesterol to total lipids ratio in medium HDL (%)	2440	0.00	-0.15	0.16	0.951	2440	-0.01	-0.18	0.16	0.912	2440	0.03	-0.10	0.16	0.674	0.953
Triglycerides to total lipids ratio in medium HDL (%)	2440	0.27	0.09	0.45	0.003	2440	0.19	-0.02	0.41	0.082	2440	-0.33	-0.51	-0.16	0.0002	<0.0001
Phospholipids to total lipds ratio in small HDL (%)	2440	0.16	0.01	0.31	0.032	2440	0.06	-0.12	0.24	0.498	2440	-0.19	-0.32	-0.05	0.007	0.005
Total cholesterol to total lipids ratio in small HDL (%)	2440	-0.21	-0.36	-0.07	0.004	2440	-0.12	-0.30	0.07	0.216	2440	0.27	0.13	0.40	0.0001	<0.0001
Cholesterol esters to total lipids ratio in small HDL (%)	2440	-0.18	-0.33	-0.03	0.016	2440	-0.08	-0.26	0.10	0.383	2440	0.22	0.08	0.36	0.002	0.001
Free cholesterol to total lipids ratio in small HDL (%)	2440	-0.11	-0.29	0.06	0.199	2440	-0.17	-0.38	0.03	0.098	2440	0.17	0.01	0.33	0.034	0.032
Triglycerides to total lipids ratio in small HDL (%)	2440	0.18	0.02	0.35	0.033	2440	0.22	0.00	0.44	0.052	2440	-0.32	-0.49	-0.15	0.0003	0.0003
Mean diameter for VLDL particles (nm)	2440	0.44	0.27	0.60	<0.0001	2440	0.08	-0.12	0.28	0.440	2440	-0.37	-0.52	-0.22	<0.0001	<0.0001
Mean diameter for LDL particles (nm)	2440	-0.14	-0.30	0.03	0.101	2440	-0.01	-0.19	0.18	0.938	2440	0.05	-0.09	0.19	0.501	0.308
Mean diameter for HDL particles (nm)	2440	-0.35	-0.50	-0.20	<0.0001	2440	-0.22	-0.40	-0.04	0.018	2440	0.44	0.30	0.58	<0.0001	<0.0001
Serum total cholesterol (mmol/l)	2440	-0.01	-0.17	0.16	0.949	2440	-0.01	-0.21	0.19	0.897	2440	0.06	-0.09	0.21	0.400	0.801
Total cholesterol in VLDL (mmol/l)	2440	0.32	0.15	0.49	0.0002	2440	0.13	-0.09	0.35	0.243	2440	-0.31	-0.47	-0.15	0.0002	<0.0001
Remnant cholesterol (non-HDL, non-LDL -cholesterol) (mmol/l)	2440	0.21	0.04	0.38	0.018	2440	0.10	-0.12	0.32	0.362	2440	-0.20	-0.36	-0.04	0.015	0.004
Total cholesterol in LDL (mmol/l)	2440	0.01	-0.16	0.17	0.950	2440	0.04	-0.17	0.25	0.695	2440	0.00	-0.15	0.16	0.958	0.973
Total cholesterol in HDL (mmol/l)	2440	-0.27	-0.42	-0.12	0.001	2440	-0.22	-0.42	-0.03	0.022	2440	0.42	0.27	0.56	<0.0001	<0.0001
Total cholesterol in HDL2 (mmol/l)	2440	-0.30	-0.45	-0.14	0.0002	2440	-0.24	-0.43	-0.04	0.018	2440	0.44	0.29	0.59	<0.0001	<0.0001
Total cholesterol in HDL3 (mmol/l)	2440	-0.21	-0.37	-0.06	0.006	2440	-0.19	-0.38	0.00	0.045	2440	0.35	0.21	0.50	<0.0001	<0.0001
Esterified cholesterol (mmol/l)	2430	0.02	-0.14	0.18	0.779	2430	-0.02	-0.23	0.18	0.832	2430	0.04	-0.11	0.19	0.575	0.923
Free cholesterol (mmol/l)	2428	-0.05	-0.21	0.11	0.513	2428	0.00	-0.19	0.19	0.994	2428	0.10	-0.05	0.24	0.179	0.417
Serum total triglycerides (mmol/l)	2440	0.31	0.14	0.47	0.0004	2440	0.14	-0.09	0.36	0.234	2440	-0.31	-0.48	-0.14	0.0003	<0.0001
Triglycerides in VLDL (mmol/l)	2440	0.38	0.21	0.55	<0.0001	2440	0.14	-0.09	0.37	0.238	2440	-0.36	-0.53	-0.19	<0.0001	<0.0001
Triglycerides in LDL (mmol/l)	2440	-0.10	-0.26	0.05	0.188	2440	0.06	-0.13	0.25	0.542	2440	0.04	-0.10	0.19	0.576	0.394
Triglycerides in HDL (mmol/l)	2440	0.15	-0.02	0.31	0.078	2440	0.08	-0.12	0.29	0.422	2440	-0.16	-0.32	0.00	0.050	0.047
Diacylglycerol (mmol/l)	2373	0.17	0.00	0.35	0.048	2373	0.02	-0.20	0.24	0.830	2373	-0.09	-0.25	0.07	0.283	0.120
Ratio of diacylglycerol to triglycerides	2374	0.04	-0.13	0.21	0.649	2374	0.00	-0.20	0.19	0.967	2374	0.04	-0.11	0.19	0.579	0.957
Total phosphoglycerides (mmol/l)	2428	-0.11	-0.27	0.04	0.148	2428	-0.01	-0.21	0.18	0.914	2428	0.13	-0.01	0.28	0.076	0.106
Ratio of triglycerides to phosphoglycerides	2428	0.43	0.26	0.61	<0.0001	2428	0.07	-0.17	0.31	0.562	2428	-0.36	-0.53	-0.19	<0.0001	<0.0001
Phosphatidylcholine and other cholines (mmol/l)	2409	-0.09	-0.25	0.06	0.239	2409	-0.06	-0.25	0.13	0.560	2409	0.14	-0.01	0.28	0.067	0.122
Total cholines (mmol/l)	2430	-0.13	-0.28	0.03	0.108	2430	-0.03	-0.22	0.16	0.776	2430	0.15	0.01	0.30	0.040	0.053
Apolipoprotein A-I (g/l)	2440	-0.16	-0.31	-0.02	0.031	2440	-0.18	-0.37	0.00	0.052	2440	0.32	0.17	0.46	<0.0001	<0.0001
Apolipoprotein B (g/l)	2440	0.22	0.05	0.39	0.012	2440	0.12	-0.09	0.34	0.266	2440	-0.23	-0.39	-0.07	0.005	0.001
Ratio of apolipoprotein B to apolipoprotein A-I	2440	0.29	0.11	0.46	0.001	2440	0.21	-0.01	0.44	0.060	2440	-0.37	-0.53	-0.20	<0.0001	<0.0001
Total fatty acids (mmol/l)	2430	0.09	-0.07	0.25	0.267	2430	0.06	-0.14	0.27	0.554	2430	-0.07	-0.23	0.08	0.365	0.388
Estimated description of fatty acid chain length, not actual carbon number	2430	0.12	-0.04	0.28	0.149	2430	-0.07	-0.27	0.13	0.489	2430	-0.02	-0.16	0.12	0.803	0.402
Estimated degree of unsaturation	2430	-0.01	-0.17	0.16	0.940	2430	-0.18	-0.38	0.02	0.072	2430	0.18	0.02	0.33	0.024	0.060
22:6, docosahexaenoic acid (mmol/l)	2430	0.07	-0.09	0.22	0.390	2430	-0.03	-0.22	0.15	0.743	2430	-0.01	-0.16	0.13	0.862	0.739
18:2, linoleic acid (mmol/l)	2429	0.02	-0.13	0.18	0.766	2429	-0.02	-0.21	0.18	0.856	2429	0.02	-0.12	0.17	0.753	0.962

Online Table 3 Associations of regional fat indexes at age 10y with cardiometabolic traits at age 18y in ALSPAC

	At age 10y															
	**Trunk fat index (per 1.1 kg/m^2 higher)**					**Arm fat index (per 0.2 kg/m^2 higher)**					**Leg fat index (per 0.9 kg/m^2 higher)**					
	Adj. for age, sex, ethnicity, maternal education, arm fat index, leg fat index					*Adj. for age, sex, ethnicity, maternal education, trunk fat index, leg fat index*					*Adj. for age, sex, ethnicity, maternal education, trunk fat index, arm fat index*					***P-value for regional heterogeneity***
**Standardized outcome at age 18y**	**N**	**Beta**	**LCL**	**UCL**	**P-value**	**N**	**Beta**	**LCL**	**UCL**	**P-value**	**N**	**Beta**	**LCL**	**UCL**	**P-value**	
Conjugated linoleic acid (mmol/l)	2429	0.10	-0.06	0.26	0.240	2429	-0.03	-0.24	0.17	0.763	2429	-0.01	-0.16	0.13	0.850	0.586
Omega-3 fatty acids (mmol/l)	2430	0.11	-0.04	0.27	0.146	2430	0.04	-0.16	0.23	0.729	2430	-0.08	-0.24	0.07	0.301	0.266
Omega-6 fatty acids (mmol/l)	2430	0.02	-0.13	0.18	0.763	2430	-0.01	-0.21	0.19	0.898	2430	0.03	-0.12	0.18	0.673	0.961
Polyunsaturated fatty acids (mmol/l)	2429	0.04	-0.12	0.19	0.655	2429	-0.01	-0.21	0.19	0.952	2429	0.02	-0.13	0.17	0.809	0.967
Monounsaturated fatty acids; 16:1, 18:1 (mmol/l)	2430	0.12	-0.04	0.28	0.135	2430	0.09	-0.13	0.30	0.431	2430	-0.11	-0.27	0.05	0.180	0.149
Saturated fatty acids (mmol/l)	2429	0.09	-0.07	0.25	0.289	2429	0.09	-0.12	0.29	0.415	2429	-0.10	-0.26	0.06	0.213	0.269
Ratio of 22:6 docosahexaenoic acid to total fatty acids (%)	2431	0.06	-0.10	0.22	0.465	2431	-0.11	-0.30	0.08	0.261	2431	0.04	-0.10	0.18	0.560	0.551
Ratio of 18:2 linoleic acid to total fatty acids (%)	2430	-0.12	-0.29	0.04	0.149	2430	-0.11	-0.30	0.08	0.248	2430	0.13	-0.01	0.28	0.069	0.063
Ratio of conjugated linoleic acid to total fatty acids (%)	2430	0.09	-0.06	0.25	0.232	2430	-0.07	-0.26	0.13	0.506	2430	0.00	-0.14	0.14	0.968	0.566
Ratio of omega-3 fatty acids to total fatty acids (%)	2431	0.10	-0.06	0.26	0.213	2431	-0.05	-0.25	0.15	0.620	2431	-0.03	-0.19	0.13	0.715	0.485
Ratio of omega-6 fatty acids to total fatty acids (%)	2431	-0.17	-0.33	0.00	0.044	2431	-0.12	-0.32	0.08	0.226	2431	0.20	0.05	0.35	0.010	0.006
Ratio of polyunsaturated fatty acids to total fatty acids (%)	2430	-0.13	-0.30	0.03	0.109	2430	-0.13	-0.33	0.07	0.212	2430	0.18	0.03	0.33	0.022	0.021
Ratio of monounsaturated fatty acids to total fatty acids (%)	2431	0.11	-0.05	0.28	0.174	2431	0.10	-0.12	0.31	0.376	2431	-0.12	-0.27	0.03	0.124	0.111
Ratio of saturated fatty acids to total fatty acids (%)	2430	0.01	-0.16	0.18	0.928	2430	0.03	-0.19	0.25	0.815	2430	-0.06	-0.22	0.10	0.478	0.822
Insulin (mu/l)	2478	0.27	0.04	0.49	0.023	2478	0.25	-0.05	0.55	0.105	2478	-0.32	-0.59	-0.05	0.020	0.028
Glucose (mmol/l)	2439	-0.01	-0.16	0.15	0.941	2439	0.22	-0.02	0.45	0.070	2439	-0.17	-0.32	-0.01	0.037	0.074
Lactate (mmol/l)	2439	-0.07	-0.23	0.10	0.423	2439	0.14	-0.07	0.36	0.191	2439	-0.03	-0.20	0.14	0.745	0.476
Pyruvate (mmol/l)	2439	0.03	-0.12	0.19	0.665	2439	0.15	-0.05	0.35	0.130	2439	-0.07	-0.23	0.08	0.339	0.345
Citrate (mmol/l)	2439	-0.13	-0.28	0.02	0.084	2439	0.07	-0.11	0.25	0.432	2439	-0.09	-0.23	0.06	0.228	0.399
Alanine (mmol/l)	2439	0.12	-0.04	0.29	0.149	2439	0.06	-0.14	0.26	0.541	2439	-0.15	-0.30	0.00	0.046	0.064
Glutamine (mmol/l)	2439	0.12	-0.02	0.27	0.099	2439	-0.13	-0.30	0.05	0.163	2439	-0.04	-0.17	0.10	0.614	0.176
Histidine (mmol/l)	2439	0.04	-0.13	0.20	0.681	2439	0.01	-0.19	0.21	0.913	2439	-0.03	-0.18	0.13	0.738	0.886
Isoleucine (mmol/l)	2439	0.32	0.16	0.48	<0.0001	2439	0.17	-0.02	0.36	0.077	2439	-0.37	-0.52	-0.22	<0.0001	<0.0001
Leucine (mmol/l)	2439	0.26	0.12	0.40	0.0002	2439	0.08	-0.09	0.25	0.378	2439	-0.26	-0.39	-0.13	<0.0001	<0.0001
Valine (mmol/l)	2439	0.25	0.11	0.40	0.001	2439	0.18	0.00	0.37	0.053	2439	-0.30	-0.43	-0.16	<0.0001	<0.0001
Phenylalanine (mmol/l)	2438	0.14	-0.03	0.31	0.103	2438	0.01	-0.18	0.20	0.947	2438	-0.04	-0.19	0.11	0.619	0.389
Tyrosine (mmol/l)	2439	0.27	0.11	0.42	0.001	2439	-0.05	-0.24	0.14	0.605	2439	-0.08	-0.24	0.07	0.284	0.012
Acetate (mmol/l)	2438	-0.02	-0.19	0.15	0.832	2438	-0.06	-0.29	0.16	0.592	2438	0.03	-0.09	0.15	0.590	0.701
Acetoacetate (mmol/l)	2439	-0.10	-0.24	0.04	0.161	2439	0.00	-0.22	0.23	0.968	2439	0.10	-0.07	0.26	0.244	0.172
3-hydroxybutyrate (mmol/l)	2436	-0.18	-0.33	-0.03	0.016	2436	0.03	-0.16	0.23	0.734	2436	0.14	-0.01	0.29	0.063	0.017
Creatinine (mmol/l)	2439	0.07	-0.06	0.20	0.300	2439	-0.02	-0.19	0.15	0.825	2439	-0.04	-0.17	0.09	0.557	0.532
Albumin (signal area)	2440	-0.01	-0.15	0.13	0.899	2440	-0.10	-0.28	0.08	0.284	2440	0.05	-0.09	0.20	0.471	0.588
Glycoprotein acetyls, mainly a1-acid glycoprotein (mmol/l)	2439	0.29	0.13	0.46	0.001	2439	0.01	-0.19	0.22	0.907	2439	-0.10	-0.27	0.06	0.204	0.008
C-reactive protein (mg/l)	2519	0.01	-0.16	0.19	0.875	2519	0.03	-0.15	0.21	0.752	2519	0.04	-0.12	0.20	0.620	0.983

Complete case sample

	At age 10y															
	**Trunk fat index (per 1.1 kg/m^2 higher)**					**Arm fat index (per 0.2 kg/m^2 higher)**					**Leg fat index (per 0.9 kg/m^2 higher)**					
	Adj. for age, sex, ethnicity, maternal education, arm fat index, leg fat index					*Adj. for age, sex, ethnicity, maternal education, trunk fat index, leg fat index*					*Adj. for age, sex, ethnicity, maternal education, trunk fat index, arm fat index*					***P-value for regional heterogeneity***
**Standardized outcome at age 18y**	**N**	**Beta**	**LCL**	**UCL**	**P-value**	**N**	**Beta**	**LCL**	**UCL**	**P-value**	**N**	**Beta**	**LCL**	**UCL**	**P-value**	
Systolic blood pressure (mmHg)	1722	0.10	-0.06	0.26	0.221	1722	-0.02	-0.22	0.17	0.807	1722	0.11	-0.05	0.27	0.173	0.685
Diastolic blood pressure (mmHg)	1722	0.17	-0.02	0.35	0.074	1722	0.05	-0.19	0.29	0.690	1722	0.02	-0.16	0.21	0.822	0.556
Concentration of chylomicrons and extremely large VLDL particles (mol/l)	1722	0.38	0.16	0.60	0.001	1722	0.07	-0.20	0.34	0.598	1722	-0.27	-0.46	-0.09	0.004	0.0001
Total lipids in chylomicrons and extremely large VLDL (mmol/l)	1722	0.38	0.17	0.60	0.0005	1722	0.07	-0.20	0.34	0.614	1722	-0.27	-0.46	-0.09	0.004	<0.0001

Online Table 3 Associations of regional fat indexes at age 10y with cardiometabolic traits at age 18y in ALSPAC

	At age 10y<br>Trunk fat index (per 1.1 kg/m^2 higher)<br>*Adj. for age, sex, ethnicity, maternal education, arm fat index, leg fat index*					Arm fat index (per 0.2 kg/m^2 higher)<br>*Adj. for age, sex, ethnicity, maternal education, trunk fat index, leg fat index*					Leg fat index (per 0.9 kg/m^2 higher)<br>*Adj. for age, sex, ethnicity, maternal education, trunk fat index, arm fat index*					
**Standardized outcome at age 18y**	**N**	**Beta**	**LCL**	**UCL**	**P-value**	**N**	**Beta**	**LCL**	**UCL**	**P-value**	**N**	**Beta**	**LCL**	**UCL**	**P-value**	***P-value for regional heterogeneity***
Phospholipids in chylomicrons and extremely large VLDL (mmol/l)	1722	0.37	0.16	0.59	0.001	1722	0.07	-0.20	0.34	0.600	1722	-0.27	-0.45	-0.08	0.005	0.0001
Total cholesterol in chylomicrons and extremely large VLDL (mmol/l)	1722	0.38	0.16	0.59	0.001	1722	0.04	-0.23	0.31	0.772	1722	-0.25	-0.44	-0.06	0.012	0.0003
Cholesterol esters in chylomicrons and extremely large VLDL (mmol/l)	1722	0.37	0.16	0.58	0.001	1722	0.01	-0.25	0.28	0.922	1722	-0.23	-0.42	-0.03	0.023	0.001
Free cholesterol in chylomicrons and extremely large VLDL (mmol/l)	1722	0.37	0.15	0.58	0.001	1722	0.07	-0.20	0.34	0.609	1722	-0.26	-0.45	-0.08	0.006	0.0002
Triglycerides in chylomicrons and extremely large VLDL (mmol/l)	1722	0.38	0.17	0.60	0.0005	1722	0.08	-0.19	0.34	0.582	1722	-0.28	-0.47	-0.09	0.003	<0.0001
Concentration of very large VLDL particles (mol/l)	1722	0.39	0.18	0.60	0.0003	1722	0.07	-0.20	0.34	0.623	1722	-0.28	-0.47	-0.09	0.004	<0.0001
Total lipids in very large VLDL (mmol/l)	1722	0.39	0.18	0.60	0.0003	1722	0.06	-0.21	0.33	0.652	1722	-0.28	-0.47	-0.09	0.004	<0.0001
Phospholipids in very large VLDL (mmol/l)	1722	0.38	0.16	0.59	0.001	1722	0.06	-0.21	0.33	0.664	1722	-0.27	-0.46	-0.08	0.006	0.0002
Total cholesterol in very large VLDL (mmol/l)	1722	0.39	0.18	0.60	0.0003	1722	0.05	-0.22	0.33	0.691	1722	-0.27	-0.46	-0.08	0.006	0.0001
Cholesterol esters in very large VLDL (mmol/l)	1722	0.40	0.19	0.61	0.0002	1722	0.05	-0.22	0.32	0.705	1722	-0.28	-0.47	-0.08	0.005	<0.0001
Free cholesterol in very large VLDL (mmol/l)	1722	0.38	0.16	0.59	0.001	1722	0.06	-0.21	0.33	0.676	1722	-0.26	-0.45	-0.07	0.007	0.0002
Triglycerides in very large VLDL (mmol/l)	1722	0.39	0.18	0.60	0.0003	1722	0.06	-0.20	0.33	0.638	1722	-0.28	-0.47	-0.10	0.003	<0.0001
Concentration of large VLDL particles (mol/l)	1722	0.40	0.19	0.61	0.0002	1722	0.06	-0.20	0.33	0.634	1722	-0.29	-0.48	-0.10	0.002	<0.0001
Total lipids in large VLDL (mmol/l)	1722	0.40	0.19	0.61	0.0002	1722	0.06	-0.21	0.33	0.659	1722	-0.29	-0.48	-0.10	0.003	<0.0001
Phospholipids in large VLDL (mmol/l)	1722	0.39	0.19	0.60	0.0002	1722	0.06	-0.21	0.32	0.674	1722	-0.28	-0.47	-0.09	0.004	<0.0001
Total cholesterol in large VLDL (mmol/l)	1722	0.39	0.19	0.60	0.0002	1722	0.04	-0.22	0.31	0.744	1722	-0.27	-0.46	-0.08	0.006	<0.0001
Cholesterol esters in large VLDL (mmol/l)	1722	0.40	0.19	0.61	0.0002	1722	0.03	-0.24	0.30	0.805	1722	-0.26	-0.46	-0.07	0.008	0.0001
Free cholesterol in large VLDL (mmol/l)	1722	0.39	0.18	0.60	0.0003	1722	0.05	-0.21	0.32	0.686	1722	-0.28	-0.47	-0.08	0.005	<0.0001
Triglycerides in large VLDL (mmol/l)	1722	0.40	0.19	0.61	0.0001	1722	0.07	-0.20	0.33	0.620	1722	-0.30	-0.49	-0.11	0.002	<0.0001
Concentration of medium VLDL particles (mol/l)	1722	0.41	0.20	0.62	0.0001	1722	0.07	-0.20	0.34	0.617	1722	-0.31	-0.50	-0.12	0.002	<0.0001
Total lipids in medium VLDL (mmol/l)	1722	0.41	0.20	0.62	0.0001	1722	0.06	-0.21	0.33	0.648	1722	-0.30	-0.50	-0.11	0.002	<0.0001
Phospholipids in medium VLDL (mmol/l)	1722	0.40	0.19	0.60	0.0002	1722	0.07	-0.20	0.34	0.632	1722	-0.30	-0.49	-0.10	0.003	<0.0001
Total cholesterol in medium VLDL (mmol/l)	1722	0.37	0.16	0.58	0.001	1722	0.05	-0.22	0.32	0.732	1722	-0.26	-0.46	-0.06	0.009	0.0003
Cholesterol esters in medium VLDL (mmol/l)	1722	0.35	0.13	0.56	0.001	1722	0.03	-0.24	0.30	0.828	1722	-0.23	-0.43	-0.03	0.023	0.001
Free cholesterol in medium VLDL (mmol/l)	1722	0.38	0.17	0.59	0.0004	1722	0.07	-0.20	0.33	0.631	1722	-0.29	-0.48	-0.09	0.004	<0.0001
Triglycerides in medium VLDL (mmol/l)	1722	0.43	0.22	0.64	<0.0001	1722	0.07	-0.20	0.34	0.622	1722	-0.32	-0.51	-0.13	0.001	<0.0001
Concentration of small VLDL particles (mol/l)	1722	0.36	0.15	0.56	0.001	1722	0.08	-0.19	0.34	0.574	1722	-0.29	-0.48	-0.09	0.004	0.0001
Total lipids in small VLDL (mmol/l)	1722	0.35	0.15	0.56	0.001	1722	0.08	-0.19	0.35	0.575	1722	-0.28	-0.48	-0.09	0.004	0.0002
Phospholipids in small VLDL (mmol/l)	1722	0.33	0.13	0.53	0.001	1722	0.06	-0.19	0.32	0.622	1722	-0.26	-0.45	-0.07	0.009	0.000
Total cholesterol in small VLDL (mmol/l)	1722	0.32	0.10	0.53	0.004	1722	0.07	-0.21	0.35	0.608	1722	-0.26	-0.46	-0.06	0.012	0.001
Cholesterol esters in small VLDL (mmol/l)	1722	0.30	0.08	0.52	0.007	1722	0.07	-0.21	0.36	0.624	1722	-0.25	-0.45	-0.05	0.015	0.002
Free cholesterol in small VLDL (mmol/l)	1722	0.30	0.10	0.51	0.003	1722	0.07	-0.19	0.34	0.593	1722	-0.25	-0.44	-0.05	0.013	0.001
Triglycerides in small VLDL (mmol/l)	1722	0.35	0.15	0.56	0.001	1722	0.08	-0.19	0.34	0.574	1722	-0.28	-0.47	-0.09	0.004	0.0001
Concentration of very small VLDL particles (mol/l)	1722	0.12	-0.09	0.33	0.252	1722	0.06	-0.21	0.33	0.663	1722	-0.11	-0.31	0.10	0.298	0.334
Total lipids in very small VLDL (mmol/l)	1722	0.18	-0.04	0.39	0.111	1722	0.07	-0.21	0.35	0.609	1722	-0.16	-0.37	0.04	0.122	0.097
Phospholipids in very small VLDL (mmol/l)	1722	0.08	-0.13	0.29	0.432	1722	0.04	-0.23	0.31	0.770	1722	-0.06	-0.27	0.14	0.532	0.630
Total cholesterol in very small VLDL (mmol/l)	1722	0.19	-0.03	0.42	0.091	1722	0.07	-0.22	0.36	0.630	1722	-0.18	-0.38	0.02	0.073	0.053
Cholesterol esters in very small VLDL (mmol/l)	1722	0.23	0.01	0.45	0.044	1722	0.08	-0.21	0.37	0.589	1722	-0.22	-0.42	-0.02	0.032	0.015
Free cholesterol in very small VLDL (mmol/l)	1722	0.09	-0.13	0.31	0.434	1722	0.04	-0.23	0.31	0.779	1722	-0.09	-0.29	0.11	0.399	0.539
Triglycerides in very small VLDL (mmol/l)	1722	0.16	-0.04	0.35	0.108	1722	0.09	-0.17	0.34	0.498	1722	-0.16	-0.36	0.04	0.111	0.099
Concentration of IDL particles (mol/l)	1722	0.03	-0.18	0.24	0.793	1722	0.04	-0.22	0.31	0.758	1722	-0.03	-0.22	0.17	0.799	0.920
Total lipids in IDL (mmol/l)	1722	0.05	-0.16	0.26	0.667	1722	0.04	-0.23	0.31	0.784	1722	-0.03	-0.23	0.17	0.749	0.867
Phospholipids in IDL (mmol/l)	1722	0.01	-0.20	0.22	0.917	1722	0.02	-0.24	0.29	0.858	1722	0.01	-0.19	0.20	0.958	0.996
Total cholesterol in IDL (mmol/l)	1722	0.08	-0.14	0.30	0.463	1722	0.03	-0.24	0.31	0.802	1722	-0.06	-0.26	0.14	0.581	0.674
Cholesterol esters in IDL (mmol/l)	1722	0.12	-0.10	0.34	0.283	1722	0.04	-0.24	0.32	0.776	1722	-0.09	-0.29	0.11	0.393	0.415
Free cholesterol in IDL (mmol/l)	1722	-0.01	-0.22	0.19	0.897	1722	0.02	-0.24	0.28	0.882	1722	0.02	-0.17	0.22	0.836	0.973
Triglycerides in IDL (mmol/l)	1722	-0.09	-0.27	0.10	0.358	1722	0.06	-0.18	0.31	0.603	1722	0.03	-0.16	0.22	0.740	0.626
Concentration of large LDL particles (mol/l)	1722	0.04	-0.17	0.25	0.713	1722	0.02	-0.24	0.29	0.853	1722	-0.01	-0.20	0.19	0.947	0.954
Total lipids in large LDL (mmol/l)	1722	0.04	-0.17	0.25	0.709	1722	0.03	-0.24	0.29	0.843	1722	-0.01	-0.20	0.19	0.935	0.950
Phospholipids in large LDL (mmol/l)	1722	0.07	-0.14	0.28	0.524	1722	0.02	-0.24	0.29	0.865	1722	-0.02	-0.22	0.17	0.820	0.838

Online Table 3 Associations of regional fat indexes at age 10y with cardiometabolic traits at age 18y in ALSPAC

	At age 10y															
	**Trunk fat index (per 1.1 kg/m^2 higher)**					**Arm fat index (per 0.2 kg/m^2 higher)**					**Leg fat index (per 0.9 kg/m^2 higher)**					
	Adj. for age, sex, ethnicity, maternal education, arm fat index, leg fat index					*Adj. for age, sex, ethnicity, maternal education, trunk fat index, leg fat index*					*Adj. for age, sex, ethnicity, maternal education, trunk fat index, arm fat index*					
**Standardized outcome at age 18y**	**N**	**Beta**	**LCL**	**UCL**	**P-value**	**N**	**Beta**	**LCL**	**UCL**	**P-value**	**N**	**Beta**	**LCL**	**UCL**	**P-value**	***P-value for regional heterogeneity***
Total cholesterol in large LDL (mmol/l)	1722	0.05	-0.16	0.26	0.621	1722	0.02	-0.24	0.29	0.858	1722	-0.02	-0.21	0.18	0.869	0.901
Cholesterol esters in large LDL (mmol/l)	1722	0.07	-0.14	0.28	0.513	1722	0.02	-0.24	0.29	0.856	1722	-0.03	-0.22	0.17	0.785	0.816
Free cholesterol in large LDL (mmol/l)	1722	0.00	-0.21	0.21	0.995	1722	0.02	-0.24	0.29	0.867	1722	0.02	-0.18	0.21	0.860	0.991
Triglycerides in large LDL (mmol/l)	1722	-0.12	-0.31	0.07	0.202	1722	0.05	-0.20	0.29	0.713	1722	0.08	-0.10	0.27	0.392	0.357
Concentration of medium LDL particles (mol/l)	1722	0.07	-0.14	0.28	0.511	1722	0.03	-0.24	0.29	0.836	1722	-0.03	-0.22	0.17	0.779	0.811
Total lipids in medium LDL (mmol/l)	1722	0.06	-0.14	0.27	0.542	1722	0.03	-0.24	0.29	0.845	1722	-0.02	-0.22	0.17	0.818	0.846
Phospholipids in medium LDL (mmol/l)	1722	0.10	-0.10	0.31	0.318	1722	0.03	-0.24	0.29	0.844	1722	-0.04	-0.23	0.15	0.687	0.627
Total cholesterol in medium LDL (mmol/l)	1722	0.07	-0.14	0.29	0.489	1722	0.03	-0.24	0.30	0.847	1722	-0.03	-0.23	0.16	0.742	0.781
Cholesterol esters in medium LDL (mmol/l)	1722	0.08	-0.13	0.29	0.452	1722	0.02	-0.25	0.29	0.865	1722	-0.04	-0.23	0.16	0.717	0.748
Free cholesterol in medium LDL (mmol/l)	1722	0.04	-0.16	0.25	0.672	1722	0.04	-0.23	0.30	0.778	1722	-0.02	-0.21	0.18	0.861	0.908
Triglycerides in medium LDL (mmol/l)	1722	-0.12	-0.31	0.08	0.250	1722	0.02	-0.22	0.26	0.855	1722	0.10	-0.08	0.28	0.285	0.350
Concentration of small LDL particles (mol/l)	1722	0.08	-0.13	0.29	0.449	1722	0.03	-0.24	0.29	0.841	1722	-0.03	-0.22	0.16	0.755	0.759
Total lipids in small LDL (mmol/l)	1722	0.08	-0.13	0.29	0.436	1722	0.02	-0.24	0.29	0.859	1722	-0.03	-0.23	0.16	0.746	0.748
Phospholipids in small LDL (mmol/l)	1722	0.11	-0.10	0.32	0.289	1722	0.02	-0.24	0.28	0.906	1722	-0.04	-0.23	0.15	0.700	0.606
Total cholesterol in small LDL (mmol/l)	1722	0.08	-0.13	0.29	0.471	1722	0.02	-0.25	0.29	0.868	1722	-0.03	-0.23	0.17	0.755	0.775
Cholesterol esters in small LDL (mmol/l)	1722	0.08	-0.14	0.29	0.477	1722	0.02	-0.25	0.29	0.888	1722	-0.03	-0.23	0.17	0.779	0.792
Free cholesterol in small LDL (mmol/l)	1722	0.08	-0.13	0.29	0.477	1722	0.04	-0.23	0.31	0.785	1722	-0.04	-0.24	0.15	0.665	0.721
Triglycerides in small LDL (mmol/l)	1722	0.03	-0.17	0.24	0.743	1722	0.04	-0.21	0.29	0.749	1722	-0.02	-0.20	0.17	0.865	0.930
Concentration of very large HDL particles (mol/l)	1722	-0.36	-0.55	-0.18	0.0001	1722	-0.10	-0.32	0.12	0.363	1722	0.37	0.20	0.54	<0.0001	<0.0001
Total lipids in very large HDL (mmol/l)	1722	-0.35	-0.54	-0.16	0.0003	1722	-0.09	-0.31	0.13	0.415	1722	0.35	0.18	0.52	<0.0001	<0.0001
Phospholipids in very large HDL (mmol/l)	1722	-0.40	-0.58	-0.22	<0.0001	1722	-0.11	-0.33	0.11	0.313	1722	0.40	0.24	0.57	<0.0001	<0.0001
Total cholesterol in very large HDL (mmol/l)	1722	-0.27	-0.46	-0.07	0.008	1722	-0.07	-0.30	0.16	0.568	1722	0.27	0.10	0.45	0.002	0.001
Cholesterol esters in very large HDL (mmol/l)	1722	-0.23	-0.43	-0.03	0.024	1722	-0.06	-0.29	0.18	0.630	1722	0.24	0.06	0.42	0.008	0.005
Free cholesterol in very large HDL (mmol/l)	1722	-0.34	-0.53	-0.15	0.0004	1722	-0.09	-0.31	0.13	0.417	1722	0.35	0.18	0.53	<0.0001	<0.0001
Triglycerides in very large HDL (mmol/l)	1722	-0.10	-0.31	0.10	0.304	1722	0.03	-0.22	0.27	0.815	1722	0.07	-0.13	0.26	0.492	0.532
Concentration of large HDL particles (mol/l)	1722	-0.38	-0.57	-0.19	0.0001	1722	-0.13	-0.37	0.11	0.285	1722	0.39	0.22	0.56	<0.0001	<0.0001
Total lipids in large HDL (mmol/l)	1722	-0.38	-0.57	-0.19	<0.0001	1722	-0.13	-0.36	0.11	0.283	1722	0.40	0.23	0.57	<0.0001	<0.0001
Phospholipids in large HDL (mmol/l)	1722	-0.36	-0.54	-0.17	0.0002	1722	-0.13	-0.36	0.11	0.284	1722	0.38	0.22	0.55	<0.0001	<0.0001
Total cholesterol in large HDL (mmol/l)	1722	-0.41	-0.60	-0.22	<0.0001	1722	-0.13	-0.36	0.11	0.294	1722	0.41	0.23	0.58	<0.0001	<0.0001
Cholesterol esters in large HDL (mmol/l)	1722	-0.41	-0.60	-0.22	<0.0001	1722	-0.13	-0.37	0.11	0.287	1722	0.41	0.23	0.58	<0.0001	<0.0001
Free cholesterol in large HDL (mmol/l)	1722	-0.40	-0.59	-0.21	<0.0001	1722	-0.12	-0.35	0.11	0.313	1722	0.40	0.23	0.57	<0.0001	<0.0001
Triglycerides in large HDL (mmol/l)	1722	-0.04	-0.23	0.15	0.663	1722	-0.08	-0.31	0.14	0.478	1722	0.10	-0.08	0.28	0.260	0.485
Concentration of medium HDL particles (mol/l)	1722	-0.07	-0.29	0.14	0.498	1722	-0.14	-0.39	0.11	0.279	1722	0.21	0.03	0.39	0.022	0.079
Total lipids in medium HDL (mmol/l)	1722	-0.09	-0.30	0.12	0.384	1722	-0.14	-0.39	0.11	0.263	1722	0.23	0.05	0.41	0.015	0.048
Phospholipids in medium HDL (mmol/l)	1722	-0.11	-0.33	0.10	0.291	1722	-0.14	-0.39	0.11	0.278	1722	0.24	0.06	0.42	0.010	0.032
Total cholesterol in medium HDL (mmol/l)	1722	-0.11	-0.32	0.10	0.291	1722	-0.14	-0.39	0.11	0.273	1722	0.23	0.05	0.42	0.014	0.038
Cholesterol esters in medium HDL (mmol/l)	1722	-0.12	-0.33	0.09	0.265	1722	-0.14	-0.39	0.11	0.276	1722	0.24	0.05	0.42	0.013	0.035
Free cholesterol in medium HDL (mmol/l)	1722	-0.08	-0.28	0.12	0.453	1722	-0.13	-0.37	0.10	0.269	1722	0.21	0.03	0.39	0.020	0.065
Triglycerides in medium HDL (mmol/l)	1722	0.29	0.09	0.49	0.005	1722	-0.04	-0.28	0.20	0.759	1722	-0.14	-0.32	0.05	0.150	0.020
Concentration of small HDL particles (mol/l)	1722	0.13	-0.09	0.34	0.264	1722	-0.09	-0.33	0.16	0.483	1722	0.03	-0.16	0.22	0.742	0.607
Total lipids in small HDL (mmol/l)	1722	0.03	-0.19	0.24	0.800	1722	-0.10	-0.35	0.14	0.417	1722	0.11	-0.08	0.29	0.247	0.547
Phospholipids in small HDL (mmol/l)	1722	0.10	-0.12	0.33	0.369	1722	-0.08	-0.33	0.18	0.553	1722	0.03	-0.17	0.23	0.759	0.716
Total cholesterol in small HDL (mmol/l)	1722	-0.09	-0.28	0.10	0.346	1722	-0.12	-0.35	0.11	0.300	1722	0.20	0.03	0.37	0.018	0.053
Cholesterol esters in small HDL (mmol/l)	1722	-0.10	-0.28	0.08	0.294	1722	-0.11	-0.34	0.11	0.312	1722	0.20	0.04	0.36	0.016	0.042
Free cholesterol in small HDL (mmol/l)	1722	-0.04	-0.27	0.19	0.725	1722	-0.11	-0.38	0.16	0.413	1722	0.15	-0.05	0.34	0.142	0.343
Triglycerides in small HDL (mmol/l)	1722	0.20	0.00	0.40	0.046	1722	0.06	-0.19	0.30	0.644	1722	-0.16	-0.35	0.03	0.090	0.058
Phospholipids to total lipds ratio in chylomicrons and extremely large VLDL (%)	1722	0.01	-0.08	0.10	0.768	1722	-0.03	-0.14	0.08	0.623	1722	0.07	-0.02	0.15	0.124	0.514
Total cholesterol to total lipids ratio in chylomicrons and extremely large VLDL (%)	1722	0.08	-0.11	0.27	0.388	1722	-0.05	-0.28	0.19	0.694	1722	0.00	-0.19	0.19	0.995	0.754
Cholesterol esters to total lipids ratio in chylomicrons and extremely large VLDL (%)	1722	0.07	-0.10	0.25	0.422	1722	-0.07	-0.30	0.15	0.528	1722	0.00	-0.20	0.19	0.980	0.693
Free cholesterol to total lipids ratio in chylomicrons and extremely large VLDL (%)	1722	0.11	-0.07	0.30	0.217	1722	0.04	-0.19	0.28	0.713	1722	-0.03	-0.20	0.14	0.739	0.563

Online Table 3 Associations of regional fat indexes at age 10y with cardiometabolic traits at age 18y in ALSPAC

At age 10y

	**Trunk fat index (per 1.1 kg/m^2 higher)** *Adj. for age, sex, ethnicity, maternal education, arm fat index, leg fat index*					**Arm fat index (per 0.2 kg/m^2 higher)** *Adj. for age, sex, ethnicity, maternal education, trunk fat index, leg fat index*					**Leg fat index (per 0.9 kg/m^2 higher)** *Adj. for age, sex, ethnicity, maternal education, trunk fat index, arm fat index*					
**Standardized outcome at age 18y**	**N**	**Beta**	**LCL**	**UCL**	**P-value**	**N**	**Beta**	**LCL**	**UCL**	**P-value**	**N**	**Beta**	**LCL**	**UCL**	**P-value**	***P-value for regional heterogeneity***
Triglycerides to total lipids ratio in chylomicrons and extremely large VLDL (%)	1722	-0.13	-0.33	0.07	0.200	1722	0.06	-0.15	0.27	0.581	1722	0.02	-0.16	0.20	0.809	0.543
Phospholipids to total lipds ratio in very large VLDL (%)	1722	0.20	0.03	0.37	0.025	1722	0.01	-0.22	0.23	0.949	1722	-0.08	-0.25	0.09	0.367	0.103
Total cholesterol to total lipids ratio in very large VLDL (%)	1722	-0.26	-0.55	0.03	0.076	1722	0.05	-0.19	0.29	0.665	1722	0.16	-0.03	0.34	0.098	0.139
Cholesterol esters to total lipids ratio in very large VLDL (%)	1722	-0.19	-0.40	0.02	0.075	1722	0.04	-0.15	0.24	0.670	1722	0.10	-0.04	0.24	0.178	0.168
Free cholesterol to total lipids ratio in very large VLDL (%)	1722	-0.13	-0.26	0.00	0.042	1722	-0.02	-0.18	0.13	0.784	1722	0.15	0.03	0.27	0.017	0.018
Triglycerides to total lipids ratio in very large VLDL (%)	1722	0.12	-0.06	0.30	0.198	1722	0.00	-0.22	0.23	0.991	1722	-0.11	-0.27	0.05	0.193	0.226
Phospholipids to total lipds ratio in large VLDL (%)	1722	0.21	0.02	0.39	0.026	1722	0.07	-0.18	0.31	0.583	1722	-0.18	-0.34	-0.01	0.041	0.008
Total cholesterol to total lipids ratio in large VLDL (%)	1722	0.06	-0.14	0.25	0.568	1722	0.10	-0.15	0.36	0.416	1722	-0.10	-0.28	0.08	0.261	0.388
Cholesterol esters to total lipids ratio in large VLDL (%)	1722	-0.16	-0.34	0.03	0.097	1722	0.13	-0.10	0.37	0.262	1722	0.01	-0.16	0.18	0.901	0.265
Free cholesterol to total lipids ratio in large VLDL (%)	1722	0.13	0.05	0.21	0.001	1722	0.01	-0.10	0.11	0.904	1722	-0.09	-0.16	-0.01	0.027	0.001
Triglycerides to total lipids ratio in large VLDL (%)	1722	-0.17	-0.38	0.04	0.105	1722	-0.05	-0.30	0.20	0.703	1722	0.15	-0.03	0.33	0.111	0.086
Phospholipids to total lipds ratio in medium VLDL (%)	1722	-0.33	-0.51	-0.14	0.001	1722	0.01	-0.22	0.25	0.907	1722	0.24	0.07	0.41	0.006	0.0002
Total cholesterol to total lipids ratio in medium VLDL (%)	1722	-0.02	-0.22	0.18	0.865	1722	0.11	-0.14	0.37	0.393	1722	-0.07	-0.25	0.11	0.434	0.650
Cholesterol esters to total lipids ratio in medium VLDL (%)	1722	-0.05	-0.24	0.15	0.632	1722	0.10	-0.15	0.34	0.427	1722	-0.05	-0.23	0.13	0.582	0.730
Free cholesterol to total lipids ratio in medium VLDL (%)	1722	0.09	-0.11	0.30	0.384	1722	0.09	-0.18	0.35	0.526	1722	-0.10	-0.28	0.07	0.245	0.298
Triglycerides to total lipids ratio in medium VLDL (%)	1722	0.08	-0.12	0.28	0.437	1722	-0.11	-0.36	0.15	0.405	1722	0.02	-0.16	0.20	0.806	0.683
Phospholipids to total lipds ratio in small VLDL (%)	1722	-0.30	-0.52	-0.08	0.008	1722	-0.12	-0.40	0.17	0.433	1722	0.30	0.10	0.50	0.003	0.0004
Total cholesterol to total lipids ratio in small VLDL (%)	1722	-0.11	-0.31	0.09	0.296	1722	0.09	-0.16	0.35	0.465	1722	-0.01	-0.19	0.17	0.913	0.610
Cholesterol esters to total lipids ratio in small VLDL (%)	1722	-0.06	-0.27	0.14	0.549	1722	0.10	-0.16	0.36	0.435	1722	-0.05	-0.23	0.13	0.588	0.724
Free cholesterol to total lipids ratio in small VLDL (%)	1722	-0.33	-0.54	-0.12	0.002	1722	-0.02	-0.27	0.23	0.890	1722	0.25	0.08	0.42	0.004	0.0003
Triglycerides to total lipids ratio in small VLDL (%)	1722	0.21	0.01	0.41	0.035	1722	-0.06	-0.30	0.18	0.645	1722	-0.09	-0.27	0.08	0.301	0.094
Phospholipids to total lipds ratio in very small VLDL (%)	1722	-0.17	-0.35	0.02	0.080	1722	0.02	-0.21	0.24	0.892	1722	0.15	-0.02	0.31	0.081	0.070
Total cholesterol to total lipids ratio in very small VLDL (%)	1722	0.04	-0.16	0.25	0.669	1722	0.00	-0.24	0.25	0.983	1722	-0.06	-0.23	0.11	0.479	0.756
Cholesterol esters to total lipids ratio in very small VLDL (%)	1722	0.13	-0.07	0.33	0.203	1722	0.02	-0.22	0.26	0.885	1722	-0.13	-0.30	0.04	0.131	0.178
Free cholesterol to total lipids ratio in very small VLDL (%)	1722	-0.26	-0.52	0.00	0.051	1722	-0.06	-0.32	0.20	0.666	1722	0.21	0.03	0.38	0.021	0.018
Triglycerides to total lipids ratio in very small VLDL (%)	1722	0.05	-0.15	0.26	0.603	1722	-0.01	-0.26	0.24	0.947	1722	-0.03	-0.20	0.15	0.771	0.859
Phospholipids to total lipds ratio in IDL (%)	1722	-0.23	-0.45	-0.01	0.040	1722	-0.18	-0.46	0.09	0.187	1722	0.32	0.14	0.51	0.001	0.0004
Total cholesterol to total lipids ratio in IDL (%)	1722	0.19	-0.02	0.39	0.073	1722	0.07	-0.20	0.33	0.620	1722	-0.18	-0.36	0.00	0.044	0.029
Cholesterol esters to total lipids ratio in IDL (%)	1722	0.33	0.12	0.53	0.002	1722	0.07	-0.19	0.34	0.587	1722	-0.29	-0.46	-0.12	0.001	<0.0001
Free cholesterol to total lipids ratio in IDL (%)	1722	-0.30	-0.49	-0.11	0.002	1722	-0.02	-0.26	0.23	0.902	1722	0.24	0.07	0.42	0.007	0.0004
Triglycerides to total lipids ratio in IDL (%)	1722	-0.13	-0.33	0.06	0.185	1722	0.00	-0.26	0.25	0.974	1722	0.09	-0.09	0.26	0.339	0.301
Phospholipids to total lipds ratio in large LDL (%)	1722	0.11	-0.08	0.30	0.263	1722	-0.16	-0.41	0.09	0.209	1722	0.00	-0.18	0.18	0.992	0.410
Total cholesterol to total lipids ratio in large LDL (%)	1722	0.03	-0.17	0.23	0.748	1722	0.13	-0.13	0.40	0.326	1722	-0.09	-0.28	0.10	0.347	0.477
Cholesterol esters to total lipids ratio in large LDL (%)	1722	0.08	-0.12	0.28	0.409	1722	0.14	-0.12	0.41	0.290	1722	-0.12	-0.31	0.07	0.212	0.238
Free cholesterol to total lipids ratio in large LDL (%)	1722	-0.17	-0.37	0.03	0.091	1722	-0.09	-0.35	0.16	0.480	1722	0.13	-0.05	0.31	0.164	0.096
Triglycerides to total lipids ratio in large LDL (%)	1722	-0.18	-0.38	0.02	0.073	1722	-0.03	-0.30	0.24	0.818	1722	0.14	-0.04	0.32	0.121	0.070
Phospholipids to total lipds ratio in medium LDL (%)	1722	0.10	-0.10	0.29	0.346	1722	-0.16	-0.41	0.09	0.207	1722	0.02	-0.16	0.20	0.843	0.479
Total cholesterol to total lipids ratio in medium LDL (%)	1722	-0.01	-0.20	0.19	0.957	1722	0.17	-0.09	0.43	0.210	1722	-0.09	-0.28	0.09	0.321	0.438
Cholesterol esters to total lipids ratio in medium LDL (%)	1722	0.00	-0.20	0.20	0.970	1722	0.16	-0.10	0.42	0.234	1722	-0.08	-0.27	0.11	0.407	0.503
Free cholesterol to total lipids ratio in medium LDL (%)	1722	-0.02	-0.22	0.18	0.863	1722	-0.12	-0.37	0.13	0.352	1722	0.05	-0.13	0.22	0.615	0.686
Triglycerides to total lipids ratio in medium LDL (%)	1722	-0.19	-0.37	-0.01	0.041	1722	-0.04	-0.28	0.21	0.772	1722	0.17	0.01	0.33	0.037	0.018
Phospholipids to total lipds ratio in small LDL (%)	1722	0.05	-0.14	0.25	0.610	1722	-0.16	-0.41	0.09	0.213	1722	0.04	-0.14	0.22	0.640	0.567
Total cholesterol to total lipids ratio in small LDL (%)	1722	-0.05	-0.24	0.15	0.635	1722	0.15	-0.11	0.41	0.270	1722	-0.05	-0.23	0.14	0.628	0.628
Cholesterol esters to total lipids ratio in small LDL (%)	1722	-0.02	-0.22	0.18	0.839	1722	0.13	-0.13	0.39	0.321	1722	-0.04	-0.22	0.15	0.707	0.717
Free cholesterol to total lipids ratio in small LDL (%)	1722	-0.03	-0.23	0.17	0.770	1722	-0.07	-0.32	0.17	0.570	1722	0.01	-0.17	0.18	0.919	0.902
Triglycerides to total lipids ratio in small LDL (%)	1722	0.00	-0.19	0.20	0.978	1722	0.00	-0.26	0.26	0.976	1722	0.02	-0.16	0.20	0.819	0.988
Phospholipids to total lipds ratio in very large HDL (%)	1722	-0.36	-0.54	-0.18	<0.0001	1722	-0.17	-0.39	0.05	0.131	1722	0.39	0.22	0.55	<0.0001	<0.0001
Total cholesterol to total lipids ratio in very large HDL (%)	1722	0.34	0.16	0.52	0.0002	1722	0.16	-0.06	0.37	0.153	1722	-0.36	-0.53	-0.20	<0.0001	<0.0001
Cholesterol esters to total lipids ratio in very large HDL (%)	1722	0.34	0.16	0.52	0.0002	1722	0.16	-0.06	0.37	0.154	1722	-0.37	-0.54	-0.20	<0.0001	<0.0001
Free cholesterol to total lipids ratio in very large HDL (%)	1722	-0.12	-0.32	0.08	0.244	1722	-0.07	-0.30	0.17	0.576	1722	0.19	0.00	0.38	0.050	0.110

Online Table 3 Associations of regional fat indexes at age 10y with cardiometabolic traits at age 18y in ALSPAC

At age 10y

	Trunk fat index (per 1.1 kg/m^2 higher) — *Adj. for age, sex, ethnicity, maternal education, arm fat index, leg fat index*					Arm fat index (per 0.2 kg/m^2 higher) — *Adj. for age, sex, ethnicity, maternal education, trunk fat index, leg fat index*					Leg fat index (per 0.9 kg/m^2 higher) — *Adj. for age, sex, ethnicity, maternal education, trunk fat index, arm fat index*					
**Standardized outcome at age 18y**	**N**	**Beta**	**LCL**	**UCL**	**P-value**	**N**	**Beta**	**LCL**	**UCL**	**P-value**	**N**	**Beta**	**LCL**	**UCL**	**P-value**	***P-value for regional heterogeneity***
Triglycerides to total lipids ratio in very large HDL (%)	1722	0.22	-0.01	0.45	0.062	1722	0.11	-0.16	0.39	0.429	1722	-0.24	-0.45	-0.02	0.031	0.033
Phospholipids to total lipds ratio in large HDL (%)	1722	0.36	0.15	0.58	0.001	1722	0.07	-0.18	0.32	0.573	1722	-0.24	-0.43	-0.06	0.009	0.0005
Total cholesterol to total lipids ratio in large HDL (%)	1722	-0.39	-0.61	-0.17	0.000	1722	-0.10	-0.36	0.16	0.463	1722	0.30	0.10	0.49	0.003	<0.0001
Cholesterol esters to total lipids ratio in large HDL (%)	1722	-0.39	-0.61	-0.16	0.001	1722	-0.11	-0.38	0.16	0.429	1722	0.30	0.10	0.50	0.003	0.0001
Free cholesterol to total lipids ratio in large HDL (%)	1722	-0.30	-0.50	-0.09	0.004	1722	-0.05	-0.27	0.18	0.688	1722	0.21	0.03	0.39	0.025	0.007
Triglycerides to total lipids ratio in large HDL (%)	1722	0.35	0.13	0.56	0.002	1722	0.14	-0.16	0.43	0.360	1722	-0.34	-0.55	-0.13	0.001	0.0001
Phospholipids to total lipds ratio in medium HDL (%)	1722	-0.16	-0.37	0.05	0.128	1722	-0.05	-0.30	0.20	0.683	1722	0.18	0.01	0.36	0.042	0.058
Total cholesterol to total lipids ratio in medium HDL (%)	1722	-0.01	-0.21	0.19	0.919	1722	0.01	-0.23	0.25	0.940	1722	-0.04	-0.22	0.14	0.647	0.956
Cholesterol esters to total lipids ratio in medium HDL (%)	1722	-0.02	-0.22	0.18	0.825	1722	0.01	-0.23	0.25	0.943	1722	-0.04	-0.22	0.14	0.697	0.973
Free cholesterol to total lipids ratio in medium HDL (%)	1722	0.06	-0.13	0.25	0.558	1722	-0.01	-0.21	0.20	0.939	1722	-0.02	-0.19	0.15	0.801	0.869
Triglycerides to total lipids ratio in medium HDL (%)	1722	0.31	0.08	0.54	0.008	1722	0.07	-0.20	0.33	0.627	1722	-0.23	-0.45	-0.02	0.036	0.011
Phospholipids to total lipds ratio in small HDL (%)	1722	0.16	-0.03	0.35	0.100	1722	0.08	-0.14	0.29	0.478	1722	-0.20	-0.36	-0.03	0.019	0.026
Total cholesterol to total lipids ratio in small HDL (%)	1722	-0.20	-0.39	-0.02	0.029	1722	-0.12	-0.33	0.10	0.294	1722	0.26	0.10	0.41	0.002	0.001
Cholesterol esters to total lipids ratio in small HDL (%)	1722	-0.16	-0.35	0.02	0.080	1722	-0.10	-0.32	0.11	0.359	1722	0.22	0.06	0.38	0.007	0.008
Free cholesterol to total lipids ratio in small HDL (%)	1722	-0.17	-0.39	0.06	0.142	1722	-0.05	-0.31	0.22	0.731	1722	0.10	-0.10	0.31	0.329	0.286
Triglycerides to total lipids ratio in small HDL (%)	1722	0.18	-0.03	0.39	0.099	1722	0.14	-0.13	0.40	0.324	1722	-0.22	-0.44	0.00	0.046	0.054
Mean diameter for VLDL particles (nm)	1722	0.45	0.25	0.66	<0.0001	1722	0.01	-0.23	0.25	0.960	1722	-0.30	-0.47	-0.12	0.001	<0.0001
Mean diameter for LDL particles (nm)	1722	-0.15	-0.35	0.05	0.142	1722	0.01	-0.21	0.23	0.949	1722	0.01	-0.15	0.18	0.882	0.519
Mean diameter for HDL particles (nm)	1722	-0.40	-0.58	-0.21	<0.0001	1722	-0.13	-0.35	0.09	0.255	1722	0.39	0.22	0.56	<0.0001	<0.0001
Serum total cholesterol (mmol/l)	1722	0.03	-0.17	0.24	0.743	1722	-0.01	-0.27	0.24	0.924	1722	0.03	-0.15	0.22	0.721	0.972
Total cholesterol in VLDL (mmol/l)	1722	0.36	0.14	0.57	0.001	1722	0.07	-0.21	0.35	0.635	1722	-0.28	-0.48	-0.07	0.007	0.0003
Remnant cholesterol (non-HDL, non-LDL -cholesterol) (mmol/l)	1722	0.26	0.04	0.48	0.022	1722	0.06	-0.23	0.34	0.699	1722	-0.19	-0.40	0.01	0.064	0.018
Total cholesterol in LDL (mmol/l)	1722	0.06	-0.15	0.28	0.547	1722	0.02	-0.24	0.29	0.856	1722	-0.02	-0.22	0.17	0.807	0.843
Total cholesterol in HDL (mmol/l)	1722	-0.31	-0.50	-0.11	0.002	1722	-0.14	-0.37	0.09	0.238	1722	0.37	0.19	0.54	<0.0001	<0.0001
Total cholesterol in HDL2 (mmol/l)	1722	-0.33	-0.53	-0.14	0.001	1722	-0.14	-0.38	0.10	0.237	1722	0.38	0.21	0.56	<0.0001	<0.0001
Total cholesterol in HDL3 (mmol/l)	1722	-0.24	-0.43	-0.05	0.013	1722	-0.13	-0.35	0.10	0.273	1722	0.32	0.15	0.49	0.0002	0.0002
Esterified cholesterol (mmol/l)	1722	0.05	-0.15	0.26	0.616	1722	-0.01	-0.27	0.25	0.942	1722	0.01	-0.18	0.20	0.932	0.941
Free cholesterol (mmol/l)	1722	-0.01	-0.21	0.18	0.897	1722	-0.02	-0.26	0.22	0.888	1722	0.09	-0.09	0.27	0.325	0.726
Serum total triglycerides (mmol/l)	1722	0.32	0.12	0.53	0.002	1722	0.07	-0.19	0.34	0.597	1722	-0.25	-0.44	-0.05	0.013	0.001
Triglycerides in VLDL (mmol/l)	1722	0.39	0.19	0.60	0.0002	1722	0.07	-0.19	0.34	0.597	1722	-0.30	-0.49	-0.11	0.002	<0.0001
Triglycerides in LDL (mmol/l)	1722	-0.09	-0.29	0.10	0.341	1722	0.04	-0.20	0.28	0.759	1722	0.07	-0.11	0.25	0.459	0.528
Triglycerides in HDL (mmol/l)	1722	0.16	-0.04	0.36	0.107	1722	0.00	-0.24	0.24	0.992	1722	-0.09	-0.28	0.10	0.373	0.269
Diacylglycerol (mmol/l)	1722	0.24	0.02	0.45	0.032	1722	-0.08	-0.32	0.16	0.515	1722	-0.05	-0.22	0.12	0.583	0.146
Ratio of diacylglycerol to triglycerides	1722	0.08	-0.11	0.28	0.404	1722	-0.06	-0.29	0.17	0.605	1722	0.05	-0.13	0.22	0.596	0.767
Total phosphoglycerides (mmol/l)	1722	-0.12	-0.31	0.08	0.239	1722	-0.01	-0.25	0.23	0.946	1722	0.13	-0.05	0.32	0.156	0.232
Ratio of triglycerides to phosphoglycerides	1722	0.42	0.19	0.64	0.0002	1722	0.03	-0.25	0.30	0.859	1722	-0.29	-0.48	-0.11	0.002	<0.0001
Phosphatidylcholine and other cholines (mmol/l)	1722	-0.09	-0.28	0.11	0.372	1722	-0.07	-0.30	0.16	0.562	1722	0.15	-0.02	0.33	0.088	0.196
Total cholines (mmol/l)	1722	-0.14	-0.34	0.06	0.160	1722	-0.02	-0.25	0.22	0.884	1722	0.15	-0.02	0.33	0.092	0.125
Apolipoprotein A-I (g/l)	1722	-0.19	-0.37	0.00	0.049	1722	-0.12	-0.34	0.10	0.292	1722	0.27	0.11	0.44	0.001	0.002
Apolipoprotein B (g/l)	1722	0.27	0.05	0.49	0.016	1722	0.07	-0.21	0.35	0.618	1722	-0.21	-0.42	-0.01	0.044	0.011
Ratio of apolipoprotein B to apolipoprotein A-I	1722	0.34	0.12	0.56	0.003	1722	0.14	-0.15	0.43	0.349	1722	-0.33	-0.54	-0.12	0.002	0.0002
Total fatty acids (mmol/l)	1722	0.10	-0.09	0.30	0.294	1722	0.02	-0.23	0.27	0.873	1722	-0.04	-0.23	0.15	0.681	0.623
Estimated description of fatty acid chain length, not actual carbon number	1722	0.06	-0.12	0.24	0.522	1722	-0.10	-0.33	0.13	0.414	1722	0.05	-0.12	0.21	0.560	0.698
Estimated degree of unsaturation	1722	-0.05	-0.24	0.13	0.562	1722	-0.12	-0.35	0.10	0.278	1722	0.15	-0.03	0.33	0.113	0.230
22:6, docosahexaenoic acid (mmol/l)	1722	0.03	-0.14	0.21	0.704	1722	-0.04	-0.26	0.17	0.697	1722	0.02	-0.16	0.19	0.866	0.910
18:2, linoleic acid (mmol/l)	1722	0.06	-0.13	0.25	0.517	1722	-0.07	-0.31	0.18	0.589	1722	0.04	-0.14	0.22	0.688	0.813
Conjugated linoleic acid (mmol/l)	1722	0.16	-0.02	0.34	0.078	1722	-0.14	-0.34	0.07	0.202	1722	0.04	-0.12	0.20	0.596	0.256
Omega-3 fatty acids (mmol/l)	1722	0.13	-0.05	0.32	0.164	1722	-0.03	-0.27	0.21	0.835	1722	-0.06	-0.25	0.14	0.577	0.420
Omega-6 fatty acids (mmol/l)	1722	0.05	-0.14	0.25	0.591	1722	-0.04	-0.29	0.21	0.751	1722	0.03	-0.15	0.22	0.725	0.902
Polyunsaturated fatty acids (mmol/l)	1722	0.07	-0.13	0.26	0.499	1722	-0.04	-0.29	0.21	0.751	1722	0.02	-0.17	0.21	0.816	0.867

Online Table 3 Associations of regional fat indexes at age 10y with cardiometabolic traits at age 18y in ALSPAC

At age 10y

	Trunk fat index (per 1.1 kg/m^2 higher)					Arm fat index (per 0.2 kg/m^2 higher)					Leg fat index (per 0.9 kg/m^2 higher)					
	Adj. for age, sex, ethnicity, maternal education, arm fat index, leg fat index					*Adj. for age, sex, ethnicity, maternal education, trunk fat index, leg fat index*					*Adj. for age, sex, ethnicity, maternal education, trunk fat index, arm fat index*					***P-value for regional heterogeneity***
**Standardized outcome at age 18y**	**N**	**Beta**	**LCL**	**UCL**	**P-value**	**N**	**Beta**	**LCL**	**UCL**	**P-value**	**N**	**Beta**	**LCL**	**UCL**	**P-value**	
Monounsaturated fatty acids; 16:1, 18:1 (mmol/l)	1722	0.14	-0.05	0.33	0.157	1722	0.05	-0.21	0.31	0.699	1722	-0.08	-0.27	0.11	0.403	0.312
Saturated fatty acids (mmol/l)	1722	0.09	-0.11	0.28	0.398	1722	0.04	-0.21	0.29	0.749	1722	-0.05	-0.24	0.15	0.627	0.681
Ratio of 22:6 docosahexaenoic acid to total fatty acids (%)	1722	0.02	-0.17	0.20	0.874	1722	-0.12	-0.34	0.10	0.292	1722	0.07	-0.09	0.24	0.374	0.536
Ratio of 18:2 linoleic acid to total fatty acids (%)	1722	-0.09	-0.29	0.10	0.336	1722	-0.11	-0.32	0.10	0.299	1722	0.10	-0.07	0.27	0.250	0.284
Ratio of conjugated linoleic acid to total fatty acids (%)	1722	0.17	0.01	0.33	0.040	1722	-0.17	-0.36	0.02	0.073	1722	0.06	-0.08	0.20	0.423	0.111
Ratio of omega-3 fatty acids to total fatty acids (%)	1722	0.12	-0.08	0.31	0.237	1722	-0.10	-0.34	0.15	0.446	1722	-0.03	-0.22	0.17	0.768	0.469
Ratio of omega-6 fatty acids to total fatty acids (%)	1722	-0.14	-0.33	0.04	0.135	1722	-0.09	-0.31	0.14	0.444	1722	0.13	-0.05	0.30	0.154	0.148
Ratio of polyunsaturated fatty acids to total fatty acids (%)	1722	-0.11	-0.30	0.08	0.259	1722	-0.11	-0.33	0.12	0.359	1722	0.11	-0.07	0.29	0.213	0.249
Ratio of monounsaturated fatty acids to total fatty acids (%)	1722	0.14	-0.05	0.33	0.145	1722	0.07	-0.18	0.32	0.581	1722	-0.11	-0.28	0.07	0.223	0.160
Ratio of saturated fatty acids to total fatty acids (%)	1722	-0.06	-0.25	0.13	0.524	1722	0.03	-0.21	0.28	0.795	1722	0.01	-0.18	0.19	0.929	0.846
Insulin (mu/l)	1722	0.30	0.15	0.44	<0.0001	1722	0.01	-0.17	0.19	0.899	1722	-0.14	-0.29	0.01	0.072	0.001
Glucose (mmol/l)	1722	0.13	-0.02	0.27	0.085	1722	0.00	-0.18	0.18	0.996	1722	-0.09	-0.24	0.06	0.236	0.147
Lactate (mmol/l)	1722	-0.09	-0.28	0.10	0.330	1722	0.15	-0.10	0.40	0.237	1722	-0.01	-0.21	0.19	0.917	0.463
Pyruvate (mmol/l)	1722	0.02	-0.15	0.19	0.830	1722	0.15	-0.09	0.39	0.224	1722	-0.06	-0.24	0.13	0.561	0.590
Citrate (mmol/l)	1722	-0.09	-0.27	0.09	0.343	1722	0.04	-0.18	0.26	0.696	1722	-0.09	-0.26	0.08	0.317	0.737
Alanine (mmol/l)	1722	0.18	-0.01	0.38	0.063	1722	0.00	-0.24	0.23	0.969	1722	-0.12	-0.30	0.06	0.194	0.111
Glutamine (mmol/l)	1722	0.18	0.01	0.35	0.035	1722	-0.17	-0.38	0.04	0.120	1722	-0.05	-0.21	0.11	0.542	0.073
Histidine (mmol/l)	1722	0.17	-0.03	0.37	0.094	1722	-0.08	-0.33	0.17	0.521	1722	-0.07	-0.25	0.12	0.476	0.233
Isoleucine (mmol/l)	1722	0.30	0.12	0.48	0.001	1722	0.12	-0.10	0.35	0.291	1722	-0.30	-0.47	-0.14	0.0003	<0.0001
Leucine (mmol/l)	1722	0.25	0.09	0.42	0.003	1722	0.05	-0.15	0.24	0.647	1722	-0.22	-0.36	-0.08	0.002	0.0003
Valine (mmol/l)	1722	0.27	0.09	0.44	0.003	1722	0.15	-0.06	0.36	0.172	1722	-0.28	-0.43	-0.13	0.0003	<0.0001
Phenylalanine (mmol/l)	1722	0.23	0.04	0.43	0.020	1722	-0.18	-0.41	0.05	0.133	1722	0.06	-0.11	0.23	0.485	0.116
Tyrosine (mmol/l)	1722	0.30	0.11	0.48	0.002	1722	-0.10	-0.33	0.13	0.380	1722	-0.05	-0.23	0.13	0.601	0.021
Acetate (mmol/l)	1722	-0.07	-0.14	0.00	0.044	1722	0.00	-0.09	0.10	0.970	1722	0.02	-0.06	0.09	0.671	0.239
Acetoacetate (mmol/l)	1722	-0.13	-0.30	0.04	0.133	1722	0.01	-0.28	0.29	0.971	1722	0.14	-0.07	0.35	0.196	0.100
3-hydroxybutyrate (mmol/l)	1722	-0.23	-0.40	-0.05	0.012	1722	0.08	-0.17	0.33	0.548	1722	0.14	-0.04	0.33	0.135	0.019
Creatinine (mmol/l)	1722	0.08	-0.07	0.23	0.294	1722	-0.06	-0.25	0.13	0.506	1722	0.00	-0.15	0.15	0.994	0.605
Albumin (signal area)	1722	-0.03	-0.21	0.15	0.757	1722	-0.01	-0.24	0.22	0.933	1722	-0.03	-0.21	0.15	0.722	0.993
Glycoprotein acetyls, mainly a1-acid glycoprotein (mmol/l)	1722	0.29	0.08	0.49	0.006	1722	-0.07	-0.30	0.16	0.553	1722	-0.05	-0.22	0.13	0.622	0.064
C-reactive protein (mg/l)	1722	0.01	-0.16	0.18	0.930	1722	-0.02	-0.24	0.20	0.857	1722	0.07	-0.12	0.26	0.482	0.868

Online Table 4 Associations of regional fat indexes at age 18y with cardiometabolic traits at age 18y in ALSPAC

At age 18y

	**Trunk fat index (per 2.0 kg/m^2 higher)** *Adj. for age, sex, ethnicity, maternal education, smoking, alcohol, puberty timing, arm fat index, leg fat index*					**Arm fat index (per 0.3 kg/m^2 higher)** *Adj. for age, sex, ethnicity, maternal education, smoking, alcohol, puberty timing, trunk fat index, leg fat index*					**Leg fat index (per 1.3 kg/m^2 higher)** *Adj. for age, sex, ethnicity, maternal education, smoking, alcohol, puberty timing, trunk fat index, arm fat index*					
**Standardized outcome at age 18y**	**N**	**Beta**	**LCL**	**UCL**	**P-value**	**N**	**Beta**	**LCL**	**UCL**	**P-value**	**N**	**Beta**	**LCL**	**UCL**	**P-value**	***P-value for regional heterogeneity***
Systolic blood pressure (mmHg)	3016	0.25	0.15	0.36	<0.0001	3016	0.03	-0.09	0.16	0.590	3016	-0.02	-0.15	0.10	0.707	0.009
Diastolic blood pressure (mmHg)	3016	0.28	0.15	0.40	<0.0001	3016	0.11	-0.03	0.24	0.136	3016	-0.03	-0.17	0.10	0.628	0.018
Concentration of chylomicrons and extremely large VLDL particles (mol/l)	2058	0.66	0.48	0.83	<0.0001	2058	0.12	-0.13	0.37	0.358	2058	-0.42	-0.64	-0.20	0.0002	<0.0001
Total lipids in chylomicrons and extremely large VLDL (mmol/l)	2058	0.66	0.48	0.83	<0.0001	2058	0.11	-0.13	0.36	0.357	2058	-0.42	-0.64	-0.21	<0.0001	<0.0001
Phospholipids in chylomicrons and extremely large VLDL (mmol/l)	2058	0.65	0.47	0.82	<0.0001	2058	0.11	-0.13	0.36	0.357	2058	-0.42	-0.63	-0.21	<0.0001	<0.0001
Total cholesterol in chylomicrons and extremely large VLDL (mmol/l)	2058	0.64	0.46	0.81	<0.0001	2058	0.09	-0.15	0.33	0.479	2058	-0.38	-0.60	-0.17	0.0005	<0.0001
Cholesterol esters in chylomicrons and extremely large VLDL (mmol/l)	2058	0.62	0.44	0.79	<0.0001	2058	0.07	-0.17	0.30	0.590	2058	-0.35	-0.57	-0.13	0.002	<0.0001
Free cholesterol in chylomicrons and extremely large VLDL (mmol/l)	2058	0.64	0.47	0.82	<0.0001	2058	0.11	-0.13	0.35	0.377	2058	-0.41	-0.63	-0.20	0.0001	<0.0001
Triglycerides in chylomicrons and extremely large VLDL (mmol/l)	2058	0.66	0.48	0.83	<0.0001	2058	0.12	-0.12	0.36	0.333	2058	-0.43	-0.64	-0.22	<0.0001	<0.0001
Concentration of very large VLDL particles (mol/l)	2058	0.67	0.50	0.85	<0.0001	2058	0.09	-0.15	0.33	0.446	2058	-0.43	-0.64	-0.22	<0.0001	<0.0001
Total lipids in very large VLDL (mmol/l)	2058	0.68	0.50	0.85	<0.0001	2058	0.09	-0.15	0.32	0.478	2058	-0.43	-0.64	-0.22	<0.0001	<0.0001
Phospholipids in very large VLDL (mmol/l)	2058	0.66	0.48	0.84	<0.0001	2058	0.09	-0.15	0.33	0.480	2058	-0.41	-0.62	-0.20	0.0001	<0.0001
Total cholesterol in very large VLDL (mmol/l)	2058	0.67	0.49	0.84	<0.0001	2058	0.09	-0.14	0.33	0.441	2058	-0.41	-0.62	-0.20	0.0002	<0.0001
Cholesterol esters in very large VLDL (mmol/l)	2058	0.68	0.51	0.85	<0.0001	2058	0.09	-0.14	0.32	0.453	2058	-0.42	-0.63	-0.20	0.0001	<0.0001
Free cholesterol in very large VLDL (mmol/l)	2058	0.65	0.47	0.83	<0.0001	2058	0.10	-0.15	0.34	0.429	2058	-0.40	-0.62	-0.19	0.0002	<0.0001
Triglycerides in very large VLDL (mmol/l)	2058	0.68	0.51	0.85	<0.0001	2058	0.08	-0.15	0.31	0.494	2058	-0.44	-0.64	-0.23	<0.0001	<0.0001
Concentration of large VLDL particles (mol/l)	2058	0.69	0.52	0.86	<0.0001	2058	0.06	-0.16	0.28	0.600	2058	-0.43	-0.63	-0.23	<0.0001	<0.0001
Total lipids in large VLDL (mmol/l)	2058	0.69	0.52	0.87	<0.0001	2058	0.06	-0.16	0.28	0.608	2058	-0.43	-0.63	-0.23	<0.0001	<0.0001
Phospholipids in large VLDL (mmol/l)	2058	0.68	0.51	0.86	<0.0001	2058	0.05	-0.17	0.28	0.643	2058	-0.42	-0.62	-0.22	<0.0001	<0.0001
Total cholesterol in large VLDL (mmol/l)	2058	0.68	0.51	0.86	<0.0001	2058	0.05	-0.17	0.28	0.654	2058	-0.40	-0.61	-0.20	<0.0001	<0.0001
Cholesterol esters in large VLDL (mmol/l)	2058	0.68	0.51	0.86	<0.0001	2058	0.04	-0.18	0.27	0.689	2058	-0.39	-0.59	-0.19	0.0001	<0.0001
Free cholesterol in large VLDL (mmol/l)	2058	0.68	0.50	0.85	<0.0001	2058	0.06	-0.17	0.29	0.624	2058	-0.41	-0.62	-0.21	<0.0001	<0.0001
Triglycerides in large VLDL (mmol/l)	2058	0.69	0.52	0.87	<0.0001	2058	0.06	-0.16	0.28	0.581	2058	-0.44	-0.64	-0.24	<0.0001	<0.0001
Concentration of medium VLDL particles (mol/l)	2058	0.71	0.54	0.88	<0.0001	2058	0.05	-0.16	0.27	0.635	2058	-0.43	-0.63	-0.24	<0.0001	<0.0001
Total lipids in medium VLDL (mmol/l)	2058	0.71	0.53	0.88	<0.0001	2058	0.05	-0.17	0.26	0.658	2058	-0.42	-0.62	-0.23	<0.0001	<0.0001
Phospholipids in medium VLDL (mmol/l)	2058	0.69	0.51	0.86	<0.0001	2058	0.04	-0.17	0.26	0.686	2058	-0.41	-0.60	-0.21	<0.0001	<0.0001
Total cholesterol in medium VLDL (mmol/l)	2058	0.65	0.47	0.82	<0.0001	2058	0.03	-0.18	0.25	0.777	2058	-0.35	-0.55	-0.15	0.001	<0.0001
Cholesterol esters in medium VLDL (mmol/l)	2058	0.60	0.42	0.78	<0.0001	2058	0.02	-0.20	0.23	0.865	2058	-0.30	-0.50	-0.10	0.003	<0.0001
Free cholesterol in medium VLDL (mmol/l)	2058	0.67	0.49	0.84	<0.0001	2058	0.04	-0.17	0.26	0.687	2058	-0.40	-0.59	-0.20	<0.0001	<0.0001
Triglycerides in medium VLDL (mmol/l)	2058	0.72	0.55	0.89	<0.0001	2058	0.06	-0.16	0.27	0.599	2058	-0.45	-0.64	-0.26	<0.0001	<0.0001
Concentration of small VLDL particles (mol/l)	2058	0.64	0.47	0.81	<0.0001	2058	0.03	-0.18	0.24	0.800	2058	-0.37	-0.56	-0.18	0.0002	<0.0001
Total lipids in small VLDL (mmol/l)	2058	0.63	0.45	0.80	<0.0001	2058	0.03	-0.18	0.24	0.751	2058	-0.35	-0.54	-0.16	0.0003	<0.0001
Phospholipids in small VLDL (mmol/l)	2058	0.59	0.42	0.76	<0.0001	2058	0.00	-0.20	0.21	0.974	2058	-0.31	-0.50	-0.13	0.001	<0.0001
Total cholesterol in small VLDL (mmol/l)	2058	0.53	0.35	0.70	<0.0001	2058	0.05	-0.16	0.27	0.611	2058	-0.28	-0.46	-0.09	0.004	<0.0001
Cholesterol esters in small VLDL (mmol/l)	2058	0.48	0.31	0.66	<0.0001	2058	0.07	-0.14	0.29	0.495	2058	-0.25	-0.44	-0.07	0.007	<0.0001
Free cholesterol in small VLDL (mmol/l)	2058	0.55	0.38	0.73	<0.0001	2058	0.01	-0.20	0.22	0.916	2058	-0.29	-0.48	-0.10	0.002	<0.0001
Triglycerides in small VLDL (mmol/l)	2058	0.65	0.47	0.82	<0.0001	2058	0.02	-0.19	0.24	0.826	2058	-0.39	-0.58	-0.19	<0.0001	<0.0001
Concentration of very small VLDL particles (mol/l)	2058	0.25	0.08	0.43	0.005	2058	0.00	-0.20	0.21	0.963	2058	-0.07	-0.26	0.11	0.445	0.088
Total lipids in very small VLDL (mmol/l)	2058	0.30	0.12	0.48	0.001	2058	0.04	-0.17	0.25	0.700	2058	-0.12	-0.31	0.06	0.198	0.019
Phospholipids in very small VLDL (mmol/l)	2058	0.17	-0.01	0.34	0.062	2058	0.01	-0.20	0.21	0.958	2058	-0.01	-0.19	0.17	0.928	0.450
Total cholesterol in very small VLDL (mmol/l)	2058	0.27	0.09	0.45	0.004	2058	0.08	-0.14	0.29	0.474	2058	-0.13	-0.31	0.05	0.152	0.025
Cholesterol esters in very small VLDL (mmol/l)	2058	0.32	0.15	0.50	0.0004	2058	0.10	-0.11	0.31	0.359	2058	-0.18	-0.36	0.00	0.049	0.002
Free cholesterol in very small VLDL (mmol/l)	2058	0.11	-0.08	0.30	0.269	2058	0.02	-0.19	0.23	0.853	2058	0.00	-0.18	0.18	0.989	0.767
Triglycerides in very small VLDL (mmol/l)	2058	0.38	0.21	0.56	<0.0001	2058	-0.02	-0.23	0.20	0.890	2058	-0.18	-0.37	0.00	0.055	0.001
Concentration of IDL particles (mol/l)	2058	0.08	-0.09	0.26	0.357	2058	0.02	-0.18	0.23	0.835	2058	0.02	-0.16	0.20	0.806	0.904
Total lipids in IDL (mmol/l)	2058	0.10	-0.08	0.27	0.285	2058	0.02	-0.18	0.23	0.825	2058	0.03	-0.15	0.21	0.768	0.873
Phospholipids in IDL (mmol/l)	2058	0.04	-0.14	0.21	0.674	2058	0.01	-0.19	0.22	0.894	2058	0.07	-0.11	0.24	0.461	0.950
Total cholesterol in IDL (mmol/l)	2058	0.13	-0.05	0.30	0.156	2058	0.04	-0.17	0.24	0.730	2058	0.00	-0.18	0.18	0.975	0.687
Cholesterol esters in IDL (mmol/l)	2058	0.19	0.01	0.36	0.040	2058	0.05	-0.16	0.25	0.671	2058	-0.04	-0.22	0.15	0.695	0.321

Online Table 4 Associations of regional fat indexes at age 18y with cardiometabolic traits at age 18y in ALSPAC

At age 18y

	**Trunk fat index (per 2.0 kg/m^2 higher)** *Adj. for age, sex, ethnicity, maternal education, smoking, alcohol, puberty timing, arm fat index, leg fat index*					**Arm fat index (per 0.3 kg/m^2 higher)** *Adj. for age, sex, ethnicity, maternal education, smoking, alcohol, puberty timing, trunk fat index, leg fat index*					**Leg fat index (per 1.3 kg/m^2 higher)** *Adj. for age, sex, ethnicity, maternal education, smoking, alcohol, puberty timing, trunk fat index, arm fat index*					
**Standardized outcome at age 18y**	**N**	**Beta**	**LCL**	**UCL**	**P-value**	**N**	**Beta**	**LCL**	**UCL**	**P-value**	**N**	**Beta**	**LCL**	**UCL**	**P-value**	***P-value for regional heterogeneity***
Free cholesterol in IDL (mmol/l)	2058	-0.02	-0.19	0.16	0.841	2058	0.01	-0.19	0.22	0.892	2058	0.10	-0.08	0.27	0.270	0.704
Triglycerides in IDL (mmol/l)	2058	0.00	-0.17	0.17	0.985	2058	-0.03	-0.24	0.17	0.743	2058	0.06	-0.11	0.24	0.474	0.825
Concentration of large LDL particles (mol/l)	2058	0.09	-0.09	0.26	0.326	2058	0.02	-0.18	0.22	0.856	2058	0.03	-0.14	0.20	0.724	0.899
Total lipids in large LDL (mmol/l)	2058	0.09	-0.08	0.26	0.319	2058	0.02	-0.18	0.22	0.837	2058	0.04	-0.14	0.21	0.683	0.909
Phospholipids in large LDL (mmol/l)	2058	0.13	-0.04	0.30	0.147	2058	0.02	-0.18	0.22	0.838	2058	0.02	-0.16	0.19	0.842	0.709
Total cholesterol in large LDL (mmol/l)	2058	0.09	-0.08	0.27	0.280	2058	0.03	-0.18	0.23	0.795	2058	0.03	-0.14	0.21	0.722	0.884
Cholesterol esters in large LDL (mmol/l)	2058	0.12	-0.05	0.29	0.163	2058	0.03	-0.17	0.23	0.781	2058	0.01	-0.16	0.19	0.869	0.732
Free cholesterol in large LDL (mmol/l)	2058	0.01	-0.16	0.18	0.921	2058	0.02	-0.18	0.22	0.840	2058	0.08	-0.09	0.26	0.343	0.853
Triglycerides in large LDL (mmol/l)	2058	-0.07	-0.24	0.10	0.434	2058	-0.02	-0.22	0.18	0.807	2058	0.10	-0.08	0.27	0.274	0.504
Concentration of medium LDL particles (mol/l)	2058	0.14	-0.04	0.32	0.122	2058	0.02	-0.19	0.22	0.859	2058	0.00	-0.17	0.17	0.981	0.595
Total lipids in medium LDL (mmol/l)	2058	0.12	-0.05	0.29	0.163	2058	0.02	-0.18	0.23	0.827	2058	0.01	-0.16	0.18	0.893	0.713
Phospholipids in medium LDL (mmol/l)	2058	0.20	0.03	0.36	0.021	2058	0.03	-0.17	0.23	0.748	2058	-0.03	-0.20	0.14	0.731	0.252
Total cholesterol in medium LDL (mmol/l)	2058	0.12	-0.05	0.30	0.165	2058	0.03	-0.18	0.23	0.806	2058	0.01	-0.16	0.18	0.904	0.715
Cholesterol esters in medium LDL (mmol/l)	2058	0.13	-0.04	0.31	0.144	2058	0.02	-0.18	0.23	0.838	2058	0.01	-0.17	0.18	0.924	0.674
Free cholesterol in medium LDL (mmol/l)	2058	0.09	-0.08	0.26	0.305	2058	0.04	-0.16	0.25	0.671	2058	0.02	-0.15	0.19	0.821	0.883
Triglycerides in medium LDL (mmol/l)	2058	-0.07	-0.26	0.11	0.418	2058	-0.03	-0.23	0.17	0.767	2058	0.11	-0.06	0.27	0.218	0.430
Concentration of small LDL particles (mol/l)	2058	0.15	-0.03	0.32	0.103	2058	0.03	-0.17	0.24	0.756	2058	-0.02	-0.19	0.15	0.784	0.487
Total lipids in small LDL (mmol/l)	2058	0.14	-0.03	0.31	0.111	2058	0.03	-0.17	0.23	0.774	2058	-0.01	-0.18	0.16	0.938	0.570
Phospholipids in small LDL (mmol/l)	2058	0.19	0.02	0.35	0.027	2058	0.04	-0.16	0.23	0.713	2058	-0.05	-0.22	0.12	0.599	0.236
Total cholesterol in small LDL (mmol/l)	2058	0.12	-0.06	0.29	0.187	2058	0.03	-0.17	0.24	0.761	2058	0.01	-0.16	0.18	0.914	0.740
Cholesterol esters in small LDL (mmol/l)	2058	0.12	-0.06	0.29	0.189	2058	0.03	-0.18	0.23	0.810	2058	0.01	-0.16	0.19	0.877	0.749
Free cholesterol in small LDL (mmol/l)	2058	0.10	-0.07	0.27	0.231	2058	0.06	-0.14	0.26	0.576	2058	-0.01	-0.18	0.16	0.916	0.709
Triglycerides in small LDL (mmol/l)	2058	0.16	-0.02	0.35	0.084	2058	-0.01	-0.23	0.20	0.893	2058	-0.04	-0.22	0.14	0.659	0.380
Concentration of very large HDL particles (mol/l)	2058	-0.57	-0.72	-0.42	<0.0001	2058	0.01	-0.16	0.17	0.948	2058	0.36	0.21	0.52	<0.0001	<0.0001
Total lipids in very large HDL (mmol/l)	2058	-0.55	-0.71	-0.40	<0.0001	2058	0.03	-0.14	0.20	0.700	2058	0.33	0.17	0.49	<0.0001	<0.0001
Phospholipids in very large HDL (mmol/l)	2058	-0.60	-0.75	-0.46	<0.0001	2058	-0.03	-0.19	0.14	0.736	2058	0.41	0.26	0.56	<0.0001	<0.0001
Total cholesterol in very large HDL (mmol/l)	2058	-0.46	-0.63	-0.30	<0.0001	2058	0.10	-0.08	0.27	0.296	2058	0.22	0.06	0.38	0.007	<0.0001
Cholesterol esters in very large HDL (mmol/l)	2058	-0.43	-0.59	-0.26	<0.0001	2058	0.12	-0.07	0.30	0.210	2058	0.18	0.02	0.34	0.028	<0.0001
Free cholesterol in very large HDL (mmol/l)	2058	-0.53	-0.69	-0.38	<0.0001	2058	0.04	-0.14	0.21	0.686	2058	0.32	0.16	0.47	<0.0001	<0.0001
Triglycerides in very large HDL (mmol/l)	2058	-0.07	-0.23	0.10	0.417	2058	0.04	-0.17	0.25	0.717	2058	0.04	-0.15	0.23	0.693	0.711
Concentration of large HDL particles (mol/l)	2058	-0.59	-0.74	-0.44	<0.0001	2058	-0.09	-0.26	0.08	0.322	2058	0.42	0.26	0.59	<0.0001	<0.0001
Total lipids in large HDL (mmol/l)	2058	-0.59	-0.74	-0.44	<0.0001	2058	-0.09	-0.26	0.09	0.324	2058	0.42	0.26	0.59	<0.0001	<0.0001
Phospholipids in large HDL (mmol/l)	2058	-0.55	-0.69	-0.40	<0.0001	2058	-0.09	-0.26	0.08	0.295	2058	0.41	0.25	0.57	<0.0001	<0.0001
Total cholesterol in large HDL (mmol/l)	2058	-0.63	-0.78	-0.48	<0.0001	2058	-0.08	-0.25	0.10	0.372	2058	0.43	0.27	0.60	<0.0001	<0.0001
Cholesterol esters in large HDL (mmol/l)	2058	-0.63	-0.78	-0.48	<0.0001	2058	-0.08	-0.26	0.09	0.367	2058	0.43	0.27	0.60	<0.0001	<0.0001
Free cholesterol in large HDL (mmol/l)	2058	-0.62	-0.77	-0.47	<0.0001	2058	-0.08	-0.25	0.10	0.390	2058	0.43	0.27	0.59	<0.0001	<0.0001
Triglycerides in large HDL (mmol/l)	2058	-0.04	-0.20	0.12	0.597	2058	-0.14	-0.32	0.04	0.135	2058	0.15	-0.02	0.32	0.075	0.142
Concentration of medium HDL particles (mol/l)	2058	-0.13	-0.31	0.04	0.140	2058	-0.11	-0.30	0.07	0.230	2058	0.19	0.01	0.37	0.040	0.072
Total lipids in medium HDL (mmol/l)	2058	-0.17	-0.34	0.01	0.058	2058	-0.11	-0.30	0.08	0.249	2058	0.21	0.02	0.39	0.026	0.037
Phospholipids in medium HDL (mmol/l)	2058	-0.20	-0.37	-0.02	0.029	2058	-0.10	-0.28	0.08	0.290	2058	0.22	0.03	0.40	0.022	0.025
Total cholesterol in medium HDL (mmol/l)	2058	-0.21	-0.38	-0.04	0.015	2058	-0.11	-0.31	0.09	0.272	2058	0.23	0.04	0.41	0.016	0.012
Cholesterol esters in medium HDL (mmol/l)	2058	-0.22	-0.39	-0.05	0.011	2058	-0.11	-0.31	0.09	0.283	2058	0.23	0.04	0.42	0.016	0.011
Free cholesterol in medium HDL (mmol/l)	2058	-0.15	-0.32	0.02	0.077	2058	-0.12	-0.31	0.07	0.219	2058	0.22	0.05	0.40	0.014	0.023
Triglycerides in medium HDL (mmol/l)	2058	0.51	0.33	0.69	<0.0001	2058	-0.08	-0.27	0.11	0.419	2058	-0.20	-0.38	-0.02	0.027	<0.0001
Concentration of small HDL particles (mol/l)	2058	0.18	0.00	0.37	0.049	2058	-0.09	-0.28	0.10	0.374	2058	-0.03	-0.23	0.16	0.722	0.221
Total lipids in small HDL (mmol/l)	2058	0.03	-0.14	0.21	0.702	2058	-0.10	-0.29	0.08	0.270	2058	0.08	-0.11	0.26	0.403	0.497
Phospholipids in small HDL (mmol/l)	2058	0.15	-0.03	0.34	0.106	2058	-0.08	-0.28	0.12	0.443	2058	-0.05	-0.24	0.15	0.643	0.329
Total cholesterol in small HDL (mmol/l)	2058	-0.16	-0.32	0.00	0.045	2058	-0.11	-0.28	0.06	0.211	2058	0.24	0.07	0.40	0.005	0.009
Cholesterol esters in small HDL (mmol/l)	2058	-0.17	-0.32	-0.02	0.029	2058	-0.10	-0.27	0.07	0.231	2058	0.25	0.10	0.41	0.001	0.003

Online Table 4 Associations of regional fat indexes at age 18y with cardiometabolic traits at age 18y in ALSPAC

At age 18y

	At age 18y: Trunk fat index (per 2.0 kg/m^2 higher)					Arm fat index (per 0.3 kg/m^2 higher)					Leg fat index (per 1.3 kg/m^2 higher)					
	Adj. for age, sex, ethnicity, maternal education, smoking, alcohol, puberty timing, arm fat index, leg fat index					*Adj. for age, sex, ethnicity, maternal education, smoking, alcohol, puberty timing, trunk fat index, leg fat index*					*Adj. for age, sex, ethnicity, maternal education, smoking, alcohol, puberty timing, trunk fat index, arm fat index*					*P-value for regional heterogeneity*
**Standardized outcome at age 18y**	**N**	**Beta**	**LCL**	**UCL**	**P-value**	**N**	**Beta**	**LCL**	**UCL**	**P-value**	**N**	**Beta**	**LCL**	**UCL**	**P-value**	
Free cholesterol in small HDL (mmol/l)	2058	-0.09	-0.27	0.10	0.354	2058	-0.09	-0.29	0.10	0.354	2058	0.10	-0.10	0.30	0.347	0.458
Triglycerides in small HDL (mmol/l)	2058	0.41	0.23	0.59	<0.0001	2058	-0.03	-0.24	0.18	0.790	2058	-0.22	-0.41	-0.03	0.025	0.0002
Phospholipids to total lipds ratio in chylomicrons and extremely large VLDL (%)	2058	-0.01	-0.08	0.06	0.792	2058	-0.01	-0.11	0.09	0.826	2058	0.05	-0.03	0.13	0.232	0.594
Total cholesterol to total lipids ratio in chylomicrons and extremely large VLDL (%)	2058	0.22	0.07	0.37	0.004	2058	-0.11	-0.31	0.09	0.287	2058	0.01	-0.18	0.20	0.909	0.058
Cholesterol esters to total lipids ratio in chylomicrons and extremely large VLDL (%)	2058	0.17	0.02	0.32	0.023	2058	-0.13	-0.34	0.09	0.242	2058	0.03	-0.17	0.24	0.737	0.125
Free cholesterol to total lipids ratio in chylomicrons and extremely large VLDL (%)	2058	0.21	0.07	0.36	0.005	2058	0.04	-0.15	0.23	0.685	2058	-0.10	-0.25	0.06	0.236	0.045
Triglycerides to total lipids ratio in chylomicrons and extremely large VLDL (%)	2058	-0.19	-0.32	-0.06	0.003	2058	0.09	-0.09	0.27	0.338	2058	-0.01	-0.17	0.16	0.929	0.061
Phospholipids to total lipds ratio in very large VLDL (%)	2058	0.31	0.17	0.45	<0.0001	2058	0.08	-0.09	0.26	0.352	2058	-0.17	-0.32	-0.02	0.026	0.0004
Total cholesterol to total lipids ratio in very large VLDL (%)	2058	-0.19	-0.32	-0.07	0.003	2058	0.02	-0.15	0.20	0.810	2058	0.13	-0.01	0.28	0.070	0.006
Cholesterol esters to total lipids ratio in very large VLDL (%)	2058	-0.08	-0.23	0.06	0.251	2058	-0.03	-0.19	0.13	0.726	2058	0.07	-0.05	0.19	0.227	0.309
Free cholesterol to total lipids ratio in very large VLDL (%)	2058	-0.11	-0.26	0.04	0.162	2058	0.03	-0.12	0.17	0.726	2058	0.10	-0.01	0.21	0.082	0.170
Triglycerides to total lipids ratio in very large VLDL (%)	2058	0.12	-0.02	0.26	0.094	2058	-0.06	-0.25	0.12	0.484	2058	-0.09	-0.25	0.06	0.228	0.168
Phospholipids to total lipds ratio in large VLDL (%)	2058	0.33	0.20	0.46	<0.0001	2058	0.01	-0.16	0.19	0.896	2058	-0.18	-0.33	-0.04	0.013	<0.0001
Total cholesterol to total lipids ratio in large VLDL (%)	2058	0.23	0.09	0.36	0.001	2058	0.03	-0.15	0.22	0.724	2058	-0.09	-0.25	0.07	0.281	0.029
Cholesterol esters to total lipids ratio in large VLDL (%)	2058	-0.04	-0.17	0.09	0.532	2058	0.07	-0.13	0.26	0.502	2058	0.05	-0.12	0.22	0.562	0.631
Free cholesterol to total lipids ratio in large VLDL (%)	2058	0.21	0.14	0.27	<0.0001	2058	-0.01	-0.09	0.07	0.784	2058	-0.11	-0.17	-0.04	0.002	<0.0001
Triglycerides to total lipids ratio in large VLDL (%)	2058	-0.28	-0.42	-0.15	<0.0001	2058	-0.03	-0.21	0.15	0.740	2058	0.14	-0.02	0.29	0.079	0.001
Phospholipids to total lipds ratio in medium VLDL (%)	2058	-0.49	-0.65	-0.33	<0.0001	2058	0.02	-0.16	0.20	0.831	2058	0.31	0.15	0.46	0.0001	<0.0001
Total cholesterol to total lipids ratio in medium VLDL (%)	2058	0.07	-0.08	0.23	0.338	2058	0.07	-0.12	0.25	0.488	2058	-0.03	-0.19	0.13	0.715	0.686
Cholesterol esters to total lipids ratio in medium VLDL (%)	2058	0.03	-0.13	0.19	0.743	2058	0.06	-0.13	0.24	0.567	2058	0.01	-0.15	0.18	0.858	0.967
Free cholesterol to total lipids ratio in medium VLDL (%)	2058	0.19	0.04	0.33	0.011	2058	0.07	-0.13	0.26	0.492	2058	-0.15	-0.32	0.01	0.068	0.024
Triglycerides to total lipids ratio in medium VLDL (%)	2058	0.02	-0.13	0.18	0.768	2058	-0.06	-0.25	0.13	0.516	2058	-0.03	-0.19	0.13	0.711	0.843
Phospholipids to total lipds ratio in small VLDL (%)	2058	-0.50	-0.66	-0.34	<0.0001	2058	-0.15	-0.34	0.05	0.147	2058	0.37	0.20	0.55	<0.0001	<0.0001
Total cholesterol to total lipids ratio in small VLDL (%)	2058	-0.17	-0.33	0.00	0.046	2058	0.13	-0.07	0.32	0.211	2058	0.08	-0.08	0.25	0.306	0.094
Cholesterol esters to total lipids ratio in small VLDL (%)	2058	-0.10	-0.26	0.06	0.223	2058	0.15	-0.05	0.34	0.146	2058	0.03	-0.13	0.19	0.698	0.300
Free cholesterol to total lipids ratio in small VLDL (%)	2058	-0.45	-0.61	-0.29	<0.0001	2058	-0.12	-0.30	0.06	0.207	2058	0.36	0.20	0.51	<0.0001	<0.0001
Triglycerides to total lipids ratio in small VLDL (%)	2058	0.34	0.18	0.51	<0.0001	2058	-0.08	-0.28	0.12	0.419	2058	-0.21	-0.38	-0.05	0.012	0.0001
Phospholipids to total lipds ratio in very small VLDL (%)	2058	-0.14	-0.29	0.01	0.064	2058	-0.04	-0.21	0.13	0.653	2058	0.18	0.03	0.33	0.021	0.034
Total cholesterol to total lipids ratio in very small VLDL (%)	2058	-0.08	-0.25	0.09	0.344	2058	0.11	-0.09	0.31	0.268	2058	-0.03	-0.19	0.13	0.711	0.510
Cholesterol esters to total lipids ratio in very small VLDL (%)	2058	0.06	-0.10	0.22	0.451	2058	0.13	-0.05	0.31	0.168	2058	-0.12	-0.28	0.03	0.120	0.181
Free cholesterol to total lipids ratio in very small VLDL (%)	2058	-0.51	-0.76	-0.27	<0.0001	2058	-0.02	-0.26	0.22	0.888	2058	0.31	0.13	0.49	0.001	<0.0001
Triglycerides to total lipids ratio in very small VLDL (%)	2058	0.20	0.02	0.38	0.027	2058	-0.11	-0.32	0.10	0.293	2058	-0.09	-0.26	0.08	0.311	0.078
Phospholipids to total lipds ratio in IDL (%)	2058	-0.46	-0.66	-0.26	<0.0001	2058	-0.12	-0.33	0.09	0.256	2058	0.34	0.16	0.52	0.0003	<0.0001
Total cholesterol to total lipids ratio in IDL (%)	2058	0.23	0.05	0.41	0.011	2058	0.14	-0.04	0.33	0.135	2058	-0.19	-0.36	-0.02	0.025	0.008
Cholesterol esters to total lipids ratio in IDL (%)	2058	0.45	0.27	0.63	<0.0001	2058	0.15	-0.04	0.33	0.119	2058	-0.33	-0.49	-0.17	<0.0001	<0.0001
Free cholesterol to total lipids ratio in IDL (%)	2058	-0.49	-0.65	-0.34	<0.0001	2058	0.01	-0.18	0.20	0.932	2058	0.32	0.15	0.49	0.0002	<0.0001
Triglycerides to total lipids ratio in IDL (%)	2058	-0.09	-0.25	0.08	0.304	2058	-0.11	-0.30	0.07	0.231	2058	0.08	-0.09	0.25	0.336	0.347
Phospholipids to total lipds ratio in large LDL (%)	2058	0.02	-0.13	0.16	0.826	2058	-0.08	-0.26	0.10	0.356	2058	-0.02	-0.18	0.15	0.843	0.784
Total cholesterol to total lipids ratio in large LDL (%)	2058	0.10	-0.05	0.25	0.176	2058	0.13	-0.05	0.31	0.159	2058	-0.07	-0.23	0.10	0.425	0.325
Cholesterol esters to total lipids ratio in large LDL (%)	2058	0.22	0.08	0.36	0.002	2058	0.13	-0.05	0.30	0.167	2058	-0.13	-0.29	0.03	0.117	0.018
Free cholesterol to total lipids ratio in large LDL (%)	2058	-0.41	-0.55	-0.26	<0.0001	2058	-0.05	-0.24	0.14	0.613	2058	0.22	0.05	0.39	0.011	<0.0001
Triglycerides to total lipids ratio in large LDL (%)	2058	-0.18	-0.34	-0.02	0.026	2058	-0.11	-0.30	0.07	0.229	2058	0.13	-0.04	0.30	0.140	0.073
Phospholipids to total lipds ratio in medium LDL (%)	2058	0.02	-0.13	0.16	0.814	2058	-0.09	-0.27	0.09	0.315	2058	0.00	-0.16	0.16	0.960	0.751
Total cholesterol to total lipids ratio in medium LDL (%)	2058	0.08	-0.07	0.22	0.293	2058	0.13	-0.05	0.30	0.151	2058	-0.07	-0.23	0.09	0.366	0.338
Cholesterol esters to total lipids ratio in medium LDL (%)	2058	0.12	-0.02	0.26	0.099	2058	0.11	-0.07	0.28	0.235	2058	-0.07	-0.23	0.09	0.379	0.273
Free cholesterol to total lipids ratio in medium LDL (%)	2058	-0.16	-0.29	-0.02	0.023	2058	-0.06	-0.23	0.12	0.519	2058	0.06	-0.10	0.21	0.468	0.195
Triglycerides to total lipids ratio in medium LDL (%)	2058	-0.20	-0.35	-0.05	0.008	2058	-0.10	-0.26	0.06	0.219	2058	0.17	0.02	0.32	0.024	0.010
Phospholipids to total lipds ratio in small LDL (%)	2058	-0.05	-0.19	0.09	0.527	2058	-0.10	-0.27	0.08	0.287	2058	0.02	-0.14	0.18	0.830	0.747
Total cholesterol to total lipids ratio in small LDL (%)	2058	0.00	-0.15	0.14	0.985	2058	0.12	-0.06	0.30	0.189	2058	-0.01	-0.17	0.15	0.930	0.664

Online Table 4 Associations of regional fat indexes at age 18y with cardiometabolic traits at age 18y in ALSPAC

At age 18y

	Trunk fat index (per 2.0 kg/m^2 higher) *Adj. for age, sex, ethnicity, maternal education, smoking, alcohol, puberty timing, arm fat index, leg fat index*					Arm fat index (per 0.3 kg/m^2 higher) *Adj. for age, sex, ethnicity, maternal education, smoking, alcohol, puberty timing, trunk fat index, leg fat index*					Leg fat index (per 1.3 kg/m^2 higher) *Adj. for age, sex, ethnicity, maternal education, smoking, alcohol, puberty timing, trunk fat index, arm fat index*					*P-value for regional heterogeneity*
**Standardized outcome at age 18y**	**N**	**Beta**	**LCL**	**UCL**	**P-value**	**N**	**Beta**	**LCL**	**UCL**	**P-value**	**N**	**Beta**	**LCL**	**UCL**	**P-value**	
Cholesterol esters to total lipids ratio in small LDL (%)	2058	0.06	-0.08	0.21	0.379	2058	0.09	-0.08	0.27	0.301	2058	-0.02	-0.18	0.14	0.810	0.710
Free cholesterol to total lipids ratio in small LDL (%)	2058	-0.16	-0.31	-0.02	0.029	2058	-0.03	-0.21	0.15	0.742	2058	0.04	-0.12	0.19	0.642	0.258
Triglycerides to total lipids ratio in small LDL (%)	2058	0.13	-0.03	0.29	0.120	2058	-0.09	-0.29	0.10	0.362	2058	-0.02	-0.20	0.15	0.778	0.323
Phospholipids to total lipds ratio in very large HDL (%)	2058	-0.56	-0.71	-0.42	<0.0001	2058	-0.15	-0.31	0.00	0.057	2058	0.47	0.32	0.62	<0.0001	<0.0001
Total cholesterol to total lipids ratio in very large HDL (%)	2058	0.49	0.35	0.64	<0.0001	2058	0.16	0.00	0.31	0.043	2058	-0.44	-0.58	-0.29	<0.0001	<0.0001
Cholesterol esters to total lipids ratio in very large HDL (%)	2058	0.49	0.35	0.64	<0.0001	2058	0.15	-0.01	0.30	0.060	2058	-0.43	-0.58	-0.28	<0.0001	<0.0001
Free cholesterol to total lipids ratio in very large HDL (%)	2058	-0.18	-0.36	0.00	0.047	2058	0.07	-0.13	0.27	0.482	2058	0.08	-0.09	0.26	0.362	0.165
Triglycerides to total lipids ratio in very large HDL (%)	2058	0.47	0.27	0.66	<0.0001	2058	0.01	-0.22	0.23	0.956	2058	-0.26	-0.49	-0.02	0.032	0.0003
Phospholipids to total lipds ratio in large HDL (%)	2058	0.63	0.46	0.80	<0.0001	2058	0.01	-0.21	0.22	0.961	2058	-0.29	-0.48	-0.10	0.003	<0.0001
Total cholesterol to total lipids ratio in large HDL (%)	2058	-0.68	-0.85	-0.50	<0.0001	2058	-0.03	-0.24	0.19	0.810	2058	0.34	0.15	0.54	0.001	<0.0001
Cholesterol esters to total lipids ratio in large HDL (%)	2058	-0.67	-0.85	-0.49	<0.0001	2058	-0.03	-0.25	0.19	0.789	2058	0.33	0.13	0.54	0.002	<0.0001
Free cholesterol to total lipids ratio in large HDL (%)	2058	-0.55	-0.71	-0.38	<0.0001	2058	-0.01	-0.21	0.18	0.887	2058	0.32	0.15	0.49	0.0003	<0.0001
Triglycerides to total lipids ratio in large HDL (%)	2058	0.61	0.43	0.80	<0.0001	2058	0.04	-0.17	0.26	0.680	2058	-0.36	-0.58	-0.15	0.001	<0.0001
Phospholipids to total lipds ratio in medium HDL (%)	2058	-0.21	-0.38	-0.04	0.016	2058	0.03	-0.18	0.24	0.783	2058	0.10	-0.07	0.28	0.246	0.080
Total cholesterol to total lipids ratio in medium HDL (%)	2058	-0.12	-0.29	0.05	0.174	2058	-0.03	-0.25	0.19	0.785	2058	0.06	-0.12	0.25	0.501	0.468
Cholesterol esters to total lipids ratio in medium HDL (%)	2058	-0.13	-0.30	0.04	0.138	2058	-0.02	-0.24	0.19	0.840	2058	0.05	-0.14	0.23	0.601	0.478
Free cholesterol to total lipids ratio in medium HDL (%)	2058	0.03	-0.13	0.18	0.732	2058	-0.08	-0.28	0.12	0.423	2058	0.12	-0.06	0.30	0.188	0.505
Triglycerides to total lipids ratio in medium HDL (%)	2058	0.60	0.40	0.79	<0.0001	2058	0.01	-0.21	0.23	0.906	2058	-0.31	-0.52	-0.09	0.005	<0.0001
Phospholipids to total lipds ratio in small HDL (%)	2058	0.24	0.09	0.38	0.002	2058	0.06	-0.11	0.23	0.472	2058	-0.27	-0.41	-0.12	0.0003	0.0001
Total cholesterol to total lipids ratio in small HDL (%)	2058	-0.34	-0.49	-0.20	<0.0001	2058	-0.08	-0.26	0.10	0.396	2058	0.34	0.19	0.49	<0.0001	<0.0001
Cholesterol esters to total lipids ratio in small HDL (%)	2058	-0.27	-0.42	-0.12	0.0003	2058	-0.07	-0.25	0.11	0.447	2058	0.31	0.16	0.46	<0.0001	<0.0001
Free cholesterol to total lipids ratio in small HDL (%)	2058	-0.32	-0.50	-0.15	0.0003	2058	-0.01	-0.21	0.19	0.939	2058	0.05	-0.14	0.24	0.594	0.041
Triglycerides to total lipids ratio in small HDL (%)	2058	0.41	0.22	0.60	<0.0001	2058	0.06	-0.19	0.31	0.651	2058	-0.28	-0.52	-0.05	0.020	0.001
Mean diameter for VLDL particles (nm)	2058	0.71	0.55	0.87	<0.0001	2058	0.02	-0.18	0.21	0.876	2058	-0.44	-0.61	-0.27	<0.0001	<0.0001
Mean diameter for LDL particles (nm)	2058	-0.21	-0.36	-0.06	0.007	2058	-0.06	-0.24	0.13	0.552	2058	0.12	-0.04	0.28	0.158	0.046
Mean diameter for HDL particles (nm)	2058	-0.63	-0.77	-0.48	<0.0001	2058	-0.04	-0.20	0.13	0.648	2058	0.42	0.26	0.57	<0.0001	<0.0001
Serum total cholesterol (mmol/l)	2058	0.06	-0.11	0.22	0.504	2058	0.02	-0.18	0.21	0.868	2058	0.06	-0.11	0.23	0.499	0.962
Total cholesterol in VLDL (mmol/l)	2058	0.60	0.42	0.77	<0.0001	2058	0.06	-0.15	0.28	0.562	2058	-0.33	-0.52	-0.13	0.001	<0.0001
Remnant cholesterol (non-HDL, non-LDL -cholesterol) (mmol/l)	2058	0.42	0.24	0.60	<0.0001	2058	0.06	-0.16	0.27	0.609	2058	-0.20	-0.39	-0.01	0.044	0.0003
Total cholesterol in LDL (mmol/l)	2058	0.11	-0.06	0.28	0.221	2058	0.03	-0.18	0.23	0.793	2058	0.02	-0.15	0.20	0.811	0.811
Total cholesterol in HDL (mmol/l)	2058	-0.50	-0.66	-0.35	<0.0001	2058	-0.06	-0.23	0.11	0.496	2058	0.37	0.20	0.54	<0.0001	<0.0001
Total cholesterol in HDL2 (mmol/l)	2058	-0.56	-0.72	-0.40	<0.0001	2058	-0.06	-0.24	0.12	0.493	2058	0.40	0.22	0.57	<0.0001	<0.0001
Total cholesterol in HDL3 (mmol/l)	2058	-0.37	-0.53	-0.22	<0.0001	2058	-0.05	-0.22	0.11	0.533	2058	0.30	0.14	0.46	0.0003	<0.0001
Esterified cholesterol (mmol/l)	2051	0.09	-0.08	0.25	0.315	2051	0.03	-0.16	0.23	0.745	2051	0.01	-0.16	0.18	0.922	0.854
Free cholesterol (mmol/l)	2049	0.01	-0.16	0.17	0.921	2049	-0.04	-0.23	0.16	0.712	2049	0.16	0.00	0.33	0.054	0.358
Serum total triglycerides (mmol/l)	2058	0.60	0.42	0.78	<0.0001	2058	0.03	-0.19	0.26	0.759	2058	-0.35	-0.55	-0.15	0.001	<0.0001
Triglycerides in VLDL (mmol/l)	2058	0.69	0.52	0.86	<0.0001	2058	0.05	-0.17	0.27	0.631	2058	-0.43	-0.62	-0.23	<0.0001	<0.0001
Triglycerides in LDL (mmol/l)	2058	-0.03	-0.21	0.15	0.739	2058	-0.02	-0.23	0.18	0.809	2058	0.08	-0.10	0.25	0.391	0.726
Triglycerides in HDL (mmol/l)	2058	0.34	0.16	0.52	0.0002	2058	-0.06	-0.26	0.15	0.581	2058	-0.13	-0.32	0.06	0.168	0.006
Diacylglycerol (mmol/l)	1997	0.36	0.16	0.55	0.0003	1997	0.07	-0.16	0.30	0.546	1997	-0.22	-0.41	-0.02	0.029	0.002
Ratio of diacylglycerol to triglycerides	1998	0.05	-0.13	0.23	0.584	1998	0.08	-0.14	0.29	0.474	1998	-0.02	-0.19	0.14	0.777	0.780
Total phosphoglycerides (mmol/l)	2049	-0.12	-0.28	0.05	0.174	2049	-0.02	-0.21	0.17	0.845	2049	0.13	-0.04	0.30	0.125	0.212
Ratio of triglycerides to phosphoglycerides	2049	0.68	0.51	0.85	<0.0001	2049	0.00	-0.22	0.22	0.998	2049	-0.39	-0.59	-0.20	<0.0001	<0.0001
Phosphatidylcholine and other cholines (mmol/l)	2034	-0.08	-0.25	0.09	0.339	2034	-0.07	-0.25	0.12	0.460	2034	0.12	-0.04	0.29	0.148	0.277
Total cholines (mmol/l)	2051	-0.11	-0.28	0.05	0.182	2051	-0.06	-0.24	0.13	0.534	2051	0.17	0.01	0.33	0.043	0.095
Apolipoprotein A-I (g/l)	2058	-0.29	-0.45	-0.14	0.0001	2058	-0.05	-0.22	0.12	0.541	2058	0.26	0.10	0.42	0.001	0.0001
Apolipoprotein B (g/l)	2058	0.48	0.30	0.66	<0.0001	2058	0.05	-0.17	0.26	0.669	2058	-0.24	-0.43	-0.04	0.017	<0.0001
Ratio of apolipoprotein B to apolipoprotein A-I	2058	0.61	0.43	0.79	<0.0001	2058	0.08	-0.14	0.30	0.472	2058	-0.36	-0.56	-0.15	0.001	<0.0001
Total fatty acids (mmol/l)	2051	0.26	0.09	0.43	0.003	2051	0.01	-0.20	0.21	0.960	2051	-0.09	-0.27	0.10	0.365	0.062

Online Table 4 Associations of regional fat indexes at age 18y with cardiometabolic traits at age 18y in ALSPAC

	At age 18y															
	**Trunk fat index (per 2.0 kg/m^2 higher)**					**Arm fat index (per 0.3 kg/m^2 higher)**					**Leg fat index (per 1.3 kg/m^2 higher)**					
	Adj. for age, sex, ethnicity, maternal education, smoking, alcohol, puberty timing, arm fat index, leg fat index					*Adj. for age, sex, ethnicity, maternal education, smoking, alcohol, puberty timing, trunk fat index, leg fat index*					*Adj. for age, sex, ethnicity, maternal education, smoking, alcohol, puberty timing, trunk fat index, arm fat index*					*P-value for regional heterogeneity*
**Standardized outcome at age 18y**	**N**	**Beta**	**LCL**	**UCL**	**P-value**	**N**	**Beta**	**LCL**	**UCL**	**P-value**	**N**	**Beta**	**LCL**	**UCL**	**P-value**	
Estimated description of fatty acid chain length, not actual carbon number	2052	0.01	-0.16	0.17	0.947	2052	-0.01	-0.20	0.18	0.921	2052	0.00	-0.16	0.16	0.969	0.995
Estimated degree of unsaturation	2051	-0.26	-0.41	-0.10	0.001	2051	-0.04	-0.23	0.15	0.694	2051	0.23	0.07	0.39	0.005	0.001
22:6, docosahexaenoic acid (mmol/l)	2051	-0.06	-0.23	0.11	0.499	2051	0.09	-0.10	0.28	0.353	2051	0.02	-0.14	0.19	0.810	0.657
18:2, linoleic acid (mmol/l)	2051	0.15	-0.01	0.32	0.066	2051	-0.10	-0.30	0.09	0.294	2051	0.02	-0.15	0.20	0.791	0.270
Conjugated linoleic acid (mmol/l)	2050	0.15	-0.01	0.32	0.072	2050	0.13	-0.07	0.33	0.203	2050	-0.18	-0.36	0.00	0.049	0.054
Omega-3 fatty acids (mmol/l)	2051	0.10	-0.06	0.26	0.241	2051	0.13	-0.07	0.33	0.212	2051	-0.09	-0.25	0.07	0.265	0.245
Omega-6 fatty acids (mmol/l)	2051	0.13	-0.03	0.30	0.116	2051	-0.06	-0.26	0.13	0.533	2051	0.04	-0.14	0.21	0.685	0.483
Polyunsaturated fatty acids (mmol/l)	2051	0.13	-0.03	0.30	0.114	2051	-0.04	-0.23	0.16	0.699	2051	0.02	-0.15	0.19	0.825	0.532
Monounsaturated fatty acids; 16:1, 18:1 (mmol/l)	2051	0.35	0.18	0.51	<0.0001	2051	0.00	-0.21	0.21	0.973	2051	-0.13	-0.32	0.06	0.171	0.004
Saturated fatty acids (mmol/l)	2050	0.22	0.05	0.40	0.013	2050	0.04	-0.16	0.25	0.686	2050	-0.11	-0.30	0.07	0.236	0.096
Ratio of 22:6 docosahexaenoic acid to total fatty acids (%)	2052	-0.23	-0.38	-0.07	0.005	2052	0.12	-0.05	0.30	0.170	2052	0.07	-0.08	0.23	0.351	0.030
Ratio of 18:2 linoleic acid to total fatty acids (%)	2052	-0.20	-0.37	-0.04	0.016	2052	-0.16	-0.36	0.03	0.092	2052	0.17	0.01	0.34	0.040	0.013
Ratio of conjugated linoleic acid to total fatty acids (%)	2051	0.10	-0.05	0.25	0.185	2051	0.12	-0.07	0.30	0.211	2051	-0.14	-0.31	0.03	0.107	0.156
Ratio of omega-3 fatty acids to total fatty acids (%)	2052	-0.10	-0.25	0.06	0.219	2052	0.16	-0.03	0.35	0.102	2052	-0.05	-0.21	0.11	0.514	0.251
Ratio of omega-6 fatty acids to total fatty acids (%)	2052	-0.31	-0.47	-0.14	0.0003	2052	-0.10	-0.30	0.09	0.299	2052	0.24	0.08	0.41	0.004	0.0002
Ratio of polyunsaturated fatty acids to total fatty acids (%)	2052	-0.32	-0.48	-0.15	0.0002	2052	-0.06	-0.25	0.14	0.549	2052	0.22	0.05	0.38	0.011	0.001
Ratio of monounsaturated fatty acids to total fatty acids (%)	2052	0.35	0.19	0.51	<0.0001	2052	0.00	-0.19	0.19	0.971	2052	-0.16	-0.32	0.00	0.055	0.0005
Ratio of saturated fatty acids to total fatty acids (%)	2051	-0.10	-0.26	0.07	0.257	2051	0.08	-0.11	0.27	0.411	2051	-0.05	-0.22	0.12	0.585	0.547
Insulin (mu/l)	2097	0.49	0.19	0.79	0.001	2097	-0.19	-0.47	0.09	0.185	2097	0.02	-0.14	0.19	0.780	0.017
Glucose (mmol/l)	2057	0.08	-0.10	0.26	0.393	2057	0.13	-0.10	0.35	0.263	2057	-0.08	-0.20	0.03	0.148	0.093
Lactate (mmol/l)	2057	-0.17	-0.32	-0.01	0.036	2057	0.24	0.07	0.41	0.006	2057	-0.03	-0.18	0.13	0.722	0.021
Pyruvate (mmol/l)	2057	0.07	-0.08	0.22	0.355	2057	0.30	0.13	0.47	0.001	2057	-0.21	-0.36	-0.06	0.006	0.001
Citrate (mmol/l)	2057	-0.20	-0.35	-0.04	0.011	2057	-0.09	-0.27	0.09	0.330	2057	0.14	0.00	0.29	0.058	0.016
Alanine (mmol/l)	2057	0.30	0.14	0.46	0.0002	2057	-0.07	-0.25	0.12	0.481	2057	-0.13	-0.30	0.03	0.107	0.004
Glutamine (mmol/l)	2057	0.11	-0.03	0.26	0.117	2057	-0.07	-0.25	0.10	0.419	2057	-0.08	-0.22	0.06	0.276	0.199
Histidine (mmol/l)	2057	0.20	0.04	0.36	0.015	2057	-0.12	-0.32	0.08	0.233	2057	-0.05	-0.20	0.11	0.553	0.071
Isoleucine (mmol/l)	2057	0.56	0.42	0.71	<0.0001	2057	-0.03	-0.20	0.14	0.703	2057	-0.33	-0.48	-0.18	<0.0001	<0.0001
Leucine (mmol/l)	2057	0.42	0.29	0.56	<0.0001	2057	-0.11	-0.27	0.05	0.160	2057	-0.19	-0.33	-0.05	0.006	<0.0001
Valine (mmol/l)	2057	0.36	0.21	0.50	<0.0001	2057	0.04	-0.12	0.20	0.609	2057	-0.19	-0.34	-0.04	0.016	0.0001
Phenylalanine (mmol/l)	2057	0.35	0.20	0.50	<0.0001	2057	-0.24	-0.41	-0.06	0.008	2057	0.06	-0.08	0.21	0.390	0.0003
Tyrosine (mmol/l)	2057	0.37	0.23	0.52	<0.0001	2057	-0.04	-0.21	0.14	0.688	2057	-0.06	-0.22	0.10	0.474	0.001
Acetate (mmol/l)	2057	-0.11	-0.20	-0.02	0.013	2057	0.09	-0.03	0.20	0.139	2057	-0.06	-0.19	0.08	0.429	0.043
Acetoacetate (mmol/l)	2057	-0.17	-0.33	-0.01	0.033	2057	0.09	-0.09	0.28	0.315	2057	0.02	-0.12	0.16	0.744	0.163
3-hydroxybutyrate (mmol/l)	2054	-0.21	-0.40	-0.02	0.027	2054	0.08	-0.11	0.28	0.400	2054	0.06	-0.09	0.21	0.442	0.132
Creatinine (mmol/l)	2057	0.27	0.14	0.40	<0.0001	2057	-0.24	-0.39	-0.09	0.002	2057	-0.05	-0.18	0.07	0.411	0.0001
Albumin (signal area)	2058	-0.01	-0.16	0.13	0.844	2058	0.03	-0.15	0.20	0.761	2058	-0.11	-0.27	0.05	0.189	0.630
Glycoprotein acetyls, mainly a1-acid glycoprotein (mmol/l)	2057	0.52	0.36	0.67	<0.0001	2057	0.02	-0.17	0.21	0.847	2057	-0.20	-0.35	-0.04	0.015	<0.0001
C-reactive protein (mg/l)	2124	0.11	-0.01	0.23	0.080	2124	-0.02	-0.13	0.09	0.701	2124	0.02	-0.12	0.17	0.739	0.415

Complete case sample

	At age 18y			
	**Trunk fat index (per 1.9 kg/m^2 higher)**	**Arm fat index (per 0.3 kg/m^2 higher)**	**Leg fat index (per 1.3 kg/m^2 higher)**	
	Adj. for age, sex, ethnicity, maternal education, smoking, alcohol, puberty timing, arm fat index, leg fat index	*Adj. for age, sex, ethnicity, maternal education, smoking, alcohol, puberty timing, trunk fat index, leg fat index*	*Adj. for age, sex, ethnicity, maternal education, smoking, alcohol, puberty timing, trunk fat index, arm fat index*	*P-value for*

Online Table 4 Associations of regional fat indexes at age 18y with cardiometabolic traits at age 18y in ALSPAC

	At age 18y															
	Trunk fat index (per 2.0 kg/m^2 higher)					Arm fat index (per 0.3 kg/m^2 higher)					Leg fat index (per 1.3 kg/m^2 higher)					
	Adj. for age, sex, ethnicity, maternal education, smoking, alcohol, puberty timing, arm fat index, leg fat index					*Adj. for age, sex, ethnicity, maternal education, smoking, alcohol, puberty timing, trunk fat index, leg fat index*					*Adj. for age, sex, ethnicity, maternal education, smoking, alcohol, puberty timing, trunk fat index, arm fat index*					*P-value for regional heterogeneity*
**Standardized outcome at age 18y**	**N**	**Beta**	**LCL**	**UCL**	**P-value**	**N**	**Beta**	**LCL**	**UCL**	**P-value**	**N**	**Beta**	**LCL**	**UCL**	**P-value**	
**Standardized outcome at age 18y**	**N**	**Beta**	**LCL**	**UCL**	**P-value**	**N**	**Beta**	**LCL**	**UCL**	**P-value**	**N**	**Beta**	**LCL**	**UCL**	**P-value**	*regional heterogeneity*
Systolic blood pressure (mmHg)	1722	0.21	0.07	0.35	0.002	1722	0.02	-0.16	0.19	0.844	1722	0.01	-0.15	0.17	0.930	0.172
Diastolic blood pressure (mmHg)	1722	0.21	0.04	0.38	0.018	1722	0.20	-0.01	0.40	0.061	1722	-0.06	-0.26	0.13	0.526	0.186
Concentration of chylomicrons and extremely large VLDL particles (mol/l)	1722	0.66	0.48	0.84	<0.0001	1722	0.12	-0.17	0.40	0.422	1722	-0.41	-0.66	-0.16	0.001	<0.0001
Total lipids in chylomicrons and extremely large VLDL (mmol/l)	1722	0.66	0.48	0.83	<0.0001	1722	0.11	-0.16	0.39	0.418	1722	-0.42	-0.66	-0.17	0.001	<0.0001
Phospholipids in chylomicrons and extremely large VLDL (mmol/l)	1722	0.65	0.47	0.83	<0.0001	1722	0.12	-0.16	0.40	0.406	1722	-0.42	-0.66	-0.17	0.001	<0.0001
Total cholesterol in chylomicrons and extremely large VLDL (mmol/l)	1722	0.64	0.47	0.82	<0.0001	1722	0.07	-0.20	0.35	0.599	1722	-0.37	-0.61	-0.12	0.003	<0.0001
Cholesterol esters in chylomicrons and extremely large VLDL (mmol/l)	1722	0.63	0.45	0.81	<0.0001	1722	0.04	-0.23	0.31	0.779	1722	-0.32	-0.57	-0.08	0.010	<0.0001
Free cholesterol in chylomicrons and extremely large VLDL (mmol/l)	1722	0.64	0.46	0.82	<0.0001	1722	0.11	-0.17	0.39	0.427	1722	-0.41	-0.65	-0.16	0.001	<0.0001
Triglycerides in chylomicrons and extremely large VLDL (mmol/l)	1722	0.66	0.48	0.84	<0.0001	1722	0.12	-0.15	0.40	0.384	1722	-0.43	-0.67	-0.19	0.001	<0.0001
Concentration of very large VLDL particles (mol/l)	1722	0.68	0.50	0.85	<0.0001	1722	0.09	-0.18	0.36	0.522	1722	-0.42	-0.66	-0.18	0.001	<0.0001
Total lipids in very large VLDL (mmol/l)	1722	0.68	0.50	0.86	<0.0001	1722	0.08	-0.19	0.34	0.566	1722	-0.42	-0.65	-0.18	0.001	<0.0001
Phospholipids in very large VLDL (mmol/l)	1722	0.66	0.48	0.84	<0.0001	1722	0.08	-0.19	0.35	0.558	1722	-0.40	-0.64	-0.16	0.001	<0.0001
Total cholesterol in very large VLDL (mmol/l)	1722	0.67	0.50	0.85	<0.0001	1722	0.08	-0.19	0.35	0.546	1722	-0.39	-0.64	-0.15	0.001	<0.0001
Cholesterol esters in very large VLDL (mmol/l)	1722	0.69	0.51	0.86	<0.0001	1722	0.07	-0.19	0.34	0.583	1722	-0.40	-0.64	-0.16	0.001	<0.0001
Free cholesterol in very large VLDL (mmol/l)	1722	0.65	0.47	0.83	<0.0001	1722	0.09	-0.18	0.37	0.510	1722	-0.39	-0.63	-0.14	0.002	<0.0001
Triglycerides in very large VLDL (mmol/l)	1722	0.68	0.51	0.86	<0.0001	1722	0.07	-0.19	0.34	0.578	1722	-0.43	-0.66	-0.19	0.0004	<0.0001
Concentration of large VLDL particles (mol/l)	1722	0.69	0.52	0.87	<0.0001	1722	0.04	-0.21	0.29	0.738	1722	-0.41	-0.64	-0.19	0.0003	<0.0001
Total lipids in large VLDL (mmol/l)	1722	0.69	0.52	0.87	<0.0001	1722	0.04	-0.21	0.29	0.752	1722	-0.41	-0.63	-0.18	0.0004	<0.0001
Phospholipids in large VLDL (mmol/l)	1722	0.68	0.51	0.86	<0.0001	1722	0.04	-0.21	0.29	0.775	1722	-0.39	-0.62	-0.17	0.001	<0.0001
Total cholesterol in large VLDL (mmol/l)	1722	0.68	0.51	0.86	<0.0001	1722	0.03	-0.22	0.28	0.807	1722	-0.38	-0.60	-0.15	0.001	<0.0001
Cholesterol esters in large VLDL (mmol/l)	1722	0.68	0.51	0.86	<0.0001	1722	0.02	-0.23	0.27	0.879	1722	-0.36	-0.58	-0.13	0.002	<0.0001
Free cholesterol in large VLDL (mmol/l)	1722	0.68	0.50	0.85	<0.0001	1722	0.04	-0.21	0.30	0.741	1722	-0.39	-0.62	-0.16	0.001	<0.0001
Triglycerides in large VLDL (mmol/l)	1722	0.70	0.52	0.87	<0.0001	1722	0.04	-0.20	0.29	0.723	1722	-0.42	-0.64	-0.20	0.0002	<0.0001
Concentration of medium VLDL particles (mol/l)	1722	0.71	0.53	0.89	<0.0001	1722	0.03	-0.21	0.27	0.818	1722	-0.40	-0.62	-0.19	0.0003	<0.0001
Total lipids in medium VLDL (mmol/l)	1722	0.71	0.53	0.88	<0.0001	1722	0.02	-0.21	0.26	0.848	1722	-0.39	-0.61	-0.17	0.0004	<0.0001
Phospholipids in medium VLDL (mmol/l)	1722	0.68	0.50	0.86	<0.0001	1722	0.02	-0.22	0.26	0.867	1722	-0.37	-0.59	-0.15	0.001	<0.0001
Total cholesterol in medium VLDL (mmol/l)	1722	0.64	0.46	0.82	<0.0001	1722	0.00	-0.23	0.24	0.977	1722	-0.30	-0.53	-0.08	0.007	<0.0001
Cholesterol esters in medium VLDL (mmol/l)	1722	0.59	0.40	0.78	<0.0001	1722	-0.01	-0.25	0.23	0.927	1722	-0.24	-0.47	-0.02	0.033	<0.0001
Free cholesterol in medium VLDL (mmol/l)	1722	0.66	0.48	0.84	<0.0001	1722	0.02	-0.22	0.26	0.863	1722	-0.36	-0.58	-0.14	0.001	<0.0001
Triglycerides in medium VLDL (mmol/l)	1722	0.73	0.55	0.90	<0.0001	1722	0.03	-0.20	0.27	0.782	1722	-0.43	-0.65	-0.22	<0.0001	<0.0001
Concentration of small VLDL particles (mol/l)	1722	0.62	0.44	0.80	<0.0001	1722	0.00	-0.23	0.24	0.984	1722	-0.32	-0.53	-0.10	0.004	<0.0001
Total lipids in small VLDL (mmol/l)	1722	0.61	0.42	0.79	<0.0001	1722	0.01	-0.22	0.25	0.925	1722	-0.30	-0.51	-0.08	0.006	<0.0001
Phospholipids in small VLDL (mmol/l)	1722	0.56	0.38	0.74	<0.0001	1722	-0.03	-0.26	0.21	0.822	1722	-0.25	-0.45	-0.04	0.019	<0.0001
Total cholesterol in small VLDL (mmol/l)	1722	0.50	0.31	0.69	<0.0001	1722	0.04	-0.20	0.28	0.734	1722	-0.22	-0.43	-0.01	0.041	<0.0001
Cholesterol esters in small VLDL (mmol/l)	1722	0.46	0.26	0.65	<0.0001	1722	0.07	-0.17	0.30	0.588	1722	-0.20	-0.42	0.01	0.058	0.0003
Free cholesterol in small VLDL (mmol/l)	1722	0.53	0.34	0.71	<0.0001	1722	-0.01	-0.25	0.22	0.923	1722	-0.23	-0.44	-0.02	0.034	<0.0001
Triglycerides in small VLDL (mmol/l)	1722	0.64	0.46	0.82	<0.0001	1722	0.00	-0.24	0.24	0.996	1722	-0.34	-0.56	-0.13	0.002	<0.0001
Concentration of very small VLDL particles (mol/l)	1722	0.21	0.01	0.40	0.036	1722	0.00	-0.24	0.23	0.969	1722	0.00	-0.20	0.21	0.965	0.393
Total lipids in very small VLDL (mmol/l)	1722	0.25	0.06	0.45	0.011	1722	0.04	-0.19	0.28	0.724	1722	-0.06	-0.27	0.15	0.581	0.166
Phospholipids in very small VLDL (mmol/l)	1722	0.13	-0.07	0.32	0.196	1722	0.00	-0.23	0.22	0.984	1722	0.06	-0.14	0.26	0.544	0.793
Total cholesterol in very small VLDL (mmol/l)	1722	0.22	0.02	0.43	0.029	1722	0.09	-0.15	0.33	0.455	1722	-0.09	-0.30	0.12	0.399	0.171
Cholesterol esters in very small VLDL (mmol/l)	1722	0.28	0.09	0.48	0.004	1722	0.12	-0.12	0.35	0.342	1722	-0.15	-0.36	0.06	0.160	0.032
Free cholesterol in very small VLDL (mmol/l)	1722	0.06	-0.16	0.27	0.603	1722	0.02	-0.21	0.26	0.845	1722	0.06	-0.14	0.27	0.537	0.978
Triglycerides in very small VLDL (mmol/l)	1722	0.35	0.16	0.54	0.0002	1722	-0.03	-0.27	0.21	0.810	1722	-0.12	-0.33	0.09	0.264	0.010
Concentration of IDL particles (mol/l)	1722	0.05	-0.15	0.24	0.641	1722	0.03	-0.20	0.26	0.793	1722	0.08	-0.12	0.28	0.456	0.968
Total lipids in IDL (mmol/l)	1722	0.06	-0.13	0.25	0.551	1722	0.03	-0.20	0.25	0.827	1722	0.08	-0.12	0.28	0.407	0.952
Phospholipids in IDL (mmol/l)	1722	0.00	-0.19	0.20	0.977	1722	0.02	-0.21	0.25	0.860	1722	0.12	-0.08	0.31	0.237	0.745

Online Table 4 Associations of regional fat indexes at age 18y with cardiometabolic traits at age 18y in ALSPAC

	At age 18y															
	**Trunk fat index (per 2.0 kg/m^2 higher)**					**Arm fat index (per 0.3 kg/m^2 higher)**					**Leg fat index (per 1.3 kg/m^2 higher)**					
	Adj. for age, sex, ethnicity, maternal education, smoking, alcohol, puberty timing, arm fat index, leg fat index					*Adj. for age, sex, ethnicity, maternal education, smoking, alcohol, puberty timing, trunk fat index, leg fat index*					*Adj. for age, sex, ethnicity, maternal education, smoking, alcohol, puberty timing, trunk fat index, arm fat index*					***P-value for regional heterogeneity***
**Standardized outcome at age 18y**	**N**	**Beta**	**LCL**	**UCL**	**P-value**	**N**	**Beta**	**LCL**	**UCL**	**P-value**	**N**	**Beta**	**LCL**	**UCL**	**P-value**	
Total cholesterol in IDL (mmol/l)	1722	0.09	-0.10	0.29	0.343	1722	0.04	-0.19	0.27	0.752	1722	0.06	-0.14	0.26	0.577	0.953
Cholesterol esters in IDL (mmol/l)	1722	0.15	-0.04	0.35	0.122	1722	0.04	-0.19	0.28	0.721	1722	0.02	-0.19	0.22	0.859	0.693
Free cholesterol in IDL (mmol/l)	1722	-0.05	-0.25	0.14	0.574	1722	0.02	-0.20	0.25	0.839	1722	0.15	-0.05	0.35	0.134	0.430
Triglycerides in IDL (mmol/l)	1722	-0.04	-0.23	0.15	0.668	1722	-0.03	-0.26	0.20	0.784	1722	0.13	-0.07	0.33	0.203	0.527
Concentration of large LDL particles (mol/l)	1722	0.06	-0.13	0.25	0.551	1722	0.02	-0.21	0.24	0.882	1722	0.09	-0.11	0.28	0.381	0.933
Total lipids in large LDL (mmol/l)	1722	0.06	-0.13	0.24	0.565	1722	0.02	-0.21	0.24	0.875	1722	0.09	-0.10	0.29	0.339	0.915
Phospholipids in large LDL (mmol/l)	1722	0.10	-0.09	0.29	0.305	1722	0.01	-0.22	0.24	0.930	1722	0.08	-0.12	0.27	0.424	0.894
Total cholesterol in large LDL (mmol/l)	1722	0.06	-0.13	0.25	0.508	1722	0.02	-0.20	0.25	0.831	1722	0.09	-0.11	0.28	0.377	0.944
Cholesterol esters in large LDL (mmol/l)	1722	0.09	-0.10	0.28	0.339	1722	0.02	-0.20	0.25	0.828	1722	0.07	-0.12	0.27	0.472	0.939
Free cholesterol in large LDL (mmol/l)	1722	-0.02	-0.21	0.17	0.808	1722	0.02	-0.20	0.25	0.842	1722	0.14	-0.06	0.33	0.167	0.578
Triglycerides in large LDL (mmol/l)	1722	-0.11	-0.30	0.08	0.275	1722	-0.02	-0.24	0.21	0.886	1722	0.15	-0.05	0.34	0.134	0.282
Concentration of medium LDL particles (mol/l)	1722	0.11	-0.08	0.31	0.252	1722	0.01	-0.22	0.24	0.907	1722	0.05	-0.14	0.25	0.598	0.863
Total lipids in medium LDL (mmol/l)	1722	0.09	-0.10	0.28	0.335	1722	0.02	-0.21	0.24	0.883	1722	0.07	-0.12	0.26	0.477	0.923
Phospholipids in medium LDL (mmol/l)	1722	0.17	-0.01	0.35	0.067	1722	0.01	-0.21	0.24	0.914	1722	0.04	-0.15	0.23	0.699	0.592
Total cholesterol in medium LDL (mmol/l)	1722	0.09	-0.10	0.29	0.339	1722	0.02	-0.20	0.25	0.839	1722	0.07	-0.13	0.26	0.510	0.936
Cholesterol esters in medium LDL (mmol/l)	1722	0.10	-0.09	0.30	0.308	1722	0.02	-0.21	0.25	0.858	1722	0.06	-0.13	0.26	0.533	0.917
Free cholesterol in medium LDL (mmol/l)	1722	0.06	-0.12	0.25	0.516	1722	0.03	-0.19	0.26	0.763	1722	0.08	-0.12	0.27	0.427	0.973
Triglycerides in medium LDL (mmol/l)	1722	-0.10	-0.30	0.10	0.339	1722	-0.03	-0.25	0.20	0.799	1722	0.15	-0.04	0.34	0.114	0.279
Concentration of small LDL particles (mol/l)	1722	0.12	-0.07	0.32	0.220	1722	0.03	-0.20	0.26	0.793	1722	0.03	-0.16	0.22	0.781	0.816
Total lipids in small LDL (mmol/l)	1722	0.11	-0.08	0.30	0.247	1722	0.03	-0.20	0.25	0.826	1722	0.05	-0.14	0.24	0.613	0.880
Phospholipids in small LDL (mmol/l)	1722	0.16	-0.02	0.35	0.076	1722	0.02	-0.20	0.24	0.850	1722	0.02	-0.17	0.21	0.859	0.571
Total cholesterol in small LDL (mmol/l)	1722	0.09	-0.10	0.28	0.369	1722	0.03	-0.20	0.26	0.787	1722	0.06	-0.13	0.26	0.529	0.958
Cholesterol esters in small LDL (mmol/l)	1722	0.09	-0.11	0.29	0.370	1722	0.03	-0.20	0.26	0.819	1722	0.07	-0.13	0.26	0.513	0.950
Free cholesterol in small LDL (mmol/l)	1722	0.08	-0.11	0.26	0.422	1722	0.05	-0.18	0.28	0.667	1722	0.05	-0.14	0.24	0.627	0.979
Triglycerides in small LDL (mmol/l)	1722	0.14	-0.06	0.34	0.173	1722	-0.02	-0.26	0.22	0.858	1722	0.01	-0.19	0.21	0.925	0.644
Concentration of very large HDL particles (mol/l)	1722	-0.58	-0.74	-0.42	<0.0001	1722	0.04	-0.14	0.23	0.647	1722	0.36	0.18	0.54	<0.0001	<0.0001
Total lipids in very large HDL (mmol/l)	1722	-0.57	-0.74	-0.40	<0.0001	1722	0.08	-0.11	0.27	0.415	1722	0.32	0.14	0.50	0.001	<0.0001
Phospholipids in very large HDL (mmol/l)	1722	-0.62	-0.78	-0.45	<0.0001	1722	0.00	-0.18	0.18	0.990	1722	0.41	0.24	0.59	<0.0001	<0.0001
Total cholesterol in very large HDL (mmol/l)	1722	-0.47	-0.65	-0.30	<0.0001	1722	0.16	-0.05	0.36	0.131	1722	0.19	0.01	0.38	0.041	<0.0001
Cholesterol esters in very large HDL (mmol/l)	1722	-0.44	-0.62	-0.26	<0.0001	1722	0.18	-0.03	0.38	0.085	1722	0.15	-0.04	0.33	0.119	<0.0001
Free cholesterol in very large HDL (mmol/l)	1722	-0.54	-0.72	-0.37	<0.0001	1722	0.08	-0.11	0.28	0.416	1722	0.30	0.12	0.49	0.001	<0.0001
Triglycerides in very large HDL (mmol/l)	1722	-0.11	-0.28	0.07	0.231	1722	0.09	-0.14	0.32	0.446	1722	0.05	-0.17	0.26	0.682	0.442
Concentration of large HDL particles (mol/l)	1722	-0.61	-0.77	-0.44	<0.0001	1722	-0.08	-0.27	0.11	0.417	1722	0.45	0.26	0.63	<0.0001	<0.0001
Total lipids in large HDL (mmol/l)	1722	-0.60	-0.77	-0.44	<0.0001	1722	-0.08	-0.27	0.11	0.405	1722	0.45	0.26	0.63	<0.0001	<0.0001
Phospholipids in large HDL (mmol/l)	1722	-0.56	-0.73	-0.40	<0.0001	1722	-0.09	-0.28	0.10	0.352	1722	0.44	0.25	0.63	<0.0001	<0.0001
Total cholesterol in large HDL (mmol/l)	1722	-0.64	-0.80	-0.47	<0.0001	1722	-0.07	-0.27	0.13	0.473	1722	0.45	0.26	0.64	<0.0001	<0.0001
Cholesterol esters in large HDL (mmol/l)	1722	-0.64	-0.81	-0.48	<0.0001	1722	-0.07	-0.27	0.12	0.469	1722	0.45	0.26	0.64	<0.0001	<0.0001
Free cholesterol in large HDL (mmol/l)	1722	-0.63	-0.79	-0.46	<0.0001	1722	-0.07	-0.27	0.13	0.481	1722	0.45	0.27	0.64	<0.0001	<0.0001
Triglycerides in large HDL (mmol/l)	1722	-0.10	-0.27	0.07	0.233	1722	-0.12	-0.31	0.07	0.218	1722	0.21	0.03	0.40	0.023	0.060
Concentration of medium HDL particles (mol/l)	1722	-0.15	-0.35	0.05	0.151	1722	-0.16	-0.37	0.04	0.116	1722	0.25	0.05	0.46	0.017	0.032
Total lipids in medium HDL (mmol/l)	1722	-0.18	-0.37	0.02	0.074	1722	-0.16	-0.37	0.05	0.124	1722	0.27	0.06	0.48	0.012	0.019
Phospholipids in medium HDL (mmol/l)	1722	-0.21	-0.41	-0.01	0.039	1722	-0.14	-0.35	0.06	0.163	1722	0.28	0.06	0.49	0.011	0.015
Total cholesterol in medium HDL (mmol/l)	1722	-0.22	-0.41	-0.03	0.026	1722	-0.16	-0.38	0.06	0.143	1722	0.28	0.07	0.49	0.009	0.010
Cholesterol esters in medium HDL (mmol/l)	1722	-0.22	-0.41	-0.03	0.022	1722	-0.16	-0.39	0.06	0.147	1722	0.28	0.07	0.49	0.011	0.010
Free cholesterol in medium HDL (mmol/l)	1722	-0.17	-0.35	0.02	0.083	1722	-0.17	-0.38	0.04	0.122	1722	0.28	0.08	0.48	0.005	0.010
Triglycerides in medium HDL (mmol/l)	1722	0.49	0.30	0.68	<0.0001	1722	-0.12	-0.34	0.09	0.251	1722	-0.13	-0.33	0.06	0.174	0.0001
Concentration of small HDL particles (mol/l)	1722	0.18	-0.02	0.39	0.079	1722	-0.15	-0.37	0.06	0.155	1722	0.02	-0.20	0.23	0.858	0.187
Total lipids in small HDL (mmol/l)	1722	0.03	-0.17	0.23	0.734	1722	-0.17	-0.38	0.03	0.098	1722	0.14	-0.07	0.35	0.180	0.200
Phospholipids in small HDL (mmol/l)	1722	0.15	-0.06	0.36	0.157	1722	-0.12	-0.35	0.10	0.269	1722	-0.01	-0.24	0.21	0.900	0.345

Online Table 4 Associations of regional fat indexes at age 18y with cardiometabolic traits at age 18y in ALSPAC

	At age 18y															
	**Trunk fat index (per 2.0 kg/m^2 higher)** *Adj. for age, sex, ethnicity, maternal education, smoking, alcohol, puberty timing, arm fat index, leg fat index*					**Arm fat index (per 0.3 kg/m^2 higher)** *Adj. for age, sex, ethnicity, maternal education, smoking, alcohol, puberty timing, trunk fat index, leg fat index*					**Leg fat index (per 1.3 kg/m^2 higher)** *Adj. for age, sex, ethnicity, maternal education, smoking, alcohol, puberty timing, trunk fat index, arm fat index*					
**Standardized outcome at age 18y**	**N**	**Beta**	**LCL**	**UCL**	**P-value**	**N**	**Beta**	**LCL**	**UCL**	**P-value**	**N**	**Beta**	**LCL**	**UCL**	**P-value**	***P-value for regional heterogeneity***
Total cholesterol in small HDL (mmol/l)	1722	-0.16	-0.34	0.02	0.088	1722	-0.19	-0.39	0.00	0.050	1722	0.32	0.13	0.51	0.001	0.002
Cholesterol esters in small HDL (mmol/l)	1722	-0.16	-0.33	0.01	0.066	1722	-0.19	-0.38	0.00	0.048	1722	0.34	0.16	0.51	0.0002	0.001
Free cholesterol in small HDL (mmol/l)	1722	-0.10	-0.31	0.12	0.373	1722	-0.14	-0.36	0.08	0.223	1722	0.14	-0.09	0.37	0.231	0.317
Triglycerides in small HDL (mmol/l)	1722	0.39	0.20	0.59	<0.0001	1722	-0.05	-0.29	0.19	0.697	1722	-0.18	-0.39	0.04	0.107	0.002
Phospholipids to total lipds ratio in chylomicrons and extremely large VLDL (%)	1722	-0.01	-0.09	0.07	0.766	1722	-0.03	-0.14	0.09	0.635	1722	0.07	-0.02	0.17	0.136	0.420
Total cholesterol to total lipids ratio in chylomicrons and extremely large VLDL (%)	1722	0.23	0.06	0.39	0.007	1722	-0.15	-0.39	0.09	0.216	1722	0.05	-0.18	0.27	0.690	0.069
Cholesterol esters to total lipids ratio in chylomicrons and extremely large VLDL (%)	1722	0.18	0.01	0.34	0.036	1722	-0.16	-0.41	0.09	0.220	1722	0.06	-0.18	0.30	0.611	0.150
Free cholesterol to total lipids ratio in chylomicrons and extremely large VLDL (%)	1722	0.22	0.07	0.38	0.005	1722	0.00	-0.21	0.22	0.998	1722	-0.07	-0.25	0.11	0.452	0.076
Triglycerides to total lipids ratio in chylomicrons and extremely large VLDL (%)	1722	-0.19	-0.33	-0.06	0.006	1722	0.12	-0.08	0.33	0.246	1722	-0.04	-0.23	0.15	0.688	0.073
Phospholipids to total lipds ratio in very large VLDL (%)	1722	0.32	0.17	0.46	<0.0001	1722	0.06	-0.14	0.25	0.569	1722	-0.15	-0.32	0.01	0.071	0.001
Total cholesterol to total lipids ratio in very large VLDL (%)	1722	-0.20	-0.34	-0.06	0.006	1722	0.06	-0.15	0.26	0.579	1722	0.10	-0.07	0.27	0.238	0.023
Cholesterol esters to total lipids ratio in very large VLDL (%)	1722	-0.14	-0.25	-0.03	0.014	1722	0.04	-0.13	0.21	0.679	1722	0.06	-0.08	0.20	0.402	0.075
Free cholesterol to total lipids ratio in very large VLDL (%)	1722	-0.14	-0.26	-0.02	0.020	1722	0.06	-0.08	0.21	0.389	1722	0.09	-0.03	0.21	0.158	0.050
Triglycerides to total lipids ratio in very large VLDL (%)	1722	0.09	-0.06	0.24	0.229	1722	-0.08	-0.29	0.13	0.444	1722	-0.06	-0.23	0.12	0.523	0.397
Phospholipids to total lipds ratio in large VLDL (%)	1722	0.32	0.18	0.46	<0.0001	1722	-0.01	-0.21	0.19	0.911	1722	-0.16	-0.33	0.01	0.060	0.0003
Total cholesterol to total lipids ratio in large VLDL (%)	1722	0.23	0.08	0.37	0.002	1722	0.03	-0.18	0.24	0.760	1722	-0.09	-0.28	0.10	0.334	0.049
Cholesterol esters to total lipids ratio in large VLDL (%)	1722	-0.04	-0.18	0.10	0.554	1722	0.08	-0.14	0.30	0.484	1722	0.03	-0.17	0.23	0.793	0.684
Free cholesterol to total lipids ratio in large VLDL (%)	1722	0.20	0.13	0.27	<0.0001	1722	-0.02	-0.11	0.07	0.713	1722	-0.10	-0.17	-0.02	0.014	<0.0001
Triglycerides to total lipids ratio in large VLDL (%)	1722	-0.29	-0.43	-0.15	<0.0001	1722	-0.02	-0.22	0.19	0.876	1722	0.13	-0.05	0.31	0.146	0.002
Phospholipids to total lipds ratio in medium VLDL (%)	1722	-0.48	-0.64	-0.31	<0.0001	1722	0.01	-0.18	0.21	0.906	1722	0.31	0.14	0.48	0.0004	<0.0001
Total cholesterol to total lipids ratio in medium VLDL (%)	1722	0.05	-0.11	0.22	0.539	1722	0.07	-0.14	0.28	0.494	1722	-0.02	-0.20	0.17	0.854	0.845
Cholesterol esters to total lipids ratio in medium VLDL (%)	1722	0.00	-0.18	0.17	0.985	1722	0.08	-0.14	0.29	0.482	1722	0.02	-0.17	0.20	0.847	0.907
Free cholesterol to total lipids ratio in medium VLDL (%)	1722	0.19	0.04	0.35	0.014	1722	0.03	-0.19	0.25	0.803	1722	-0.12	-0.31	0.07	0.215	0.065
Triglycerides to total lipids ratio in medium VLDL (%)	1722	0.05	-0.12	0.21	0.585	1722	-0.07	-0.28	0.14	0.516	1722	-0.04	-0.23	0.14	0.643	0.730
Phospholipids to total lipds ratio in small VLDL (%)	1722	-0.50	-0.67	-0.32	<0.0001	1722	-0.18	-0.40	0.05	0.119	1722	0.40	0.20	0.61	0.0001	<0.0001
Total cholesterol to total lipids ratio in small VLDL (%)	1722	-0.20	-0.38	-0.02	0.029	1722	0.18	-0.04	0.40	0.109	1722	0.06	-0.12	0.25	0.496	0.066
Cholesterol esters to total lipids ratio in small VLDL (%)	1722	-0.13	-0.31	0.05	0.150	1722	0.20	-0.02	0.42	0.069	1722	0.00	-0.18	0.19	0.969	0.180
Free cholesterol to total lipids ratio in small VLDL (%)	1722	-0.48	-0.66	-0.30	<0.0001	1722	-0.13	-0.33	0.08	0.221	1722	0.41	0.23	0.58	<0.0001	<0.0001
Triglycerides to total lipids ratio in small VLDL (%)	1722	0.37	0.19	0.55	<0.0001	1722	-0.12	-0.35	0.10	0.266	1722	-0.20	-0.38	-0.01	0.037	0.0002
Phospholipids to total lipds ratio in very small VLDL (%)	1722	-0.15	-0.32	0.01	0.068	1722	-0.06	-0.24	0.13	0.535	1722	0.23	0.06	0.40	0.007	0.017
Total cholesterol to total lipids ratio in very small VLDL (%)	1722	-0.09	-0.28	0.10	0.340	1722	0.15	-0.06	0.37	0.169	1722	-0.07	-0.25	0.11	0.422	0.357
Cholesterol esters to total lipids ratio in very small VLDL (%)	1722	0.06	-0.11	0.23	0.494	1722	0.17	-0.03	0.37	0.095	1722	-0.18	-0.35	0.00	0.045	0.082
Free cholesterol to total lipids ratio in very small VLDL (%)	1722	-0.54	-0.81	-0.27	0.0001	1722	-0.01	-0.29	0.26	0.919	1722	0.33	0.12	0.54	0.002	<0.0001
Triglycerides to total lipids ratio in very small VLDL (%)	1722	0.22	0.03	0.40	0.026	1722	-0.14	-0.37	0.08	0.210	1722	-0.07	-0.26	0.12	0.476	0.085
Phospholipids to total lipds ratio in IDL (%)	1722	-0.43	-0.66	-0.21	0.0002	1722	-0.11	-0.35	0.13	0.378	1722	0.30	0.09	0.52	0.005	0.0003
Total cholesterol to total lipids ratio in IDL (%)	1722	0.22	0.03	0.41	0.024	1722	0.15	-0.05	0.36	0.147	1722	-0.19	-0.38	-0.01	0.040	0.019
Cholesterol esters to total lipids ratio in IDL (%)	1722	0.45	0.25	0.64	<0.0001	1722	0.14	-0.06	0.34	0.179	1722	-0.34	-0.52	-0.16	0.0003	<0.0001
Free cholesterol to total lipids ratio in IDL (%)	1722	-0.52	-0.68	-0.35	<0.0001	1722	0.05	-0.16	0.25	0.661	1722	0.33	0.14	0.52	0.001	<0.0001
Triglycerides to total lipids ratio in IDL (%)	1722	-0.09	-0.26	0.09	0.345	1722	-0.13	-0.34	0.08	0.213	1722	0.10	-0.09	0.29	0.291	0.330
Phospholipids to total lipds ratio in large LDL (%)	1722	0.06	-0.10	0.21	0.476	1722	-0.13	-0.32	0.07	0.206	1722	-0.03	-0.22	0.15	0.720	0.483
Total cholesterol to total lipids ratio in large LDL (%)	1722	0.07	-0.09	0.23	0.379	1722	0.16	-0.03	0.36	0.100	1722	-0.06	-0.24	0.12	0.510	0.400
Cholesterol esters to total lipids ratio in large LDL (%)	1722	0.19	0.04	0.35	0.013	1722	0.15	-0.04	0.34	0.130	1722	-0.11	-0.30	0.07	0.216	0.077
Free cholesterol to total lipids ratio in large LDL (%)	1722	-0.40	-0.55	-0.25	<0.0001	1722	-0.03	-0.25	0.18	0.765	1722	0.19	0.00	0.38	0.052	<0.0001
Triglycerides to total lipids ratio in large LDL (%)	1722	-0.18	-0.36	-0.01	0.040	1722	-0.12	-0.32	0.08	0.248	1722	0.14	-0.05	0.33	0.148	0.100
Phospholipids to total lipds ratio in medium LDL (%)	1722	0.05	-0.10	0.21	0.503	1722	-0.13	-0.32	0.06	0.180	1722	-0.02	-0.20	0.16	0.850	0.491
Total cholesterol to total lipids ratio in medium LDL (%)	1722	0.04	-0.12	0.19	0.638	1722	0.17	-0.02	0.36	0.074	1722	-0.07	-0.25	0.11	0.448	0.353
Cholesterol esters to total lipids ratio in medium LDL (%)	1722	0.08	-0.07	0.23	0.300	1722	0.15	-0.05	0.34	0.136	1722	-0.06	-0.24	0.12	0.509	0.431
Free cholesterol to total lipids ratio in medium LDL (%)	1722	-0.13	-0.27	0.02	0.090	1722	-0.08	-0.27	0.11	0.397	1722	0.04	-0.14	0.21	0.668	0.457
Triglycerides to total lipids ratio in medium LDL (%)	1722	-0.19	-0.35	-0.03	0.020	1722	-0.12	-0.30	0.06	0.199	1722	0.19	0.02	0.35	0.024	0.016

Online Table 4 Associations of regional fat indexes at age 18y with cardiometabolic traits at age 18y in ALSPAC

At age 18y

	Trunk fat index (per 2.0 kg/m^2 higher) Adj. for age, sex, ethnicity, maternal education, smoking, alcohol, puberty timing, arm fat index, leg fat index					Arm fat index (per 0.3 kg/m^2 higher) Adj. for age, sex, ethnicity, maternal education, smoking, alcohol, puberty timing, trunk fat index, leg fat index					Leg fat index (per 1.3 kg/m^2 higher) Adj. for age, sex, ethnicity, maternal education, smoking, alcohol, puberty timing, trunk fat index, arm fat index					
**Standardized outcome at age 18y**	**N**	**Beta**	**LCL**	**UCL**	**P-value**	**N**	**Beta**	**LCL**	**UCL**	**P-value**	**N**	**Beta**	**LCL**	**UCL**	**P-value**	***P-value for regional heterogeneity***
Phospholipids to total lipds ratio in small LDL (%)	1722	-0.01	-0.16	0.15	0.927	1722	-0.13	-0.32	0.06	0.170	1722	0.00	-0.18	0.18	0.978	0.669
Total cholesterol to total lipids ratio in small LDL (%)	1722	-0.04	-0.20	0.12	0.606	1722	0.16	-0.03	0.36	0.095	1722	0.00	-0.18	0.18	0.998	0.417
Cholesterol esters to total lipids ratio in small LDL (%)	1722	0.03	-0.13	0.18	0.735	1722	0.13	-0.06	0.32	0.183	1722	-0.01	-0.18	0.17	0.955	0.710
Free cholesterol to total lipids ratio in small LDL (%)	1722	-0.14	-0.30	0.02	0.088	1722	-0.05	-0.24	0.15	0.649	1722	0.01	-0.16	0.19	0.885	0.535
Triglycerides to total lipids ratio in small LDL (%)	1722	0.14	-0.03	0.32	0.110	1722	-0.12	-0.34	0.10	0.281	1722	0.00	-0.20	0.19	0.968	0.293
Phospholipids to total lipds ratio in very large HDL (%)	1722	-0.57	-0.73	-0.42	<0.0001	1722	-0.18	-0.35	-0.01	0.043	1722	0.51	0.34	0.68	<0.0001	<0.0001
Total cholesterol to total lipids ratio in very large HDL (%)	1722	0.51	0.35	0.67	<0.0001	1722	0.19	0.02	0.35	0.028	1722	-0.48	-0.65	-0.32	<0.0001	<0.0001
Cholesterol esters to total lipids ratio in very large HDL (%)	1722	0.51	0.35	0.67	<0.0001	1722	0.17	0.01	0.34	0.042	1722	-0.48	-0.64	-0.31	<0.0001	<0.0001
Free cholesterol to total lipids ratio in very large HDL (%)	1722	-0.14	-0.34	0.05	0.155	1722	0.08	-0.14	0.31	0.467	1722	0.05	-0.15	0.25	0.619	0.388
Triglycerides to total lipids ratio in very large HDL (%)	1722	0.45	0.23	0.66	<0.0001	1722	0.00	-0.25	0.25	0.997	1722	-0.22	-0.49	0.05	0.107	0.003
Phospholipids to total lipds ratio in large HDL (%)	1722	0.62	0.44	0.81	<0.0001	1722	-0.02	-0.27	0.22	0.847	1722	-0.26	-0.47	-0.04	0.019	<0.0001
Total cholesterol to total lipids ratio in large HDL (%)	1722	-0.67	-0.86	-0.48	<0.0001	1722	0.00	-0.24	0.24	0.998	1722	0.31	0.09	0.54	0.007	<0.0001
Cholesterol esters to total lipids ratio in large HDL (%)	1722	-0.66	-0.86	-0.47	<0.0001	1722	0.00	-0.25	0.25	0.988	1722	0.30	0.06	0.53	0.014	<0.0001
Free cholesterol to total lipids ratio in large HDL (%)	1722	-0.54	-0.72	-0.36	<0.0001	1722	-0.01	-0.22	0.21	0.953	1722	0.31	0.12	0.50	0.002	<0.0001
Triglycerides to total lipids ratio in large HDL (%)	1722	0.61	0.41	0.80	<0.0001	1722	0.03	-0.20	0.27	0.775	1722	-0.34	-0.59	-0.09	0.007	<0.0001
Phospholipids to total lipds ratio in medium HDL (%)	1722	-0.23	-0.42	-0.04	0.018	1722	0.04	-0.20	0.28	0.718	1722	0.12	-0.08	0.32	0.257	0.078
Total cholesterol to total lipids ratio in medium HDL (%)	1722	-0.10	-0.28	0.09	0.298	1722	-0.03	-0.28	0.22	0.798	1722	0.04	-0.17	0.24	0.737	0.704
Cholesterol esters to total lipids ratio in medium HDL (%)	1722	-0.10	-0.29	0.08	0.268	1722	-0.03	-0.28	0.22	0.810	1722	0.02	-0.19	0.23	0.852	0.737
Free cholesterol to total lipids ratio in medium HDL (%)	1722	0.01	-0.16	0.18	0.892	1722	-0.06	-0.26	0.15	0.573	1722	0.12	-0.06	0.31	0.192	0.553
Triglycerides to total lipids ratio in medium HDL (%)	1722	0.59	0.37	0.80	<0.0001	1722	-0.01	-0.26	0.24	0.948	1722	-0.27	-0.52	-0.02	0.035	<0.0001
Phospholipids to total lipds ratio in small HDL (%)	1722	0.23	0.07	0.40	0.005	1722	0.12	-0.07	0.30	0.215	1722	-0.34	-0.51	-0.18	<0.0001	<0.0001
Total cholesterol to total lipids ratio in small HDL (%)	1722	-0.34	-0.49	-0.18	<0.0001	1722	-0.14	-0.34	0.06	0.170	1722	0.41	0.24	0.58	<0.0001	<0.0001
Cholesterol esters to total lipids ratio in small HDL (%)	1722	-0.26	-0.42	-0.10	0.001	1722	-0.13	-0.33	0.06	0.178	1722	0.38	0.21	0.55	<0.0001	<0.0001
Free cholesterol to total lipids ratio in small HDL (%)	1722	-0.34	-0.54	-0.14	0.001	1722	0.05	-0.17	0.28	0.655	1722	0.01	-0.21	0.23	0.954	0.066
Triglycerides to total lipids ratio in small HDL (%)	1722	0.39	0.19	0.60	0.0002	1722	0.08	-0.21	0.37	0.600	1722	-0.27	-0.55	0.01	0.060	0.005
Mean diameter for VLDL particles (nm)	1722	0.72	0.56	0.89	<0.0001	1722	-0.02	-0.23	0.20	0.888	1722	-0.43	-0.62	-0.24	<0.0001	<0.0001
Mean diameter for LDL particles (nm)	1722	-0.22	-0.38	-0.05	0.010	1722	-0.05	-0.24	0.15	0.636	1722	0.10	-0.07	0.28	0.250	0.085
Mean diameter for HDL particles (nm)	1722	-0.65	-0.81	-0.50	<0.0001	1722	0.00	-0.18	0.19	0.975	1722	0.42	0.24	0.60	<0.0001	<0.0001
Serum total cholesterol (mmol/l)	1722	0.03	-0.16	0.21	0.779	1722	0.01	-0.21	0.22	0.943	1722	0.12	-0.07	0.31	0.205	0.754
Total cholesterol in VLDL (mmol/l)	1722	0.57	0.39	0.76	<0.0001	1722	0.05	-0.19	0.29	0.681	1722	-0.28	-0.50	-0.06	0.013	<0.0001
Remnant cholesterol (non-HDL, non-LDL -cholesterol) (mmol/l)	1722	0.39	0.20	0.58	<0.0001	1722	0.05	-0.19	0.29	0.695	1722	-0.14	-0.35	0.07	0.201	0.006
Total cholesterol in LDL (mmol/l)	1722	0.08	-0.11	0.27	0.423	1722	0.03	-0.20	0.25	0.827	1722	0.08	-0.12	0.27	0.442	0.956
Total cholesterol in HDL (mmol/l)	1722	-0.51	-0.68	-0.34	<0.0001	1722	-0.07	-0.27	0.12	0.465	1722	0.40	0.20	0.60	<0.0001	<0.0001
Total cholesterol in HDL2 (mmol/l)	1722	-0.56	-0.74	-0.39	<0.0001	1722	-0.07	-0.27	0.13	0.474	1722	0.42	0.22	0.62	<0.0001	<0.0001
Total cholesterol in HDL3 (mmol/l)	1722	-0.39	-0.56	-0.22	<0.0001	1722	-0.07	-0.26	0.12	0.478	1722	0.34	0.15	0.53	0.0003	<0.0001
Esterified cholesterol (mmol/l)	1722	0.04	-0.14	0.23	0.638	1722	0.04	-0.18	0.25	0.747	1722	0.07	-0.12	0.26	0.478	0.979
Free cholesterol (mmol/l)	1722	-0.02	-0.20	0.16	0.842	1722	-0.06	-0.27	0.15	0.590	1722	0.23	0.05	0.41	0.012	0.131
Serum total triglycerides (mmol/l)	1722	0.59	0.41	0.77	<0.0001	1722	0.02	-0.23	0.27	0.885	1722	-0.32	-0.54	-0.09	0.006	<0.0001
Triglycerides in VLDL (mmol/l)	1722	0.69	0.51	0.87	<0.0001	1722	0.03	-0.21	0.28	0.784	1722	-0.40	-0.62	-0.18	0.0004	<0.0001
Triglycerides in LDL (mmol/l)	1722	-0.06	-0.26	0.14	0.544	1722	-0.02	-0.25	0.21	0.853	1722	0.13	-0.07	0.32	0.202	0.489
Triglycerides in HDL (mmol/l)	1722	0.31	0.12	0.50	0.001	1722	-0.07	-0.29	0.16	0.552	1722	-0.08	-0.28	0.13	0.463	0.035
Diacylglycerol (mmol/l)	1722	0.35	0.14	0.56	0.001	1722	0.04	-0.20	0.29	0.727	1722	-0.17	-0.38	0.04	0.123	0.011
Ratio of diacylglycerol to triglycerides	1722	0.09	-0.09	0.27	0.333	1722	0.02	-0.18	0.22	0.857	1722	0.00	-0.18	0.17	0.974	0.824
Total phosphoglycerides (mmol/l)	1722	-0.16	-0.34	0.02	0.084	1722	-0.02	-0.23	0.19	0.849	1722	0.20	0.01	0.38	0.037	0.067
Ratio of triglycerides to phosphoglycerides	1722	0.69	0.51	0.87	<0.0001	1722	-0.03	-0.27	0.21	0.786	1722	-0.37	-0.59	-0.15	0.001	<0.0001
Phosphatidylcholine and other cholines (mmol/l)	1722	-0.12	-0.30	0.06	0.201	1722	-0.07	-0.27	0.13	0.495	1722	0.18	0.00	0.36	0.054	0.123
Total cholines (mmol/l)	1722	-0.17	-0.35	0.01	0.067	1722	-0.04	-0.25	0.16	0.681	1722	0.22	0.04	0.40	0.014	0.028
Apolipoprotein A-I (g/l)	1722	-0.31	-0.48	-0.14	0.0004	1722	-0.08	-0.26	0.11	0.420	1722	0.31	0.13	0.50	0.001	0.0002
Apolipoprotein B (g/l)	1722	0.45	0.26	0.64	<0.0001	1722	0.04	-0.21	0.28	0.774	1722	-0.18	-0.40	0.04	0.104	0.001

Online Table 4 Associations of regional fat indexes at age 18y with cardiometabolic traits at age 18y in ALSPAC

	**At age 18y**															
	**Trunk fat index (per 2.0 kg/m^2 higher)**					**Arm fat index (per 0.3 kg/m^2 higher)**					**Leg fat index (per 1.3 kg/m^2 higher)**					
	Adj. for age, sex, ethnicity, maternal education, smoking, alcohol, puberty timing, arm fat index, leg fat index					*Adj. for age, sex, ethnicity, maternal education, smoking, alcohol, puberty timing, trunk fat index, leg fat index*					*Adj. for age, sex, ethnicity, maternal education, smoking, alcohol, puberty timing, trunk fat index, arm fat index*					***P-value for regional heterogeneity***
**Standardized outcome at age 18y**	**N**	**Beta**	**LCL**	**UCL**	**P-value**	**N**	**Beta**	**LCL**	**UCL**	**P-value**	**N**	**Beta**	**LCL**	**UCL**	**P-value**	
Ratio of apolipoprotein B to apolipoprotein A-I	1722	0.59	0.40	0.79	<0.0001	1722	0.08	-0.17	0.32	0.540	1722	-0.33	-0.56	-0.09	0.008	<0.0001
Total fatty acids (mmol/l)	1722	0.22	0.03	0.40	0.021	1722	-0.02	-0.25	0.21	0.876	1722	-0.01	-0.21	0.20	0.944	0.274
Estimated description of fatty acid chain length, not actual carbon number	1722	0.11	-0.05	0.27	0.179	1722	-0.07	-0.26	0.13	0.516	1722	-0.04	-0.20	0.13	0.662	0.427
Estimated degree of unsaturation	1722	-0.20	-0.37	-0.04	0.015	1722	-0.06	-0.26	0.14	0.571	1722	0.21	0.03	0.38	0.019	0.012
22:6, docosahexaenoic acid (mmol/l)	1722	-0.07	-0.26	0.12	0.475	1722	0.07	-0.14	0.29	0.489	1722	0.05	-0.13	0.24	0.566	0.660
18:2, linoleic acid (mmol/l)	1722	0.08	-0.09	0.26	0.352	1722	-0.12	-0.33	0.09	0.281	1722	0.13	-0.05	0.31	0.162	0.358
Conjugated linoleic acid (mmol/l)	1722	0.14	-0.04	0.31	0.130	1722	0.15	-0.05	0.36	0.144	1722	-0.15	-0.35	0.04	0.129	0.136
Omega-3 fatty acids (mmol/l)	1722	0.08	-0.10	0.25	0.401	1722	0.10	-0.12	0.31	0.383	1722	-0.04	-0.22	0.15	0.702	0.669
Omega-6 fatty acids (mmol/l)	1722	0.07	-0.11	0.25	0.438	1722	-0.08	-0.30	0.13	0.464	1722	0.14	-0.05	0.32	0.150	0.479
Polyunsaturated fatty acids (mmol/l)	1722	0.07	-0.10	0.25	0.415	1722	-0.06	-0.27	0.16	0.591	1722	0.12	-0.07	0.30	0.211	0.607
Monounsaturated fatty acids; 16:1, 18:1 (mmol/l)	1722	0.35	0.18	0.53	<0.0001	1722	-0.05	-0.29	0.18	0.663	1722	-0.07	-0.28	0.14	0.519	0.009
Saturated fatty acids (mmol/l)	1722	0.17	-0.02	0.36	0.078	1722	0.05	-0.18	0.28	0.672	1722	-0.06	-0.26	0.15	0.592	0.392
Ratio of 22:6 docosahexaenoic acid to total fatty acids (%)	1722	-0.21	-0.39	-0.04	0.016	1722	0.10	-0.09	0.29	0.287	1722	0.08	-0.10	0.25	0.374	0.075
Ratio of 18:2 linoleic acid to total fatty acids (%)	1722	-0.26	-0.43	-0.09	0.003	1722	-0.12	-0.33	0.08	0.237	1722	0.20	0.03	0.38	0.024	0.005
Ratio of conjugated linoleic acid to total fatty acids (%)	1722	0.10	-0.05	0.26	0.196	1722	0.12	-0.06	0.30	0.180	1722	-0.12	-0.30	0.06	0.191	0.233
Ratio of omega-3 fatty acids to total fatty acids (%)	1722	-0.09	-0.25	0.08	0.320	1722	0.14	-0.07	0.35	0.190	1722	-0.05	-0.23	0.13	0.572	0.397
Ratio of omega-6 fatty acids to total fatty acids (%)	1722	-0.36	-0.53	-0.19	<0.0001	1722	-0.07	-0.29	0.15	0.526	1722	0.27	0.08	0.45	0.004	<0.0001
Ratio of polyunsaturated fatty acids to total fatty acids (%)	1722	-0.36	-0.53	-0.19	<0.0001	1722	-0.03	-0.25	0.18	0.766	1722	0.24	0.05	0.42	0.011	0.0002
Ratio of monounsaturated fatty acids to total fatty acids (%)	1722	0.42	0.26	0.58	<0.0001	1722	-0.10	-0.31	0.11	0.356	1722	-0.14	-0.32	0.04	0.138	<0.0001
Ratio of saturated fatty acids to total fatty acids (%)	1722	-0.14	-0.31	0.03	0.097	1722	0.17	-0.03	0.38	0.102	1722	-0.11	-0.28	0.07	0.234	0.162
Insulin (mu/l)	1722	0.53	0.17	0.88	0.004	1722	-0.23	-0.56	0.10	0.173	1722	0.04	-0.16	0.24	0.718	0.036
Glucose (mmol/l)	1722	0.19	0.06	0.32	0.004	1722	-0.04	-0.18	0.10	0.564	1722	-0.04	-0.17	0.09	0.521	0.065
Lactate (mmol/l)	1722	-0.17	-0.34	0.01	0.063	1722	0.22	0.02	0.41	0.030	1722	0.01	-0.17	0.19	0.930	0.071
Pyruvate (mmol/l)	1722	0.08	-0.09	0.24	0.350	1722	0.24	0.06	0.43	0.011	1722	-0.17	-0.34	0.00	0.054	0.032
Citrate (mmol/l)	1722	-0.20	-0.37	-0.03	0.020	1722	-0.06	-0.26	0.14	0.555	1722	0.13	-0.03	0.30	0.117	0.049
Alanine (mmol/l)	1722	0.33	0.16	0.50	0.0002	1722	-0.09	-0.29	0.11	0.394	1722	-0.16	-0.34	0.03	0.095	0.002
Glutamine (mmol/l)	1722	0.14	-0.02	0.29	0.081	1722	-0.05	-0.24	0.14	0.621	1722	-0.13	-0.29	0.03	0.116	0.105
Histidine (mmol/l)	1722	0.24	0.07	0.41	0.005	1722	-0.18	-0.40	0.04	0.106	1722	-0.04	-0.21	0.13	0.638	0.028
Isoleucine (mmol/l)	1722	0.47	0.31	0.62	<0.0001	1722	0.09	-0.10	0.28	0.348	1722	-0.35	-0.52	-0.18	<0.0001	<0.0001
Leucine (mmol/l)	1722	0.35	0.21	0.49	<0.0001	1722	-0.01	-0.18	0.16	0.917	1722	-0.21	-0.36	-0.07	0.005	<0.0001
Valine (mmol/l)	1722	0.26	0.12	0.41	0.0004	1722	0.16	-0.01	0.34	0.063	1722	-0.21	-0.37	-0.05	0.010	0.001
Phenylalanine (mmol/l)	1722	0.35	0.19	0.51	<0.0001	1722	-0.22	-0.41	-0.02	0.027	1722	0.05	-0.12	0.22	0.562	0.001
Tyrosine (mmol/l)	1722	0.35	0.20	0.51	<0.0001	1722	-0.02	-0.21	0.16	0.810	1722	-0.04	-0.22	0.14	0.652	0.005
Acetate (mmol/l)	1722	-0.12	-0.19	-0.06	0.0003	1722	0.02	-0.06	0.10	0.599	1722	0.05	-0.02	0.12	0.139	0.004
Acetoacetate (mmol/l)	1722	-0.22	-0.39	-0.04	0.018	1722	0.07	-0.14	0.29	0.494	1722	0.09	-0.07	0.25	0.252	0.064
3-hydroxybutyrate (mmol/l)	1722	-0.22	-0.43	-0.01	0.042	1722	0.04	-0.19	0.26	0.743	1722	0.12	-0.05	0.29	0.183	0.105
Creatinine (mmol/l)	1722	0.26	0.13	0.39	0.0001	1722	-0.21	-0.37	-0.06	0.007	1722	-0.08	-0.22	0.07	0.303	0.0003
Albumin (signal area)	1722	0.01	-0.15	0.17	0.923	1722	-0.04	-0.23	0.16	0.702	1722	-0.06	-0.24	0.12	0.506	0.890
Glycoprotein acetyls, mainly a1-acid glycoprotein (mmol/l)	1722	0.47	0.30	0.64	<0.0001	1722	-0.08	-0.28	0.13	0.471	1722	-0.07	-0.24	0.10	0.423	0.0001
C-reactive protein (mg/l)	1722	0.06	-0.05	0.17	0.278	1722	-0.08	-0.20	0.04	0.203	1722	0.11	-0.03	0.26	0.132	0.247

Online Table 5 Associations of lean mass index at age 10y and 18y with cardiometabolic traits at age 18y in ALSPAC

	At age 10y										At age 18y									
	Lean mass index (per 1.0 kg/m^2 higher)										Lean mass index (per 2.2 kg/m^2 higher)									
	Adj. for age, sex, ethnicity, maternal education					*Additionally adj. for fat mass index at 10y*					*Adj. for age, sex, ethnicity, maternal education, smoking, alcohol, puberty timing*					*Additionally adj. for fat mass index at 18y*				
**Standardized outcome at age 18y**	**N**	**Beta**	**LCL**	**UCL**	**P-value**	**N**	**Beta**	**LCL**	**UCL**	**P-value**	**N**	**Beta**	**LCL**	**UCL**	**P-value**	**N**	**Beta**	**LCL**	**UCL**	**P-value**
Systolic blood pressure (mmHg)	3548	0.18	0.14	0.21	<0.0001	3548	0.13	0.10	0.17	<0.0001	3016	0.33	0.28	0.39	<0.0001	3016	0.27	0.22	0.32	<0.0001
Diastolic blood pressure (mmHg)	3548	0.07	0.03	0.11	0.0004	3548	-0.01	-0.05	0.03	0.521	3016	0.05	-0.01	0.12	0.099	3016	-0.04	-0.10	0.02	0.170
Concentration of chylomicrons and extremely large VLDL particles (mol/l)	2440	0.08	0.02	0.13	0.005	2440	0.03	-0.03	0.08	0.324	2058	0.15	0.08	0.23	<0.0001	2058	0.07	0.00	0.14	0.052
Total lipids in chylomicrons and extremely large VLDL (mmol/l)	2440	0.08	0.02	0.13	0.005	2440	0.03	-0.03	0.08	0.314	2058	0.15	0.08	0.23	<0.0001	2058	0.07	0.00	0.14	0.050
Phospholipids in chylomicrons and extremely large VLDL (mmol/l)	2440	0.07	0.02	0.13	0.006	2440	0.03	-0.03	0.08	0.336	2058	0.14	0.07	0.22	0.0002	2058	0.06	-0.01	0.13	0.073
Total cholesterol in chylomicrons and extremely large VLDL (mmol/l)	2440	0.07	0.02	0.13	0.006	2440	0.03	-0.02	0.08	0.296	2058	0.16	0.09	0.24	<0.0001	2058	0.08	0.01	0.15	0.021
Cholesterol esters in chylomicrons and extremely large VLDL (mmol/l)	2440	0.07	0.02	0.13	0.008	2440	0.03	-0.03	0.08	0.309	2058	0.17	0.09	0.25	<0.0001	2058	0.09	0.02	0.17	0.011
Free cholesterol in chylomicrons and extremely large VLDL (mmol/l)	2440	0.08	0.02	0.13	0.005	2440	0.03	-0.02	0.08	0.292	2058	0.15	0.07	0.23	0.0001	2058	0.07	0.00	0.14	0.053
Triglycerides in chylomicrons and extremely large VLDL (mmol/l)	2440	0.08	0.02	0.13	0.005	2440	0.03	-0.03	0.08	0.320	2058	0.15	0.07	0.23	0.0001	2058	0.07	0.00	0.14	0.059
Concentration of very large VLDL particles (mol/l)	2440	0.08	0.03	0.13	0.003	2440	0.03	-0.02	0.08	0.231	2058	0.16	0.09	0.24	<0.0001	2058	0.08	0.01	0.15	0.018
Total lipids in very large VLDL (mmol/l)	2440	0.08	0.03	0.13	0.003	2440	0.03	-0.02	0.08	0.226	2058	0.16	0.09	0.24	<0.0001	2058	0.08	0.01	0.15	0.017
Phospholipids in very large VLDL (mmol/l)	2440	0.08	0.02	0.13	0.005	2440	0.03	-0.02	0.08	0.259	2058	0.16	0.08	0.23	<0.0001	2058	0.08	0.01	0.15	0.029
Total cholesterol in very large VLDL (mmol/l)	2440	0.08	0.02	0.13	0.004	2440	0.03	-0.02	0.08	0.284	2058	0.17	0.09	0.24	<0.0001	2058	0.08	0.01	0.15	0.020
Cholesterol esters in very large VLDL (mmol/l)	2440	0.08	0.03	0.13	0.004	2440	0.03	-0.02	0.08	0.267	2058	0.18	0.10	0.25	<0.0001	2058	0.09	0.02	0.16	0.010
Free cholesterol in very large VLDL (mmol/l)	2440	0.07	0.02	0.13	0.006	2440	0.03	-0.03	0.08	0.309	2058	0.15	0.08	0.23	<0.0001	2058	0.07	0.00	0.14	0.041
Triglycerides in very large VLDL (mmol/l)	2440	0.08	0.03	0.13	0.003	2440	0.03	-0.02	0.08	0.203	2058	0.16	0.09	0.24	<0.0001	2058	0.08	0.02	0.15	0.015
Concentration of large VLDL particles (mol/l)	2440	0.08	0.03	0.13	0.003	2440	0.03	-0.02	0.08	0.221	2058	0.16	0.09	0.24	<0.0001	2058	0.09	0.02	0.15	0.011
Total lipids in large VLDL (mmol/l)	2440	0.08	0.03	0.13	0.003	2440	0.03	-0.02	0.08	0.219	2058	0.16	0.09	0.24	<0.0001	2058	0.09	0.02	0.15	0.010
Phospholipids in large VLDL (mmol/l)	2440	0.08	0.03	0.13	0.004	2440	0.03	-0.02	0.08	0.227	2058	0.16	0.09	0.23	<0.0001	2058	0.08	0.02	0.15	0.013
Total cholesterol in large VLDL (mmol/l)	2440	0.08	0.02	0.13	0.004	2440	0.03	-0.02	0.08	0.235	2058	0.17	0.10	0.24	<0.0001	2058	0.09	0.02	0.16	0.009
Cholesterol esters in large VLDL (mmol/l)	2440	0.08	0.02	0.13	0.004	2440	0.03	-0.02	0.08	0.244	2058	0.18	0.10	0.25	<0.0001	2058	0.10	0.03	0.16	0.006
Free cholesterol in large VLDL (mmol/l)	2440	0.08	0.02	0.13	0.004	2440	0.03	-0.02	0.08	0.230	2058	0.16	0.09	0.24	<0.0001	2058	0.08	0.02	0.15	0.014
Triglycerides in large VLDL (mmol/l)	2440	0.08	0.03	0.13	0.003	2440	0.03	-0.02	0.08	0.213	2058	0.16	0.09	0.24	<0.0001	2058	0.09	0.02	0.15	0.011
Concentration of medium VLDL particles (mol/l)	2440	0.08	0.02	0.13	0.004	2440	0.03	-0.02	0.08	0.254	2058	0.17	0.10	0.24	<0.0001	2058	0.09	0.02	0.15	0.009
Total lipids in medium VLDL (mmol/l)	2440	0.08	0.03	0.13	0.003	2440	0.03	-0.02	0.08	0.245	2058	0.17	0.10	0.24	<0.0001	2058	0.09	0.02	0.16	0.007
Phospholipids in medium VLDL (mmol/l)	2440	0.07	0.02	0.13	0.005	2440	0.03	-0.02	0.08	0.262	2058	0.16	0.09	0.24	<0.0001	2058	0.09	0.02	0.15	0.012
Total cholesterol in medium VLDL (mmol/l)	2440	0.07	0.02	0.13	0.005	2440	0.03	-0.02	0.08	0.253	2058	0.17	0.10	0.24	<0.0001	2058	0.09	0.02	0.16	0.009
Cholesterol esters in medium VLDL (mmol/l)	2440	0.07	0.02	0.12	0.007	2440	0.03	-0.02	0.08	0.269	2058	0.17	0.10	0.25	<0.0001	2058	0.10	0.02	0.17	0.008
Free cholesterol in medium VLDL (mmol/l)	2440	0.07	0.02	0.12	0.006	2440	0.03	-0.02	0.08	0.262	2058	0.16	0.09	0.23	<0.0001	2058	0.08	0.02	0.15	0.016
Triglycerides in medium VLDL (mmol/l)	2440	0.08	0.03	0.13	0.003	2440	0.03	-0.02	0.08	0.249	2058	0.17	0.10	0.24	<0.0001	2058	0.09	0.02	0.16	0.007
Concentration of small VLDL particles (mol/l)	2440	0.06	0.01	0.11	0.022	2440	0.02	-0.03	0.07	0.466	2058	0.14	0.07	0.21	0.0001	2058	0.07	0.00	0.14	0.046
Total lipids in small VLDL (mmol/l)	2440	0.05	0.00	0.10	0.039	2440	0.01	-0.04	0.06	0.648	2058	0.13	0.06	0.21	0.0003	2058	0.06	-0.01	0.13	0.084
Phospholipids in small VLDL (mmol/l)	2440	0.05	0.00	0.10	0.062	2440	0.01	-0.04	0.06	0.712	2058	0.12	0.05	0.19	0.001	2058	0.06	-0.01	0.12	0.099
Total cholesterol in small VLDL (mmol/l)	2440	0.04	-0.01	0.09	0.139	2440	0.00	-0.05	0.05	0.949	2058	0.11	0.04	0.18	0.003	2058	0.04	-0.03	0.11	0.280
Cholesterol esters in small VLDL (mmol/l)	2440	0.03	-0.02	0.08	0.201	2440	-0.01	-0.06	0.05	0.801	2058	0.10	0.03	0.18	0.007	2058	0.03	-0.04	0.10	0.405
Free cholesterol in small VLDL (mmol/l)	2440	0.04	-0.01	0.09	0.080	2440	0.01	-0.04	0.06	0.743	2058	0.12	0.05	0.19	0.001	2058	0.05	-0.02	0.12	0.140
Triglycerides in small VLDL (mmol/l)	2440	0.06	0.01	0.11	0.015	2440	0.02	-0.03	0.07	0.363	2058	0.14	0.07	0.21	0.0001	2058	0.07	0.00	0.14	0.036
Concentration of very small VLDL particles (mol/l)	2440	0.01	-0.03	0.06	0.570	2440	-0.01	-0.06	0.04	0.763	2058	0.05	-0.02	0.12	0.172	2058	0.01	-0.06	0.08	0.880
Total lipids in very small VLDL (mmol/l)	2440	0.01	-0.04	0.06	0.619	2440	-0.02	-0.07	0.04	0.563	2058	0.06	-0.02	0.13	0.124	2058	0.01	-0.07	0.08	0.869
Phospholipids in very small VLDL (mmol/l)	2440	0.00	-0.04	0.05	0.872	2440	-0.01	-0.06	0.04	0.568	2058	0.02	-0.05	0.09	0.604	2058	-0.02	-0.09	0.05	0.586
Total cholesterol in very small VLDL (mmol/l)	2440	0.01	-0.04	0.05	0.834	2440	-0.02	-0.08	0.03	0.362	2058	0.06	-0.01	0.14	0.115	2058	0.01	-0.06	0.09	0.772
Cholesterol esters in very small VLDL (mmol/l)	2440	0.01	-0.04	0.06	0.629	2440	-0.02	-0.07	0.03	0.423	2058	0.07	0.00	0.15	0.064	2058	0.02	-0.06	0.09	0.680
Free cholesterol in very small VLDL (mmol/l)	2440	-0.01	-0.06	0.04	0.613	2440	-0.03	-0.08	0.02	0.259	2058	0.03	-0.04	0.10	0.445	2058	0.00	-0.07	0.07	0.979
Triglycerides in very small VLDL (mmol/l)	2440	0.03	-0.01	0.08	0.162	2440	0.01	-0.04	0.06	0.629	2058	0.07	0.00	0.14	0.043	2058	0.03	-0.04	0.09	0.451
Concentration of IDL particles (mol/l)	2440	0.00	-0.05	0.05	0.984	2440	-0.01	-0.06	0.04	0.615	2058	0.00	-0.07	0.07	0.905	2058	-0.03	-0.10	0.04	0.355
Total lipids in IDL (mmol/l)	2440	0.00	-0.05	0.05	0.951	2440	-0.02	-0.07	0.03	0.495	2058	0.00	-0.07	0.07	0.992	2058	-0.03	-0.10	0.04	0.368
Phospholipids in IDL (mmol/l)	2440	0.00	-0.05	0.04	0.835	2440	-0.02	-0.07	0.03	0.505	2058	-0.02	-0.09	0.05	0.621	2058	-0.04	-0.11	0.03	0.221
Total cholesterol in IDL (mmol/l)	2440	0.00	-0.05	0.05	0.965	2440	-0.02	-0.07	0.03	0.426	2058	0.01	-0.06	0.08	0.787	2058	-0.03	-0.10	0.05	0.453
Cholesterol esters in IDL (mmol/l)	2440	0.00	-0.04	0.05	0.870	2440	-0.02	-0.07	0.03	0.453	2058	0.03	-0.05	0.10	0.494	2058	-0.02	-0.09	0.06	0.613
Free cholesterol in IDL (mmol/l)	2440	-0.01	-0.06	0.03	0.575	2440	-0.02	-0.07	0.03	0.377	2058	-0.03	-0.10	0.04	0.433	2058	-0.05	-0.12	0.02	0.181
Triglycerides in IDL (mmol/l)	2440	0.00	-0.04	0.05	0.885	2440	0.00	-0.04	0.05	0.872	2058	-0.02	-0.08	0.05	0.614	2058	-0.02	-0.09	0.04	0.466
Concentration of large LDL particles (mol/l)	2440	0.00	-0.05	0.04	0.932	2440	-0.02	-0.07	0.03	0.464	2058	-0.01	-0.08	0.05	0.686	2058	-0.05	-0.11	0.02	0.189
Total lipids in large LDL (mmol/l)	2440	0.00	-0.05	0.04	0.915	2440	-0.02	-0.07	0.03	0.427	2058	-0.01	-0.08	0.06	0.760	2058	-0.04	-0.11	0.03	0.212
Phospholipids in large LDL (mmol/l)	2440	0.00	-0.05	0.05	0.969	2440	-0.02	-0.07	0.03	0.430	2058	0.00	-0.07	0.07	0.954	2058	-0.04	-0.11	0.03	0.253
Total cholesterol in large LDL (mmol/l)	2440	0.00	-0.05	0.04	0.879	2440	-0.02	-0.07	0.03	0.376	2058	-0.01	-0.08	0.06	0.793	2058	-0.04	-0.11	0.03	0.213
Cholesterol esters in large LDL (mmol/l)	2440	0.00	-0.05	0.05	0.986	2440	-0.02	-0.07	0.03	0.406	2058	0.00	-0.07	0.07	0.937	2058	-0.04	-0.11	0.03	0.250
Free cholesterol in large LDL (mmol/l)	2440	-0.01	-0.06	0.03	0.570	2440	-0.03	-0.07	0.02	0.298	2058	-0.03	-0.10	0.04	0.401	2058	-0.05	-0.13	0.02	0.125
Triglycerides in large LDL (mmol/l)	2440	0.00	-0.04	0.04	0.971	2440	0.00	-0.04	0.05	0.917	2058	-0.03	-0.10	0.03	0.274	2058	-0.03	-0.10	0.03	0.277

Online Table 5 Associations of lean mass index at age 10y and 18y with cardiometabolic traits at age 18y in ALSPAC

	At age 10y										At age 18y									
	**Lean mass index (per 1.0 kg/m^2 higher)**										**Lean mass index (per 2.2 kg/m^2 higher)**									
	Adj. for age, sex, ethnicity, maternal education					*Additionally adj. for fat mass index at 10y*					*Adj. for age, sex, ethnicity, maternal education, smoking, alcohol, puberty timing*					*Additionally adj. for fat mass index at 18y*				
**Standardized outcome at age 18y**	**N**	**Beta**	**LCL**	**UCL**	**P-value**	**N**	**Beta**	**LCL**	**UCL**	**P-value**	**N**	**Beta**	**LCL**	**UCL**	**P-value**	**N**	**Beta**	**LCL**	**UCL**	**P-value**
Concentration of medium LDL particles (mol/l)	2440	0.00	-0.04	0.05	0.895	2440	-0.02	-0.06	0.03	0.509	2058	-0.01	-0.07	0.06	0.832	2058	-0.04	-0.11	0.02	0.199
Total lipids in medium LDL (mmol/l)	2440	0.00	-0.05	0.04	0.959	2440	-0.02	-0.07	0.03	0.396	2058	-0.01	-0.08	0.06	0.804	2058	-0.05	-0.11	0.02	0.194
Phospholipids in medium LDL (mmol/l)	2440	0.00	-0.05	0.05	0.952	2440	-0.03	-0.08	0.02	0.288	2058	0.00	-0.06	0.07	0.917	2058	-0.04	-0.11	0.02	0.210
Total cholesterol in medium LDL (mmol/l)	2440	0.00	-0.05	0.05	0.963	2440	-0.02	-0.07	0.03	0.402	2058	-0.01	-0.07	0.06	0.868	2058	-0.04	-0.11	0.03	0.227
Cholesterol esters in medium LDL (mmol/l)	2440	0.00	-0.04	0.05	0.942	2440	-0.02	-0.07	0.03	0.476	2058	0.00	-0.07	0.07	0.956	2058	-0.04	-0.11	0.03	0.268
Free cholesterol in medium LDL (mmol/l)	2440	-0.01	-0.06	0.03	0.554	2440	-0.03	-0.08	0.01	0.166	2058	-0.02	-0.09	0.05	0.527	2058	-0.06	-0.13	0.01	0.106
Triglycerides in medium LDL (mmol/l)	2440	0.00	-0.05	0.04	0.837	2440	0.00	-0.05	0.04	0.944	2058	-0.05	-0.11	0.01	0.103	2058	-0.05	-0.11	0.01	0.104
Concentration of small LDL particles (mol/l)	2440	0.00	-0.05	0.04	0.900	2440	-0.02	-0.07	0.02	0.321	2058	-0.02	-0.08	0.05	0.648	2058	-0.05	-0.12	0.01	0.127
Total lipids in small LDL (mmol/l)	2440	0.00	-0.05	0.04	0.888	2440	-0.03	-0.07	0.02	0.310	2058	-0.01	-0.08	0.06	0.755	2058	-0.05	-0.12	0.02	0.159
Phospholipids in small LDL (mmol/l)	2440	0.00	-0.05	0.04	0.910	2440	-0.03	-0.08	0.02	0.233	2058	-0.01	-0.07	0.06	0.881	2058	-0.05	-0.12	0.02	0.166
Total cholesterol in small LDL (mmol/l)	2440	-0.01	-0.05	0.04	0.797	2440	-0.03	-0.08	0.02	0.285	2058	-0.01	-0.08	0.05	0.691	2058	-0.05	-0.12	0.02	0.153
Cholesterol esters in small LDL (mmol/l)	2440	0.00	-0.05	0.04	0.930	2440	-0.02	-0.07	0.03	0.381	2058	-0.01	-0.08	0.06	0.741	2058	-0.05	-0.12	0.02	0.172
Free cholesterol in small LDL (mmol/l)	2440	-0.02	-0.07	0.02	0.317	2440	-0.05	-0.10	0.00	0.059	2058	-0.02	-0.09	0.05	0.511	2058	-0.06	-0.13	0.01	0.101
Triglycerides in small LDL (mmol/l)	2440	0.02	-0.03	0.06	0.441	2440	0.01	-0.04	0.05	0.835	2058	0.00	-0.06	0.07	0.922	2058	-0.02	-0.09	0.04	0.478
Concentration of very large HDL particles (mol/l)	2440	-0.05	-0.09	-0.01	0.029	2440	-0.02	-0.07	0.02	0.331	2058	-0.15	-0.22	-0.08	<0.0001	2058	-0.10	-0.17	-0.03	0.004
Total lipids in very large HDL (mmol/l)	2440	-0.05	-0.10	-0.01	0.018	2440	-0.03	-0.08	0.02	0.209	2058	-0.15	-0.22	-0.08	<0.0001	2058	-0.10	-0.17	-0.03	0.004
Phospholipids in very large HDL (mmol/l)	2440	-0.05	-0.09	0.00	0.031	2440	-0.02	-0.06	0.03	0.449	2058	-0.15	-0.22	-0.09	<0.0001	2058	-0.10	-0.16	-0.03	0.003
Total cholesterol in very large HDL (mmol/l)	2440	-0.06	-0.11	-0.01	0.012	2440	-0.04	-0.09	0.00	0.073	2058	-0.14	-0.21	-0.07	0.0002	2058	-0.10	-0.18	-0.03	0.006
Cholesterol esters in very large HDL (mmol/l)	2440	-0.06	-0.11	-0.01	0.011	2440	-0.05	-0.10	0.00	0.052	2058	-0.13	-0.21	-0.06	0.001	2058	-0.10	-0.18	-0.03	0.008
Free cholesterol in very large HDL (mmol/l)	2440	-0.05	-0.10	-0.01	0.021	2440	-0.03	-0.08	0.02	0.215	2058	-0.15	-0.22	-0.08	<0.0001	2058	-0.10	-0.17	-0.04	0.003
Triglycerides in very large HDL (mmol/l)	2440	0.02	-0.03	0.07	0.399	2440	0.03	-0.02	0.08	0.289	2058	0.02	-0.05	0.10	0.545	2058	0.02	-0.05	0.10	0.550
Concentration of large HDL particles (mol/l)	2440	-0.04	-0.09	0.00	0.056	2440	-0.01	-0.06	0.03	0.646	2058	-0.14	-0.20	-0.07	<0.0001	2058	-0.08	-0.14	-0.02	0.013
Total lipids in large HDL (mmol/l)	2440	-0.05	-0.09	0.00	0.047	2440	-0.01	-0.06	0.03	0.587	2058	-0.14	-0.21	-0.08	<0.0001	2058	-0.08	-0.14	-0.02	0.009
Phospholipids in large HDL (mmol/l)	2440	-0.04	-0.08	0.00	0.075	2440	-0.01	-0.06	0.03	0.613	2058	-0.13	-0.20	-0.07	<0.0001	2058	-0.08	-0.14	-0.02	0.010
Total cholesterol in large HDL (mmol/l)	2440	-0.05	-0.10	-0.01	0.025	2440	-0.02	-0.06	0.03	0.510	2058	-0.15	-0.22	-0.09	<0.0001	2058	-0.09	-0.15	-0.03	0.006
Cholesterol esters in large HDL (mmol/l)	2440	-0.05	-0.10	-0.01	0.026	2440	-0.01	-0.06	0.03	0.529	2058	-0.15	-0.22	-0.09	<0.0001	2058	-0.09	-0.15	-0.02	0.007
Free cholesterol in large HDL (mmol/l)	2440	-0.05	-0.10	-0.01	0.021	2440	-0.02	-0.07	0.03	0.412	2058	-0.15	-0.22	-0.09	<0.0001	2058	-0.09	-0.15	-0.03	0.004
Triglycerides in large HDL (mmol/l)	2440	0.04	0.00	0.09	0.053	2440	0.05	0.01	0.10	0.029	2058	0.05	-0.02	0.11	0.137	2058	0.06	-0.01	0.12	0.089
Concentration of medium HDL particles (mol/l)	2440	-0.01	-0.05	0.04	0.789	2440	-0.01	-0.05	0.04	0.719	2058	-0.04	-0.10	0.02	0.192	2058	-0.03	-0.09	0.03	0.365
Total lipids in medium HDL (mmol/l)	2440	-0.01	-0.05	0.03	0.621	2440	-0.01	-0.06	0.03	0.630	2058	-0.06	-0.12	0.01	0.083	2058	-0.04	-0.10	0.02	0.215
Phospholipids in medium HDL (mmol/l)	2440	-0.02	-0.06	0.03	0.480	2440	-0.01	-0.06	0.03	0.566	2058	-0.06	-0.12	0.00	0.052	2058	-0.04	-0.10	0.02	0.179
Total cholesterol in medium HDL (mmol/l)	2440	-0.02	-0.06	0.03	0.426	2440	-0.02	-0.06	0.03	0.494	2058	-0.07	-0.14	-0.01	0.033	2058	-0.05	-0.12	0.02	0.126
Cholesterol esters in medium HDL (mmol/l)	2440	-0.02	-0.06	0.03	0.391	2440	-0.02	-0.06	0.03	0.476	2058	-0.08	-0.15	-0.01	0.025	2058	-0.06	-0.13	0.01	0.110
Free cholesterol in medium HDL (mmol/l)	2440	-0.01	-0.05	0.03	0.617	2440	-0.01	-0.06	0.03	0.547	2058	-0.05	-0.12	0.01	0.121	2058	-0.04	-0.11	0.03	0.227
Triglycerides in medium HDL (mmol/l)	2440	0.08	0.03	0.13	0.002	2440	0.05	0.00	0.10	0.059	2058	0.15	0.08	0.22	<0.0001	2058	0.10	0.03	0.17	0.004
Concentration of small HDL particles (mol/l)	2440	0.02	-0.03	0.06	0.498	2440	0.00	-0.05	0.04	0.842	2058	0.01	-0.06	0.08	0.751	2058	-0.01	-0.07	0.06	0.879
Total lipids in small HDL (mmol/l)	2440	0.00	-0.05	0.04	0.876	2440	-0.01	-0.06	0.03	0.544	2058	-0.03	-0.09	0.04	0.410	2058	-0.03	-0.10	0.03	0.358
Phospholipids in small HDL (mmol/l)	2440	0.02	-0.02	0.07	0.360	2440	0.01	-0.04	0.05	0.806	2058	0.02	-0.05	0.09	0.620	2058	0.01	-0.06	0.08	0.791
Total cholesterol in small HDL (mmol/l)	2440	-0.04	-0.08	0.00	0.060	2440	-0.04	-0.08	0.00	0.068	2058	-0.09	-0.15	-0.03	0.003	2058	-0.08	-0.14	-0.02	0.007
Cholesterol esters in small HDL (mmol/l)	2440	-0.04	-0.08	0.00	0.030	2440	-0.05	-0.09	0.00	0.033	2058	-0.09	-0.15	-0.04	0.001	2058	-0.09	-0.15	-0.03	0.003
Free cholesterol in small HDL (mmol/l)	2440	-0.01	-0.05	0.04	0.698	2440	-0.01	-0.05	0.04	0.805	2058	-0.04	-0.11	0.03	0.225	2058	-0.02	-0.09	0.05	0.499
Triglycerides in small HDL (mmol/l)	2440	0.06	0.01	0.11	0.018	2440	0.04	-0.01	0.09	0.138	2058	0.11	0.04	0.18	0.002	2058	0.07	0.00	0.14	0.044
Phospholipids to total lipds ratio in chylomicrons and extremely large VLDL (%)	2440	0.02	-0.01	0.06	0.203	2440	0.01	-0.03	0.05	0.629	2058	-0.02	-0.05	0.02	0.393	2058	-0.02	-0.06	0.01	0.238
Total cholesterol to total lipids ratio in chylomicrons and extremely large VLDL (%)	2440	0.04	-0.02	0.09	0.215	2440	0.03	-0.03	0.09	0.338	2058	0.09	0.03	0.16	0.006	2058	0.07	0.00	0.13	0.058
Cholesterol esters to total lipids ratio in chylomicrons and extremely large VLDL (%)	2440	0.02	-0.04	0.08	0.452	2440	0.03	-0.04	0.09	0.394	2058	0.08	0.01	0.15	0.019	2058	0.06	-0.01	0.13	0.072
Free cholesterol to total lipids ratio in chylomicrons and extremely large VLDL (%)	2440	0.06	0.01	0.11	0.027	2440	0.02	-0.03	0.08	0.413	2058	0.05	-0.02	0.13	0.140	2058	0.02	-0.05	0.09	0.633
Triglycerides to total lipids ratio in chylomicrons and extremely large VLDL (%)	2440	-0.06	-0.11	0.00	0.036	2440	-0.05	-0.11	0.01	0.105	2058	-0.09	-0.15	-0.02	0.006	2058	-0.06	-0.13	0.00	0.056
Phospholipids to total lipds ratio in very large VLDL (%)	2440	0.04	0.00	0.09	0.078	2440	0.00	-0.05	0.06	0.851	2058	0.06	-0.01	0.12	0.107	2058	0.00	-0.06	0.07	0.913
Total cholesterol to total lipids ratio in very large VLDL (%)	2440	0.01	-0.04	0.06	0.780	2440	0.02	-0.03	0.08	0.421	2058	0.03	-0.04	0.09	0.377	2058	0.04	-0.03	0.11	0.222
Cholesterol esters to total lipids ratio in very large VLDL (%)	2440	0.01	-0.05	0.08	0.640	2440	0.03	-0.03	0.08	0.354	2058	0.02	-0.03	0.08	0.350	2058	0.04	-0.02	0.09	0.189
Free cholesterol to total lipids ratio in very large VLDL (%)	2440	0.02	-0.04	0.07	0.523	2440	0.01	-0.04	0.06	0.670	2058	0.02	-0.03	0.07	0.533	2058	0.02	-0.04	0.07	0.575
Triglycerides to total lipids ratio in very large VLDL (%)	2440	0.01	-0.04	0.06	0.668	2440	0.01	-0.05	0.06	0.853	2058	-0.05	-0.12	0.02	0.141	2058	-0.05	-0.12	0.02	0.193
Phospholipids to total lipds ratio in large VLDL (%)	2440	0.04	0.00	0.09	0.069	2440	0.02	-0.03	0.07	0.523	2058	0.04	-0.03	0.10	0.274	2058	0.00	-0.07	0.06	0.944
Total cholesterol to total lipids ratio in large VLDL (%)	2440	0.06	0.01	0.11	0.013	2440	0.05	0.00	0.10	0.070	2058	0.13	0.05	0.20	0.001	2058	0.09	0.01	0.17	0.028
Cholesterol esters to total lipids ratio in large VLDL (%)	2440	0.05	0.01	0.10	0.026	2440	0.06	0.00	0.11	0.033	2058	0.12	0.03	0.20	0.005	2058	0.10	0.02	0.19	0.017
Free cholesterol to total lipids ratio in large VLDL (%)	2440	0.00	-0.04	0.04	0.998	2440	-0.01	-0.06	0.03	0.537	2058	0.03	0.00	0.06	0.054	2058	0.01	-0.02	0.04	0.647
Triglycerides to total lipids ratio in large VLDL (%)	2440	-0.06	-0.11	-0.01	0.012	2440	-0.04	-0.09	0.01	0.096	2058	-0.10	-0.17	-0.03	0.003	2058	-0.06	-0.13	0.01	0.080
Phospholipids to total lipds ratio in medium VLDL (%)	2440	-0.03	-0.07	0.02	0.206	2440	-0.01	-0.05	0.04	0.758	2058	-0.10	-0.17	-0.03	0.003	2058	-0.06	-0.13	0.01	0.084

Online Table 5 Associations of lean mass index at age 10y and 18y with cardiometabolic traits at age 18y in ALSPAC

	At age 10y										At age 18y									
	Lean mass index (per 1.0 kg/m^2 higher)										Lean mass index (per 2.2 kg/m^2 higher)									
	Adj. for age, sex, ethnicity, maternal education					*Additionally adj. for fat mass index at 10y*					*Adj. for age, sex, ethnicity, maternal education, smoking, alcohol, puberty timing*					*Additionally adj. for fat mass index at 18y*				
**Standardized outcome at age 18y**	**N**	**Beta**	**LCL**	**UCL**	**P-value**	**N**	**Beta**	**LCL**	**UCL**	**P-value**	**N**	**Beta**	**LCL**	**UCL**	**P-value**	**N**	**Beta**	**LCL**	**UCL**	**P-value**
Total cholesterol to total lipids ratio in medium VLDL (%)	2440	0.03	-0.02	0.07	0.219	2440	0.02	-0.02	0.07	0.337	2058	0.07	0.00	0.14	0.035	2058	0.05	-0.02	0.12	0.163
Cholesterol esters to total lipids ratio in medium VLDL (%)	2440	0.02	-0.02	0.07	0.324	2440	0.02	-0.02	0.07	0.357	2058	0.08	0.01	0.15	0.027	2058	0.06	-0.01	0.13	0.102
Free cholesterol to total lipids ratio in medium VLDL (%)	2440	0.03	-0.02	0.08	0.291	2440	0.01	-0.04	0.06	0.729	2058	0.02	-0.05	0.08	0.589	2058	-0.01	-0.07	0.06	0.821
Triglycerides to total lipids ratio in medium VLDL (%)	2440	-0.02	-0.07	0.02	0.322	2440	-0.02	-0.07	0.03	0.364	2058	-0.05	-0.12	0.02	0.168	2058	-0.03	-0.10	0.04	0.333
Phospholipids to total lipds ratio in small VLDL (%)	2440	-0.04	-0.09	0.01	0.098	2440	-0.01	-0.06	0.04	0.754	2058	-0.11	-0.17	-0.04	0.002	2058	-0.04	-0.11	0.02	0.214
Total cholesterol to total lipids ratio in small VLDL (%)	2440	-0.03	-0.07	0.02	0.271	2440	-0.03	-0.07	0.02	0.291	2058	-0.02	-0.10	0.05	0.570	2058	-0.03	-0.10	0.05	0.461
Cholesterol esters to total lipids ratio in small VLDL (%)	2440	-0.02	-0.07	0.03	0.408	2440	-0.02	-0.07	0.02	0.323	2058	-0.01	-0.08	0.07	0.852	2058	-0.02	-0.10	0.05	0.569
Free cholesterol to total lipids ratio in small VLDL (%)	2440	-0.04	-0.08	0.00	0.068	2440	-0.01	-0.05	0.03	0.645	2058	-0.10	-0.16	-0.04	0.002	2058	-0.05	-0.11	0.02	0.140
Triglycerides to total lipids ratio in small VLDL (%)	2440	0.04	-0.01	0.08	0.085	2440	0.03	-0.02	0.08	0.244	2058	0.06	-0.01	0.13	0.119	2058	0.04	-0.03	0.11	0.258
Phospholipids to total lipds ratio in very small VLDL (%)	2440	-0.01	-0.05	0.04	0.761	2440	0.00	-0.05	0.04	0.949	2058	-0.05	-0.12	0.01	0.084	2058	-0.05	-0.12	0.01	0.095
Total cholesterol to total lipids ratio in very small VLDL (%)	2440	-0.02	-0.06	0.02	0.362	2440	-0.03	-0.07	0.02	0.280	2058	0.01	-0.05	0.08	0.682	2058	0.02	-0.05	0.08	0.628
Cholesterol esters to total lipids ratio in very small VLDL (%)	2440	0.00	-0.05	0.04	0.847	2440	-0.02	-0.06	0.03	0.447	2058	0.03	-0.03	0.10	0.271	2058	0.02	-0.04	0.08	0.516
Free cholesterol to total lipids ratio in very small VLDL (%)	2440	-0.06	-0.11	-0.02	0.006	2440	-0.04	-0.08	0.01	0.121	2058	-0.07	-0.13	0.00	0.049	2058	-0.01	-0.07	0.05	0.726
Triglycerides to total lipids ratio in very small VLDL (%)	2440	0.03	-0.02	0.08	0.188	2440	0.03	-0.02	0.08	0.178	2058	0.02	-0.05	0.09	0.551	2058	0.02	-0.05	0.09	0.623
Phospholipids to total lipds ratio in IDL (%)	2440	-0.03	-0.07	0.02	0.261	2440	0.01	-0.04	0.05	0.779	2058	-0.13	-0.20	-0.06	0.0003	2058	-0.07	-0.14	0.00	0.037
Total cholesterol to total lipids ratio in IDL (%)	2440	0.01	-0.04	0.05	0.712	2440	-0.02	-0.07	0.03	0.394	2058	0.08	0.01	0.15	0.030	2058	0.04	-0.03	0.11	0.324
Cholesterol esters to total lipids ratio in IDL (%)	2440	0.03	-0.01	0.07	0.174	2440	-0.01	-0.05	0.04	0.709	2058	0.13	0.07	0.20	<0.0001	2058	0.07	0.01	0.14	0.030
Free cholesterol to total lipids ratio in IDL (%)	2440	-0.05	-0.10	0.00	0.035	2440	-0.03	-0.08	0.02	0.249	2058	-0.12	-0.19	-0.05	0.0004	2058	-0.08	-0.15	-0.02	0.016
Triglycerides to total lipids ratio in IDL (%)	2440	0.00	-0.04	0.05	0.983	2440	0.02	-0.03	0.07	0.389	2058	-0.04	-0.11	0.03	0.286	2058	-0.01	-0.08	0.06	0.743
Phospholipids to total lipds ratio in large LDL (%)	2440	0.00	-0.04	0.05	0.889	2440	0.01	-0.03	0.06	0.599	2058	0.02	-0.05	0.08	0.659	2058	0.03	-0.03	0.10	0.332
Total cholesterol to total lipids ratio in large LDL (%)	2440	0.00	-0.04	0.05	0.919	2440	-0.02	-0.07	0.03	0.395	2058	0.02	-0.05	0.10	0.509	2058	-0.01	-0.09	0.06	0.749
Cholesterol esters to total lipids ratio in large LDL (%)	2440	0.02	-0.03	0.06	0.448	2440	-0.01	-0.06	0.03	0.593	2058	0.05	-0.03	0.12	0.200	2058	0.00	-0.07	0.07	0.943
Free cholesterol to total lipids ratio in large LDL (%)	2440	-0.05	-0.09	0.00	0.059	2440	-0.01	-0.06	0.04	0.622	2058	-0.08	-0.15	-0.01	0.020	2058	-0.02	-0.09	0.04	0.478
Triglycerides to total lipids ratio in large LDL (%)	2440	-0.01	-0.05	0.04	0.758	2440	0.02	-0.03	0.07	0.419	2058	-0.06	-0.13	0.01	0.114	2058	-0.02	-0.09	0.05	0.598
Phospholipids to total lipds ratio in medium LDL (%)	2440	-0.01	-0.05	0.04	0.780	2440	0.00	-0.05	0.05	0.966	2058	0.00	-0.07	0.07	0.933	2058	0.02	-0.05	0.09	0.581
Total cholesterol to total lipids ratio in medium LDL (%)	2440	0.01	-0.03	0.06	0.548	2440	0.00	-0.05	0.04	0.901	2058	0.03	-0.04	0.11	0.353	2058	0.01	-0.07	0.08	0.882
Cholesterol esters to total lipids ratio in medium LDL (%)	2440	0.02	-0.03	0.06	0.403	2440	0.00	-0.05	0.05	0.999	2058	0.04	-0.03	0.11	0.311	2058	0.00	-0.07	0.07	0.943
Free cholesterol to total lipids ratio in medium LDL (%)	2440	-0.03	-0.07	0.02	0.247	2440	-0.01	-0.05	0.04	0.789	2058	-0.03	-0.10	0.03	0.304	2058	0.00	-0.06	0.07	0.967
Triglycerides to total lipids ratio in medium LDL (%)	2440	-0.01	-0.05	0.03	0.578	2440	0.01	-0.03	0.05	0.615	2058	-0.08	-0.14	-0.02	0.013	2058	-0.05	-0.11	0.01	0.124
Phospholipids to total lipds ratio in small LDL (%)	2440	-0.01	-0.05	0.04	0.773	2440	0.01	-0.04	0.05	0.730	2058	-0.01	-0.07	0.06	0.857	2058	0.02	-0.05	0.09	0.546
Total cholesterol to total lipids ratio in small LDL (%)	2440	0.00	-0.05	0.04	0.914	2440	-0.02	-0.06	0.03	0.499	2058	0.00	-0.07	0.07	0.925	2058	-0.02	-0.09	0.05	0.582
Cholesterol esters to total lipids ratio in small LDL (%)	2440	0.01	-0.04	0.05	0.673	2440	-0.01	-0.06	0.04	0.748	2058	0.01	-0.06	0.08	0.714	2058	-0.02	-0.09	0.05	0.624
Free cholesterol to total lipids ratio in small LDL (%)	2440	-0.03	-0.07	0.01	0.174	2440	-0.01	-0.06	0.03	0.642	2058	-0.03	-0.09	0.04	0.410	2058	0.01	-0.06	0.07	0.786
Triglycerides to total lipids ratio in small LDL (%)	2440	0.02	-0.02	0.07	0.294	2440	0.03	-0.02	0.07	0.288	2058	0.00	-0.07	0.07	0.908	2058	0.00	-0.07	0.07	0.976
Phospholipids to total lipids ratio in very large HDL (%)	2440	-0.05	-0.09	0.00	0.036	2440	-0.01	-0.05	0.04	0.815	2058	-0.15	-0.21	-0.08	<0.0001	2058	-0.09	-0.15	-0.03	0.004
Total cholesterol to total lipids ratio in very large HDL (%)	2440	0.03	-0.01	0.07	0.141	2440	-0.01	-0.05	0.04	0.729	2058	0.12	0.06	0.18	0.0002	2058	0.07	0.01	0.13	0.030
Cholesterol esters to total lipids ratio in very large HDL (%)	2440	0.03	-0.01	0.07	0.130	2440	-0.01	-0.05	0.04	0.817	2058	0.12	0.06	0.19	0.0001	2058	0.07	0.01	0.13	0.019
Free cholesterol to total lipids ratio in very large HDL (%)	2440	-0.03	-0.07	0.02	0.244	2440	-0.03	-0.08	0.02	0.290	2058	-0.09	-0.16	-0.01	0.021	2058	-0.08	-0.15	-0.01	0.033
Triglycerides to total lipids ratio in very large HDL (%)	2440	0.09	0.03	0.14	0.001	2440	0.06	0.01	0.11	0.013	2058	0.17	0.09	0.26	<0.0001	2058	0.12	0.05	0.20	0.002
Phospholipids to total lipds ratio in large HDL (%)	2440	0.08	0.03	0.13	0.002	2440	0.02	-0.03	0.07	0.347	2058	0.14	0.07	0.21	0.0002	2058	0.06	-0.01	0.12	0.083
Total cholesterol to total lipids ratio in large HDL (%)	2440	-0.10	-0.15	-0.04	0.0003	2440	-0.04	-0.09	0.01	0.091	2058	-0.18	-0.26	-0.10	<0.0001	2058	-0.10	-0.16	-0.03	0.005
Cholesterol esters to total lipids ratio in large HDL (%)	2440	-0.09	-0.15	-0.04	0.0004	2440	-0.04	-0.09	0.01	0.124	2058	-0.17	-0.25	-0.10	<0.0001	2058	-0.09	-0.16	-0.02	0.014
Free cholesterol to total lipids ratio in large HDL (%)	2440	-0.09	-0.14	-0.04	0.0004	2440	-0.05	-0.10	0.00	0.032	2058	-0.17	-0.24	-0.10	<0.0001	2058	-0.11	-0.18	-0.04	0.001
Triglycerides to total lipids ratio in large HDL (%)	2440	0.11	0.06	0.16	<0.0001	2440	0.07	0.02	0.12	0.004	2058	0.21	0.14	0.29	<0.0001	2058	0.14	0.07	0.21	<0.0001
Phospholipids to total lipids ratio in medium HDL (%)	2440	-0.04	-0.08	0.01	0.109	2440	-0.03	-0.07	0.02	0.267	2058	-0.06	-0.13	0.02	0.134	2058	-0.04	-0.12	0.04	0.310
Total cholesterol to total lipids ratio in medium HDL (%)	2440	-0.02	-0.07	0.03	0.405	2440	-0.01	-0.06	0.04	0.696	2058	-0.05	-0.13	0.03	0.202	2058	-0.03	-0.11	0.05	0.429
Cholesterol esters to total lipids ratio in medium HDL (%)	2440	-0.02	-0.07	0.02	0.342	2440	-0.01	-0.06	0.04	0.665	2058	-0.06	-0.13	0.02	0.122	2058	-0.04	-0.11	0.04	0.341
Free cholesterol to total lipids ratio in medium HDL (%)	2440	0.00	-0.04	0.04	0.894	2440	-0.01	-0.05	0.04	0.793	2058	0.02	-0.06	0.10	0.632	2058	0.01	-0.08	0.09	0.891
Triglycerides to total lipids ratio in medium HDL (%)	2440	0.10	0.05	0.15	0.0001	2440	0.07	0.01	0.12	0.012	2058	0.20	0.12	0.28	<0.0001	2058	0.13	0.06	0.20	0.0004
Phospholipids to total lipds ratio in small HDL (%)	2440	0.05	0.01	0.10	0.008	2440	0.05	0.01	0.09	0.026	2058	0.10	0.04	0.16	0.002	2058	0.09	0.03	0.16	0.005
Total cholesterol to total lipids ratio in small HDL (%)	2440	-0.08	-0.12	-0.03	0.0003	2440	-0.06	-0.11	-0.02	0.003	2058	-0.14	-0.20	-0.07	<0.0001	2058	-0.12	-0.18	-0.05	0.0003
Cholesterol esters to total lipids ratio in small HDL (%)	2440	-0.07	-0.11	-0.03	0.001	2440	-0.06	-0.10	-0.02	0.005	2058	-0.12	-0.18	-0.05	0.0003	2058	-0.11	-0.17	-0.05	0.001
Free cholesterol to total lipids ratio in small HDL (%)	2440	-0.03	-0.07	0.02	0.301	2440	0.01	-0.04	0.06	0.627	2058	-0.06	-0.13	0.02	0.133	2058	0.01	-0.06	0.08	0.828
Triglycerides to total lipids ratio in small HDL (%)	2440	0.08	0.02	0.13	0.004	2440	0.06	0.00	0.11	0.033	2058	0.14	0.06	0.22	0.0005	2058	0.10	0.02	0.17	0.012
Mean diameter for VLDL particles (nm)	2440	0.07	0.02	0.12	0.004	2440	0.03	-0.02	0.08	0.261	2058	0.15	0.08	0.22	<0.0001	2058	0.08	0.01	0.15	0.018
Mean diameter for LDL particles (nm)	2440	0.01	-0.03	0.06	0.633	2440	0.05	0.00	0.09	0.052	2058	0.02	-0.05	0.09	0.589	2058	0.06	-0.01	0.13	0.118
Mean diameter for HDL particles (nm)	2440	-0.06	-0.10	-0.01	0.010	2440	-0.02	-0.07	0.02	0.327	2058	-0.15	-0.22	-0.08	<0.0001	2058	-0.09	-0.16	-0.03	0.006
Serum total cholesterol (mmol/l)	2440	-0.01	-0.05	0.04	0.744	2440	-0.03	-0.07	0.02	0.300	2058	-0.02	-0.09	0.05	0.593	2058	-0.05	-0.12	0.02	0.167

Online Table 5 Associations of lean mass index at age 10y and 18y with cardiometabolic traits at age 18y in ALSPAC

	At age 10y										At age 18y									
	**Lean mass index (per 1.0 kg/m^2 higher)**										**Lean mass index (per 2.2 kg/m^2 higher)**									
	Adj. for age, sex, ethnicity, maternal education					*Additionally adj. for fat mass index at 10y*					*Adj. for age, sex, ethnicity, maternal education, smoking, alcohol, puberty timing*					*Additionally adj. for fat mass index at 18y*				
**Standardized outcome at age 18y**	**N**	**Beta**	**LCL**	**UCL**	**P-value**	**N**	**Beta**	**LCL**	**UCL**	**P-value**	**N**	**Beta**	**LCL**	**UCL**	**P-value**	**N**	**Beta**	**LCL**	**UCL**	**P-value**
Total cholesterol in VLDL (mmol/l)	2440	0.05	0.00	0.11	0.039	2440	0.01	-0.04	0.06	0.726	2058	0.14	0.07	0.22	0.0002	2058	0.06	-0.01	0.14	0.074
Remnant cholesterol (non-HDL, non-LDL -cholesterol) (mmol/l)	2440	0.03	-0.02	0.08	0.219	2440	-0.01	-0.06	0.05	0.850	2058	0.09	0.02	0.17	0.017	2058	0.03	-0.05	0.10	0.498
Total cholesterol in LDL (mmol/l)	2440	0.00	-0.05	0.04	0.886	2440	-0.02	-0.07	0.03	0.362	2058	-0.01	-0.08	0.06	0.794	2058	-0.05	-0.12	0.02	0.203
Total cholesterol in HDL (mmol/l)	2440	-0.05	-0.10	-0.01	0.016	2440	-0.03	-0.08	0.01	0.145	2058	-0.15	-0.21	-0.08	<0.0001	2058	-0.10	-0.16	-0.04	0.002
Total cholesterol in HDL2 (mmol/l)	2440	-0.06	-0.11	-0.02	0.007	2440	-0.04	-0.08	0.01	0.110	2058	-0.16	-0.23	-0.10	<0.0001	2058	-0.11	-0.17	-0.05	0.001
Total cholesterol in HDL3 (mmol/l)	2440	-0.04	-0.08	0.01	0.086	2440	-0.03	-0.07	0.02	0.268	2058	-0.11	-0.17	-0.05	0.0005	2058	-0.08	-0.14	-0.02	0.011
Esterified cholesterol (mmol/l)	2430	-0.01	-0.05	0.04	0.734	2430	-0.03	-0.07	0.02	0.302	2051	-0.01	-0.08	0.05	0.699	2051	-0.04	-0.11	0.03	0.225
Free cholesterol (mmol/l)	2428	0.00	-0.05	0.04	0.883	2428	-0.02	-0.07	0.03	0.392	2049	-0.02	-0.09	0.04	0.496	2049	-0.05	-0.12	0.01	0.125
Serum total triglycerides (mmol/l)	2440	0.07	0.02	0.12	0.010	2440	0.03	-0.02	0.08	0.279	2058	0.14	0.06	0.21	0.0002	2058	0.07	0.00	0.13	0.050
Triglycerides in VLDL (mmol/l)	2440	0.07	0.02	0.12	0.005	2440	0.03	-0.02	0.08	0.266	2058	0.16	0.09	0.23	<0.0001	2058	0.08	0.02	0.15	0.016
Triglycerides in LDL (mmol/l)	2440	0.00	-0.04	0.05	0.949	2440	0.00	-0.04	0.05	0.942	2058	-0.03	-0.09	0.03	0.296	2058	-0.04	-0.10	0.02	0.234
Triglycerides in HDL (mmol/l)	2440	0.07	0.02	0.11	0.007	2440	0.05	0.00	0.10	0.053	2058	0.12	0.05	0.18	0.001	2058	0.08	0.01	0.15	0.017
Diacylglycerol (mmol/l)	2373	0.07	0.02	0.12	0.004	2373	0.04	-0.01	0.09	0.104	1997	0.14	0.06	0.21	0.0002	1997	0.09	0.01	0.16	0.019
Ratio of diacylglycerol to triglycerides	2374	0.04	-0.01	0.08	0.103	2374	0.01	-0.03	0.06	0.568	1998	0.06	-0.01	0.13	0.085	1998	0.04	-0.03	0.11	0.281
Total phosphoglycerides (mmol/l)	2428	0.01	-0.03	0.06	0.622	2428	0.01	-0.04	0.06	0.727	2049	-0.02	-0.08	0.04	0.565	2049	-0.02	-0.08	0.05	0.623
Ratio of triglycerides to phosphoglycerides	2428	0.09	0.03	0.14	0.001	2428	0.05	0.00	0.10	0.073	2049	0.18	0.10	0.25	<0.0001	2049	0.11	0.04	0.17	0.002
Phosphatidylcholine and other cholines (mmol/l)	2409	0.00	-0.04	0.05	0.880	2409	0.01	-0.04	0.06	0.710	2034	-0.01	-0.07	0.05	0.720	2034	0.00	-0.07	0.06	0.907
Total cholines (mmol/l)	2430	0.00	-0.04	0.04	0.982	2430	0.00	-0.05	0.05	0.970	2051	-0.01	-0.07	0.05	0.683	2051	-0.01	-0.07	0.05	0.746
Apolipoprotein A-I (g/l)	2440	-0.04	-0.08	0.01	0.101	2440	-0.03	-0.07	0.02	0.198	2058	-0.11	-0.17	-0.05	0.001	2058	-0.09	-0.15	-0.02	0.006
Apolipoprotein B (g/l)	2440	0.04	-0.01	0.09	0.123	2440	0.00	-0.05	0.05	0.913	2058	0.09	0.02	0.17	0.012	2058	0.03	-0.04	0.10	0.477
Ratio of apolipoprotein B to apolipoprotein A-I	2440	0.06	0.01	0.11	0.017	2440	0.02	-0.03	0.07	0.408	2058	0.15	0.07	0.23	0.0001	2058	0.07	0.00	0.14	0.057
Total fatty acids (mmol/l)	2430	0.03	-0.02	0.08	0.245	2430	0.00	-0.05	0.05	0.921	2051	0.05	-0.02	0.12	0.139	2051	0.01	-0.06	0.07	0.804
Estimated description of fatty acid chain length, not actual carbon number	2430	0.03	-0.02	0.07	0.206	2430	0.02	-0.03	0.07	0.378	2052	0.01	-0.06	0.08	0.771	2052	0.01	-0.06	0.08	0.736
Estimated degree of unsaturation	2430	0.00	-0.05	0.05	0.973	2430	0.00	-0.05	0.06	0.904	2051	-0.04	-0.11	0.04	0.372	2051	-0.02	-0.09	0.06	0.664
22:6, docosahexaenoic acid (mmol/l)	2430	0.02	-0.02	0.07	0.332	2430	0.02	-0.03	0.07	0.504	2051	0.02	-0.05	0.08	0.554	2051	0.01	-0.06	0.08	0.774
18:2, linoleic acid (mmol/l)	2429	0.00	-0.05	0.04	0.832	2429	-0.02	-0.06	0.03	0.506	2051	0.02	-0.04	0.09	0.541	2051	0.00	-0.06	0.07	0.944
Conjugated linoleic acid (mmol/l)	2429	0.05	0.00	0.09	0.047	2429	0.03	-0.02	0.08	0.207	2050	0.08	-0.01	0.18	0.093	2050	0.06	-0.04	0.16	0.245
Omega-3 fatty acids (mmol/l)	2430	0.04	-0.01	0.08	0.117	2430	0.02	-0.03	0.07	0.493	2051	0.05	-0.02	0.12	0.189	2051	0.02	-0.05	0.09	0.630
Omega-6 fatty acids (mmol/l)	2430	0.00	-0.04	0.05	0.903	2430	-0.01	-0.06	0.04	0.600	2051	0.02	-0.05	0.08	0.553	2051	-0.01	-0.07	0.06	0.868
Polyunsaturated fatty acids (mmol/l)	2429	0.01	-0.04	0.05	0.722	2429	-0.01	-0.06	0.04	0.730	2051	0.02	-0.04	0.09	0.470	2051	0.00	-0.07	0.06	0.937
Monounsaturated fatty acids; 16:1, 18:1 (mmol/l)	2430	0.04	-0.01	0.08	0.152	2430	0.00	-0.05	0.05	0.888	2051	0.03	-0.03	0.10	0.354	2051	-0.02	-0.09	0.04	0.512
Saturated fatty acids (mmol/l)	2429	0.03	-0.01	0.08	0.173	2429	0.01	-0.04	0.06	0.666	2050	0.08	0.01	0.15	0.027	2050	0.04	-0.03	0.11	0.224
Ratio of 22:6 docosahexaenoic acid to total fatty acids (%)	2431	0.02	-0.03	0.06	0.439	2431	0.02	-0.03	0.07	0.365	2052	0.00	-0.07	0.07	0.984	2052	0.01	-0.06	0.08	0.754
Ratio of 18:2 linoleic acid to total fatty acids (%)	2430	-0.06	-0.11	-0.01	0.020	2430	-0.03	-0.08	0.02	0.279	2052	-0.04	-0.11	0.03	0.220	2052	0.00	-0.07	0.07	0.976
Ratio of conjugated linoleic acid to total fatty acids (%)	2430	0.04	0.00	0.09	0.066	2430	0.04	-0.01	0.09	0.166	2051	0.08	-0.01	0.17	0.084	2051	0.06	-0.03	0.16	0.184
Ratio of omega-3 fatty acids to total fatty acids (%)	2431	0.03	-0.02	0.07	0.264	2431	0.02	-0.03	0.07	0.384	2052	0.02	-0.05	0.09	0.604	2052	0.02	-0.05	0.09	0.617
Ratio of omega-6 fatty acids to total fatty acids (%)	2431	-0.05	-0.10	0.00	0.036	2431	-0.02	-0.07	0.03	0.344	2052	-0.06	-0.13	0.02	0.129	2052	-0.02	-0.09	0.06	0.675
Ratio of polyunsaturated fatty acids to total fatty acids (%)	2430	-0.04	-0.09	0.01	0.085	2430	-0.02	-0.07	0.03	0.489	2052	-0.05	-0.12	0.02	0.185	2052	-0.01	-0.08	0.06	0.779
Ratio of monounsaturated fatty acids to total fatty acids (%)	2431	0.03	-0.02	0.07	0.277	2431	0.00	-0.05	0.04	0.875	2052	-0.03	-0.10	0.04	0.447	2052	-0.07	-0.14	-0.01	0.032
Ratio of saturated fatty acids to total fatty acids (%)	2430	0.02	-0.03	0.06	0.478	2430	0.03	-0.02	0.08	0.265	2051	0.10	0.03	0.17	0.007	2051	0.12	0.04	0.19	0.001
Insulin (mu/l)	2478	0.11	0.03	0.20	0.006	2478	0.06	0.00	0.12	0.056	2097	0.16	0.06	0.26	0.002	2097	0.09	0.01	0.16	0.022
Glucose (mmol/l)	2439	0.05	0.00	0.10	0.071	2439	0.04	-0.02	0.09	0.214	2057	0.05	-0.01	0.11	0.105	2057	0.02	-0.04	0.08	0.452
Lactate (mmol/l)	2439	-0.04	-0.08	0.00	0.065	2439	-0.06	-0.11	-0.02	0.007	2057	-0.07	-0.14	-0.01	0.028	2057	-0.08	-0.15	-0.02	0.016
Pyruvate (mmol/l)	2439	0.04	-0.01	0.08	0.114	2439	0.00	-0.05	0.04	0.954	2057	0.02	-0.05	0.09	0.504	2057	-0.01	-0.08	0.06	0.721
Citrate (mmol/l)	2439	-0.08	-0.12	-0.03	0.001	2439	-0.03	-0.08	0.02	0.187	2057	-0.10	-0.17	-0.03	0.005	2057	-0.07	-0.14	0.00	0.063
Alanine (mmol/l)	2439	0.03	-0.01	0.08	0.152	2439	0.03	-0.02	0.08	0.283	2057	0.10	0.03	0.17	0.007	2057	0.07	0.00	0.15	0.044
Glutamine (mmol/l)	2439	-0.04	-0.09	0.00	0.044	2439	-0.03	-0.08	0.01	0.136	2057	-0.06	-0.12	0.00	0.057	2057	-0.06	-0.12	0.01	0.084
Histidine (mmol/l)	2439	0.03	-0.02	0.08	0.194	2439	0.03	-0.02	0.07	0.288	2057	0.05	-0.02	0.12	0.160	2057	0.04	-0.03	0.12	0.250
Isoleucine (mmol/l)	2439	0.10	0.05	0.15	<0.0001	2439	0.07	0.02	0.11	0.005	2057	0.19	0.12	0.26	<0.0001	2057	0.14	0.07	0.21	<0.0001
Leucine (mmol/l)	2439	0.11	0.07	0.15	<0.0001	2439	0.10	0.05	0.14	<0.0001	2057	0.21	0.15	0.27	<0.0001	2057	0.19	0.12	0.25	<0.0001
Valine (mmol/l)	2439	0.12	0.08	0.17	<0.0001	2439	0.09	0.04	0.13	0.0002	2057	0.21	0.13	0.28	<0.0001	2057	0.16	0.09	0.24	<0.0001
Phenylalanine (mmol/l)	2438	0.10	0.05	0.15	<0.0001	2438	0.07	0.02	0.12	0.003	2057	0.17	0.10	0.24	<0.0001	2057	0.13	0.06	0.20	0.0002
Tyrosine (mmol/l)	2439	0.09	0.04	0.14	0.0001	2439	0.05	0.01	0.10	0.025	2057	0.15	0.08	0.22	<0.0001	2057	0.09	0.02	0.15	0.007
Acetate (mmol/l)	2438	-0.05	-0.10	0.01	0.095	2438	-0.03	-0.09	0.02	0.249	2057	-0.06	-0.12	0.00	0.034	2057	-0.04	-0.10	0.01	0.122
Acetoacetate (mmol/l)	2439	-0.08	-0.13	-0.03	0.001	2439	-0.09	-0.15	-0.04	0.001	2057	-0.12	-0.18	-0.06	0.0002	2057	-0.11	-0.17	-0.05	0.001
3-hydroxybutyrate (mmol/l)	2436	-0.10	-0.14	-0.05	<0.0001	2436	-0.11	-0.16	-0.06	<0.0001	2054	-0.15	-0.22	-0.08	<0.0001	2054	-0.14	-0.21	-0.07	<0.0001
Creatinine (mmol/l)	2439	0.15	0.11	0.19	<0.0001	2439	0.16	0.12	0.21	<0.0001	2057	0.30	0.23	0.36	<0.0001	2057	0.31	0.24	0.37	<0.0001
Albumin (signal area)	2440	0.00	-0.05	0.04	0.830	2440	0.02	-0.03	0.06	0.526	2058	-0.05	-0.12	0.02	0.155	2058	-0.03	-0.10	0.04	0.387

Online Table 5 Associations of lean mass index at age 10y and 18y with cardiometabolic traits at age 18y in ALSPAC

	At age 10y										At age 18y									
	Lean mass index (per 1.0 kg/m² higher)										Lean mass index (per 2.2 kg/m² higher)									
	Adj. for age, sex, ethnicity, maternal education					*Additionally adj. for fat mass index at 10y*					*Adj. for age, sex, ethnicity, maternal education, smoking, alcohol, puberty timing*					*Additionally adj. for fat mass index at 18y*				
**Standardized outcome at age 18y**	**N**	**Beta**	**LCL**	**UCL**	**P-value**	**N**	**Beta**	**LCL**	**UCL**	**P-value**	**N**	**Beta**	**LCL**	**UCL**	**P-value**	**N**	**Beta**	**LCL**	**UCL**	**P-value**
Glycoprotein acetyls, mainly a1-acid glycoprotein (mmol/l)	2439	0.07	0.02	0.12	0.004	2439	0.01	-0.04	0.06	0.752	2057	0.09	0.02	0.16	0.014	2057	0.01	-0.06	0.07	0.828
C-reactive protein (mg/l)	2519	0.02	0.00	0.05	0.070	2519	0.00	-0.03	0.03	0.857	2124	0.01	-0.03	0.06	0.617	2124	-0.01	-0.06	0.03	0.568

Complete case sample

	At age 10y										At age 18y									
	Lean mass index (per 0.9 kg/m² higher)										Lean mass index (per 2.1 kg/m² higher)									
	Adj. for age, sex, ethnicity, maternal education					*Additionally adj. for fat mass index at 10y*					*Adj. for age, sex, ethnicity, maternal education, smoking, alcohol, puberty timing*					*Additionally adj. for fat mass index at 18y*				
**Standardized outcome at age 18y**	**N**	**Beta**	**LCL**	**UCL**	**P-value**	**N**	**Beta**	**LCL**	**UCL**	**P-value**	**N**	**Beta**	**LCL**	**UCL**	**P-value**	**N**	**Beta**	**LCL**	**UCL**	**P-value**
Systolic blood pressure (mmHg)	1722	0.20	0.15	0.25	<0.0001	1722	0.16	0.11	0.21	<0.0001	1722	0.36	0.29	0.42	<0.0001	1722	0.31	0.25	0.38	<0.0001
Diastolic blood pressure (mmHg)	1722	0.09	0.03	0.15	0.002	1722	0.02	-0.04	0.07	0.595	1722	0.05	-0.04	0.14	0.257	1722	-0.02	-0.10	0.06	0.563
Concentration of chylomicrons and extremely large VLDL particles (mol/l)	1722	0.08	0.01	0.15	0.018	1722	0.03	-0.04	0.09	0.460	1722	0.16	0.08	0.25	0.0002	1722	0.08	0.00	0.15	0.055
Total lipids in chylomicrons and extremely large VLDL (mmol/l)	1722	0.08	0.01	0.14	0.019	1722	0.02	-0.04	0.09	0.470	1722	0.16	0.07	0.24	0.0003	1722	0.07	0.00	0.15	0.057
Phospholipids in chylomicrons and extremely large VLDL (mmol/l)	1722	0.08	0.01	0.14	0.024	1722	0.02	-0.04	0.09	0.529	1722	0.15	0.07	0.23	0.0005	1722	0.07	-0.01	0.14	0.080
Total cholesterol in chylomicrons and extremely large VLDL (mmol/l)	1722	0.08	0.01	0.14	0.020	1722	0.03	-0.04	0.09	0.418	1722	0.17	0.08	0.25	0.0001	1722	0.09	0.01	0.16	0.031
Cholesterol esters in chylomicrons and extremely large VLDL (mmol/l)	1722	0.08	0.01	0.14	0.022	1722	0.03	-0.04	0.10	0.380	1722	0.17	0.09	0.26	<0.0001	1722	0.09	0.01	0.17	0.020
Free cholesterol in chylomicrons and extremely large VLDL (mmol/l)	1722	0.08	0.01	0.14	0.021	1722	0.02	-0.04	0.09	0.476	1722	0.15	0.07	0.24	0.0003	1722	0.07	0.00	0.15	0.062
Triglycerides in chylomicrons and extremely large VLDL (mmol/l)	1722	0.08	0.01	0.14	0.019	1722	0.02	-0.04	0.09	0.480	1722	0.16	0.07	0.24	0.0003	1722	0.07	0.00	0.15	0.064
Concentration of very large VLDL particles (mol/l)	1722	0.08	0.01	0.14	0.017	1722	0.03	-0.04	0.09	0.441	1722	0.17	0.09	0.25	<0.0001	1722	0.09	0.01	0.16	0.023
Total lipids in very large VLDL (mmol/l)	1722	0.08	0.01	0.14	0.018	1722	0.02	-0.04	0.09	0.445	1722	0.17	0.09	0.25	<0.0001	1722	0.09	0.01	0.16	0.022
Phospholipids in very large VLDL (mmol/l)	1722	0.07	0.01	0.14	0.024	1722	0.02	-0.04	0.09	0.493	1722	0.16	0.08	0.24	0.0001	1722	0.08	0.01	0.16	0.036
Total cholesterol in very large VLDL (mmol/l)	1722	0.08	0.02	0.15	0.016	1722	0.03	-0.04	0.09	0.419	1722	0.17	0.09	0.26	<0.0001	1722	0.09	0.01	0.16	0.025
Cholesterol esters in very large VLDL (mmol/l)	1722	0.08	0.02	0.15	0.012	1722	0.03	-0.04	0.09	0.373	1722	0.18	0.10	0.27	<0.0001	1722	0.10	0.02	0.17	0.013
Free cholesterol in very large VLDL (mmol/l)	1722	0.08	0.01	0.14	0.022	1722	0.02	-0.04	0.09	0.480	1722	0.16	0.08	0.24	0.0002	1722	0.08	0.00	0.15	0.049
Triglycerides in very large VLDL (mmol/l)	1722	0.08	0.01	0.14	0.018	1722	0.02	-0.04	0.09	0.444	1722	0.17	0.09	0.25	<0.0001	1722	0.09	0.01	0.16	0.019
Concentration of large VLDL particles (mol/l)	1722	0.07	0.01	0.14	0.020	1722	0.02	-0.04	0.08	0.475	1722	0.17	0.09	0.25	<0.0001	1722	0.09	0.02	0.16	0.015
Total lipids in large VLDL (mmol/l)	1722	0.08	0.01	0.14	0.018	1722	0.02	-0.04	0.09	0.453	1722	0.17	0.09	0.25	<0.0001	1722	0.09	0.02	0.17	0.014
Phospholipids in large VLDL (mmol/l)	1722	0.07	0.01	0.14	0.019	1722	0.02	-0.04	0.09	0.459	1722	0.17	0.09	0.25	<0.0001	1722	0.09	0.02	0.16	0.016
Total cholesterol in large VLDL (mmol/l)	1722	0.08	0.01	0.14	0.017	1722	0.03	-0.04	0.09	0.413	1722	0.18	0.09	0.26	<0.0001	1722	0.10	0.02	0.17	0.012
Cholesterol esters in large VLDL (mmol/l)	1722	0.08	0.02	0.14	0.014	1722	0.03	-0.03	0.09	0.372	1722	0.18	0.10	0.26	<0.0001	1722	0.10	0.03	0.18	0.009
Free cholesterol in large VLDL (mmol/l)	1722	0.07	0.01	0.14	0.021	1722	0.02	-0.04	0.09	0.460	1722	0.17	0.09	0.25	<0.0001	1722	0.09	0.02	0.16	0.018
Triglycerides in large VLDL (mmol/l)	1722	0.07	0.01	0.14	0.019	1722	0.02	-0.04	0.08	0.471	1722	0.17	0.09	0.25	<0.0001	1722	0.09	0.02	0.16	0.014
Concentration of medium VLDL particles (mol/l)	1722	0.08	0.01	0.14	0.015	1722	0.03	-0.04	0.09	0.418	1722	0.18	0.10	0.26	<0.0001	1722	0.10	0.02	0.17	0.010
Total lipids in medium VLDL (mmol/l)	1722	0.08	0.02	0.14	0.013	1722	0.03	-0.03	0.09	0.384	1722	0.18	0.10	0.26	<0.0001	1722	0.10	0.03	0.17	0.008
Phospholipids in medium VLDL (mmol/l)	1722	0.08	0.01	0.14	0.016	1722	0.03	-0.03	0.09	0.386	1722	0.17	0.09	0.25	<0.0001	1722	0.09	0.02	0.17	0.012
Total cholesterol in medium VLDL (mmol/l)	1722	0.08	0.02	0.14	0.012	1722	0.03	-0.03	0.10	0.282	1722	0.18	0.10	0.26	<0.0001	1722	0.10	0.03	0.18	0.008
Cholesterol esters in medium VLDL (mmol/l)	1722	0.08	0.02	0.14	0.011	1722	0.04	-0.02	0.10	0.230	1722	0.18	0.10	0.27	<0.0001	1722	0.11	0.03	0.18	0.006
Free cholesterol in medium VLDL (mmol/l)	1722	0.08	0.01	0.14	0.018	1722	0.03	-0.04	0.09	0.395	1722	0.17	0.09	0.25	<0.0001	1722	0.09	0.02	0.16	0.016
Triglycerides in medium VLDL (mmol/l)	1722	0.08	0.01	0.14	0.015	1722	0.02	-0.04	0.08	0.460	1722	0.17	0.10	0.25	<0.0001	1722	0.10	0.02	0.17	0.009
Concentration of small VLDL particles (mol/l)	1722	0.06	0.00	0.13	0.043	1722	0.02	-0.04	0.08	0.545	1722	0.15	0.07	0.23	0.0003	1722	0.08	0.00	0.15	0.045
Total lipids in small VLDL (mmol/l)	1722	0.06	0.00	0.12	0.053	1722	0.02	-0.05	0.08	0.600	1722	0.14	0.06	0.23	0.0005	1722	0.07	-0.01	0.15	0.071
Phospholipids in small VLDL (mmol/l)	1722	0.05	-0.01	0.11	0.122	1722	0.01	-0.06	0.07	0.846	1722	0.13	0.05	0.21	0.002	1722	0.06	-0.01	0.14	0.110
Total cholesterol in small VLDL (mmol/l)	1722	0.06	-0.01	0.12	0.073	1722	0.02	-0.05	0.08	0.593	1722	0.13	0.05	0.21	0.002	1722	0.05	-0.02	0.13	0.168
Cholesterol esters in small VLDL (mmol/l)	1722	0.06	-0.01	0.12	0.073	1722	0.02	-0.05	0.09	0.548	1722	0.12	0.04	0.20	0.004	1722	0.05	-0.03	0.13	0.226
Free cholesterol in small VLDL (mmol/l)	1722	0.05	-0.01	0.11	0.104	1722	0.01	-0.05	0.08	0.717	1722	0.13	0.05	0.21	0.002	1722	0.06	-0.02	0.14	0.117
Triglycerides in small VLDL (mmol/l)	1722	0.06	0.00	0.12	0.043	1722	0.02	-0.04	0.08	0.554	1722	0.15	0.07	0.23	0.0004	1722	0.08	0.00	0.15	0.044
Concentration of very small VLDL particles (mol/l)	1722	0.03	-0.03	0.09	0.266	1722	0.01	-0.05	0.08	0.710	1722	0.07	-0.01	0.15	0.071	1722	0.03	-0.05	0.10	0.515
Total lipids in very small VLDL (mmol/l)	1722	0.04	-0.02	0.10	0.224	1722	0.01	-0.05	0.08	0.724	1722	0.08	0.00	0.16	0.044	1722	0.03	-0.05	0.11	0.470
Phospholipids in very small VLDL (mmol/l)	1722	0.03	-0.03	0.09	0.358	1722	0.01	-0.05	0.07	0.777	1722	0.05	-0.03	0.13	0.214	1722	0.01	-0.07	0.09	0.840
Total cholesterol in very small VLDL (mmol/l)	1722	0.03	-0.03	0.09	0.266	1722	0.01	-0.05	0.07	0.762	1722	0.08	0.00	0.17	0.044	1722	0.03	-0.05	0.12	0.416
Cholesterol esters in very small VLDL (mmol/l)	1722	0.04	-0.02	0.10	0.170	1722	0.01	-0.05	0.08	0.656	1722	0.09	0.01	0.18	0.025	1722	0.04	-0.04	0.12	0.364
Free cholesterol in very small VLDL (mmol/l)	1722	0.01	-0.05	0.07	0.714	1722	0.00	-0.07	0.06	0.931	1722	0.05	-0.03	0.13	0.195	1722	0.02	-0.06	0.10	0.598

Online Table 5 Associations of lean mass index at age 10y and 18y with cardiometabolic traits at age 18y in ALSPAC

	At age 10y										At age 18y									
	**Lean mass index (per 1.0 kg/m^2 higher)**										**Lean mass index (per 2.2 kg/m^2 higher)**									
	Adj. for age, sex, ethnicity, maternal education					*Additionally adj. for fat mass index at 10y*					*Adj. for age, sex, ethnicity, maternal education, smoking, alcohol, puberty timing*					*Additionally adj. for fat mass index at 18y*				
**Standardized outcome at age 18y**	**N**	**Beta**	**LCL**	**UCL**	**P-value**	**N**	**Beta**	**LCL**	**UCL**	**P-value**	**N**	**Beta**	**LCL**	**UCL**	**P-value**	**N**	**Beta**	**LCL**	**UCL**	**P-value**
Triglycerides in very small VLDL (mmol/l)	1722	0.04	-0.02	0.10	0.187	1722	0.01	-0.05	0.08	0.677	1722	0.08	0.00	0.16	0.044	1722	0.03	-0.04	0.11	0.403
Concentration of IDL particles (mol/l)	1722	0.03	-0.03	0.08	0.398	1722	0.01	-0.05	0.07	0.712	1722	0.03	-0.05	0.11	0.466	1722	0.00	-0.08	0.07	0.925
Total lipids in IDL (mmol/l)	1722	0.02	-0.03	0.08	0.428	1722	0.01	-0.05	0.07	0.814	1722	0.03	-0.04	0.11	0.394	1722	0.00	-0.08	0.08	0.957
Phospholipids in IDL (mmol/l)	1722	0.02	-0.04	0.08	0.533	1722	0.01	-0.06	0.07	0.870	1722	0.02	-0.06	0.09	0.690	1722	-0.01	-0.09	0.06	0.724
Total cholesterol in IDL (mmol/l)	1722	0.03	-0.03	0.08	0.389	1722	0.01	-0.06	0.07	0.823	1722	0.05	-0.03	0.13	0.258	1722	0.01	-0.08	0.09	0.897
Cholesterol esters in IDL (mmol/l)	1722	0.03	-0.03	0.09	0.299	1722	0.01	-0.05	0.07	0.779	1722	0.06	-0.02	0.14	0.133	1722	0.01	-0.07	0.10	0.723
Free cholesterol in IDL (mmol/l)	1722	0.01	-0.05	0.07	0.696	1722	0.00	-0.06	0.06	0.938	1722	0.01	-0.07	0.08	0.866	1722	-0.02	-0.10	0.06	0.671
Triglycerides in IDL (mmol/l)	1722	0.01	-0.04	0.07	0.644	1722	0.01	-0.05	0.07	0.731	1722	-0.01	-0.08	0.06	0.856	1722	-0.02	-0.09	0.05	0.629
Concentration of large LDL particles (mol/l)	1722	0.02	-0.04	0.07	0.558	1722	0.00	-0.06	0.06	0.938	1722	0.02	-0.06	0.09	0.629	1722	-0.02	-0.09	0.06	0.668
Total lipids in large LDL (mmol/l)	1722	0.02	-0.04	0.07	0.548	1722	0.00	-0.06	0.06	0.949	1722	0.02	-0.05	0.10	0.569	1722	-0.01	-0.09	0.06	0.713
Phospholipids in large LDL (mmol/l)	1722	0.02	-0.04	0.08	0.485	1722	0.00	-0.06	0.06	0.941	1722	0.03	-0.04	0.11	0.416	1722	-0.01	-0.09	0.07	0.805
Total cholesterol in large LDL (mmol/l)	1722	0.02	-0.04	0.08	0.549	1722	0.00	-0.06	0.06	0.925	1722	0.03	-0.05	0.10	0.519	1722	-0.01	-0.09	0.06	0.744
Cholesterol esters in large LDL (mmol/l)	1722	0.02	-0.04	0.08	0.489	1722	0.00	-0.06	0.06	0.945	1722	0.03	-0.04	0.11	0.419	1722	-0.01	-0.09	0.07	0.810
Free cholesterol in large LDL (mmol/l)	1722	0.01	-0.05	0.07	0.758	1722	0.00	-0.07	0.06	0.873	1722	0.00	-0.07	0.08	0.902	1722	-0.02	-0.10	0.05	0.552
Triglycerides in large LDL (mmol/l)	1722	0.01	-0.05	0.06	0.815	1722	0.01	-0.05	0.06	0.852	1722	-0.03	-0.10	0.04	0.459	1722	-0.03	-0.10	0.04	0.397
Concentration of medium LDL particles (mol/l)	1722	0.02	-0.04	0.08	0.515	1722	-0.01	-0.07	0.06	0.871	1722	0.02	-0.05	0.10	0.518	1722	-0.02	-0.09	0.06	0.684
Total lipids in medium LDL (mmol/l)	1722	0.02	-0.04	0.07	0.577	1722	-0.01	-0.07	0.05	0.820	1722	0.02	-0.05	0.10	0.537	1722	-0.02	-0.09	0.06	0.678
Phospholipids in medium LDL (mmol/l)	1722	0.02	-0.04	0.07	0.607	1722	-0.02	-0.08	0.04	0.596	1722	0.03	-0.04	0.11	0.390	1722	-0.02	-0.09	0.06	0.667
Total cholesterol in medium LDL (mmol/l)	1722	0.02	-0.04	0.08	0.525	1722	0.00	-0.07	0.06	0.896	1722	0.03	-0.05	0.10	0.452	1722	-0.01	-0.09	0.07	0.784
Cholesterol esters in medium LDL (mmol/l)	1722	0.02	-0.04	0.08	0.462	1722	0.00	-0.06	0.06	0.979	1722	0.03	-0.04	0.11	0.399	1722	-0.01	-0.08	0.07	0.848
Free cholesterol in medium LDL (mmol/l)	1722	0.01	-0.05	0.06	0.857	1722	-0.02	-0.08	0.04	0.550	1722	0.01	-0.06	0.09	0.727	1722	-0.02	-0.10	0.05	0.534
Triglycerides in medium LDL (mmol/l)	1722	0.00	-0.05	0.05	0.949	1722	0.00	-0.06	0.05	0.880	1722	-0.04	-0.11	0.02	0.203	1722	-0.05	-0.12	0.02	0.164
Concentration of small LDL particles (mol/l)	1722	0.01	-0.05	0.07	0.765	1722	-0.02	-0.08	0.04	0.537	1722	0.01	-0.06	0.09	0.692	1722	-0.02	-0.10	0.05	0.513
Total lipids in small LDL (mmol/l)	1722	0.01	-0.05	0.07	0.678	1722	-0.01	-0.08	0.05	0.649	1722	0.02	-0.05	0.10	0.578	1722	-0.02	-0.10	0.06	0.604
Phospholipids in small LDL (mmol/l)	1722	0.01	-0.05	0.06	0.813	1722	-0.03	-0.09	0.04	0.417	1722	0.02	-0.05	0.10	0.516	1722	-0.02	-0.10	0.05	0.589
Total cholesterol in small LDL (mmol/l)	1722	0.01	-0.05	0.07	0.674	1722	-0.01	-0.07	0.05	0.703	1722	0.02	-0.06	0.10	0.600	1722	-0.02	-0.10	0.06	0.622
Cholesterol esters in small LDL (mmol/l)	1722	0.02	-0.04	0.07	0.571	1722	-0.01	-0.07	0.05	0.828	1722	0.02	-0.05	0.10	0.553	1722	-0.02	-0.09	0.06	0.668
Free cholesterol in small LDL (mmol/l)	1722	-0.01	-0.07	0.05	0.801	1722	-0.03	-0.10	0.03	0.270	1722	0.01	-0.07	0.09	0.828	1722	-0.03	-0.11	0.05	0.456
Triglycerides in small LDL (mmol/l)	1722	0.02	-0.04	0.07	0.513	1722	0.00	-0.06	0.06	0.983	1722	0.01	-0.06	0.08	0.754	1722	-0.02	-0.09	0.05	0.614
Concentration of very large HDL particles (mol/l)	1722	-0.05	-0.10	0.00	0.062	1722	-0.02	-0.08	0.03	0.380	1722	-0.14	-0.22	-0.07	0.0002	1722	-0.10	-0.17	-0.02	0.010
Total lipids in very large HDL (mmol/l)	1722	-0.06	-0.11	0.00	0.044	1722	-0.03	-0.09	0.02	0.254	1722	-0.14	-0.22	-0.07	0.0003	1722	-0.10	-0.18	-0.02	0.010
Phospholipids in very large HDL (mmol/l)	1722	-0.05	-0.10	0.00	0.054	1722	-0.02	-0.08	0.03	0.450	1722	-0.15	-0.22	-0.07	<0.0001	1722	-0.10	-0.17	-0.03	0.007
Total cholesterol in very large HDL (mmol/l)	1722	-0.06	-0.12	0.00	0.036	1722	-0.05	-0.11	0.01	0.112	1722	-0.13	-0.22	-0.05	0.002	1722	-0.10	-0.19	-0.02	0.016
Cholesterol esters in very large HDL (mmol/l)	1722	-0.06	-0.12	0.00	0.038	1722	-0.05	-0.11	0.01	0.094	1722	-0.13	-0.21	-0.04	0.003	1722	-0.10	-0.19	-0.02	0.021
Free cholesterol in very large HDL (mmol/l)	1722	-0.06	-0.11	0.00	0.044	1722	-0.04	-0.09	0.02	0.226	1722	-0.14	-0.22	-0.06	0.0004	1722	-0.10	-0.18	-0.02	0.010
Triglycerides in very large HDL (mmol/l)	1722	0.04	-0.02	0.10	0.192	1722	0.05	-0.02	0.11	0.147	1722	0.03	-0.05	0.11	0.419	1722	0.03	-0.05	0.11	0.474
Concentration of large HDL particles (mol/l)	1722	-0.06	-0.11	-0.01	0.031	1722	-0.03	-0.08	0.03	0.344	1722	-0.14	-0.21	-0.07	0.0001	1722	-0.08	-0.15	-0.01	0.020
Total lipids in large HDL (mmol/l)	1722	-0.06	-0.11	-0.01	0.025	1722	-0.03	-0.08	0.03	0.306	1722	-0.14	-0.21	-0.07	<0.0001	1722	-0.08	-0.15	-0.02	0.015
Phospholipids in large HDL (mmol/l)	1722	-0.06	-0.11	0.00	0.034	1722	-0.03	-0.08	0.02	0.284	1722	-0.13	-0.20	-0.06	0.0002	1722	-0.08	-0.15	-0.01	0.017
Total cholesterol in large HDL (mmol/l)	1722	-0.07	-0.12	-0.01	0.017	1722	-0.03	-0.09	0.03	0.287	1722	-0.15	-0.23	-0.08	<0.0001	1722	-0.09	-0.16	-0.02	0.010
Cholesterol esters in large HDL (mmol/l)	1722	-0.07	-0.12	-0.01	0.016	1722	-0.03	-0.09	0.03	0.291	1722	-0.15	-0.23	-0.08	<0.0001	1722	-0.09	-0.16	-0.02	0.010
Free cholesterol in large HDL (mmol/l)	1722	-0.07	-0.12	-0.01	0.016	1722	-0.03	-0.09	0.02	0.250	1722	-0.15	-0.22	-0.08	<0.0001	1722	-0.09	-0.16	-0.02	0.011
Triglycerides in large HDL (mmol/l)	1722	0.04	-0.01	0.09	0.134	1722	0.05	0.00	0.11	0.070	1722	0.06	-0.01	0.12	0.104	1722	0.06	-0.01	0.13	0.078
Concentration of medium HDL particles (mol/l)	1722	-0.04	-0.09	0.01	0.135	1722	-0.05	-0.10	0.01	0.103	1722	-0.05	-0.12	0.02	0.128	1722	-0.04	-0.11	0.03	0.253
Total lipids in medium HDL (mmol/l)	1722	-0.05	-0.10	0.01	0.095	1722	-0.05	-0.11	0.01	0.087	1722	-0.07	-0.14	0.01	0.069	1722	-0.05	-0.12	0.02	0.175
Phospholipids in medium HDL (mmol/l)	1722	-0.05	-0.10	0.01	0.077	1722	-0.05	-0.11	0.01	0.083	1722	-0.08	-0.15	-0.01	0.030	1722	-0.06	-0.13	0.01	0.103
Total cholesterol in medium HDL (mmol/l)	1722	-0.05	-0.11	0.00	0.055	1722	-0.05	-0.11	0.00	0.066	1722	-0.08	-0.16	0.00	0.050	1722	-0.06	-0.13	0.02	0.163
Cholesterol esters in medium HDL (mmol/l)	1722	-0.06	-0.11	0.00	0.046	1722	-0.06	-0.11	0.00	0.059	1722	-0.08	-0.16	0.00	0.039	1722	-0.06	-0.14	0.02	0.144
Free cholesterol in medium HDL (mmol/l)	1722	-0.04	-0.10	0.01	0.129	1722	-0.05	-0.10	0.01	0.105	1722	-0.05	-0.13	0.02	0.150	1722	-0.04	-0.12	0.03	0.259
Triglycerides in medium HDL (mmol/l)	1722	0.07	0.01	0.13	0.023	1722	0.03	-0.03	0.09	0.269	1722	0.15	0.07	0.22	0.0002	1722	0.09	0.02	0.17	0.015
Concentration of small HDL particles (mol/l)	1722	-0.02	-0.08	0.03	0.418	1722	-0.05	-0.11	0.01	0.093	1722	-0.01	-0.09	0.07	0.810	1722	-0.02	-0.10	0.05	0.546
Total lipids in small HDL (mmol/l)	1722	-0.04	-0.09	0.02	0.170	1722	-0.06	-0.11	0.00	0.061	1722	-0.04	-0.12	0.03	0.224	1722	-0.05	-0.12	0.03	0.202
Phospholipids in small HDL (mmol/l)	1722	-0.02	-0.08	0.04	0.479	1722	-0.04	-0.10	0.02	0.150	1722	-0.01	-0.09	0.08	0.894	1722	-0.01	-0.09	0.07	0.793
Total cholesterol in small HDL (mmol/l)	1722	-0.06	-0.11	-0.01	0.020	1722	-0.06	-0.12	-0.01	0.019	1722	-0.10	-0.16	-0.03	0.004	1722	-0.09	-0.15	-0.02	0.008
Cholesterol esters in small HDL (mmol/l)	1722	-0.06	-0.11	-0.01	0.022	1722	-0.06	-0.11	-0.01	0.022	1722	-0.09	-0.16	-0.03	0.003	1722	-0.09	-0.15	-0.03	0.004
Free cholesterol in small HDL (mmol/l)	1722	-0.05	-0.11	0.01	0.090	1722	-0.05	-0.11	0.01	0.080	1722	-0.06	-0.14	0.01	0.107	1722	-0.04	-0.12	0.03	0.271
Triglycerides in small HDL (mmol/l)	1722	0.06	0.00	0.12	0.059	1722	0.03	-0.03	0.09	0.332	1722	0.10	0.02	0.18	0.012	1722	0.06	-0.02	0.14	0.122
Phospholipids to total lipds ratio in chylomicrons and extremely large VLDL (%)	1722	0.01	-0.02	0.04	0.460	1722	-0.01	-0.04	0.03	0.763	1722	-0.01	-0.05	0.03	0.573	1722	-0.02	-0.06	0.02	0.385

Online Table 5 Associations of lean mass index at age 10y and 18y with cardiometabolic traits at age 18y in ALSPAC

	**At age 10y**										**At age 18y**									
	**Lean mass index (per 1.0 kg/m^2 higher)**										**Lean mass index (per 2.2 kg/m^2 higher)**									
	Adj. for age, sex, ethnicity, maternal education					*Additionally adj. for fat mass index at 10y*					*Adj. for age, sex, ethnicity, maternal education, smoking, alcohol, puberty timing*					*Additionally adj. for fat mass index at 18y*				
**Standardized outcome at age 18y**	**N**	**Beta**	**LCL**	**UCL**	**P-value**	**N**	**Beta**	**LCL**	**UCL**	**P-value**	**N**	**Beta**	**LCL**	**UCL**	**P-value**	**N**	**Beta**	**LCL**	**UCL**	**P-value**
Total cholesterol to total lipids ratio in chylomicrons and extremely large VLDL (%)	1722	0.06	-0.01	0.13	0.101	1722	0.05	-0.03	0.13	0.189	1722	0.11	0.04	0.19	0.004	1722	0.09	0.01	0.16	0.031
Cholesterol esters to total lipids ratio in chylomicrons and extremely large VLDL (%)	1722	0.05	-0.03	0.12	0.224	1722	0.05	-0.03	0.14	0.218	1722	0.10	0.02	0.17	0.013	1722	0.08	0.00	0.16	0.050
Free cholesterol to total lipids ratio in chylomicrons and extremely large VLDL (%)	1722	0.07	0.00	0.13	0.036	1722	0.03	-0.04	0.09	0.423	1722	0.07	-0.01	0.14	0.103	1722	0.03	-0.05	0.11	0.447
Triglycerides to total lipids ratio in chylomicrons and extremely large VLDL (%)	1722	-0.08	-0.14	-0.01	0.028	1722	-0.07	-0.14	0.01	0.087	1722	-0.11	-0.18	-0.04	0.003	1722	-0.08	-0.16	-0.01	0.025
Phospholipids to total lipds ratio in very large VLDL (%)	1722	0.05	-0.01	0.10	0.092	1722	0.01	-0.05	0.07	0.816	1722	0.08	0.00	0.15	0.039	1722	0.03	-0.05	0.10	0.458
Total cholesterol to total lipids ratio in very large VLDL (%)	1722	0.02	-0.04	0.08	0.560	1722	0.04	-0.03	0.11	0.287	1722	0.04	-0.04	0.11	0.368	1722	0.05	-0.03	0.13	0.244
Cholesterol esters to total lipids ratio in very large VLDL (%)	1722	0.00	-0.05	0.05	0.961	1722	0.02	-0.04	0.07	0.539	1722	0.02	-0.04	0.09	0.428	1722	0.04	-0.03	0.10	0.244
Free cholesterol to total lipids ratio in very large VLDL (%)	1722	0.00	-0.03	0.04	0.911	1722	0.00	-0.04	0.04	0.893	1722	0.03	-0.03	0.09	0.353	1722	0.03	-0.03	0.09	0.358
Triglycerides to total lipids ratio in very large VLDL (%)	1722	0.00	-0.06	0.06	0.927	1722	-0.01	-0.07	0.06	0.827	1722	-0.07	-0.15	0.01	0.100	1722	-0.06	-0.14	0.02	0.147
Phospholipids to total lipds ratio in large VLDL (%)	1722	0.06	0.00	0.11	0.054	1722	0.03	-0.03	0.09	0.381	1722	0.06	-0.02	0.13	0.134	1722	0.02	-0.05	0.10	0.580
Total cholesterol to total lipids ratio in large VLDL (%)	1722	0.09	0.03	0.15	0.005	1722	0.07	0.01	0.14	0.027	1722	0.15	0.07	0.24	0.001	1722	0.12	0.03	0.21	0.012
Cholesterol esters to total lipids ratio in large VLDL (%)	1722	0.07	0.01	0.13	0.020	1722	0.08	0.01	0.15	0.018	1722	0.14	0.04	0.23	0.005	1722	0.13	0.03	0.23	0.013
Free cholesterol to total lipids ratio in large VLDL (%)	1722	0.03	0.00	0.05	0.045	1722	0.01	-0.02	0.04	0.474	1722	0.04	0.00	0.07	0.029	1722	0.02	-0.02	0.05	0.365
Triglycerides to total lipids ratio in large VLDL (%)	1722	-0.08	-0.14	-0.02	0.007	1722	-0.06	-0.13	0.00	0.050	1722	-0.13	-0.21	-0.05	0.001	1722	-0.09	-0.17	-0.01	0.024
Phospholipids to total lipds ratio in medium VLDL (%)	1722	-0.03	-0.08	0.02	0.245	1722	-0.01	-0.07	0.05	0.724	1722	-0.08	-0.16	-0.01	0.033	1722	-0.04	-0.12	0.03	0.278
Total cholesterol to total lipids ratio in medium VLDL (%)	1722	0.05	0.00	0.10	0.071	1722	0.05	-0.01	0.10	0.109	1722	0.10	0.02	0.17	0.011	1722	0.08	0.00	0.15	0.052
Cholesterol esters to total lipids ratio in medium VLDL (%)	1722	0.04	-0.01	0.10	0.097	1722	0.05	-0.01	0.10	0.088	1722	0.10	0.02	0.17	0.011	1722	0.08	0.00	0.16	0.042
Free cholesterol to total lipids ratio in medium VLDL (%)	1722	0.03	-0.03	0.09	0.268	1722	0.01	-0.05	0.08	0.724	1722	0.04	-0.03	0.11	0.247	1722	0.02	-0.05	0.09	0.625
Triglycerides to total lipids ratio in medium VLDL (%)	1722	-0.04	-0.09	0.01	0.147	1722	-0.04	-0.10	0.02	0.152	1722	-0.07	-0.15	0.00	0.054	1722	-0.06	-0.14	0.01	0.115
Phospholipids to total lipds ratio in small VLDL (%)	1722	-0.08	-0.14	-0.02	0.005	1722	-0.05	-0.11	0.01	0.082	1722	-0.14	-0.21	-0.06	0.0003	1722	-0.08	-0.15	0.00	0.042
Total cholesterol to total lipids ratio in small VLDL (%)	1722	0.01	-0.05	0.06	0.768	1722	0.02	-0.04	0.07	0.569	1722	0.00	-0.08	0.08	0.987	1722	-0.01	-0.09	0.08	0.886
Cholesterol esters to total lipids ratio in small VLDL (%)	1722	0.02	-0.04	0.07	0.531	1722	0.02	-0.04	0.08	0.449	1722	0.01	-0.07	0.10	0.725	1722	0.00	-0.08	0.08	0.970
Free cholesterol to total lipids ratio in small VLDL (%)	1722	-0.06	-0.11	-0.01	0.025	1722	-0.03	-0.09	0.02	0.232	1722	-0.10	-0.17	-0.03	0.004	1722	-0.05	-0.12	0.02	0.149
Triglycerides to total lipids ratio in small VLDL (%)	1722	0.02	-0.04	0.07	0.507	1722	0.00	-0.06	0.06	0.971	1722	0.05	-0.03	0.13	0.244	1722	0.03	-0.05	0.11	0.452
Phospholipids to total lipds ratio in very small VLDL (%)	1722	0.00	-0.05	0.05	0.899	1722	0.00	-0.05	0.06	0.908	1722	-0.02	-0.09	0.05	0.508	1722	-0.03	-0.10	0.05	0.483
Total cholesterol to total lipids ratio in very small VLDL (%)	1722	-0.01	-0.06	0.05	0.850	1722	0.00	-0.06	0.06	0.996	1722	0.01	-0.06	0.08	0.774	1722	0.01	-0.05	0.08	0.674
Cholesterol esters to total lipids ratio in very small VLDL (%)	1722	0.01	-0.04	0.07	0.620	1722	0.01	-0.05	0.07	0.734	1722	0.03	-0.04	0.10	0.358	1722	0.02	-0.05	0.09	0.563
Free cholesterol to total lipids ratio in very small VLDL (%)	1722	-0.07	-0.12	-0.01	0.020	1722	-0.03	-0.09	0.02	0.214	1722	-0.07	-0.14	0.00	0.064	1722	-0.01	-0.08	0.06	0.717
Triglycerides to total lipids ratio in very small VLDL (%)	1722	0.00	-0.05	0.06	0.865	1722	0.00	-0.06	0.06	0.961	1722	0.00	-0.07	0.08	0.924	1722	0.00	-0.08	0.08	0.984
Phospholipids to total lipds ratio in IDL (%)	1722	-0.05	-0.11	0.01	0.085	1722	-0.02	-0.08	0.03	0.427	1722	-0.15	-0.22	-0.08	<0.0001	1722	-0.10	-0.17	-0.02	0.009
Total cholesterol to total lipids ratio in IDL (%)	1722	0.03	-0.02	0.09	0.220	1722	0.01	-0.04	0.07	0.689	1722	0.11	0.03	0.18	0.004	1722	0.07	-0.01	0.14	0.075
Cholesterol esters to total lipids ratio in IDL (%)	1722	0.05	0.00	0.10	0.054	1722	0.02	-0.03	0.07	0.467	1722	0.16	0.09	0.22	<0.0001	1722	0.10	0.03	0.17	0.005
Free cholesterol to total lipids ratio in IDL (%)	1722	-0.04	-0.10	0.02	0.178	1722	-0.02	-0.08	0.04	0.551	1722	-0.10	-0.18	-0.03	0.008	1722	-0.07	-0.14	0.01	0.085
Triglycerides to total lipids ratio in IDL (%)	1722	-0.02	-0.07	0.03	0.472	1722	0.00	-0.06	0.05	0.880	1722	-0.06	-0.14	0.01	0.095	1722	-0.04	-0.12	0.04	0.311
Phospholipids to total lipds ratio in large LDL (%)	1722	-0.01	-0.07	0.04	0.628	1722	0.00	-0.06	0.06	0.925	1722	-0.01	-0.09	0.06	0.725	1722	0.01	-0.07	0.08	0.866
Total cholesterol to total lipids ratio in large LDL (%)	1722	0.03	-0.03	0.08	0.360	1722	0.00	-0.06	0.06	0.982	1722	0.06	-0.02	0.14	0.116	1722	0.03	-0.05	0.11	0.498
Cholesterol esters to total lipids ratio in large LDL (%)	1722	0.04	-0.02	0.09	0.202	1722	0.00	-0.06	0.06	0.968	1722	0.09	0.01	0.17	0.031	1722	0.04	-0.04	0.12	0.350
Free cholesterol to total lipids ratio in large LDL (%)	1722	-0.04	-0.10	0.02	0.149	1722	0.00	-0.06	0.06	0.994	1722	-0.10	-0.17	-0.02	0.009	1722	-0.04	-0.11	0.03	0.238
Triglycerides to total lipids ratio in large LDL (%)	1722	-0.03	-0.08	0.03	0.330	1722	-0.01	-0.06	0.05	0.856	1722	-0.09	-0.17	-0.01	0.024	1722	-0.05	-0.13	0.02	0.180
Phospholipids to total lipds ratio in medium LDL (%)	1722	-0.03	-0.08	0.03	0.336	1722	-0.01	-0.07	0.05	0.682	1722	-0.03	-0.11	0.04	0.379	1722	-0.02	-0.09	0.06	0.680
Total cholesterol to total lipids ratio in medium LDL (%)	1722	0.04	-0.01	0.10	0.147	1722	0.02	-0.04	0.08	0.480	1722	0.08	0.00	0.16	0.052	1722	0.05	-0.03	0.13	0.222
Cholesterol esters to total lipids ratio in medium LDL (%)	1722	0.04	-0.01	0.10	0.148	1722	0.02	-0.04	0.08	0.596	1722	0.08	0.00	0.15	0.057	1722	0.04	-0.04	0.12	0.300
Free cholesterol to total lipids ratio in medium LDL (%)	1722	-0.04	-0.09	0.02	0.192	1722	-0.01	-0.07	0.05	0.782	1722	-0.06	-0.13	0.01	0.104	1722	-0.02	-0.10	0.05	0.518
Triglycerides to total lipids ratio in medium LDL (%)	1722	-0.03	-0.08	0.02	0.232	1722	-0.01	-0.06	0.03	0.561	1722	-0.10	-0.17	-0.03	0.003	1722	-0.07	-0.14	-0.01	0.033
Phospholipids to total lipds ratio in small LDL (%)	1722	-0.03	-0.08	0.03	0.307	1722	-0.01	-0.06	0.05	0.807	1722	-0.04	-0.12	0.03	0.290	1722	-0.01	-0.09	0.06	0.749
Total cholesterol to total lipids ratio in small LDL (%)	1722	0.03	-0.03	0.08	0.376	1722	0.01	-0.05	0.07	0.795	1722	0.04	-0.04	0.12	0.304	1722	0.02	-0.06	0.10	0.674
Cholesterol esters to total lipids ratio in small LDL (%)	1722	0.03	-0.02	0.09	0.272	1722	0.01	-0.05	0.07	0.802	1722	0.05	-0.03	0.13	0.201	1722	0.02	-0.06	0.10	0.634
Free cholesterol to total lipids ratio in small LDL (%)	1722	-0.04	-0.09	0.02	0.176	1722	-0.01	-0.07	0.05	0.771	1722	-0.06	-0.13	0.02	0.126	1722	-0.02	-0.09	0.05	0.605
Triglycerides to total lipids ratio in small LDL (%)	1722	0.00	-0.05	0.06	0.886	1722	0.00	-0.06	0.05	0.927	1722	-0.01	-0.09	0.06	0.741	1722	-0.02	-0.10	0.06	0.610
Phospholipids to total lipds ratio in very large HDL (%)	1722	-0.06	-0.11	-0.01	0.030	1722	-0.01	-0.07	0.04	0.607	1722	-0.15	-0.22	-0.08	<0.0001	1722	-0.10	-0.16	-0.03	0.005
Total cholesterol to total lipids ratio in very large HDL (%)	1722	0.04	-0.01	0.09	0.121	1722	0.00	-0.05	0.05	0.936	1722	0.12	0.06	0.19	0.0003	1722	0.07	0.01	0.14	0.031
Cholesterol esters to total lipids ratio in very large HDL (%)	1722	0.04	-0.01	0.09	0.098	1722	0.00	-0.05	0.05	0.910	1722	0.13	0.06	0.19	0.0002	1722	0.08	0.01	0.14	0.021
Free cholesterol to total lipids ratio in very large HDL (%)	1722	-0.04	-0.10	0.01	0.134	1722	-0.05	-0.11	0.01	0.098	1722	-0.08	-0.16	0.00	0.038	1722	-0.08	-0.16	0.00	0.042
Triglycerides to total lipids ratio in very large HDL (%)	1722	0.10	0.04	0.17	0.002	1722	0.08	0.02	0.14	0.012	1722	0.19	0.09	0.28	0.0001	1722	0.14	0.05	0.23	0.002
Phospholipids to total lipds ratio in large HDL (%)	1722	0.07	0.01	0.13	0.032	1722	0.01	-0.05	0.07	0.788	1722	0.15	0.06	0.23	0.001	1722	0.07	-0.01	0.14	0.084
Total cholesterol to total lipids ratio in large HDL (%)	1722	-0.10	-0.16	-0.03	0.003	1722	-0.04	-0.10	0.02	0.227	1722	-0.19	-0.27	-0.10	<0.0001	1722	-0.11	-0.18	-0.03	0.006
Cholesterol esters to total lipids ratio in large HDL (%)	1722	-0.10	-0.16	-0.03	0.004	1722	-0.04	-0.10	0.03	0.240	1722	-0.19	-0.28	-0.10	<0.0001	1722	-0.10	-0.18	-0.03	0.008
Free cholesterol to total lipids ratio in large HDL (%)	1722	-0.08	-0.13	-0.02	0.009	1722	-0.04	-0.10	0.02	0.195	1722	-0.15	-0.23	-0.07	0.0003	1722	-0.09	-0.17	-0.02	0.017

Online Table 5 Associations of lean mass index at age 10y and 18y with cardiometabolic traits at age 18y in ALSPAC

	**At age 10y**										**At age 18y**									
	**Lean mass index (per 1.0 kg/m^2 higher)**										**Lean mass index (per 2.2 kg/m^2 higher)**									
	Adj. for age, sex, ethnicity, maternal education					*Additionally adj. for fat mass index at 10y*					*Adj. for age, sex, ethnicity, maternal education, smoking, alcohol, puberty timing*					*Additionally adj. for fat mass index at 18y*				
**Standardized outcome at age 18y**	**N**	**Beta**	**LCL**	**UCL**	**P-value**	**N**	**Beta**	**LCL**	**UCL**	**P-value**	**N**	**Beta**	**LCL**	**UCL**	**P-value**	**N**	**Beta**	**LCL**	**UCL**	**P-value**
Triglycerides to total lipids ratio in large HDL (%)	1722	0.12	0.06	0.18	0.0001	1722	0.08	0.03	0.14	0.005	1722	0.22	0.14	0.31	<0.0001	1722	0.15	0.08	0.23	<0.0001
Phospholipids to total lipds ratio in medium HDL (%)	1722	-0.05	-0.10	0.01	0.105	1722	-0.04	-0.10	0.02	0.176	1722	-0.10	-0.18	-0.01	0.030	1722	-0.08	-0.17	0.01	0.069
Total cholesterol to total lipids ratio in medium HDL (%)	1722	-0.02	-0.07	0.04	0.592	1722	0.00	-0.06	0.06	0.956	1722	-0.02	-0.11	0.07	0.672	1722	0.00	-0.08	0.09	0.952
Cholesterol esters to total lipids ratio in medium HDL (%)	1722	-0.02	-0.08	0.04	0.482	1722	0.00	-0.07	0.06	0.878	1722	-0.03	-0.12	0.05	0.478	1722	0.00	-0.09	0.08	0.907
Free cholesterol to total lipids ratio in medium HDL (%)	1722	0.01	-0.04	0.06	0.611	1722	0.01	-0.05	0.06	0.854	1722	0.04	-0.04	0.12	0.289	1722	0.03	-0.05	0.10	0.511
Triglycerides to total lipids ratio in medium HDL (%)	1722	0.11	0.05	0.17	0.001	1722	0.07	0.01	0.14	0.026	1722	0.20	0.11	0.29	<0.0001	1722	0.13	0.05	0.21	0.002
Phospholipids to total lipds ratio in small HDL (%)	1722	0.05	0.00	0.10	0.059	1722	0.04	-0.01	0.09	0.137	1722	0.09	0.02	0.16	0.010	1722	0.09	0.02	0.16	0.015
Total cholesterol to total lipids ratio in small HDL (%)	1722	-0.07	-0.12	-0.02	0.004	1722	-0.06	-0.11	-0.01	0.023	1722	-0.13	-0.20	-0.06	0.0003	1722	-0.11	-0.18	-0.04	0.001
Cholesterol esters to total lipids ratio in small HDL (%)	1722	-0.06	-0.11	-0.01	0.017	1722	-0.05	-0.10	0.00	0.045	1722	-0.11	-0.18	-0.04	0.002	1722	-0.10	-0.17	-0.03	0.004
Free cholesterol to total lipids ratio in small HDL (%)	1722	-0.05	-0.11	0.01	0.095	1722	-0.02	-0.08	0.04	0.591	1722	-0.07	-0.15	0.01	0.103	1722	0.00	-0.09	0.08	0.922
Triglycerides to total lipids ratio in small HDL (%)	1722	0.09	0.03	0.16	0.005	1722	0.07	0.01	0.14	0.034	1722	0.14	0.05	0.23	0.002	1722	0.09	0.01	0.18	0.033
Mean diameter for VLDL particles (nm)	1722	0.07	0.01	0.12	0.023	1722	0.02	-0.04	0.08	0.545	1722	0.15	0.08	0.23	0.0001	1722	0.09	0.01	0.16	0.023
Mean diameter for LDL particles (nm)	1722	0.04	-0.01	0.10	0.117	1722	0.10	0.04	0.15	0.001	1722	0.01	-0.06	0.09	0.733	1722	0.05	-0.03	0.13	0.192
Mean diameter for HDL particles (nm)	1722	-0.06	-0.11	-0.01	0.029	1722	-0.02	-0.08	0.03	0.418	1722	-0.15	-0.23	-0.07	0.0001	1722	-0.09	-0.17	-0.02	0.014
Serum total cholesterol (mmol/l)	1722	0.01	-0.05	0.06	0.846	1722	-0.01	-0.07	0.05	0.631	1722	0.01	-0.06	0.08	0.782	1722	-0.02	-0.10	0.05	0.564
Total cholesterol in VLDL (mmol/l)	1722	0.07	0.01	0.13	0.030	1722	0.03	-0.04	0.09	0.449	1722	0.16	0.08	0.24	0.0002	1722	0.08	0.00	0.16	0.043
Remnant cholesterol (non-HDL, non-LDL -cholesterol) (mmol/l)	1722	0.05	-0.01	0.12	0.088	1722	0.02	-0.05	0.08	0.592	1722	0.12	0.04	0.20	0.004	1722	0.05	-0.03	0.13	0.213
Total cholesterol in LDL (mmol/l)	1722	0.02	-0.04	0.07	0.566	1722	-0.01	-0.07	0.06	0.870	1722	0.03	-0.05	0.10	0.513	1722	-0.01	-0.09	0.06	0.730
Total cholesterol in HDL (mmol/l)	1722	-0.07	-0.13	-0.02	0.005	1722	-0.06	-0.11	0.00	0.043	1722	-0.15	-0.22	-0.08	<0.0001	1722	-0.10	-0.17	-0.03	0.003
Total cholesterol in HDL2 (mmol/l)	1722	-0.08	-0.14	-0.03	0.003	1722	-0.06	-0.11	0.00	0.035	1722	-0.17	-0.24	-0.09	<0.0001	1722	-0.12	-0.19	-0.04	0.001
Total cholesterol in HDL3 (mmol/l)	1722	-0.06	-0.11	0.00	0.032	1722	-0.05	-0.10	0.01	0.084	1722	-0.11	-0.18	-0.04	0.002	1722	-0.08	-0.15	-0.01	0.022
Esterified cholesterol (mmol/l)	1722	0.00	-0.05	0.06	0.922	1722	-0.02	-0.08	0.04	0.600	1722	0.01	-0.06	0.09	0.744	1722	-0.02	-0.10	0.06	0.624
Free cholesterol (mmol/l)	1722	0.01	-0.04	0.07	0.687	1722	-0.01	-0.07	0.05	0.733	1722	0.00	-0.07	0.08	0.901	1722	-0.03	-0.10	0.05	0.478
Serum total triglycerides (mmol/l)	1722	0.07	0.01	0.13	0.034	1722	0.02	-0.04	0.09	0.477	1722	0.14	0.06	0.22	0.001	1722	0.07	0.00	0.15	0.056
Triglycerides in VLDL (mmol/l)	1722	0.07	0.01	0.13	0.022	1722	0.02	-0.04	0.08	0.484	1722	0.16	0.08	0.25	<0.0001	1722	0.09	0.01	0.16	0.020
Triglycerides in LDL (mmol/l)	1722	0.01	-0.05	0.06	0.820	1722	0.00	-0.06	0.06	0.959	1722	-0.02	-0.09	0.04	0.477	1722	-0.03	-0.10	0.04	0.340
Triglycerides in HDL (mmol/l)	1722	0.06	0.01	0.12	0.029	1722	0.04	-0.02	0.11	0.149	1722	0.11	0.04	0.19	0.003	1722	0.08	0.00	0.15	0.038
Diacylglycerol (mmol/l)	1722	0.08	0.02	0.14	0.010	1722	0.05	-0.01	0.11	0.122	1722	0.16	0.08	0.24	<0.0001	1722	0.11	0.03	0.19	0.005
Ratio of diacylglycerol to triglycerides	1722	0.04	-0.01	0.10	0.111	1722	0.02	-0.03	0.08	0.422	1722	0.10	0.03	0.17	0.006	1722	0.08	0.01	0.16	0.034
Total phosphoglycerides (mmol/l)	1722	0.01	-0.05	0.06	0.851	1722	0.00	-0.06	0.06	0.946	1722	0.00	-0.07	0.07	0.932	1722	0.00	-0.07	0.07	0.932
Ratio of triglycerides to phosphoglycerides	1722	0.08	0.02	0.15	0.007	1722	0.04	-0.02	0.10	0.171	1722	0.17	0.09	0.25	<0.0001	1722	0.10	0.02	0.17	0.009
Phosphatidylcholine and other cholines (mmol/l)	1722	0.00	-0.05	0.05	0.953	1722	0.00	-0.05	0.06	0.939	1722	0.00	-0.06	0.07	0.922	1722	0.01	-0.06	0.08	0.828
Total cholines (mmol/l)	1722	-0.01	-0.06	0.05	0.837	1722	-0.01	-0.06	0.05	0.846	1722	-0.01	-0.08	0.06	0.795	1722	-0.01	-0.08	0.06	0.813
Apolipoprotein A-I (g/l)	1722	-0.05	-0.10	0.00	0.047	1722	-0.05	-0.10	0.01	0.078	1722	-0.10	-0.17	-0.03	0.005	1722	-0.08	-0.15	-0.01	0.022
Apolipoprotein B (g/l)	1722	0.06	0.00	0.12	0.064	1722	0.02	-0.04	0.09	0.539	1722	0.12	0.04	0.20	0.004	1722	0.05	-0.03	0.13	0.206
Ratio of apolipoprotein B to apolipoprotein A-I	1722	0.09	0.03	0.15	0.006	1722	0.05	-0.02	0.11	0.159	1722	0.17	0.09	0.26	<0.0001	1722	0.09	0.01	0.17	0.022
Total fatty acids (mmol/l)	1722	0.03	-0.03	0.09	0.299	1722	0.00	-0.06	0.07	0.903	1722	0.06	-0.01	0.14	0.093	1722	0.02	-0.05	0.09	0.584
Estimated description of fatty acid chain length, not actual carbon number	1722	0.01	-0.04	0.06	0.793	1722	0.00	-0.05	0.06	0.903	1722	-0.03	-0.11	0.04	0.366	1722	-0.04	-0.11	0.04	0.310
Estimated degree of unsaturation	1722	-0.03	-0.08	0.03	0.307	1722	-0.02	-0.08	0.04	0.476	1722	-0.06	-0.14	0.02	0.123	1722	-0.05	-0.12	0.03	0.233
22:6, docosahexaenoic acid (mmol/l)	1722	0.02	-0.03	0.08	0.470	1722	0.02	-0.04	0.08	0.513	1722	0.02	-0.05	0.09	0.559	1722	0.01	-0.06	0.08	0.782
18:2, linoleic acid (mmol/l)	1722	0.00	-0.05	0.06	0.998	1722	-0.01	-0.07	0.05	0.693	1722	0.04	-0.03	0.11	0.305	1722	0.02	-0.05	0.09	0.657
Conjugated linoleic acid (mmol/l)	1722	0.02	-0.03	0.06	0.542	1722	-0.01	-0.06	0.04	0.750	1722	0.00	-0.07	0.07	0.972	1722	-0.03	-0.10	0.04	0.386
Omega-3 fatty acids (mmol/l)	1722	0.01	-0.05	0.07	0.733	1722	-0.01	-0.07	0.05	0.833	1722	0.06	-0.02	0.13	0.152	1722	0.03	-0.05	0.11	0.489
Omega-6 fatty acids (mmol/l)	1722	0.01	-0.05	0.06	0.792	1722	-0.01	-0.07	0.05	0.773	1722	0.04	-0.03	0.11	0.313	1722	0.01	-0.06	0.08	0.798
Polyunsaturated fatty acids (mmol/l)	1722	0.01	-0.05	0.06	0.775	1722	-0.01	-0.07	0.05	0.772	1722	0.04	-0.03	0.11	0.265	1722	0.01	-0.06	0.08	0.740
Monounsaturated fatty acids; 16:1, 18:1 (mmol/l)	1722	0.04	-0.02	0.10	0.181	1722	0.01	-0.06	0.07	0.844	1722	0.04	-0.03	0.12	0.288	1722	-0.01	-0.09	0.06	0.697
Saturated fatty acids (mmol/l)	1722	0.04	-0.02	0.10	0.235	1722	0.01	-0.05	0.07	0.711	1722	0.09	0.01	0.17	0.022	1722	0.05	-0.02	0.13	0.164
Ratio of 22:6 docosahexaenoic acid to total fatty acids (%)	1722	0.01	-0.05	0.06	0.762	1722	0.02	-0.04	0.08	0.511	1722	-0.01	-0.08	0.07	0.852	1722	0.00	-0.07	0.08	0.936
Ratio of 18:2 linoleic acid to total fatty acids (%)	1722	-0.05	-0.11	0.00	0.073	1722	-0.02	-0.08	0.04	0.545	1722	-0.04	-0.12	0.03	0.284	1722	0.00	-0.07	0.08	0.978
Ratio of conjugated linoleic acid to total fatty acids (%)	1722	0.01	-0.04	0.05	0.687	1722	-0.01	-0.06	0.04	0.665	1722	0.00	-0.06	0.06	0.984	1722	-0.03	-0.09	0.04	0.446
Ratio of omega-3 fatty acids to total fatty acids (%)	1722	-0.02	-0.07	0.04	0.567	1722	-0.01	-0.08	0.05	0.636	1722	0.02	-0.06	0.10	0.599	1722	0.02	-0.06	0.10	0.581
Ratio of omega-6 fatty acids to total fatty acids (%)	1722	-0.05	-0.10	0.01	0.096	1722	-0.02	-0.08	0.04	0.573	1722	-0.05	-0.13	0.03	0.196	1722	-0.01	-0.09	0.07	0.755
Ratio of polyunsaturated fatty acids to total fatty acids (%)	1722	-0.05	-0.11	0.01	0.091	1722	-0.02	-0.08	0.04	0.522	1722	-0.04	-0.12	0.03	0.268	1722	-0.01	-0.08	0.07	0.870
Ratio of monounsaturated fatty acids to total fatty acids (%)	1722	0.03	-0.02	0.09	0.278	1722	0.00	-0.06	0.06	0.920	1722	-0.03	-0.11	0.04	0.401	1722	-0.08	-0.16	-0.01	0.033
Ratio of saturated fatty acids to total fatty acids (%)	1722	0.02	-0.03	0.07	0.492	1722	0.03	-0.03	0.08	0.303	1722	0.10	0.02	0.18	0.012	1722	0.12	0.04	0.20	0.002
Insulin (mu/l)	1722	0.09	0.03	0.14	0.003	1722	0.04	-0.01	0.09	0.155	1722	0.19	0.07	0.32	0.002	1722	0.12	0.03	0.21	0.009
Glucose (mmol/l)	1722	0.02	-0.02	0.06	0.427	1722	0.01	-0.04	0.05	0.791	1722	0.05	-0.02	0.12	0.159	1722	0.02	-0.04	0.09	0.480
Lactate (mmol/l)	1722	-0.04	-0.09	0.01	0.108	1722	-0.06	-0.12	-0.01	0.021	1722	-0.07	-0.14	0.00	0.051	1722	-0.08	-0.16	-0.01	0.027

Online Table 5 Associations of lean mass index at age 10y and 18y with cardiometabolic traits at age 18y in ALSPAC

	At age 10y										At age 18y									
	Lean mass index (per 1.0 kg/m^2 higher)										Lean mass index (per 2.2 kg/m^2 higher)									
	Adj. for age, sex, ethnicity, maternal education					*Additionally adj. for fat mass index at 10y*					*Adj. for age, sex, ethnicity, maternal education, smoking, alcohol, puberty timing*					*Additionally adj. for fat mass index at 18y*				
**Standardized outcome at age 18y**	**N**	**Beta**	**LCL**	**UCL**	**P-value**	**N**	**Beta**	**LCL**	**UCL**	**P-value**	**N**	**Beta**	**LCL**	**UCL**	**P-value**	**N**	**Beta**	**LCL**	**UCL**	**P-value**
Pyruvate (mmol/l)	1722	0.03	-0.03	0.08	0.341	1722	-0.01	-0.07	0.04	0.650	1722	0.00	-0.08	0.07	0.901	1722	-0.04	-0.12	0.04	0.328
Citrate (mmol/l)	1722	-0.06	-0.12	-0.01	0.029	1722	-0.02	-0.08	0.04	0.472	1722	-0.10	-0.18	-0.02	0.014	1722	-0.07	-0.15	0.01	0.090
Alanine (mmol/l)	1722	0.03	-0.02	0.09	0.258	1722	0.02	-0.05	0.08	0.600	1722	0.09	0.01	0.17	0.028	1722	0.07	-0.01	0.15	0.102
Glutamine (mmol/l)	1722	-0.02	-0.07	0.03	0.429	1722	-0.01	-0.06	0.05	0.756	1722	-0.04	-0.11	0.02	0.209	1722	-0.04	-0.11	0.03	0.264
Histidine (mmol/l)	1722	0.02	-0.04	0.07	0.604	1722	0.01	-0.05	0.07	0.748	1722	0.05	-0.03	0.13	0.202	1722	0.05	-0.04	0.13	0.282
Isoleucine (mmol/l)	1722	0.08	0.03	0.14	0.001	1722	0.05	0.00	0.11	0.050	1722	0.19	0.12	0.26	<0.0001	1722	0.15	0.08	0.21	<0.0001
Leucine (mmol/l)	1722	0.09	0.04	0.13	0.0001	1722	0.07	0.02	0.12	0.003	1722	0.22	0.16	0.27	<0.0001	1722	0.19	0.13	0.25	<0.0001
Valine (mmol/l)	1722	0.11	0.06	0.16	<0.0001	1722	0.07	0.02	0.13	0.006	1722	0.22	0.15	0.30	<0.0001	1722	0.18	0.11	0.25	<0.0001
Phenylalanine (mmol/l)	1722	0.08	0.02	0.13	0.006	1722	0.04	-0.01	0.10	0.132	1722	0.18	0.10	0.25	<0.0001	1722	0.14	0.06	0.21	0.0004
Tyrosine (mmol/l)	1722	0.06	0.01	0.12	0.022	1722	0.02	-0.04	0.07	0.518	1722	0.14	0.06	0.22	0.0003	1722	0.08	0.01	0.15	0.034
Acetate (mmol/l)	1722	-0.05	-0.08	-0.02	0.001	1722	-0.04	-0.06	-0.01	0.010	1722	-0.06	-0.11	-0.01	0.017	1722	-0.05	-0.10	0.00	0.040
Acetoacetate (mmol/l)	1722	-0.07	-0.12	-0.02	0.004	1722	-0.08	-0.14	-0.03	0.002	1722	-0.12	-0.19	-0.05	0.001	1722	-0.11	-0.18	-0.04	0.002
3-hydroxybutyrate (mmol/l)	1722	-0.08	-0.13	-0.03	0.002	1722	-0.09	-0.15	-0.03	0.002	1722	-0.16	-0.24	-0.08	<0.0001	1722	-0.14	-0.22	-0.07	0.0003
Creatinine (mmol/l)	1722	0.14	0.09	0.19	<0.0001	1722	0.15	0.10	0.20	<0.0001	1722	0.29	0.23	0.36	<0.0001	1722	0.30	0.24	0.37	<0.0001
Albumin (signal area)	1722	0.02	-0.03	0.07	0.406	1722	0.05	0.00	0.11	0.073	1722	-0.01	-0.09	0.07	0.798	1722	0.01	-0.07	0.09	0.839
Glycoprotein acetyls, mainly a1-acid glycoprotein (mmol/l)	1722	0.06	0.00	0.12	0.039	1722	0.01	-0.05	0.06	0.834	1722	0.09	0.02	0.17	0.015	1722	0.02	-0.05	0.09	0.611
C-reactive protein (mg/l)	1722	0.02	0.00	0.05	0.087	1722	0.01	-0.02	0.03	0.692	1722	0.01	-0.05	0.06	0.758	1722	-0.01	-0.07	0.04	0.675

Online Table 6 Associations of change in body mass index (BMI) and fat mass index from age 10y-18y with cardiometabolic traits at age 18y in ALSPAC

Change from age 10-18y

	BMI (per SD-unit gain) *Adj. for age, sex, ethnicity, maternal education, BMI at 10y*					Fat mass index (per SD-unit gain) *Adj. for age, sex, ethnicity, maternal education, fat mass index at 10y*				
**Standardized outcome at age 18y**	**N**	**Beta**	**LCL**	**UCL**	**P-value**	**N**	**Beta**	**LCL**	**UCL**	**P-value**
Systolic blood pressure (mmHg)	3506	0.29	0.24	0.35	<0.0001	3409	0.29	0.25	0.34	<0.0001
Diastolic blood pressure (mmHg)	3506	0.29	0.22	0.35	<0.0001	3409	0.39	0.34	0.44	<0.0001
Concentration of chylomicrons and extremely large VLDL particles (mol/l)	2398	0.47	0.36	0.57	<0.0001	2342	0.53	0.44	0.61	<0.0001
Total lipids in chylomicrons and extremely large VLDL (mmol/l)	2398	0.46	0.36	0.57	<0.0001	2342	0.52	0.44	0.60	<0.0001
Phospholipids in chylomicrons and extremely large VLDL (mmol/l)	2398	0.46	0.35	0.56	<0.0001	2342	0.51	0.43	0.59	<0.0001
Total cholesterol in chylomicrons and extremely large VLDL (mmol/l)	2398	0.46	0.36	0.57	<0.0001	2342	0.51	0.43	0.60	<0.0001
Cholesterol esters in chylomicrons and extremely large VLDL (mmol/l)	2398	0.46	0.36	0.56	<0.0001	2342	0.51	0.42	0.59	<0.0001
Free cholesterol in chylomicrons and extremely large VLDL (mmol/l)	2398	0.45	0.35	0.56	<0.0001	2342	0.51	0.42	0.59	<0.0001
Triglycerides in chylomicrons and extremely large VLDL (mmol/l)	2398	0.46	0.36	0.57	<0.0001	2342	0.52	0.43	0.60	<0.0001
Concentration of very large VLDL particles (mol/l)	2398	0.46	0.35	0.56	<0.0001	2342	0.51	0.42	0.59	<0.0001
Total lipids in very large VLDL (mmol/l)	2398	0.46	0.35	0.56	<0.0001	2342	0.50	0.42	0.59	<0.0001
Phospholipids in very large VLDL (mmol/l)	2398	0.45	0.35	0.56	<0.0001	2342	0.50	0.42	0.59	<0.0001
Total cholesterol in very large VLDL (mmol/l)	2398	0.47	0.37	0.58	<0.0001	2342	0.53	0.44	0.61	<0.0001
Cholesterol esters in very large VLDL (mmol/l)	2398	0.48	0.37	0.58	<0.0001	2342	0.53	0.45	0.61	<0.0001
Free cholesterol in very large VLDL (mmol/l)	2398	0.46	0.36	0.57	<0.0001	2342	0.52	0.43	0.60	<0.0001
Triglycerides in very large VLDL (mmol/l)	2398	0.45	0.34	0.55	<0.0001	2342	0.49	0.41	0.58	<0.0001
Concentration of large VLDL particles (mol/l)	2398	0.45	0.34	0.55	<0.0001	2342	0.49	0.41	0.57	<0.0001
Total lipids in large VLDL (mmol/l)	2398	0.45	0.35	0.55	<0.0001	2342	0.49	0.41	0.57	<0.0001
Phospholipids in large VLDL (mmol/l)	2398	0.45	0.34	0.55	<0.0001	2342	0.49	0.41	0.57	<0.0001
Total cholesterol in large VLDL (mmol/l)	2398	0.46	0.36	0.56	<0.0001	2342	0.50	0.42	0.59	<0.0001
Cholesterol esters in large VLDL (mmol/l)	2398	0.47	0.36	0.57	<0.0001	2342	0.51	0.43	0.59	<0.0001
Free cholesterol in large VLDL (mmol/l)	2398	0.45	0.34	0.55	<0.0001	2342	0.49	0.41	0.57	<0.0001
Triglycerides in large VLDL (mmol/l)	2398	0.45	0.34	0.55	<0.0001	2342	0.48	0.40	0.57	<0.0001
Concentration of medium VLDL particles (mol/l)	2398	0.46	0.35	0.56	<0.0001	2342	0.50	0.42	0.58	<0.0001
Total lipids in medium VLDL (mmol/l)	2398	0.46	0.36	0.56	<0.0001	2342	0.50	0.42	0.59	<0.0001
Phospholipids in medium VLDL (mmol/l)	2398	0.45	0.35	0.55	<0.0001	2342	0.49	0.41	0.57	<0.0001
Total cholesterol in medium VLDL (mmol/l)	2398	0.45	0.35	0.55	<0.0001	2342	0.49	0.41	0.57	<0.0001
Cholesterol esters in medium VLDL (mmol/l)	2398	0.44	0.34	0.54	<0.0001	2342	0.49	0.41	0.57	<0.0001
Free cholesterol in medium VLDL (mmol/l)	2398	0.44	0.34	0.54	<0.0001	2342	0.48	0.40	0.56	<0.0001
Triglycerides in medium VLDL (mmol/l)	2398	0.46	0.35	0.56	<0.0001	2342	0.50	0.42	0.58	<0.0001
Concentration of small VLDL particles (mol/l)	2398	0.42	0.33	0.52	<0.0001	2342	0.47	0.39	0.55	<0.0001
Total lipids in small VLDL (mmol/l)	2398	0.43	0.33	0.52	<0.0001	2342	0.48	0.40	0.56	<0.0001
Phospholipids in small VLDL (mmol/l)	2398	0.40	0.30	0.49	<0.0001	2342	0.44	0.36	0.52	<0.0001
Total cholesterol in small VLDL (mmol/l)	2398	0.40	0.31	0.49	<0.0001	2342	0.46	0.39	0.54	<0.0001
Cholesterol esters in small VLDL (mmol/l)	2398	0.38	0.30	0.47	<0.0001	2342	0.45	0.38	0.53	<0.0001
Free cholesterol in small VLDL (mmol/l)	2398	0.38	0.29	0.47	<0.0001	2342	0.43	0.35	0.50	<0.0001
Triglycerides in small VLDL (mmol/l)	2398	0.41	0.32	0.50	<0.0001	2342	0.45	0.37	0.53	<0.0001
Concentration of very small VLDL particles (mol/l)	2398	0.24	0.17	0.30	<0.0001	2342	0.27	0.20	0.35	<0.0001
Total lipids in very small VLDL (mmol/l)	2398	0.27	0.20	0.34	<0.0001	2342	0.32	0.24	0.39	<0.0001
Phospholipids in very small VLDL (mmol/l)	2398	0.19	0.13	0.25	<0.0001	2342	0.23	0.15	0.30	<0.0001
Total cholesterol in very small VLDL (mmol/l)	2398	0.25	0.17	0.32	<0.0001	2342	0.30	0.22	0.37	<0.0001
Cholesterol esters in very small VLDL (mmol/l)	2398	0.28	0.20	0.35	<0.0001	2342	0.34	0.26	0.41	<0.0001
Free cholesterol in very small VLDL (mmol/l)	2398	0.15	0.08	0.22	<0.0001	2342	0.17	0.10	0.25	<0.0001
Triglycerides in very small VLDL (mmol/l)	2398	0.28	0.21	0.35	<0.0001	2342	0.30	0.23	0.38	<0.0001
Concentration of IDL particles (mol/l)	2398	0.14	0.08	0.20	<0.0001	2342	0.17	0.10	0.24	<0.0001
Total lipids in IDL (mmol/l)	2398	0.15	0.09	0.22	<0.0001	2342	0.19	0.12	0.27	<0.0001
Phospholipids in IDL (mmol/l)	2398	0.11	0.05	0.17	0.0002	2342	0.15	0.08	0.22	<0.0001
Total cholesterol in IDL (mmol/l)	2398	0.17	0.11	0.24	<0.0001	2342	0.22	0.14	0.29	<0.0001
Cholesterol esters in IDL (mmol/l)	2398	0.21	0.14	0.28	<0.0001	2342	0.26	0.18	0.34	<0.0001
Free cholesterol in IDL (mmol/l)	2398	0.08	0.02	0.14	0.010	2342	0.11	0.04	0.18	0.003
Triglycerides in IDL (mmol/l)	2398	0.06	0.01	0.12	0.026	2342	0.06	0.00	0.13	0.064
Concentration of large LDL particles (mol/l)	2398	0.15	0.08	0.21	<0.0001	2342	0.18	0.11	0.25	<0.0001
Total lipids in large LDL (mmol/l)	2398	0.15	0.09	0.21	<0.0001	2342	0.19	0.12	0.26	<0.0001
Phospholipids in large LDL (mmol/l)	2398	0.18	0.11	0.24	<0.0001	2342	0.22	0.15	0.29	<0.0001
Total cholesterol in large LDL (mmol/l)	2398	0.16	0.09	0.22	<0.0001	2342	0.20	0.13	0.27	<0.0001
Cholesterol esters in large LDL (mmol/l)	2398	0.17	0.11	0.24	<0.0001	2342	0.22	0.15	0.29	<0.0001
Free cholesterol in large LDL (mmol/l)	2398	0.10	0.04	0.16	0.001	2342	0.14	0.07	0.21	0.0002
Triglycerides in large LDL (mmol/l)	2398	0.02	-0.03	0.08	0.455	2342	0.02	-0.05	0.08	0.612
Concentration of medium LDL particles (mol/l)	2398	0.17	0.11	0.24	<0.0001	2342	0.22	0.14	0.29	<0.0001
Total lipids in medium LDL (mmol/l)	2398	0.17	0.11	0.23	<0.0001	2342	0.21	0.14	0.28	<0.0001
Phospholipids in medium LDL (mmol/l)	2398	0.22	0.16	0.29	<0.0001	2342	0.27	0.20	0.34	<0.0001
Total cholesterol in medium LDL (mmol/l)	2398	0.17	0.10	0.23	<0.0001	2342	0.21	0.14	0.29	<0.0001
Cholesterol esters in medium LDL (mmol/l)	2398	0.17	0.11	0.24	<0.0001	2342	0.22	0.14	0.29	<0.0001
Free cholesterol in medium LDL (mmol/l)	2398	0.15	0.09	0.21	<0.0001	2342	0.19	0.12	0.27	<0.0001
Triglycerides in medium LDL (mmol/l)	2398	0.01	-0.04	0.07	0.599	2342	0.02	-0.05	0.08	0.600
Concentration of small LDL particles (mol/l)	2398	0.17	0.11	0.24	<0.0001	2342	0.21	0.14	0.28	<0.0001
Total lipids in small LDL (mmol/l)	2398	0.18	0.11	0.24	<0.0001	2342	0.22	0.15	0.29	<0.0001
Phospholipids in small LDL (mmol/l)	2398	0.20	0.14	0.27	<0.0001	2342	0.25	0.17	0.32	<0.0001
Total cholesterol in small LDL (mmol/l)	2398	0.16	0.10	0.23	<0.0001	2342	0.21	0.14	0.28	<0.0001
Cholesterol esters in small LDL (mmol/l)	2398	0.16	0.10	0.23	<0.0001	2342	0.21	0.14	0.28	<0.0001
Free cholesterol in small LDL (mmol/l)	2398	0.16	0.09	0.22	<0.0001	2342	0.20	0.12	0.27	<0.0001
Triglycerides in small LDL (mmol/l)	2398	0.16	0.10	0.22	<0.0001	2342	0.18	0.11	0.25	<0.0001
Concentration of very large HDL particles (mol/l)	2398	-0.30	-0.38	-0.21	<0.0001	2342	-0.34	-0.40	-0.27	<0.0001
Total lipids in very large HDL (mmol/l)	2398	-0.29	-0.37	-0.20	<0.0001	2342	-0.32	-0.38	-0.26	<0.0001
Phospholipids in very large HDL (mmol/l)	2398	-0.33	-0.41	-0.24	<0.0001	2342	-0.37	-0.43	-0.31	<0.0001
Total cholesterol in very large HDL (mmol/l)	2398	-0.23	-0.30	-0.15	<0.0001	2342	-0.25	-0.32	-0.19	<0.0001
Cholesterol esters in very large HDL (mmol/l)	2398	-0.21	-0.28	-0.13	<0.0001	2342	-0.23	-0.29	-0.16	<0.0001
Free cholesterol in very large HDL (mmol/l)	2398	-0.28	-0.36	-0.20	<0.0001	2342	-0.31	-0.38	-0.25	<0.0001
Triglycerides in very large HDL (mmol/l)	2398	0.03	-0.04	0.09	0.419	2342	0.00	-0.07	0.08	0.944
Concentration of large HDL particles (mol/l)	2398	-0.35	-0.43	-0.27	<0.0001	2342	-0.40	-0.46	-0.34	<0.0001
Total lipids in large HDL (mmol/l)	2398	-0.35	-0.43	-0.27	<0.0001	2342	-0.40	-0.46	-0.34	<0.0001
Phospholipids in large HDL (mmol/l)	2398	-0.31	-0.39	-0.24	<0.0001	2342	-0.36	-0.42	-0.30	<0.0001
Total cholesterol in large HDL (mmol/l)	2398	-0.38	-0.47	-0.29	<0.0001	2342	-0.44	-0.50	-0.38	<0.0001
Cholesterol esters in large HDL (mmol/l)	2398	-0.38	-0.47	-0.29	<0.0001	2342	-0.44	-0.50	-0.38	<0.0001
Free cholesterol in large HDL (mmol/l)	2398	-0.37	-0.46	-0.28	<0.0001	2342	-0.42	-0.48	-0.36	<0.0001
Triglycerides in large HDL (mmol/l)	2398	-0.02	-0.08	0.03	0.445	2342	-0.05	-0.12	0.02	0.143
Concentration of medium HDL particles (mol/l)	2398	-0.06	-0.12	-0.01	0.025	2342	-0.08	-0.14	-0.02	0.013
Total lipids in medium HDL (mmol/l)	2398	-0.09	-0.14	-0.04	0.001	2342	-0.11	-0.17	-0.04	0.001

Online Table 6 Associations of change in body mass index (BMI) and fat mass index from age 10y-18y with cardiometabolic traits at age 18y in ALSPAC

	Change from age 10-18y									
	BMI (per SD-unit gain)					Fat mass index (per SD-unit gain)				
	Adj. for age, sex, ethnicity, maternal education, BMI at 10y					*Adj. for age, sex, ethnicity, maternal education, fat mass index at 10y*				
**Standardized outcome at age 18y**	**N**	**Beta**	**LCL**	**UCL**	**P-value**	**N**	**Beta**	**LCL**	**UCL**	**P-value**
Phospholipids in medium HDL (mmol/l)	2398	-0.09	-0.15	-0.04	0.001	2342	-0.11	-0.18	-0.05	0.0003
Total cholesterol in medium HDL (mmol/l)	2398	-0.14	-0.19	-0.08	<0.0001	2342	-0.16	-0.22	-0.09	<0.0001
Cholesterol esters in medium HDL (mmol/l)	2398	-0.15	-0.21	-0.09	<0.0001	2342	-0.17	-0.23	-0.10	<0.0001
Free cholesterol in medium HDL (mmol/l)	2398	-0.08	-0.13	-0.02	0.004	2342	-0.09	-0.15	-0.03	0.005
Triglycerides in medium HDL (mmol/l)	2398	0.34	0.25	0.44	<0.0001	2342	0.37	0.30	0.45	<0.0001
Concentration of small HDL particles (mol/l)	2398	0.11	0.03	0.18	0.004	2342	0.12	0.05	0.18	0.001
Total lipids in small HDL (mmol/l)	2398	0.03	-0.03	0.09	0.268	2342	0.04	-0.03	0.10	0.250
Phospholipids in small HDL (mmol/l)	2398	0.07	-0.01	0.14	0.072	2342	0.07	0.00	0.14	0.068
Total cholesterol in small HDL (mmol/l)	2398	-0.05	-0.10	0.00	0.046	2342	-0.04	-0.10	0.01	0.132
Cholesterol esters in small HDL (mmol/l)	2398	-0.04	-0.09	0.01	0.141	2342	-0.02	-0.08	0.03	0.420
Free cholesterol in small HDL (mmol/l)	2398	-0.08	-0.14	-0.02	0.006	2342	-0.10	-0.17	-0.04	0.003
Triglycerides in small HDL (mmol/l)	2398	0.28	0.20	0.36	<0.0001	2342	0.29	0.21	0.37	<0.0001
Phospholipids to total lipids ratio in chylomicrons and extremely large VLDL (%)	2398	0.00	-0.03	0.03	0.883	2342	-0.01	-0.04	0.03	0.759
Total cholesterol to total lipids ratio in chylomicrons and extremely large VLDL (%)	2398	0.19	0.13	0.25	<0.0001	2342	0.20	0.13	0.27	<0.0001
Cholesterol esters to total lipids ratio in chylomicrons and extremely large VLDL (%)	2398	0.16	0.10	0.22	<0.0001	2342	0.16	0.09	0.23	<0.0001
Free cholesterol to total lipids ratio in chylomicrons and extremely large VLDL (%)	2398	0.16	0.09	0.22	<0.0001	2342	0.18	0.11	0.24	<0.0001
Triglycerides to total lipids ratio in chylomicrons and extremely large VLDL (%)	2398	-0.16	-0.22	-0.11	<0.0001	2342	-0.17	-0.22	-0.11	<0.0001
Phospholipids to total lipds ratio in very large VLDL (%)	2398	0.24	0.18	0.31	<0.0001	2342	0.27	0.21	0.33	<0.0001
Total cholesterol to total lipids ratio in very large VLDL (%)	2398	-0.08	-0.14	-0.02	0.005	2342	-0.10	-0.16	-0.04	0.002
Cholesterol esters to total lipids ratio in very large VLDL (%)	2398	-0.08	-0.14	-0.01	0.018	2342	-0.09	-0.15	-0.03	0.003
Free cholesterol to total lipids ratio in very large VLDL (%)	2398	-0.06	-0.12	0.00	0.053	2342	-0.07	-0.12	-0.01	0.016
Triglycerides to total lipids ratio in very large VLDL (%)	2398	-0.03	-0.08	0.02	0.286	2342	-0.01	-0.07	0.05	0.769
Phospholipids to total lipds ratio in large VLDL (%)	2398	0.20	0.14	0.26	<0.0001	2342	0.23	0.17	0.28	<0.0001
Total cholesterol to total lipids ratio in large VLDL (%)	2398	0.23	0.18	0.29	<0.0001	2342	0.24	0.18	0.30	<0.0001
Cholesterol esters to total lipids ratio in large VLDL (%)	2398	0.09	0.05	0.14	0.0001	2342	0.08	0.02	0.14	0.006
Free cholesterol to total lipids ratio in large VLDL (%)	2398	0.13	0.10	0.17	<0.0001	2342	0.14	0.11	0.17	<0.0001
Triglycerides to total lipids ratio in large VLDL (%)	2398	-0.24	-0.30	-0.18	<0.0001	2342	-0.25	-0.32	-0.19	<0.0001
Phospholipids to total lipds ratio in medium VLDL (%)	2398	-0.27	-0.34	-0.20	<0.0001	2342	-0.31	-0.37	-0.25	<0.0001
Total cholesterol to total lipids ratio in medium VLDL (%)	2398	0.13	0.07	0.18	<0.0001	2342	0.13	0.07	0.20	<0.0001
Cholesterol esters to total lipids ratio in medium VLDL (%)	2398	0.11	0.06	0.17	0.0001	2342	0.11	0.05	0.18	0.001
Free cholesterol to total lipids ratio in medium VLDL (%)	2398	0.12	0.07	0.18	<0.0001	2342	0.12	0.06	0.19	<0.0001
Triglycerides to total lipids ratio in medium VLDL (%)	2398	-0.07	-0.12	-0.01	0.018	2342	-0.06	-0.12	0.01	0.078
Phospholipids to total lipds ratio in small VLDL (%)	2398	-0.33	-0.40	-0.26	<0.0001	2342	-0.38	-0.45	-0.31	<0.0001
Total cholesterol to total lipids ratio in small VLDL (%)	2398	-0.01	-0.07	0.05	0.807	2342	0.03	-0.04	0.10	0.467
Cholesterol esters to total lipids ratio in small VLDL (%)	2398	0.03	-0.03	0.09	0.279	2342	0.08	0.01	0.15	0.036
Free cholesterol to total lipids ratio in small VLDL (%)	2398	-0.27	-0.34	-0.20	<0.0001	2342	-0.32	-0.39	-0.26	<0.0001
Triglycerides to total lipids ratio in small VLDL (%)	2398	0.12	0.06	0.19	0.0002	2342	0.10	0.03	0.18	0.005
Phospholipids to total lipds ratio in very small VLDL (%)	2398	-0.04	-0.10	0.02	0.166	2342	-0.04	-0.10	0.03	0.276
Total cholesterol to total lipids ratio in very small VLDL (%)	2398	-0.04	-0.10	0.02	0.165	2342	-0.03	-0.10	0.04	0.402
Cholesterol esters to total lipids ratio in very small VLDL (%)	2398	0.04	-0.01	0.10	0.136	2342	0.07	0.01	0.14	0.029
Free cholesterol to total lipids ratio in very small VLDL (%)	2398	-0.30	-0.39	-0.22	<0.0001	2342	-0.36	-0.44	-0.28	<0.0001
Triglycerides to total lipids ratio in very small VLDL (%)	2398	0.08	0.01	0.14	0.023	2342	0.06	-0.02	0.14	0.127
Phospholipids to total lipds ratio in IDL (%)	2398	-0.31	-0.39	-0.23	<0.0001	2342	-0.34	-0.41	-0.26	<0.0001
Total cholesterol to total lipids ratio in IDL (%)	2398	0.17	0.10	0.24	<0.0001	2342	0.21	0.14	0.28	<0.0001
Cholesterol esters to total lipids ratio in IDL (%)	2398	0.30	0.22	0.37	<0.0001	2342	0.34	0.27	0.41	<0.0001
Free cholesterol to total lipids ratio in IDL (%)	2398	-0.28	-0.36	-0.20	<0.0001	2342	-0.29	-0.37	-0.21	<0.0001
Triglycerides to total lipids ratio in IDL (%)	2398	-0.08	-0.14	-0.01	0.020	2342	-0.11	-0.19	-0.04	0.003
Phospholipids to total lipds ratio in large LDL (%)	2398	-0.06	-0.12	-0.01	0.028	2342	-0.08	-0.14	-0.01	0.024
Total cholesterol to total lipids ratio in large LDL (%)	2398	0.13	0.07	0.19	<0.0001	2342	0.17	0.09	0.24	<0.0001
Cholesterol esters to total lipids ratio in large LDL (%)	2398	0.21	0.15	0.27	<0.0001	2342	0.25	0.18	0.32	<0.0001
Free cholesterol to total lipids ratio in large LDL (%)	2398	-0.30	-0.38	-0.23	<0.0001	2342	-0.33	-0.40	-0.26	<0.0001
Triglycerides to total lipids ratio in large LDL (%)	2398	-0.14	-0.21	-0.07	<0.0001	2342	-0.18	-0.26	-0.11	<0.0001
Phospholipids to total lipds ratio in medium LDL (%)	2398	-0.05	-0.11	0.01	0.079	2342	-0.06	-0.13	0.00	0.065
Total cholesterol to total lipids ratio in medium LDL (%)	2398	0.10	0.04	0.16	0.001	2342	0.12	0.05	0.19	0.001
Cholesterol esters to total lipids ratio in medium LDL (%)	2398	0.14	0.08	0.20	<0.0001	2342	0.16	0.09	0.23	<0.0001
Free cholesterol to total lipids ratio in medium LDL (%)	2398	-0.16	-0.22	-0.11	<0.0001	2342	-0.19	-0.25	-0.12	<0.0001
Triglycerides to total lipids ratio in medium LDL (%)	2398	-0.12	-0.18	-0.06	<0.0001	2342	-0.15	-0.21	-0.08	<0.0001
Phospholipids to total lipds ratio in small LDL (%)	2398	-0.11	-0.16	-0.05	0.0003	2342	-0.13	-0.20	-0.06	0.0001
Total cholesterol to total lipids ratio in small LDL (%)	2398	0.07	0.01	0.13	0.019	2342	0.10	0.03	0.17	0.006
Cholesterol esters to total lipids ratio in small LDL (%)	2398	0.11	0.06	0.17	0.0002	2342	0.14	0.07	0.21	<0.0001
Free cholesterol to total lipids ratio in small LDL (%)	2398	-0.16	-0.22	-0.10	<0.0001	2342	-0.19	-0.25	-0.12	<0.0001
Triglycerides to total lipids ratio in small LDL (%)	2398	0.08	0.01	0.14	0.018	2342	0.07	-0.01	0.14	0.087
Phospholipids to total lipds ratio in very large HDL (%)	2398	-0.35	-0.44	-0.27	<0.0001	2342	-0.38	-0.45	-0.32	<0.0001
Total cholesterol to total lipids ratio in very large HDL (%)	2398	0.30	0.23	0.38	<0.0001	2342	0.33	0.27	0.40	<0.0001
Cholesterol esters to total lipids ratio in very large HDL (%)	2398	0.30	0.22	0.38	<0.0001	2342	0.33	0.27	0.40	<0.0001
Free cholesterol to total lipids ratio in very large HDL (%)	2398	-0.10	-0.17	-0.03	0.006	2342	-0.10	-0.17	-0.02	0.008
Triglycerides to total lipids ratio in very large HDL (%)	2398	0.32	0.23	0.40	<0.0001	2342	0.33	0.24	0.41	<0.0001
Phospholipids to total lipds ratio in large HDL (%)	2398	0.45	0.34	0.57	<0.0001	2342	0.52	0.44	0.59	<0.0001
Total cholesterol to total lipids ratio in large HDL (%)	2398	-0.48	-0.59	-0.36	<0.0001	2342	-0.54	-0.61	-0.46	<0.0001
Cholesterol esters to total lipids ratio in large HDL (%)	2398	-0.47	-0.59	-0.36	<0.0001	2342	-0.54	-0.62	-0.46	<0.0001
Free cholesterol to total lipids ratio in large HDL (%)	2398	-0.37	-0.47	-0.27	<0.0001	2342	-0.40	-0.48	-0.32	<0.0001
Triglycerides to total lipids ratio in large HDL (%)	2398	0.40	0.30	0.50	<0.0001	2342	0.44	0.37	0.52	<0.0001
Phospholipids to total lipds ratio in medium HDL (%)	2398	-0.06	-0.11	0.00	0.056	2342	-0.07	-0.14	-0.01	0.024
Total cholesterol to total lipids ratio in medium HDL (%)	2398	-0.15	-0.22	-0.09	<0.0001	2342	-0.16	-0.23	-0.09	<0.0001
Cholesterol esters to total lipids ratio in medium HDL (%)	2398	-0.17	-0.24	-0.11	<0.0001	2342	-0.18	-0.25	-0.12	<0.0001
Free cholesterol to total lipids ratio in medium HDL (%)	2398	0.02	-0.03	0.08	0.407	2342	0.04	-0.02	0.11	0.201
Triglycerides to total lipids ratio in medium HDL (%)	2398	0.42	0.32	0.52	<0.0001	2342	0.47	0.39	0.54	<0.0001
Phospholipids to total lipds ratio in small HDL (%)	2398	0.06	0.00	0.12	0.063	2342	0.05	-0.01	0.11	0.128
Total cholesterol to total lipids ratio in small HDL (%)	2398	-0.14	-0.20	-0.07	<0.0001	2342	-0.13	-0.19	-0.07	<0.0001
Cholesterol esters to total lipids ratio in small HDL (%)	2398	-0.08	-0.14	-0.02	0.015	2342	-0.06	-0.12	0.00	0.058
Free cholesterol to total lipids ratio in small HDL (%)	2398	-0.32	-0.39	-0.24	<0.0001	2342	-0.40	-0.47	-0.33	<0.0001
Triglycerides to total lipids ratio in small HDL (%)	2398	0.29	0.21	0.37	<0.0001	2342	0.31	0.23	0.39	<0.0001
Mean diameter for VLDL particles (nm)	2398	0.40	0.31	0.50	<0.0001	2342	0.44	0.37	0.51	<0.0001
Mean diameter for LDL particles (nm)	2398	-0.18	-0.24	-0.11	<0.0001	2342	-0.20	-0.26	-0.14	<0.0001
Mean diameter for HDL particles (nm)	2398	-0.36	-0.45	-0.26	<0.0001	2342	-0.41	-0.47	-0.35	<0.0001
Serum total cholesterol (mmol/l)	2398	0.13	0.07	0.19	<0.0001	2342	0.17	0.10	0.24	<0.0001
Total cholesterol in VLDL (mmol/l)	2398	0.44	0.34	0.54	<0.0001	2342	0.50	0.42	0.58	<0.0001
Remnant cholesterol (non-HDL, non-LDL -cholesterol) (mmol/l)	2398	0.35	0.27	0.43	<0.0001	2342	0.41	0.33	0.48	<0.0001

Online Table 6 Associations of change in body mass index (BMI) and fat mass index from age 10y-18y with cardiometabolic traits at age 18y in ALSPAC

Change from age 10-18y

	BMI (per SD-unit gain)					Fat mass index (per SD-unit gain)				
	Adj. for age, sex, ethnicity, maternal education, BMI at 10y					*Adj. for age, sex, ethnicity, maternal education, fat mass index at 10y*				
**Standardized outcome at age 18y**	**N**	**Beta**	**LCL**	**UCL**	**P-value**	**N**	**Beta**	**LCL**	**UCL**	**P-value**
Total cholesterol in LDL (mmol/l)	2398	0.16	0.10	0.23	<0.0001	2342	0.21	0.13	0.28	<0.0001
Total cholesterol in HDL (mmol/l)	2398	-0.28	-0.35	-0.21	<0.0001	2342	-0.32	-0.37	-0.26	<0.0001
Total cholesterol in HDL2 (mmol/l)	2398	-0.32	-0.40	-0.25	<0.0001	2342	-0.36	-0.42	-0.30	<0.0001
Total cholesterol in HDL3 (mmol/l)	2398	-0.19	-0.25	-0.13	<0.0001	2342	-0.22	-0.28	-0.16	<0.0001
Esterified cholesterol (mmol/l)	2388	0.13	0.07	0.20	<0.0001	2332	0.17	0.10	0.24	<0.0001
Free cholesterol (mmol/l)	2386	0.13	0.07	0.18	<0.0001	2330	0.15	0.08	0.22	<0.0001
Serum total triglycerides (mmol/l)	2398	0.40	0.31	0.50	<0.0001	2342	0.44	0.36	0.52	<0.0001
Triglycerides in VLDL (mmol/l)	2398	0.44	0.34	0.55	<0.0001	2342	0.49	0.40	0.57	<0.0001
Triglycerides in LDL (mmol/l)	2398	0.04	-0.01	0.10	0.117	2342	0.05	-0.02	0.11	0.174
Triglycerides in HDL (mmol/l)	2398	0.25	0.17	0.33	<0.0001	2342	0.25	0.18	0.33	<0.0001
Diacylglycerol (mmol/l)	2328	0.29	0.22	0.37	<0.0001	2276	0.29	0.22	0.37	<0.0001
Ratio of diacylglycerol to triglycerides	2329	0.11	0.06	0.17	0.0001	2277	0.08	0.01	0.15	0.021
Total phosphoglycerides (mmol/l)	2386	0.01	-0.05	0.06	0.799	2330	-0.01	-0.07	0.05	0.745
Ratio of triglycerides to phosphoglycerides	2386	0.40	0.30	0.50	<0.0001	2330	0.44	0.36	0.52	<0.0001
Phosphatidylcholine and other cholines (mmol/l)	2369	0.02	-0.03	0.07	0.474	2314	-0.01	-0.07	0.05	0.746
Total cholines (mmol/l)	2388	0.02	-0.04	0.07	0.558	2332	0.00	-0.07	0.06	0.908
Apolipoprotein A-I (g/l)	2398	-0.14	-0.19	-0.08	<0.0001	2342	-0.15	-0.21	-0.10	<0.0001
Apolipoprotein B (g/l)	2398	0.37	0.28	0.45	<0.0001	2342	0.42	0.35	0.50	<0.0001
Ratio of apolipoprotein B to apolipoprotein A-I	2398	0.43	0.34	0.52	<0.0001	2342	0.49	0.42	0.57	<0.0001
Total fatty acids (mmol/l)	2388	0.24	0.17	0.30	<0.0001	2332	0.26	0.18	0.33	<0.0001
Estimated description of fatty acid chain length, not actual carbon number	2389	0.02	-0.04	0.09	0.479	2333	0.02	-0.06	0.10	0.594
Estimated degree of unsaturation	2388	-0.09	-0.15	-0.04	0.002	2332	-0.10	-0.16	-0.03	0.005
22:6, docosahexaenoic acid (mmol/l)	2388	0.08	0.02	0.13	0.009	2332	0.09	0.02	0.15	0.009
18:2, linoleic acid (mmol/l)	2388	0.12	0.06	0.18	<0.0001	2332	0.11	0.05	0.18	0.001
Conjugated linoleic acid (mmol/l)	2387	0.13	0.07	0.19	<0.0001	2331	0.13	0.06	0.20	0.0003
Omega-3 fatty acids (mmol/l)	2388	0.16	0.09	0.23	<0.0001	2332	0.18	0.11	0.26	<0.0001
Omega-6 fatty acids (mmol/l)	2388	0.15	0.09	0.21	<0.0001	2332	0.15	0.08	0.22	<0.0001
Polyunsaturated fatty acids (mmol/l)	2388	0.16	0.10	0.22	<0.0001	2332	0.16	0.09	0.24	<0.0001
Monounsaturated fatty acids; 16:1, 18:1 (mmol/l)	2388	0.28	0.21	0.36	<0.0001	2332	0.33	0.26	0.40	<0.0001
Saturated fatty acids (mmol/l)	2387	0.21	0.15	0.28	<0.0001	2331	0.21	0.14	0.29	<0.0001
Ratio of 22:6 docosahexaenoic acid to total fatty acids (%)	2389	-0.06	-0.11	0.00	0.035	2333	-0.05	-0.12	0.01	0.092
Ratio of 18:2 linoleic acid to total fatty acids (%)	2389	-0.21	-0.30	-0.13	<0.0001	2333	-0.25	-0.32	-0.18	<0.0001
Ratio of conjugated linoleic acid to total fatty acids (%)	2388	0.09	0.04	0.15	0.001	2332	0.09	0.03	0.16	0.007
Ratio of omega-3 fatty acids to total fatty acids (%)	2389	-0.01	-0.07	0.05	0.721	2333	0.00	-0.07	0.07	0.897
Ratio of omega-6 fatty acids to total fatty acids (%)	2389	-0.21	-0.30	-0.12	<0.0001	2333	-0.24	-0.31	-0.17	<0.0001
Ratio of polyunsaturated fatty acids to total fatty acids (%)	2389	-0.20	-0.28	-0.12	<0.0001	2333	-0.23	-0.30	-0.15	<0.0001
Ratio of monounsaturated fatty acids to total fatty acids (%)	2389	0.22	0.15	0.30	<0.0001	2333	0.29	0.22	0.36	<0.0001
Ratio of saturated fatty acids to total fatty acids (%)	2388	-0.06	-0.12	0.01	0.077	2332	-0.12	-0.19	-0.04	0.003
Insulin (mu/l)	2435	0.49	0.31	0.67	<0.0001	2378	0.49	0.32	0.67	<0.0001
Glucose (mmol/l)	2397	0.14	0.09	0.20	<0.0001	2341	0.16	0.11	0.22	<0.0001
Lactate (mmol/l)	2397	0.00	-0.06	0.06	0.938	2341	0.02	-0.05	0.09	0.652
Pyruvate (mmol/l)	2397	0.14	0.08	0.20	<0.0001	2341	0.17	0.10	0.24	<0.0001
Citrate (mmol/l)	2397	-0.16	-0.23	-0.09	<0.0001	2341	-0.16	-0.23	-0.10	<0.0001
Alanine (mmol/l)	2397	0.13	0.07	0.19	<0.0001	2341	0.12	0.05	0.19	0.0005
Glutamine (mmol/l)	2397	-0.02	-0.09	0.05	0.488	2341	-0.03	-0.10	0.03	0.317
Histidine (mmol/l)	2397	0.06	0.00	0.12	0.037	2341	0.04	-0.03	0.11	0.239
Isoleucine (mmol/l)	2397	0.31	0.23	0.38	<0.0001	2341	0.30	0.23	0.37	<0.0001
Leucine (mmol/l)	2397	0.24	0.18	0.30	<0.0001	2341	0.20	0.14	0.27	<0.0001
Valine (mmol/l)	2397	0.27	0.20	0.35	<0.0001	2341	0.26	0.20	0.33	<0.0001
Phenylalanine (mmol/l)	2396	0.27	0.20	0.35	<0.0001	2341	0.26	0.19	0.33	<0.0001
Tyrosine (mmol/l)	2397	0.35	0.28	0.43	<0.0001	2341	0.38	0.31	0.45	<0.0001
Acetate (mmol/l)	2396	-0.07	-0.12	-0.01	0.016	2340	-0.09	-0.15	-0.04	0.001
Acetoacetate (mmol/l)	2397	-0.11	-0.19	-0.03	0.004	2341	-0.12	-0.18	-0.06	0.0002
3-hydroxybutyrate (mmol/l)	2394	-0.13	-0.19	-0.06	0.0003	2338	-0.12	-0.19	-0.05	0.001
Creatinine (mmol/l)	2397	0.14	0.09	0.20	<0.0001	2341	0.05	-0.01	0.11	0.076
Albumin (signal area)	2398	-0.10	-0.15	-0.04	0.001	2342	-0.09	-0.15	-0.03	0.005
Glycoprotein acetyls, mainly a1-acid glycoprotein (mmol/l)	2397	0.38	0.27	0.48	<0.0001	2341	0.45	0.38	0.53	<0.0001
C-reactive protein (mg/l)	2477	0.12	0.05	0.20	0.002	2419	0.15	0.06	0.24	0.001

Change from age 10-18y

Complete case sample

	BMI (per SD-unit gain)					Fat mass index (per SD-unit gain)				
	Adj. for age, sex, ethnicity, maternal education, BMI at 10y					*Adj. for age, sex, ethnicity, maternal education, fat mass index at 10y*				
**Standardized outcome at age 18y**	**N**	**Beta**	**LCL**	**UCL**	**P-value**	**N**	**Beta**	**LCL**	**UCL**	**P-value**
Systolic blood pressure (mmHg)	1722	0.33	0.26	0.39	<0.0001	1722	0.26	0.19	0.33	<0.0001
Diastolic blood pressure (mmHg)	1722	0.31	0.23	0.39	<0.0001	1722	0.37	0.29	0.45	<0.0001
Concentration of chylomicrons and extremely large VLDL particles (mol/l)	1722	0.48	0.40	0.56	<0.0001	1722	0.52	0.43	0.61	<0.0001
Total lipids in chylomicrons and extremely large VLDL (mmol/l)	1722	0.47	0.39	0.55	<0.0001	1722	0.51	0.41	0.60	<0.0001
Phospholipids in chylomicrons and extremely large VLDL (mmol/l)	1722	0.46	0.38	0.54	<0.0001	1722	0.50	0.41	0.59	<0.0001
Total cholesterol in chylomicrons and extremely large VLDL (mmol/l)	1722	0.48	0.39	0.56	<0.0001	1722	0.51	0.42	0.61	<0.0001
Cholesterol esters in chylomicrons and extremely large VLDL (mmol/l)	1722	0.48	0.39	0.57	<0.0001	1722	0.51	0.41	0.61	<0.0001
Free cholesterol in chylomicrons and extremely large VLDL (mmol/l)	1722	0.46	0.38	0.54	<0.0001	1722	0.50	0.40	0.59	<0.0001
Triglycerides in chylomicrons and extremely large VLDL (mmol/l)	1722	0.47	0.38	0.55	<0.0001	1722	0.51	0.41	0.60	<0.0001
Concentration of very large VLDL particles (mol/l)	1722	0.46	0.38	0.55	<0.0001	1722	0.49	0.40	0.59	<0.0001
Total lipids in very large VLDL (mmol/l)	1722	0.46	0.38	0.55	<0.0001	1722	0.49	0.40	0.58	<0.0001
Phospholipids in very large VLDL (mmol/l)	1722	0.46	0.38	0.55	<0.0001	1722	0.49	0.40	0.59	<0.0001
Total cholesterol in very large VLDL (mmol/l)	1722	0.49	0.40	0.57	<0.0001	1722	0.52	0.43	0.61	<0.0001
Cholesterol esters in very large VLDL (mmol/l)	1722	0.50	0.41	0.58	<0.0001	1722	0.53	0.43	0.62	<0.0001
Free cholesterol in very large VLDL (mmol/l)	1722	0.47	0.39	0.56	<0.0001	1722	0.51	0.42	0.60	<0.0001
Triglycerides in very large VLDL (mmol/l)	1722	0.45	0.37	0.53	<0.0001	1722	0.48	0.38	0.57	<0.0001
Concentration of large VLDL particles (mol/l)	1722	0.45	0.36	0.53	<0.0001	1722	0.47	0.38	0.56	<0.0001
Total lipids in large VLDL (mmol/l)	1722	0.45	0.37	0.54	<0.0001	1722	0.48	0.38	0.57	<0.0001
Phospholipids in large VLDL (mmol/l)	1722	0.45	0.37	0.53	<0.0001	1722	0.47	0.38	0.57	<0.0001

Online Table 6 Associations of change in body mass index (BMI) and fat mass index from age 10y-18y with cardiometabolic traits at age 18y in ALSPAC

	**Change from age 10-18y**									
	**BMI (per SD-unit gain)**					**Fat mass index (per SD-unit gain)**				
	Adj. for age, sex, ethnicity, maternal education, BMI at 10y					*Adj. for age, sex, ethnicity, maternal education, fat mass index at 10y*				
**Standardized outcome at age 18y**	**N**	**Beta**	**LCL**	**UCL**	**P-value**	**N**	**Beta**	**LCL**	**UCL**	**P-value**
Total cholesterol in large VLDL (mmol/l)	1722	0.47	0.39	0.55	<0.0001	1722	0.50	0.40	0.59	<0.0001
Cholesterol esters in large VLDL (mmol/l)	1722	0.48	0.40	0.57	<0.0001	1722	0.51	0.41	0.60	<0.0001
Free cholesterol in large VLDL (mmol/l)	1722	0.45	0.37	0.54	<0.0001	1722	0.48	0.38	0.57	<0.0001
Triglycerides in large VLDL (mmol/l)	1722	0.44	0.36	0.53	<0.0001	1722	0.47	0.37	0.56	<0.0001
Concentration of medium VLDL particles (mol/l)	1722	0.46	0.38	0.55	<0.0001	1722	0.49	0.40	0.58	<0.0001
Total lipids in medium VLDL (mmol/l)	1722	0.47	0.39	0.55	<0.0001	1722	0.50	0.40	0.59	<0.0001
Phospholipids in medium VLDL (mmol/l)	1722	0.46	0.38	0.54	<0.0001	1722	0.49	0.39	0.58	<0.0001
Total cholesterol in medium VLDL (mmol/l)	1722	0.48	0.39	0.56	<0.0001	1722	0.50	0.41	0.59	<0.0001
Cholesterol esters in medium VLDL (mmol/l)	1722	0.48	0.40	0.56	<0.0001	1722	0.50	0.41	0.60	<0.0001
Free cholesterol in medium VLDL (mmol/l)	1722	0.45	0.36	0.53	<0.0001	1722	0.47	0.38	0.56	<0.0001
Triglycerides in medium VLDL (mmol/l)	1722	0.46	0.38	0.54	<0.0001	1722	0.48	0.39	0.57	<0.0001
Concentration of small VLDL particles (mol/l)	1722	0.44	0.36	0.52	<0.0001	1722	0.47	0.38	0.56	<0.0001
Total lipids in small VLDL (mmol/l)	1722	0.45	0.37	0.53	<0.0001	1722	0.48	0.39	0.57	<0.0001
Phospholipids in small VLDL (mmol/l)	1722	0.42	0.34	0.50	<0.0001	1722	0.44	0.35	0.53	<0.0001
Total cholesterol in small VLDL (mmol/l)	1722	0.45	0.37	0.53	<0.0001	1722	0.48	0.39	0.57	<0.0001
Cholesterol esters in small VLDL (mmol/l)	1722	0.44	0.36	0.52	<0.0001	1722	0.48	0.39	0.57	<0.0001
Free cholesterol in small VLDL (mmol/l)	1722	0.41	0.33	0.49	<0.0001	1722	0.43	0.34	0.52	<0.0001
Triglycerides in small VLDL (mmol/l)	1722	0.41	0.33	0.49	<0.0001	1722	0.44	0.35	0.53	<0.0001
Concentration of very small VLDL particles (mol/l)	1722	0.28	0.21	0.36	<0.0001	1722	0.30	0.22	0.39	<0.0001
Total lipids in very small VLDL (mmol/l)	1722	0.33	0.25	0.41	<0.0001	1722	0.35	0.26	0.44	<0.0001
Phospholipids in very small VLDL (mmol/l)	1722	0.25	0.17	0.32	<0.0001	1722	0.27	0.18	0.36	<0.0001
Total cholesterol in very small VLDL (mmol/l)	1722	0.32	0.23	0.40	<0.0001	1722	0.34	0.25	0.43	<0.0001
Cholesterol esters in very small VLDL (mmol/l)	1722	0.35	0.27	0.43	<0.0001	1722	0.38	0.29	0.46	<0.0001
Free cholesterol in very small VLDL (mmol/l)	1722	0.21	0.13	0.29	<0.0001	1722	0.22	0.12	0.31	<0.0001
Triglycerides in very small VLDL (mmol/l)	1722	0.29	0.21	0.36	<0.0001	1722	0.31	0.22	0.39	<0.0001
Concentration of IDL particles (mol/l)	1722	0.19	0.11	0.27	<0.0001	1722	0.21	0.13	0.30	<0.0001
Total lipids in IDL (mmol/l)	1722	0.21	0.13	0.29	<0.0001	1722	0.24	0.15	0.32	<0.0001
Phospholipids in IDL (mmol/l)	1722	0.17	0.09	0.25	<0.0001	1722	0.19	0.11	0.28	<0.0001
Total cholesterol in IDL (mmol/l)	1722	0.24	0.16	0.32	<0.0001	1722	0.26	0.17	0.35	<0.0001
Cholesterol esters in IDL (mmol/l)	1722	0.28	0.20	0.36	<0.0001	1722	0.30	0.21	0.40	<0.0001
Free cholesterol in IDL (mmol/l)	1722	0.14	0.06	0.22	0.001	1722	0.16	0.07	0.24	0.0004
Triglycerides in IDL (mmol/l)	1722	0.07	0.00	0.14	0.068	1722	0.08	0.00	0.16	0.044
Concentration of large LDL particles (mol/l)	1722	0.19	0.12	0.27	<0.0001	1722	0.22	0.13	0.30	<0.0001
Total lipids in large LDL (mmol/l)	1722	0.20	0.13	0.28	<0.0001	1722	0.23	0.14	0.31	<0.0001
Phospholipids in large LDL (mmol/l)	1722	0.23	0.15	0.31	<0.0001	1722	0.26	0.17	0.34	<0.0001
Total cholesterol in large LDL (mmol/l)	1722	0.21	0.14	0.29	<0.0001	1722	0.24	0.15	0.33	<0.0001
Cholesterol esters in large LDL (mmol/l)	1722	0.23	0.15	0.31	<0.0001	1722	0.26	0.17	0.34	<0.0001
Free cholesterol in large LDL (mmol/l)	1722	0.16	0.08	0.24	<0.0001	1722	0.18	0.09	0.27	<0.0001
Triglycerides in large LDL (mmol/l)	1722	0.02	-0.05	0.09	0.591	1722	0.03	-0.05	0.11	0.426
Concentration of medium LDL particles (mol/l)	1722	0.22	0.14	0.30	<0.0001	1722	0.24	0.16	0.33	<0.0001
Total lipids in medium LDL (mmol/l)	1722	0.22	0.14	0.30	<0.0001	1722	0.24	0.16	0.33	<0.0001
Phospholipids in medium LDL (mmol/l)	1722	0.27	0.19	0.35	<0.0001	1722	0.30	0.21	0.38	<0.0001
Total cholesterol in medium LDL (mmol/l)	1722	0.22	0.15	0.30	<0.0001	1722	0.25	0.16	0.34	<0.0001
Cholesterol esters in medium LDL (mmol/l)	1722	0.23	0.15	0.31	<0.0001	1722	0.25	0.16	0.34	<0.0001
Free cholesterol in medium LDL (mmol/l)	1722	0.21	0.13	0.29	<0.0001	1722	0.23	0.14	0.32	<0.0001
Triglycerides in medium LDL (mmol/l)	1722	0.01	-0.06	0.08	0.786	1722	0.03	-0.05	0.10	0.498
Concentration of small LDL particles (mol/l)	1722	0.21	0.14	0.29	<0.0001	1722	0.24	0.15	0.32	<0.0001
Total lipids in small LDL (mmol/l)	1722	0.23	0.15	0.30	<0.0001	1722	0.25	0.16	0.34	<0.0001
Phospholipids in small LDL (mmol/l)	1722	0.24	0.17	0.32	<0.0001	1722	0.27	0.18	0.35	<0.0001
Total cholesterol in small LDL (mmol/l)	1722	0.22	0.14	0.30	<0.0001	1722	0.24	0.16	0.33	<0.0001
Cholesterol esters in small LDL (mmol/l)	1722	0.22	0.14	0.30	<0.0001	1722	0.24	0.16	0.33	<0.0001
Free cholesterol in small LDL (mmol/l)	1722	0.21	0.13	0.29	<0.0001	1722	0.23	0.14	0.32	<0.0001
Triglycerides in small LDL (mmol/l)	1722	0.16	0.09	0.23	<0.0001	1722	0.18	0.10	0.26	<0.0001
Concentration of very large HDL particles (mol/l)	1722	-0.31	-0.38	-0.24	<0.0001	1722	-0.31	-0.38	-0.24	<0.0001
Total lipids in very large HDL (mmol/l)	1722	-0.30	-0.37	-0.23	<0.0001	1722	-0.30	-0.37	-0.23	<0.0001
Phospholipids in very large HDL (mmol/l)	1722	-0.34	-0.41	-0.27	<0.0001	1722	-0.35	-0.42	-0.27	<0.0001
Total cholesterol in very large HDL (mmol/l)	1722	-0.24	-0.31	-0.17	<0.0001	1722	-0.24	-0.31	-0.16	<0.0001
Cholesterol esters in very large HDL (mmol/l)	1722	-0.22	-0.29	-0.15	<0.0001	1722	-0.21	-0.29	-0.14	<0.0001
Free cholesterol in very large HDL (mmol/l)	1722	-0.29	-0.36	-0.22	<0.0001	1722	-0.29	-0.37	-0.22	<0.0001
Triglycerides in very large HDL (mmol/l)	1722	0.03	-0.05	0.11	0.473	1722	0.03	-0.05	0.12	0.443
Concentration of large HDL particles (mol/l)	1722	-0.37	-0.43	-0.30	<0.0001	1722	-0.39	-0.46	-0.32	<0.0001
Total lipids in large HDL (mmol/l)	1722	-0.37	-0.44	-0.30	<0.0001	1722	-0.39	-0.46	-0.32	<0.0001
Phospholipids in large HDL (mmol/l)	1722	-0.33	-0.40	-0.27	<0.0001	1722	-0.35	-0.42	-0.28	<0.0001
Total cholesterol in large HDL (mmol/l)	1722	-0.41	-0.47	-0.34	<0.0001	1722	-0.43	-0.50	-0.35	<0.0001
Cholesterol esters in large HDL (mmol/l)	1722	-0.41	-0.48	-0.34	<0.0001	1722	-0.43	-0.50	-0.36	<0.0001
Free cholesterol in large HDL (mmol/l)	1722	-0.39	-0.46	-0.32	<0.0001	1722	-0.41	-0.48	-0.34	<0.0001
Triglycerides in large HDL (mmol/l)	1722	0.00	-0.08	0.07	0.894	1722	-0.02	-0.10	0.06	0.565
Concentration of medium HDL particles (mol/l)	1722	-0.08	-0.15	-0.01	0.019	1722	-0.10	-0.18	-0.03	0.005
Total lipids in medium HDL (mmol/l)	1722	-0.11	-0.18	-0.04	0.002	1722	-0.13	-0.20	-0.06	0.000
Phospholipids in medium HDL (mmol/l)	1722	-0.12	-0.18	-0.05	0.001	1722	-0.14	-0.21	-0.06	0.0002
Total cholesterol in medium HDL (mmol/l)	1722	-0.16	-0.23	-0.08	<0.0001	1722	-0.18	-0.26	-0.10	<0.0001
Cholesterol esters in medium HDL (mmol/l)	1722	-0.17	-0.24	-0.09	<0.0001	1722	-0.19	-0.27	-0.12	<0.0001
Free cholesterol in medium HDL (mmol/l)	1722	-0.09	-0.16	-0.01	0.018	1722	-0.11	-0.18	-0.03	0.006
Triglycerides in medium HDL (mmol/l)	1722	0.36	0.28	0.43	<0.0001	1722	0.36	0.28	0.44	<0.0001
Concentration of small HDL particles (mol/l)	1722	0.08	0.01	0.15	0.028	1722	0.07	-0.01	0.15	0.069
Total lipids in small HDL (mmol/l)	1722	0.01	-0.06	0.08	0.714	1722	0.00	-0.07	0.08	0.945
Phospholipids in small HDL (mmol/l)	1722	0.03	-0.04	0.11	0.383	1722	0.01	-0.07	0.09	0.722
Total cholesterol in small HDL (mmol/l)	1722	-0.05	-0.12	0.01	0.093	1722	-0.05	-0.12	0.01	0.114
Cholesterol esters in small HDL (mmol/l)	1722	-0.03	-0.09	0.03	0.318	1722	-0.02	-0.09	0.04	0.468
Free cholesterol in small HDL (mmol/l)	1722	-0.11	-0.19	-0.04	0.003	1722	-0.14	-0.22	-0.06	0.0005
Triglycerides in small HDL (mmol/l)	1722	0.26	0.19	0.34	<0.0001	1722	0.27	0.19	0.36	<0.0001
Phospholipids to total lipds ratio in chylomicrons and extremely large VLDL (%)	1722	-0.02	-0.06	0.02	0.438	1722	-0.01	-0.05	0.03	0.745
Total cholesterol to total lipids ratio in chylomicrons and extremely large VLDL (%)	1722	0.24	0.16	0.31	<0.0001	1722	0.23	0.15	0.31	<0.0001
Cholesterol esters to total lipids ratio in chylomicrons and extremely large VLDL (%)	1722	0.20	0.12	0.28	<0.0001	1722	0.19	0.11	0.28	<0.0001
Free cholesterol to total lipids ratio in chylomicrons and extremely large VLDL (%)	1722	0.15	0.08	0.22	<0.0001	1722	0.16	0.09	0.23	<0.0001
Triglycerides to total lipids ratio in chylomicrons and extremely large VLDL (%)	1722	-0.20	-0.27	-0.14	<0.0001	1722	-0.19	-0.26	-0.13	<0.0001
Phospholipids to total lipds ratio in very large VLDL (%)	1722	0.26	0.20	0.33	<0.0001	1722	0.28	0.21	0.35	<0.0001
Total cholesterol to total lipids ratio in very large VLDL (%)	1722	-0.06	-0.13	0.02	0.122	1722	-0.07	-0.15	0.01	0.074

Online Table 6 Associations of change in body mass index (BMI) and fat mass index from age 10y-18y with cardiometabolic traits at age 18y in ALSPAC

Change from age 10-18y

	BMI (per SD-unit gain) *Adj. for age, sex, ethnicity, maternal education, BMI at 10y*					Fat mass index (per SD-unit gain) *Adj. for age, sex, ethnicity, maternal education, fat mass index at 10y*				
**Standardized outcome at age 18y**	**N**	**Beta**	**LCL**	**UCL**	**P-value**	**N**	**Beta**	**LCL**	**UCL**	**P-value**
Cholesterol esters to total lipids ratio in very large VLDL (%)	1722	-0.04	-0.09	0.02	0.172	1722	-0.06	-0.11	0.00	0.064
Free cholesterol to total lipids ratio in very large VLDL (%)	1722	-0.01	-0.06	0.04	0.698	1722	-0.02	-0.08	0.03	0.397
Triglycerides to total lipids ratio in very large VLDL (%)	1722	-0.07	-0.14	-0.01	0.031	1722	-0.05	-0.12	0.02	0.158
Phospholipids to total lipds ratio in large VLDL (%)	1722	0.19	0.12	0.26	<0.0001	1722	0.21	0.14	0.28	<0.0001
Total cholesterol to total lipids ratio in large VLDL (%)	1722	0.26	0.20	0.33	<0.0001	1722	0.26	0.18	0.33	<0.0001
Cholesterol esters to total lipids ratio in large VLDL (%)	1722	0.13	0.07	0.19	<0.0001	1722	0.11	0.04	0.17	0.002
Free cholesterol to total lipids ratio in large VLDL (%)	1722	0.13	0.10	0.16	<0.0001	1722	0.13	0.10	0.17	<0.0001
Triglycerides to total lipids ratio in large VLDL (%)	1722	-0.26	-0.33	-0.19	<0.0001	1722	-0.26	-0.34	-0.19	<0.0001
Phospholipids to total lipds ratio in medium VLDL (%)	1722	-0.26	-0.32	-0.20	<0.0001	1722	-0.28	-0.35	-0.21	<0.0001
Total cholesterol to total lipids ratio in medium VLDL (%)	1722	0.17	0.10	0.24	<0.0001	1722	0.16	0.08	0.24	<0.0001
Cholesterol esters to total lipids ratio in medium VLDL (%)	1722	0.16	0.08	0.23	<0.0001	1722	0.15	0.07	0.22	0.0003
Free cholesterol to total lipids ratio in medium VLDL (%)	1722	0.12	0.06	0.19	0.0004	1722	0.13	0.06	0.20	0.001
Triglycerides to total lipids ratio in medium VLDL (%)	1722	-0.10	-0.17	-0.03	0.004	1722	-0.09	-0.17	-0.02	0.017
Phospholipids to total lipds ratio in small VLDL (%)	1722	-0.37	-0.45	-0.30	<0.0001	1722	-0.40	-0.49	-0.32	<0.0001
Total cholesterol to total lipids ratio in small VLDL (%)	1722	0.05	-0.03	0.13	0.196	1722	0.06	-0.02	0.14	0.132
Cholesterol esters to total lipids ratio in small VLDL (%)	1722	0.09	0.02	0.17	0.017	1722	0.11	0.03	0.19	0.008
Free cholesterol to total lipids ratio in small VLDL (%)	1722	-0.28	-0.35	-0.21	<0.0001	1722	-0.31	-0.38	-0.24	<0.0001
Triglycerides to total lipids ratio in small VLDL (%)	1722	0.08	0.00	0.15	0.049	1722	0.08	-0.01	0.16	0.069
Phospholipids to total lipds ratio in very small VLDL (%)	1722	-0.01	-0.08	0.06	0.715	1722	-0.01	-0.09	0.07	0.814
Total cholesterol to total lipids ratio in very small VLDL (%)	1722	-0.01	-0.08	0.06	0.822	1722	-0.01	-0.09	0.07	0.746
Cholesterol esters to total lipids ratio in very small VLDL (%)	1722	0.08	0.01	0.15	0.034	1722	0.09	0.01	0.16	0.024
Free cholesterol to total lipids ratio in very small VLDL (%)	1722	-0.29	-0.37	-0.21	<0.0001	1722	-0.34	-0.43	-0.25	<0.0001
Triglycerides to total lipids ratio in very small VLDL (%)	1722	0.02	-0.06	0.10	0.648	1722	0.02	-0.06	0.10	0.617
Phospholipids to total lipds ratio in IDL (%)	1722	-0.35	-0.43	-0.27	<0.0001	1722	-0.36	-0.45	-0.27	<0.0001
Total cholesterol to total lipids ratio in IDL (%)	1722	0.25	0.17	0.32	<0.0001	1722	0.25	0.17	0.33	<0.0001
Cholesterol esters to total lipids ratio in IDL (%)	1722	0.36	0.29	0.43	<0.0001	1722	0.37	0.29	0.45	<0.0001
Free cholesterol to total lipids ratio in IDL (%)	1722	-0.24	-0.32	-0.16	<0.0001	1722	-0.26	-0.34	-0.17	<0.0001
Triglycerides to total lipids ratio in IDL (%)	1722	-0.15	-0.22	-0.07	0.0001	1722	-0.15	-0.23	-0.07	0.0002
Phospholipids to total lipds ratio in large LDL (%)	1722	-0.09	-0.17	-0.02	0.016	1722	-0.10	-0.18	-0.02	0.014
Total cholesterol to total lipids ratio in large LDL (%)	1722	0.20	0.12	0.27	<0.0001	1722	0.20	0.12	0.28	<0.0001
Cholesterol esters to total lipids ratio in large LDL (%)	1722	0.27	0.19	0.34	<0.0001	1722	0.28	0.20	0.36	<0.0001
Free cholesterol to total lipids ratio in large LDL (%)	1722	-0.31	-0.38	-0.23	<0.0001	1722	-0.32	-0.40	-0.24	<0.0001
Triglycerides to total lipids ratio in large LDL (%)	1722	-0.21	-0.29	-0.14	<0.0001	1722	-0.21	-0.29	-0.14	<0.0001
Phospholipids to total lipds ratio in medium LDL (%)	1722	-0.09	-0.16	-0.01	0.029	1722	-0.09	-0.17	0.00	0.038
Total cholesterol to total lipids ratio in medium LDL (%)	1722	0.16	0.08	0.24	<0.0001	1722	0.15	0.07	0.24	0.0003
Cholesterol esters to total lipids ratio in medium LDL (%)	1722	0.19	0.11	0.26	<0.0001	1722	0.19	0.10	0.27	<0.0001
Free cholesterol to total lipids ratio in medium LDL (%)	1722	-0.19	-0.26	-0.12	<0.0001	1722	-0.20	-0.28	-0.12	<0.0001
Triglycerides to total lipids ratio in medium LDL (%)	1722	-0.18	-0.24	-0.11	<0.0001	1722	-0.17	-0.24	-0.10	<0.0001
Phospholipids to total lipds ratio in small LDL (%)	1722	-0.15	-0.22	-0.07	0.0002	1722	-0.15	-0.23	-0.07	0.0002
Total cholesterol to total lipids ratio in small LDL (%)	1722	0.13	0.05	0.21	0.002	1722	0.13	0.05	0.21	0.002
Cholesterol esters to total lipids ratio in small LDL (%)	1722	0.16	0.08	0.24	<0.0001	1722	0.17	0.09	0.25	<0.0001
Free cholesterol to total lipids ratio in small LDL (%)	1722	-0.18	-0.26	-0.11	<0.0001	1722	-0.20	-0.27	-0.12	<0.0001
Triglycerides to total lipids ratio in small LDL (%)	1722	0.02	-0.05	0.10	0.534	1722	0.04	-0.04	0.12	0.375
Phospholipids to total lipds ratio in very large HDL (%)	1722	-0.37	-0.44	-0.29	<0.0001	1722	-0.37	-0.45	-0.30	<0.0001
Total cholesterol to total lipids ratio in very large HDL (%)	1722	0.32	0.24	0.39	<0.0001	1722	0.32	0.25	0.40	<0.0001
Cholesterol esters to total lipids ratio in very large HDL (%)	1722	0.31	0.24	0.39	<0.0001	1722	0.32	0.24	0.40	<0.0001
Free cholesterol to total lipids ratio in very large HDL (%)	1722	-0.11	-0.19	-0.03	0.010	1722	-0.09	-0.18	0.00	0.039
Triglycerides to total lipids ratio in very large HDL (%)	1722	0.34	0.25	0.43	<0.0001	1722	0.34	0.24	0.44	<0.0001
Phospholipids to total lipds ratio in large HDL (%)	1722	0.47	0.39	0.55	<0.0001	1722	0.50	0.41	0.58	<0.0001
Total cholesterol to total lipids ratio in large HDL (%)	1722	-0.50	-0.58	-0.42	<0.0001	1722	-0.52	-0.61	-0.44	<0.0001
Cholesterol esters to total lipids ratio in large HDL (%)	1722	-0.51	-0.59	-0.42	<0.0001	1722	-0.53	-0.62	-0.44	<0.0001
Free cholesterol to total lipids ratio in large HDL (%)	1722	-0.36	-0.44	-0.28	<0.0001	1722	-0.37	-0.46	-0.29	<0.0001
Triglycerides to total lipids ratio in large HDL (%)	1722	0.44	0.35	0.52	<0.0001	1722	0.45	0.36	0.55	<0.0001
Phospholipids to total lipds ratio in medium HDL (%)	1722	-0.08	-0.16	-0.01	0.031	1722	-0.08	-0.15	0.00	0.039
Total cholesterol to total lipids ratio in medium HDL (%)	1722	-0.15	-0.22	-0.07	0.0002	1722	-0.16	-0.24	-0.08	<0.0001
Cholesterol esters to total lipids ratio in medium HDL (%)	1722	-0.17	-0.25	-0.10	<0.0001	1722	-0.19	-0.26	-0.11	<0.0001
Free cholesterol to total lipids ratio in medium HDL (%)	1722	0.07	-0.01	0.14	0.092	1722	0.07	-0.01	0.15	0.101
Triglycerides to total lipids ratio in medium HDL (%)	1722	0.45	0.37	0.53	<0.0001	1722	0.47	0.38	0.56	<0.0001
Phospholipids to total lipds ratio in small HDL (%)	1722	0.04	-0.03	0.11	0.268	1722	0.02	-0.05	0.09	0.620
Total cholesterol to total lipids ratio in small HDL (%)	1722	-0.12	-0.18	-0.05	0.001	1722	-0.10	-0.17	-0.03	0.005
Cholesterol esters to total lipids ratio in small HDL (%)	1722	-0.05	-0.12	0.01	0.118	1722	-0.03	-0.11	0.04	0.356
Free cholesterol to total lipids ratio in small HDL (%)	1722	-0.36	-0.43	-0.28	<0.0001	1722	-0.41	-0.49	-0.32	<0.0001
Triglycerides to total lipids ratio in small HDL (%)	1722	0.29	0.21	0.38	<0.0001	1722	0.31	0.22	0.41	<0.0001
Mean diameter for VLDL particles (nm)	1722	0.40	0.33	0.48	<0.0001	1722	0.42	0.34	0.50	<0.0001
Mean diameter for LDL particles (nm)	1722	-0.16	-0.23	-0.09	<0.0001	1722	-0.17	-0.24	-0.09	<0.0001
Mean diameter for HDL particles (nm)	1722	-0.37	-0.44	-0.30	<0.0001	1722	-0.38	-0.45	-0.31	<0.0001
Serum total cholesterol (mmol/l)	1722	0.18	0.10	0.25	<0.0001	1722	0.20	0.12	0.28	<0.0001
Total cholesterol in VLDL (mmol/l)	1722	0.49	0.40	0.57	<0.0001	1722	0.52	0.42	0.61	<0.0001
Remnant cholesterol (non-HDL, non-LDL -cholesterol) (mmol/l)	1722	0.41	0.33	0.49	<0.0001	1722	0.44	0.35	0.53	<0.0001
Total cholesterol in LDL (mmol/l)	1722	0.22	0.14	0.30	<0.0001	1722	0.24	0.15	0.33	<0.0001
Total cholesterol in HDL (mmol/l)	1722	-0.30	-0.37	-0.23	<0.0001	1722	-0.31	-0.38	-0.24	<0.0001
Total cholesterol in HDL2 (mmol/l)	1722	-0.35	-0.41	-0.28	<0.0001	1722	-0.36	-0.43	-0.29	<0.0001
Total cholesterol in HDL3 (mmol/l)	1722	-0.20	-0.27	-0.14	<0.0001	1722	-0.21	-0.28	-0.14	<0.0001
Esterified cholesterol (mmol/l)	1722	0.18	0.10	0.25	<0.0001	1722	0.20	0.11	0.28	<0.0001
Free cholesterol (mmol/l)	1722	0.17	0.09	0.24	<0.0001	1722	0.18	0.10	0.26	<0.0001
Serum total triglycerides (mmol/l)	1722	0.40	0.32	0.49	<0.0001	1722	0.43	0.34	0.52	<0.0001
Triglycerides in VLDL (mmol/l)	1722	0.45	0.36	0.53	<0.0001	1722	0.47	0.38	0.56	<0.0001
Triglycerides in LDL (mmol/l)	1722	0.04	-0.03	0.11	0.245	1722	0.06	-0.02	0.13	0.146
Triglycerides in HDL (mmol/l)	1722	0.25	0.18	0.33	<0.0001	1722	0.25	0.17	0.34	<0.0001
Diacylglycerol (mmol/l)	1722	0.33	0.25	0.41	<0.0001	1722	0.33	0.24	0.42	<0.0001
Ratio of diacylglycerol to triglycerides	1722	0.14	0.07	0.21	0.0001	1722	0.12	0.04	0.19	0.003
Total phosphoglycerides (mmol/l)	1722	0.00	-0.06	0.07	0.927	1722	0.00	-0.08	0.07	0.930
Ratio of triglycerides to phosphoglycerides	1722	0.41	0.33	0.49	<0.0001	1722	0.44	0.35	0.53	<0.0001
Phosphatidylcholine and other cholines (mmol/l)	1722	0.00	-0.06	0.07	0.973	1722	-0.02	-0.09	0.05	0.631
Total cholines (mmol/l)	1722	0.01	-0.06	0.08	0.802	1722	0.00	-0.07	0.08	0.968
Apolipoprotein A-I (g/l)	1722	-0.15	-0.21	-0.08	<0.0001	1722	-0.15	-0.22	-0.08	<0.0001
Apolipoprotein B (g/l)	1722	0.42	0.34	0.50	<0.0001	1722	0.45	0.36	0.54	<0.0001
Ratio of apolipoprotein B to apolipoprotein A-I	1722	0.48	0.40	0.56	<0.0001	1722	0.52	0.43	0.61	<0.0001

Online Table 6 Associations of change in body mass index (BMI) and fat mass index from age 10y-18y with cardiometabolic traits at age 18y in ALSPAC

Change from age 10-18y

	BMI (per SD-unit gain) *Adj. for age, sex, ethnicity, maternal education, BMI at 10y*					Fat mass index (per SD-unit gain) *Adj. for age, sex, ethnicity, maternal education, fat mass index at 10y*				
**Standardized outcome at age 18y**	**N**	**Beta**	**LCL**	**UCL**	**P-value**	**N**	**Beta**	**LCL**	**UCL**	**P-value**
Total fatty acids (mmol/l)	1722	0.24	0.17	0.32	<0.0001	1722	0.26	0.18	0.35	<0.0001
Estimated description of fatty acid chain length, not actual carbon number	1722	0.02	-0.06	0.09	0.681	1722	0.03	-0.05	0.11	0.410
Estimated degree of unsaturation	1722	-0.09	-0.16	-0.02	0.017	1722	-0.09	-0.17	-0.02	0.016
22:6, docosahexaenoic acid (mmol/l)	1722	0.08	0.01	0.15	0.031	1722	0.08	0.01	0.16	0.031
18:2, linoleic acid (mmol/l)	1722	0.12	0.05	0.20	0.001	1722	0.12	0.04	0.20	0.002
Conjugated linoleic acid (mmol/l)	1722	0.14	0.07	0.21	0.0001	1722	0.18	0.10	0.25	<0.0001
Omega-3 fatty acids (mmol/l)	1722	0.18	0.11	0.26	<0.0001	1722	0.19	0.10	0.27	<0.0001
Omega-6 fatty acids (mmol/l)	1722	0.15	0.08	0.23	<0.0001	1722	0.16	0.08	0.24	<0.0001
Polyunsaturated fatty acids (mmol/l)	1722	0.17	0.09	0.24	<0.0001	1722	0.17	0.09	0.25	<0.0001
Monounsaturated fatty acids; 16:1, 18:1 (mmol/l)	1722	0.29	0.22	0.37	<0.0001	1722	0.34	0.25	0.42	<0.0001
Saturated fatty acids (mmol/l)	1722	0.22	0.14	0.30	<0.0001	1722	0.22	0.14	0.31	<0.0001
Ratio of 22:6 docosahexaenoic acid to total fatty acids (%)	1722	-0.06	-0.13	0.01	0.102	1722	-0.06	-0.14	0.01	0.103
Ratio of 18:2 linoleic acid to total fatty acids (%)	1722	-0.22	-0.30	-0.15	<0.0001	1722	-0.26	-0.34	-0.18	<0.0001
Ratio of conjugated linoleic acid to total fatty acids (%)	1722	0.10	0.04	0.17	0.002	1722	0.13	0.07	0.20	0.0001
Ratio of omega-3 fatty acids to total fatty acids (%)	1722	0.01	-0.06	0.08	0.795	1722	0.00	-0.08	0.08	0.960
Ratio of omega-6 fatty acids to total fatty acids (%)	1722	-0.21	-0.29	-0.14	<0.0001	1722	-0.24	-0.32	-0.16	<0.0001
Ratio of polyunsaturated fatty acids to total fatty acids (%)	1722	-0.20	-0.28	-0.12	<0.0001	1722	-0.23	-0.31	-0.15	<0.0001
Ratio of monounsaturated fatty acids to total fatty acids (%)	1722	0.22	0.14	0.30	<0.0001	1722	0.29	0.21	0.37	<0.0001
Ratio of saturated fatty acids to total fatty acids (%)	1722	-0.06	-0.13	0.01	0.120	1722	-0.11	-0.19	-0.04	0.004
Insulin (mu/l)	1722	0.42	0.24	0.60	<0.0001	1722	0.44	0.25	0.62	<0.0001
Glucose (mmol/l)	1722	0.14	0.08	0.20	<0.0001	1722	0.15	0.09	0.21	<0.0001
Lactate (mmol/l)	1722	0.00	-0.07	0.08	0.973	1722	0.02	-0.06	0.10	0.610
Pyruvate (mmol/l)	1722	0.11	0.03	0.18	0.006	1722	0.14	0.06	0.22	0.0004
Citrate (mmol/l)	1722	-0.17	-0.24	-0.10	<0.0001	1722	-0.15	-0.23	-0.07	0.0002
Alanine (mmol/l)	1722	0.13	0.05	0.20	0.001	1722	0.12	0.04	0.20	0.003
Glutamine (mmol/l)	1722	-0.04	-0.11	0.03	0.259	1722	-0.03	-0.10	0.05	0.512
Histidine (mmol/l)	1722	0.05	-0.03	0.13	0.247	1722	0.03	-0.06	0.12	0.503
Isoleucine (mmol/l)	1722	0.30	0.23	0.37	<0.0001	1722	0.29	0.22	0.37	<0.0001
Leucine (mmol/l)	1722	0.23	0.17	0.30	<0.0001	1722	0.19	0.12	0.26	<0.0001
Valine (mmol/l)	1722	0.27	0.20	0.33	<0.0001	1722	0.24	0.17	0.31	<0.0001
Phenylalanine (mmol/l)	1722	0.26	0.19	0.33	<0.0001	1722	0.24	0.17	0.32	<0.0001
Tyrosine (mmol/l)	1722	0.35	0.27	0.42	<0.0001	1722	0.36	0.28	0.44	<0.0001
Acetate (mmol/l)	1722	-0.04	-0.08	-0.01	0.006	1722	-0.04	-0.07	-0.01	0.008
Acetoacetate (mmol/l)	1722	-0.13	-0.20	-0.06	0.001	1722	-0.13	-0.20	-0.05	0.001
3-hydroxybutyrate (mmol/l)	1722	-0.14	-0.22	-0.06	0.001	1722	-0.13	-0.21	-0.04	0.003
Creatinine (mmol/l)	1722	0.14	0.08	0.20	<0.0001	1722	0.04	-0.02	0.11	0.202
Albumin (signal area)	1722	-0.06	-0.13	0.01	0.115	1722	-0.06	-0.13	0.01	0.113
Glycoprotein acetyls, mainly a1-acid glycoprotein (mmol/l)	1722	0.39	0.32	0.47	<0.0001	1722	0.44	0.36	0.52	<0.0001
C-reactive protein (mg/l)	1722	0.10	0.01	0.20	0.034	1722	0.12	0.02	0.22	0.017

Online Table 7 Associations of change in regional fat indexes from age 10-18y with cardiometabolic traits at age 18y in ALSPAC

	Change from age 10-18y															
	**Trunk fat index (per SD-unit gain)**					**Arm fat index (per SD-unit gain)**					**Leg fat index (per SD-unit gain)**					
	Adj. for age, sex, ethnicity, maternal education, trunk fat at 10y, arm fat index change, leg fat index change					*Adj. for age, sex, ethnicity, maternal education, arm fat at 10y, trunk fat index change, leg fat index change*					*Adj. for age, sex, ethnicity, maternal education, leg fat at 10y, arm fat index change, trunk fat index change*					***P-value for regional heterogeneity***
**Standardized outcome at age 18y**	**N**	**Beta**	**LCL**	**UCL**	**P-value**	**N**	**Beta**	**LCL**	**UCL**	**P-value**	**N**	**Beta**	**LCL**	**UCL**	**P-value**	
Systolic blood pressure (mmHg)	3409	0.31	0.21	0.41	<0.0001	3409	0.07	-0.05	0.20	0.241	3409	0.11	-0.02	0.23	0.086	<0.0001
Diastolic blood pressure (mmHg)	3409	0.34	0.22	0.47	<0.0001	3409	0.18	0.04	0.31	0.011	3409	0.15	0.02	0.29	0.023	<0.0001
Concentration of chylomicrons and extremely large VLDL particles (mol/l)	2342	0.48	0.31	0.64	<0.0001	2342	0.11	-0.11	0.33	0.323	2342	0.16	-0.06	0.37	0.148	<0.0001
Total lipids in chylomicrons and extremely large VLDL (mmol/l)	2342	0.47	0.31	0.63	<0.0001	2342	0.11	-0.11	0.33	0.341	2342	0.15	-0.06	0.36	0.156	<0.0001
Phospholipids in chylomicrons and extremely large VLDL (mmol/l)	2342	0.46	0.30	0.63	<0.0001	2342	0.11	-0.11	0.33	0.327	2342	0.15	-0.06	0.36	0.162	<0.0001
Total cholesterol in chylomicrons and extremely large VLDL (mmol/l)	2342	0.47	0.31	0.64	<0.0001	2342	0.08	-0.13	0.30	0.449	2342	0.17	-0.05	0.38	0.126	<0.0001
Cholesterol esters in chylomicrons and extremely large VLDL (mmol/l)	2342	0.47	0.30	0.63	<0.0001	2342	0.07	-0.15	0.28	0.539	2342	0.17	-0.04	0.38	0.108	<0.0001
Free cholesterol in chylomicrons and extremely large VLDL (mmol/l)	2342	0.46	0.30	0.63	<0.0001	2342	0.10	-0.12	0.32	0.367	2342	0.15	-0.06	0.36	0.158	<0.0001
Triglycerides in chylomicrons and extremely large VLDL (mmol/l)	2342	0.47	0.31	0.63	<0.0001	2342	0.11	-0.11	0.33	0.323	2342	0.15	-0.06	0.36	0.164	<0.0001
Concentration of very large VLDL particles (mol/l)	2342	0.48	0.31	0.64	<0.0001	2342	0.07	-0.15	0.29	0.517	2342	0.16	-0.05	0.37	0.142	<0.0001
Total lipids in very large VLDL (mmol/l)	2342	0.48	0.31	0.64	<0.0001	2342	0.07	-0.15	0.29	0.552	2342	0.16	-0.05	0.37	0.134	<0.0001
Phospholipids in very large VLDL (mmol/l)	2342	0.47	0.30	0.63	<0.0001	2342	0.08	-0.14	0.30	0.488	2342	0.16	-0.05	0.37	0.141	<0.0001
Total cholesterol in very large VLDL (mmol/l)	2342	0.48	0.32	0.64	<0.0001	2342	0.09	-0.13	0.31	0.430	2342	0.17	-0.04	0.38	0.108	<0.0001
Cholesterol esters in very large VLDL (mmol/l)	2342	0.49	0.33	0.65	<0.0001	2342	0.08	-0.14	0.29	0.474	2342	0.18	-0.03	0.39	0.091	<0.0001
Free cholesterol in very large VLDL (mmol/l)	2342	0.47	0.30	0.63	<0.0001	2342	0.10	-0.12	0.32	0.384	2342	0.16	-0.05	0.37	0.132	<0.0001
Triglycerides in very large VLDL (mmol/l)	2342	0.47	0.31	0.64	<0.0001	2342	0.06	-0.16	0.27	0.615	2342	0.16	-0.05	0.37	0.143	<0.0001
Concentration of large VLDL particles (mol/l)	2342	0.48	0.31	0.64	<0.0001	2342	0.02	-0.19	0.24	0.823	2342	0.18	-0.03	0.38	0.093	<0.0001
Total lipids in large VLDL (mmol/l)	2342	0.48	0.31	0.65	<0.0001	2342	0.03	-0.19	0.24	0.805	2342	0.18	-0.03	0.39	0.088	<0.0001
Phospholipids in large VLDL (mmol/l)	2342	0.47	0.31	0.64	<0.0001	2342	0.03	-0.18	0.24	0.777	2342	0.18	-0.03	0.39	0.085	<0.0001
Total cholesterol in large VLDL (mmol/l)	2342	0.48	0.31	0.65	<0.0001	2342	0.04	-0.17	0.25	0.727	2342	0.19	-0.02	0.39	0.073	<0.0001
Cholesterol esters in large VLDL (mmol/l)	2342	0.48	0.32	0.65	<0.0001	2342	0.03	-0.17	0.24	0.750	2342	0.20	0.00	0.40	0.053	<0.0001
Free cholesterol in large VLDL (mmol/l)	2342	0.47	0.30	0.64	<0.0001	2342	0.04	-0.17	0.26	0.707	2342	0.17	-0.03	0.38	0.101	<0.0001
Triglycerides in large VLDL (mmol/l)	2342	0.48	0.31	0.64	<0.0001	2342	0.02	-0.19	0.24	0.845	2342	0.18	-0.03	0.38	0.096	<0.0001
Concentration of medium VLDL particles (mol/l)	2342	0.48	0.31	0.64	<0.0001	2342	0.01	-0.19	0.22	0.892	2342	0.21	0.01	0.41	0.044	<0.0001
Total lipids in medium VLDL (mmol/l)	2342	0.48	0.32	0.64	<0.0001	2342	0.02	-0.19	0.22	0.876	2342	0.21	0.01	0.41	0.037	<0.0001
Phospholipids in medium VLDL (mmol/l)	2342	0.46	0.30	0.63	<0.0001	2342	0.02	-0.19	0.22	0.881	2342	0.21	0.01	0.41	0.037	<0.0001
Total cholesterol in medium VLDL (mmol/l)	2342	0.45	0.29	0.61	<0.0001	2342	0.02	-0.18	0.23	0.822	2342	0.22	0.03	0.41	0.023	<0.0001
Cholesterol esters in medium VLDL (mmol/l)	2342	0.43	0.27	0.59	<0.0001	2342	0.03	-0.17	0.23	0.765	2342	0.23	0.04	0.41	0.017	<0.0001
Free cholesterol in medium VLDL (mmol/l)	2342	0.45	0.29	0.62	<0.0001	2342	0.01	-0.20	0.22	0.899	2342	0.20	0.01	0.40	0.042	<0.0001
Triglycerides in medium VLDL (mmol/l)	2342	0.48	0.32	0.65	<0.0001	2342	0.01	-0.20	0.22	0.907	2342	0.20	0.00	0.40	0.051	<0.0001
Concentration of small VLDL particles (mol/l)	2342	0.43	0.27	0.59	<0.0001	2342	-0.01	-0.21	0.19	0.944	2342	0.23	0.04	0.41	0.016	<0.0001
Total lipids in small VLDL (mmol/l)	2342	0.42	0.26	0.57	<0.0001	2342	0.01	-0.18	0.21	0.892	2342	0.24	0.06	0.42	0.010	<0.0001
Phospholipids in small VLDL (mmol/l)	2342	0.40	0.25	0.56	<0.0001	2342	-0.02	-0.21	0.17	0.835	2342	0.23	0.05	0.41	0.011	<0.0001
Total cholesterol in small VLDL (mmol/l)	2342	0.34	0.19	0.49	<0.0001	2342	0.08	-0.11	0.27	0.420	2342	0.24	0.06	0.42	0.008	0.001
Cholesterol esters in small VLDL (mmol/l)	2342	0.31	0.16	0.45	<0.0001	2342	0.12	-0.07	0.31	0.232	2342	0.23	0.06	0.41	0.010	0.003
Free cholesterol in small VLDL (mmol/l)	2342	0.38	0.22	0.53	<0.0001	2342	0.00	-0.20	0.19	0.981	2342	0.23	0.05	0.40	0.013	0.0002
Triglycerides in small VLDL (mmol/l)	2342	0.44	0.28	0.60	<0.0001	2342	-0.03	-0.24	0.17	0.770	2342	0.21	0.02	0.40	0.029	<0.0001
Concentration of very small VLDL particles (mol/l)	2342	0.17	0.03	0.32	0.021	2342	0.05	-0.14	0.24	0.582	2342	0.18	0.01	0.35	0.042	0.169
Total lipids in very small VLDL (mmol/l)	2342	0.18	0.03	0.33	0.016	2342	0.10	-0.09	0.29	0.307	2342	0.19	0.01	0.36	0.036	0.153
Phospholipids in very small VLDL (mmol/l)	2342	0.11	-0.04	0.25	0.155	2342	0.09	-0.10	0.28	0.341	2342	0.15	-0.02	0.32	0.076	0.576
Total cholesterol in very small VLDL (mmol/l)	2342	0.13	-0.02	0.29	0.083	2342	0.15	-0.04	0.35	0.127	2342	0.16	-0.01	0.34	0.073	0.367
Cholesterol esters in very small VLDL (mmol/l)	2342	0.17	0.02	0.32	0.025	2342	0.16	-0.03	0.36	0.098	2342	0.17	-0.01	0.34	0.064	0.163
Free cholesterol in very small VLDL (mmol/l)	2342	0.03	-0.12	0.18	0.706	2342	0.11	-0.09	0.30	0.289	2342	0.14	-0.04	0.32	0.118	0.993
Triglycerides in very small VLDL (mmol/l)	2342	0.29	0.13	0.45	0.0003	2342	-0.06	-0.25	0.14	0.579	2342	0.19	0.02	0.36	0.029	0.006
Concentration of IDL particles (mol/l)	2342	0.06	-0.09	0.20	0.456	2342	0.11	-0.08	0.30	0.251	2342	0.10	-0.06	0.27	0.224	0.859
Total lipids in IDL (mmol/l)	2342	0.06	-0.08	0.21	0.391	2342	0.12	-0.07	0.31	0.199	2342	0.12	-0.05	0.28	0.178	0.801
Phospholipids in IDL (mmol/l)	2342	0.03	-0.12	0.18	0.694	2342	0.12	-0.06	0.31	0.198	2342	0.09	-0.07	0.26	0.269	0.906
Total cholesterol in IDL (mmol/l)	2342	0.08	-0.07	0.22	0.315	2342	0.15	-0.04	0.34	0.133	2342	0.12	-0.05	0.29	0.167	0.687
Cholesterol esters in IDL (mmol/l)	2342	0.11	-0.03	0.26	0.132	2342	0.15	-0.04	0.34	0.126	2342	0.14	-0.04	0.31	0.119	0.446
Free cholesterol in IDL (mmol/l)	2342	-0.02	-0.17	0.13	0.797	2342	0.13	-0.06	0.32	0.166	2342	0.08	-0.09	0.25	0.361	0.813
Triglycerides in IDL (mmol/l)	2342	0.05	-0.10	0.20	0.515	2342	-0.04	-0.22	0.15	0.683	2342	0.09	-0.07	0.24	0.279	0.709
Concentration of large LDL particles (mol/l)	2342	0.07	-0.08	0.22	0.347	2342	0.12	-0.07	0.30	0.209	2342	0.10	-0.06	0.27	0.216	0.713

Online Table 7 Associations of change in regional fat indexes from age 10-18y with cardiometabolic traits at age 18y in ALSPAC

	Change from age 10-18y															
	**Trunk fat index (per SD-unit gain)**					**Arm fat index (per SD-unit gain)**					**Leg fat index (per SD-unit gain)**					
	Adj. for age, sex, ethnicity, maternal education, trunk fat at 10y, arm fat index change, leg fat index change					*Adj. for age, sex, ethnicity, maternal education, arm fat at 10y, trunk fat index change, leg fat index change*					*Adj. for age, sex, ethnicity, maternal education, leg fat at 10y, arm fat index change, trunk fat index change*					
**Standardized outcome at age 18y**	**N**	**Beta**	**LCL**	**UCL**	**P-value**	**N**	**Beta**	**LCL**	**UCL**	**P-value**	**N**	**Beta**	**LCL**	**UCL**	**P-value**	***P-value for regional heterogeneity***
Total lipids in large LDL (mmol/l)	2342	0.07	-0.08	0.22	0.336	2342	0.13	-0.06	0.31	0.188	2342	0.11	-0.06	0.27	0.197	0.702
Phospholipids in large LDL (mmol/l)	2342	0.10	-0.05	0.25	0.185	2342	0.13	-0.06	0.32	0.176	2342	0.12	-0.04	0.29	0.153	0.519
Total cholesterol in large LDL (mmol/l)	2342	0.07	-0.08	0.22	0.350	2342	0.14	-0.05	0.33	0.144	2342	0.11	-0.06	0.28	0.195	0.684
Cholesterol esters in large LDL (mmol/l)	2342	0.09	-0.06	0.24	0.237	2342	0.14	-0.05	0.33	0.142	2342	0.12	-0.05	0.28	0.164	0.571
Free cholesterol in large LDL (mmol/l)	2342	0.01	-0.14	0.16	0.877	2342	0.14	-0.05	0.33	0.154	2342	0.08	-0.08	0.25	0.324	0.864
Triglycerides in large LDL (mmol/l)	2342	0.01	-0.14	0.17	0.866	2342	-0.02	-0.20	0.16	0.829	2342	0.04	-0.11	0.19	0.599	0.938
Concentration of medium LDL particles (mol/l)	2342	0.11	-0.04	0.26	0.151	2342	0.11	-0.08	0.30	0.246	2342	0.12	-0.05	0.28	0.157	0.477
Total lipids in medium LDL (mmol/l)	2342	0.09	-0.05	0.24	0.211	2342	0.12	-0.06	0.31	0.193	2342	0.12	-0.05	0.28	0.163	0.560
Phospholipids in medium LDL (mmol/l)	2342	0.16	0.01	0.31	0.032	2342	0.13	-0.06	0.32	0.169	2342	0.13	-0.03	0.30	0.114	0.168
Total cholesterol in medium LDL (mmol/l)	2342	0.08	-0.07	0.23	0.273	2342	0.14	-0.05	0.33	0.157	2342	0.12	-0.05	0.28	0.166	0.626
Cholesterol esters in medium LDL (mmol/l)	2342	0.09	-0.06	0.23	0.263	2342	0.13	-0.06	0.32	0.169	2342	0.12	-0.04	0.29	0.146	0.637
Free cholesterol in medium LDL (mmol/l)	2342	0.07	-0.07	0.22	0.325	2342	0.15	-0.04	0.34	0.128	2342	0.09	-0.07	0.26	0.278	0.574
Triglycerides in medium LDL (mmol/l)	2342	0.02	-0.14	0.17	0.849	2342	-0.02	-0.20	0.16	0.827	2342	0.04	-0.11	0.19	0.610	0.945
Concentration of small LDL particles (mol/l)	2342	0.11	-0.04	0.26	0.156	2342	0.13	-0.06	0.31	0.190	2342	0.10	-0.06	0.26	0.227	0.408
Total lipids in small LDL (mmol/l)	2342	0.10	-0.05	0.25	0.191	2342	0.14	-0.05	0.32	0.158	2342	0.11	-0.05	0.28	0.173	0.491
Phospholipids in small LDL (mmol/l)	2342	0.14	-0.01	0.29	0.072	2342	0.14	-0.05	0.33	0.138	2342	0.11	-0.06	0.27	0.207	0.220
Total cholesterol in small LDL (mmol/l)	2342	0.07	-0.07	0.22	0.324	2342	0.15	-0.04	0.34	0.130	2342	0.11	-0.05	0.28	0.185	0.642
Cholesterol esters in small LDL (mmol/l)	2342	0.08	-0.07	0.23	0.321	2342	0.14	-0.05	0.33	0.142	2342	0.12	-0.05	0.28	0.167	0.665
Free cholesterol in small LDL (mmol/l)	2342	0.07	-0.08	0.22	0.373	2342	0.16	-0.03	0.36	0.100	2342	0.09	-0.08	0.26	0.301	0.563
Triglycerides in small LDL (mmol/l)	2342	0.17	0.00	0.33	0.045	2342	-0.01	-0.20	0.17	0.877	2342	0.11	-0.05	0.26	0.186	0.207
Concentration of very large HDL particles (mol/l)	2342	-0.35	-0.50	-0.20	<0.0001	2342	0.13	-0.05	0.30	0.147	2342	-0.22	-0.36	-0.07	0.003	<0.0001
Total lipids in very large HDL (mmol/l)	2342	-0.35	-0.50	-0.20	<0.0001	2342	0.16	-0.02	0.33	0.081	2342	-0.23	-0.37	-0.08	0.003	<0.0001
Phospholipids in very large HDL (mmol/l)	2342	-0.37	-0.51	-0.22	<0.0001	2342	0.09	-0.08	0.26	0.300	2342	-0.21	-0.36	-0.07	0.004	<0.0001
Total cholesterol in very large HDL (mmol/l)	2342	-0.31	-0.46	-0.16	<0.0001	2342	0.21	0.03	0.39	0.021	2342	-0.22	-0.38	-0.07	0.004	<0.0001
Cholesterol esters in very large HDL (mmol/l)	2342	-0.30	-0.45	-0.14	0.0001	2342	0.23	0.04	0.41	0.015	2342	-0.21	-0.37	-0.06	0.007	0.0001
Free cholesterol in very large HDL (mmol/l)	2342	-0.33	-0.49	-0.18	<0.0001	2342	0.16	-0.02	0.34	0.075	2342	-0.24	-0.39	-0.09	0.002	<0.0001
Triglycerides in very large HDL (mmol/l)	2342	-0.02	-0.18	0.14	0.843	2342	0.06	-0.13	0.26	0.538	2342	-0.04	-0.21	0.13	0.662	0.805
Concentration of large HDL particles (mol/l)	2342	-0.36	-0.51	-0.22	<0.0001	2342	0.02	-0.15	0.20	0.813	2342	-0.19	-0.35	-0.04	0.012	<0.0001
Total lipids in large HDL (mmol/l)	2342	-0.36	-0.51	-0.22	<0.0001	2342	0.02	-0.15	0.20	0.793	2342	-0.20	-0.35	-0.05	0.011	<0.0001
Phospholipids in large HDL (mmol/l)	2342	-0.33	-0.47	-0.18	<0.0001	2342	0.02	-0.15	0.20	0.791	2342	-0.17	-0.32	-0.02	0.026	0.0003
Total cholesterol in large HDL (mmol/l)	2342	-0.39	-0.54	-0.25	<0.0001	2342	0.03	-0.15	0.20	0.779	2342	-0.22	-0.38	-0.07	0.005	<0.0001
Cholesterol esters in large HDL (mmol/l)	2342	-0.39	-0.54	-0.25	<0.0001	2342	0.02	-0.16	0.20	0.815	2342	-0.22	-0.38	-0.07	0.005	<0.0001
Free cholesterol in large HDL (mmol/l)	2342	-0.39	-0.53	-0.24	<0.0001	2342	0.04	-0.14	0.22	0.667	2342	-0.22	-0.37	-0.06	0.006	<0.0001
Triglycerides in large HDL (mmol/l)	2342	-0.07	-0.22	0.09	0.395	2342	-0.03	-0.21	0.15	0.731	2342	0.04	-0.10	0.19	0.542	0.597
Concentration of medium HDL particles (mol/l)	2342	-0.03	-0.19	0.13	0.726	2342	-0.04	-0.21	0.14	0.661	2342	-0.02	-0.19	0.15	0.855	0.989
Total lipids in medium HDL (mmol/l)	2342	-0.06	-0.21	0.10	0.451	2342	-0.03	-0.21	0.15	0.756	2342	-0.03	-0.20	0.14	0.713	0.866
Phospholipids in medium HDL (mmol/l)	2342	-0.07	-0.22	0.09	0.400	2342	-0.03	-0.20	0.14	0.745	2342	-0.04	-0.21	0.13	0.663	0.807
Total cholesterol in medium HDL (mmol/l)	2342	-0.11	-0.26	0.04	0.148	2342	-0.01	-0.20	0.17	0.884	2342	-0.06	-0.23	0.12	0.509	0.509
Cholesterol esters in medium HDL (mmol/l)	2342	-0.12	-0.27	0.03	0.124	2342	-0.02	-0.20	0.17	0.851	2342	-0.07	-0.24	0.11	0.453	0.469
Free cholesterol in medium HDL (mmol/l)	2342	-0.07	-0.23	0.08	0.356	2342	0.00	-0.18	0.18	0.985	2342	-0.02	-0.18	0.15	0.858	0.773
Triglycerides in medium HDL (mmol/l)	2342	0.39	0.22	0.56	<0.0001	2342	-0.09	-0.28	0.10	0.347	2342	0.22	0.05	0.40	0.012	0.0002
Concentration of small HDL particles (mol/l)	2342	0.18	0.02	0.35	0.032	2342	-0.08	-0.26	0.10	0.388	2342	0.08	-0.10	0.26	0.395	0.075
Total lipids in small HDL (mmol/l)	2342	0.09	-0.07	0.25	0.271	2342	-0.08	-0.26	0.10	0.386	2342	0.06	-0.12	0.23	0.525	0.419
Phospholipids in small HDL (mmol/l)	2342	0.15	-0.01	0.32	0.071	2342	-0.09	-0.28	0.10	0.361	2342	0.04	-0.15	0.22	0.692	0.137
Total cholesterol in small HDL (mmol/l)	2342	-0.04	-0.19	0.10	0.568	2342	-0.04	-0.21	0.13	0.643	2342	0.04	-0.12	0.21	0.610	0.756
Cholesterol esters in small HDL (mmol/l)	2342	-0.05	-0.19	0.10	0.533	2342	-0.02	-0.19	0.15	0.788	2342	0.06	-0.10	0.22	0.464	0.696
Free cholesterol in small HDL (mmol/l)	2342	-0.02	-0.18	0.14	0.789	2342	-0.08	-0.27	0.10	0.382	2342	-0.03	-0.21	0.15	0.707	0.964
Triglycerides in small HDL (mmol/l)	2342	0.33	0.16	0.50	0.0001	2342	-0.12	-0.32	0.09	0.268	2342	0.17	-0.01	0.35	0.065	0.002
Phospholipids to total lipds ratio in chylomicrons and extremely large VLDL (%)	2342	0.01	-0.08	0.09	0.882	2342	0.06	-0.04	0.17	0.246	2342	-0.04	-0.19	0.11	0.631	0.535
Total cholesterol to total lipids ratio in chylomicrons and extremely large VLDL (%)	2342	0.22	0.07	0.36	0.004	2342	-0.10	-0.28	0.09	0.301	2342	0.14	-0.01	0.28	0.067	0.028
Cholesterol esters to total lipids ratio in chylomicrons and extremely large VLDL (%)	2342	0.17	0.03	0.31	0.019	2342	-0.11	-0.30	0.08	0.247	2342	0.12	-0.02	0.26	0.098	0.100
Free cholesterol to total lipids ratio in chylomicrons and extremely large VLDL (%)	2342	0.17	0.03	0.31	0.014	2342	0.02	-0.16	0.20	0.823	2342	0.12	-0.02	0.27	0.099	0.039

Online Table 7 Associations of change in regional fat indexes from age 10-18y with cardiometabolic traits at age 18y in ALSPAC

Change from age 10-18y

	Trunk fat index (per SD-unit gain) *Adj. for age, sex, ethnicity, maternal education, trunk fat at 10y, arm fat index change, leg fat index change*					Arm fat index (per SD-unit gain) *Adj. for age, sex, ethnicity, maternal education, arm fat at 10y, trunk fat index change, leg fat index change*					Leg fat index (per SD-unit gain) *Adj. for age, sex, ethnicity, maternal education, leg fat at 10y, arm fat index change, trunk fat index change*					
**Standardized outcome at age 18y**	**N**	**Beta**	**LCL**	**UCL**	**P-value**	**N**	**Beta**	**LCL**	**UCL**	**P-value**	**N**	**Beta**	**LCL**	**UCL**	**P-value**	***P-value for regional heterogeneity***
Triglycerides to total lipids ratio in chylomicrons and extremely large VLDL (%)	2342	-0.15	-0.30	0.00	0.044	2342	0.02	-0.14	0.19	0.772	2342	-0.10	-0.27	0.06	0.206	0.186
Phospholipids to total lipds ratio in very large VLDL (%)	2342	0.20	0.06	0.34	0.004	2342	0.19	0.02	0.36	0.026	2342	0.04	-0.12	0.20	0.641	0.005
Total cholesterol to total lipids ratio in very large VLDL (%)	2342	-0.10	-0.28	0.08	0.284	2342	0.08	-0.12	0.29	0.436	2342	-0.13	-0.31	0.06	0.187	0.339
Cholesterol esters to total lipids ratio in very large VLDL (%)	2342	-0.10	-0.26	0.06	0.205	2342	0.19	-0.21	0.59	0.360	2342	-0.22	-0.56	0.11	0.188	0.372
Free cholesterol to total lipids ratio in very large VLDL (%)	2342	-0.14	-0.27	-0.01	0.037	2342	0.34	-0.06	0.73	0.094	2342	-0.25	-0.58	0.08	0.139	0.110
Triglycerides to total lipids ratio in very large VLDL (%)	2342	0.08	-0.06	0.22	0.260	2342	-0.14	-0.34	0.05	0.148	2342	0.04	-0.10	0.19	0.583	0.300
Phospholipids to total lipds ratio in large VLDL (%)	2342	0.22	0.09	0.35	0.001	2342	-0.03	-0.21	0.14	0.734	2342	0.15	0.01	0.29	0.034	0.009
Total cholesterol to total lipids ratio in large VLDL (%)	2342	0.20	0.06	0.33	0.005	2342	0.00	-0.18	0.19	0.980	2342	0.15	0.01	0.29	0.039	0.058
Cholesterol esters to total lipids ratio in large VLDL (%)	2342	0.06	-0.07	0.19	0.351	2342	0.01	-0.18	0.20	0.898	2342	0.04	-0.09	0.16	0.581	0.805
Free cholesterol to total lipids ratio in large VLDL (%)	2342	0.08	-0.03	0.19	0.157	2342	0.00	-0.08	0.08	0.976	2342	0.13	0.00	0.26	0.044	0.052
Triglycerides to total lipids ratio in large VLDL (%)	2342	-0.20	-0.34	-0.06	0.006	2342	-0.01	-0.19	0.18	0.955	2342	-0.17	-0.33	0.00	0.047	0.055
Phospholipids to total lipds ratio in medium VLDL (%)	2342	-0.31	-0.46	-0.15	0.0001	2342	0.03	-0.16	0.23	0.754	2342	-0.12	-0.27	0.03	0.112	0.001
Total cholesterol to total lipids ratio in medium VLDL (%)	2342	0.10	-0.05	0.24	0.203	2342	0.01	-0.17	0.19	0.913	2342	0.09	-0.07	0.24	0.275	0.580
Cholesterol esters to total lipids ratio in medium VLDL (%)	2342	0.07	-0.08	0.21	0.375	2342	0.02	-0.16	0.20	0.828	2342	0.07	-0.08	0.23	0.358	0.831
Free cholesterol to total lipids ratio in medium VLDL (%)	2342	0.14	-0.01	0.29	0.070	2342	-0.03	-0.22	0.16	0.750	2342	0.09	-0.07	0.24	0.265	0.196
Triglycerides to total lipids ratio in medium VLDL (%)	2342	-0.02	-0.17	0.12	0.740	2342	-0.03	-0.21	0.15	0.773	2342	-0.05	-0.20	0.10	0.535	0.981
Phospholipids to total lipds ratio in small VLDL (%)	2342	-0.27	-0.42	-0.12	0.0003	2342	-0.12	-0.32	0.07	0.212	2342	-0.15	-0.32	0.03	0.108	0.006
Total cholesterol to total lipids ratio in small VLDL (%)	2342	-0.12	-0.27	0.03	0.109	2342	0.19	0.00	0.38	0.054	2342	0.00	-0.17	0.16	0.958	0.143
Cholesterol esters to total lipids ratio in small VLDL (%)	2342	-0.09	-0.24	0.06	0.255	2342	0.21	0.02	0.40	0.033	2342	0.01	-0.16	0.18	0.915	0.238
Free cholesterol to total lipids ratio in small VLDL (%)	2342	-0.24	-0.39	-0.10	0.001	2342	-0.10	-0.28	0.09	0.307	2342	-0.11	-0.27	0.05	0.185	0.008
Triglycerides to total lipids ratio in small VLDL (%)	2342	0.21	0.06	0.36	0.005	2342	-0.14	-0.34	0.05	0.148	2342	0.05	-0.12	0.23	0.555	0.017
Phospholipids to total lipds ratio in very small VLDL (%)	2342	-0.05	-0.20	0.10	0.497	2342	0.03	-0.15	0.20	0.747	2342	0.00	-0.15	0.14	0.994	0.777
Total cholesterol to total lipids ratio in very small VLDL (%)	2342	-0.13	-0.30	0.04	0.131	2342	0.16	-0.03	0.36	0.101	2342	-0.06	-0.22	0.09	0.419	0.201
Cholesterol esters to total lipids ratio in very small VLDL (%)	2342	-0.03	-0.18	0.13	0.747	2342	0.16	-0.03	0.35	0.095	2342	-0.02	-0.17	0.13	0.798	0.475
Free cholesterol to total lipids ratio in very small VLDL (%)	2342	-0.39	-0.57	-0.21	<0.0001	2342	0.05	-0.14	0.24	0.611	2342	-0.15	-0.32	0.03	0.104	0.0005
Triglycerides to total lipids ratio in very small VLDL (%)	2342	0.19	0.03	0.36	0.023	2342	-0.21	-0.41	-0.01	0.039	2342	0.07	-0.10	0.24	0.423	0.045
Phospholipids to total lipds ratio in IDL (%)	2342	-0.28	-0.45	-0.12	0.001	2342	-0.03	-0.21	0.16	0.782	2342	-0.16	-0.34	0.02	0.076	0.008
Total cholesterol to total lipids ratio in IDL (%)	2342	0.09	-0.07	0.24	0.279	2342	0.20	0.01	0.39	0.042	2342	0.04	-0.12	0.20	0.583	0.215
Cholesterol esters to total lipids ratio in IDL (%)	2342	0.23	0.08	0.39	0.003	2342	0.15	-0.03	0.33	0.106	2342	0.11	-0.05	0.27	0.175	0.011
Free cholesterol to total lipids ratio in IDL (%)	2342	-0.35	-0.50	-0.19	<0.0001	2342	0.13	-0.07	0.32	0.208	2342	-0.15	-0.34	0.05	0.140	0.0002
Triglycerides to total lipids ratio in IDL (%)	2342	0.01	-0.14	0.17	0.853	2342	-0.22	-0.41	-0.02	0.029	2342	0.00	-0.16	0.17	0.954	0.303
Phospholipids to total lipds ratio in large LDL (%)	2342	-0.03	-0.17	0.11	0.649	2342	-0.07	-0.25	0.11	0.423	2342	-0.04	-0.19	0.12	0.644	0.854
Total cholesterol to total lipids ratio in large LDL (%)	2342	0.05	-0.09	0.19	0.472	2342	0.18	-0.01	0.36	0.060	2342	0.05	-0.11	0.22	0.518	0.455
Cholesterol esters to total lipids ratio in large LDL (%)	2342	0.15	0.01	0.29	0.030	2342	0.15	-0.03	0.33	0.103	2342	0.10	-0.06	0.25	0.224	0.101
Free cholesterol to total lipids ratio in large LDL (%)	2342	-0.33	-0.48	-0.18	<0.0001	2342	0.01	-0.18	0.20	0.937	2342	-0.16	-0.32	0.01	0.066	0.001
Triglycerides to total lipids ratio in large LDL (%)	2342	-0.05	-0.20	0.10	0.544	2342	-0.20	-0.40	-0.01	0.041	2342	-0.05	-0.22	0.12	0.543	0.388
Phospholipids to total lipds ratio in medium LDL (%)	2342	-0.02	-0.15	0.12	0.831	2342	-0.07	-0.25	0.11	0.431	2342	-0.04	-0.19	0.12	0.651	0.933
Total cholesterol to total lipids ratio in medium LDL (%)	2342	0.03	-0.10	0.17	0.635	2342	0.14	-0.04	0.32	0.118	2342	0.04	-0.12	0.20	0.623	0.629
Cholesterol esters to total lipids ratio in medium LDL (%)	2342	0.09	-0.05	0.23	0.222	2342	0.11	-0.07	0.29	0.236	2342	0.07	-0.08	0.23	0.357	0.469
Free cholesterol to total lipids ratio in medium LDL (%)	2342	-0.14	-0.29	0.00	0.049	2342	-0.05	-0.23	0.13	0.577	2342	-0.10	-0.25	0.04	0.171	0.215
Triglycerides to total lipids ratio in medium LDL (%)	2342	-0.04	-0.18	0.10	0.554	2342	-0.17	-0.33	0.00	0.055	2342	-0.03	-0.17	0.12	0.725	0.382
Phospholipids to total lipds ratio in small LDL (%)	2342	-0.06	-0.20	0.07	0.374	2342	-0.09	-0.27	0.09	0.311	2342	-0.07	-0.22	0.09	0.380	0.703
Total cholesterol to total lipids ratio in small LDL (%)	2342	0.00	-0.14	0.13	0.953	2342	0.15	-0.03	0.33	0.108	2342	0.04	-0.13	0.21	0.645	0.710
Cholesterol esters to total lipids ratio in small LDL (%)	2342	0.06	-0.08	0.19	0.437	2342	0.12	-0.06	0.30	0.182	2342	0.07	-0.09	0.23	0.403	0.653
Free cholesterol to total lipids ratio in small LDL (%)	2342	-0.14	-0.29	0.00	0.056	2342	-0.05	-0.23	0.13	0.581	2342	-0.10	-0.25	0.04	0.167	0.225
Triglycerides to total lipids ratio in small LDL (%)	2342	0.18	0.02	0.34	0.028	2342	-0.19	-0.39	0.00	0.055	2342	0.08	-0.10	0.25	0.381	0.057
Phospholipids to total lipds ratio in very large HDL (%)	2342	-0.34	-0.49	-0.19	<0.0001	2342	-0.06	-0.23	0.11	0.479	2342	-0.12	-0.28	0.03	0.123	0.0001
Total cholesterol to total lipids ratio in very large HDL (%)	2342	0.29	0.15	0.44	<0.0001	2342	0.08	-0.09	0.24	0.355	2342	0.09	-0.05	0.24	0.208	0.001
Cholesterol esters to total lipids ratio in very large HDL (%)	2342	0.29	0.14	0.43	0.0001	2342	0.06	-0.11	0.23	0.475	2342	0.11	-0.04	0.26	0.146	0.001
Free cholesterol to total lipids ratio in very large HDL (%)	2342	-0.08	-0.24	0.08	0.327	2342	0.15	-0.05	0.34	0.137	2342	-0.18	-0.35	-0.01	0.043	0.097
Triglycerides to total lipids ratio in very large HDL (%)	2342	0.34	0.16	0.51	0.0002	2342	-0.07	-0.29	0.14	0.494	2342	0.19	-0.02	0.40	0.082	0.003
Phospholipids to total lipds ratio in large HDL (%)	2342	0.48	0.32	0.64	<0.0001	2342	0.02	-0.19	0.22	0.886	2342	0.26	0.07	0.45	0.009	<0.0001

Online Table 7 Associations of change in regional fat indexes from age 10-18y with cardiometabolic traits at age 18y in ALSPAC

Change from age 10-18y

	**Trunk fat index (per SD-unit gain)**					**Arm fat index (per SD-unit gain)**					**Leg fat index (per SD-unit gain)**					
	Adj. for age, sex, ethnicity, maternal education, trunk fat at 10y, arm fat index change, leg fat index change					*Adj. for age, sex, ethnicity, maternal education, arm fat at 10y, trunk fat index change, leg fat index change*					*Adj. for age, sex, ethnicity, maternal education, leg fat at 10y, arm fat index change, trunk fat index change*					***P-value for regional heterogeneity***
**Standardized outcome at age 18y**	**N**	**Beta**	**LCL**	**UCL**	**P-value**	**N**	**Beta**	**LCL**	**UCL**	**P-value**	**N**	**Beta**	**LCL**	**UCL**	**P-value**	
Total cholesterol to total lipids ratio in large HDL (%)	2342	-0.50	-0.66	-0.34	<0.0001	2342	0.00	-0.20	0.21	0.981	2342	-0.28	-0.48	-0.08	0.005	<0.0001
Cholesterol esters to total lipids ratio in large HDL (%)	2342	-0.48	-0.64	-0.32	<0.0001	2342	-0.02	-0.23	0.18	0.817	2342	-0.28	-0.48	-0.08	0.005	<0.0001
Free cholesterol to total lipids ratio in large HDL (%)	2342	-0.45	-0.61	-0.28	<0.0001	2342	0.09	-0.11	0.29	0.383	2342	-0.19	-0.38	-0.01	0.041	<0.0001
Triglycerides to total lipids ratio in large HDL (%)	2342	0.40	0.24	0.56	<0.0001	2342	-0.02	-0.22	0.17	0.814	2342	0.25	0.06	0.44	0.011	0.0001
Phospholipids to total lipds ratio in medium HDL (%)	2342	-0.03	-0.18	0.11	0.649	2342	0.01	-0.17	0.19	0.906	2342	-0.09	-0.25	0.07	0.261	0.750
Total cholesterol to total lipids ratio in medium HDL (%)	2342	-0.20	-0.34	-0.05	0.009	2342	0.02	-0.17	0.21	0.865	2342	-0.04	-0.21	0.13	0.672	0.070
Cholesterol esters to total lipids ratio in medium HDL (%)	2342	-0.20	-0.34	-0.05	0.009	2342	0.00	-0.19	0.19	0.988	2342	-0.06	-0.22	0.11	0.520	0.073
Free cholesterol to total lipids ratio in medium HDL (%)	2342	-0.06	-0.20	0.07	0.346	2342	0.08	-0.08	0.25	0.322	2342	0.09	-0.07	0.24	0.267	0.643
Triglycerides to total lipids ratio in medium HDL (%)	2342	0.46	0.30	0.62	<0.0001	2342	-0.05	-0.25	0.15	0.601	2342	0.24	0.05	0.43	0.011	<0.0001
Phospholipids to total lipds ratio in small HDL (%)	2342	0.11	-0.03	0.24	0.129	2342	-0.02	-0.19	0.15	0.802	2342	-0.03	-0.18	0.12	0.686	0.238
Total cholesterol to total lipids ratio in small HDL (%)	2342	-0.19	-0.33	-0.05	0.007	2342	0.03	-0.14	0.21	0.722	2342	-0.01	-0.17	0.16	0.946	0.023
Cholesterol esters to total lipids ratio in small HDL (%)	2342	-0.13	-0.27	0.00	0.059	2342	0.04	-0.14	0.21	0.666	2342	0.03	-0.13	0.19	0.716	0.131
Free cholesterol to total lipids ratio in small HDL (%)	2342	-0.30	-0.45	-0.16	<0.0001	2342	-0.05	-0.23	0.14	0.626	2342	-0.24	-0.41	-0.07	0.005	0.003
Triglycerides to total lipids ratio in small HDL (%)	2342	0.33	0.17	0.49	<0.0001	2342	-0.06	-0.29	0.17	0.605	2342	0.15	-0.06	0.35	0.168	0.002
Mean diameter for VLDL particles (nm)	2342	0.44	0.29	0.60	<0.0001	2342	-0.01	-0.21	0.18	0.907	2342	0.17	-0.01	0.35	0.063	<0.0001
Mean diameter for LDL particles (nm)	2342	-0.18	-0.32	-0.04	0.013	2342	-0.09	-0.27	0.09	0.304	2342	-0.04	-0.20	0.11	0.576	0.027
Mean diameter for HDL particles (nm)	2342	-0.41	-0.56	-0.27	<0.0001	2342	0.09	-0.08	0.27	0.289	2342	-0.24	-0.39	-0.08	0.002	<0.0001
Serum total cholesterol (mmol/l)	2342	0.05	-0.10	0.20	0.523	2342	0.14	-0.04	0.33	0.123	2342	0.08	-0.08	0.24	0.309	0.668
Total cholesterol in VLDL (mmol/l)	2342	0.40	0.24	0.55	<0.0001	2342	0.09	-0.11	0.28	0.387	2342	0.23	0.04	0.41	0.015	0.0001
Remnant cholesterol (non-HDL, non-LDL -cholesterol) (mmol/l)	2342	0.28	0.13	0.43	0.0003	2342	0.12	-0.07	0.32	0.215	2342	0.20	0.02	0.38	0.027	0.011
Total cholesterol in LDL (mmol/l)	2342	0.08	-0.07	0.22	0.319	2342	0.14	-0.05	0.33	0.146	2342	0.11	-0.05	0.28	0.181	0.661
Total cholesterol in HDL (mmol/l)	2342	-0.30	-0.45	-0.16	<0.0001	2342	0.06	-0.11	0.24	0.493	2342	-0.17	-0.32	-0.01	0.035	0.001
Total cholesterol in HDL2 (mmol/l)	2342	-0.34	-0.49	-0.20	<0.0001	2342	0.06	-0.12	0.24	0.533	2342	-0.19	-0.35	-0.03	0.023	0.0001
Total cholesterol in HDL3 (mmol/l)	2342	-0.21	-0.36	-0.07	0.005	2342	0.07	-0.11	0.24	0.451	2342	-0.12	-0.27	0.02	0.098	0.026
Esterified cholesterol (mmol/l)	2332	0.05	-0.10	0.20	0.523	2332	0.17	-0.02	0.35	0.077	2332	0.06	-0.10	0.23	0.452	0.532
Free cholesterol (mmol/l)	2330	0.04	-0.11	0.19	0.576	2330	0.08	-0.10	0.26	0.378	2330	0.14	-0.02	0.30	0.077	0.931
Serum total triglycerides (mmol/l)	2342	0.43	0.26	0.59	<0.0001	2342	0.00	-0.21	0.21	0.988	2342	0.19	-0.01	0.38	0.058	<0.0001
Triglycerides in VLDL (mmol/l)	2342	0.47	0.31	0.64	<0.0001	2342	0.01	-0.20	0.22	0.926	2342	0.20	-0.01	0.40	0.057	<0.0001
Triglycerides in LDL (mmol/l)	2342	0.04	-0.12	0.20	0.605	2342	-0.02	-0.20	0.16	0.835	2342	0.05	-0.10	0.20	0.498	0.877
Triglycerides in HDL (mmol/l)	2342	0.27	0.10	0.44	0.002	2342	-0.07	-0.27	0.12	0.463	2342	0.16	-0.02	0.33	0.080	0.016
Diacylglycerol (mmol/l)	2276	0.26	0.11	0.42	0.001	2276	0.08	-0.14	0.30	0.469	2276	0.09	-0.11	0.29	0.357	0.005
Ratio of diacylglycerol to triglycerides	2277	0.08	-0.06	0.23	0.268	2277	0.03	-0.16	0.22	0.759	2277	0.05	-0.11	0.21	0.530	0.385
Total phosphoglycerides (mmol/l)	2330	-0.03	-0.19	0.12	0.675	2330	0.05	-0.13	0.23	0.595	2330	0.01	-0.14	0.16	0.934	0.867
Ratio of triglycerides to phosphoglycerides	2330	0.43	0.27	0.58	<0.0001	2330	-0.03	-0.24	0.18	0.769	2330	0.22	0.00	0.43	0.048	<0.0001
Phosphatidylcholine and other cholines (mmol/l)	2314	-0.01	-0.17	0.14	0.855	2314	0.01	-0.16	0.19	0.884	2314	0.00	-0.15	0.14	0.955	0.955
Total cholines (mmol/l)	2332	-0.03	-0.19	0.12	0.691	2332	0.03	-0.14	0.21	0.715	2332	0.02	-0.13	0.17	0.772	0.903
Apolipoprotein A-I (g/l)	2342	-0.17	-0.32	-0.02	0.023	2342	0.08	-0.09	0.25	0.368	2342	-0.09	-0.23	0.06	0.251	0.082
Apolipoprotein B (g/l)	2342	0.32	0.17	0.47	<0.0001	2342	0.09	-0.11	0.28	0.394	2342	0.21	0.03	0.39	0.021	0.003
Ratio of apolipoprotein B to apolipoprotein A-I	2342	0.41	0.25	0.56	<0.0001	2342	0.05	-0.15	0.25	0.599	2342	0.24	0.06	0.43	0.010	<0.0001
Total fatty acids (mmol/l)	2332	0.21	0.05	0.37	0.010	2332	0.05	-0.14	0.24	0.615	2332	0.13	-0.04	0.29	0.142	0.080
Estimated description of fatty acid chain length, not actual carbon number	2333	0.02	-0.13	0.17	0.807	2333	-0.04	-0.23	0.15	0.691	2333	0.06	-0.10	0.22	0.472	0.832
Estimated degree of unsaturation	2332	-0.20	-0.36	-0.05	0.011	2332	0.10	-0.09	0.29	0.296	2332	0.00	-0.16	0.16	0.996	0.054
22:6, docosahexaenoic acid (mmol/l)	2332	-0.08	-0.24	0.07	0.291	2332	0.20	0.01	0.38	0.038	2332	0.05	-0.11	0.20	0.559	0.312
18:2, linoleic acid (mmol/l)	2332	0.07	-0.08	0.22	0.345	2332	0.01	-0.17	0.19	0.872	2332	0.09	-0.07	0.25	0.254	0.723
Conjugated linoleic acid (mmol/l)	2331	0.08	-0.07	0.23	0.289	2331	0.19	0.00	0.39	0.051	2331	-0.07	-0.24	0.09	0.374	0.081
Omega-3 fatty acids (mmol/l)	2332	0.04	-0.12	0.21	0.595	2332	0.22	0.03	0.42	0.027	2332	0.03	-0.13	0.19	0.727	0.280
Omega-6 fatty acids (mmol/l)	2332	0.09	-0.07	0.24	0.267	2332	0.06	-0.13	0.24	0.537	2332	0.10	-0.06	0.26	0.215	0.659
Polyunsaturated fatty acids (mmol/l)	2332	0.08	-0.07	0.24	0.286	2332	0.08	-0.10	0.27	0.378	2332	0.09	-0.06	0.25	0.242	0.652
Monounsaturated fatty acids; 16:1, 18:1 (mmol/l)	2332	0.33	0.17	0.48	<0.0001	2332	-0.03	-0.22	0.17	0.788	2332	0.18	0.00	0.36	0.045	0.002
Saturated fatty acids (mmol/l)	2331	0.17	0.00	0.34	0.044	2331	0.08	-0.12	0.28	0.441	2331	0.07	-0.10	0.25	0.391	0.170
Ratio of 22:6 docosahexaenoic acid to total fatty acids (%)	2333	-0.25	-0.39	-0.11	0.001	2333	0.25	0.07	0.43	0.006	2333	-0.04	-0.19	0.10	0.576	0.001
Ratio of 18:2 linoleic acid to total fatty acids (%)	2333	-0.27	-0.41	-0.12	0.0004	2333	-0.05	-0.24	0.14	0.601	2333	-0.06	-0.23	0.10	0.444	0.003

Online Table 7 Associations of change in regional fat indexes from age 10-18y with cardiometabolic traits at age 18y in ALSPAC

Change from age 10-18y

Standardized outcome at age 18y	Trunk fat index (per SD-unit gain)<br>*Adj. for age, sex, ethnicity, maternal education, trunk fat at 10y, arm fat index change, leg fat index change*					Arm fat index (per SD-unit gain)<br>*Adj. for age, sex, ethnicity, maternal education, arm fat at 10y, trunk fat index change, leg fat index change*					Leg fat index (per SD-unit gain)<br>*Adj. for age, sex, ethnicity, maternal education, leg fat at 10y, arm fat index change, trunk fat index change*					*P-value for regional heterogeneity*
	N	Beta	LCL	UCL	P-value	N	Beta	LCL	UCL	P-value	N	Beta	LCL	UCL	P-value	
Ratio of conjugated linoleic acid to total fatty acids (%)	2332	0.02	-0.12	0.16	0.758	2332	0.19	0.00	0.38	0.053	2332	-0.07	-0.23	0.09	0.376	0.174
Ratio of omega-3 fatty acids to total fatty acids (%)	2333	-0.15	-0.30	-0.01	0.042	2333	0.27	0.07	0.46	0.007	2333	-0.09	-0.25	0.06	0.251	0.023
Ratio of omega-6 fatty acids to total fatty acids (%)	2333	-0.30	-0.45	-0.15	0.0001	2333	0.03	-0.16	0.22	0.765	2333	-0.07	-0.25	0.10	0.409	0.001
Ratio of polyunsaturated fatty acids to total fatty acids (%)	2333	-0.32	-0.47	-0.17	<0.0001	2333	0.09	-0.10	0.28	0.347	2333	-0.09	-0.26	0.08	0.302	0.0004
Ratio of monounsaturated fatty acids to total fatty acids (%)	2333	0.37	0.22	0.52	<0.0001	2333	-0.15	-0.34	0.04	0.121	2333	0.18	0.01	0.35	0.033	<0.0001
Ratio of saturated fatty acids to total fatty acids (%)	2332	-0.12	-0.28	0.04	0.146	2332	0.09	-0.10	0.29	0.353	2332	-0.14	-0.31	0.02	0.096	0.252
Insulin (mu/l)	2378	0.51	-0.09	1.10	0.099	2378	-0.04	-0.52	0.45	0.886	2378	0.27	-0.11	0.64	0.164	0.313
Glucose (mmol/l)	2341	0.12	-0.01	0.25	0.062	2341	0.04	-0.15	0.23	0.691	2341	0.08	-0.05	0.21	0.225	0.253
Lactate (mmol/l)	2341	-0.06	-0.24	0.11	0.473	2341	0.17	-0.02	0.36	0.075	2341	-0.02	-0.20	0.15	0.803	0.394
Pyruvate (mmol/l)	2341	0.17	0.02	0.32	0.029	2341	0.18	-0.01	0.37	0.057	2341	-0.05	-0.21	0.11	0.523	0.007
Citrate (mmol/l)	2341	-0.30	-0.44	-0.15	<0.0001	2341	-0.09	-0.26	0.08	0.289	2341	0.11	-0.04	0.26	0.143	<0.0001
Alanine (mmol/l)	2341	0.19	0.05	0.34	0.010	2341	-0.06	-0.25	0.13	0.532	2341	0.01	-0.15	0.16	0.945	0.048
Glutamine (mmol/l)	2341	-0.10	-0.24	0.03	0.143	2341	0.07	-0.10	0.24	0.426	2341	-0.06	-0.20	0.08	0.417	0.256
Histidine (mmol/l)	2341	0.17	0.02	0.32	0.026	2341	-0.19	-0.37	0.00	0.046	2341	0.06	-0.12	0.23	0.506	0.040
Isoleucine (mmol/l)	2341	0.34	0.15	0.53	0.0004	2341	0.00	-0.17	0.18	0.983	2341	0.07	-0.11	0.25	0.451	0.006
Leucine (mmol/l)	2341	0.23	0.08	0.38	0.002	2341	-0.07	-0.23	0.09	0.386	2341	0.11	-0.05	0.27	0.167	0.014
Valine (mmol/l)	2341	0.20	0.05	0.34	0.009	2341	0.03	-0.14	0.19	0.756	2341	0.20	0.04	0.35	0.011	0.046
Phenylalanine (mmol/l)	2341	0.38	0.19	0.56	<0.0001	2341	-0.12	-0.29	0.05	0.161	2341	0.12	-0.06	0.30	0.183	0.001
Tyrosine (mmol/l)	2341	0.35	0.19	0.50	<0.0001	2341	0.07	-0.11	0.25	0.477	2341	0.14	-0.03	0.30	0.102	0.001
Acetate (mmol/l)	2340	-0.06	-0.17	0.05	0.281	2340	0.11	-0.14	0.35	0.391	2340	-0.21	-0.45	0.02	0.071	0.337
Acetoacetate (mmol/l)	2341	-0.13	-0.30	0.03	0.108	2341	0.11	-0.09	0.31	0.290	2341	-0.10	-0.25	0.06	0.215	0.289
3-hydroxybutyrate (mmol/l)	2338	-0.09	-0.29	0.10	0.357	2338	0.06	-0.16	0.28	0.588	2338	-0.10	-0.25	0.05	0.190	0.578
Creatinine (mmol/l)	2341	0.21	0.09	0.33	0.001	2341	-0.22	-0.37	-0.07	0.005	2341	0.03	-0.10	0.17	0.632	0.002
Albumin (signal area)	2342	-0.02	-0.16	0.12	0.772	2342	0.02	-0.16	0.19	0.843	2342	-0.17	-0.33	-0.02	0.029	0.529
Glycoprotein acetyls, mainly a1-acid glycoprotein (mmol/l)	2341	0.40	0.24	0.56	<0.0001	2341	0.11	-0.09	0.30	0.275	2341	0.20	0.01	0.38	0.038	<0.0001
C-reactive protein (mg/l)	2419	0.15	-0.02	0.32	0.075	2419	0.03	-0.12	0.18	0.677	2419	0.08	-0.07	0.23	0.315	0.220

Change from age 10-18y

Complete case sample

Standardized outcome at age 18y	Trunk fat index (per SD-unit gain)<br>*Adj. for age, sex, ethnicity, maternal education, trunk fat at 10y, arm fat index change, leg fat index change*					Arm fat index (per SD-unit gain)<br>*Adj. for age, sex, ethnicity, maternal education, arm fat at 10y, trunk fat index change, leg fat index change*					Leg fat index (per SD-unit gain)<br>*Adj. for age, sex, ethnicity, maternal education, leg fat at 10y, arm fat index change, trunk fat index change*					*P-value for regional heterogeneity*
	N	Beta	LCL	UCL	P-value	N	Beta	LCL	UCL	P-value	N	Beta	LCL	UCL	P-value	
Systolic blood pressure (mmHg)	1722	0.32	0.17	0.47	<0.0001	1722	0.08	-0.10	0.26	0.384	1722	0.06	-0.11	0.24	0.455	<0.0001
Diastolic blood pressure (mmHg)	1722	0.30	0.12	0.48	0.001	1722	0.24	0.03	0.45	0.028	1722	0.13	-0.07	0.32	0.199	0.001
Concentration of chylomicrons and extremely large VLDL particles (mol/l)	1722	0.46	0.28	0.64	<0.0001	1722	0.16	-0.10	0.43	0.231	1722	0.11	-0.12	0.35	0.333	<0.0001
Total lipids in chylomicrons and extremely large VLDL (mmol/l)	1722	0.46	0.28	0.64	<0.0001	1722	0.16	-0.10	0.43	0.231	1722	0.10	-0.12	0.33	0.373	<0.0001
Phospholipids in chylomicrons and extremely large VLDL (mmol/l)	1722	0.45	0.27	0.64	<0.0001	1722	0.16	-0.10	0.43	0.233	1722	0.10	-0.13	0.32	0.397	<0.0001
Total cholesterol in chylomicrons and extremely large VLDL (mmol/l)	1722	0.46	0.27	0.64	<0.0001	1722	0.14	-0.12	0.40	0.300	1722	0.13	-0.10	0.36	0.254	<0.0001
Cholesterol esters in chylomicrons and extremely large VLDL (mmol/l)	1722	0.44	0.26	0.63	<0.0001	1722	0.12	-0.14	0.38	0.364	1722	0.16	-0.07	0.39	0.178	0.0002
Free cholesterol in chylomicrons and extremely large VLDL (mmol/l)	1722	0.45	0.27	0.64	<0.0001	1722	0.16	-0.11	0.42	0.247	1722	0.10	-0.13	0.33	0.389	<0.0001
Triglycerides in chylomicrons and extremely large VLDL (mmol/l)	1722	0.46	0.28	0.64	<0.0001	1722	0.17	-0.10	0.43	0.219	1722	0.10	-0.13	0.33	0.404	<0.0001
Concentration of very large VLDL particles (mol/l)	1722	0.47	0.28	0.65	<0.0001	1722	0.13	-0.13	0.39	0.323	1722	0.10	-0.12	0.33	0.377	<0.0001
Total lipids in very large VLDL (mmol/l)	1722	0.47	0.28	0.65	<0.0001	1722	0.12	-0.14	0.38	0.352	1722	0.10	-0.12	0.33	0.359	<0.0001
Phospholipids in very large VLDL (mmol/l)	1722	0.46	0.27	0.65	<0.0001	1722	0.13	-0.13	0.39	0.337	1722	0.11	-0.11	0.34	0.330	<0.0001
Total cholesterol in very large VLDL (mmol/l)	1722	0.47	0.28	0.65	<0.0001	1722	0.14	-0.12	0.40	0.291	1722	0.13	-0.09	0.36	0.250	<0.0001

Online Table 7 Associations of change in regional fat indexes from age 10-18y with cardiometabolic traits at age 18y in ALSPAC

Change from age 10-18y

	Trunk fat index (per SD-unit gain) *Adj. for age, sex, ethnicity, maternal education, trunk fat at 10y, arm fat index change, leg fat index change*					Arm fat index (per SD-unit gain) *Adj. for age, sex, ethnicity, maternal education, arm fat at 10y, trunk fat index change, leg fat index change*					Leg fat index (per SD-unit gain) *Adj. for age, sex, ethnicity, maternal education, leg fat at 10y, arm fat index change, trunk fat index change*					
**Standardized outcome at age 18y**	**N**	**Beta**	**LCL**	**UCL**	**P-value**	**N**	**Beta**	**LCL**	**UCL**	**P-value**	**N**	**Beta**	**LCL**	**UCL**	**P-value**	***P-value for regional heterogeneity***
Cholesterol esters in very large VLDL (mmol/l)	1722	0.48	0.29	0.66	<0.0001	1722	0.13	-0.12	0.39	0.306	1722	0.14	-0.08	0.36	0.222	<0.0001
Free cholesterol in very large VLDL (mmol/l)	1722	0.46	0.27	0.64	<0.0001	1722	0.15	-0.12	0.41	0.276	1722	0.12	-0.10	0.35	0.289	<0.0001
Triglycerides in very large VLDL (mmol/l)	1722	0.46	0.28	0.65	<0.0001	1722	0.12	-0.14	0.37	0.381	1722	0.09	-0.13	0.31	0.413	<0.0001
Concentration of large VLDL particles (mol/l)	1722	0.47	0.28	0.66	<0.0001	1722	0.08	-0.17	0.32	0.547	1722	0.11	-0.10	0.33	0.295	<0.0001
Total lipids in large VLDL (mmol/l)	1722	0.47	0.28	0.66	<0.0001	1722	0.08	-0.17	0.33	0.538	1722	0.12	-0.09	0.33	0.272	<0.0001
Phospholipids in large VLDL (mmol/l)	1722	0.47	0.28	0.66	<0.0001	1722	0.07	-0.17	0.32	0.553	1722	0.12	-0.09	0.34	0.249	<0.0001
Total cholesterol in large VLDL (mmol/l)	1722	0.47	0.29	0.66	<0.0001	1722	0.08	-0.16	0.33	0.513	1722	0.14	-0.07	0.35	0.193	<0.0001
Cholesterol esters in large VLDL (mmol/l)	1722	0.48	0.29	0.67	<0.0001	1722	0.08	-0.16	0.32	0.524	1722	0.16	-0.05	0.37	0.139	<0.0001
Free cholesterol in large VLDL (mmol/l)	1722	0.47	0.28	0.65	<0.0001	1722	0.08	-0.17	0.34	0.508	1722	0.12	-0.09	0.34	0.264	<0.0001
Triglycerides in large VLDL (mmol/l)	1722	0.47	0.28	0.66	<0.0001	1722	0.08	-0.17	0.32	0.546	1722	0.11	-0.10	0.32	0.319	<0.0001
Concentration of medium VLDL particles (mol/l)	1722	0.48	0.29	0.67	<0.0001	1722	0.06	-0.18	0.30	0.631	1722	0.15	-0.06	0.35	0.166	<0.0001
Total lipids in medium VLDL (mmol/l)	1722	0.48	0.29	0.67	<0.0001	1722	0.06	-0.18	0.30	0.630	1722	0.16	-0.05	0.36	0.133	<0.0001
Phospholipids in medium VLDL (mmol/l)	1722	0.47	0.28	0.66	<0.0001	1722	0.05	-0.19	0.29	0.682	1722	0.16	-0.04	0.37	0.118	<0.0001
Total cholesterol in medium VLDL (mmol/l)	1722	0.45	0.26	0.64	<0.0001	1722	0.05	-0.19	0.28	0.695	1722	0.21	0.00	0.41	0.048	0.0001
Cholesterol esters in medium VLDL (mmol/l)	1722	0.43	0.24	0.62	<0.0001	1722	0.04	-0.19	0.27	0.711	1722	0.24	0.03	0.45	0.023	0.0005
Free cholesterol in medium VLDL (mmol/l)	1722	0.46	0.27	0.65	<0.0001	1722	0.05	-0.19	0.29	0.693	1722	0.16	-0.05	0.36	0.135	<0.0001
Triglycerides in medium VLDL (mmol/l)	1722	0.48	0.29	0.67	<0.0001	1722	0.07	-0.17	0.31	0.588	1722	0.13	-0.08	0.33	0.230	<0.0001
Concentration of small VLDL particles (mol/l)	1722	0.44	0.26	0.63	<0.0001	1722	0.01	-0.22	0.25	0.905	1722	0.20	-0.01	0.40	0.058	0.0001
Total lipids in small VLDL (mmol/l)	1722	0.43	0.25	0.61	<0.0001	1722	0.03	-0.21	0.26	0.828	1722	0.23	0.02	0.43	0.032	0.0002
Phospholipids in small VLDL (mmol/l)	1722	0.41	0.23	0.59	<0.0001	1722	-0.02	-0.25	0.21	0.862	1722	0.23	0.02	0.43	0.028	0.0003
Total cholesterol in small VLDL (mmol/l)	1722	0.35	0.18	0.53	<0.0001	1722	0.06	-0.17	0.29	0.588	1722	0.28	0.06	0.49	0.012	0.003
Cholesterol esters in small VLDL (mmol/l)	1722	0.32	0.14	0.50	0.0004	1722	0.10	-0.14	0.33	0.418	1722	0.28	0.06	0.50	0.012	0.010
Free cholesterol in small VLDL (mmol/l)	1722	0.39	0.20	0.57	<0.0001	1722	-0.01	-0.24	0.22	0.945	1722	0.24	0.03	0.44	0.024	0.001
Triglycerides in small VLDL (mmol/l)	1722	0.45	0.26	0.64	<0.0001	1722	0.01	-0.23	0.24	0.946	1722	0.15	-0.05	0.35	0.139	0.0001
Concentration of very small VLDL particles (mol/l)	1722	0.19	0.02	0.37	0.028	1722	-0.01	-0.23	0.21	0.933	1722	0.27	0.06	0.48	0.010	0.116
Total lipids in very small VLDL (mmol/l)	1722	0.20	0.02	0.37	0.027	1722	0.05	-0.18	0.27	0.699	1722	0.28	0.06	0.50	0.011	0.151
Phospholipids in very small VLDL (mmol/l)	1722	0.14	-0.04	0.32	0.117	1722	0.00	-0.22	0.22	0.990	1722	0.27	0.07	0.48	0.010	0.285
Total cholesterol in very small VLDL (mmol/l)	1722	0.14	-0.04	0.32	0.139	1722	0.11	-0.13	0.35	0.377	1722	0.26	0.04	0.49	0.020	0.498
Cholesterol esters in very small VLDL (mmol/l)	1722	0.17	-0.01	0.35	0.064	1722	0.14	-0.10	0.38	0.262	1722	0.25	0.03	0.47	0.027	0.325
Free cholesterol in very small VLDL (mmol/l)	1722	0.04	-0.14	0.23	0.660	1722	0.03	-0.20	0.26	0.809	1722	0.27	0.06	0.49	0.014	0.589
Triglycerides in very small VLDL (mmol/l)	1722	0.31	0.13	0.49	0.001	1722	-0.06	-0.29	0.16	0.585	1722	0.19	-0.01	0.38	0.057	0.009
Concentration of IDL particles (mol/l)	1722	0.09	-0.09	0.27	0.323	1722	0.03	-0.19	0.25	0.796	1722	0.22	0.02	0.42	0.034	0.613
Total lipids in IDL (mmol/l)	1722	0.10	-0.08	0.27	0.291	1722	0.03	-0.19	0.25	0.795	1722	0.25	0.04	0.45	0.019	0.551
Phospholipids in IDL (mmol/l)	1722	0.06	-0.11	0.24	0.481	1722	0.02	-0.19	0.24	0.825	1722	0.22	0.02	0.42	0.030	0.670
Total cholesterol in IDL (mmol/l)	1722	0.10	-0.07	0.28	0.253	1722	0.05	-0.18	0.28	0.656	1722	0.26	0.05	0.47	0.017	0.570
Cholesterol esters in IDL (mmol/l)	1722	0.14	-0.04	0.32	0.128	1722	0.06	-0.17	0.29	0.601	1722	0.27	0.06	0.48	0.013	0.419
Free cholesterol in IDL (mmol/l)	1722	0.01	-0.16	0.19	0.872	1722	0.03	-0.19	0.25	0.807	1722	0.22	0.02	0.43	0.033	0.687
Triglycerides in IDL (mmol/l)	1722	0.08	-0.10	0.26	0.390	1722	-0.09	-0.30	0.13	0.428	1722	0.14	-0.05	0.32	0.146	0.440
Concentration of large LDL particles (mol/l)	1722	0.11	-0.07	0.29	0.229	1722	0.02	-0.19	0.24	0.841	1722	0.22	0.02	0.42	0.031	0.503
Total lipids in large LDL (mmol/l)	1722	0.11	-0.07	0.28	0.234	1722	0.02	-0.19	0.24	0.826	1722	0.23	0.03	0.43	0.023	0.501
Phospholipids in large LDL (mmol/l)	1722	0.14	-0.04	0.31	0.131	1722	0.02	-0.20	0.24	0.843	1722	0.25	0.04	0.45	0.017	0.356
Total cholesterol in large LDL (mmol/l)	1722	0.10	-0.07	0.28	0.246	1722	0.04	-0.19	0.26	0.747	1722	0.24	0.04	0.44	0.021	0.536
Cholesterol esters in large LDL (mmol/l)	1722	0.12	-0.05	0.30	0.173	1722	0.04	-0.18	0.26	0.726	1722	0.24	0.04	0.45	0.019	0.452
Free cholesterol in large LDL (mmol/l)	1722	0.05	-0.13	0.23	0.604	1722	0.03	-0.20	0.25	0.814	1722	0.23	0.02	0.43	0.030	0.707
Triglycerides in large LDL (mmol/l)	1722	0.04	-0.14	0.22	0.678	1722	-0.06	-0.27	0.15	0.557	1722	0.09	-0.09	0.27	0.338	0.716
Concentration of medium LDL particles (mol/l)	1722	0.15	-0.03	0.33	0.106	1722	0.02	-0.20	0.24	0.867	1722	0.22	0.02	0.42	0.031	0.316
Total lipids in medium LDL (mmol/l)	1722	0.13	-0.05	0.31	0.148	1722	0.03	-0.19	0.24	0.822	1722	0.23	0.03	0.43	0.024	0.391
Phospholipids in medium LDL (mmol/l)	1722	0.20	0.02	0.37	0.028	1722	0.03	-0.19	0.25	0.808	1722	0.25	0.04	0.45	0.017	0.133
Total cholesterol in medium LDL (mmol/l)	1722	0.12	-0.06	0.30	0.188	1722	0.03	-0.19	0.26	0.759	1722	0.24	0.03	0.44	0.022	0.461
Cholesterol esters in medium LDL (mmol/l)	1722	0.12	-0.06	0.30	0.183	1722	0.03	-0.19	0.26	0.759	1722	0.24	0.04	0.45	0.020	0.453
Free cholesterol in medium LDL (mmol/l)	1722	0.11	-0.07	0.29	0.219	1722	0.03	-0.19	0.26	0.762	1722	0.23	0.02	0.43	0.032	0.512
Triglycerides in medium LDL (mmol/l)	1722	0.04	-0.14	0.23	0.646	1722	-0.06	-0.27	0.14	0.544	1722	0.08	-0.10	0.25	0.394	0.734

Online Table 7 Associations of change in regional fat indexes from age 10-18y with cardiometabolic traits at age 18y in ALSPAC

Change from age 10-18y

	Trunk fat index (per SD-unit gain)					Arm fat index (per SD-unit gain)					Leg fat index (per SD-unit gain)					
	Adj. for age, sex, ethnicity, maternal education, trunk fat at 10y, arm fat index change, leg fat index change					*Adj. for age, sex, ethnicity, maternal education, arm fat at 10y, trunk fat index change, leg fat index change*					*Adj. for age, sex, ethnicity, maternal education, leg fat at 10y, arm fat index change, trunk fat index change*					***P-value for regional heterogeneity***
**Standardized outcome at age 18y**	**N**	**Beta**	**LCL**	**UCL**	**P-value**	**N**	**Beta**	**LCL**	**UCL**	**P-value**	**N**	**Beta**	**LCL**	**UCL**	**P-value**	
Concentration of small LDL particles (mol/l)	1722	0.15	-0.03	0.33	0.112	1722	0.04	-0.18	0.26	0.748	1722	0.20	0.00	0.39	0.055	0.337
Total lipids in small LDL (mmol/l)	1722	0.14	-0.04	0.32	0.134	1722	0.04	-0.18	0.26	0.745	1722	0.23	0.02	0.43	0.029	0.383
Phospholipids in small LDL (mmol/l)	1722	0.17	0.00	0.35	0.055	1722	0.04	-0.18	0.26	0.724	1722	0.21	0.01	0.41	0.038	0.208
Total cholesterol in small LDL (mmol/l)	1722	0.11	-0.07	0.29	0.221	1722	0.05	-0.18	0.27	0.694	1722	0.23	0.03	0.44	0.026	0.522
Cholesterol esters in small LDL (mmol/l)	1722	0.11	-0.07	0.30	0.215	1722	0.04	-0.18	0.27	0.708	1722	0.23	0.03	0.44	0.025	0.513
Free cholesterol in small LDL (mmol/l)	1722	0.10	-0.08	0.28	0.286	1722	0.05	-0.18	0.28	0.655	1722	0.23	0.01	0.44	0.038	0.609
Triglycerides in small LDL (mmol/l)	1722	0.19	0.00	0.38	0.048	1722	-0.04	-0.27	0.18	0.692	1722	0.12	-0.06	0.31	0.187	0.164
Concentration of very large HDL particles (mol/l)	1722	-0.33	-0.52	-0.15	0.0004	1722	0.07	-0.14	0.28	0.496	1722	-0.14	-0.32	0.04	0.121	0.003
Total lipids in very large HDL (mmol/l)	1722	-0.33	-0.52	-0.15	0.0004	1722	0.11	-0.10	0.32	0.315	1722	-0.16	-0.34	0.02	0.089	0.003
Phospholipids in very large HDL (mmol/l)	1722	-0.34	-0.52	-0.16	0.0002	1722	0.02	-0.18	0.23	0.831	1722	-0.13	-0.31	0.04	0.140	0.002
Total cholesterol in very large HDL (mmol/l)	1722	-0.30	-0.49	-0.12	0.002	1722	0.19	-0.03	0.41	0.094	1722	-0.17	-0.36	0.02	0.074	0.005
Cholesterol esters in very large HDL (mmol/l)	1722	-0.29	-0.48	-0.10	0.002	1722	0.21	-0.01	0.44	0.060	1722	-0.17	-0.36	0.02	0.077	0.005
Free cholesterol in very large HDL (mmol/l)	1722	-0.31	-0.50	-0.13	0.001	1722	0.11	-0.10	0.33	0.308	1722	-0.17	-0.35	0.01	0.071	0.005
Triglycerides in very large HDL (mmol/l)	1722	-0.03	-0.21	0.16	0.790	1722	0.09	-0.13	0.31	0.431	1722	0.00	-0.19	0.19	0.986	0.815
Concentration of large HDL particles (mol/l)	1722	-0.35	-0.52	-0.17	<0.0001	1722	-0.07	-0.28	0.14	0.521	1722	-0.11	-0.29	0.08	0.258	0.001
Total lipids in large HDL (mmol/l)	1722	-0.34	-0.52	-0.17	0.0001	1722	-0.07	-0.28	0.14	0.501	1722	-0.11	-0.30	0.07	0.240	0.001
Phospholipids in large HDL (mmol/l)	1722	-0.31	-0.48	-0.13	0.001	1722	-0.08	-0.28	0.13	0.454	1722	-0.08	-0.27	0.10	0.373	0.004
Total cholesterol in large HDL (mmol/l)	1722	-0.37	-0.55	-0.20	<0.0001	1722	-0.06	-0.28	0.15	0.552	1722	-0.14	-0.33	0.05	0.150	0.0004
Cholesterol esters in large HDL (mmol/l)	1722	-0.38	-0.55	-0.20	<0.0001	1722	-0.06	-0.28	0.15	0.556	1722	-0.14	-0.33	0.05	0.140	0.0004
Free cholesterol in large HDL (mmol/l)	1722	-0.36	-0.54	-0.19	<0.0001	1722	-0.07	-0.28	0.15	0.543	1722	-0.12	-0.31	0.07	0.206	0.001
Triglycerides in large HDL (mmol/l)	1722	-0.06	-0.23	0.11	0.500	1722	-0.09	-0.28	0.10	0.346	1722	0.13	-0.03	0.29	0.111	0.207
Concentration of medium HDL particles (mol/l)	1722	-0.03	-0.22	0.16	0.782	1722	-0.13	-0.34	0.08	0.232	1722	0.03	-0.18	0.24	0.748	0.707
Total lipids in medium HDL (mmol/l)	1722	-0.05	-0.24	0.14	0.588	1722	-0.13	-0.34	0.09	0.245	1722	0.02	-0.19	0.23	0.847	0.694
Phospholipids in medium HDL (mmol/l)	1722	-0.07	-0.25	0.12	0.489	1722	-0.11	-0.32	0.10	0.305	1722	0.01	-0.20	0.22	0.918	0.697
Total cholesterol in medium HDL (mmol/l)	1722	-0.09	-0.28	0.09	0.313	1722	-0.13	-0.36	0.09	0.236	1722	0.00	-0.21	0.22	0.972	0.534
Cholesterol esters in medium HDL (mmol/l)	1722	-0.10	-0.28	0.08	0.280	1722	-0.14	-0.36	0.09	0.234	1722	-0.01	-0.23	0.21	0.934	0.530
Free cholesterol in medium HDL (mmol/l)	1722	-0.05	-0.24	0.13	0.573	1722	-0.13	-0.34	0.08	0.235	1722	0.07	-0.14	0.28	0.519	0.535
Triglycerides in medium HDL (mmol/l)	1722	0.38	0.19	0.58	0.0001	1722	-0.07	-0.29	0.15	0.519	1722	0.19	0.00	0.39	0.049	0.001
Concentration of small HDL particles (mol/l)	1722	0.18	-0.02	0.38	0.076	1722	-0.13	-0.35	0.10	0.268	1722	0.06	-0.17	0.28	0.625	0.120
Total lipids in small HDL (mmol/l)	1722	0.10	-0.10	0.29	0.320	1722	-0.15	-0.36	0.06	0.169	1722	0.07	-0.15	0.29	0.521	0.323
Phospholipids in small HDL (mmol/l)	1722	0.14	-0.06	0.35	0.165	1722	-0.11	-0.34	0.12	0.366	1722	-0.01	-0.24	0.22	0.929	0.255
Total cholesterol in small HDL (mmol/l)	1722	-0.02	-0.19	0.16	0.864	1722	-0.16	-0.36	0.04	0.111	1722	0.13	-0.07	0.33	0.213	0.287
Cholesterol esters in small HDL (mmol/l)	1722	-0.01	-0.18	0.16	0.883	1722	-0.16	-0.36	0.04	0.118	1722	0.16	-0.03	0.35	0.095	0.199
Free cholesterol in small HDL (mmol/l)	1722	-0.02	-0.22	0.17	0.816	1722	-0.12	-0.35	0.10	0.291	1722	-0.04	-0.27	0.18	0.704	0.918
Triglycerides in small HDL (mmol/l)	1722	0.32	0.13	0.51	0.001	1722	-0.06	-0.30	0.17	0.598	1722	0.12	-0.08	0.32	0.241	0.009
Phospholipids to total lipds ratio in chylomicrons and extremely large VLDL (%)	1722	0.01	-0.08	0.10	0.820	1722	-0.02	-0.13	0.10	0.780	1722	0.05	-0.05	0.14	0.336	0.767
Total cholesterol to total lipids ratio in chylomicrons and extremely large VLDL (%)	1722	0.25	0.07	0.43	0.006	1722	-0.10	-0.33	0.13	0.400	1722	0.15	-0.03	0.33	0.103	0.042
Cholesterol esters to total lipids ratio in chylomicrons and extremely large VLDL (%)	1722	0.18	0.01	0.36	0.041	1722	-0.09	-0.33	0.15	0.467	1722	0.14	-0.04	0.31	0.130	0.209
Free cholesterol to total lipids ratio in chylomicrons and extremely large VLDL (%)	1722	0.22	0.06	0.38	0.007	1722	-0.02	-0.23	0.19	0.832	1722	0.09	-0.08	0.26	0.309	0.015
Triglycerides to total lipids ratio in chylomicrons and extremely large VLDL (%)	1722	-0.18	-0.36	0.01	0.069	1722	0.06	-0.14	0.27	0.533	1722	-0.17	-0.36	0.03	0.100	0.170
Phospholipids to total lipds ratio in very large VLDL (%)	1722	0.25	0.09	0.41	0.002	1722	0.09	-0.11	0.29	0.365	1722	0.10	-0.07	0.27	0.237	0.007
Total cholesterol to total lipids ratio in very large VLDL (%)	1722	-0.01	-0.24	0.22	0.933	1722	0.00	-0.24	0.25	0.971	1722	-0.11	-0.34	0.13	0.372	0.868
Cholesterol esters to total lipids ratio in very large VLDL (%)	1722	-0.01	-0.17	0.16	0.948	1722	0.00	-0.19	0.18	0.965	1722	-0.09	-0.25	0.06	0.247	0.875
Free cholesterol to total lipids ratio in very large VLDL (%)	1722	-0.03	-0.15	0.09	0.613	1722	0.08	-0.08	0.25	0.316	1722	-0.06	-0.20	0.07	0.360	0.493
Triglycerides to total lipids ratio in very large VLDL (%)	1722	-0.01	-0.17	0.16	0.920	1722	-0.10	-0.33	0.13	0.395	1722	0.03	-0.14	0.20	0.719	0.706
Phospholipids to total lipds ratio in large VLDL (%)	1722	0.22	0.06	0.38	0.007	1722	-0.04	-0.25	0.17	0.697	1722	0.14	-0.03	0.30	0.099	0.028
Total cholesterol to total lipids ratio in large VLDL (%)	1722	0.25	0.08	0.41	0.003	1722	-0.01	-0.23	0.22	0.954	1722	0.12	-0.05	0.29	0.157	0.029
Cholesterol esters to total lipids ratio in large VLDL (%)	1722	0.11	-0.04	0.26	0.162	1722	0.00	-0.23	0.24	0.978	1722	0.03	-0.12	0.18	0.699	0.510
Free cholesterol to total lipids ratio in large VLDL (%)	1722	0.13	0.06	0.20	0.0003	1722	-0.01	-0.10	0.09	0.879	1722	0.07	-0.01	0.15	0.085	0.003
Triglycerides to total lipids ratio in large VLDL (%)	1722	-0.21	-0.38	-0.04	0.017	1722	-0.02	-0.24	0.21	0.871	1722	-0.15	-0.34	0.05	0.145	0.103
Phospholipids to total lipds ratio in medium VLDL (%)	1722	-0.25	-0.43	-0.08	0.005	1722	-0.06	-0.27	0.16	0.600	1722	-0.05	-0.22	0.12	0.581	0.015
Total cholesterol to total lipids ratio in medium VLDL (%)	1722	0.10	-0.07	0.27	0.256	1722	0.01	-0.21	0.22	0.953	1722	0.13	-0.05	0.31	0.169	0.631

Online Table 7 Associations of change in regional fat indexes from age 10-18y with cardiometabolic traits at age 18y in ALSPAC

Change from age 10-18y

	Trunk fat index (per SD-unit gain) *Adj. for age, sex, ethnicity, maternal education, trunk fat at 10y, arm fat index change, leg fat index change*					Arm fat index (per SD-unit gain) *Adj. for age, sex, ethnicity, maternal education, arm fat at 10y, trunk fat index change, leg fat index change*					Leg fat index (per SD-unit gain) *Adj. for age, sex, ethnicity, maternal education, leg fat at 10y, arm fat index change, trunk fat index change*					
**Standardized outcome at age 18y**	**N**	**Beta**	**LCL**	**UCL**	**P-value**	**N**	**Beta**	**LCL**	**UCL**	**P-value**	**N**	**Beta**	**LCL**	**UCL**	**P-value**	***P-value for regional heterogeneity***
Cholesterol esters to total lipids ratio in medium VLDL (%)	1722	0.06	-0.11	0.24	0.482	1722	0.02	-0.20	0.24	0.860	1722	0.13	-0.06	0.32	0.186	0.847
Free cholesterol to total lipids ratio in medium VLDL (%)	1722	0.17	-0.01	0.34	0.067	1722	-0.03	-0.25	0.19	0.775	1722	0.07	-0.10	0.24	0.419	0.165
Triglycerides to total lipids ratio in medium VLDL (%)	1722	-0.04	-0.21	0.13	0.636	1722	0.01	-0.20	0.22	0.943	1722	-0.11	-0.29	0.07	0.220	0.820
Phospholipids to total lipds ratio in small VLDL (%)	1722	-0.30	-0.47	-0.12	0.001	1722	-0.19	-0.42	0.05	0.120	1722	-0.08	-0.30	0.14	0.464	0.005
Total cholesterol to total lipids ratio in small VLDL (%)	1722	-0.11	-0.29	0.06	0.204	1722	0.14	-0.09	0.37	0.233	1722	0.09	-0.11	0.29	0.367	0.309
Cholesterol esters to total lipids ratio in small VLDL (%)	1722	-0.08	-0.26	0.10	0.385	1722	0.17	-0.06	0.40	0.148	1722	0.09	-0.11	0.29	0.371	0.500
Free cholesterol to total lipids ratio in small VLDL (%)	1722	-0.24	-0.41	-0.07	0.007	1722	-0.20	-0.42	0.02	0.071	1722	0.02	-0.17	0.21	0.831	0.004
Triglycerides to total lipids ratio in small VLDL (%)	1722	0.22	0.04	0.40	0.016	1722	-0.08	-0.32	0.15	0.486	1722	-0.06	-0.25	0.14	0.561	0.032
Phospholipids to total lipds ratio in very small VLDL (%)	1722	0.02	-0.16	0.19	0.854	1722	-0.10	-0.29	0.10	0.330	1722	0.10	-0.07	0.26	0.243	0.526
Total cholesterol to total lipids ratio in very small VLDL (%)	1722	-0.16	-0.36	0.03	0.104	1722	0.19	-0.04	0.41	0.107	1722	-0.03	-0.20	0.14	0.744	0.194
Cholesterol esters to total lipids ratio in very small VLDL (%)	1722	-0.06	-0.25	0.12	0.507	1722	0.21	0.00	0.43	0.055	1722	-0.02	-0.19	0.15	0.790	0.344
Free cholesterol to total lipids ratio in very small VLDL (%)	1722	-0.40	-0.61	-0.19	0.0002	1722	-0.02	-0.26	0.21	0.838	1722	-0.02	-0.21	0.17	0.841	0.001
Triglycerides to total lipids ratio in very small VLDL (%)	1722	0.19	0.00	0.39	0.055	1722	-0.17	-0.41	0.08	0.181	1722	-0.03	-0.22	0.16	0.732	0.140
Phospholipids to total lipds ratio in IDL (%)	1722	-0.28	-0.48	-0.08	0.006	1722	-0.04	-0.27	0.18	0.698	1722	-0.17	-0.38	0.03	0.093	0.045
Total cholesterol to total lipids ratio in IDL (%)	1722	0.10	-0.09	0.29	0.318	1722	0.17	-0.06	0.41	0.150	1722	0.11	-0.09	0.31	0.292	0.492
Cholesterol esters to total lipids ratio in IDL (%)	1722	0.23	0.04	0.42	0.020	1722	0.17	-0.06	0.40	0.138	1722	0.13	-0.07	0.32	0.210	0.053
Free cholesterol to total lipids ratio in IDL (%)	1722	-0.30	-0.48	-0.12	0.001	1722	0.02	-0.20	0.24	0.850	1722	-0.03	-0.23	0.17	0.758	0.008
Triglycerides to total lipids ratio in IDL (%)	1722	0.00	-0.18	0.19	0.972	1722	-0.18	-0.42	0.06	0.136	1722	-0.07	-0.26	0.13	0.511	0.743
Phospholipids to total lipds ratio in large LDL (%)	1722	-0.08	-0.24	0.09	0.356	1722	-0.02	-0.22	0.17	0.811	1722	-0.09	-0.27	0.09	0.332	0.660
Total cholesterol to total lipids ratio in large LDL (%)	1722	0.09	-0.07	0.26	0.263	1722	0.11	-0.11	0.33	0.327	1722	0.13	-0.07	0.34	0.192	0.563
Cholesterol esters to total lipids ratio in large LDL (%)	1722	0.20	0.04	0.36	0.016	1722	0.09	-0.12	0.30	0.382	1722	0.15	-0.04	0.35	0.124	0.070
Free cholesterol to total lipids ratio in large LDL (%)	1722	-0.36	-0.53	-0.19	<0.0001	1722	0.00	-0.21	0.21	0.998	1722	-0.12	-0.29	0.06	0.192	0.000
Triglycerides to total lipids ratio in large LDL (%)	1722	-0.06	-0.24	0.11	0.472	1722	-0.15	-0.39	0.08	0.206	1722	-0.12	-0.32	0.09	0.272	0.710
Phospholipids to total lipds ratio in medium LDL (%)	1722	-0.06	-0.23	0.10	0.455	1722	-0.03	-0.23	0.17	0.795	1722	-0.08	-0.26	0.10	0.370	0.744
Total cholesterol to total lipids ratio in medium LDL (%)	1722	0.08	-0.08	0.24	0.329	1722	0.09	-0.12	0.30	0.412	1722	0.10	-0.09	0.29	0.307	0.615
Cholesterol esters to total lipids ratio in medium LDL (%)	1722	0.14	-0.03	0.30	0.103	1722	0.06	-0.14	0.27	0.545	1722	0.12	-0.07	0.30	0.220	0.274
Free cholesterol to total lipids ratio in medium LDL (%)	1722	-0.19	-0.35	-0.02	0.025	1722	-0.02	-0.22	0.18	0.855	1722	-0.12	-0.29	0.05	0.162	0.093
Triglycerides to total lipids ratio in medium LDL (%)	1722	-0.05	-0.21	0.11	0.567	1722	-0.15	-0.35	0.06	0.164	1722	-0.06	-0.24	0.12	0.528	0.660
Phospholipids to total lipds ratio in small LDL (%)	1722	-0.11	-0.27	0.05	0.190	1722	-0.04	-0.24	0.16	0.688	1722	-0.12	-0.30	0.06	0.191	0.450
Total cholesterol to total lipids ratio in small LDL (%)	1722	0.04	-0.12	0.20	0.634	1722	0.09	-0.12	0.30	0.419	1722	0.11	-0.08	0.31	0.257	0.904
Cholesterol esters to total lipids ratio in small LDL (%)	1722	0.11	-0.06	0.27	0.207	1722	0.06	-0.14	0.27	0.536	1722	0.12	-0.06	0.31	0.195	0.469
Free cholesterol to total lipids ratio in small LDL (%)	1722	-0.20	-0.37	-0.02	0.025	1722	-0.01	-0.21	0.19	0.898	1722	-0.11	-0.28	0.05	0.173	0.084
Triglycerides to total lipids ratio in small LDL (%)	1722	0.17	-0.01	0.36	0.066	1722	-0.14	-0.38	0.09	0.237	1722	0.00	-0.20	0.20	0.994	0.175
Phospholipids to total lipds ratio in very large HDL (%)	1722	-0.34	-0.51	-0.17	0.0001	1722	-0.14	-0.34	0.06	0.161	1722	-0.04	-0.22	0.14	0.663	0.0003
Total cholesterol to total lipids ratio in very large HDL (%)	1722	0.29	0.12	0.46	0.001	1722	0.15	-0.04	0.34	0.127	1722	0.01	-0.16	0.18	0.885	0.001
Cholesterol esters to total lipids ratio in very large HDL (%)	1722	0.29	0.12	0.46	0.001	1722	0.14	-0.06	0.33	0.168	1722	0.03	-0.15	0.20	0.776	0.002
Free cholesterol to total lipids ratio in very large HDL (%)	1722	-0.06	-0.26	0.13	0.512	1722	0.13	-0.10	0.35	0.265	1722	-0.16	-0.38	0.05	0.137	0.287
Triglycerides to total lipids ratio in very large HDL (%)	1722	0.32	0.11	0.53	0.003	1722	-0.02	-0.26	0.22	0.888	1722	0.17	-0.07	0.41	0.166	0.024
Phospholipids to total lipds ratio in large HDL (%)	1722	0.50	0.31	0.69	<0.0001	1722	-0.01	-0.26	0.24	0.941	1722	0.24	0.01	0.46	0.042	<0.0001
Total cholesterol to total lipids ratio in large HDL (%)	1722	-0.51	-0.70	-0.32	<0.0001	1722	-0.01	-0.25	0.23	0.944	1722	-0.24	-0.47	-0.01	0.041	<0.0001
Cholesterol esters to total lipids ratio in large HDL (%)	1722	-0.51	-0.70	-0.32	<0.0001	1722	0.00	-0.25	0.24	0.981	1722	-0.27	-0.50	-0.03	0.028	<0.0001
Free cholesterol to total lipids ratio in large HDL (%)	1722	-0.41	-0.61	-0.22	<0.0001	1722	-0.03	-0.26	0.20	0.809	1722	-0.07	-0.27	0.13	0.500	0.0004
Triglycerides to total lipids ratio in large HDL (%)	1722	0.41	0.21	0.61	<0.0001	1722	0.02	-0.20	0.25	0.861	1722	0.21	-0.01	0.43	0.066	0.002
Phospholipids to total lipds ratio in medium HDL (%)	1722	-0.07	-0.25	0.11	0.424	1722	0.07	-0.15	0.29	0.553	1722	-0.10	-0.30	0.11	0.348	0.545
Total cholesterol to total lipids ratio in medium HDL (%)	1722	-0.17	-0.34	0.01	0.060	1722	-0.06	-0.29	0.17	0.605	1722	0.00	-0.21	0.20	0.981	0.175
Cholesterol esters to total lipids ratio in medium HDL (%)	1722	-0.17	-0.34	0.00	0.051	1722	-0.06	-0.29	0.16	0.590	1722	-0.04	-0.24	0.16	0.702	0.181
Free cholesterol to total lipids ratio in medium HDL (%)	1722	-0.03	-0.19	0.14	0.756	1722	-0.04	-0.25	0.16	0.670	1722	0.20	0.01	0.40	0.038	0.389
Triglycerides to total lipids ratio in medium HDL (%)	1722	0.46	0.27	0.65	<0.0001	1722	0.01	-0.22	0.25	0.906	1722	0.18	-0.04	0.40	0.103	0.0001
Phospholipids to total lipds ratio in small HDL (%)	1722	0.08	-0.09	0.24	0.354	1722	0.09	-0.11	0.29	0.370	1722	-0.16	-0.34	0.01	0.069	0.127
Total cholesterol to total lipids ratio in small HDL (%)	1722	-0.16	-0.32	0.01	0.060	1722	-0.10	-0.31	0.11	0.353	1722	0.14	-0.05	0.32	0.145	0.030
Cholesterol esters to total lipids ratio in small HDL (%)	1722	-0.10	-0.26	0.06	0.239	1722	-0.09	-0.30	0.11	0.373	1722	0.17	-0.01	0.35	0.065	0.087
Free cholesterol to total lipids ratio in small HDL (%)	1722	-0.33	-0.50	-0.16	0.0001	1722	0.03	-0.19	0.25	0.776	1722	-0.30	-0.51	-0.10	0.004	0.003

Online Table 7 Associations of change in regional fat indexes from age 10-18y with cardiometabolic traits at age 18y in ALSPAC

Change from age 10-18y

	Trunk fat index (per SD-unit gain) *Adj. for age, sex, ethnicity, maternal education, trunk fat at 10y, arm fat index change, leg fat index change*					Arm fat index (per SD-unit gain) *Adj. for age, sex, ethnicity, maternal education, arm fat at 10y, trunk fat index change, leg fat index change*					Leg fat index (per SD-unit gain) *Adj. for age, sex, ethnicity, maternal education, leg fat at 10y, arm fat index change, trunk fat index change*					
**Standardized outcome at age 18y**	**N**	**Beta**	**LCL**	**UCL**	**P-value**	**N**	**Beta**	**LCL**	**UCL**	**P-value**	**N**	**Beta**	**LCL**	**UCL**	**P-value**	***P-value for regional heterogeneity***
Triglycerides to total lipids ratio in small HDL (%)	1722	0.31	0.12	0.50	0.001	1722	0.04	-0.23	0.32	0.766	1722	0.08	-0.16	0.32	0.517	0.013
Mean diameter for VLDL particles (nm)	1722	0.45	0.27	0.62	<0.0001	1722	0.04	-0.19	0.28	0.706	1722	0.08	-0.12	0.28	0.412	<0.0001
Mean diameter for LDL particles (nm)	1722	-0.20	-0.37	-0.03	0.020	1722	-0.06	-0.27	0.14	0.546	1722	-0.03	-0.22	0.16	0.763	0.022
Mean diameter for HDL particles (nm)	1722	-0.41	-0.59	-0.23	<0.0001	1722	0.04	-0.17	0.24	0.726	1722	-0.14	-0.32	0.04	0.134	0.0001
Serum total cholesterol (mmol/l)	1722	0.08	-0.10	0.26	0.368	1722	0.04	-0.18	0.25	0.737	1722	0.21	0.02	0.41	0.033	0.663
Total cholesterol in VLDL (mmol/l)	1722	0.40	0.22	0.58	<0.0001	1722	0.09	-0.15	0.32	0.459	1722	0.25	0.04	0.47	0.021	0.001
Remnant cholesterol (non-HDL, non-LDL -cholesterol) (mmol/l)	1722	0.29	0.11	0.47	0.002	1722	0.08	-0.15	0.31	0.501	1722	0.28	0.06	0.50	0.012	0.022
Total cholesterol in LDL (mmol/l)	1722	0.11	-0.07	0.29	0.221	1722	0.04	-0.19	0.26	0.742	1722	0.24	0.04	0.44	0.022	0.509
Total cholesterol in HDL (mmol/l)	1722	-0.28	-0.46	-0.10	0.002	1722	-0.04	-0.25	0.17	0.678	1722	-0.08	-0.27	0.11	0.410	0.015
Total cholesterol in HDL2 (mmol/l)	1722	-0.32	-0.50	-0.15	0.0004	1722	-0.05	-0.26	0.17	0.660	1722	-0.10	-0.30	0.09	0.309	0.004
Total cholesterol in HDL3 (mmol/l)	1722	-0.19	-0.36	-0.01	0.036	1722	-0.04	-0.24	0.17	0.729	1722	-0.04	-0.22	0.14	0.684	0.146
Esterified cholesterol (mmol/l)	1722	0.07	-0.11	0.25	0.448	1722	0.07	-0.15	0.28	0.554	1722	0.19	-0.01	0.38	0.068	0.817
Free cholesterol (mmol/l)	1722	0.10	-0.07	0.27	0.253	1722	-0.03	-0.24	0.17	0.743	1722	0.25	0.07	0.44	0.008	0.306
Serum total triglycerides (mmol/l)	1722	0.43	0.24	0.62	<0.0001	1722	0.03	-0.21	0.27	0.801	1722	0.14	-0.06	0.35	0.170	0.0002
Triglycerides in VLDL (mmol/l)	1722	0.47	0.28	0.66	<0.0001	1722	0.06	-0.18	0.30	0.632	1722	0.13	-0.08	0.34	0.222	<0.0001
Triglycerides in LDL (mmol/l)	1722	0.07	-0.12	0.25	0.471	1722	-0.06	-0.27	0.15	0.573	1722	0.09	-0.09	0.27	0.316	0.650
Triglycerides in HDL (mmol/l)	1722	0.26	0.07	0.45	0.007	1722	-0.05	-0.27	0.17	0.660	1722	0.15	-0.04	0.33	0.125	0.041
Diacylglycerol (mmol/l)	1722	0.27	0.08	0.45	0.006	1722	0.16	-0.09	0.40	0.204	1722	0.06	-0.14	0.26	0.571	0.013
Ratio of diacylglycerol to triglycerides	1722	0.09	-0.09	0.27	0.308	1722	0.08	-0.13	0.29	0.469	1722	0.03	-0.14	0.21	0.713	0.412
Total phosphoglycerides (mmol/l)	1722	-0.02	-0.21	0.16	0.795	1722	-0.03	-0.23	0.17	0.779	1722	0.09	-0.09	0.27	0.325	0.791
Ratio of triglycerides to phosphoglycerides	1722	0.45	0.26	0.63	<0.0001	1722	0.01	-0.22	0.24	0.918	1722	0.14	-0.08	0.36	0.208	<0.0001
Phosphatidylcholine and other cholines (mmol/l)	1722	0.01	-0.17	0.19	0.908	1722	-0.06	-0.25	0.13	0.559	1722	0.04	-0.13	0.21	0.665	0.875
Total cholines (mmol/l)	1722	-0.01	-0.19	0.17	0.925	1722	-0.05	-0.25	0.15	0.612	1722	0.09	-0.08	0.27	0.298	0.708
Apolipoprotein A-I (g/l)	1722	-0.14	-0.31	0.04	0.123	1722	-0.04	-0.24	0.16	0.683	1722	0.01	-0.17	0.19	0.923	0.315
Apolipoprotein B (g/l)	1722	0.34	0.16	0.52	0.0002	1722	0.05	-0.18	0.28	0.659	1722	0.26	0.05	0.47	0.017	0.004
Ratio of apolipoprotein B to apolipoprotein A-I	1722	0.42	0.24	0.60	<0.0001	1722	0.07	-0.17	0.31	0.571	1722	0.25	0.02	0.47	0.032	0.0004
Total fatty acids (mmol/l)	1722	0.22	0.04	0.41	0.018	1722	-0.01	-0.23	0.21	0.939	1722	0.19	0.00	0.37	0.052	0.088
Estimated description of fatty acid chain length, not actual carbon number	1722	0.10	-0.06	0.26	0.234	1722	0.00	-0.21	0.22	0.970	1722	-0.08	-0.26	0.10	0.407	0.357
Estimated degree of unsaturation	1722	-0.13	-0.30	0.05	0.155	1722	-0.01	-0.22	0.21	0.953	1722	0.02	-0.16	0.20	0.843	0.364
22:6, docosahexaenoic acid (mmol/l)	1722	-0.07	-0.25	0.11	0.457	1722	0.13	-0.09	0.34	0.250	1722	0.09	-0.09	0.26	0.329	0.659
18:2, linoleic acid (mmol/l)	1722	0.08	-0.10	0.25	0.381	1722	-0.08	-0.29	0.13	0.467	1722	0.20	0.02	0.38	0.032	0.336
Conjugated linoleic acid (mmol/l)	1722	0.06	-0.11	0.22	0.486	1722	0.31	0.09	0.53	0.005	1722	-0.09	-0.29	0.10	0.336	0.033
Omega-3 fatty acids (mmol/l)	1722	0.02	-0.17	0.21	0.819	1722	0.15	-0.07	0.37	0.181	1722	0.13	-0.06	0.31	0.180	0.924
Omega-6 fatty acids (mmol/l)	1722	0.10	-0.08	0.27	0.289	1722	-0.06	-0.27	0.16	0.610	1722	0.22	0.04	0.40	0.018	0.330
Polyunsaturated fatty acids (mmol/l)	1722	0.09	-0.09	0.27	0.327	1722	-0.03	-0.24	0.19	0.791	1722	0.22	0.04	0.40	0.019	0.429
Monounsaturated fatty acids; 16:1, 18:1 (mmol/l)	1722	0.36	0.18	0.54	0.0001	1722	-0.06	-0.28	0.17	0.615	1722	0.18	-0.01	0.38	0.064	0.002
Saturated fatty acids (mmol/l)	1722	0.17	-0.02	0.35	0.080	1722	0.05	-0.17	0.28	0.639	1722	0.12	-0.07	0.31	0.205	0.264
Ratio of 22:6 docosahexaenoic acid to total fatty acids (%)	1722	-0.24	-0.41	-0.08	0.004	1722	0.21	0.00	0.42	0.055	1722	-0.02	-0.19	0.15	0.826	0.014
Ratio of 18:2 linoleic acid to total fatty acids (%)	1722	-0.28	-0.45	-0.11	0.001	1722	-0.10	-0.32	0.11	0.350	1722	0.00	-0.18	0.18	0.988	0.002
Ratio of conjugated linoleic acid to total fatty acids (%)	1722	0.01	-0.14	0.15	0.921	1722	0.30	0.10	0.51	0.004	1722	-0.10	-0.28	0.09	0.309	0.049
Ratio of omega-3 fatty acids to total fatty acids (%)	1722	-0.20	-0.37	-0.02	0.026	1722	0.24	0.02	0.46	0.034	1722	-0.02	-0.20	0.16	0.805	0.039
Ratio of omega-6 fatty acids to total fatty acids (%)	1722	-0.31	-0.48	-0.14	0.0004	1722	-0.06	-0.29	0.16	0.575	1722	0.04	-0.15	0.22	0.715	0.001
Ratio of polyunsaturated fatty acids to total fatty acids (%)	1722	-0.34	-0.51	-0.17	0.0001	1722	0.00	-0.23	0.22	0.974	1722	0.03	-0.16	0.21	0.771	0.0004
Ratio of monounsaturated fatty acids to total fatty acids (%)	1722	0.42	0.25	0.59	<0.0001	1722	-0.13	-0.35	0.09	0.254	1722	0.10	-0.09	0.29	0.292	<0.0001
Ratio of saturated fatty acids to total fatty acids (%)	1722	-0.16	-0.34	0.02	0.088	1722	0.18	-0.04	0.40	0.107	1722	-0.17	-0.35	0.01	0.064	0.089
Insulin (mu/l)	1722	0.43	0.06	0.79	0.023	1722	-0.20	-0.60	0.21	0.335	1722	0.44	0.14	0.74	0.004	0.075
Glucose (mmol/l)	1722	0.13	-0.01	0.26	0.064	1722	-0.04	-0.20	0.12	0.640	1722	0.12	-0.01	0.26	0.068	0.195
Lactate (mmol/l)	1722	-0.05	-0.26	0.16	0.643	1722	0.15	-0.06	0.36	0.163	1722	-0.01	-0.21	0.20	0.948	0.574
Pyruvate (mmol/l)	1722	0.13	-0.05	0.30	0.159	1722	0.19	-0.02	0.40	0.077	1722	-0.05	-0.24	0.13	0.570	0.042
Citrate (mmol/l)	1722	-0.28	-0.45	-0.11	0.001	1722	-0.13	-0.33	0.08	0.221	1722	0.15	-0.02	0.33	0.090	<0.0001
Alanine (mmol/l)	1722	0.20	0.04	0.37	0.015	1722	-0.07	-0.28	0.13	0.482	1722	0.02	-0.16	0.21	0.806	0.049
Glutamine (mmol/l)	1722	-0.09	-0.24	0.07	0.276	1722	0.08	-0.12	0.28	0.420	1722	-0.08	-0.24	0.09	0.368	0.417

Online Table 7 Associations of change in regional fat indexes from age 10-18y with cardiometabolic traits at age 18y in ALSPAC

	Change from age 10-18y														
	**Trunk fat index (per SD-unit gain)**					**Arm fat index (per SD-unit gain)**					**Leg fat index (per SD-unit gain)**				
	Adj. for age, sex, ethnicity, maternal education, trunk fat at 10y, arm fat index change, leg fat index change					*Adj. for age, sex, ethnicity, maternal education, arm fat at 10y, trunk fat index change, leg fat index change*					*Adj. for age, sex, ethnicity, maternal education, leg fat at 10y, arm fat index change, trunk fat index change*				***P-value for regional heterogeneity***
**Standardized outcome at age 18y**	**N**	**Beta**	**LCL**	**UCL**	**P-value**	**N**	**Beta**	**LCL**	**UCL**	**P-value**	**N**	**Beta**	**LCL**	**UCL**	**P-value**	
Histidine (mmol/l)	1722	0.10	-0.07	0.28	0.245	1722	-0.15	-0.38	0.08	0.200	1722	0.06	-0.15	0.28	0.548	0.333
Isoleucine (mmol/l)	1722	0.27	0.11	0.44	0.001	1722	0.05	-0.15	0.25	0.621	1722	0.09	-0.10	0.28	0.335	0.007
Leucine (mmol/l)	1722	0.17	0.03	0.32	0.020	1722	-0.02	-0.21	0.16	0.796	1722	0.12	-0.06	0.30	0.206	0.078
Valine (mmol/l)	1722	0.10	-0.05	0.24	0.208	1722	0.11	-0.08	0.30	0.275	1722	0.21	0.04	0.38	0.014	0.409
Phenylalanine (mmol/l)	1722	0.30	0.14	0.46	0.0003	1722	-0.08	-0.28	0.12	0.429	1722	0.14	-0.03	0.31	0.104	0.002
Tyrosine (mmol/l)	1722	0.23	0.06	0.39	0.008	1722	0.12	-0.08	0.32	0.239	1722	0.22	0.04	0.40	0.016	0.044
Acetate (mmol/l)	1722	-0.08	-0.15	-0.01	0.022	1722	0.01	-0.07	0.09	0.818	1722	0.00	-0.07	0.07	0.955	0.041
Acetoacetate (mmol/l)	1722	-0.09	-0.30	0.12	0.410	1722	0.06	-0.19	0.30	0.658	1722	-0.10	-0.28	0.08	0.275	0.688
3-hydroxybutyrate (mmol/l)	1722	-0.01	-0.26	0.24	0.936	1722	-0.05	-0.31	0.22	0.736	1722	-0.10	-0.27	0.08	0.275	0.943
Creatinine (mmol/l)	1722	0.26	0.12	0.41	0.0005	1722	-0.19	-0.36	-0.01	0.035	1722	-0.08	-0.23	0.08	0.323	0.002
Albumin (signal area)	1722	-0.01	-0.17	0.15	0.892	1722	-0.04	-0.25	0.17	0.727	1722	-0.10	-0.29	0.08	0.274	0.940
Glycoprotein acetyls, mainly a1-acid glycoprotein (mmol/l)	1722	0.41	0.24	0.59	<0.0001	1722	0.03	-0.19	0.25	0.805	1722	0.23	0.04	0.42	0.019	<0.0001
C-reactive protein (mg/l)	1722	0.14	-0.01	0.30	0.067	1722	-0.06	-0.21	0.10	0.489	1722	0.11	-0.05	0.27	0.167	0.150

Online Table 8 Associations of change in lean mass index from age 10-18y with cardiometabolic traits at age 18y in ALSPAC

Change from age 10-18y

Lean mass index (per SD-unit gain)

	Adj. for age, sex, ethnicity, maternal education, lean mass index at 10y					*Additionally adj. for fat mass index change*				
**Standardized outcome at age 18y**	**N**	**Beta**	**LCL**	**UCL**	**P-value**	**N**	**Beta**	**LCL**	**UCL**	**P-value**
Systolic blood pressure (mmHg)	3409	0.37	0.30	0.43	<0.0001	3409	0.19	0.16	0.22	<0.0001
Diastolic blood pressure (mmHg)	3409	0.05	-0.02	0.13	0.182	3409	0.08	0.04	0.13	<0.0001
Concentration of chylomicrons and extremely large VLDL particles (mol/l)	2342	0.26	0.16	0.35	<0.0001	2342	0.10	0.05	0.15	0.0003
Total lipids in chylomicrons and extremely large VLDL (mmol/l)	2342	0.26	0.17	0.35	<0.0001	2342	0.10	0.05	0.15	0.0003
Phospholipids in chylomicrons and extremely large VLDL (mmol/l)	2342	0.26	0.17	0.35	<0.0001	2342	0.10	0.04	0.15	0.0004
Total cholesterol in chylomicrons and extremely large VLDL (mmol/l)	2342	0.27	0.17	0.36	<0.0001	2342	0.10	0.04	0.15	0.0004
Cholesterol esters in chylomicrons and extremely large VLDL (mmol/l)	2342	0.27	0.17	0.36	<0.0001	2342	0.09	0.04	0.15	0.001
Free cholesterol in chylomicrons and extremely large VLDL (mmol/l)	2342	0.26	0.17	0.35	<0.0001	2342	0.10	0.05	0.15	0.0003
Triglycerides in chylomicrons and extremely large VLDL (mmol/l)	2342	0.26	0.16	0.35	<0.0001	2342	0.10	0.05	0.15	0.0003
Concentration of very large VLDL particles (mol/l)	2342	0.28	0.18	0.37	<0.0001	2342	0.10	0.05	0.16	0.0002
Total lipids in very large VLDL (mmol/l)	2342	0.28	0.19	0.37	<0.0001	2342	0.10	0.05	0.16	0.0002
Phospholipids in very large VLDL (mmol/l)	2342	0.27	0.18	0.37	<0.0001	2342	0.10	0.05	0.15	0.0003
Total cholesterol in very large VLDL (mmol/l)	2342	0.27	0.18	0.37	<0.0001	2342	0.10	0.05	0.16	0.0002
Cholesterol esters in very large VLDL (mmol/l)	2342	0.28	0.18	0.37	<0.0001	2342	0.10	0.05	0.16	0.0002
Free cholesterol in very large VLDL (mmol/l)	2342	0.26	0.17	0.36	<0.0001	2342	0.10	0.04	0.15	0.0003
Triglycerides in very large VLDL (mmol/l)	2342	0.28	0.19	0.37	<0.0001	2342	0.10	0.05	0.16	0.0002
Concentration of large VLDL particles (mol/l)	2342	0.29	0.20	0.38	<0.0001	2342	0.10	0.05	0.15	0.0002
Total lipids in large VLDL (mmol/l)	2342	0.29	0.20	0.38	<0.0001	2342	0.10	0.05	0.15	0.0002
Phospholipids in large VLDL (mmol/l)	2342	0.28	0.19	0.38	<0.0001	2342	0.10	0.05	0.15	0.0002
Total cholesterol in large VLDL (mmol/l)	2342	0.28	0.19	0.38	<0.0001	2342	0.10	0.05	0.15	0.0002
Cholesterol esters in large VLDL (mmol/l)	2342	0.28	0.19	0.37	<0.0001	2342	0.10	0.05	0.15	0.0002
Free cholesterol in large VLDL (mmol/l)	2342	0.28	0.19	0.38	<0.0001	2342	0.10	0.05	0.15	0.0003
Triglycerides in large VLDL (mmol/l)	2342	0.29	0.20	0.38	<0.0001	2342	0.10	0.05	0.15	0.0002
Concentration of medium VLDL particles (mol/l)	2342	0.29	0.19	0.38	<0.0001	2342	0.10	0.05	0.15	0.0002
Total lipids in medium VLDL (mmol/l)	2342	0.28	0.19	0.38	<0.0001	2342	0.10	0.05	0.15	0.0002
Phospholipids in medium VLDL (mmol/l)	2342	0.28	0.19	0.37	<0.0001	2342	0.10	0.04	0.15	0.0003
Total cholesterol in medium VLDL (mmol/l)	2342	0.26	0.17	0.35	<0.0001	2342	0.09	0.04	0.14	0.0005
Cholesterol esters in medium VLDL (mmol/l)	2342	0.24	0.15	0.34	<0.0001	2342	0.09	0.04	0.14	0.001
Free cholesterol in medium VLDL (mmol/l)	2342	0.27	0.18	0.36	<0.0001	2342	0.09	0.04	0.15	0.0004
Triglycerides in medium VLDL (mmol/l)	2342	0.29	0.20	0.38	<0.0001	2342	0.10	0.05	0.15	0.0002
Concentration of small VLDL particles (mol/l)	2342	0.25	0.16	0.34	<0.0001	2342	0.08	0.03	0.13	0.002
Total lipids in small VLDL (mmol/l)	2342	0.24	0.15	0.32	<0.0001	2342	0.07	0.02	0.12	0.005
Phospholipids in small VLDL (mmol/l)	2342	0.24	0.15	0.32	<0.0001	2342	0.07	0.02	0.12	0.010
Total cholesterol in small VLDL (mmol/l)	2342	0.17	0.08	0.26	0.0002	2342	0.06	0.01	0.11	0.029
Cholesterol esters in small VLDL (mmol/l)	2342	0.14	0.05	0.23	0.003	2342	0.05	0.00	0.10	0.051
Free cholesterol in small VLDL (mmol/l)	2342	0.22	0.13	0.30	<0.0001	2342	0.06	0.01	0.11	0.016
Triglycerides in small VLDL (mmol/l)	2342	0.26	0.17	0.35	<0.0001	2342	0.08	0.03	0.13	0.001
Concentration of very small VLDL particles (mol/l)	2342	0.09	0.00	0.17	0.042	2342	0.02	-0.03	0.07	0.369
Total lipids in very small VLDL (mmol/l)	2342	0.09	0.00	0.18	0.056	2342	0.02	-0.03	0.07	0.367
Phospholipids in very small VLDL (mmol/l)	2342	0.04	-0.05	0.12	0.376	2342	0.01	-0.04	0.06	0.719
Total cholesterol in very small VLDL (mmol/l)	2342	0.06	-0.03	0.16	0.199	2342	0.01	-0.03	0.06	0.570
Cholesterol esters in very small VLDL (mmol/l)	2342	0.07	-0.03	0.17	0.171	2342	0.02	-0.03	0.07	0.357
Free cholesterol in very small VLDL (mmol/l)	2342	0.05	-0.04	0.14	0.312	2342	-0.01	-0.06	0.04	0.708
Triglycerides in very small VLDL (mmol/l)	2342	0.16	0.08	0.24	<0.0001	2342	0.05	0.00	0.10	0.049
Concentration of IDL particles (mol/l)	2342	0.01	-0.08	0.09	0.871	2342	0.00	-0.04	0.05	0.922
Total lipids in IDL (mmol/l)	2342	0.01	-0.08	0.10	0.842	2342	0.00	-0.05	0.05	0.965
Phospholipids in IDL (mmol/l)	2342	-0.01	-0.10	0.07	0.791	2342	0.00	-0.05	0.04	0.861
Total cholesterol in IDL (mmol/l)	2342	0.01	-0.08	0.10	0.781	2342	0.00	-0.05	0.05	0.947
Cholesterol esters in IDL (mmol/l)	2342	0.03	-0.06	0.12	0.481	2342	0.01	-0.04	0.06	0.732
Free cholesterol in IDL (mmol/l)	2342	-0.04	-0.12	0.05	0.421	2342	-0.01	-0.06	0.03	0.534
Triglycerides in IDL (mmol/l)	2342	0.03	-0.05	0.10	0.487	2342	0.01	-0.04	0.05	0.734
Concentration of large LDL particles (mol/l)	2342	0.01	-0.07	0.10	0.723	2342	0.00	-0.05	0.05	0.978
Total lipids in large LDL (mmol/l)	2342	0.01	-0.07	0.10	0.764	2342	0.00	-0.05	0.05	0.997
Phospholipids in large LDL (mmol/l)	2342	0.02	-0.06	0.11	0.572	2342	0.00	-0.04	0.05	0.867
Total cholesterol in large LDL (mmol/l)	2342	0.01	-0.08	0.09	0.827	2342	0.00	-0.05	0.05	0.951
Cholesterol esters in large LDL (mmol/l)	2342	0.02	-0.06	0.11	0.632	2342	0.00	-0.04	0.05	0.913
Free cholesterol in large LDL (mmol/l)	2342	-0.03	-0.11	0.06	0.550	2342	-0.01	-0.06	0.03	0.559
Triglycerides in large LDL (mmol/l)	2342	0.01	-0.07	0.08	0.810	2342	0.00	-0.04	0.05	0.940
Concentration of medium LDL particles (mol/l)	2342	0.04	-0.05	0.12	0.385	2342	0.01	-0.04	0.05	0.753
Total lipids in medium LDL (mmol/l)	2342	0.03	-0.05	0.11	0.493	2342	0.00	-0.04	0.05	0.915
Phospholipids in medium LDL (mmol/l)	2342	0.06	-0.02	0.14	0.157	2342	0.01	-0.04	0.05	0.758
Total cholesterol in medium LDL (mmol/l)	2342	0.02	-0.06	0.11	0.590	2342	0.00	-0.04	0.05	0.931
Cholesterol esters in medium LDL (mmol/l)	2342	0.03	-0.06	0.11	0.534	2342	0.01	-0.04	0.05	0.830
Free cholesterol in medium LDL (mmol/l)	2342	0.01	-0.08	0.09	0.834	2342	-0.01	-0.06	0.03	0.620
Triglycerides in medium LDL (mmol/l)	2342	0.00	-0.08	0.07	0.907	2342	0.00	-0.05	0.04	0.918
Concentration of small LDL particles (mol/l)	2342	0.05	-0.03	0.13	0.238	2342	0.00	-0.04	0.05	0.945
Total lipids in small LDL (mmol/l)	2342	0.04	-0.05	0.12	0.374	2342	0.00	-0.05	0.05	0.971
Phospholipids in small LDL (mmol/l)	2342	0.07	-0.01	0.15	0.103	2342	0.00	-0.04	0.05	0.930
Total cholesterol in small LDL (mmol/l)	2342	0.02	-0.06	0.10	0.638	2342	0.00	-0.05	0.04	0.904
Cholesterol esters in small LDL (mmol/l)	2342	0.02	-0.06	0.10	0.652	2342	0.00	-0.05	0.05	0.961
Free cholesterol in small LDL (mmol/l)	2342	0.02	-0.06	0.11	0.572	2342	-0.02	-0.07	0.03	0.388
Triglycerides in small LDL (mmol/l)	2342	0.09	0.01	0.16	0.027	2342	0.03	-0.02	0.07	0.258
Concentration of very large HDL particles (mol/l)	2342	-0.18	-0.27	-0.10	<0.0001	2342	-0.07	-0.11	-0.02	0.003
Total lipids in very large HDL (mmol/l)	2342	-0.18	-0.26	-0.09	<0.0001	2342	-0.07	-0.12	-0.03	0.002
Phospholipids in very large HDL (mmol/l)	2342	-0.20	-0.29	-0.12	<0.0001	2342	-0.07	-0.11	-0.02	0.002
Total cholesterol in very large HDL (mmol/l)	2342	-0.14	-0.23	-0.05	0.001	2342	-0.07	-0.12	-0.02	0.003
Cholesterol esters in very large HDL (mmol/l)	2342	-0.12	-0.21	-0.04	0.005	2342	-0.07	-0.12	-0.02	0.004
Free cholesterol in very large HDL (mmol/l)	2342	-0.18	-0.26	-0.09	<0.0001	2342	-0.07	-0.12	-0.02	0.003
Triglycerides in very large HDL (mmol/l)	2342	0.03	-0.06	0.12	0.508	2342	0.02	-0.03	0.07	0.388
Concentration of large HDL particles (mol/l)	2342	-0.16	-0.24	-0.07	0.0002	2342	-0.07	-0.11	-0.02	0.004
Total lipids in large HDL (mmol/l)	2342	-0.17	-0.25	-0.08	<0.0001	2342	-0.07	-0.11	-0.02	0.003
Phospholipids in large HDL (mmol/l)	2342	-0.14	-0.22	-0.06	0.0004	2342	-0.06	-0.10	-0.02	0.006
Total cholesterol in large HDL (mmol/l)	2342	-0.19	-0.27	-0.10	<0.0001	2342	-0.08	-0.12	-0.03	0.001
Cholesterol esters in large HDL (mmol/l)	2342	-0.19	-0.27	-0.10	<0.0001	2342	-0.08	-0.12	-0.03	0.001
Free cholesterol in large HDL (mmol/l)	2342	-0.19	-0.28	-0.11	<0.0001	2342	-0.08	-0.12	-0.03	0.001
Triglycerides in large HDL (mmol/l)	2342	0.06	-0.02	0.14	0.125	2342	0.03	-0.01	0.08	0.142

Online Table 8 Associations of change in lean mass index from age 10-18y with cardiometabolic traits at age 18y in ALSPAC

Change from age 10-18y

Lean mass index (per SD-unit gain)

	Adj. for age, sex, ethnicity, maternal education, lean mass index at 10y					*Additionally adj. for fat mass index change*				
**Standardized outcome at age 18y**	**N**	**Beta**	**LCL**	**UCL**	**P-value**	**N**	**Beta**	**LCL**	**UCL**	**P-value**
Concentration of medium HDL particles (mol/l)	2342	0.05	-0.03	0.13	0.230	2342	-0.02	-0.06	0.03	0.500
Total lipids in medium HDL (mmol/l)	2342	0.02	-0.06	0.10	0.559	2342	-0.02	-0.07	0.02	0.329
Phospholipids in medium HDL (mmol/l)	2342	0.02	-0.05	0.10	0.576	2342	-0.02	-0.07	0.02	0.277
Total cholesterol in medium HDL (mmol/l)	2342	-0.01	-0.10	0.07	0.764	2342	-0.03	-0.08	0.01	0.145
Cholesterol esters in medium HDL (mmol/l)	2342	-0.02	-0.11	0.07	0.665	2342	-0.04	-0.08	0.01	0.125
Free cholesterol in medium HDL (mmol/l)	2342	0.01	-0.07	0.09	0.753	2342	-0.02	-0.07	0.02	0.292
Triglycerides in medium HDL (mmol/l)	2342	0.26	0.17	0.34	<0.0001	2342	0.09	0.04	0.14	0.0002
Concentration of small HDL particles (mol/l)	2342	0.15	0.06	0.23	0.001	2342	0.02	-0.03	0.07	0.402
Total lipids in small HDL (mmol/l)	2342	0.09	0.01	0.16	0.031	2342	0.00	-0.05	0.04	0.875
Phospholipids in small HDL (mmol/l)	2342	0.15	0.06	0.24	0.001	2342	0.02	-0.02	0.07	0.308
Total cholesterol in small HDL (mmol/l)	2342	-0.03	-0.10	0.04	0.407	2342	-0.05	-0.09	0.00	0.030
Cholesterol esters in small HDL (mmol/l)	2342	-0.05	-0.13	0.02	0.134	2342	-0.05	-0.09	-0.01	0.014
Free cholesterol in small HDL (mmol/l)	2342	0.07	-0.02	0.15	0.129	2342	-0.01	-0.06	0.03	0.566
Triglycerides in small HDL (mmol/l)	2342	0.22	0.13	0.30	<0.0001	2342	0.08	0.03	0.13	0.003
Phospholipids to total lipds ratio in chylomicrons and extremely large VLDL (%)	2342	0.04	-0.03	0.11	0.296	2342	0.02	-0.01	0.06	0.194
Total cholesterol to total lipids ratio in chylomicrons and extremely large VLDL (%)	2342	0.10	0.01	0.19	0.029	2342	0.04	-0.02	0.10	0.165
Cholesterol esters to total lipids ratio in chylomicrons and extremely large VLDL (%)	2342	0.07	-0.02	0.16	0.123	2342	0.03	-0.03	0.09	0.405
Free cholesterol to total lipids ratio in chylomicrons and extremely large VLDL (%)	2342	0.10	0.01	0.18	0.024	2342	0.07	0.01	0.12	0.012
Triglycerides to total lipids ratio in chylomicrons and extremely large VLDL (%)	2342	-0.11	-0.19	-0.02	0.013	2342	-0.06	-0.12	-0.01	0.024
Phospholipids to total lipds ratio in very large VLDL (%)	2342	0.13	0.04	0.22	0.003	2342	0.05	0.01	0.10	0.028
Total cholesterol to total lipids ratio in very large VLDL (%)	2342	-0.08	-0.16	0.01	0.079	2342	0.00	-0.05	0.06	0.862
Cholesterol esters to total lipids ratio in very large VLDL (%)	2342	-0.07	-0.17	0.02	0.134	2342	0.01	-0.05	0.07	0.713
Free cholesterol to total lipids ratio in very large VLDL (%)	2342	-0.06	-0.16	0.04	0.228	2342	0.02	-0.04	0.07	0.562
Triglycerides to total lipids ratio in very large VLDL (%)	2342	-0.02	-0.11	0.07	0.658	2342	0.01	-0.04	0.06	0.733
Phospholipids to total lipds ratio in large VLDL (%)	2342	0.09	0.01	0.17	0.028	2342	0.05	0.00	0.10	0.043
Total cholesterol to total lipids ratio in large VLDL (%)	2342	0.12	0.02	0.23	0.016	2342	0.07	0.02	0.12	0.006
Cholesterol esters to total lipids ratio in large VLDL (%)	2342	0.05	-0.06	0.16	0.374	2342	0.06	0.01	0.11	0.030
Free cholesterol to total lipids ratio in large VLDL (%)	2342	0.09	0.02	0.17	0.018	2342	0.01	-0.04	0.05	0.817
Triglycerides to total lipids ratio in large VLDL (%)	2342	-0.12	-0.21	-0.03	0.012	2342	-0.07	-0.12	-0.02	0.005
Phospholipids to total lipds ratio in medium VLDL (%)	2342	-0.12	-0.20	-0.04	0.004	2342	-0.04	-0.09	0.00	0.059
Total cholesterol to total lipids ratio in medium VLDL (%)	2342	0.04	-0.04	0.13	0.306	2342	0.03	-0.02	0.07	0.236
Cholesterol esters to total lipids ratio in medium VLDL (%)	2342	0.02	-0.06	0.11	0.579	2342	0.02	-0.02	0.06	0.375
Free cholesterol to total lipids ratio in medium VLDL (%)	2342	0.09	0.01	0.17	0.022	2342	0.03	-0.02	0.08	0.242
Triglycerides to total lipids ratio in medium VLDL (%)	2342	-0.01	-0.10	0.07	0.737	2342	-0.02	-0.06	0.03	0.407
Phospholipids to total lipds ratio in small VLDL (%)	2342	-0.11	-0.20	-0.02	0.014	2342	-0.06	-0.11	-0.01	0.016
Total cholesterol to total lipids ratio in small VLDL (%)	2342	-0.12	-0.22	-0.03	0.010	2342	-0.03	-0.07	0.02	0.263
Cholesterol esters to total lipids ratio in small VLDL (%)	2342	-0.11	-0.20	-0.02	0.020	2342	-0.02	-0.06	0.03	0.465
Free cholesterol to total lipids ratio in small VLDL (%)	2342	-0.10	-0.18	-0.02	0.011	2342	-0.06	-0.10	-0.02	0.007
Triglycerides to total lipids ratio in small VLDL (%)	2342	0.16	0.07	0.25	0.0005	2342	0.05	0.00	0.09	0.047
Phospholipids to total lipds ratio in very small VLDL (%)	2342	-0.08	-0.15	0.00	0.058	2342	-0.01	-0.06	0.03	0.511
Total cholesterol to total lipids ratio in very small VLDL (%)	2342	-0.06	-0.14	0.02	0.150	2342	-0.02	-0.07	0.02	0.312
Cholesterol esters to total lipids ratio in very small VLDL (%)	2342	-0.03	-0.11	0.05	0.455	2342	0.00	-0.05	0.04	0.946
Free cholesterol to total lipids ratio in very small VLDL (%)	2342	-0.11	-0.19	-0.03	0.010	2342	-0.08	-0.13	-0.04	0.001
Triglycerides to total lipids ratio in very small VLDL (%)	2342	0.12	0.03	0.21	0.008	2342	0.04	-0.01	0.09	0.100
Phospholipids to total lipds ratio in IDL (%)	2342	-0.15	-0.24	-0.06	0.001	2342	-0.04	-0.09	0.01	0.094
Total cholesterol to total lipids ratio in IDL (%)	2342	0.03	-0.06	0.12	0.505	2342	0.01	-0.04	0.05	0.719
Cholesterol esters to total lipids ratio in IDL (%)	2342	0.12	0.04	0.21	0.005	2342	0.04	-0.01	0.08	0.083
Free cholesterol to total lipids ratio in IDL (%)	2342	-0.22	-0.31	-0.12	<0.0001	2342	-0.07	-0.12	-0.02	0.004
Triglycerides to total lipids ratio in IDL (%)	2342	0.03	-0.06	0.12	0.567	2342	0.01	-0.04	0.05	0.791
Phospholipids to total lipds ratio in large LDL (%)	2342	0.02	-0.07	0.10	0.666	2342	0.00	-0.04	0.05	0.854
Total cholesterol to total lipids ratio in large LDL (%)	2342	-0.01	-0.11	0.08	0.806	2342	0.00	-0.05	0.05	0.998
Cholesterol esters to total lipids ratio in large LDL (%)	2342	0.05	-0.04	0.14	0.282	2342	0.02	-0.03	0.07	0.394
Free cholesterol to total lipids ratio in large LDL (%)	2342	-0.19	-0.27	-0.10	<0.0001	2342	-0.06	-0.11	-0.01	0.017
Triglycerides to total lipids ratio in large LDL (%)	2342	0.00	-0.09	0.09	0.948	2342	0.00	-0.05	0.04	0.842
Phospholipids to total lipds ratio in medium LDL (%)	2342	0.02	-0.07	0.11	0.696	2342	0.00	-0.05	0.04	0.852
Total cholesterol to total lipids ratio in medium LDL (%)	2342	0.00	-0.09	0.09	0.969	2342	0.01	-0.03	0.06	0.619
Cholesterol esters to total lipids ratio in medium LDL (%)	2342	0.03	-0.06	0.12	0.572	2342	0.02	-0.03	0.07	0.402
Free cholesterol to total lipids ratio in medium LDL (%)	2342	-0.06	-0.14	0.02	0.169	2342	-0.03	-0.08	0.02	0.193
Triglycerides to total lipids ratio in medium LDL (%)	2342	-0.04	-0.12	0.04	0.350	2342	-0.01	-0.05	0.03	0.605
Phospholipids to total lipds ratio in small LDL (%)	2342	0.00	-0.08	0.09	0.938	2342	-0.01	-0.05	0.04	0.742
Total cholesterol to total lipids ratio in small LDL (%)	2342	-0.04	-0.13	0.05	0.419	2342	0.00	-0.05	0.04	0.835
Cholesterol esters to total lipids ratio in small LDL (%)	2342	0.00	-0.09	0.09	0.965	2342	0.01	-0.04	0.06	0.697
Free cholesterol to total lipids ratio in small LDL (%)	2342	-0.06	-0.14	0.02	0.170	2342	-0.03	-0.08	0.01	0.148
Triglycerides to total lipids ratio in small LDL (%)	2342	0.10	0.01	0.19	0.030	2342	0.03	-0.01	0.08	0.150
Phospholipids to total lipds ratio in very large HDL (%)	2342	-0.23	-0.32	-0.14	<0.0001	2342	-0.07	-0.11	-0.02	0.002
Total cholesterol to total lipids ratio in very large HDL (%)	2342	0.20	0.11	0.29	<0.0001	2342	0.05	0.01	0.09	0.020
Cholesterol esters to total lipids ratio in very large HDL (%)	2342	0.20	0.12	0.29	<0.0001	2342	0.05	0.01	0.09	0.017
Free cholesterol to total lipids ratio in very large HDL (%)	2342	-0.11	-0.19	-0.02	0.013	2342	-0.04	-0.09	0.01	0.096
Triglycerides to total lipids ratio in very large HDL (%)	2342	0.21	0.11	0.30	<0.0001	2342	0.10	0.05	0.15	0.0002
Phospholipids to total lipds ratio in large HDL (%)	2342	0.25	0.16	0.34	<0.0001	2342	0.10	0.05	0.15	0.0001
Total cholesterol to total lipids ratio in large HDL (%)	2342	-0.27	-0.37	-0.18	<0.0001	2342	-0.12	-0.17	-0.06	<0.0001
Cholesterol esters to total lipids ratio in large HDL (%)	2342	-0.25	-0.35	-0.16	<0.0001	2342	-0.11	-0.16	-0.06	<0.0001
Free cholesterol to total lipids ratio in large HDL (%)	2342	-0.28	-0.37	-0.19	<0.0001	2342	-0.11	-0.16	-0.06	<0.0001
Triglycerides to total lipids ratio in large HDL (%)	2342	0.24	0.15	0.33	<0.0001	2342	0.12	0.07	0.17	<0.0001
Phospholipids to total lipds ratio in medium HDL (%)	2342	-0.01	-0.10	0.08	0.876	2342	-0.03	-0.08	0.01	0.178
Total cholesterol to total lipids ratio in medium HDL (%)	2342	-0.11	-0.21	-0.02	0.014	2342	-0.03	-0.08	0.01	0.173
Cholesterol esters to total lipids ratio in medium HDL (%)	2342	-0.12	-0.21	-0.03	0.008	2342	-0.04	-0.08	0.01	0.139
Free cholesterol to total lipids ratio in medium HDL (%)	2342	-0.03	-0.11	0.06	0.523	2342	0.00	-0.05	0.04	0.868
Triglycerides to total lipids ratio in medium HDL (%)	2342	0.25	0.16	0.35	<0.0001	2342	0.12	0.07	0.17	<0.0001
Phospholipids to total lipds ratio in small HDL (%)	2342	0.12	0.04	0.21	0.004	2342	0.06	0.02	0.10	0.003
Total cholesterol to total lipids ratio in small HDL (%)	2342	-0.17	-0.25	-0.09	<0.0001	2342	-0.09	-0.13	-0.05	<0.0001
Cholesterol esters to total lipids ratio in small HDL (%)	2342	-0.15	-0.24	-0.07	0.0003	2342	-0.08	-0.12	-0.03	0.0004
Free cholesterol to total lipids ratio in small HDL (%)	2342	-0.03	-0.12	0.07	0.561	2342	-0.04	-0.09	0.01	0.116
Triglycerides to total lipids ratio in small HDL (%)	2342	0.19	0.09	0.28	<0.0001	2342	0.10	0.04	0.15	0.0005
Mean diameter for VLDL particles (nm)	2342	0.27	0.18	0.36	<0.0001	2342	0.09	0.04	0.14	0.0002
Mean diameter for LDL particles (nm)	2342	-0.16	-0.25	-0.07	0.001	2342	0.01	-0.04	0.05	0.793

Online Table 8 Associations of change in lean mass index from age 10-18y with cardiometabolic traits at age 18y in ALSPAC

Change from age 10-18y

Lean mass index (per SD-unit gain)

Standardized outcome at age 18y	Adj. for age, sex, ethnicity, maternal education, lean mass index at 10y					Additionally adj. for fat mass index change				
	N	Beta	LCL	UCL	P-value	N	Beta	LCL	UCL	P-value
Mean diameter for HDL particles (nm)	2342	-0.21	-0.30	-0.13	<0.0001	2342	-0.08	-0.13	-0.04	0.0004
Serum total cholesterol (mmol/l)	2342	0.02	-0.06	0.10	0.657	2342	-0.01	-0.05	0.04	0.752
Total cholesterol in VLDL (mmol/l)	2342	0.22	0.13	0.31	<0.0001	2342	0.07	0.02	0.13	0.005
Remnant cholesterol (non-HDL, non-LDL -cholesterol) (mmol/l)	2342	0.14	0.05	0.23	0.003	2342	0.04	-0.01	0.09	0.083
Total cholesterol in LDL (mmol/l)	2342	0.02	-0.07	0.10	0.714	2342	0.00	-0.05	0.05	0.976
Total cholesterol in HDL (mmol/l)	2342	-0.13	-0.22	-0.05	0.001	2342	-0.07	-0.12	-0.03	0.001
Total cholesterol in HDL2 (mmol/l)	2342	-0.17	-0.25	-0.08	<0.0001	2342	-0.08	-0.13	-0.04	0.0003
Total cholesterol in HDL3 (mmol/l)	2342	-0.07	-0.15	0.00	0.065	2342	-0.05	-0.10	-0.01	0.016
Esterified cholesterol (mmol/l)	2332	0.02	-0.06	0.10	0.638	2332	-0.01	-0.05	0.04	0.772
Free cholesterol (mmol/l)	2330	0.02	-0.07	0.10	0.688	2330	0.00	-0.05	0.04	0.866
Serum total triglycerides (mmol/l)	2342	0.25	0.17	0.34	<0.0001	2342	0.09	0.04	0.14	0.001
Triglycerides in VLDL (mmol/l)	2342	0.28	0.19	0.37	<0.0001	2342	0.10	0.04	0.15	0.0003
Triglycerides in LDL (mmol/l)	2342	0.02	-0.06	0.09	0.622	2342	0.01	-0.04	0.05	0.825
Triglycerides in HDL (mmol/l)	2342	0.20	0.12	0.29	<0.0001	2342	0.08	0.03	0.13	0.002
Diacylglycerol (mmol/l)	2276	0.24	0.15	0.33	<0.0001	2276	0.09	0.03	0.14	0.001
Ratio of diacylglycerol to triglycerides	2277	0.13	0.04	0.23	0.004	2277	0.04	-0.01	0.08	0.125
Total phosphoglycerides (mmol/l)	2330	0.03	-0.05	0.10	0.481	2330	0.01	-0.04	0.05	0.804
Ratio of triglycerides to phosphoglycerides	2330	0.26	0.17	0.35	<0.0001	2330	0.11	0.06	0.16	<0.0001
Phosphatidylcholine and other cholines (mmol/l)	2314	0.06	-0.02	0.13	0.121	2314	0.00	-0.05	0.04	0.973
Total cholines (mmol/l)	2332	0.04	-0.04	0.11	0.351	2332	0.00	-0.05	0.04	0.870
Apolipoprotein A-I (g/l)	2342	-0.06	-0.14	0.02	0.117	2342	-0.05	-0.09	-0.01	0.024
Apolipoprotein B (g/l)	2342	0.16	0.07	0.25	0.0004	2342	0.05	0.00	0.11	0.034
Ratio of apolipoprotein B to apolipoprotein A-I	2342	0.19	0.09	0.28	<0.0001	2342	0.08	0.03	0.14	0.001
Total fatty acids (mmol/l)	2332	0.13	0.05	0.21	0.001	2332	0.04	-0.01	0.09	0.128
Estimated description of fatty acid chain length, not actual carbon number	2333	0.03	-0.06	0.12	0.514	2333	0.04	-0.01	0.08	0.118
Estimated degree of unsaturation	2332	-0.06	-0.15	0.04	0.228	2332	-0.01	-0.06	0.04	0.711
22:6, docosahexaenoic acid (mmol/l)	2332	0.04	-0.04	0.12	0.337	2332	0.03	-0.02	0.08	0.212
18:2, linoleic acid (mmol/l)	2332	0.07	-0.01	0.15	0.088	2332	-0.01	-0.05	0.04	0.783
Conjugated linoleic acid (mmol/l)	2331	0.13	0.00	0.26	0.047	2331	0.05	0.00	0.09	0.052
Omega-3 fatty acids (mmol/l)	2332	0.14	0.05	0.22	0.002	2332	0.04	0.00	0.09	0.078
Omega-6 fatty acids (mmol/l)	2332	0.07	-0.01	0.15	0.081	2332	0.00	-0.04	0.05	0.888
Polyunsaturated fatty acids (mmol/l)	2332	0.08	0.00	0.16	0.043	2332	0.01	-0.04	0.06	0.706
Monounsaturated fatty acids; 16:1, 18:1 (mmol/l)	2332	0.11	0.03	0.19	0.010	2332	0.05	0.00	0.10	0.052
Saturated fatty acids (mmol/l)	2331	0.17	0.09	0.25	<0.0001	2331	0.04	-0.01	0.09	0.079
Ratio of 22:6 docosahexaenoic acid to total fatty acids (%)	2333	-0.03	-0.12	0.05	0.457	2333	0.02	-0.03	0.06	0.397
Ratio of 18:2 linoleic acid to total fatty acids (%)	2333	-0.12	-0.20	-0.03	0.009	2333	-0.08	-0.12	-0.03	0.003
Ratio of conjugated linoleic acid to total fatty acids (%)	2332	0.12	0.00	0.24	0.042	2332	0.04	-0.01	0.09	0.093
Ratio of omega-3 fatty acids to total fatty acids (%)	2333	0.06	-0.03	0.15	0.169	2333	0.02	-0.02	0.07	0.335
Ratio of omega-6 fatty acids to total fatty acids (%)	2333	-0.14	-0.23	-0.05	0.002	2333	-0.07	-0.12	-0.02	0.005
Ratio of polyunsaturated fatty acids to total fatty acids (%)	2333	-0.12	-0.21	-0.03	0.010	2333	-0.06	-0.11	-0.01	0.016
Ratio of monounsaturated fatty acids to total fatty acids (%)	2333	0.01	-0.08	0.09	0.892	2333	0.04	-0.01	0.09	0.117
Ratio of saturated fatty acids to total fatty acids (%)	2332	0.14	0.05	0.23	0.003	2332	0.02	-0.02	0.07	0.348
Insulin (mu/l)	2378	0.27	0.09	0.45	0.003	2378	0.13	0.04	0.22	0.003
Glucose (mmol/l)	2341	0.05	-0.05	0.14	0.342	2341	0.05	0.00	0.11	0.049
Lactate (mmol/l)	2341	-0.05	-0.13	0.03	0.213	2341	-0.04	-0.08	0.00	0.061
Pyruvate (mmol/l)	2341	0.01	-0.07	0.10	0.743	2341	0.04	-0.01	0.08	0.104
Citrate (mmol/l)	2341	-0.18	-0.27	-0.09	0.0001	2341	-0.07	-0.12	-0.02	0.004
Alanine (mmol/l)	2341	0.12	0.03	0.21	0.006	2341	0.04	-0.01	0.08	0.139
Glutamine (mmol/l)	2341	-0.11	-0.18	-0.03	0.006	2341	-0.04	-0.09	0.00	0.053
Histidine (mmol/l)	2341	0.06	-0.02	0.15	0.163	2341	0.03	-0.02	0.08	0.213
Isoleucine (mmol/l)	2341	0.25	0.15	0.35	<0.0001	2341	0.11	0.06	0.16	<0.0001
Leucine (mmol/l)	2341	0.28	0.19	0.37	<0.0001	2341	0.12	0.07	0.16	<0.0001
Valine (mmol/l)	2341	0.25	0.16	0.35	<0.0001	2341	0.13	0.09	0.18	<0.0001
Phenylalanine (mmol/l)	2341	0.23	0.14	0.32	<0.0001	2341	0.11	0.06	0.15	<0.0001
Tyrosine (mmol/l)	2341	0.18	0.10	0.27	<0.0001	2341	0.10	0.05	0.14	<0.0001
Acetate (mmol/l)	2340	-0.03	-0.11	0.05	0.409	2340	-0.05	-0.10	0.01	0.101
Acetoacetate (mmol/l)	2341	-0.09	-0.18	-0.01	0.025	2341	-0.08	-0.12	-0.03	0.0003
3-hydroxybutyrate (mmol/l)	2338	-0.12	-0.21	-0.03	0.008	2338	-0.09	-0.14	-0.05	<0.0001
Creatinine (mmol/l)	2341	0.39	0.31	0.48	<0.0001	2341	0.16	0.12	0.20	<0.0001
Albumin (signal area)	2342	-0.07	-0.16	0.01	0.102	2342	0.00	-0.04	0.04	0.979
Glycoprotein acetyls, mainly a1-acid glycoprotein (mmol/l)	2341	0.15	0.06	0.23	0.001	2341	0.09	0.04	0.14	0.001
C-reactive protein (mg/l)	2419	0.03	-0.04	0.10	0.444	2419	0.03	0.00	0.06	0.039

Change from age 10-18y

Lean mass index (per SD-unit gain)

Complete case sample

Standardized outcome at age 18y	Adj. for age, sex, ethnicity, maternal education, lean mass index at 10y					Additionally adj. for fat mass index change				
	N	Beta	LCL	UCL	P-value	N	Beta	LCL	UCL	P-value
Systolic blood pressure (mmHg)	1722	0.39	0.31	0.48	<0.0001	1722	0.38	0.29	0.47	<0.0001
Diastolic blood pressure (mmHg)	1722	0.04	-0.07	0.15	0.482	1722	0.01	-0.10	0.13	0.835
Concentration of chylomicrons and extremely large VLDL particles (mol/l)	1722	0.19	0.10	0.29	<0.0001	1722	0.14	0.05	0.23	0.004
Total lipids in chylomicrons and extremely large VLDL (mmol/l)	1722	0.19	0.10	0.29	<0.0001	1722	0.14	0.05	0.24	0.003
Phospholipids in chylomicrons and extremely large VLDL (mmol/l)	1722	0.19	0.09	0.28	<0.0001	1722	0.14	0.04	0.23	0.005
Total cholesterol in chylomicrons and extremely large VLDL (mmol/l)	1722	0.21	0.11	0.31	<0.0001	1722	0.16	0.06	0.25	0.002
Cholesterol esters in chylomicrons and extremely large VLDL (mmol/l)	1722	0.22	0.12	0.32	<0.0001	1722	0.17	0.07	0.27	0.001
Free cholesterol in chylomicrons and extremely large VLDL (mmol/l)	1722	0.19	0.10	0.28	<0.0001	1722	0.14	0.04	0.23	0.004
Triglycerides in chylomicrons and extremely large VLDL (mmol/l)	1722	0.19	0.10	0.28	<0.0001	1722	0.14	0.04	0.23	0.004
Concentration of very large VLDL particles (mol/l)	1722	0.21	0.12	0.30	<0.0001	1722	0.16	0.06	0.25	0.001
Total lipids in very large VLDL (mmol/l)	1722	0.21	0.12	0.31	<0.0001	1722	0.16	0.07	0.25	0.001
Phospholipids in very large VLDL (mmol/l)	1722	0.20	0.11	0.30	<0.0001	1722	0.15	0.06	0.25	0.002
Total cholesterol in very large VLDL (mmol/l)	1722	0.21	0.12	0.31	<0.0001	1722	0.16	0.06	0.26	0.001

Online Table 8 Associations of change in lean mass index from age 10-18y with cardiometabolic traits at age 18y in ALSPAC

Change from age 10-18y

Lean mass index (per SD-unit gain)

	Adj. for age, sex, ethnicity, maternal education, lean mass index at 10y					*Additionally adj. for fat mass index change*				
**Standardized outcome at age 18y**	**N**	**Beta**	**LCL**	**UCL**	**P-value**	**N**	**Beta**	**LCL**	**UCL**	**P-value**
Cholesterol esters in very large VLDL (mmol/l)	1722	0.23	0.13	0.32	<0.0001	1722	0.17	0.07	0.27	0.001
Free cholesterol in very large VLDL (mmol/l)	1722	0.20	0.11	0.30	<0.0001	1722	0.15	0.05	0.24	0.003
Triglycerides in very large VLDL (mmol/l)	1722	0.21	0.12	0.30	<0.0001	1722	0.16	0.07	0.26	0.001
Concentration of large VLDL particles (mol/l)	1722	0.22	0.12	0.32	<0.0001	1722	0.17	0.08	0.27	0.0005
Total lipids in large VLDL (mmol/l)	1722	0.22	0.13	0.32	<0.0001	1722	0.17	0.08	0.27	0.0005
Phospholipids in large VLDL (mmol/l)	1722	0.22	0.12	0.31	<0.0001	1722	0.17	0.07	0.26	0.001
Total cholesterol in large VLDL (mmol/l)	1722	0.23	0.13	0.32	<0.0001	1722	0.17	0.08	0.27	0.0005
Cholesterol esters in large VLDL (mmol/l)	1722	0.23	0.13	0.33	<0.0001	1722	0.18	0.08	0.28	0.0004
Free cholesterol in large VLDL (mmol/l)	1722	0.22	0.12	0.31	<0.0001	1722	0.17	0.07	0.26	0.001
Triglycerides in large VLDL (mmol/l)	1722	0.22	0.12	0.31	<0.0001	1722	0.17	0.07	0.27	0.000
Concentration of medium VLDL particles (mol/l)	1722	0.23	0.13	0.32	<0.0001	1722	0.18	0.08	0.27	0.0004
Total lipids in medium VLDL (mmol/l)	1722	0.23	0.13	0.33	<0.0001	1722	0.18	0.08	0.28	0.0004
Phospholipids in medium VLDL (mmol/l)	1722	0.22	0.12	0.32	<0.0001	1722	0.17	0.07	0.27	0.001
Total cholesterol in medium VLDL (mmol/l)	1722	0.23	0.13	0.32	<0.0001	1722	0.17	0.07	0.27	0.001
Cholesterol esters in medium VLDL (mmol/l)	1722	0.23	0.13	0.33	<0.0001	1722	0.17	0.07	0.27	0.001
Free cholesterol in medium VLDL (mmol/l)	1722	0.21	0.12	0.31	<0.0001	1722	0.16	0.07	0.26	0.001
Triglycerides in medium VLDL (mmol/l)	1722	0.23	0.13	0.33	<0.0001	1722	0.18	0.08	0.28	0.0003
Concentration of small VLDL particles (mol/l)	1722	0.20	0.11	0.30	<0.0001	1722	0.15	0.05	0.25	0.002
Total lipids in small VLDL (mmol/l)	1722	0.20	0.10	0.30	<0.0001	1722	0.14	0.05	0.24	0.004
Phospholipids in small VLDL (mmol/l)	1722	0.20	0.10	0.29	<0.0001	1722	0.15	0.05	0.25	0.003
Total cholesterol in small VLDL (mmol/l)	1722	0.17	0.07	0.27	0.001	1722	0.12	0.01	0.22	0.032
Cholesterol esters in small VLDL (mmol/l)	1722	0.15	0.05	0.26	0.005	1722	0.10	-0.01	0.21	0.076
Free cholesterol in small VLDL (mmol/l)	1722	0.18	0.09	0.28	0.0002	1722	0.14	0.04	0.23	0.007
Triglycerides in small VLDL (mmol/l)	1722	0.20	0.10	0.29	<0.0001	1722	0.15	0.05	0.25	0.003
Concentration of very small VLDL particles (mol/l)	1722	0.09	-0.01	0.19	0.075	1722	0.05	-0.05	0.16	0.297
Total lipids in very small VLDL (mmol/l)	1722	0.11	0.00	0.21	0.048	1722	0.06	-0.04	0.17	0.239
Phospholipids in very small VLDL (mmol/l)	1722	0.05	-0.05	0.16	0.288	1722	0.02	-0.08	0.12	0.676
Total cholesterol in very small VLDL (mmol/l)	1722	0.11	0.00	0.22	0.051	1722	0.07	-0.04	0.19	0.219
Cholesterol esters in very small VLDL (mmol/l)	1722	0.12	0.00	0.23	0.044	1722	0.07	-0.04	0.19	0.219
Free cholesterol in very small VLDL (mmol/l)	1722	0.09	-0.02	0.20	0.093	1722	0.06	-0.04	0.17	0.242
Triglycerides in very small VLDL (mmol/l)	1722	0.11	0.01	0.20	0.027	1722	0.07	-0.02	0.17	0.143
Concentration of IDL particles (mol/l)	1722	0.02	-0.08	0.12	0.737	1722	-0.01	-0.11	0.09	0.861
Total lipids in IDL (mmol/l)	1722	0.03	-0.07	0.13	0.548	1722	0.00	-0.10	0.11	0.959
Phospholipids in IDL (mmol/l)	1722	0.01	-0.09	0.11	0.892	1722	-0.02	-0.12	0.09	0.750
Total cholesterol in IDL (mmol/l)	1722	0.05	-0.06	0.15	0.371	1722	0.02	-0.09	0.12	0.765
Cholesterol esters in IDL (mmol/l)	1722	0.07	-0.04	0.18	0.201	1722	0.03	-0.08	0.14	0.549
Free cholesterol in IDL (mmol/l)	1722	0.00	-0.11	0.10	0.943	1722	-0.02	-0.13	0.08	0.655
Triglycerides in IDL (mmol/l)	1722	-0.02	-0.11	0.07	0.655	1722	-0.03	-0.12	0.06	0.495
Concentration of large LDL particles (mol/l)	1722	0.02	-0.08	0.12	0.660	1722	0.00	-0.10	0.10	0.946
Total lipids in large LDL (mmol/l)	1722	0.03	-0.07	0.13	0.606	1722	0.00	-0.10	0.10	0.991
Phospholipids in large LDL (mmol/l)	1722	0.04	-0.06	0.14	0.418	1722	0.01	-0.09	0.11	0.827
Total cholesterol in large LDL (mmol/l)	1722	0.03	-0.07	0.13	0.555	1722	0.00	-0.10	0.11	0.963
Cholesterol esters in large LDL (mmol/l)	1722	0.04	-0.06	0.14	0.447	1722	0.01	-0.09	0.11	0.860
Free cholesterol in large LDL (mmol/l)	1722	0.00	-0.10	0.10	0.963	1722	-0.02	-0.12	0.08	0.712
Triglycerides in large LDL (mmol/l)	1722	-0.04	-0.13	0.05	0.364	1722	-0.05	-0.14	0.04	0.317
Concentration of medium LDL particles (mol/l)	1722	0.03	-0.06	0.13	0.480	1722	0.01	-0.09	0.11	0.889
Total lipids in medium LDL (mmol/l)	1722	0.04	-0.06	0.13	0.469	1722	0.01	-0.09	0.11	0.870
Phospholipids in medium LDL (mmol/l)	1722	0.06	-0.03	0.16	0.193	1722	0.03	-0.07	0.13	0.532
Total cholesterol in medium LDL (mmol/l)	1722	0.04	-0.06	0.14	0.444	1722	0.01	-0.09	0.11	0.838
Cholesterol esters in medium LDL (mmol/l)	1722	0.04	-0.06	0.14	0.428	1722	0.01	-0.09	0.11	0.826
Free cholesterol in medium LDL (mmol/l)	1722	0.03	-0.07	0.13	0.509	1722	0.01	-0.09	0.11	0.876
Triglycerides in medium LDL (mmol/l)	1722	-0.06	-0.15	0.03	0.192	1722	-0.06	-0.15	0.03	0.169
Concentration of small LDL particles (mol/l)	1722	0.04	-0.06	0.14	0.401	1722	0.02	-0.08	0.11	0.751
Total lipids in small LDL (mmol/l)	1722	0.04	-0.06	0.14	0.398	1722	0.01	-0.09	0.11	0.777
Phospholipids in small LDL (mmol/l)	1722	0.06	-0.03	0.16	0.192	1722	0.04	-0.06	0.13	0.473
Total cholesterol in small LDL (mmol/l)	1722	0.04	-0.06	0.14	0.480	1722	0.01	-0.09	0.11	0.868
Cholesterol esters in small LDL (mmol/l)	1722	0.03	-0.07	0.13	0.529	1722	0.00	-0.10	0.11	0.936
Free cholesterol in small LDL (mmol/l)	1722	0.05	-0.05	0.16	0.298	1722	0.03	-0.07	0.13	0.575
Triglycerides in small LDL (mmol/l)	1722	0.02	-0.07	0.11	0.654	1722	0.00	-0.09	0.09	0.997
Concentration of very large HDL particles (mol/l)	1722	-0.20	-0.30	-0.10	0.0001	1722	-0.17	-0.27	-0.06	0.001
Total lipids in very large HDL (mmol/l)	1722	-0.19	-0.30	-0.09	0.0003	1722	-0.16	-0.26	-0.05	0.003
Phospholipids in very large HDL (mmol/l)	1722	-0.21	-0.31	-0.11	<0.0001	1722	-0.18	-0.28	-0.08	0.000
Total cholesterol in very large HDL (mmol/l)	1722	-0.16	-0.27	-0.05	0.005	1722	-0.13	-0.24	-0.02	0.021
Cholesterol esters in very large HDL (mmol/l)	1722	-0.14	-0.25	-0.03	0.014	1722	-0.11	-0.23	0.00	0.044
Free cholesterol in very large HDL (mmol/l)	1722	-0.19	-0.30	-0.09	0.0003	1722	-0.16	-0.26	-0.06	0.003
Triglycerides in very large HDL (mmol/l)	1722	-0.01	-0.12	0.09	0.776	1722	-0.02	-0.12	0.08	0.681
Concentration of large HDL particles (mol/l)	1722	-0.17	-0.26	-0.07	0.001	1722	-0.12	-0.22	-0.03	0.010
Total lipids in large HDL (mmol/l)	1722	-0.18	-0.27	-0.08	0.0003	1722	-0.13	-0.23	-0.04	0.006
Phospholipids in large HDL (mmol/l)	1722	-0.16	-0.25	-0.06	0.001	1722	-0.12	-0.21	-0.02	0.013
Total cholesterol in large HDL (mmol/l)	1722	-0.20	-0.30	-0.10	<0.0001	1722	-0.15	-0.25	-0.05	0.003
Cholesterol esters in large HDL (mmol/l)	1722	-0.20	-0.30	-0.10	<0.0001	1722	-0.15	-0.25	-0.05	0.003
Free cholesterol in large HDL (mmol/l)	1722	-0.19	-0.29	-0.09	0.0001	1722	-0.15	-0.25	-0.05	0.003
Triglycerides in large HDL (mmol/l)	1722	0.03	-0.06	0.12	0.495	1722	0.03	-0.06	0.12	0.486
Concentration of medium HDL particles (mol/l)	1722	0.02	-0.08	0.11	0.702	1722	0.04	-0.06	0.13	0.462
Total lipids in medium HDL (mmol/l)	1722	0.00	-0.10	0.09	0.962	1722	0.02	-0.08	0.11	0.727
Phospholipids in medium HDL (mmol/l)	1722	-0.01	-0.10	0.08	0.823	1722	0.01	-0.08	0.10	0.847
Total cholesterol in medium HDL (mmol/l)	1722	-0.02	-0.13	0.08	0.646	1722	0.00	-0.11	0.11	0.990
Cholesterol esters in medium HDL (mmol/l)	1722	-0.03	-0.14	0.08	0.576	1722	0.00	-0.11	0.10	0.952
Free cholesterol in medium HDL (mmol/l)	1722	0.00	-0.10	0.10	0.996	1722	0.02	-0.08	0.12	0.747
Triglycerides in medium HDL (mmol/l)	1722	0.21	0.11	0.30	<0.0001	1722	0.17	0.07	0.27	0.001
Concentration of small HDL particles (mol/l)	1722	0.10	0.00	0.20	0.057	1722	0.10	0.00	0.20	0.061
Total lipids in small HDL (mmol/l)	1722	0.05	-0.05	0.14	0.317	1722	0.05	-0.04	0.15	0.271
Phospholipids in small HDL (mmol/l)	1722	0.10	-0.01	0.21	0.076	1722	0.11	0.00	0.22	0.061
Total cholesterol in small HDL (mmol/l)	1722	-0.04	-0.12	0.05	0.408	1722	-0.03	-0.11	0.06	0.524
Cholesterol esters in small HDL (mmol/l)	1722	-0.05	-0.13	0.03	0.252	1722	-0.04	-0.13	0.04	0.290
Free cholesterol in small HDL (mmol/l)	1722	0.02	-0.08	0.13	0.664	1722	0.05	-0.06	0.15	0.404

Online Table 8 Associations of change in lean mass index from age 10-18y with cardiometabolic traits at age 18y in ALSPAC

Change from age 10-18y

Lean mass index (per SD-unit gain)

	Adj. for age, sex, ethnicity, maternal education, lean mass index at 10y					*Additionally adj. for fat mass index change*				
**Standardized outcome at age 18y**	**N**	**Beta**	**LCL**	**UCL**	**P-value**	**N**	**Beta**	**LCL**	**UCL**	**P-value**
Triglycerides in small HDL (mmol/l)	1722	0.14	0.04	0.24	0.005	1722	0.11	0.01	0.21	0.025
Phospholipids to total lipds ratio in chylomicrons and extremely large VLDL (%)	1722	-0.02	-0.08	0.03	0.402	1722	-0.02	-0.07	0.04	0.541
Total cholesterol to total lipids ratio in chylomicrons and extremely large VLDL (%)	1722	0.14	0.03	0.24	0.010	1722	0.11	0.00	0.22	0.045
Cholesterol esters to total lipids ratio in chylomicrons and extremely large VLDL (%)	1722	0.12	0.01	0.23	0.040	1722	0.09	-0.02	0.20	0.125
Free cholesterol to total lipids ratio in chylomicrons and extremely large VLDL (%)	1722	0.06	-0.03	0.16	0.195	1722	0.06	-0.04	0.15	0.268
Triglycerides to total lipids ratio in chylomicrons and extremely large VLDL (%)	1722	-0.12	-0.22	-0.02	0.020	1722	-0.10	-0.20	0.01	0.063
Phospholipids to total lipds ratio in very large VLDL (%)	1722	0.11	0.01	0.20	0.026	1722	0.08	-0.01	0.18	0.095
Total cholesterol to total lipids ratio in very large VLDL (%)	1722	-0.02	-0.12	0.08	0.712	1722	-0.01	-0.12	0.09	0.780
Cholesterol esters to total lipids ratio in very large VLDL (%)	1722	0.01	-0.07	0.08	0.863	1722	0.01	-0.06	0.08	0.806
Free cholesterol to total lipids ratio in very large VLDL (%)	1722	0.02	-0.06	0.10	0.573	1722	0.03	-0.05	0.11	0.524
Triglycerides to total lipids ratio in very large VLDL (%)	1722	-0.08	-0.19	0.02	0.117	1722	-0.08	-0.18	0.03	0.162
Phospholipids to total lipds ratio in large VLDL (%)	1722	0.06	-0.04	0.15	0.242	1722	0.04	-0.06	0.13	0.459
Total cholesterol to total lipids ratio in large VLDL (%)	1722	0.15	0.02	0.28	0.020	1722	0.12	-0.01	0.25	0.071
Cholesterol esters to total lipids ratio in large VLDL (%)	1722	0.11	-0.03	0.25	0.134	1722	0.09	-0.06	0.24	0.217
Free cholesterol to total lipids ratio in large VLDL (%)	1722	0.05	0.00	0.09	0.029	1722	0.03	-0.01	0.08	0.123
Triglycerides to total lipids ratio in large VLDL (%)	1722	-0.13	-0.24	-0.02	0.024	1722	-0.10	-0.21	0.01	0.085
Phospholipids to total lipds ratio in medium VLDL (%)	1722	-0.12	-0.22	-0.02	0.018	1722	-0.09	-0.19	0.01	0.084
Total cholesterol to total lipids ratio in medium VLDL (%)	1722	0.09	-0.01	0.19	0.081	1722	0.07	-0.03	0.17	0.184
Cholesterol esters to total lipids ratio in medium VLDL (%)	1722	0.09	-0.01	0.20	0.090	1722	0.07	-0.04	0.18	0.196
Free cholesterol to total lipids ratio in medium VLDL (%)	1722	0.05	-0.04	0.14	0.244	1722	0.04	-0.05	0.13	0.354
Triglycerides to total lipids ratio in medium VLDL (%)	1722	-0.06	-0.16	0.04	0.252	1722	-0.05	-0.15	0.06	0.375
Phospholipids to total lipds ratio in small VLDL (%)	1722	-0.12	-0.22	-0.01	0.024	1722	-0.07	-0.17	0.03	0.185
Total cholesterol to total lipids ratio in small VLDL (%)	1722	-0.03	-0.15	0.08	0.562	1722	-0.05	-0.16	0.07	0.430
Cholesterol esters to total lipids ratio in small VLDL (%)	1722	-0.02	-0.13	0.09	0.722	1722	-0.04	-0.15	0.07	0.506
Free cholesterol to total lipids ratio in small VLDL (%)	1722	-0.09	-0.18	0.00	0.052	1722	-0.05	-0.14	0.04	0.233
Triglycerides to total lipids ratio in small VLDL (%)	1722	0.07	-0.03	0.18	0.178	1722	0.07	-0.04	0.18	0.206
Phospholipids to total lipds ratio in very small VLDL (%)	1722	-0.06	-0.15	0.03	0.213	1722	-0.06	-0.15	0.04	0.222
Total cholesterol to total lipids ratio in very small VLDL (%)	1722	0.02	-0.08	0.11	0.712	1722	0.02	-0.08	0.11	0.705
Cholesterol esters to total lipids ratio in very small VLDL (%)	1722	0.03	-0.06	0.12	0.545	1722	0.02	-0.08	0.11	0.705
Free cholesterol to total lipids ratio in very small VLDL (%)	1722	-0.03	-0.12	0.06	0.556	1722	0.01	-0.08	0.10	0.817
Triglycerides to total lipids ratio in very small VLDL (%)	1722	0.02	-0.09	0.12	0.746	1722	0.02	-0.09	0.12	0.759
Phospholipids to total lipds ratio in IDL (%)	1722	-0.19	-0.29	-0.09	0.0002	1722	-0.15	-0.25	-0.05	0.004
Total cholesterol to total lipids ratio in IDL (%)	1722	0.13	0.03	0.24	0.013	1722	0.10	0.00	0.21	0.049
Cholesterol esters to total lipids ratio in IDL (%)	1722	0.20	0.10	0.30	<0.0001	1722	0.16	0.06	0.26	0.002
Free cholesterol to total lipids ratio in IDL (%)	1722	-0.15	-0.25	-0.04	0.005	1722	-0.12	-0.22	-0.01	0.025
Triglycerides to total lipids ratio in IDL (%)	1722	-0.08	-0.18	0.03	0.150	1722	-0.06	-0.17	0.04	0.253
Phospholipids to total lipds ratio in large LDL (%)	1722	-0.01	-0.11	0.10	0.912	1722	0.00	-0.10	0.11	0.950
Total cholesterol to total lipids ratio in large LDL (%)	1722	0.07	-0.04	0.18	0.211	1722	0.05	-0.06	0.16	0.375
Cholesterol esters to total lipids ratio in large LDL (%)	1722	0.11	0.00	0.21	0.051	1722	0.08	-0.03	0.19	0.151
Free cholesterol to total lipids ratio in large LDL (%)	1722	-0.14	-0.24	-0.05	0.003	1722	-0.11	-0.21	-0.02	0.018
Triglycerides to total lipids ratio in large LDL (%)	1722	-0.11	-0.21	0.00	0.045	1722	-0.09	-0.19	0.02	0.114
Phospholipids to total lipds ratio in medium LDL (%)	1722	-0.02	-0.12	0.09	0.730	1722	-0.01	-0.12	0.09	0.837
Total cholesterol to total lipids ratio in medium LDL (%)	1722	0.07	-0.04	0.18	0.205	1722	0.06	-0.05	0.17	0.319
Cholesterol esters to total lipids ratio in medium LDL (%)	1722	0.08	-0.03	0.18	0.166	1722	0.06	-0.05	0.16	0.291
Free cholesterol to total lipids ratio in medium LDL (%)	1722	-0.07	-0.16	0.03	0.172	1722	-0.05	-0.15	0.05	0.325
Triglycerides to total lipids ratio in medium LDL (%)	1722	-0.12	-0.22	-0.03	0.008	1722	-0.11	-0.20	-0.01	0.023
Phospholipids to total lipds ratio in small LDL (%)	1722	-0.03	-0.14	0.07	0.507	1722	-0.02	-0.12	0.08	0.707
Total cholesterol to total lipids ratio in small LDL (%)	1722	0.03	-0.08	0.14	0.561	1722	0.02	-0.09	0.13	0.729
Cholesterol esters to total lipids ratio in small LDL (%)	1722	0.05	-0.06	0.15	0.399	1722	0.03	-0.08	0.14	0.588
Free cholesterol to total lipids ratio in small LDL (%)	1722	-0.05	-0.15	0.04	0.265	1722	-0.04	-0.13	0.06	0.463
Triglycerides to total lipids ratio in small LDL (%)	1722	-0.01	-0.11	0.09	0.872	1722	-0.01	-0.11	0.09	0.823
Phospholipids to total lipds ratio in very large HDL (%)	1722	-0.23	-0.32	-0.13	<0.0001	1722	-0.19	-0.29	-0.10	<0.0001
Total cholesterol to total lipids ratio in very large HDL (%)	1722	0.20	0.11	0.30	<0.0001	1722	0.17	0.08	0.27	0.0003
Cholesterol esters to total lipids ratio in very large HDL (%)	1722	0.21	0.11	0.30	<0.0001	1722	0.18	0.08	0.27	0.0003
Free cholesterol to total lipids ratio in very large HDL (%)	1722	-0.11	-0.22	-0.01	0.030	1722	-0.10	-0.21	0.00	0.057
Triglycerides to total lipids ratio in very large HDL (%)	1722	0.18	0.07	0.29	0.002	1722	0.14	0.03	0.25	0.014
Phospholipids to total lipds ratio in large HDL (%)	1722	0.22	0.12	0.33	<0.0001	1722	0.17	0.07	0.28	0.001
Total cholesterol to total lipids ratio in large HDL (%)	1722	-0.24	-0.35	-0.14	<0.0001	1722	-0.19	-0.30	-0.08	0.001
Cholesterol esters to total lipids ratio in large HDL (%)	1722	-0.24	-0.35	-0.13	<0.0001	1722	-0.18	-0.29	-0.07	0.001
Free cholesterol to total lipids ratio in large HDL (%)	1722	-0.20	-0.31	-0.10	0.0001	1722	-0.17	-0.27	-0.06	0.002
Triglycerides to total lipids ratio in large HDL (%)	1722	0.22	0.12	0.32	<0.0001	1722	0.17	0.07	0.28	0.001
Phospholipids to total lipds ratio in medium HDL (%)	1722	-0.05	-0.16	0.06	0.389	1722	-0.04	-0.16	0.07	0.474
Total cholesterol to total lipids ratio in medium HDL (%)	1722	-0.06	-0.17	0.05	0.282	1722	-0.04	-0.15	0.07	0.448
Cholesterol esters to total lipids ratio in medium HDL (%)	1722	-0.07	-0.18	0.04	0.198	1722	-0.05	-0.16	0.06	0.366
Free cholesterol to total lipids ratio in medium HDL (%)	1722	0.02	-0.09	0.12	0.772	1722	0.01	-0.10	0.11	0.872
Triglycerides to total lipids ratio in medium HDL (%)	1722	0.21	0.10	0.33	0.0002	1722	0.16	0.05	0.28	0.005
Phospholipids to total lipds ratio in small HDL (%)	1722	0.10	0.00	0.20	0.046	1722	0.10	0.00	0.20	0.043
Total cholesterol to total lipids ratio in small HDL (%)	1722	-0.13	-0.23	-0.04	0.007	1722	-0.12	-0.22	-0.03	0.012
Cholesterol esters to total lipids ratio in small HDL (%)	1722	-0.11	-0.21	-0.02	0.022	1722	-0.11	-0.21	-0.02	0.023
Free cholesterol to total lipids ratio in small HDL (%)	1722	-0.05	-0.16	0.06	0.363	1722	0.00	-0.12	0.11	0.951
Triglycerides to total lipids ratio in small HDL (%)	1722	0.12	0.01	0.23	0.033	1722	0.08	-0.03	0.20	0.138
Mean diameter for VLDL particles (nm)	1722	0.21	0.11	0.31	<0.0001	1722	0.17	0.07	0.27	0.001
Mean diameter for LDL particles (nm)	1722	-0.11	-0.22	0.00	0.041	1722	-0.10	-0.21	0.00	0.057
Mean diameter for HDL particles (nm)	1722	-0.21	-0.32	-0.11	<0.0001	1722	-0.17	-0.28	-0.07	0.001
Serum total cholesterol (mmol/l)	1722	0.03	-0.07	0.13	0.572	1722	0.01	-0.09	0.11	0.909
Total cholesterol in VLDL (mmol/l)	1722	0.21	0.10	0.31	<0.0001	1722	0.15	0.04	0.25	0.005
Remnant cholesterol (non-HDL, non-LDL -cholesterol) (mmol/l)	1722	0.15	0.04	0.25	0.005	1722	0.10	-0.01	0.21	0.070
Total cholesterol in LDL (mmol/l)	1722	0.03	-0.07	0.13	0.505	1722	0.01	-0.10	0.11	0.906
Total cholesterol in HDL (mmol/l)	1722	-0.15	-0.24	-0.05	0.003	1722	-0.11	-0.21	-0.01	0.027
Total cholesterol in HDL2 (mmol/l)	1722	-0.17	-0.27	-0.07	0.001	1722	-0.13	-0.23	-0.03	0.011
Total cholesterol in HDL3 (mmol/l)	1722	-0.10	-0.19	0.00	0.047	1722	-0.07	-0.17	0.02	0.146
Esterified cholesterol (mmol/l)	1722	0.04	-0.07	0.14	0.486	1722	0.01	-0.09	0.12	0.800
Free cholesterol (mmol/l)	1722	0.01	-0.09	0.10	0.877	1722	-0.01	-0.11	0.09	0.805
Serum total triglycerides (mmol/l)	1722	0.18	0.09	0.28	0.0002	1722	0.14	0.04	0.23	0.005
Triglycerides in VLDL (mmol/l)	1722	0.21	0.12	0.31	<0.0001	1722	0.17	0.07	0.26	0.001
Triglycerides in LDL (mmol/l)	1722	-0.04	-0.12	0.05	0.427	1722	-0.04	-0.13	0.05	0.345

Online Table 8 Associations of change in lean mass index from age 10-18y with cardiometabolic traits at age 18y in ALSPAC

Change from age 10-18y

Lean mass index (per SD-unit gain)

	Adj. for age, sex, ethnicity, maternal education, lean mass index at 10y					*Additionally adj. for fat mass index change*				
**Standardized outcome at age 18y**	**N**	**Beta**	**LCL**	**UCL**	**P-value**	**N**	**Beta**	**LCL**	**UCL**	**P-value**
Triglycerides in HDL (mmol/l)	1722	0.14	0.05	0.23	0.003	1722	0.11	0.02	0.21	0.019
Diacylglycerol (mmol/l)	1722	0.20	0.10	0.30	<0.0001	1722	0.16	0.06	0.26	0.001
Ratio of diacylglycerol to triglycerides	1722	0.15	0.04	0.25	0.008	1722	0.14	0.03	0.24	0.012
Total phosphoglycerides (mmol/l)	1722	0.01	-0.08	0.10	0.832	1722	0.01	-0.08	0.10	0.805
Ratio of triglycerides to phosphoglycerides	1722	0.19	0.09	0.29	0.0002	1722	0.14	0.04	0.24	0.006
Phosphatidylcholine and other cholines (mmol/l)	1722	0.03	-0.06	0.12	0.478	1722	0.03	-0.05	0.12	0.445
Total cholines (mmol/l)	1722	0.01	-0.08	0.10	0.782	1722	0.01	-0.08	0.10	0.786
Apolipoprotein A-I (g/l)	1722	-0.08	-0.18	0.01	0.082	1722	-0.06	-0.16	0.03	0.181
Apolipoprotein B (g/l)	1722	0.15	0.05	0.25	0.005	1722	0.09	-0.01	0.20	0.069
Ratio of apolipoprotein B to apolipoprotein A-I	1722	0.19	0.08	0.29	0.001	1722	0.13	0.02	0.24	0.021
Total fatty acids (mmol/l)	1722	0.09	0.00	0.18	0.044	1722	0.07	-0.03	0.16	0.167
Estimated description of fatty acid chain length, not actual carbon number	1722	-0.05	-0.15	0.05	0.345	1722	-0.05	-0.15	0.05	0.306
Estimated degree of unsaturation	1722	-0.06	-0.17	0.04	0.214	1722	-0.05	-0.16	0.05	0.293
22:6, docosahexaenoic acid (mmol/l)	1722	0.02	-0.08	0.11	0.719	1722	0.01	-0.09	0.10	0.908
18:2, linoleic acid (mmol/l)	1722	0.06	-0.03	0.15	0.171	1722	0.05	-0.04	0.14	0.289
Conjugated linoleic acid (mmol/l)	1722	0.00	-0.09	0.09	0.960	1722	-0.02	-0.11	0.07	0.655
Omega-3 fatty acids (mmol/l)	1722	0.11	0.01	0.21	0.032	1722	0.09	-0.01	0.19	0.083
Omega-6 fatty acids (mmol/l)	1722	0.06	-0.03	0.15	0.186	1722	0.04	-0.05	0.14	0.353
Polyunsaturated fatty acids (mmol/l)	1722	0.07	-0.02	0.16	0.132	1722	0.05	-0.04	0.15	0.275
Monounsaturated fatty acids; 16:1, 18:1 (mmol/l)	1722	0.05	-0.04	0.14	0.298	1722	0.01	-0.08	0.10	0.819
Saturated fatty acids (mmol/l)	1722	0.13	0.04	0.23	0.006	1722	0.11	0.01	0.21	0.025
Ratio of 22:6 docosahexaenoic acid to total fatty acids (%)	1722	-0.03	-0.13	0.07	0.513	1722	-0.03	-0.13	0.07	0.585
Ratio of 18:2 linoleic acid to total fatty acids (%)	1722	-0.06	-0.16	0.04	0.243	1722	-0.03	-0.13	0.07	0.518
Ratio of conjugated linoleic acid to total fatty acids (%)	1722	0.00	-0.09	0.09	0.976	1722	-0.01	-0.10	0.07	0.742
Ratio of omega-3 fatty acids to total fatty acids (%)	1722	0.07	-0.03	0.17	0.176	1722	0.07	-0.03	0.17	0.176
Ratio of omega-6 fatty acids to total fatty acids (%)	1722	-0.07	-0.17	0.03	0.159	1722	-0.05	-0.15	0.05	0.345
Ratio of polyunsaturated fatty acids to total fatty acids (%)	1722	-0.05	-0.15	0.05	0.311	1722	-0.03	-0.13	0.07	0.571
Ratio of monounsaturated fatty acids to total fatty acids (%)	1722	-0.06	-0.16	0.04	0.241	1722	-0.09	-0.20	0.01	0.074
Ratio of saturated fatty acids to total fatty acids (%)	1722	0.15	0.04	0.25	0.006	1722	0.17	0.06	0.27	0.002
Insulin (mu/l)	1722	0.22	0.08	0.36	0.002	1722	0.17	0.05	0.30	0.005
Glucose (mmol/l)	1722	0.05	-0.03	0.13	0.205	1722	0.03	-0.05	0.12	0.399
Lactate (mmol/l)	1722	-0.06	-0.16	0.04	0.265	1722	-0.05	-0.15	0.05	0.287
Pyruvate (mmol/l)	1722	-0.04	-0.14	0.06	0.435	1722	-0.05	-0.15	0.05	0.338
Citrate (mmol/l)	1722	-0.16	-0.27	-0.05	0.004	1722	-0.16	-0.27	-0.04	0.006
Alanine (mmol/l)	1722	0.10	0.00	0.20	0.056	1722	0.09	-0.01	0.19	0.091
Glutamine (mmol/l)	1722	-0.09	-0.18	0.00	0.050	1722	-0.09	-0.18	0.00	0.046
Histidine (mmol/l)	1722	0.05	-0.06	0.16	0.382	1722	0.05	-0.06	0.15	0.401
Isoleucine (mmol/l)	1722	0.23	0.14	0.32	<0.0001	1722	0.20	0.11	0.30	<0.0001
Leucine (mmol/l)	1722	0.26	0.18	0.34	<0.0001	1722	0.24	0.16	0.33	<0.0001
Valine (mmol/l)	1722	0.24	0.14	0.34	<0.0001	1722	0.22	0.13	0.32	<0.0001
Phenylalanine (mmol/l)	1722	0.22	0.12	0.32	<0.0001	1722	0.20	0.10	0.30	<0.0001
Tyrosine (mmol/l)	1722	0.16	0.06	0.26	0.002	1722	0.12	0.03	0.22	0.014
Acetate (mmol/l)	1722	-0.03	-0.09	0.02	0.207	1722	-0.03	-0.09	0.02	0.224
Acetoacetate (mmol/l)	1722	-0.07	-0.17	0.03	0.146	1722	-0.05	-0.16	0.05	0.308
3-hydroxybutyrate (mmol/l)	1722	-0.11	-0.23	0.00	0.048	1722	-0.09	-0.21	0.02	0.101
Creatinine (mmol/l)	1722	0.36	0.27	0.45	<0.0001	1722	0.36	0.27	0.45	<0.0001
Albumin (signal area)	1722	-0.06	-0.17	0.04	0.244	1722	-0.07	-0.18	0.04	0.241
Glycoprotein acetyls, mainly a1-acid glycoprotein (mmol/l)	1722	0.12	0.02	0.21	0.021	1722	0.07	-0.03	0.17	0.153
C-reactive protein (mg/l)	1722	0.01	-0.08	0.10	0.774	1722	0.00	-0.08	0.09	0.970

Online Table 9 Interactions between fat mass index and lean mass index in relation to cardiometabolic traits at age 18y in ALSPAC

Model 1 & 3 adjusted for lean mass index, age, age*lean mass index, sex, sex*lean mass index, ethnicity, ethnicity*lean mass index, education, education*lean mass index,
Model 2 additionally adjusted for smoking, smoking*lean mass index, alcohol, alcohol*lean mass index, puberty timing, puberty timing*lean mass index

	Model 1 Interaction of fat mass index at age 10y with lean mass index at age 10y					Model 2 Interaction of fat mass index at age 18y with lean mass index at age 18y					Model 3 Interaction of change in fat mass index (age 10-18y) with change in lean mass index (age 10-18y)				
**Standardized outcome at age 18y**	**N**	**Beta**	**LCI**	**UCI**	**P-value**	**N**	**Beta**	**LCI**	**UCI**	**P-value**	**N**	**Beta**	**LCI**	**UCI**	**P-value**
Systolic blood pressure (mmHg)	3548	0.01	-0.03	0.04	0.701	3016	-0.02	-0.06	0.02	0.445	3409	0.00	-0.05	0.05	0.992
Diastolic blood pressure (mmHg)	3548	0.05	0.01	0.08	0.008	3016	0.05	0.01	0.10	0.018	3409	0.06	0.00	0.12	0.063
Concentration of chylomicrons and extremely large VLDL particles (mol/l)	2440	0.06	0.00	0.11	0.041	2058	0.11	0.05	0.17	0.0001	2342	0.12	0.03	0.21	0.010
Total lipids in chylomicrons and extremely large VLDL (mmol/l)	2440	0.05	0.00	0.11	0.042	2058	0.11	0.05	0.17	0.0002	2342	0.12	0.03	0.21	0.010
Phospholipids in chylomicrons and extremely large VLDL (mmol/l)	2440	0.06	0.00	0.11	0.038	2058	0.11	0.05	0.17	0.0001	2342	0.12	0.03	0.21	0.011
Total cholesterol in chylomicrons and extremely large VLDL (mmol/l)	2440	0.06	0.00	0.11	0.047	2058	0.11	0.05	0.17	0.0002	2342	0.12	0.03	0.21	0.011
Cholesterol esters in chylomicrons and extremely large VLDL (mmol/l)	2440	0.05	0.00	0.11	0.065	2058	0.10	0.05	0.16	0.0004	2342	0.11	0.02	0.20	0.016
Free cholesterol in chylomicrons and extremely large VLDL (mmol/l)	2440	0.06	0.00	0.11	0.034	2058	0.11	0.06	0.17	0.0001	2342	0.12	0.03	0.21	0.010
Triglycerides in chylomicrons and extremely large VLDL (mmol/l)	2440	0.05	0.00	0.11	0.042	2058	0.11	0.05	0.17	0.0002	2342	0.12	0.03	0.21	0.010
Concentration of very large VLDL particles (mol/l)	2440	0.06	0.01	0.12	0.022	2058	0.12	0.06	0.17	0.0001	2342	0.12	0.03	0.21	0.007
Total lipids in very large VLDL (mmol/l)	2440	0.06	0.01	0.12	0.022	2058	0.11	0.06	0.17	0.0001	2342	0.12	0.03	0.21	0.007
Phospholipids in very large VLDL (mmol/l)	2440	0.06	0.01	0.12	0.022	2058	0.12	0.06	0.18	<0.0001	2342	0.12	0.03	0.21	0.009
Total cholesterol in very large VLDL (mmol/l)	2440	0.06	0.00	0.11	0.040	2058	0.11	0.05	0.17	0.0001	2342	0.12	0.03	0.21	0.009
Cholesterol esters in very large VLDL (mmol/l)	2440	0.06	0.00	0.11	0.045	2058	0.11	0.05	0.16	0.0002	2342	0.12	0.03	0.21	0.009
Free cholesterol in very large VLDL (mmol/l)	2440	0.06	0.00	0.11	0.036	2058	0.11	0.06	0.17	<0.0001	2342	0.12	0.03	0.21	0.010
Triglycerides in very large VLDL (mmol/l)	2440	0.06	0.01	0.12	0.018	2058	0.11	0.06	0.17	0.0001	2342	0.12	0.03	0.21	0.007
Concentration of large VLDL particles (mol/l)	2440	0.07	0.01	0.12	0.016	2058	0.11	0.05	0.17	0.0001	2342	0.12	0.04	0.21	0.005
Total lipids in large VLDL (mmol/l)	2440	0.07	0.01	0.12	0.017	2058	0.11	0.05	0.17	0.0002	2342	0.12	0.04	0.21	0.005
Phospholipids in large VLDL (mmol/l)	2440	0.07	0.01	0.12	0.017	2058	0.11	0.05	0.17	0.0001	2342	0.12	0.04	0.21	0.006
Total cholesterol in large VLDL (mmol/l)	2440	0.06	0.01	0.12	0.024	2058	0.11	0.05	0.16	0.0002	2342	0.12	0.03	0.21	0.008
Cholesterol esters in large VLDL (mmol/l)	2440	0.06	0.00	0.11	0.034	2058	0.10	0.05	0.16	0.0004	2342	0.11	0.03	0.20	0.011
Free cholesterol in large VLDL (mmol/l)	2440	0.07	0.01	0.12	0.018	2058	0.11	0.06	0.17	0.0001	2342	0.12	0.03	0.21	0.007
Triglycerides in large VLDL (mmol/l)	2440	0.07	0.01	0.12	0.015	2058	0.11	0.05	0.17	0.0002	2342	0.12	0.04	0.21	0.005
Concentration of medium VLDL particles (mol/l)	2440	0.06	0.01	0.12	0.023	2058	0.10	0.04	0.15	0.0004	2342	0.12	0.03	0.20	0.006
Total lipids in medium VLDL (mmol/l)	2440	0.06	0.01	0.11	0.028	2058	0.10	0.04	0.15	0.001	2342	0.12	0.03	0.20	0.008
Phospholipids in medium VLDL (mmol/l)	2440	0.06	0.01	0.11	0.029	2058	0.10	0.04	0.15	0.001	2342	0.11	0.03	0.20	0.009
Total cholesterol in medium VLDL (mmol/l)	2440	0.05	0.00	0.10	0.069	2058	0.09	0.03	0.14	0.001	2342	0.10	0.01	0.19	0.025
Cholesterol esters in medium VLDL (mmol/l)	2440	0.04	-0.01	0.09	0.155	2058	0.07	0.02	0.13	0.006	2342	0.08	-0.01	0.17	0.065
Free cholesterol in medium VLDL (mmol/l)	2440	0.06	0.01	0.11	0.024	2058	0.10	0.04	0.15	0.0004	2342	0.12	0.03	0.20	0.009
Triglycerides in medium VLDL (mmol/l)	2440	0.06	0.01	0.12	0.019	2058	0.10	0.04	0.15	0.001	2342	0.12	0.04	0.20	0.005
Concentration of small VLDL particles (mol/l)	2440	0.05	0.00	0.10	0.049	2058	0.08	0.03	0.13	0.002	2342	0.10	0.02	0.18	0.020
Total lipids in small VLDL (mmol/l)	2440	0.04	-0.01	0.09	0.102	2058	0.07	0.02	0.12	0.006	2342	0.09	0.01	0.18	0.033
Phospholipids in small VLDL (mmol/l)	2440	0.04	-0.01	0.09	0.094	2058	0.07	0.02	0.12	0.004	2342	0.09	0.01	0.17	0.034
Total cholesterol in small VLDL (mmol/l)	2440	0.01	-0.03	0.06	0.578	2058	0.04	0.00	0.09	0.078	2342	0.06	-0.03	0.15	0.164
Cholesterol esters in small VLDL (mmol/l)	2440	0.00	-0.05	0.05	0.977	2058	0.03	-0.02	0.08	0.263	2342	0.05	-0.04	0.14	0.297
Free cholesterol in small VLDL (mmol/l)	2440	0.04	-0.01	0.08	0.123	2058	0.07	0.02	0.12	0.006	2342	0.08	0.00	0.17	0.048
Triglycerides in small VLDL (mmol/l)	2440	0.06	0.01	0.11	0.017	2058	0.09	0.03	0.14	0.002	2342	0.11	0.03	0.19	0.010
Concentration of very small VLDL particles (mol/l)	2440	0.00	-0.04	0.05	0.827	2058	0.04	-0.01	0.09	0.107	2342	0.04	-0.05	0.13	0.382
Total lipids in very small VLDL (mmol/l)	2440	-0.01	-0.05	0.04	0.747	2058	0.03	-0.02	0.08	0.241	2342	0.04	-0.05	0.12	0.437
Phospholipids in very small VLDL (mmol/l)	2440	-0.01	-0.05	0.03	0.706	2058	0.02	-0.02	0.07	0.330	2342	0.02	-0.07	0.11	0.633
Total cholesterol in very small VLDL (mmol/l)	2440	-0.03	-0.07	0.02	0.223	2058	0.01	-0.04	0.06	0.802	2342	0.01	-0.08	0.11	0.754
Cholesterol esters in very small VLDL (mmol/l)	2440	-0.03	-0.07	0.02	0.232	2058	0.00	-0.05	0.05	0.885	2342	0.01	-0.08	0.11	0.747
Free cholesterol in very small VLDL (mmol/l)	2440	-0.02	-0.06	0.02	0.231	2058	0.01	-0.04	0.06	0.627	2342	0.01	-0.08	0.10	0.790
Triglycerides in very small VLDL (mmol/l)	2440	0.04	0.00	0.09	0.048	2058	0.07	0.02	0.12	0.005	2342	0.08	0.00	0.16	0.054
Concentration of IDL particles (mol/l)	2440	-0.01	-0.05	0.03	0.593	2058	0.02	-0.03	0.07	0.369	2342	0.01	-0.07	0.10	0.747
Total lipids in IDL (mmol/l)	2440	-0.02	-0.06	0.02	0.403	2058	0.02	-0.03	0.07	0.457	2342	0.01	-0.08	0.10	0.830
Phospholipids in IDL (mmol/l)	2440	-0.02	-0.06	0.02	0.321	2058	0.01	-0.04	0.06	0.651	2342	0.00	-0.09	0.09	0.981

Online Table 9 Interactions between fat mass index and lean mass index in relation to cardiometabolic traits at age 18y in ALSPAC

Model 1 & 3 adjusted for lean mass index, age, age*lean mass index, sex, sex*lean mass index, ethnicity, ethnicity*lean mass index, education, education*lean mass index,
Model 2 additionally adjusted for smoking, smoking*lean mass index, alcohol, alcohol*lean mass index, puberty timing, puberty timing*lean mass index

	Model 1: Interaction of fat mass index at age 10y with lean mass index at age 10y					Model 2: Interaction of fat mass index at age 18y with lean mass index at age 18y					Model 3: Interaction of change in fat mass index (age 10-18y) with change in lean mass index (age 10-18y)				
**Standardized outcome at age 18y**	**N**	**Beta**	**LCI**	**UCI**	**P-value**	**N**	**Beta**	**LCI**	**UCI**	**P-value**	**N**	**Beta**	**LCI**	**UCI**	**P-value**
Total cholesterol in IDL (mmol/l)	2440	-0.02	-0.06	0.02	0.283	2058	0.01	-0.04	0.06	0.615	2342	0.00	-0.08	0.09	0.922
Cholesterol esters in IDL (mmol/l)	2440	-0.02	-0.06	0.02	0.363	2058	0.02	-0.03	0.07	0.485	2342	0.01	-0.08	0.10	0.799
Free cholesterol in IDL (mmol/l)	2440	-0.03	-0.07	0.01	0.148	2058	0.00	-0.05	0.05	0.986	2342	-0.01	-0.10	0.07	0.770
Triglycerides in IDL (mmol/l)	2440	0.02	-0.01	0.06	0.206	2058	0.06	0.01	0.11	0.018	2342	0.05	-0.03	0.13	0.216
Concentration of large LDL particles (mol/l)	2440	-0.01	-0.05	0.03	0.598	2058	0.02	-0.02	0.07	0.355	2342	0.02	-0.07	0.10	0.689
Total lipids in large LDL (mmol/l)	2440	-0.01	-0.05	0.03	0.521	2058	0.02	-0.02	0.07	0.342	2342	0.02	-0.07	0.10	0.709
Phospholipids in large LDL (mmol/l)	2440	-0.01	-0.05	0.03	0.535	2058	0.02	-0.02	0.07	0.350	2342	0.02	-0.07	0.10	0.705
Total cholesterol in large LDL (mmol/l)	2440	-0.02	-0.06	0.02	0.390	2058	0.02	-0.03	0.06	0.490	2342	0.01	-0.08	0.10	0.819
Cholesterol esters in large LDL (mmol/l)	2440	-0.01	-0.05	0.02	0.471	2058	0.02	-0.03	0.07	0.403	2342	0.01	-0.07	0.10	0.738
Free cholesterol in large LDL (mmol/l)	2440	-0.02	-0.06	0.01	0.194	2058	0.01	-0.04	0.05	0.814	2342	0.00	-0.09	0.08	0.915
Triglycerides in large LDL (mmol/l)	2440	0.02	-0.02	0.06	0.252	2058	0.06	0.01	0.11	0.012	2342	0.05	-0.03	0.14	0.189
Concentration of medium LDL particles (mol/l)	2440	-0.01	-0.04	0.03	0.779	2058	0.03	-0.02	0.07	0.268	2342	0.03	-0.06	0.11	0.548
Total lipids in medium LDL (mmol/l)	2440	-0.01	-0.05	0.03	0.637	2058	0.03	-0.02	0.07	0.282	2342	0.02	-0.06	0.11	0.598
Phospholipids in medium LDL (mmol/l)	2440	0.00	-0.04	0.04	0.987	2058	0.04	-0.01	0.09	0.080	2342	0.04	-0.04	0.12	0.354
Total cholesterol in medium LDL (mmol/l)	2440	-0.02	-0.05	0.02	0.430	2058	0.02	-0.03	0.06	0.485	2342	0.01	-0.07	0.10	0.757
Cholesterol esters in medium LDL (mmol/l)	2440	-0.01	-0.05	0.02	0.448	2058	0.02	-0.03	0.06	0.514	2342	0.01	-0.07	0.10	0.761
Free cholesterol in medium LDL (mmol/l)	2440	-0.02	-0.06	0.02	0.375	2058	0.02	-0.03	0.07	0.372	2342	0.01	-0.07	0.10	0.746
Triglycerides in medium LDL (mmol/l)	2440	0.02	-0.02	0.06	0.276	2058	0.05	0.00	0.10	0.043	2342	0.05	-0.03	0.13	0.238
Concentration of small LDL particles (mol/l)	2440	0.00	-0.04	0.03	0.856	2058	0.03	-0.01	0.08	0.156	2342	0.03	-0.05	0.12	0.446
Total lipids in small LDL (mmol/l)	2440	-0.01	-0.05	0.03	0.641	2058	0.03	-0.02	0.07	0.265	2342	0.02	-0.06	0.11	0.575
Phospholipids in small LDL (mmol/l)	2440	0.00	-0.04	0.04	0.933	2058	0.05	0.00	0.10	0.056	2342	0.04	-0.04	0.12	0.333
Total cholesterol in small LDL (mmol/l)	2440	-0.02	-0.06	0.02	0.371	2058	0.01	-0.03	0.06	0.554	2342	0.01	-0.07	0.10	0.792
Cholesterol esters in small LDL (mmol/l)	2440	-0.02	-0.06	0.02	0.392	2058	0.01	-0.04	0.06	0.617	2342	0.01	-0.08	0.10	0.833
Free cholesterol in small LDL (mmol/l)	2440	-0.02	-0.06	0.02	0.318	2058	0.02	-0.03	0.07	0.343	2342	0.02	-0.06	0.10	0.637
Triglycerides in small LDL (mmol/l)	2440	0.03	-0.01	0.07	0.090	2058	0.07	0.02	0.12	0.006	2342	0.08	-0.01	0.16	0.080
Concentration of very large HDL particles (mol/l)	2440	-0.01	-0.05	0.03	0.614	2058	0.01	-0.04	0.06	0.681	2342	-0.04	-0.11	0.04	0.353
Total lipids in very large HDL (mmol/l)	2440	-0.01	-0.05	0.03	0.563	2058	0.01	-0.04	0.06	0.641	2342	-0.03	-0.11	0.04	0.415
Phospholipids in very large HDL (mmol/l)	2440	-0.01	-0.05	0.03	0.594	2058	0.00	-0.05	0.05	0.956	2342	-0.05	-0.12	0.03	0.220
Total cholesterol in very large HDL (mmol/l)	2440	-0.01	-0.06	0.03	0.484	2058	0.02	-0.03	0.07	0.540	2342	-0.02	-0.09	0.06	0.667
Cholesterol esters in very large HDL (mmol/l)	2440	-0.02	-0.06	0.03	0.444	2058	0.02	-0.03	0.07	0.516	2342	-0.01	-0.09	0.07	0.804
Free cholesterol in very large HDL (mmol/l)	2440	-0.01	-0.05	0.03	0.631	2058	0.01	-0.04	0.06	0.599	2342	-0.04	-0.11	0.04	0.368
Triglycerides in very large HDL (mmol/l)	2440	0.03	-0.01	0.07	0.169	2058	0.08	0.03	0.13	0.003	2342	0.06	-0.03	0.15	0.182
Concentration of large HDL particles (mol/l)	2440	-0.01	-0.06	0.03	0.633	2058	0.01	-0.04	0.06	0.802	2342	-0.04	-0.12	0.03	0.265
Total lipids in large HDL (mmol/l)	2440	-0.01	-0.06	0.03	0.645	2058	0.00	-0.05	0.05	0.895	2342	-0.05	-0.13	0.03	0.205
Phospholipids in large HDL (mmol/l)	2440	-0.01	-0.06	0.03	0.650	2058	0.01	-0.04	0.06	0.782	2342	-0.04	-0.12	0.03	0.284
Total cholesterol in large HDL (mmol/l)	2440	-0.01	-0.06	0.03	0.635	2058	0.00	-0.05	0.05	0.957	2342	-0.06	-0.13	0.02	0.152
Cholesterol esters in large HDL (mmol/l)	2440	-0.01	-0.06	0.04	0.671	2058	0.00	-0.05	0.05	0.979	2342	-0.06	-0.13	0.02	0.160
Free cholesterol in large HDL (mmol/l)	2440	-0.01	-0.06	0.03	0.520	2058	0.00	-0.05	0.04	0.886	2342	-0.06	-0.14	0.02	0.129
Triglycerides in large HDL (mmol/l)	2440	0.01	-0.03	0.05	0.731	2058	0.03	-0.02	0.08	0.178	2342	-0.01	-0.09	0.08	0.895
Concentration of medium HDL particles (mol/l)	2440	0.01	-0.03	0.06	0.589	2058	0.04	-0.01	0.10	0.136	2342	0.02	-0.06	0.10	0.598
Total lipids in medium HDL (mmol/l)	2440	0.01	-0.04	0.06	0.702	2058	0.04	-0.02	0.10	0.214	2342	0.01	-0.07	0.08	0.882
Phospholipids in medium HDL (mmol/l)	2440	0.01	-0.03	0.06	0.583	2058	0.04	-0.02	0.10	0.152	2342	0.02	-0.05	0.10	0.567
Total cholesterol in medium HDL (mmol/l)	2440	0.00	-0.05	0.04	0.934	2058	0.02	-0.04	0.08	0.501	2342	-0.02	-0.10	0.05	0.543
Cholesterol esters in medium HDL (mmol/l)	2440	0.00	-0.05	0.04	0.932	2058	0.02	-0.04	0.07	0.547	2342	-0.03	-0.11	0.05	0.507
Free cholesterol in medium HDL (mmol/l)	2440	0.00	-0.04	0.04	0.958	2058	0.03	-0.03	0.08	0.315	2342	-0.01	-0.09	0.06	0.747
Triglycerides in medium HDL (mmol/l)	2440	0.04	-0.01	0.09	0.088	2058	0.06	0.02	0.11	0.010	2342	0.08	-0.01	0.16	0.074
Concentration of small HDL particles (mol/l)	2440	0.03	-0.02	0.07	0.232	2058	0.05	-0.01	0.11	0.086	2342	0.06	-0.02	0.13	0.153

Online Table 9 Interactions between fat mass index and lean mass index in relation to cardiometabolic traits at age 18y in ALSPAC

Model 1 & 3 adjusted for lean mass index, age, age*lean mass index, sex, sex*lean mass index, ethnicity, ethnicity*lean mass index, education, education*lean mass index,
Model 2 additionally adjusted for smoking, smoking*lean mass index, alcohol, alcohol*lean mass index, puberty timing, puberty timing*lean mass index

	Model 1<br>Interaction of fat mass index at age 10y with lean mass index at age 10y					Model 2<br>Interaction of fat mass index at age 18y with lean mass index at age 18y					Model 3<br>Interaction of change in fat mass index (age 10-18y) with change in lean mass index (age 10-18y)				
**Standardized outcome at age 18y**	**N**	**Beta**	**LCI**	**UCI**	**P-value**	**N**	**Beta**	**LCI**	**UCI**	**P-value**	**N**	**Beta**	**LCI**	**UCI**	**P-value**
Total lipids in small HDL (mmol/l)	2440	0.02	-0.03	0.07	0.370	2058	0.04	-0.02	0.09	0.174	2342	0.04	-0.03	0.12	0.248
Phospholipids in small HDL (mmol/l)	2440	0.03	-0.02	0.07	0.275	2058	0.05	-0.01	0.11	0.123	2342	0.05	-0.04	0.13	0.276
Total cholesterol in small HDL (mmol/l)	2440	0.00	-0.04	0.04	0.895	2058	0.01	-0.04	0.05	0.772	2342	0.01	-0.05	0.08	0.687
Cholesterol esters in small HDL (mmol/l)	2440	0.00	-0.04	0.04	0.912	2058	0.00	-0.05	0.04	0.914	2342	0.01	-0.06	0.08	0.773
Free cholesterol in small HDL (mmol/l)	2440	0.02	-0.03	0.07	0.438	2058	0.04	-0.02	0.10	0.227	2342	0.02	-0.06	0.10	0.552
Triglycerides in small HDL (mmol/l)	2440	0.06	0.01	0.11	0.019	2058	0.09	0.04	0.14	0.0003	2342	0.13	0.04	0.21	0.003
Phospholipids to total lipds ratio in chylomicrons and extremely large VLDL (%)	2440	0.00	-0.03	0.02	0.770	2058	0.03	0.00	0.05	0.018	2342	0.01	-0.04	0.06	0.670
Total cholesterol to total lipids ratio in chylomicrons and extremely large VLDL (%)	2440	0.02	-0.03	0.06	0.464	2058	0.03	-0.01	0.08	0.109	2342	0.09	0.00	0.18	0.043
Cholesterol esters to total lipids ratio in chylomicrons and extremely large VLDL (%)	2440	0.01	-0.04	0.05	0.782	2058	0.02	-0.02	0.06	0.417	2342	0.07	-0.03	0.17	0.148
Free cholesterol to total lipids ratio in chylomicrons and extremely large VLDL (%)	2440	0.02	-0.02	0.06	0.266	2058	0.06	0.01	0.10	0.009	2342	0.08	0.00	0.17	0.065
Triglycerides to total lipids ratio in chylomicrons and extremely large VLDL (%)	2440	0.00	-0.03	0.04	0.810	2058	-0.02	-0.06	0.02	0.365	2342	-0.07	-0.14	-0.01	0.025
Phospholipids to total lipds ratio in very large VLDL (%)	2440	0.00	-0.04	0.04	0.909	2058	0.05	0.01	0.09	0.026	2342	0.08	0.00	0.15	0.047
Total cholesterol to total lipids ratio in very large VLDL (%)	2440	-0.02	-0.06	0.01	0.195	2058	0.00	-0.05	0.04	0.844	2342	-0.04	-0.10	0.02	0.194
Cholesterol esters to total lipids ratio in very large VLDL (%)	2440	0.00	-0.04	0.04	0.958	2058	0.00	-0.03	0.03	0.953	2342	-0.02	-0.09	0.05	0.556
Free cholesterol to total lipids ratio in very large VLDL (%)	2440	-0.01	-0.04	0.03	0.615	2058	0.03	-0.01	0.06	0.119	2342	0.02	-0.05	0.09	0.615
Triglycerides to total lipids ratio in very large VLDL (%)	2440	0.02	-0.02	0.05	0.434	2058	-0.03	-0.07	0.02	0.211	2342	0.02	-0.05	0.09	0.641
Phospholipids to total lipds ratio in large VLDL (%)	2440	0.00	-0.03	0.04	0.915	2058	0.01	-0.03	0.05	0.525	2342	0.07	0.00	0.14	0.066
Total cholesterol to total lipids ratio in large VLDL (%)	2440	-0.02	-0.05	0.02	0.372	2058	-0.01	-0.05	0.04	0.765	2342	0.03	-0.04	0.11	0.415
Cholesterol esters to total lipids ratio in large VLDL (%)	2440	-0.04	-0.07	-0.01	0.023	2058	-0.03	-0.07	0.01	0.204	2342	-0.02	-0.08	0.05	0.655
Free cholesterol to total lipids ratio in large VLDL (%)	2440	0.01	-0.01	0.03	0.289	2058	0.01	-0.01	0.03	0.239	2342	0.04	0.00	0.07	0.029
Triglycerides to total lipids ratio in large VLDL (%)	2440	0.01	-0.03	0.05	0.575	2058	-0.01	-0.06	0.04	0.658	2342	-0.05	-0.13	0.02	0.143
Phospholipids to total lipds ratio in medium VLDL (%)	2440	-0.02	-0.06	0.02	0.417	2058	0.03	-0.01	0.08	0.117	2342	0.01	-0.07	0.09	0.848
Total cholesterol to total lipids ratio in medium VLDL (%)	2440	-0.03	-0.07	0.01	0.154	2058	0.01	-0.04	0.05	0.763	2342	0.01	-0.07	0.09	0.827
Cholesterol esters to total lipids ratio in medium VLDL (%)	2440	-0.04	-0.07	0.00	0.053	2058	-0.01	-0.05	0.04	0.793	2342	-0.01	-0.10	0.07	0.754
Free cholesterol to total lipids ratio in medium VLDL (%)	2440	0.02	-0.02	0.06	0.403	2058	0.05	0.00	0.09	0.039	2342	0.07	-0.01	0.15	0.068
Triglycerides to total lipids ratio in medium VLDL (%)	2440	0.03	-0.01	0.07	0.136	2058	-0.01	-0.06	0.03	0.613	2342	-0.01	-0.09	0.07	0.787
Phospholipids to total lipds ratio in small VLDL (%)	2440	-0.01	-0.06	0.04	0.704	2058	-0.01	-0.05	0.04	0.787	2342	-0.05	-0.14	0.04	0.302
Total cholesterol to total lipids ratio in small VLDL (%)	2440	-0.06	-0.10	-0.02	0.002	2058	-0.04	-0.09	0.00	0.076	2342	-0.06	-0.14	0.02	0.153
Cholesterol esters to total lipids ratio in small VLDL (%)	2440	-0.06	-0.10	-0.02	0.003	2058	-0.05	-0.10	0.00	0.046	2342	-0.06	-0.14	0.03	0.182
Free cholesterol to total lipids ratio in small VLDL (%)	2440	-0.02	-0.06	0.03	0.480	2058	0.02	-0.02	0.06	0.381	2342	-0.03	-0.11	0.05	0.452
Triglycerides to total lipids ratio in small VLDL (%)	2440	0.07	0.03	0.11	0.001	2058	0.05	0.00	0.10	0.063	2342	0.08	0.00	0.15	0.049
Phospholipids to total lipds ratio in very small VLDL (%)	2440	0.00	-0.04	0.03	0.801	2058	0.02	-0.03	0.06	0.430	2342	-0.01	-0.09	0.08	0.873
Total cholesterol to total lipids ratio in very small VLDL (%)	2440	-0.05	-0.09	-0.01	0.016	2058	-0.06	-0.11	-0.01	0.029	2342	-0.06	-0.15	0.03	0.175
Cholesterol esters to total lipids ratio in very small VLDL (%)	2440	-0.04	-0.09	0.00	0.043	2058	-0.05	-0.10	0.00	0.036	2342	-0.05	-0.13	0.03	0.248
Free cholesterol to total lipids ratio in very small VLDL (%)	2440	-0.04	-0.08	0.00	0.036	2058	-0.03	-0.09	0.03	0.289	2342	-0.07	-0.17	0.04	0.229
Triglycerides to total lipids ratio in very small VLDL (%)	2440	0.06	0.02	0.11	0.003	2058	0.06	0.00	0.11	0.037	2342	0.08	-0.01	0.17	0.076
Phospholipids to total lipds ratio in IDL (%)	2440	-0.01	-0.06	0.04	0.751	2058	-0.05	-0.10	0.00	0.055	2342	-0.05	-0.15	0.05	0.303
Total cholesterol to total lipids ratio in IDL (%)	2440	-0.04	-0.09	0.00	0.078	2058	-0.02	-0.07	0.03	0.381	2342	-0.04	-0.13	0.05	0.371
Cholesterol esters to total lipids ratio in IDL (%)	2440	-0.02	-0.07	0.03	0.394	2058	0.00	-0.05	0.05	0.899	2342	0.00	-0.09	0.09	0.973
Free cholesterol to total lipids ratio in IDL (%)	2440	-0.05	-0.10	-0.01	0.013	2058	-0.06	-0.11	-0.01	0.020	2342	-0.10	-0.18	-0.03	0.008
Triglycerides to total lipids ratio in IDL (%)	2440	0.06	0.01	0.10	0.020	2058	0.05	0.00	0.10	0.054	2342	0.07	-0.02	0.16	0.110
Phospholipids to total lipds ratio in large LDL (%)	2440	0.01	-0.02	0.05	0.500	2058	-0.03	-0.07	0.02	0.196	2342	-0.01	-0.09	0.07	0.830
Total cholesterol to total lipids ratio in large LDL (%)	2440	-0.04	-0.09	0.00	0.069	2058	-0.01	-0.06	0.04	0.659	2342	-0.04	-0.12	0.05	0.416
Cholesterol esters to total lipids ratio in large LDL (%)	2440	-0.02	-0.06	0.02	0.317	2058	0.02	-0.02	0.07	0.369	2342	0.00	-0.08	0.09	0.938
Free cholesterol to total lipids ratio in large LDL (%)	2440	-0.04	-0.08	0.00	0.071	2058	-0.09	-0.14	-0.04	0.001	2342	-0.10	-0.19	-0.02	0.014
Triglycerides to total lipids ratio in large LDL (%)	2440	0.05	0.00	0.10	0.043	2058	0.05	0.00	0.10	0.047	2342	0.07	-0.02	0.16	0.138
Phospholipids to total lipds ratio in medium LDL (%)	2440	0.03	-0.01	0.06	0.172	2058	-0.01	-0.05	0.04	0.692	2342	0.01	-0.07	0.09	0.810

Online Table 9 Interactions between fat mass index and lean mass index in relation to cardiometabolic traits at age 18y in ALSPAC

Model 1 & 3 adjusted for lean mass index, age, age*lean mass index, sex, sex*lean mass index, ethnicity, ethnicity*lean mass index, education, education*lean mass index,
Model 2 additionally adjusted for smoking, smoking*lean mass index, alcohol, alcohol*lean mass index, puberty timing, puberty timing*lean mass index

	Model 1: Interaction of fat mass index at age 10y with lean mass index at age 10y					Model 2: Interaction of fat mass index at age 18y with lean mass index at age 18y					Model 3: Interaction of change in fat mass index (age 10-18y) with change in lean mass index (age 10-18y)				
**Standardized outcome at age 18y**	**N**	**Beta**	**LCI**	**UCI**	**P-value**	**N**	**Beta**	**LCI**	**UCI**	**P-value**	**N**	**Beta**	**LCI**	**UCI**	**P-value**
Total cholesterol to total lipids ratio in medium LDL (%)	2440	-0.04	-0.08	0.00	0.038	2058	-0.01	-0.06	0.03	0.634	2342	-0.04	-0.12	0.05	0.373
Cholesterol esters to total lipids ratio in medium LDL (%)	2440	-0.03	-0.07	0.01	0.152	2058	0.01	-0.03	0.06	0.658	2342	-0.01	-0.10	0.08	0.814
Free cholesterol to total lipids ratio in medium LDL (%)	2440	0.00	-0.03	0.04	0.871	2058	-0.04	-0.08	0.01	0.094	2342	-0.03	-0.11	0.05	0.429
Triglycerides to total lipids ratio in medium LDL (%)	2440	0.04	0.00	0.09	0.078	2058	0.04	-0.01	0.08	0.085	2342	0.07	-0.02	0.15	0.123
Phospholipids to total lipds ratio in small LDL (%)	2440	0.03	-0.01	0.06	0.165	2058	-0.01	-0.05	0.04	0.724	2342	0.00	-0.08	0.08	0.960
Total cholesterol to total lipids ratio in small LDL (%)	2440	-0.05	-0.09	0.00	0.028	2058	-0.02	-0.06	0.03	0.512	2342	-0.04	-0.12	0.05	0.361
Cholesterol esters to total lipids ratio in small LDL (%)	2440	-0.03	-0.07	0.01	0.127	2058	0.00	-0.04	0.05	0.864	2342	-0.02	-0.10	0.07	0.725
Free cholesterol to total lipids ratio in small LDL (%)	2440	0.00	-0.04	0.04	0.926	2058	-0.04	-0.08	0.01	0.113	2342	-0.03	-0.12	0.06	0.500
Triglycerides to total lipids ratio in small LDL (%)	2440	0.06	0.01	0.11	0.012	2058	0.07	0.02	0.12	0.007	2342	0.11	0.02	0.20	0.015
Phospholipids to total lipds ratio in very large HDL (%)	2440	-0.02	-0.06	0.02	0.294	2058	-0.06	-0.11	-0.01	0.014	2342	-0.10	-0.18	-0.02	0.012
Total cholesterol to total lipids ratio in very large HDL (%)	2440	0.01	-0.03	0.05	0.571	2058	0.04	-0.01	0.09	0.087	2342	0.08	0.00	0.16	0.040
Cholesterol esters to total lipids ratio in very large HDL (%)	2440	0.01	-0.03	0.05	0.604	2058	0.04	-0.01	0.09	0.103	2342	0.09	0.01	0.17	0.034
Free cholesterol to total lipids ratio in very large HDL (%)	2440	0.00	-0.04	0.05	0.847	2058	0.01	-0.05	0.07	0.724	2342	-0.06	-0.14	0.01	0.104
Triglycerides to total lipids ratio in very large HDL (%)	2440	0.06	0.00	0.12	0.057	2058	0.10	0.03	0.17	0.006	2342	0.10	0.02	0.19	0.017
Phospholipids to total lipds ratio in large HDL (%)	2440	0.03	-0.02	0.08	0.227	2058	0.07	0.02	0.12	0.006	2342	0.12	0.04	0.21	0.006
Total cholesterol to total lipids ratio in large HDL (%)	2440	-0.04	-0.09	0.02	0.176	2058	-0.08	-0.14	-0.03	0.004	2342	-0.12	-0.21	-0.04	0.006
Cholesterol esters to total lipids ratio in large HDL (%)	2440	-0.03	-0.09	0.02	0.251	2058	-0.08	-0.14	-0.02	0.010	2342	-0.11	-0.20	-0.03	0.012
Free cholesterol to total lipids ratio in large HDL (%)	2440	-0.05	-0.10	0.00	0.067	2058	-0.08	-0.13	-0.03	0.002	2342	-0.13	-0.22	-0.04	0.004
Triglycerides to total lipids ratio in large HDL (%)	2440	0.04	-0.01	0.10	0.145	2058	0.08	0.02	0.15	0.016	2342	0.09	0.01	0.17	0.027
Phospholipids to total lipds ratio in medium HDL (%)	2440	0.02	-0.02	0.07	0.367	2058	0.05	-0.01	0.10	0.089	2342	0.08	0.00	0.15	0.046
Total cholesterol to total lipids ratio in medium HDL (%)	2440	-0.04	-0.08	0.01	0.120	2058	-0.07	-0.12	-0.02	0.005	2342	-0.10	-0.18	-0.02	0.010
Cholesterol esters to total lipids ratio in medium HDL (%)	2440	-0.03	-0.08	0.01	0.139	2058	-0.07	-0.12	-0.02	0.004	2342	-0.10	-0.17	-0.02	0.012
Free cholesterol to total lipids ratio in medium HDL (%)	2440	-0.03	-0.07	0.01	0.180	2058	-0.03	-0.08	0.02	0.275	2342	-0.06	-0.13	0.01	0.103
Triglycerides to total lipids ratio in medium HDL (%)	2440	0.04	-0.01	0.10	0.113	2058	0.07	0.01	0.13	0.018	2342	0.08	0.00	0.17	0.054
Phospholipids to total lipds ratio in small HDL (%)	2440	0.01	-0.03	0.04	0.749	2058	0.01	-0.03	0.06	0.561	2342	0.00	-0.08	0.07	0.906
Total cholesterol to total lipids ratio in small HDL (%)	2440	-0.02	-0.06	0.02	0.270	2058	-0.04	-0.08	0.01	0.097	2342	-0.03	-0.10	0.04	0.429
Cholesterol esters to total lipids ratio in small HDL (%)	2440	-0.02	-0.05	0.02	0.306	2058	-0.04	-0.08	0.01	0.124	2342	-0.02	-0.09	0.05	0.612
Free cholesterol to total lipids ratio in small HDL (%)	2440	0.00	-0.05	0.04	0.932	2058	0.00	-0.06	0.06	0.988	2342	-0.05	-0.14	0.03	0.216
Triglycerides to total lipids ratio in small HDL (%)	2440	0.05	0.00	0.11	0.041	2058	0.09	0.03	0.15	0.002	2342	0.12	0.03	0.20	0.006
Mean diameter for VLDL particles (nm)	2440	0.05	0.01	0.10	0.024	2058	0.06	0.02	0.11	0.010	2342	0.10	0.03	0.18	0.009
Mean diameter for LDL particles (nm)	2440	-0.02	-0.06	0.02	0.374	2058	-0.06	-0.11	0.00	0.038	2342	-0.07	-0.14	0.00	0.064
Mean diameter for HDL particles (nm)	2440	-0.02	-0.06	0.02	0.321	2058	-0.01	-0.06	0.03	0.590	2342	-0.07	-0.14	0.00	0.066
Serum total cholesterol (mmol/l)	2440	-0.01	-0.05	0.03	0.620	2058	0.03	-0.02	0.08	0.189	2342	0.02	-0.07	0.10	0.698
Total cholesterol in VLDL (mmol/l)	2440	0.03	-0.02	0.08	0.310	2058	0.07	0.02	0.12	0.008	2342	0.08	-0.01	0.17	0.069
Remnant cholesterol (non-HDL, non-LDL -cholesterol) (mmol/l)	2440	0.01	-0.04	0.05	0.811	2058	0.05	0.00	0.10	0.058	2342	0.05	-0.04	0.14	0.256
Total cholesterol in LDL (mmol/l)	2440	-0.02	-0.06	0.02	0.398	2058	0.02	-0.03	0.06	0.499	2342	0.01	-0.07	0.10	0.795
Total cholesterol in HDL (mmol/l)	2440	-0.01	-0.06	0.04	0.705	2058	0.01	-0.04	0.06	0.684	2342	-0.03	-0.11	0.04	0.399
Total cholesterol in HDL2 (mmol/l)	2440	-0.02	-0.06	0.03	0.525	2058	0.00	-0.05	0.05	0.994	2342	-0.04	-0.12	0.03	0.244
Total cholesterol in HDL3 (mmol/l)	2440	0.00	-0.04	0.05	0.891	2058	0.03	-0.02	0.08	0.267	2342	-0.01	-0.08	0.07	0.825
Esterified cholesterol (mmol/l)	2430	-0.01	-0.05	0.03	0.492	2051	0.03	-0.02	0.08	0.281	2332	0.01	-0.07	0.10	0.739
Free cholesterol (mmol/l)	2428	0.00	-0.04	0.04	0.990	2049	0.04	-0.01	0.08	0.126	2330	0.01	-0.07	0.10	0.721
Serum total triglycerides (mmol/l)	2440	0.06	0.01	0.11	0.017	2058	0.10	0.05	0.16	0.0002	2342	0.12	0.03	0.21	0.007
Triglycerides in VLDL (mmol/l)	2440	0.06	0.01	0.12	0.017	2058	0.10	0.05	0.16	0.0003	2342	0.12	0.03	0.21	0.006
Triglycerides in LDL (mmol/l)	2440	0.02	-0.01	0.06	0.212	2058	0.06	0.01	0.11	0.015	2342	0.06	-0.03	0.14	0.172
Triglycerides in HDL (mmol/l)	2440	0.05	0.00	0.10	0.053	2058	0.09	0.03	0.14	0.001	2342	0.09	0.01	0.18	0.035
Diacylglycerol (mmol/l)	2373	0.02	-0.02	0.07	0.312	1997	0.04	-0.02	0.10	0.161	2276	0.05	-0.04	0.14	0.243
Ratio of diacylglycerol to triglycerides	2374	0.00	-0.04	0.04	0.822	1998	-0.01	-0.06	0.05	0.830	2277	0.01	-0.07	0.09	0.794

Online Table 9 Interactions between fat mass index and lean mass index in relation to cardiometabolic traits at age 18y in ALSPAC

Model 1 & 3 adjusted for lean mass index, age, age*lean mass index, sex, sex*lean mass index, ethnicity, ethnicity*lean mass index, education, education*lean mass index,
Model 2 additionally adjusted for smoking, smoking*lean mass index, alcohol, alcohol*lean mass index, puberty timing, puberty timing*lean mass index

	Model 1 Interaction of fat mass index at age 10y with lean mass index at age 10y					Model 2 Interaction of fat mass index at age 18y with lean mass index at age 18y					Model 3 Interaction of change in fat mass index (age 10-18y) with change in lean mass index (age 10-18y)				
Standardized outcome at age 18y	N	Beta	LCI	UCI	P-value	N	Beta	LCI	UCI	P-value	N	Beta	LCI	UCI	P-value
Total phosphoglycerides (mmol/l)	2428	0.01	-0.03	0.05	0.691	2049	0.06	0.01	0.11	0.015	2330	0.04	-0.04	0.11	0.356
Ratio of triglycerides to phosphoglycerides	2428	0.06	0.01	0.11	0.028	2049	0.09	0.03	0.14	0.002	2330	0.10	0.01	0.20	0.036
Phosphatidylcholine and other cholines (mmol/l)	2409	0.02	-0.02	0.06	0.416	2034	0.07	0.01	0.13	0.018	2314	0.04	-0.04	0.11	0.325
Total cholines (mmol/l)	2430	0.00	-0.04	0.04	0.957	2051	0.05	0.00	0.10	0.061	2332	0.04	-0.04	0.12	0.274
Apolipoprotein A-I (g/l)	2440	0.00	-0.04	0.04	0.895	2058	0.04	-0.02	0.09	0.170	2342	0.00	-0.08	0.07	0.899
Apolipoprotein B (g/l)	2440	0.02	-0.03	0.07	0.365	2058	0.06	0.01	0.11	0.019	2342	0.07	-0.02	0.16	0.134
Ratio of apolipoprotein B to apolipoprotein A-I	2440	0.02	-0.03	0.08	0.369	2058	0.06	0.00	0.11	0.043	2342	0.08	-0.01	0.17	0.069
Total fatty acids (mmol/l)	2430	0.03	-0.01	0.07	0.184	2051	0.09	0.04	0.14	0.001	2332	0.07	-0.01	0.16	0.086
Estimated description of fatty acid chain length, not actual carbon number	2430	0.04	0.01	0.08	0.018	2052	0.04	-0.02	0.09	0.174	2333	0.00	-0.08	0.09	0.921
Estimated degree of unsaturation	2430	-0.03	-0.07	0.01	0.202	2051	-0.02	-0.08	0.03	0.377	2332	-0.10	-0.18	-0.01	0.021
22:6, docosahexaenoic acid (mmol/l)	2430	0.00	-0.04	0.04	0.917	2051	0.03	-0.02	0.08	0.251	2332	-0.01	-0.08	0.06	0.754
18:2, linoleic acid (mmol/l)	2429	0.01	-0.02	0.05	0.468	2051	0.06	0.02	0.11	0.009	2332	0.02	-0.06	0.10	0.602
Conjugated linoleic acid (mmol/l)	2429	-0.02	-0.05	0.02	0.406	2050	-0.01	-0.06	0.05	0.839	2331	-0.01	-0.15	0.12	0.822
Omega-3 fatty acids (mmol/l)	2430	0.00	-0.04	0.04	0.887	2051	0.04	-0.01	0.09	0.102	2332	0.00	-0.08	0.08	0.943
Omega-6 fatty acids (mmol/l)	2430	0.01	-0.03	0.05	0.672	2051	0.06	0.01	0.11	0.011	2332	0.03	-0.06	0.11	0.543
Polyunsaturated fatty acids (mmol/l)	2429	0.01	-0.03	0.05	0.698	2051	0.06	0.01	0.11	0.011	2332	0.02	-0.06	0.11	0.589
Monounsaturated fatty acids; 16:1, 18:1 (mmol/l)	2430	0.05	0.00	0.09	0.045	2051	0.08	0.03	0.13	0.002	2332	0.09	0.01	0.17	0.025
Saturated fatty acids (mmol/l)	2429	0.03	-0.02	0.07	0.256	2050	0.10	0.04	0.15	0.001	2331	0.08	0.00	0.16	0.055
Ratio of 22:6 docosahexaenoic acid to total fatty acids (%)	2431	-0.02	-0.06	0.02	0.393	2052	-0.01	-0.05	0.04	0.788	2333	-0.06	-0.13	0.01	0.073
Ratio of 18:2 linoleic acid to total fatty acids (%)	2430	-0.02	-0.06	0.02	0.270	2052	-0.04	-0.09	0.01	0.131	2333	-0.09	-0.17	-0.01	0.021
Ratio of conjugated linoleic acid to total fatty acids (%)	2430	-0.01	-0.05	0.02	0.443	2051	-0.01	-0.06	0.03	0.567	2332	0.00	-0.11	0.11	0.947
Ratio of omega-3 fatty acids to total fatty acids (%)	2431	-0.02	-0.06	0.02	0.338	2052	-0.02	-0.07	0.02	0.336	2333	-0.09	-0.16	-0.01	0.019
Ratio of omega-6 fatty acids to total fatty acids (%)	2431	-0.04	-0.09	0.00	0.044	2052	-0.05	-0.10	-0.01	0.029	2333	-0.11	-0.18	-0.03	0.009
Ratio of polyunsaturated fatty acids to total fatty acids (%)	2430	-0.05	-0.09	0.00	0.034	2052	-0.06	-0.11	-0.01	0.022	2333	-0.12	-0.20	-0.04	0.002
Ratio of monounsaturated fatty acids to total fatty acids (%)	2431	0.05	0.01	0.09	0.021	2052	0.02	-0.03	0.07	0.378	2333	0.09	0.01	0.17	0.032
Ratio of saturated fatty acids to total fatty acids (%)	2430	-0.01	-0.05	0.03	0.591	2051	0.04	-0.01	0.09	0.131	2332	0.03	-0.05	0.11	0.471
Insulin (mu/l)	2478	0.12	-0.03	0.27	0.130	2097	0.14	0.06	0.21	0.001	2378	0.14	0.04	0.23	0.005
Glucose (mmol/l)	2439	0.03	0.00	0.07	0.068	2057	0.03	0.00	0.07	0.055	2341	0.10	0.03	0.17	0.005
Lactate (mmol/l)	2439	0.00	-0.05	0.04	0.872	2057	0.05	0.01	0.09	0.024	2341	0.08	0.00	0.16	0.050
Pyruvate (mmol/l)	2439	-0.02	-0.06	0.02	0.412	2057	0.04	-0.01	0.09	0.120	2341	0.08	0.01	0.16	0.026
Citrate (mmol/l)	2439	0.03	-0.01	0.07	0.151	2057	-0.02	-0.07	0.03	0.462	2341	-0.04	-0.11	0.03	0.288
Alanine (mmol/l)	2439	0.03	-0.01	0.07	0.140	2057	0.08	0.03	0.12	0.001	2341	0.04	-0.03	0.11	0.306
Glutamine (mmol/l)	2439	0.04	0.00	0.08	0.033	2057	0.03	-0.01	0.08	0.155	2341	-0.01	-0.08	0.07	0.840
Histidine (mmol/l)	2439	0.02	-0.02	0.06	0.365	2057	0.03	-0.02	0.08	0.305	2341	0.00	-0.08	0.07	0.978
Isoleucine (mmol/l)	2439	0.05	-0.01	0.10	0.104	2057	0.06	0.01	0.11	0.015	2341	0.05	-0.02	0.12	0.194
Leucine (mmol/l)	2439	0.02	-0.02	0.06	0.380	2057	0.02	-0.03	0.06	0.464	2341	0.03	-0.04	0.10	0.364
Valine (mmol/l)	2439	0.02	-0.02	0.07	0.322	2057	0.01	-0.04	0.06	0.802	2341	0.05	-0.03	0.12	0.220
Phenylalanine (mmol/l)	2438	0.02	-0.04	0.08	0.454	2057	-0.01	-0.05	0.04	0.832	2341	0.02	-0.06	0.09	0.686
Tyrosine (mmol/l)	2439	0.04	0.00	0.09	0.075	2057	0.05	0.00	0.09	0.058	2341	0.08	0.01	0.16	0.030
Acetate (mmol/l)	2438	-0.02	-0.04	0.01	0.225	2057	-0.02	-0.06	0.02	0.293	2340	-0.03	-0.08	0.02	0.249
Acetoacetate (mmol/l)	2439	-0.03	-0.07	0.01	0.101	2057	0.01	-0.04	0.06	0.804	2341	-0.07	-0.14	0.00	0.044
3-hydroxybutyrate (mmol/l)	2436	-0.02	-0.06	0.02	0.330	2054	0.03	-0.03	0.09	0.282	2338	-0.04	-0.11	0.03	0.246
Creatinine (mmol/l)	2439	0.02	-0.02	0.05	0.354	2057	0.01	-0.04	0.05	0.807	2341	-0.03	-0.10	0.03	0.345
Albumin (signal area)	2440	0.01	-0.03	0.05	0.592	2058	0.05	0.00	0.09	0.042	2342	-0.04	-0.12	0.03	0.268
Glycoprotein acetyls, mainly a1-acid glycoprotein (mmol/l)	2439	0.01	-0.04	0.06	0.752	2057	0.06	0.01	0.11	0.012	2341	0.08	0.00	0.16	0.041
C-reactive protein (mg/l)	2519	-0.01	-0.05	0.03	0.610	2124	-0.01	-0.05	0.03	0.561	2419	0.04	-0.02	0.10	0.218

Online Table 9 Interactions between fat mass index and lean mass index in relation to cardiometabolic traits at age 18y in ALSPAC

Model 1 & 3 adjusted for lean mass index, age, age*lean mass index, sex, sex*lean mass index, ethnicity, ethnicity*lean mass index, education, education*lean mass index,
Model 2 additionally adjusted for smoking, smoking*lean mass index, alcohol, alcohol*lean mass index, puberty timing, puberty timing*lean mass index

	**Model 1** Interaction of fat mass index at age 10y with lean mass index at age 10y					**Model 2** Interaction of fat mass index at age 18y with lean mass index at age 18y					**Model 3** Interaction of change in fat mass index (age 10-18y) with change in lean mass index (age 10-18y)				
**Standardized outcome at age 18y**	**N**	**Beta**	**LCI**	**UCI**	**P-value**	**N**	**Beta**	**LCI**	**UCI**	**P-value**	**N**	**Beta**	**LCI**	**UCI**	**P-value**
**Complete case sample**	Interaction of fat mass index at age 10y with lean mass index at age 10y					Interaction of fat mass index at age 18y with lean mass index at age 18y					Interaction of change in fat mass index (age 10-18y) with change in lean mass index (age 10-18y)				
**Standardized outcome at age 15y**	**N**	**Beta**	**LCI**	**UCI**	**P-value**	**N**	**Beta**	**LCI**	**UCI**	**P-value**	**N**	**Beta**	**LCI**	**UCI**	**P-value**
Systolic blood pressure (mmHg)	1722	0.01	-0.04	0.06	0.743	1722	-0.01	-0.07	0.04	0.649	1722	0.00	-0.09	0.08	0.980
Diastolic blood pressure (mmHg)	1722	0.04	-0.01	0.09	0.113	1722	0.07	0.02	0.13	0.013	1722	0.03	-0.07	0.13	0.577
Concentration of chylomicrons and extremely large VLDL particles (mol/l)	1722	0.05	-0.01	0.10	0.081	1722	0.11	0.05	0.18	0.0005	1722	0.10	0.01	0.19	0.031
Total lipids in chylomicrons and extremely large VLDL (mmol/l)	1722	0.05	-0.01	0.10	0.080	1722	0.11	0.05	0.17	0.001	1722	0.10	0.01	0.19	0.031
Phospholipids in chylomicrons and extremely large VLDL (mmol/l)	1722	0.05	0.00	0.11	0.070	1722	0.11	0.05	0.18	0.0005	1722	0.09	0.01	0.18	0.037
Total cholesterol in chylomicrons and extremely large VLDL (mmol/l)	1722	0.05	-0.01	0.11	0.091	1722	0.11	0.05	0.17	0.0004	1722	0.11	0.02	0.19	0.019
Cholesterol esters in chylomicrons and extremely large VLDL (mmol/l)	1722	0.04	-0.01	0.10	0.129	1722	0.11	0.04	0.17	0.001	1722	0.11	0.02	0.20	0.017
Free cholesterol in chylomicrons and extremely large VLDL (mmol/l)	1722	0.05	0.00	0.11	0.063	1722	0.11	0.05	0.18	0.0004	1722	0.10	0.01	0.19	0.031
Triglycerides in chylomicrons and extremely large VLDL (mmol/l)	1722	0.05	-0.01	0.10	0.080	1722	0.11	0.05	0.17	0.001	1722	0.10	0.01	0.18	0.035
Concentration of very large VLDL particles (mol/l)	1722	0.06	0.00	0.11	0.043	1722	0.12	0.05	0.18	0.0003	1722	0.10	0.01	0.18	0.027
Total lipids in very large VLDL (mmol/l)	1722	0.06	0.00	0.11	0.043	1722	0.12	0.05	0.18	0.0003	1722	0.10	0.01	0.18	0.028
Phospholipids in very large VLDL (mmol/l)	1722	0.06	0.00	0.11	0.041	1722	0.12	0.06	0.18	0.0002	1722	0.10	0.01	0.18	0.031
Total cholesterol in very large VLDL (mmol/l)	1722	0.05	-0.01	0.11	0.081	1722	0.11	0.05	0.17	0.0004	1722	0.10	0.01	0.19	0.022
Cholesterol esters in very large VLDL (mmol/l)	1722	0.05	-0.01	0.11	0.096	1722	0.11	0.05	0.17	0.0004	1722	0.10	0.02	0.19	0.020
Free cholesterol in very large VLDL (mmol/l)	1722	0.05	0.00	0.11	0.068	1722	0.12	0.05	0.18	0.0003	1722	0.10	0.01	0.19	0.026
Triglycerides in very large VLDL (mmol/l)	1722	0.06	0.00	0.12	0.036	1722	0.12	0.05	0.18	0.0003	1722	0.10	0.01	0.18	0.030
Concentration of large VLDL particles (mol/l)	1722	0.06	0.00	0.12	0.035	1722	0.11	0.05	0.18	0.0003	1722	0.10	0.02	0.19	0.018
Total lipids in large VLDL (mmol/l)	1722	0.06	0.00	0.12	0.038	1722	0.11	0.05	0.17	0.0003	1722	0.10	0.02	0.19	0.019
Phospholipids in large VLDL (mmol/l)	1722	0.06	0.00	0.12	0.035	1722	0.11	0.05	0.18	0.0003	1722	0.10	0.02	0.19	0.021
Total cholesterol in large VLDL (mmol/l)	1722	0.06	0.00	0.11	0.055	1722	0.11	0.05	0.17	0.0003	1722	0.10	0.02	0.19	0.020
Cholesterol esters in large VLDL (mmol/l)	1722	0.05	-0.01	0.11	0.085	1722	0.10	0.05	0.16	0.001	1722	0.11	0.02	0.19	0.017
Free cholesterol in large VLDL (mmol/l)	1722	0.06	0.00	0.12	0.035	1722	0.12	0.05	0.18	0.0002	1722	0.10	0.01	0.18	0.024
Triglycerides in large VLDL (mmol/l)	1722	0.06	0.00	0.12	0.034	1722	0.11	0.05	0.17	0.0004	1722	0.10	0.02	0.19	0.018
Concentration of medium VLDL particles (mol/l)	1722	0.06	0.00	0.11	0.054	1722	0.10	0.04	0.16	0.001	1722	0.10	0.02	0.19	0.019
Total lipids in medium VLDL (mmol/l)	1722	0.05	0.00	0.11	0.067	1722	0.10	0.04	0.16	0.001	1722	0.10	0.02	0.19	0.020
Phospholipids in medium VLDL (mmol/l)	1722	0.05	0.00	0.11	0.062	1722	0.10	0.04	0.16	0.001	1722	0.10	0.01	0.19	0.022
Total cholesterol in medium VLDL (mmol/l)	1722	0.04	-0.01	0.10	0.145	1722	0.09	0.03	0.15	0.002	1722	0.10	0.01	0.19	0.028
Cholesterol esters in medium VLDL (mmol/l)	1722	0.03	-0.03	0.09	0.303	1722	0.07	0.02	0.13	0.009	1722	0.09	0.00	0.18	0.043
Free cholesterol in medium VLDL (mmol/l)	1722	0.06	0.00	0.11	0.051	1722	0.10	0.04	0.16	0.001	1722	0.10	0.01	0.19	0.022
Triglycerides in medium VLDL (mmol/l)	1722	0.06	0.00	0.11	0.048	1722	0.10	0.04	0.16	0.001	1722	0.10	0.02	0.18	0.019
Concentration of small VLDL particles (mol/l)	1722	0.04	-0.01	0.10	0.120	1722	0.08	0.03	0.14	0.003	1722	0.09	0.00	0.17	0.043
Total lipids in small VLDL (mmol/l)	1722	0.03	-0.02	0.09	0.232	1722	0.07	0.02	0.13	0.009	1722	0.08	0.00	0.17	0.058
Phospholipids in small VLDL (mmol/l)	1722	0.03	-0.02	0.08	0.242	1722	0.07	0.02	0.13	0.006	1722	0.08	0.00	0.17	0.052
Total cholesterol in small VLDL (mmol/l)	1722	0.01	-0.05	0.06	0.852	1722	0.04	-0.02	0.09	0.162	1722	0.06	-0.03	0.16	0.178
Cholesterol esters in small VLDL (mmol/l)	1722	-0.01	-0.06	0.05	0.781	1722	0.02	-0.03	0.07	0.486	1722	0.05	-0.04	0.15	0.284
Free cholesterol in small VLDL (mmol/l)	1722	0.03	-0.02	0.08	0.257	1722	0.07	0.02	0.12	0.011	1722	0.08	-0.01	0.16	0.072
Triglycerides in small VLDL (mmol/l)	1722	0.05	0.00	0.11	0.048	1722	0.09	0.04	0.15	0.002	1722	0.09	0.01	0.17	0.031
Concentration of very small VLDL particles (mol/l)	1722	0.00	-0.05	0.05	0.928	1722	0.03	-0.02	0.08	0.250	1722	0.04	-0.05	0.13	0.367
Total lipids in very small VLDL (mmol/l)	1722	-0.01	-0.06	0.04	0.677	1722	0.02	-0.04	0.07	0.537	1722	0.04	-0.06	0.14	0.413
Phospholipids in very small VLDL (mmol/l)	1722	-0.01	-0.06	0.04	0.661	1722	0.01	-0.04	0.06	0.705	1722	0.02	-0.07	0.12	0.616
Total cholesterol in very small VLDL (mmol/l)	1722	-0.03	-0.08	0.02	0.249	1722	-0.01	-0.06	0.04	0.721	1722	0.03	-0.07	0.13	0.566

Online Table 9 Interactions between fat mass index and lean mass index in relation to cardiometabolic traits at age 18y in ALSPAC

Model 1 & 3 adjusted for lean mass index, age, age*lean mass index, sex, sex*lean mass index, ethnicity, ethnicity*lean mass index, education, education*lean mass index,
Model 2 additionally adjusted for smoking, smoking*lean mass index, alcohol, alcohol*lean mass index, puberty timing, puberty timing*lean mass index

	Model 1 Interaction of fat mass index at age 10y with lean mass index at age 10y					Model 2 Interaction of fat mass index at age 18y with lean mass index at age 18y					Model 3 Interaction of change in fat mass index (age 10-18y) with change in lean mass index (age 10-18y)				
**Standardized outcome at age 18y**	**N**	**Beta**	**LCI**	**UCI**	**P-value**	**N**	**Beta**	**LCI**	**UCI**	**P-value**	**N**	**Beta**	**LCI**	**UCI**	**P-value**
Cholesterol esters in very small VLDL (mmol/l)	1722	-0.03	-0.09	0.02	0.246	1722	-0.01	-0.07	0.04	0.677	1722	0.03	-0.07	0.13	0.587
Free cholesterol in very small VLDL (mmol/l)	1722	-0.03	-0.07	0.02	0.302	1722	0.00	-0.06	0.05	0.877	1722	0.03	-0.07	0.13	0.541
Triglycerides in very small VLDL (mmol/l)	1722	0.04	-0.01	0.09	0.099	1722	0.08	0.02	0.13	0.006	1722	0.06	-0.02	0.15	0.134
Concentration of IDL particles (mol/l)	1722	-0.01	-0.05	0.03	0.650	1722	0.01	-0.05	0.06	0.836	1722	0.01	-0.08	0.11	0.789
Total lipids in IDL (mmol/l)	1722	-0.02	-0.06	0.03	0.440	1722	0.00	-0.05	0.05	0.958	1722	0.01	-0.09	0.11	0.802
Phospholipids in IDL (mmol/l)	1722	-0.02	-0.06	0.02	0.364	1722	-0.01	-0.06	0.05	0.821	1722	0.00	-0.10	0.10	0.971
Total cholesterol in IDL (mmol/l)	1722	-0.02	-0.07	0.02	0.319	1722	-0.01	-0.06	0.05	0.824	1722	0.01	-0.09	0.11	0.819
Cholesterol esters in IDL (mmol/l)	1722	-0.02	-0.07	0.03	0.375	1722	0.00	-0.05	0.05	0.988	1722	0.02	-0.08	0.12	0.694
Free cholesterol in IDL (mmol/l)	1722	-0.03	-0.07	0.02	0.217	1722	-0.02	-0.07	0.03	0.484	1722	-0.01	-0.11	0.09	0.858
Triglycerides in IDL (mmol/l)	1722	0.03	-0.01	0.07	0.200	1722	0.06	0.00	0.11	0.034	1722	0.04	-0.05	0.12	0.423
Concentration of large LDL particles (mol/l)	1722	-0.01	-0.06	0.03	0.534	1722	0.01	-0.04	0.06	0.785	1722	0.01	-0.08	0.11	0.784
Total lipids in large LDL (mmol/l)	1722	-0.02	-0.06	0.03	0.479	1722	0.01	-0.04	0.06	0.762	1722	0.01	-0.08	0.11	0.767
Phospholipids in large LDL (mmol/l)	1722	-0.02	-0.06	0.03	0.451	1722	0.01	-0.04	0.06	0.765	1722	0.02	-0.08	0.11	0.736
Total cholesterol in large LDL (mmol/l)	1722	-0.02	-0.07	0.02	0.362	1722	0.00	-0.05	0.05	0.983	1722	0.01	-0.09	0.11	0.844
Cholesterol esters in large LDL (mmol/l)	1722	-0.02	-0.06	0.03	0.418	1722	0.00	-0.05	0.06	0.866	1722	0.01	-0.08	0.11	0.781
Free cholesterol in large LDL (mmol/l)	1722	-0.03	-0.07	0.02	0.219	1722	-0.01	-0.06	0.04	0.668	1722	0.00	-0.10	0.09	0.955
Triglycerides in large LDL (mmol/l)	1722	0.03	-0.02	0.07	0.228	1722	0.06	0.01	0.11	0.031	1722	0.04	-0.05	0.13	0.381
Concentration of medium LDL particles (mol/l)	1722	-0.01	-0.05	0.03	0.625	1722	0.01	-0.04	0.06	0.626	1722	0.02	-0.08	0.12	0.721
Total lipids in medium LDL (mmol/l)	1722	-0.01	-0.06	0.03	0.533	1722	0.01	-0.04	0.06	0.658	1722	0.02	-0.08	0.11	0.724
Phospholipids in medium LDL (mmol/l)	1722	-0.01	-0.05	0.04	0.744	1722	0.03	-0.02	0.08	0.239	1722	0.04	-0.06	0.13	0.447
Total cholesterol in medium LDL (mmol/l)	1722	-0.02	-0.07	0.02	0.374	1722	0.00	-0.05	0.05	0.979	1722	0.01	-0.09	0.11	0.853
Cholesterol esters in medium LDL (mmol/l)	1722	-0.02	-0.07	0.03	0.393	1722	0.00	-0.05	0.05	0.985	1722	0.01	-0.09	0.11	0.868
Free cholesterol in medium LDL (mmol/l)	1722	-0.02	-0.07	0.02	0.318	1722	0.01	-0.05	0.06	0.815	1722	0.01	-0.08	0.11	0.790
Triglycerides in medium LDL (mmol/l)	1722	0.02	-0.02	0.07	0.302	1722	0.05	-0.01	0.10	0.084	1722	0.03	-0.06	0.12	0.497
Concentration of small LDL particles (mol/l)	1722	-0.01	-0.05	0.04	0.687	1722	0.02	-0.03	0.07	0.450	1722	0.02	-0.08	0.12	0.671
Total lipids in small LDL (mmol/l)	1722	-0.01	-0.06	0.03	0.528	1722	0.01	-0.04	0.06	0.646	1722	0.02	-0.08	0.11	0.737
Phospholipids in small LDL (mmol/l)	1722	0.00	-0.05	0.04	0.859	1722	0.03	-0.02	0.09	0.215	1722	0.03	-0.06	0.13	0.482
Total cholesterol in small LDL (mmol/l)	1722	-0.02	-0.07	0.02	0.324	1722	0.00	-0.05	0.05	0.944	1722	0.01	-0.09	0.10	0.903
Cholesterol esters in small LDL (mmol/l)	1722	-0.02	-0.07	0.02	0.343	1722	0.00	-0.06	0.05	0.881	1722	0.00	-0.10	0.10	0.964
Free cholesterol in small LDL (mmol/l)	1722	-0.03	-0.07	0.02	0.278	1722	0.01	-0.05	0.06	0.780	1722	0.02	-0.07	0.12	0.655
Triglycerides in small LDL (mmol/l)	1722	0.03	-0.01	0.08	0.139	1722	0.07	0.01	0.12	0.013	1722	0.05	-0.04	0.14	0.246
Concentration of very large HDL particles (mol/l)	1722	0.00	-0.04	0.05	0.848	1722	0.00	-0.05	0.05	0.998	1722	-0.04	-0.13	0.05	0.396
Total lipids in very large HDL (mmol/l)	1722	0.00	-0.05	0.05	0.916	1722	0.00	-0.06	0.06	0.999	1722	-0.04	-0.13	0.05	0.417
Phospholipids in very large HDL (mmol/l)	1722	0.00	-0.04	0.05	0.882	1722	-0.01	-0.06	0.05	0.805	1722	-0.05	-0.14	0.04	0.280
Total cholesterol in very large HDL (mmol/l)	1722	0.00	-0.05	0.05	0.933	1722	0.00	-0.06	0.06	0.954	1722	-0.03	-0.11	0.06	0.562
Cholesterol esters in very large HDL (mmol/l)	1722	0.00	-0.05	0.05	0.859	1722	0.00	-0.06	0.06	0.948	1722	-0.02	-0.11	0.07	0.672
Free cholesterol in very large HDL (mmol/l)	1722	0.00	-0.04	0.05	0.843	1722	0.00	-0.05	0.06	0.920	1722	-0.04	-0.13	0.05	0.347
Triglycerides in very large HDL (mmol/l)	1722	0.05	0.00	0.10	0.058	1722	0.07	0.01	0.12	0.018	1722	0.06	-0.04	0.15	0.223
Concentration of large HDL particles (mol/l)	1722	0.00	-0.05	0.06	0.951	1722	0.00	-0.06	0.06	0.966	1722	-0.04	-0.13	0.05	0.399
Total lipids in large HDL (mmol/l)	1722	0.00	-0.05	0.06	0.942	1722	0.00	-0.06	0.05	0.926	1722	-0.05	-0.13	0.04	0.303
Phospholipids in large HDL (mmol/l)	1722	0.00	-0.05	0.05	0.998	1722	0.00	-0.06	0.06	0.989	1722	-0.04	-0.13	0.05	0.380
Total cholesterol in large HDL (mmol/l)	1722	0.00	-0.05	0.06	0.912	1722	-0.01	-0.06	0.05	0.821	1722	-0.05	-0.14	0.04	0.239
Cholesterol esters in large HDL (mmol/l)	1722	0.00	-0.05	0.06	0.873	1722	-0.01	-0.06	0.05	0.853	1722	-0.05	-0.14	0.04	0.253
Free cholesterol in large HDL (mmol/l)	1722	0.00	-0.05	0.05	0.957	1722	-0.01	-0.06	0.04	0.715	1722	-0.06	-0.15	0.03	0.197
Triglycerides in large HDL (mmol/l)	1722	0.02	-0.02	0.06	0.371	1722	0.02	-0.03	0.07	0.449	1722	0.00	-0.09	0.09	0.921
Concentration of medium HDL particles (mol/l)	1722	0.01	-0.05	0.07	0.732	1722	0.05	-0.02	0.11	0.180	1722	0.03	-0.05	0.12	0.455
Total lipids in medium HDL (mmol/l)	1722	0.01	-0.05	0.06	0.813	1722	0.04	-0.03	0.10	0.272	1722	0.02	-0.07	0.10	0.720

Online Table 9 Interactions between fat mass index and lean mass index in relation to cardiometabolic traits at age 18y in ALSPAC

Model 1 & 3 adjusted for lean mass index, age, age*lean mass index, sex, sex*lean mass index, ethnicity, ethnicity*lean mass index, education, education*lean mass index,
Model 2 additionally adjusted for smoking, smoking*lean mass index, alcohol, alcohol*lean mass index, puberty timing, puberty timing*lean mass index

	Model 1 Interaction of fat mass index at age 10y with lean mass index at age 10y					Model 2 Interaction of fat mass index at age 18y with lean mass index at age 18y					Model 3 Interaction of change in fat mass index (age 10-18y) with change in lean mass index (age 10-18y)				
**Standardized outcome at age 18y**	**N**	**Beta**	**LCI**	**UCI**	**P-value**	**N**	**Beta**	**LCI**	**UCI**	**P-value**	**N**	**Beta**	**LCI**	**UCI**	**P-value**
Phospholipids in medium HDL (mmol/l)	1722	0.01	-0.05	0.07	0.703	1722	0.04	-0.02	0.11	0.194	1722	0.04	-0.05	0.12	0.426
Total cholesterol in medium HDL (mmol/l)	1722	0.00	-0.06	0.05	0.919	1722	0.02	-0.05	0.08	0.597	1722	-0.02	-0.11	0.07	0.677
Cholesterol esters in medium HDL (mmol/l)	1722	0.00	-0.06	0.05	0.934	1722	0.02	-0.05	0.08	0.642	1722	-0.02	-0.11	0.07	0.642
Free cholesterol in medium HDL (mmol/l)	1722	0.00	-0.06	0.05	0.868	1722	0.03	-0.04	0.09	0.401	1722	-0.01	-0.10	0.08	0.862
Triglycerides in medium HDL (mmol/l)	1722	0.03	-0.02	0.08	0.235	1722	0.07	0.02	0.12	0.008	1722	0.09	0.01	0.17	0.033
Concentration of small HDL particles (mol/l)	1722	0.02	-0.04	0.07	0.579	1722	0.06	0.00	0.12	0.062	1722	0.06	-0.03	0.14	0.200
Total lipids in small HDL (mmol/l)	1722	0.01	-0.05	0.07	0.728	1722	0.05	-0.02	0.11	0.145	1722	0.05	-0.04	0.13	0.264
Phospholipids in small HDL (mmol/l)	1722	0.02	-0.04	0.07	0.571	1722	0.06	-0.01	0.12	0.087	1722	0.05	-0.05	0.14	0.337
Total cholesterol in small HDL (mmol/l)	1722	-0.01	-0.06	0.04	0.780	1722	0.01	-0.04	0.06	0.742	1722	0.03	-0.05	0.10	0.532
Cholesterol esters in small HDL (mmol/l)	1722	-0.01	-0.06	0.03	0.574	1722	0.00	-0.05	0.05	0.942	1722	0.02	-0.06	0.10	0.580
Free cholesterol in small HDL (mmol/l)	1722	0.02	-0.05	0.08	0.612	1722	0.05	-0.03	0.12	0.212	1722	0.03	-0.06	0.12	0.528
Triglycerides in small HDL (mmol/l)	1722	0.05	0.00	0.11	0.042	1722	0.10	0.05	0.16	0.0002	1722	0.12	0.04	0.20	0.005
Phospholipids to total lipds ratio in chylomicrons and extremely large VLDL (%)	1722	0.00	-0.02	0.03	0.886	1722	0.03	0.00	0.06	0.034	1722	0.00	-0.06	0.06	0.944
Total cholesterol to total lipids ratio in chylomicrons and extremely large VLDL (%)	1722	0.00	-0.04	0.05	0.848	1722	0.02	-0.02	0.07	0.345	1722	0.13	0.03	0.24	0.010
Cholesterol esters to total lipids ratio in chylomicrons and extremely large VLDL (%)	1722	-0.01	-0.06	0.04	0.705	1722	0.00	-0.04	0.05	0.877	1722	0.12	0.00	0.23	0.046
Free cholesterol to total lipids ratio in chylomicrons and extremely large VLDL (%)	1722	0.03	-0.02	0.08	0.186	1722	0.06	0.01	0.10	0.018	1722	0.08	-0.02	0.18	0.121
Triglycerides to total lipids ratio in chylomicrons and extremely large VLDL (%)	1722	0.02	-0.03	0.07	0.432	1722	0.00	-0.05	0.04	0.856	1722	-0.10	-0.18	-0.03	0.006
Phospholipids to total lipds ratio in very large VLDL (%)	1722	0.01	-0.04	0.05	0.711	1722	0.04	0.00	0.09	0.063	1722	0.08	-0.01	0.16	0.088
Total cholesterol to total lipids ratio in very large VLDL (%)	1722	-0.02	-0.07	0.02	0.251	1722	-0.02	-0.07	0.03	0.418	1722	-0.07	-0.13	0.00	0.046
Cholesterol esters to total lipids ratio in very large VLDL (%)	1722	0.00	-0.04	0.03	0.783	1722	-0.01	-0.05	0.02	0.447	1722	-0.05	-0.11	0.01	0.079
Free cholesterol to total lipids ratio in very large VLDL (%)	1722	-0.02	-0.04	0.01	0.213	1722	0.01	-0.02	0.05	0.451	1722	-0.04	-0.09	0.02	0.169
Triglycerides to total lipids ratio in very large VLDL (%)	1722	0.01	-0.04	0.05	0.761	1722	-0.01	-0.06	0.03	0.608	1722	0.04	-0.03	0.11	0.274
Phospholipids to total lipds ratio in large VLDL (%)	1722	0.00	-0.04	0.05	0.817	1722	0.02	-0.03	0.06	0.503	1722	0.08	0.00	0.16	0.047
Total cholesterol to total lipids ratio in large VLDL (%)	1722	-0.02	-0.06	0.03	0.483	1722	-0.02	-0.07	0.03	0.493	1722	0.03	-0.06	0.11	0.511
Cholesterol esters to total lipids ratio in large VLDL (%)	1722	-0.03	-0.07	0.00	0.047	1722	-0.04	-0.09	0.01	0.083	1722	-0.03	-0.11	0.05	0.470
Free cholesterol to total lipids ratio in large VLDL (%)	1722	0.01	-0.01	0.03	0.408	1722	0.01	-0.01	0.03	0.288	1722	0.04	0.00	0.08	0.036
Triglycerides to total lipids ratio in large VLDL (%)	1722	0.01	-0.04	0.05	0.796	1722	-0.01	-0.06	0.05	0.797	1722	-0.07	-0.15	0.02	0.117
Phospholipids to total lipds ratio in medium VLDL (%)	1722	0.00	-0.05	0.05	0.997	1722	0.03	-0.02	0.07	0.287	1722	-0.02	-0.11	0.06	0.608
Total cholesterol to total lipids ratio in medium VLDL (%)	1722	-0.02	-0.06	0.02	0.375	1722	-0.01	-0.06	0.04	0.801	1722	0.04	-0.05	0.12	0.367
Cholesterol esters to total lipids ratio in medium VLDL (%)	1722	-0.03	-0.07	0.01	0.137	1722	-0.02	-0.07	0.03	0.438	1722	0.02	-0.07	0.11	0.643
Free cholesterol to total lipids ratio in medium VLDL (%)	1722	0.03	-0.02	0.08	0.199	1722	0.04	-0.01	0.09	0.110	1722	0.08	-0.01	0.16	0.070
Triglycerides to total lipids ratio in medium VLDL (%)	1722	0.02	-0.02	0.06	0.400	1722	0.00	-0.05	0.05	0.916	1722	-0.03	-0.12	0.06	0.484
Phospholipids to total lipds ratio in small VLDL (%)	1722	-0.01	-0.07	0.05	0.722	1722	0.00	-0.05	0.05	0.966	1722	-0.04	-0.15	0.06	0.421
Total cholesterol to total lipids ratio in small VLDL (%)	1722	-0.07	-0.11	-0.02	0.005	1722	-0.06	-0.12	-0.01	0.019	1722	-0.04	-0.13	0.05	0.339
Cholesterol esters to total lipids ratio in small VLDL (%)	1722	-0.07	-0.11	-0.02	0.007	1722	-0.07	-0.12	-0.02	0.012	1722	-0.04	-0.14	0.05	0.359
Free cholesterol to total lipids ratio in small VLDL (%)	1722	-0.01	-0.06	0.05	0.825	1722	0.01	-0.04	0.06	0.624	1722	-0.02	-0.11	0.07	0.658
Triglycerides to total lipids ratio in small VLDL (%)	1722	0.07	0.03	0.11	0.001	1722	0.06	0.01	0.12	0.019	1722	0.06	-0.02	0.14	0.166
Phospholipids to total lipds ratio in very small VLDL (%)	1722	-0.01	-0.04	0.03	0.789	1722	0.01	-0.04	0.06	0.742	1722	-0.01	-0.11	0.08	0.764
Total cholesterol to total lipids ratio in very small VLDL (%)	1722	-0.05	-0.10	-0.01	0.029	1722	-0.07	-0.12	-0.01	0.015	1722	-0.04	-0.13	0.06	0.452
Cholesterol esters to total lipids ratio in very small VLDL (%)	1722	-0.05	-0.10	0.00	0.067	1722	-0.06	-0.12	-0.01	0.028	1722	-0.03	-0.12	0.06	0.488
Free cholesterol to total lipids ratio in very small VLDL (%)	1722	-0.04	-0.08	0.00	0.077	1722	-0.04	-0.10	0.02	0.171	1722	-0.02	-0.14	0.09	0.674
Triglycerides to total lipids ratio in very small VLDL (%)	1722	0.07	0.02	0.12	0.007	1722	0.08	0.02	0.13	0.007	1722	0.05	-0.04	0.15	0.255
Phospholipids to total lipds ratio in IDL (%)	1722	-0.01	-0.07	0.05	0.757	1722	-0.05	-0.10	0.01	0.100	1722	-0.07	-0.18	0.03	0.175
Total cholesterol to total lipids ratio in IDL (%)	1722	-0.05	-0.11	0.01	0.088	1722	-0.04	-0.09	0.01	0.116	1722	-0.02	-0.12	0.08	0.722
Cholesterol esters to total lipids ratio in IDL (%)	1722	-0.03	-0.09	0.03	0.317	1722	-0.01	-0.06	0.05	0.775	1722	0.03	-0.08	0.13	0.607
Free cholesterol to total lipids ratio in IDL (%)	1722	-0.05	-0.10	-0.01	0.021	1722	-0.08	-0.14	-0.02	0.005	1722	-0.11	-0.19	-0.02	0.013
Triglycerides to total lipids ratio in IDL (%)	1722	0.07	0.01	0.12	0.023	1722	0.07	0.02	0.12	0.008	1722	0.05	-0.04	0.15	0.287

Online Table 9 Interactions between fat mass index and lean mass index in relation to cardiometabolic traits at age 18y in ALSPAC

Model 1 & 3 adjusted for lean mass index, age, age*lean mass index, sex, sex*lean mass index, ethnicity, ethnicity*lean mass index, education, education*lean mass index,
Model 2 additionally adjusted for smoking, smoking*lean mass index, alcohol, alcohol*lean mass index, puberty timing, puberty timing*lean mass index

	**Model 1** Interaction of fat mass index at age 10y with lean mass index at age 10y					**Model 2** Interaction of fat mass index at age 18y with lean mass index at age 18y					**Model 3** Interaction of change in fat mass index (age 10-18y) with change in lean mass index (age 10-18y)				
**Standardized outcome at age 18y**	**N**	**Beta**	**LCI**	**UCI**	**P-value**	**N**	**Beta**	**LCI**	**UCI**	**P-value**	**N**	**Beta**	**LCI**	**UCI**	**P-value**
Phospholipids to total lipds ratio in large LDL (%)	1722	0.02	-0.03	0.06	0.448	1722	-0.01	-0.06	0.04	0.629	1722	0.01	-0.09	0.10	0.913
Total cholesterol to total lipids ratio in large LDL (%)	1722	-0.05	-0.11	0.00	0.049	1722	-0.03	-0.08	0.01	0.167	1722	-0.04	-0.14	0.06	0.461
Cholesterol esters to total lipids ratio in large LDL (%)	1722	-0.03	-0.09	0.02	0.182	1722	0.00	-0.05	0.05	0.975	1722	0.00	-0.11	0.10	0.931
Free cholesterol to total lipids ratio in large LDL (%)	1722	-0.03	-0.08	0.01	0.153	1722	-0.09	-0.14	-0.03	0.002	1722	-0.09	-0.17	0.00	0.052
Triglycerides to total lipids ratio in large LDL (%)	1722	0.07	0.01	0.13	0.035	1722	0.07	0.02	0.12	0.008	1722	0.06	-0.05	0.16	0.288
Phospholipids to total lipds ratio in medium LDL (%)	1722	0.03	-0.01	0.07	0.134	1722	0.01	-0.04	0.06	0.614	1722	0.03	-0.07	0.12	0.576
Total cholesterol to total lipids ratio in medium LDL (%)	1722	-0.05	-0.10	0.00	0.032	1722	-0.04	-0.09	0.01	0.138	1722	-0.05	-0.15	0.05	0.349
Cholesterol esters to total lipids ratio in medium LDL (%)	1722	-0.04	-0.08	0.01	0.099	1722	-0.01	-0.06	0.04	0.631	1722	-0.02	-0.13	0.08	0.638
Free cholesterol to total lipids ratio in medium LDL (%)	1722	0.01	-0.03	0.05	0.604	1722	-0.02	-0.07	0.02	0.303	1722	-0.01	-0.11	0.08	0.762
Triglycerides to total lipids ratio in medium LDL (%)	1722	0.05	-0.01	0.11	0.091	1722	0.05	0.01	0.10	0.029	1722	0.05	-0.04	0.15	0.299
Phospholipids to total lipds ratio in small LDL (%)	1722	0.04	-0.01	0.08	0.102	1722	0.01	-0.04	0.06	0.624	1722	0.02	-0.08	0.11	0.719
Total cholesterol to total lipids ratio in small LDL (%)	1722	-0.06	-0.11	-0.01	0.022	1722	-0.04	-0.09	0.01	0.125	1722	-0.05	-0.15	0.05	0.350
Cholesterol esters to total lipids ratio in small LDL (%)	1722	-0.04	-0.09	0.00	0.077	1722	-0.02	-0.07	0.03	0.480	1722	-0.03	-0.13	0.07	0.541
Free cholesterol to total lipids ratio in small LDL (%)	1722	0.01	-0.04	0.05	0.749	1722	-0.02	-0.07	0.02	0.323	1722	0.00	-0.10	0.09	0.933
Triglycerides to total lipids ratio in small LDL (%)	1722	0.07	0.01	0.13	0.020	1722	0.09	0.03	0.14	0.002	1722	0.10	0.00	0.19	0.051
Phospholipids to total lipds ratio in very large HDL (%)	1722	-0.01	-0.06	0.03	0.578	1722	-0.06	-0.11	0.00	0.037	1722	-0.09	-0.18	0.00	0.038
Total cholesterol to total lipids ratio in very large HDL (%)	1722	0.00	-0.04	0.05	0.950	1722	0.04	-0.01	0.09	0.154	1722	0.08	-0.01	0.16	0.087
Cholesterol esters to total lipids ratio in very large HDL (%)	1722	0.00	-0.04	0.04	0.998	1722	0.04	-0.02	0.09	0.182	1722	0.08	-0.01	0.17	0.070
Free cholesterol to total lipids ratio in very large HDL (%)	1722	0.01	-0.04	0.07	0.604	1722	0.02	-0.05	0.09	0.557	1722	-0.08	-0.17	0.01	0.082
Triglycerides to total lipids ratio in very large HDL (%)	1722	0.06	-0.01	0.13	0.087	1722	0.10	0.02	0.18	0.011	1722	0.09	0.00	0.18	0.039
Phospholipids to total lipds ratio in large HDL (%)	1722	0.01	-0.05	0.07	0.715	1722	0.06	0.01	0.12	0.023	1722	0.10	0.01	0.19	0.039
Total cholesterol to total lipids ratio in large HDL (%)	1722	-0.02	-0.08	0.04	0.493	1722	-0.08	-0.14	-0.02	0.013	1722	-0.10	-0.19	-0.01	0.031
Cholesterol esters to total lipids ratio in large HDL (%)	1722	-0.01	-0.08	0.05	0.647	1722	-0.07	-0.14	0.00	0.035	1722	-0.09	-0.18	0.00	0.063
Free cholesterol to total lipids ratio in large HDL (%)	1722	-0.04	-0.09	0.02	0.178	1722	-0.09	-0.14	-0.03	0.001	1722	-0.12	-0.21	-0.03	0.012
Triglycerides to total lipids ratio in large HDL (%)	1722	0.04	-0.03	0.11	0.243	1722	0.08	0.01	0.15	0.029	1722	0.08	-0.01	0.17	0.072
Phospholipids to total lipds ratio in medium HDL (%)	1722	0.02	-0.04	0.08	0.464	1722	0.05	-0.01	0.12	0.089	1722	0.09	0.01	0.18	0.034
Total cholesterol to total lipids ratio in medium HDL (%)	1722	-0.03	-0.09	0.02	0.243	1722	-0.08	-0.14	-0.02	0.005	1722	-0.12	-0.20	-0.04	0.004
Cholesterol esters to total lipids ratio in medium HDL (%)	1722	-0.03	-0.08	0.02	0.287	1722	-0.08	-0.14	-0.03	0.004	1722	-0.11	-0.19	-0.03	0.005
Free cholesterol to total lipids ratio in medium HDL (%)	1722	-0.03	-0.08	0.02	0.217	1722	-0.03	-0.08	0.02	0.226	1722	-0.08	-0.16	0.01	0.078
Triglycerides to total lipids ratio in medium HDL (%)	1722	0.03	-0.03	0.09	0.272	1722	0.08	0.01	0.14	0.017	1722	0.09	0.00	0.18	0.040
Phospholipids to total lipds ratio in small HDL (%)	1722	0.01	-0.03	0.05	0.585	1722	0.02	-0.03	0.07	0.425	1722	-0.01	-0.10	0.07	0.756
Total cholesterol to total lipids ratio in small HDL (%)	1722	-0.03	-0.07	0.02	0.216	1722	-0.05	-0.10	0.00	0.068	1722	-0.02	-0.10	0.06	0.682
Cholesterol esters to total lipids ratio in small HDL (%)	1722	-0.03	-0.07	0.01	0.192	1722	-0.04	-0.09	0.01	0.091	1722	-0.01	-0.09	0.07	0.855
Free cholesterol to total lipids ratio in small HDL (%)	1722	0.02	-0.04	0.07	0.521	1722	0.00	-0.07	0.06	0.969	1722	-0.05	-0.14	0.04	0.288
Triglycerides to total lipids ratio in small HDL (%)	1722	0.06	0.00	0.11	0.060	1722	0.10	0.03	0.17	0.003	1722	0.11	0.02	0.19	0.014
Mean diameter for VLDL particles (nm)	1722	0.05	0.00	0.10	0.048	1722	0.07	0.02	0.12	0.010	1722	0.10	0.01	0.18	0.021
Mean diameter for LDL particles (nm)	1722	0.00	-0.05	0.05	0.976	1722	-0.05	-0.11	0.01	0.101	1722	-0.04	-0.11	0.04	0.371
Mean diameter for HDL particles (nm)	1722	0.00	-0.05	0.04	0.846	1722	-0.02	-0.08	0.03	0.370	1722	-0.07	-0.15	0.02	0.142
Serum total cholesterol (mmol/l)	1722	-0.01	-0.06	0.03	0.605	1722	0.02	-0.04	0.07	0.543	1722	0.02	-0.08	0.11	0.715
Total cholesterol in VLDL (mmol/l)	1722	0.02	-0.04	0.08	0.509	1722	0.06	0.01	0.12	0.024	1722	0.08	-0.01	0.18	0.082
Remnant cholesterol (non-HDL, non-LDL -cholesterol) (mmol/l)	1722	0.00	-0.05	0.05	0.989	1722	0.04	-0.02	0.09	0.182	1722	0.06	-0.04	0.15	0.261
Total cholesterol in LDL (mmol/l)	1722	-0.02	-0.07	0.02	0.358	1722	0.00	-0.05	0.05	0.996	1722	0.01	-0.09	0.11	0.858
Total cholesterol in HDL (mmol/l)	1722	0.00	-0.06	0.05	0.953	1722	0.00	-0.06	0.07	0.883	1722	-0.03	-0.12	0.06	0.505
Total cholesterol in HDL2 (mmol/l)	1722	-0.01	-0.06	0.05	0.784	1722	-0.01	-0.07	0.05	0.844	1722	-0.04	-0.13	0.04	0.333
Total cholesterol in HDL3 (mmol/l)	1722	0.01	-0.04	0.06	0.725	1722	0.02	-0.04	0.09	0.465	1722	0.00	-0.09	0.08	0.915
Esterified cholesterol (mmol/l)	1722	-0.02	-0.06	0.03	0.453	1722	0.01	-0.04	0.07	0.699	1722	0.02	-0.08	0.11	0.744
Free cholesterol (mmol/l)	1722	0.00	-0.04	0.05	0.904	1722	0.03	-0.02	0.08	0.268	1722	0.02	-0.07	0.11	0.667

Online Table 9 Interactions between fat mass index and lean mass index in relation to cardiometabolic traits at age 18y in ALSPAC

Model 1 & 3 adjusted for lean mass index, age, age*lean mass index, sex, sex*lean mass index, ethnicity, ethnicity*lean mass index, education, education*lean mass index,
Model 2 additionally adjusted for smoking, smoking*lean mass index, alcohol, alcohol*lean mass index, puberty timing, puberty timing*lean mass index

	**Model 1** Interaction of fat mass index at age 10y with lean mass index at age 10y					**Model 2** Interaction of fat mass index at age 18y with lean mass index at age 18y					**Model 3** Interaction of change in fat mass index (age 10-18y) with change in lean mass index (age 10-18y)				
**Standardized outcome at age 18y**	**N**	**Beta**	**LCI**	**UCI**	**P-value**	**N**	**Beta**	**LCI**	**UCI**	**P-value**	**N**	**Beta**	**LCI**	**UCI**	**P-value**
Serum total triglycerides (mmol/l)	1722	0.06	0.00	0.11	0.034	1722	0.11	0.05	0.17	0.0004	1722	0.10	0.01	0.18	0.024
Triglycerides in VLDL (mmol/l)	1722	0.06	0.00	0.11	0.041	1722	0.11	0.05	0.17	0.000	1722	0.10	0.01	0.18	0.022
Triglycerides in LDL (mmol/l)	1722	0.03	-0.02	0.07	0.224	1722	0.06	0.00	0.11	0.035	1722	0.04	-0.05	0.13	0.382
Triglycerides in HDL (mmol/l)	1722	0.05	0.00	0.10	0.068	1722	0.09	0.03	0.14	0.001	1722	0.10	0.01	0.18	0.026
Diacylglycerol (mmol/l)	1722	0.01	-0.04	0.06	0.662	1722	0.04	-0.02	0.11	0.224	1722	0.06	-0.04	0.15	0.250
Ratio of diacylglycerol to triglycerides	1722	-0.02	-0.06	0.03	0.454	1722	-0.01	-0.06	0.04	0.714	1722	0.02	-0.08	0.11	0.747
Total phosphoglycerides (mmol/l)	1722	0.01	-0.03	0.06	0.622	1722	0.06	0.00	0.11	0.045	1722	0.04	-0.04	0.13	0.315
Ratio of triglycerides to phosphoglycerides	1722	0.06	0.00	0.11	0.040	1722	0.09	0.03	0.15	0.002	1722	0.07	-0.02	0.17	0.113
Phosphatidylcholine and other cholines (mmol/l)	1722	0.01	-0.03	0.06	0.558	1722	0.06	-0.01	0.12	0.082	1722	0.04	-0.04	0.12	0.354
Total cholines (mmol/l)	1722	0.01	-0.04	0.05	0.812	1722	0.04	-0.02	0.10	0.164	1722	0.05	-0.04	0.14	0.266
Apolipoprotein A-I (g/l)	1722	0.01	-0.04	0.06	0.760	1722	0.03	-0.03	0.09	0.361	1722	-0.01	-0.09	0.08	0.872
Apolipoprotein B (g/l)	1722	0.02	-0.04	0.07	0.531	1722	0.05	0.00	0.11	0.057	1722	0.06	-0.03	0.16	0.202
Ratio of apolipoprotein B to apolipoprotein A-I	1722	0.02	-0.04	0.08	0.588	1722	0.05	-0.01	0.11	0.087	1722	0.07	-0.02	0.17	0.132
Total fatty acids (mmol/l)	1722	0.03	-0.02	0.08	0.217	1722	0.08	0.02	0.14	0.006	1722	0.06	-0.03	0.15	0.166
Estimated description of fatty acid chain length, not actual carbon number	1722	0.04	0.00	0.08	0.068	1722	0.07	0.02	0.12	0.010	1722	0.00	-0.08	0.09	0.916
Estimated degree of unsaturation	1722	-0.04	-0.09	0.01	0.108	1722	-0.01	-0.06	0.04	0.712	1722	-0.10	-0.18	-0.02	0.019
22:6, docosahexaenoic acid (mmol/l)	1722	-0.01	-0.05	0.03	0.576	1722	0.03	-0.03	0.08	0.304	1722	-0.02	-0.10	0.05	0.540
18:2, linoleic acid (mmol/l)	1722	0.02	-0.03	0.06	0.432	1722	0.06	0.00	0.11	0.034	1722	0.01	-0.08	0.10	0.778
Conjugated linoleic acid (mmol/l)	1722	0.00	-0.04	0.04	0.878	1722	0.03	-0.03	0.08	0.369	1722	0.03	-0.04	0.11	0.357
Omega-3 fatty acids (mmol/l)	1722	0.00	-0.04	0.05	0.898	1722	0.03	-0.03	0.08	0.329	1722	-0.02	-0.12	0.07	0.612
Omega-6 fatty acids (mmol/l)	1722	0.01	-0.03	0.05	0.697	1722	0.05	0.00	0.11	0.044	1722	0.02	-0.07	0.11	0.660
Polyunsaturated fatty acids (mmol/l)	1722	0.01	-0.03	0.05	0.713	1722	0.05	0.00	0.11	0.049	1722	0.02	-0.08	0.11	0.746
Monounsaturated fatty acids; 16:1, 18:1 (mmol/l)	1722	0.04	-0.01	0.09	0.080	1722	0.08	0.03	0.14	0.004	1722	0.08	-0.01	0.17	0.068
Saturated fatty acids (mmol/l)	1722	0.03	-0.02	0.08	0.254	1722	0.09	0.02	0.15	0.006	1722	0.07	-0.01	0.16	0.100
Ratio of 22:6 docosahexaenoic acid to total fatty acids (%)	1722	-0.03	-0.07	0.01	0.150	1722	0.00	-0.05	0.05	0.871	1722	-0.08	-0.15	0.00	0.051
Ratio of 18:2 linoleic acid to total fatty acids (%)	1722	-0.02	-0.07	0.02	0.339	1722	-0.04	-0.10	0.02	0.173	1722	-0.09	-0.18	-0.01	0.036
Ratio of conjugated linoleic acid to total fatty acids (%)	1722	0.00	-0.04	0.04	0.964	1722	0.02	-0.03	0.07	0.444	1722	0.04	-0.03	0.10	0.304
Ratio of omega-3 fatty acids to total fatty acids (%)	1722	-0.02	-0.07	0.02	0.358	1722	-0.04	-0.09	0.02	0.168	1722	-0.11	-0.20	-0.02	0.013
Ratio of omega-6 fatty acids to total fatty acids (%)	1722	-0.05	-0.10	0.00	0.057	1722	-0.05	-0.11	0.00	0.048	1722	-0.10	-0.19	-0.01	0.028
Ratio of polyunsaturated fatty acids to total fatty acids (%)	1722	-0.05	-0.10	0.00	0.044	1722	-0.06	-0.11	-0.01	0.028	1722	-0.12	-0.21	-0.03	0.006
Ratio of monounsaturated fatty acids to total fatty acids (%)	1722	0.05	0.00	0.09	0.066	1722	0.04	-0.01	0.09	0.165	1722	0.08	-0.01	0.17	0.084
Ratio of saturated fatty acids to total fatty acids (%)	1722	0.00	-0.05	0.05	0.978	1722	0.02	-0.03	0.08	0.383	1722	0.04	-0.04	0.13	0.341
Insulin (mu/l)	1722	0.04	-0.02	0.10	0.155	1722	0.15	0.06	0.24	0.001	1722	0.13	0.03	0.23	0.008
Glucose (mmol/l)	1722	0.04	0.01	0.08	0.018	1722	0.02	-0.02	0.06	0.256	1722	0.08	0.01	0.15	0.033
Lactate (mmol/l)	1722	0.01	-0.04	0.05	0.811	1722	0.06	0.02	0.11	0.009	1722	0.12	0.03	0.21	0.011
Pyruvate (mmol/l)	1722	-0.02	-0.07	0.02	0.303	1722	0.03	-0.03	0.08	0.325	1722	0.10	0.02	0.19	0.012
Citrate (mmol/l)	1722	0.03	-0.02	0.08	0.186	1722	-0.02	-0.08	0.04	0.441	1722	-0.05	-0.14	0.04	0.285
Alanine (mmol/l)	1722	0.03	-0.02	0.07	0.212	1722	0.07	0.02	0.12	0.004	1722	0.06	-0.02	0.14	0.152
Glutamine (mmol/l)	1722	0.04	0.00	0.09	0.063	1722	0.03	-0.03	0.08	0.339	1722	-0.01	-0.09	0.08	0.826
Histidine (mmol/l)	1722	0.02	-0.03	0.07	0.459	1722	0.03	-0.02	0.09	0.259	1722	-0.01	-0.10	0.08	0.786
Isoleucine (mmol/l)	1722	0.02	-0.02	0.07	0.328	1722	0.05	0.00	0.11	0.051	1722	0.05	-0.03	0.12	0.254
Leucine (mmol/l)	1722	0.00	-0.03	0.04	0.863	1722	0.01	-0.04	0.05	0.819	1722	0.02	-0.06	0.10	0.614
Valine (mmol/l)	1722	0.01	-0.03	0.05	0.620	1722	-0.01	-0.06	0.04	0.746	1722	0.07	-0.02	0.15	0.118
Phenylalanine (mmol/l)	1722	0.01	-0.04	0.06	0.700	1722	-0.01	-0.06	0.04	0.692	1722	0.00	-0.08	0.08	0.915
Tyrosine (mmol/l)	1722	0.03	-0.02	0.09	0.189	1722	0.05	0.00	0.10	0.065	1722	0.07	-0.01	0.16	0.098
Acetate (mmol/l)	1722	0.00	-0.02	0.02	0.866	1722	0.00	-0.02	0.02	0.961	1722	-0.02	-0.05	0.01	0.120
Acetoacetate (mmol/l)	1722	-0.04	-0.08	0.00	0.071	1722	0.02	-0.04	0.08	0.472	1722	-0.10	-0.18	-0.02	0.017

Online Table 9 Interactions between fat mass index and lean mass index in relation to cardiometabolic traits at age 18y in ALSPAC

Model 1 & 3 adjusted for lean mass index, age, age*lean mass index, sex, sex*lean mass index, ethnicity, ethnicity*lean mass index, education, education*lean mass index,
Model 2 additionally adjusted for smoking, smoking*lean mass index, alcohol, alcohol*lean mass index, puberty timing, puberty timing*lean mass index

	Model 1: Interaction of fat mass index at age 10y with lean mass index at age 10y					Model 2: Interaction of fat mass index at age 18y with lean mass index at age 18y					Model 3: Interaction of change in fat mass index (age 10-18y) with change in lean mass index (age 10-18y)				
**Standardized outcome at age 18y**	**N**	**Beta**	**LCI**	**UCI**	**P-value**	**N**	**Beta**	**LCI**	**UCI**	**P-value**	**N**	**Beta**	**LCI**	**UCI**	**P-value**
3-hydroxybutyrate (mmol/l)	1722	-0.02	-0.07	0.02	0.356	1722	0.05	-0.02	0.12	0.177	1722	-0.05	-0.13	0.04	0.295
Creatinine (mmol/l)	1722	0.04	-0.01	0.08	0.110	1722	0.01	-0.04	0.06	0.681	1722	-0.03	-0.10	0.04	0.428
Albumin (signal area)	1722	0.02	-0.02	0.07	0.317	1722	0.04	-0.01	0.09	0.126	1722	-0.04	-0.13	0.05	0.388
Glycoprotein acetyls, mainly a1-acid glycoprotein (mmol/l)	1722	0.01	-0.04	0.06	0.798	1722	0.07	0.02	0.12	0.004	1722	0.10	0.01	0.18	0.024
C-reactive protein (mg/l)	1722	-0.01	-0.05	0.04	0.744	1722	0.00	-0.05	0.04	0.821	1722	0.03	-0.03	0.10	0.319

Online Table 10 Examining non-linear associations of change in total and regional fat indexes from age 10-18y with summary cardiometabolic traits at age 18y

	**Standardized outcome at age 18y**									
	SBP *Beta (95% CI)*	***DBP*** *Beta (95% CI)*	***Total cholesterol*** *Beta (95% CI)*	***LDL cholesterol*** *Beta (95% CI)*	***HDL cholesterol*** *Beta (95% CI)*	***Triglycerides*** *Beta (95% CI)*	***Insulin*** *Beta (95% CI)*	***Glucose*** *Beta (95% CI)*	***Glycoprotein acetyls*** *Beta (95% CI)*	***CRP*** *Beta (95% CI)*
Change in BMI (SD-unit) [1]	*n=3506*	*n=3506*	*n=2398*	*n=2398*	*n=2398*	*n=2398*	*n=2435*	*n=2397*	*n=2397*	*n=2477*
Lowest tertile (most negative)	0.00	0.00	0.00	0.00	0.00	0.00	0.00	0.00	0.00	0.00
Intermediate tertile	0.26 (0.19, 0.33)	0.20 (0.12, 0.28)	0.07 (-0.02, 0.17)	0.07 (-0.02, 0.17)	-0.09 (-0.19, -0.0004)	0.19 (0.10, 0.28)	0.20 (0.12, 0.29)	0.05 (-0.08, 0.17)	0.16 (0.06, 0.25)	0.05 (-0.03, 0.12)
Highest tertile (most positive)	0.50 (0.43, 0.57)	0.43 (0.35, 0.52)	0.22 (0.12, 0.32)	0.25 (0.15, 0.35)	-0.34 (-0.44, -0.25)	0.54 (0.44, 0.65)	0.52 (0.38, 0.67)	0.18 (0.06, 0.31)	0.52 (0.42, 0.62)	0.14 (0.06, 0.23)
Change in fat mass index (SD-unit) [1]	*n=3409*	*n=3409*	*n=2342*	*n=2342*	*n=2342*	*n=2342*	*n=2378*	*n=2341*	*n=2341*	*n=2419*
Lowest tertile (most negative)	0.00	0.00	0.00	0.00	0.00	0.00	0.00	0.00	0.00	0.00
Intermediate tertile	0.11 (0.04, 0.18)	0.19 (0.10, 0.27)	0.10 (0.002, 0.19)	0.11 (0.01, 0.20)	-0.12 (-0.21, -0.03)	0.18 (0.09, 0.28)	0.24 (0.16, 0.32)	0.02 (-0.11, 0.15)	0.17 (0.08, 0.26)	0.05 (-0.02, 0.13)
Highest tertile (most positive)	0.35 (0.27, 0.43)	0.47 (0.38, 0.56)	0.29 (0.18, 0.40)	0.35 (0.23, 0.46)	-0.36 (-0.46, -0.25)	0.40 (0.29, 0.51)	0.61 (0.43, 0.79)	0.17 (0.04, 0.31)	0.56 (0.44, 0.67)	0.17 (0.07, 0.28)
Change in trunk fat index (SD-unit) [2]	*n=3409*	*n=3409*	*n=2342*	*n=2342*	*n=2342*	*n=2342*	*n=2378*	*n=2341*	*n=2341*	*n=2419*
Lowest tertile (most negative)	0.00	0.00	0.00	0.00	0.00	0.00	0.00	0.00	0.00	0.00
Intermediate tertile	0.13 (0.03, 0.22)	0.11 (0.003, 0.22)	-0.01 (-0.12, 0.11)	-0.02 (-0.13, 0.10)	-0.09 (-0.20, 0.03)	0.13 (0.02, 0.24)	0.21 (0.09, 0.33)	-0.04 (-0.25, 0.17)	0.12 (0.01, 0.24)	0.01 (-0.07, 0.09)
Highest tertile (most positive)	0.29 (0.17, 0.41)	0.28 (0.13, 0.43)	0.11 (-0.06, 0.28)	0.14 (-0.04, 0.31)	-0.29 (-0.46, -0.13)	0.39 (0.22, 0.55)	0.34 (0.03, 0.66)	-0.03 (-0.25, 0.20)	0.41 (0.24, 0.57)	0.09 (-0.03, 0.20)
Change in arm fat index (SD-unit) [2]	*n=3409*	*n=3409*	*n=2342*	*n=2342*	*n=2342*	*n=2342*	*n=2378*	*n=2341*	*n=2341*	*n=2419*
Lowest tertile (most negative)	0.00	0.00	0.00	0.00	0.00	0.00	0.00	0.00	0.00	0.00
Intermediate tertile	0.08 (-0.03, 0.19)	0.17 (0.04, 0.29)	0.20 (0.06, 0.34)	0.21 (0.07, 0.35)	0.06 (-0.07, 0.19)	0.06 (-0.08, 0.20)	0.14 (-0.06, 0.33)	-0.07 (-0.34, 0.20)	0.03 (-0.12, 0.18)	0.05 (-0.06, 0.17)
Highest tertile (most positive)	0.11 (-0.04, 0.25)	0.19 (0.02, 0.36)	0.27 (0.06, 0.47)	0.28 (0.07, 0.49)	0.05 (-0.15, 0.24)	0.19 (-0.03, 0.41)	0.17 (-0.04, 0.39)	-0.02 (-0.29, 0.25)	0.15 (-0.06, 0.37)	0.15 (-0.02, 0.32)
Change in leg fat index (SD-unit) [2]	*n=3409*	*n=3409*	*n=2342*	*n=2342*	*n=2342*	*n=2342*	*n=2378*	*n=2341*	*n=2341*	*n=2419*
Lowest tertile (most negative)	0.00	0.00	0.00	0.00	0.00	0.00	0.00	0.00	0.00	0.00
Intermediate tertile	-0.02 (-0.13, 0.09)	0.04 (-0.10, 0.17)	-0.03 (-0.18, 0.12)	-0.02 (-0.17, 0.13)	-0.06 (-0.19, 0.08)	0.02 (-0.13, 0.16)	-0.01 (-0.26, 0.23)	0.24 (-0.11, 0.59)	0.03 (-0.12, 0.19)	-0.02 (-0.16, 0.11)
Highest tertile (most positive)	0.07 (-0.08, 0.21)	0.16 (-0.02, 0.33)	-0.004 (-0.21, 0.20)	0.01 (-0.20, 0.21)	-0.10 (-0.29, 0.09)	0.05 (-0.17, 0.27)	0.18 (-0.16, 0.51)	0.31 (-0.02, 0.64)	0.11 (-0.11, 0.33)	-0.04 (-0.22, 0.14)
Change in lean mass index (SD-unit) [1]	*n=3409*	*n=3409*	*n=2342*	*n=2342*	*n=2342*	*n=2342*	*n=2378*	*n=2341*	*n=2341*	*n=2419*
Lowest tertile (most negative)	0.00	0.00	0.00	0.00	0.00	0.00	0.00	0.00	0.00	0.00
Intermediate tertile	0.17 (0.09, 0.24)	0.05 (-0.04, 0.14)	-0.03 (-0.14, 0.07)	-0.03 (-0.13, 0.08)	-0.09 (-0.19, 0.02)	0.10 (-0.01, 0.20)	0.14 (-0.02, 0.31)	-0.04 (-0.18, 0.10)	0.13 (0.02, 0.24)	0.04 (-0.06, 0.13)
Highest tertile (most positive)	0.33 (0.23, 0.42)	0.07 (-0.04, 0.17)	0.04 (-0.08, 0.16)	0.03 (-0.09, 0.15)	-0.07 (-0.19, 0.05)	0.23 (0.11, 0.36)	0.17 (-0.01, 0.35)	-0.004 (-0.19, 0.19)	0.15 (0.03, 0.28)	0.04 (-0.06, 0.13)

[1] Models adjusted for age, sex, ethnicity, maternal education, index at age 10y

[2] Models adjusted for age, sex, ethnicity, maternal education, index at age 10y, change in other regional indexes

Online Table 11 Comparing variance explained in cardiometabolic traits by change (age 10-18y) versus current (age 18y) values of body mass index (BMI), fat mass index, and lean mass index in ALSPAC

	BMI				Fat mass index				Lean mass index			
	Adj. for age, sex, ethnicity, maternal education				*Adj. for age, sex, ethnicity, maternal education*				*Adj. for age, sex, ethnicity, maternal education*			
	Change		Current		Change		Current		Change		Current	
**Standardized outcome at age 18y**	N	% R^2	N	% R^2	N	% R^2	N	% R^2	N	% R^2	N	% R^2
Systolic blood pressure (mmHg)	3635	28.34	4067	34.28	3409	27.59	3988	32.57	3409	26.87	3988	31.56
Diastolic blood pressure (mmHg)	3635	4.26	4067	11.53	3409	4.34	3988	13.91	3409	3.21	3988	3.73
Concentration of chylomicrons and extremely large VLDL particles (mol/l)	2472	7.05	2775	11.34	2342	6.03	2724	11.58	2342	1.59	2724	3.06
Total lipids in chylomicrons and extremely large VLDL (mmol/l)	2472	6.89	2775	11.11	2342	5.84	2724	11.27	2342	1.59	2724	3.09
Phospholipids in chylomicrons and extremely large VLDL (mmol/l)	2472	6.50	2775	10.64	2342	5.50	2724	10.88	2342	1.38	2724	2.84
Total cholesterol in chylomicrons and extremely large VLDL (mmol/l)	2472	6.38	2775	10.38	2342	5.21	2724	10.33	2342	0.93	2724	2.57
Cholesterol esters in chylomicrons and extremely large VLDL (mmol/l)	2472	6.15	2775	9.87	2342	4.93	2724	9.68	2342	0.73	2724	2.38
Free cholesterol in chylomicrons and extremely large VLDL (mmol/l)	2472	6.37	2775	10.45	2342	5.34	2724	10.60	2342	1.25	2724	2.76
Triglycerides in chylomicrons and extremely large VLDL (mmol/l)	2472	7.08	2775	11.32	2342	6.06	2724	11.53	2342	1.85	2724	3.29
Concentration of very large VLDL particles (mol/l)	2472	7.36	2775	11.55	2342	6.19	2724	11.46	2342	2.22	2724	3.84
Total lipids in very large VLDL (mmol/l)	2472	7.16	2775	11.30	2342	5.96	2724	11.18	2342	2.05	2724	3.70
Phospholipids in very large VLDL (mmol/l)	2472	6.46	2775	10.45	2342	5.33	2724	10.45	2342	1.35	2724	2.96
Total cholesterol in very large VLDL (mmol/l)	2472	7.04	2775	11.35	2342	5.86	2724	11.38	2342	1.46	2724	3.07
Cholesterol esters in very large VLDL (mmol/l)	2472	7.61	2775	12.05	2342	6.35	2724	11.98	2342	1.88	2724	3.50
Free cholesterol in very large VLDL (mmol/l)	2472	6.41	2775	10.53	2342	5.33	2724	10.67	2342	1.06	2724	2.62
Triglycerides in very large VLDL (mmol/l)	2472	7.34	2775	11.42	2342	6.14	2724	11.22	2342	2.46	2724	4.12
Concentration of large VLDL particles (mol/l)	2472	7.07	2775	11.16	2342	5.76	2724	10.89	2342	2.23	2724	3.96
Total lipids in large VLDL (mmol/l)	2472	6.97	2775	11.09	2342	5.65	2724	10.83	2342	2.06	2724	3.78
Phospholipids in large VLDL (mmol/l)	2472	6.51	2775	10.58	2342	5.22	2724	10.37	2342	1.62	2724	3.33
Total cholesterol in large VLDL (mmol/l)	2472	6.55	2775	10.63	2342	5.21	2724	10.43	2342	1.33	2724	3.02
Cholesterol esters in large VLDL (mmol/l)	2472	6.58	2775	10.71	2342	5.20	2724	10.51	2342	1.15	2724	2.82
Free cholesterol in large VLDL (mmol/l)	2472	6.43	2775	10.38	2342	5.16	2724	10.19	2342	1.50	2724	3.19
Triglycerides in large VLDL (mmol/l)	2472	7.30	2775	11.41	2342	6.01	2724	11.12	2342	2.56	2724	4.29
Concentration of medium VLDL particles (mol/l)	2472	7.18	2775	11.38	2342	5.84	2724	11.18	2342	2.14	2724	3.82
Total lipids in medium VLDL (mmol/l)	2472	6.97	2775	11.20	2342	5.61	2724	11.00	2342	1.80	2724	3.49
Phospholipids in medium VLDL (mmol/l)	2472	6.17	2775	10.13	2342	4.89	2724	9.99	2342	1.25	2724	2.84
Total cholesterol in medium VLDL (mmol/l)	2472	5.78	2775	9.48	2342	4.55	2724	9.39	2342	0.72	2724	2.20
Cholesterol esters in medium VLDL (mmol/l)	2472	5.75	2775	9.08	2342	4.62	2724	9.07	2342	0.80	2724	2.12
Free cholesterol in medium VLDL (mmol/l)	2472	5.74	2775	9.51	2342	4.51	2724	9.36	2342	1.08	2724	2.62
Triglycerides in medium VLDL (mmol/l)	2472	8.33	2775	12.65	2342	6.99	2724	12.41	2342	3.36	2724	5.06
Concentration of small VLDL particles (mol/l)	2472	5.63	2775	8.55	2342	4.56	2724	8.61	2342	1.14	2724	2.26
Total lipids in small VLDL (mmol/l)	2472	5.81	2775	8.65	2342	4.80	2724	8.93	2342	1.17	2724	2.15
Phospholipids in small VLDL (mmol/l)	2472	6.14	2775	8.46	2342	5.27	2724	8.64	2342	2.28	2724	3.02
Total cholesterol in small VLDL (mmol/l)	2472	5.50	2775	7.74	2342	4.83	2724	8.44	2342	1.28	2724	1.98
Cholesterol esters in small VLDL (mmol/l)	2472	4.87	2775	6.93	2342	4.35	2724	7.77	2342	0.87	2724	1.49
Free cholesterol in small VLDL (mmol/l)	2472	5.96	2775	8.00	2342	5.16	2724	8.25	2342	2.29	2724	2.92
Triglycerides in small VLDL (mmol/l)	2472	5.38	2775	8.31	2342	4.28	2724	8.17	2342	1.29	2724	2.51
Concentration of very small VLDL particles (mol/l)	2472	8.97	2775	9.40	2342	8.78	2724	9.88	2342	7.50	2724	7.62
Total lipids in very small VLDL (mmol/l)	2472	5.65	2775	6.48	2342	5.35	2724	7.06	2342	3.64	2724	3.95
Phospholipids in very small VLDL (mmol/l)	2472	10.29	2775	10.51	2342	10.32	2724	11.10	2342	9.39	2724	9.52
Total cholesterol in very small VLDL (mmol/l)	2472	2.55	2775	3.30	2342	2.29	2724	3.86	2342	0.77	2724	0.99
Cholesterol esters in very small VLDL (mmol/l)	2472	2.71	2775	3.71	2342	2.45	2724	4.35	2342	0.52	2724	0.71
Free cholesterol in very small VLDL (mmol/l)	2472	5.17	2775	5.34	2342	5.06	2724	5.68	2342	4.54	2724	4.71
Triglycerides in very small VLDL (mmol/l)	2472	7.26	2775	7.87	2342	6.98	2724	7.94	2342	5.45	2724	5.40
Concentration of IDL particles (mol/l)	2472	10.39	2775	10.48	2342	10.64	2724	10.96	2342	10.06	2724	10.15
Total lipids in IDL (mmol/l)	2472	9.76	2775	9.91	2342	9.91	2724	10.47	2342	9.23	2724	9.38
Phospholipids in IDL (mmol/l)	2472	11.68	2775	11.75	2342	12.03	2724	12.26	2342	11.60	2724	11.68
Total cholesterol in IDL (mmol/l)	2472	7.13	2775	7.38	2342	7.18	2724	8.00	2342	6.33	2724	6.55
Cholesterol esters in IDL (mmol/l)	2472	6.02	2775	6.45	2342	5.97	2724	7.15	2342	4.80	2724	5.02
Free cholesterol in IDL (mmol/l)	2472	10.46	2775	10.50	2342	10.70	2724	10.90	2342	10.45	2724	10.68
Triglycerides in IDL (mmol/l)	2472	17.11	2775	16.36	2342	17.57	2724	16.64	2342	17.46	2724	16.55
Concentration of large LDL particles (mol/l)	2472	12.96	2775	13.09	2342	13.35	2724	13.68	2342	12.77	2724	12.70
Total lipids in large LDL (mmol/l)	2472	12.42	2775	12.55	2342	12.71	2724	13.17	2342	12.08	2724	12.07
Phospholipids in large LDL (mmol/l)	2472	12.06	2775	12.32	2342	12.32	2724	13.03	2342	11.51	2724	11.51
Total cholesterol in large LDL (mmol/l)	2472	10.92	2775	11.10	2342	11.15	2724	11.75	2342	10.47	2724	10.55
Cholesterol esters in large LDL (mmol/l)	2472	10.72	2775	10.97	2342	10.93	2724	11.67	2342	10.12	2724	10.18
Free cholesterol in large LDL (mmol/l)	2472	11.46	2775	11.51	2342	11.73	2724	11.99	2342	11.37	2724	11.55
Triglycerides in large LDL (mmol/l)	2472	19.27	2775	18.64	2342	19.93	2724	18.89	2342	19.92	2724	18.90
Concentration of medium LDL particles (mol/l)	2472	12.83	2775	13.06	2342	13.25	2724	13.74	2342	12.48	2724	12.32
Total lipids in medium LDL (mmol/l)	2472	12.33	2775	12.54	2342	12.62	2724	13.21	2342	11.88	2724	11.82
Phospholipids in medium LDL (mmol/l)	2472	12.49	2775	12.99	2342	12.59	2724	13.85	2342	11.46	2724	11.34
Total cholesterol in medium LDL (mmol/l)	2472	10.57	2775	10.81	2342	10.82	2724	11.48	2342	10.07	2724	10.11
Cholesterol esters in medium LDL (mmol/l)	2472	10.57	2775	10.83	2342	10.85	2724	11.50	2342	10.07	2724	10.10
Free cholesterol in medium LDL (mmol/l)	2472	10.32	2775	10.47	2342	10.44	2724	11.12	2342	9.83	2724	9.94
Triglycerides in medium LDL (mmol/l)	2472	20.78	2775	20.32	2342	21.74	2724	20.59	2342	21.72	2724	20.61
Concentration of small LDL particles (mol/l)	2472	11.91	2775	12.16	2342	12.28	2724	12.82	2342	11.60	2724	11.38
Total lipids in small LDL (mmol/l)	2472	11.40	2775	11.68	2342	11.67	2724	12.38	2342	10.91	2724	10.85
Phospholipids in small LDL (mmol/l)	2472	11.47	2775	11.87	2342	11.62	2724	12.62	2342	10.78	2724	10.54
Total cholesterol in small LDL (mmol/l)	2472	10.12	2775	10.38	2342	10.37	2724	11.06	2342	9.66	2724	9.74
Cholesterol esters in small LDL (mmol/l)	2472	10.49	2775	10.75	2342	10.82	2724	11.43	2342	10.10	2724	10.13
Free cholesterol in small LDL (mmol/l)	2472	7.89	2775	8.13	2342	7.87	2724	8.76	2342	7.30	2724	7.46
Triglycerides in small LDL (mmol/l)	2472	14.83	2775	14.63	2342	15.45	2724	14.90	2342	14.93	2724	14.00
Concentration of very large HDL particles (mol/l)	2472	15.16	2775	16.64	2342	15.33	2724	16.56	2342	13.62	2724	13.87
Total lipids in very large HDL (mmol/l)	2472	12.22	2775	13.66	2342	12.33	2724	13.48	2342	10.70	2724	11.08
Phospholipids in very large HDL (mmol/l)	2472	17.76	2775	19.47	2342	17.91	2724	19.44	2342	15.85	2724	16.06
Total cholesterol in very large HDL (mmol/l)	2472	5.80	2775	6.80	2342	5.81	2724	6.51	2342	4.74	2724	5.22
Cholesterol esters in very large HDL (mmol/l)	2472	4.15	2775	4.98	2342	4.13	2724	4.70	2342	3.28	2724	3.74
Free cholesterol in very large HDL (mmol/l)	2472	10.99	2775	12.34	2342	11.11	2724	12.13	2342	9.55	2724	9.90
Triglycerides in very large HDL (mmol/l)	2472	6.13	2775	6.07	2342	6.33	2724	6.10	2342	6.32	2724	6.14
Concentration of large HDL particles (mol/l)	2472	21.95	2775	23.13	2342	22.20	2724	23.40	2342	19.51	2724	19.13
Total lipids in large HDL (mmol/l)	2472	20.74	2775	21.98	2342	20.91	2724	22.16	2342	18.22	2724	17.94
Phospholipids in large HDL (mmol/l)	2472	22.91	2775	23.65	2342	23.30	2724	23.81	2342	21.07	2724	20.49
Total cholesterol in large HDL (mmol/l)	2472	18.36	2775	20.14	2342	18.27	2724	20.30	2342	15.14	2724	15.21
Cholesterol esters in large HDL (mmol/l)	2472	18.34	2775	20.16	2342	18.24	2724	20.35	2342	15.08	2724	15.14
Free cholesterol in large HDL (mmol/l)	2472	18.29	2775	19.93	2342	18.24	2724	19.97	2342	15.28	2724	15.38
Triglycerides in large HDL (mmol/l)	2472	15.15	2775	14.29	2342	15.77	2724	14.66	2342	15.71	2724	14.75
Concentration of medium HDL particles (mol/l)	2472	18.94	2775	18.27	2342	19.41	2724	18.40	2342	19.23	2724	18.33
Total lipids in medium HDL (mmol/l)	2472	18.11	2775	17.44	2342	18.55	2724	17.53	2342	18.23	2724	17.36
Phospholipids in medium HDL (mmol/l)	2472	20.73	2775	20.14	2342	21.21	2724	20.26	2342	20.91	2724	20.01
Total cholesterol in medium HDL (mmol/l)	2472	13.14	2775	12.48	2342	13.38	2724	12.46	2342	12.73	2724	12.08
Cholesterol esters in medium HDL (mmol/l)	2472	12.29	2775	11.63	2342	12.50	2724	11.60	2342	11.75	2724	11.14
Free cholesterol in medium HDL (mmol/l)	2472	16.65	2775	15.78	2342	17.02	2724	15.84	2342	16.75	2724	15.76
Triglycerides in medium HDL (mmol/l)	2472	8.69	2775	11.12	2342	8.30	2724	10.91	2342	6.36	2724	7.40
Concentration of small HDL particles (mol/l)	2472	9.09	2775	9.35	2342	9.22	2724	9.33	2342	9.26	2724	8.85
Total lipids in small HDL (mmol/l)	2472	15.45	2775	15.13	2342	15.77	2724	15.13	2342	15.84	2724	15.06
Phospholipids in small HDL (mmol/l)	2472	4.85	2775	4.94	2342	4.88	2724	4.85	2342	4.99	2724	4.81
Total cholesterol in small HDL (mmol/l)	2472	26.37	2775	25.95	2342	27.00	2724	26.03	2342	26.97	2724	26.12
Cholesterol esters in small HDL (mmol/l)	2472	28.19	2775	27.78	2342	28.86	2724	27.92	2342	28.85	2724	28.15
Free cholesterol in small HDL (mmol/l)	2472	9.41	2775	8.94	2342	9.58	2724	8.90	2342	9.34	2724	8.67
Triglycerides in small HDL (mmol/l)	2472	6.07	2775	7.18	2342	5.63	2724	6.72	2342	4.38	2724	4.98
Phospholipids to total lipds ratio in chylomicrons and extremely large VLDL (%)	2472	1.30	2775	1.11	2342	1.32	2724	1.08	2342	1.26	2724	1.09
Total cholesterol to total lipids ratio in chylomicrons and extremely large VLDL (%)	2472	5.75	2775	5.82	2342	5.69	2724	5.81	2342	4.98	2724	5.01
Cholesterol esters to total lipids ratio in chylomicrons and extremely large VLDL (%)	2472	4.44	2775	4.10	2342	4.41	2724	4.07	2342	3.79	2724	3.79
Free cholesterol to total lipids ratio in chylomicrons and extremely large VLDL (%)	2472	4.33	2775	5.96	2342	4.42	2724	6.20	2342	4.25	2724	4.25
Triglycerides to total lipids ratio in chylomicrons and extremely large VLDL (%)	2472	5.18	2775	5.98	2342	5.18	2724	5.91	2342	4.78	2724	5.42

Online Table 11 Comparing variance explained in cardiometabolic traits by change (age 10-18y) versus current (age 18y) values of body mass index (BMI), fat mass index, and lean mass index in ALSPAC

	**BMI**				**Fat mass index**				**Lean mass index**			
	Adj. for age, sex, ethnicity, maternal education				*Adj. for age, sex, ethnicity, maternal education*				*Adj. for age, sex, ethnicity, maternal education*			
	**Change**		**Current**		**Change**		**Current**		**Change**		**Current**	
**Standardized outcome at age 18y**	**N**	**% R^2**	**N**	**% R^2**	**N**	**% R^2**	**N**	**% R^2**	**N**	**% R^2**	**N**	**% R^2**
Phospholipids to total lipids ratio in very large VLDL (%)	2472	3.89	2775	6.05	2342	3.73	2724	6.48	2342	3.00	2724	3.24
Total cholesterol to total lipids ratio in very large VLDL (%)	2472	1.92	2775	2.33	2342	1.88	2724	2.41	2342	1.87	2724	2.02
Cholesterol esters to total lipids ratio in very large VLDL (%)	2472	0.65	2775	0.71	2342	0.62	2724	0.79	2342	0.63	2724	0.50
Free cholesterol to total lipids ratio in very large VLDL (%)	2472	3.15	2775	3.32	2342	3.12	2724	3.30	2342	3.03	2724	3.30
Triglycerides to total lipids ratio in very large VLDL (%)	2472	5.16	2775	5.30	2342	5.18	2724	5.38	2342	5.18	2724	5.38
Phospholipids to total lipds ratio in large VLDL (%)	2472	1.83	2775	3.34	2342	1.77	2724	3.58	2342	1.11	2724	1.22
Total cholesterol to total lipids ratio in large VLDL (%)	2472	4.04	2775	4.60	2342	3.84	2724	4.51	2342	2.81	2724	3.16
Cholesterol esters to total lipids ratio in large VLDL (%)	2472	5.22	2775	4.96	2342	5.25	2724	5.01	2342	5.05	2724	5.09
Free cholesterol to total lipids ratio in large VLDL (%)	2472	0.59	2775	0.80	2342	0.47	2724	0.81	2342	0.32	2724	0.28
Triglycerides to total lipids ratio in large VLDL (%)	2472	3.20	2775	4.15	2342	3.05	2724	4.13	2342	2.06	2724	2.32
Phospholipids to total lipds ratio in medium VLDL (%)	2472	20.48	2775	20.80	2342	20.45	2724	20.82	2342	18.76	2724	18.60
Total cholesterol to total lipids ratio in medium VLDL (%)	2472	10.40	2775	10.23	2342	10.66	2724	10.64	2342	10.30	2724	10.12
Cholesterol esters to total lipids ratio in medium VLDL (%)	2472	9.25	2775	8.96	2342	9.53	2724	9.41	2342	9.18	2724	9.10
Free cholesterol to total lipids ratio in medium VLDL (%)	2472	6.34	2775	6.59	2342	6.36	2724	6.73	2342	6.24	2724	5.97
Triglycerides to total lipids ratio in medium VLDL (%)	2472	13.57	2775	13.31	2342	13.96	2724	13.77	2342	13.88	2724	13.63
Phospholipids to total lipds ratio in small VLDL (%)	2472	7.09	2775	8.38	2342	6.58	2724	8.62	2342	4.08	2724	4.00
Total cholesterol to total lipids ratio in small VLDL (%)	2472	2.52	2775	2.59	2342	2.53	2724	2.66	2342	2.52	2724	2.93
Cholesterol esters to total lipids ratio in small VLDL (%)	2472	1.61	2775	1.52	2342	1.68	2724	1.68	2342	1.54	2724	1.73
Free cholesterol to total lipids ratio in small VLDL (%)	2472	20.67	2775	21.38	2342	20.66	2724	21.69	2342	18.98	2724	18.49
Triglycerides to total lipids ratio in small VLDL (%)	2472	4.55	2775	5.13	2342	4.31	2724	4.95	2342	4.31	2724	5.03
Phospholipids to total lipds ratio in very small VLDL (%)	2472	20.64	2775	20.38	2342	21.46	2724	20.65	2342	21.48	2724	20.80
Total cholesterol to total lipids ratio in very small VLDL (%)	2472	11.05	2775	10.49	2342	11.72	2724	10.53	2342	11.71	2724	10.55
Cholesterol esters to total lipids ratio in very small VLDL (%)	2472	14.65	2775	13.98	2342	15.24	2724	14.17	2342	15.14	2724	13.96
Free cholesterol to total lipids ratio in very small VLDL (%)	2472	3.60	2775	5.34	2342	3.73	2724	5.86	2342	1.18	2724	1.72
Triglycerides to total lipids ratio in very small VLDL (%)	2472	1.89	2775	1.72	2342	1.96	2724	1.59	2342	1.94	2724	1.79
Phospholipids to total lipds ratio in IDL (%)	2472	6.18	2775	7.85	2342	5.85	2724	7.80	2342	3.96	2724	3.85
Total cholesterol to total lipids ratio in IDL (%)	2472	6.54	2775	6.64	2342	6.72	2724	6.86	2342	6.07	2724	5.08
Cholesterol esters to total lipids ratio in IDL (%)	2472	14.33	2775	15.44	2342	14.46	2724	15.68	2342	12.71	2724	11.50
Free cholesterol to total lipids ratio in IDL (%)	2472	8.86	2775	10.35	2342	8.46	2724	9.89	2342	7.29	2724	8.23
Triglycerides to total lipids ratio in IDL (%)	2472	4.98	2775	4.57	2342	5.17	2724	4.65	2342	5.01	2724	4.14
Phospholipids to total lipds ratio in large LDL (%)	2472	12.39	2775	12.40	2342	12.75	2724	12.73	2342	12.66	2724	12.51
Total cholesterol to total lipids ratio in large LDL (%)	2472	2.44	2775	2.68	2342	2.47	2724	3.21	2342	2.09	2724	2.11
Cholesterol esters to total lipids ratio in large LDL (%)	2472	4.92	2775	5.75	2342	4.87	2724	6.50	2342	4.02	2724	4.01
Free cholesterol to total lipids ratio in large LDL (%)	2472	9.56	2775	11.11	2342	9.29	2724	11.36	2342	7.89	2724	7.68
Triglycerides to total lipids ratio in large LDL (%)	2472	5.41	2775	5.34	2342	5.51	2724	5.59	2342	5.04	2724	4.27
Phospholipids to total lipds ratio in medium LDL (%)	2472	10.29	2775	10.26	2342	10.67	2724	10.55	2342	10.61	2724	10.41
Total cholesterol to total lipids ratio in medium LDL (%)	2472	2.92	2775	3.08	2342	3.01	2724	3.43	2342	2.80	2724	2.80
Cholesterol esters to total lipids ratio in medium LDL (%)	2472	6.93	2775	7.25	2342	7.13	2724	7.71	2342	6.78	2724	6.68
Free cholesterol to total lipids ratio in medium LDL (%)	2472	13.39	2775	13.77	2342	13.82	2724	14.24	2342	13.35	2724	12.91
Triglycerides to total lipids ratio in medium LDL (%)	2472	13.02	2775	12.84	2342	13.31	2724	12.91	2342	12.98	2724	11.87
Phospholipids to total lipds ratio in small LDL (%)	2472	9.53	2775	9.68	2342	9.82	2724	10.12	2342	9.56	2724	9.48
Total cholesterol to total lipids ratio in small LDL (%)	2472	4.59	2775	4.68	2342	4.73	2724	5.03	2342	4.58	2724	4.71
Cholesterol esters to total lipids ratio in small LDL (%)	2472	8.36	2775	8.54	2342	8.65	2724	9.04	2342	8.37	2724	8.27
Free cholesterol to total lipids ratio in small LDL (%)	2472	13.92	2775	14.19	2342	14.58	2724	14.72	2342	14.12	2724	13.41
Triglycerides to total lipids ratio in small LDL (%)	2472	6.51	2775	6.15	2342	6.70	2724	5.96	2342	6.64	2724	6.04
Phospholipids to total lipds ratio in very large HDL (%)	2472	21.36	2775	23.46	2342	21.15	2724	23.35	2342	19.19	2724	19.10
Total cholesterol to total lipids ratio in very large HDL (%)	2472	21.88	2775	23.40	2342	21.89	2724	23.47	2342	20.48	2724	20.12
Cholesterol esters to total lipids ratio in very large HDL (%)	2472	20.58	2775	22.06	2342	20.57	2724	22.09	2342	19.15	2724	18.87
Free cholesterol to total lipids ratio in very large HDL (%)	2472	0.81	2775	1.13	2342	0.80	2724	0.95	2342	0.58	2724	0.99
Triglycerides to total lipids ratio in very large HDL (%)	2472	3.12	2775	5.15	2342	2.33	2724	4.51	2342	0.41	2724	1.89
Phospholipids to total lipds ratio in large HDL (%)	2472	6.59	2775	11.69	2342	5.55	2724	12.30	2342	1.82	2724	3.50
Total cholesterol to total lipids ratio in large HDL (%)	2472	6.41	2775	11.98	2342	5.17	2724	12.14	2342	0.85	2724	2.98
Cholesterol esters to total lipids ratio in large HDL (%)	2472	6.14	2775	11.77	2342	4.96	2724	12.18	2342	0.68	2724	2.61
Free cholesterol to total lipids ratio in large HDL (%)	2472	4.57	2775	7.80	2342	3.67	2724	7.17	2342	1.19	2724	3.13
Triglycerides to total lipids ratio in large HDL (%)	2472	7.55	2775	11.35	2342	6.85	2724	10.70	2342	3.44	2724	5.16
Phospholipids to total lipds ratio in medium HDL (%)	2472	9.91	2775	10.23	2342	10.10	2724	10.25	2342	10.09	2724	9.99
Total cholesterol to total lipids ratio in medium HDL (%)	2472	7.72	2775	8.01	2342	7.67	2724	7.92	2342	7.21	2724	7.46
Cholesterol esters to total lipids ratio in medium HDL (%)	2472	9.14	2775	9.24	2342	9.10	2724	9.19	2342	8.50	2724	8.55
Free cholesterol to total lipids ratio in medium HDL (%)	2472	0.57	2775	0.46	2342	0.49	2724	0.49	2342	0.49	2724	0.39
Triglycerides to total lipids ratio in medium HDL (%)	2472	4.93	2775	8.49	2342	4.13	2724	8.02	2342	0.60	2724	2.56
Phospholipids to total lipds ratio in small HDL (%)	2472	23.02	2775	23.22	2342	23.43	2724	23.31	2342	23.43	2724	23.66
Total cholesterol to total lipids ratio in small HDL (%)	2472	23.05	2775	23.70	2342	23.41	2724	23.50	2342	23.24	2724	23.72
Cholesterol esters to total lipids ratio in small HDL (%)	2472	22.47	2775	22.75	2342	22.84	2724	22.67	2342	22.83	2724	23.24
Free cholesterol to total lipids ratio in small HDL (%)	2472	8.01	2775	9.64	2342	7.80	2724	10.58	2342	5.14	2724	5.64
Triglycerides to total lipids ratio in small HDL (%)	2472	2.93	2775	4.41	2342	2.29	2724	3.81	2342	0.64	2724	1.93
Mean diameter for VLDL particles (nm)	2472	9.69	2775	13.20	2342	8.59	2724	12.87	2342	6.07	2724	7.46
Mean diameter for LDL particles (nm)	2472	4.59	2775	5.18	2342	4.44	2724	5.63	2342	4.43	2724	3.87
Mean diameter for HDL particles (nm)	2472	15.54	2775	17.99	2342	15.46	2724	18.03	2342	12.99	2724	13.65
Serum total cholesterol (mmol/l)	2472	13.93	2775	14.01	2342	14.29	2724	14.60	2342	13.87	2724	13.69
Total cholesterol in VLDL (mmol/l)	2472	5.67	2775	8.84	2342	4.67	2724	9.29	2342	0.66	2724	1.68
Remnant cholesterol (non-HDL, non-LDL -cholesterol) (mmol/l)	2472	5.41	2775	7.11	2342	4.90	2724	7.86	2342	2.18	2724	2.64
Total cholesterol in LDL (mmol/l)	2472	10.69	2775	10.90	2342	10.93	2724	11.56	2342	10.22	2724	10.29
Total cholesterol in HDL (mmol/l)	2472	20.67	2775	21.29	2342	20.99	2724	21.19	2342	19.20	2724	18.92
Total cholesterol in HDL2 (mmol/l)	2472	19.48	2775	20.51	2342	19.55	2724	20.31	2342	17.27	2724	17.23
Total cholesterol in HDL3 (mmol/l)	2472	21.20	2775	21.17	2342	21.86	2724	21.25	2342	20.94	2724	20.28
Esterified cholesterol (mmol/l)	2462	12.55	2765	12.69	2332	12.95	2714	13.30	2332	12.46	2714	12.39
Free cholesterol (mmol/l)	2460	14.86	2763	14.76	2330	15.10	2712	15.21	2330	14.81	2712	14.35
Serum total triglycerides (mmol/l)	2472	4.99	2775	8.00	2342	3.98	2724	7.86	2342	1.08	2724	2.34
Triglycerides in VLDL (mmol/l)	2472	6.73	2775	10.61	2342	5.45	2724	10.42	2342	1.93	2724	3.53
Triglycerides in LDL (mmol/l)	2472	19.12	2775	18.55	2342	19.89	2724	18.81	2342	19.84	2724	18.77
Triglycerides in HDL (mmol/l)	2472	9.66	2775	10.53	2342	9.55	2724	10.26	2342	8.61	2724	8.94
Diacylglycerol (mmol/l)	2400	2.83	2697	4.74	2276	1.97	2647	4.12	2276	1.01	2647	2.01
Ratio of diacylglycerol to triglycerides	2401	0.69	2698	1.27	2277	0.47	2648	1.09	2277	0.53	2648	0.79
Total phosphoglycerides (mmol/l)	2460	18.47	2763	18.00	2330	19.10	2712	18.17	2330	19.10	2712	18.17
Ratio of triglycerides to phosphoglycerides	2460	7.52	2763	11.41	2330	6.70	2712	10.97	2330	3.90	2712	5.64
Phosphatidylcholine and other cholines (mmol/l)	2443	18.61	2743	18.05	2314	19.35	2693	18.30	2314	19.38	2693	18.30
Total cholines (mmol/l)	2462	20.09	2765	19.58	2332	21.00	2714	19.85	2332	21.02	2714	19.85
Apolipoprotein A-I (g/l)	2472	22.11	2775	21.84	2342	22.85	2724	21.91	2342	22.34	2724	21.57
Apolipoprotein B (g/l)	2472	5.88	2775	7.79	2342	5.34	2724	8.46	2342	2.40	2724	2.88
Ratio of apolipoprotein B to apolipoprotein A-I	2472	5.93	2775	8.68	2342	5.09	2724	9.06	2342	0.98	2724	1.80
Total fatty acids (mmol/l)	2462	11.56	2765	12.20	2332	11.53	2714	12.47	2332	10.63	2714	10.21
Estimated description of fatty acid chain length, not actual carbon number	2463	0.89	2766	0.94	2333	1.14	2715	0.94	2333	1.16	2715	1.02
Estimated degree of unsaturation	2462	4.01	2765	4.18	2332	4.08	2714	4.18	2332	3.88	2714	3.96
22:6, docosahexaenoic acid (mmol/l)	2462	14.58	2765	14.60	2332	14.80	2714	14.77	2332	14.65	2714	14.50
18:2, linoleic acid (mmol/l)	2462	13.63	2765	13.30	2332	14.16	2714	13.77	2332	14.03	2714	13.38
Conjugated linoleic acid (mmol/l)	2461	1.32	2764	1.64	2331	1.31	2713	1.61	2331	1.15	2713	1.08
Omega-3 fatty acids (mmol/l)	2462	7.29	2765	8.07	2332	7.76	2714	8.27	2332	7.36	2714	7.12
Omega-6 fatty acids (mmol/l)	2462	14.53	2765	14.39	2332	14.81	2714	14.82	2332	14.50	2714	14.01
Polyunsaturated fatty acids (mmol/l)	2462	14.25	2765	14.20	2332	14.58	2714	14.63	2332	14.24	2714	13.69
Monounsaturated fatty acids; 16:1, 18:1 (mmol/l)	2462	8.65	2765	9.99	2332	8.50	2714	10.39	2332	6.83	2714	6.65
Saturated fatty acids (mmol/l)	2461	8.77	2764	9.43	2331	8.61	2713	9.43	2331	8.09	2713	7.94
Ratio of 22:6 docosahexaenoic acid to total fatty acids (%)	2463	10.74	2766	10.79	2333	10.52	2715	10.79	2333	10.52	2715	10.77
Ratio of 18:2 linoleic acid to total fatty acids (%)	2463	2.24	2766	3.96	2333	2.38	2715	4.44	2333	1.60	2715	1.96
Ratio of conjugated linoleic acid to total fatty acids (%)	2462	0.91	2765	0.98	2332	0.94	2714	0.95	2332	0.88	2714	0.78
Ratio of omega-3 fatty acids to total fatty acids (%)	2463	3.61	2766	3.68	2333	3.67	2715	3.64	2333	3.67	2715	3.76

Online Table 11 Comparing variance explained in cardiometabolic traits by change (age 10-18y) versus current (age 18y) values of body mass index (BMI), fat mass index, and lean mass index in ALSPAC

	BMI				Fat mass index				Lean mass index			
	Adj. for age, sex, ethnicity, maternal education				*Adj. for age, sex, ethnicity, maternal education*				*Adj. for age, sex, ethnicity, maternal education*			
	Change		Current		Change		Current		Change		Current	
**Standardized outcome at age 18y**	N	% R^2	N	% R^2	N	% R^2	N	% R^2	N	% R^2	N	% R^2
Ratio of omega-6 fatty acids to total fatty acids (%)	2463	3.06	2766	4.59	2333	2.92	2715	4.70	2333	2.20	2715	2.74
Ratio of polyunsaturated fatty acids to total fatty acids (%)	2463	3.57	2766	4.71	2333	3.37	2715	4.80	2333	2.69	2715	3.05
Ratio of monounsaturated fatty acids to total fatty acids (%)	2463	3.58	2766	4.78	2333	3.61	2715	5.37	2333	2.48	2715	2.59
Ratio of saturated fatty acids to total fatty acids (%)	2462	3.08	2765	3.33	2332	3.25	2714	3.54	2332	3.18	2714	3.62
Insulin (mu/l)	2512	5.73	2822	10.52	2378	4.15	2770	10.28	2378	1.32	2770	2.84
Glucose (mmol/l)	2471	4.31	2774	4.81	2341	4.02	2723	4.70	2341	3.71	2723	4.11
Lactate (mmol/l)	2471	0.55	2774	0.56	2341	0.56	2723	0.67	2341	0.52	2723	0.71
Pyruvate (mmol/l)	2471	0.48	2774	2.01	2341	0.31	2723	2.33	2341	0.15	2723	0.38
Citrate (mmol/l)	2471	1.90	2773	4.34	2341	1.81	2722	3.98	2341	1.81	2722	2.46
Alanine (mmol/l)	2471	1.56	2774	2.13	2341	1.40	2723	2.03	2341	1.26	2723	1.75
Glutamine (mmol/l)	2471	20.50	2774	20.79	2341	20.35	2723	20.53	2341	20.36	2723	20.65
Histidine (mmol/l)	2471	2.97	2774	3.26	2341	2.91	2723	3.18	2341	2.90	2723	3.20
Isoleucine (mmol/l)	2471	16.29	2774	19.56	2341	15.70	2723	18.61	2341	14.57	2723	16.69
Leucine (mmol/l)	2471	28.96	2774	31.02	2341	28.67	2723	29.85	2341	28.21	2723	30.57
Valine (mmol/l)	2471	18.52	2774	22.41	2341	18.25	2723	21.38	2341	17.61	2723	19.81
Phenylalanine (mmol/l)	2470	5.40	2773	7.71	2341	4.73	2723	6.85	2341	3.98	2723	5.94
Tyrosine (mmol/l)	2471	10.56	2774	13.59	2341	9.65	2723	13.04	2341	7.65	2723	8.90
Acetate (mmol/l)	2470	0.48	2773	0.99	2340	0.51	2722	1.04	2340	0.44	2722	0.65
Acetoacetate (mmol/l)	2471	0.98	2774	0.96	2341	1.25	2723	0.83	2341	0.79	2723	1.16
3-hydroxybutyrate (mmol/l)	2468	3.08	2770	3.33	2338	3.13	2719	2.87	2338	2.79	2719	3.39
Creatinine (mmol/l)	2471	32.77	2774	33.42	2341	32.99	2723	32.58	2341	33.17	2723	36.94
Albumin (signal area)	2472	9.16	2775	9.35	2342	9.00	2724	9.18	2342	9.00	2724	8.83
Glycoprotein acetyls, mainly a1-acid glycoprotein (mmol/l)	2471	9.79	2774	15.21	2341	9.41	2723	16.36	2341	6.91	2723	7.47
C-reactive protein (mg/l)	2557	1.60	2871	3.02	2419	1.55	2819	3.30	2419	1.24	2819	1.35

Online Table 12 Comparing variance explained in cardiometabolic traits by change (age 10-18y) versus current (age 18y) values of regional fat indexes in ALSPAC

	Trunk fat index				Arm fat index				Leg fat index			
	Adj. for age, sex, ethnicity, maternal education				*Adj. for age, sex, ethnicity, maternal education*				*Adj. for age, sex, ethnicity, maternal education*			
	Change		Current		Change		Current		Change		Current	
**Standardized outcome at age 18y**	N	% R^2	N	% R^2	N	% R^2	N	% R^2	N	% R^2	N	% R^2
Systolic blood pressure (mmHg)	3409	27.82	3988	32.81	3409	27.32	3988	31.96	3409	27.21	3988	31.69
Diastolic blood pressure (mmHg)	3409	4.67	3988	14.05	3409	4.01	3988	13.33	3409	3.78	3988	12.58
Concentration of chylomicrons and extremely large VLDL particles (mol/l)	2342	6.65	2724	12.84	2342	4.91	2724	11.27	2342	4.41	2724	8.57
Total lipids in chylomicrons and extremely large VLDL (mmol/l)	2342	6.44	2724	12.53	2342	4.75	2724	10.93	2342	4.27	2724	8.31
Phospholipids in chylomicrons and extremely large VLDL (mmol/l)	2342	6.09	2724	12.10	2342	4.45	2724	10.55	2342	3.97	2724	8.00
Total cholesterol in chylomicrons and extremely large VLDL (mmol/l)	2342	5.79	2724	11.55	2342	4.08	2724	9.89	2342	3.67	2724	7.51
Cholesterol esters in chylomicrons and extremely large VLDL (mmol/l)	2342	5.48	2724	10.83	2342	3.80	2724	9.17	2342	3.45	2724	7.02
Free cholesterol in chylomicrons and extremely large VLDL (mmol/l)	2342	5.93	2724	11.80	2342	4.29	2724	10.27	2342	3.83	2724	7.76
Triglycerides in chylomicrons and extremely large VLDL (mmol/l)	2342	6.67	2724	12.80	2342	4.99	2724	11.21	2342	4.50	2724	8.55
Concentration of very large VLDL particles (mol/l)	2342	6.77	2724	12.75	2342	5.07	2724	11.09	2342	4.69	2724	8.53
Total lipids in very large VLDL (mmol/l)	2342	6.53	2724	12.47	2342	4.84	2724	10.77	2342	4.49	2724	8.28
Phospholipids in very large VLDL (mmol/l)	2342	5.90	2724	11.67	2342	4.24	2724	10.06	2342	3.86	2724	7.64
Total cholesterol in very large VLDL (mmol/l)	2342	6.47	2724	12.67	2342	4.68	2724	10.97	2342	4.27	2724	8.37
Cholesterol esters in very large VLDL (mmol/l)	2342	6.97	2724	13.32	2342	5.13	2724	11.54	2342	4.74	2724	8.87
Free cholesterol in very large VLDL (mmol/l)	2342	5.92	2724	11.89	2342	4.22	2724	10.30	2342	3.78	2724	7.79
Triglycerides in very large VLDL (mmol/l)	2342	6.69	2724	12.50	2342	5.06	2724	10.81	2342	4.73	2724	8.37
Concentration of large VLDL particles (mol/l)	2342	6.28	2724	12.20	2342	4.63	2724	10.39	2342	4.41	2724	8.06
Total lipids in large VLDL (mmol/l)	2342	6.18	2724	12.13	2342	4.52	2724	10.31	2342	4.29	2724	7.98
Phospholipids in large VLDL (mmol/l)	2342	5.73	2724	11.64	2342	4.10	2724	9.86	2342	3.87	2724	7.57
Total cholesterol in large VLDL (mmol/l)	2342	5.76	2724	11.70	2342	4.05	2724	9.89	2342	3.79	2724	7.58
Cholesterol esters in large VLDL (mmol/l)	2342	5.75	2724	11.79	2342	3.99	2724	9.93	2342	3.76	2724	7.63
Free cholesterol in large VLDL (mmol/l)	2342	5.68	2724	11.44	2342	4.06	2724	9.71	2342	3.79	2724	7.42
Triglycerides in large VLDL (mmol/l)	2342	6.52	2724	12.43	2342	4.89	2724	10.62	2342	4.68	2724	8.29
Concentration of medium VLDL particles (mol/l)	2342	6.33	2724	12.52	2342	4.65	2724	10.64	2342	4.49	2724	8.26
Total lipids in medium VLDL (mmol/l)	2342	6.11	2724	12.34	2342	4.40	2724	10.44	2342	4.25	2724	8.07
Phospholipids in medium VLDL (mmol/l)	2342	5.35	2724	11.23	2342	3.74	2724	9.45	2342	3.61	2724	7.23
Total cholesterol in medium VLDL (mmol/l)	2342	5.00	2724	10.51	2342	3.42	2724	8.86	2342	3.29	2724	6.82
Cholesterol esters in medium VLDL (mmol/l)	2342	5.04	2724	10.04	2342	3.55	2724	8.56	2342	3.44	2724	6.74
Free cholesterol in medium VLDL (mmol/l)	2342	4.96	2724	10.54	2342	3.43	2724	8.86	2342	3.30	2724	6.75
Triglycerides in medium VLDL (mmol/l)	2342	7.49	2724	13.81	2342	5.79	2724	11.86	2342	5.63	2724	9.41
Concentration of small VLDL particles (mol/l)	2342	4.93	2724	9.67	2342	3.49	2724	8.09	2342	3.47	2724	6.28
Total lipids in small VLDL (mmol/l)	2342	5.16	2724	9.95	2342	3.74	2724	8.41	2342	3.71	2724	6.61
Phospholipids in small VLDL (mmol/l)	2342	5.57	2724	9.52	2342	4.32	2724	8.08	2342	4.35	2724	6.69
Total cholesterol in small VLDL (mmol/l)	2342	5.07	2724	9.19	2342	4.01	2724	8.08	2342	3.92	2724	6.57
Cholesterol esters in small VLDL (mmol/l)	2342	4.55	2724	8.42	2342	3.65	2724	7.51	2342	3.50	2724	6.07
Free cholesterol in small VLDL (mmol/l)	2342	5.43	2724	9.03	2342	4.31	2724	7.79	2342	4.32	2724	6.47
Triglycerides in small VLDL (mmol/l)	2342	4.65	2724	9.22	2342	3.25	2724	7.67	2342	3.25	2724	5.91
Concentration of very small VLDL particles (mol/l)	2342	8.84	2724	10.03	2342	8.53	2724	9.77	2342	8.53	2724	9.39
Total lipids in very small VLDL (mmol/l)	2342	5.41	2724	7.30	2342	5.06	2724	6.96	2342	5.00	2724	6.33
Phospholipids in very small VLDL (mmol/l)	2342	10.34	2724	11.12	2342	10.21	2724	11.05	2342	10.18	2724	10.86
Total cholesterol in very small VLDL (mmol/l)	2342	2.33	2724	4.10	2342	2.10	2724	3.83	2342	1.98	2724	3.16
Cholesterol esters in very small VLDL (mmol/l)	2342	2.53	2724	4.69	2342	2.19	2724	4.33	2342	2.02	2724	3.41
Free cholesterol in very small VLDL (mmol/l)	2342	5.03	2724	5.72	2342	5.02	2724	5.66	2342	5.01	2724	5.51
Triglycerides in very small VLDL (mmol/l)	2342	7.11	2724	8.29	2342	6.47	2724	7.71	2342	6.57	2724	7.11
Concentration of IDL particles (mol/l)	2342	10.64	2724	10.94	2342	10.62	2724	10.97	2342	10.56	2724	10.88
Total lipids in IDL (mmol/l)	2342	9.92	2724	10.45	2342	9.89	2724	10.46	2342	9.81	2724	10.36
Phospholipids in IDL (mmol/l)	2342	12.03	2724	12.21	2342	12.04	2724	12.26	2342	11.98	2724	12.25
Total cholesterol in IDL (mmol/l)	2342	7.20	2724	8.01	2342	7.14	2724	8.00	2342	7.04	2724	7.82
Cholesterol esters in IDL (mmol/l)	2342	6.02	2724	7.21	2342	5.88	2724	7.13	2342	5.75	2724	6.80
Free cholesterol in IDL (mmol/l)	2342	10.69	2724	10.85	2342	10.75	2724	10.91	2342	10.70	2724	10.94
Triglycerides in IDL (mmol/l)	2342	17.56	2724	16.63	2342	17.54	2724	16.63	2342	17.57	2724	16.65
Concentration of large LDL particles (mol/l)	2342	13.37	2724	13.65	2342	13.33	2724	13.66	2342	13.26	2724	13.60
Total lipids in large LDL (mmol/l)	2342	12.73	2724	13.14	2342	12.69	2724	13.15	2342	12.61	2724	13.08
Phospholipids in large LDL (mmol/l)	2342	12.36	2724	13.02	2342	12.27	2724	12.99	2342	12.17	2724	12.87
Total cholesterol in large LDL (mmol/l)	2342	11.18	2724	11.72	2342	11.14	2724	11.73	2342	11.04	2724	11.64
Cholesterol esters in large LDL (mmol/l)	2342	10.96	2724	11.65	2342	10.90	2724	11.64	2342	10.79	2724	11.50
Free cholesterol in large LDL (mmol/l)	2342	11.73	2724	11.94	2342	11.77	2724	12.00	2342	11.70	2724	12.01
Triglycerides in large LDL (mmol/l)	2342	19.93	2724	18.89	2342	19.93	2724	18.89	2342	19.93	2724	18.90
Concentration of medium LDL particles (mol/l)	2342	13.29	2724	13.74	2342	13.19	2724	13.68	2342	13.10	2724	13.57
Total lipids in medium LDL (mmol/l)	2342	12.65	2724	13.20	2342	12.58	2724	13.17	2342	12.49	2724	13.05
Phospholipids in medium LDL (mmol/l)	2342	12.68	2724	13.90	2342	12.46	2724	13.73	2342	12.32	2724	13.46
Total cholesterol in medium LDL (mmol/l)	2342	10.84	2724	11.47	2342	10.78	2724	11.45	2342	10.69	2724	11.32
Cholesterol esters in medium LDL (mmol/l)	2342	10.87	2724	11.49	2342	10.81	2724	11.46	2342	10.72	2724	11.34
Free cholesterol in medium LDL (mmol/l)	2342	10.47	2724	11.10	2342	10.43	2724	11.10	2342	10.32	2724	11.00
Triglycerides in medium LDL (mmol/l)	2342	21.74	2724	20.59	2342	21.74	2724	20.59	2342	21.74	2724	20.60
Concentration of small LDL particles (mol/l)	2342	12.33	2724	12.84	2342	12.23	2724	12.75	2342	12.12	2724	12.61
Total lipids in small LDL (mmol/l)	2342	11.71	2724	12.38	2342	11.63	2724	12.32	2342	11.52	2724	12.18
Phospholipids in small LDL (mmol/l)	2342	11.70	2724	12.69	2342	11.55	2724	12.50	2342	11.39	2724	12.27
Total cholesterol in small LDL (mmol/l)	2342	10.39	2724	11.04	2342	10.35	2724	11.02	2342	10.25	2724	10.91
Cholesterol esters in small LDL (mmol/l)	2342	10.84	2724	11.41	2342	10.80	2724	11.39	2342	10.70	2724	11.29
Free cholesterol in small LDL (mmol/l)	2342	7.90	2724	8.78	2342	7.87	2724	8.72	2342	7.75	2724	8.57
Triglycerides in small LDL (mmol/l)	2342	15.51	2724	14.95	2342	15.31	2724	14.81	2342	15.32	2724	14.72
Concentration of very large HDL particles (mol/l)	2342	15.47	2724	17.28	2342	14.62	2724	16.26	2342	14.84	2724	15.24
Total lipids in very large HDL (mmol/l)	2342	12.46	2724	14.13	2342	11.62	2724	13.17	2342	11.88	2724	12.29
Phospholipids in very large HDL (mmol/l)	2342	18.08	2724	20.34	2342	17.11	2724	19.12	2342	17.28	2724	17.79
Total cholesterol in very large HDL (mmol/l)	2342	5.89	2724	6.87	2342	5.28	2724	6.27	2342	5.55	2724	5.83
Cholesterol esters in very large HDL (mmol/l)	2342	4.20	2724	4.97	2342	3.69	2724	4.50	2342	3.93	2724	4.19
Free cholesterol in very large HDL (mmol/l)	2342	11.21	2724	12.75	2342	10.43	2724	11.83	2342	10.71	2724	10.99
Triglycerides in very large HDL (mmol/l)	2342	6.33	2724	6.10	2342	6.34	2724	6.11	2342	6.33	2724	6.10
Concentration of large HDL particles (mol/l)	2342	22.42	2724	24.30	2342	21.36	2724	23.24	2342	21.41	2724	21.61
Total lipids in large HDL (mmol/l)	2342	21.12	2724	23.08	2342	20.06	2724	21.99	2342	20.13	2724	20.34
Phospholipids in large HDL (mmol/l)	2342	23.47	2724	24.59	2342	22.60	2724	23.71	2342	22.65	2724	22.30
Total cholesterol in large HDL (mmol/l)	2342	18.52	2724	21.35	2342	17.28	2724	20.05	2342	17.37	2724	18.18
Cholesterol esters in large HDL (mmol/l)	2342	18.49	2724	21.40	2342	17.25	2724	20.10	2342	17.32	2724	18.21
Free cholesterol in large HDL (mmol/l)	2342	18.47	2724	20.98	2342	17.28	2724	19.72	2342	17.38	2724	17.97
Triglycerides in large HDL (mmol/l)	2342	15.78	2724	14.68	2342	15.76	2724	14.68	2342	15.74	2724	14.64
Concentration of medium HDL particles (mol/l)	2342	19.41	2724	18.44	2342	19.38	2724	18.47	2342	19.37	2724	18.34
Total lipids in medium HDL (mmol/l)	2342	18.54	2724	17.60	2342	18.47	2724	17.62	2342	18.47	2724	17.41
Phospholipids in medium HDL (mmol/l)	2342	21.22	2724	20.34	2342	21.14	2724	20.35	2342	21.14	2724	20.11
Total cholesterol in medium HDL (mmol/l)	2342	13.39	2724	12.60	2342	13.22	2724	12.57	2342	13.24	2724	12.21
Cholesterol esters in medium HDL (mmol/l)	2342	12.51	2724	11.76	2342	12.32	2724	11.72	2342	12.33	2724	11.32
Free cholesterol in medium HDL (mmol/l)	2342	17.02	2724	15.90	2342	16.95	2724	15.90	2342	16.95	2724	15.76
Triglycerides in medium HDL (mmol/l)	2342	8.54	2724	11.49	2342	7.57	2724	10.29	2342	7.68	2724	9.60
Concentration of small HDL particles (mol/l)	2342	9.25	2724	9.40	2342	9.16	2724	9.11	2342	9.17	2724	9.19
Total lipids in small HDL (mmol/l)	2342	15.77	2724	15.12	2342	15.77	2724	15.07	2342	15.77	2724	15.15
Phospholipids in small HDL (mmol/l)	2342	4.89	2724	4.90	2342	4.88	2724	4.73	2342	4.88	2724	4.78
Total cholesterol in small HDL (mmol/l)	2342	27.01	2724	26.08	2342	26.99	2724	26.09	2342	26.97	2724	25.96

Online Table 12 Comparing variance explained in cardiometabolic traits by change (age 10-18y) versus current (age 18y) values of regional fat indexes in ALSPAC

	Trunk fat index				Arm fat index				Leg fat index			
	Adj. for age, sex, ethnicity, maternal education				*Adj. for age, sex, ethnicity, maternal education*				*Adj. for age, sex, ethnicity, maternal education*			
	Change		Current		Change		Current		Change		Current	
**Standardized outcome at age 18y**	N	% R^2	N	% R^2	N	% R^2	N	% R^2	N	% R^2	N	% R^2
Cholesterol esters in small HDL (mmol/l)	2342	28.86	2724	27.97	2342	28.85	2724	27.97	2342	28.85	2724	27.89
Free cholesterol in small HDL (mmol/l)	2342	9.57	2724	8.93	2342	9.55	2724	9.00	2342	9.52	2724	8.81
Triglycerides in small HDL (mmol/l)	2342	5.79	2724	7.17	2342	5.09	2724	6.43	2342	5.21	2724	5.78
Phospholipids to total lipds ratio in chylomicrons and extremely large VLDL (%)	2342	1.30	2724	1.07	2342	1.30	2724	1.06	2342	1.34	2724	1.11
Total cholesterol to total lipids ratio in chylomicrons and extremely large VLDL (%)	2342	5.74	2724	5.90	2342	5.44	2724	5.64	2342	5.53	2724	5.60
Cholesterol esters to total lipids ratio in chylomicrons and extremely large VLDL (%)	2342	4.42	2724	4.11	2342	4.21	2724	3.97	2342	4.32	2724	3.98
Free cholesterol to total lipids ratio in chylomicrons and extremely large VLDL (%)	2342	4.48	2724	6.30	2342	4.35	2724	6.10	2342	4.34	2724	5.80
Triglycerides to total lipids ratio in chylomicrons and extremely large VLDL (%)	2342	5.21	2724	5.95	2342	5.06	2724	5.75	2342	5.08	2724	5.80
Phospholipids to total lipds ratio in very large VLDL (%)	2342	3.89	2724	6.74	2342	3.65	2724	6.31	2342	3.40	2724	5.70
Total cholesterol to total lipids ratio in very large VLDL (%)	2342	1.88	2724	2.52	2342	1.83	2724	2.32	2342	1.86	2724	2.25
Cholesterol esters to total lipids ratio in very large VLDL (%)	2342	0.61	2724	0.83	2342	0.55	2724	0.75	2342	0.64	2724	0.71
Free cholesterol to total lipids ratio in very large VLDL (%)	2342	3.08	2724	3.32	2342	2.97	2724	3.30	2342	3.17	2724	3.29
Triglycerides to total lipids ratio in very large VLDL (%)	2342	5.18	2724	5.39	2342	5.23	2724	5.38	2342	5.19	2724	5.38
Phospholipids to total lipds ratio in large VLDL (%)	2342	1.86	2724	3.89	2342	1.52	2724	3.31	2342	1.55	2724	2.88
Total cholesterol to total lipids ratio in large VLDL (%)	2342	3.91	2724	4.59	2342	3.59	2724	4.40	2342	3.61	2724	4.19
Cholesterol esters to total lipids ratio in large VLDL (%)	2342	5.27	2724	4.98	2342	5.23	2724	5.03	2342	5.22	2724	5.04
Free cholesterol to total lipids ratio in large VLDL (%)	2342	0.46	2724	0.85	2342	0.41	2724	0.75	2342	0.44	2724	0.69
Triglycerides to total lipids ratio in large VLDL (%)	2342	3.12	2724	4.32	2342	2.79	2724	3.97	2342	2.82	2724	3.63
Phospholipids to total lipds ratio in medium VLDL (%)	2342	20.60	2724	21.40	2342	19.91	2724	20.56	2342	19.91	2724	19.78
Total cholesterol to total lipids ratio in medium VLDL (%)	2342	10.68	2724	10.65	2342	10.60	2724	10.66	2342	10.61	2724	10.55
Cholesterol esters to total lipids ratio in medium VLDL (%)	2342	9.54	2724	9.40	2342	9.49	2724	9.43	2342	9.50	2724	9.38
Free cholesterol to total lipids ratio in medium VLDL (%)	2342	6.39	2724	6.83	2342	6.30	2724	6.69	2342	6.31	2724	6.48
Triglycerides to total lipids ratio in medium VLDL (%)	2342	13.96	2724	13.75	2342	13.96	2724	13.79	2342	13.96	2724	13.78
Phospholipids to total lipds ratio in small VLDL (%)	2342	6.77	2724	9.32	2342	6.06	2724	8.69	2342	5.86	2724	7.00
Total cholesterol to total lipids ratio in small VLDL (%)	2342	2.52	2724	2.66	2342	2.60	2724	2.69	2342	2.55	2724	2.68
Cholesterol esters to total lipids ratio in small VLDL (%)	2342	1.66	2724	1.65	2342	1.78	2724	1.75	2342	1.68	2724	1.69
Free cholesterol to total lipids ratio in small VLDL (%)	2342	20.80	2724	22.29	2342	20.27	2724	21.69	2342	20.12	2724	20.41
Triglycerides to total lipids ratio in small VLDL (%)	2342	4.34	2724	5.11	2342	4.24	2724	4.85	2342	4.26	2724	4.71
Phospholipids to total lipds ratio in very small VLDL (%)	2342	21.46	2724	20.70	2342	21.45	2724	20.65	2342	21.45	2724	20.61
Total cholesterol to total lipids ratio in very small VLDL (%)	2342	11.72	2724	10.53	2342	11.70	2724	10.52	2342	11.71	2724	10.53
Cholesterol esters to total lipids ratio in very small VLDL (%)	2342	15.24	2724	14.22	2342	15.27	2724	14.23	2342	15.21	2724	14.08
Free cholesterol to total lipids ratio in very small VLDL (%)	2342	4.06	2724	6.44	2342	2.90	2724	5.60	2342	2.88	2724	4.53
Triglycerides to total lipids ratio in very small VLDL (%)	2342	1.98	2724	1.62	2342	1.91	2724	1.54	2342	1.94	2724	1.54
Phospholipids to total lipds ratio in IDL (%)	2342	6.04	2724	8.52	2342	5.28	2724	7.73	2342	5.26	2724	6.29
Total cholesterol to total lipids ratio in IDL (%)	2342	6.78	2724	7.04	2342	6.70	2724	6.95	2342	6.51	2724	6.37
Cholesterol esters to total lipids ratio in IDL (%)	2342	14.64	2724	16.31	2342	14.12	2724	15.65	2342	13.87	2724	14.26
Free cholesterol to total lipids ratio in IDL (%)	2342	8.61	2724	10.49	2342	7.91	2724	9.58	2342	8.01	2724	8.80
Triglycerides to total lipids ratio in IDL (%)	2342	5.19	2724	4.67	2342	5.23	2724	4.72	2342	5.12	2724	4.55
Phospholipids to total lipds ratio in large LDL (%)	2342	12.76	2724	12.70	2342	12.75	2724	12.77	2342	12.73	2724	12.72
Total cholesterol to total lipids ratio in large LDL (%)	2342	2.51	2724	3.21	2342	2.49	2724	3.30	2342	2.37	2724	3.04
Cholesterol esters to total lipids ratio in large LDL (%)	2342	4.98	2724	6.61	2342	4.77	2724	6.55	2342	4.62	2724	6.00
Free cholesterol to total lipids ratio in large LDL (%)	2342	9.52	2724	11.79	2342	8.81	2724	11.09	2342	8.77	2724	10.24
Triglycerides to total lipids ratio in large LDL (%)	2342	5.54	2724	5.67	2342	5.53	2724	5.65	2342	5.38	2724	5.31
Phospholipids to total lipds ratio in medium LDL (%)	2342	10.68	2724	10.53	2342	10.68	2724	10.60	2342	10.66	2724	10.55
Total cholesterol to total lipids ratio in medium LDL (%)	2342	3.04	2724	3.44	2342	3.03	2724	3.53	2342	2.96	2724	3.33
Cholesterol esters to total lipids ratio in medium LDL (%)	2342	7.17	2724	7.72	2342	7.10	2724	7.78	2342	7.04	2724	7.54
Free cholesterol to total lipids ratio in medium LDL (%)	2342	13.88	2724	14.28	2342	13.73	2724	14.23	2342	13.69	2724	14.02
Triglycerides to total lipids ratio in medium LDL (%)	2342	13.34	2724	13.05	2342	13.32	2724	12.98	2342	13.20	2724	12.58
Phospholipids to total lipds ratio in small LDL (%)	2342	9.85	2724	10.10	2342	9.81	2724	10.18	2342	9.77	2724	10.05
Total cholesterol to total lipids ratio in small LDL (%)	2342	4.73	2724	5.01	2342	4.76	2724	5.12	2342	4.70	2724	5.01
Cholesterol esters to total lipids ratio in small LDL (%)	2342	8.68	2724	9.01	2342	8.65	2724	9.10	2342	8.59	2724	8.96
Free cholesterol to total lipids ratio in small LDL (%)	2342	14.64	2724	14.73	2342	14.48	2724	14.69	2342	14.45	2724	14.53
Triglycerides to total lipids ratio in small LDL (%)	2342	6.72	2724	5.99	2342	6.64	2724	5.91	2342	6.67	2724	5.92
Phospholipids to total lipds ratio in very large HDL (%)	2342	21.41	2724	24.36	2342	20.55	2724	23.10	2342	20.38	2724	21.35
Total cholesterol to total lipids ratio in very large HDL (%)	2342	22.09	2724	24.29	2342	21.47	2724	23.27	2342	21.30	2724	21.91
Cholesterol esters to total lipids ratio in very large HDL (%)	2342	20.75	2724	22.90	2342	20.13	2724	21.88	2342	20.00	2724	20.55
Free cholesterol to total lipids ratio in very large HDL (%)	2342	0.76	2724	1.03	2342	0.67	2724	0.89	2342	0.81	2724	0.83
Triglycerides to total lipids ratio in very large HDL (%)	2342	2.54	2724	5.07	2342	1.63	2724	4.37	2342	1.75	2724	3.24
Phospholipids to total lipds ratio in large HDL (%)	2342	6.06	2724	13.33	2342	4.40	2724	11.53	2342	4.31	2724	9.75
Total cholesterol to total lipids ratio in large HDL (%)	2342	5.69	2724	13.33	2342	3.81	2724	11.42	2342	3.79	2724	9.23
Cholesterol esters to total lipids ratio in large HDL (%)	2342	5.48	2724	13.33	2342	3.68	2724	11.51	2342	3.62	2724	9.28
Free cholesterol to total lipids ratio in large HDL (%)	2342	4.04	2724	7.97	2342	2.72	2724	6.62	2342	2.77	2724	5.42
Triglycerides to total lipids ratio in large HDL (%)	2342	7.18	2724	11.65	2342	5.75	2724	10.36	2342	5.85	2724	8.50
Phospholipids to total lipds ratio in medium HDL (%)	2342	10.10	2724	10.29	2342	10.08	2724	10.24	2342	10.10	2724	10.17
Total cholesterol to total lipids ratio in medium HDL (%)	2342	7.76	2724	8.00	2342	7.53	2724	7.87	2342	7.49	2724	7.72
Cholesterol esters to total lipids ratio in medium HDL (%)	2342	9.19	2724	9.28	2342	8.94	2724	9.13	2342	8.89	2724	8.95
Free cholesterol to total lipids ratio in medium HDL (%)	2342	0.48	2724	0.46	2342	0.50	2724	0.48	2342	0.50	2724	0.52
Triglycerides to total lipids ratio in medium HDL (%)	2342	4.52	2724	8.90	2342	2.99	2724	7.61	2342	3.06	2724	5.94
Phospholipids to total lipds ratio in small HDL (%)	2342	23.43	2724	23.41	2342	23.42	2724	23.34	2342	23.42	2724	23.18
Total cholesterol to total lipids ratio in small HDL (%)	2342	23.46	2724	23.77	2342	23.31	2724	23.55	2342	23.30	2724	23.06
Cholesterol esters to total lipids ratio in small HDL (%)	2342	22.85	2724	22.81	2342	22.82	2724	22.71	2342	22.81	2724	22.49
Free cholesterol to total lipids ratio in small HDL (%)	2342	8.01	2724	10.81	2342	7.18	2724	10.32	2342	7.18	2724	9.70
Triglycerides to total lipids ratio in small HDL (%)	2342	2.48	2724	4.32	2342	1.72	2724	3.80	2342	1.77	2724	2.74
Mean diameter for VLDL particles (nm)	2342	8.96	2724	14.07	2342	7.69	2724	12.27	2342	7.59	2724	10.47
Mean diameter for LDL particles (nm)	2342	4.54	2724	5.80	2342	4.37	2724	5.39	2342	4.27	2724	5.19
Mean diameter for HDL particles (nm)	2342	15.70	2724	18.99	2342	14.52	2724	17.63	2342	14.70	2724	16.12
Serum total cholesterol (mmol/l)	2342	14.31	2724	14.56	2342	14.31	2724	14.56	2342	14.22	2724	14.56
Total cholesterol in VLDL (mmol/l)	2342	5.03	2724	10.29	2342	3.69	2724	8.89	2342	3.49	2724	6.88
Remnant cholesterol (non-HDL, non-LDL -cholesterol) (mmol/l)	2342	5.10	2724	8.34	2342	4.39	2724	7.65	2342	4.20	2724	6.51
Total cholesterol in LDL (mmol/l)	2342	10.95	2724	11.54	2342	10.91	2724	11.54	2342	10.81	2724	11.43
Total cholesterol in HDL (mmol/l)	2342	21.12	2724	21.81	2342	20.38	2724	21.17	2342	20.50	2724	20.02
Total cholesterol in HDL2 (mmol/l)	2342	19.73	2724	21.09	2342	18.78	2724	20.26	2342	18.90	2724	18.79
Total cholesterol in HDL3 (mmol/l)	2342	21.91	2724	21.58	2342	21.53	2724	21.28	2342	21.62	2724	20.68
Esterified cholesterol (mmol/l)	2332	12.98	2714	13.29	2332	12.98	2714	13.26	2332	12.85	2714	13.20
Free cholesterol (mmol/l)	2330	15.10	2712	15.10	2330	15.09	2712	15.15	2330	15.08	2712	15.29
Serum total triglycerides (mmol/l)	2342	4.36	2724	8.82	2342	3.05	2724	7.43	2342	2.96	2724	5.72
Triglycerides in VLDL (mmol/l)	2342	5.94	2724	11.70	2342	4.31	2724	9.91	2342	4.16	2724	7.65
Triglycerides in LDL (mmol/l)	2342	19.89	2724	18.80	2342	19.88	2724	18.80	2342	19.89	2724	18.82
Triglycerides in HDL (mmol/l)	2342	9.66	2724	10.54	2342	9.18	2724	10.02	2342	9.25	2724	9.61
Diacylglycerol (mmol/l)	2276	2.16	2647	4.47	2276	1.71	2647	3.96	2276	1.55	2647	3.23
Ratio of diacylglycerol to triglycerides	2277	0.48	2648	1.09	2277	0.47	2648	1.09	2277	0.46	2648	1.02
Total phosphoglycerides (mmol/l)	2330	19.10	2712	18.17	2330	19.10	2712	18.17	2330	19.10	2712	18.18
Ratio of triglycerides to phosphoglycerides	2330	7.01	2712	12.11	2330	5.73	2712	10.48	2330	5.75	2712	8.59
Phosphatidylcholine and other cholines (mmol/l)	2314	19.35	2693	18.30	2314	19.35	2693	18.30	2314	19.35	2693	18.29
Total cholines (mmol/l)	2332	21.00	2714	19.86	2332	21.00	2714	19.85	2332	21.00	2714	19.87
Apolipoprotein A-I (g/l)	2342	22.87	2724	22.10	2342	22.64	2724	21.94	2342	22.71	2724	21.59
Apolipoprotein B (g/l)	2342	5.58	2724	9.04	2342	4.68	2724	8.20	2342	4.54	2724	6.92

Online Table 12 Comparing variance explained in cardiometabolic traits by change (age 10-18y) versus current (age 18y) values of regional fat indexes in ALSPAC

	**Trunk fat index**				**Arm fat index**				**Leg fat index**			
	Adj. for age, sex, ethnicity, maternal education				*Adj. for age, sex, ethnicity, maternal education*				*Adj. for age, sex, ethnicity, maternal education*			
	**Change**		**Current**		**Change**		**Current**		**Change**		**Current**	
**Standardized outcome at age 18y**	**N**	**% R^2**	**N**	**% R^2**	**N**	**% R^2**	**N**	**% R^2**	**N**	**% R^2**	**N**	**% R^2**
Ratio of apolipoprotein B to apolipoprotein A-I	2342	5.45	2724	10.03	2342	4.04	2724	8.84	2342	3.95	2724	6.72
Total fatty acids (mmol/l)	2332	11.65	2714	12.64	2332	11.31	2714	12.28	2332	11.24	2714	11.93
Estimated description of fatty acid chain length, not actual carbon number	2333	1.14	2715	0.96	2333	1.15	2715	0.93	2333	1.14	2715	0.92
Estimated degree of unsaturation	2332	4.16	2714	4.26	2332	3.94	2714	4.18	2332	3.95	2714	4.04
22:6, docosahexaenoic acid (mmol/l)	2332	14.77	2714	14.75	2332	14.87	2714	14.81	2332	14.81	2714	14.74
18:2, linoleic acid (mmol/l)	2332	14.17	2714	13.80	2332	14.12	2714	13.67	2332	14.13	2714	13.69
Conjugated linoleic acid (mmol/l)	2331	1.37	2713	1.69	2331	1.36	2713	1.76	2331	1.20	2713	1.37
Omega-3 fatty acids (mmol/l)	2332	7.79	2714	8.37	2332	7.81	2714	8.34	2332	7.63	2714	7.90
Omega-6 fatty acids (mmol/l)	2332	14.83	2714	14.84	2332	14.76	2714	14.69	2332	14.74	2714	14.71
Polyunsaturated fatty acids (mmol/l)	2332	14.61	2714	14.67	2332	14.55	2714	14.51	2332	14.50	2714	14.48
Monounsaturated fatty acids; 16:1, 18:1 (mmol/l)	2332	8.72	2714	10.67	2332	7.98	2714	10.02	2332	7.99	2714	9.55
Saturated fatty acids (mmol/l)	2331	8.71	2713	9.61	2331	8.48	2713	9.38	2331	8.39	2713	8.93
Ratio of 22:6 docosahexaenoic acid to total fatty acids (%)	2333	10.56	2715	10.81	2333	10.46	2715	10.77	2333	10.48	2715	10.77
Ratio of 18:2 linoleic acid to total fatty acids (%)	2333	2.55	2715	4.60	2333	2.15	2715	4.47	2333	2.03	2715	3.83
Ratio of conjugated linoleic acid to total fatty acids (%)	2332	0.95	2714	0.98	2332	0.98	2714	1.04	2332	0.88	2714	0.83
Ratio of omega-3 fatty acids to total fatty acids (%)	2333	3.68	2715	3.65	2333	3.67	2715	3.69	2333	3.67	2715	3.63
Ratio of omega-6 fatty acids to total fatty acids (%)	2333	3.10	2715	5.01	2333	2.63	2715	4.67	2333	2.58	2715	3.96
Ratio of polyunsaturated fatty acids to total fatty acids (%)	2333	3.54	2715	5.05	2333	3.04	2715	4.70	2333	3.05	2715	4.19
Ratio of monounsaturated fatty acids to total fatty acids (%)	2333	3.81	2715	5.58	2333	3.10	2715	5.03	2333	3.22	2715	4.78
Ratio of saturated fatty acids to total fatty acids (%)	2332	3.25	2714	3.52	2332	3.17	2714	3.45	2332	3.24	2714	3.56
Insulin (mu/l)	2378	4.61	2770	11.13	2378	3.12	2770	9.85	2378	3.09	2770	8.04
Glucose (mmol/l)	2341	4.03	2723	4.72	2341	3.95	2723	4.81	2341	3.93	2723	4.55
Lactate (mmol/l)	2341	0.55	2723	0.65	2341	0.53	2723	0.75	2341	0.56	2723	0.66
Pyruvate (mmol/l)	2341	0.42	2723	2.47	2341	0.30	2723	2.58	2341	0.18	2723	1.82
Citrate (mmol/l)	2341	1.91	2722	4.23	2341	1.79	2722	3.82	2341	1.78	2722	3.35
Alanine (mmol/l)	2341	1.45	2723	2.22	2341	1.31	2723	1.95	2341	1.30	2723	1.71
Glutamine (mmol/l)	2341	20.35	2723	20.50	2341	20.35	2723	20.55	2341	20.35	2723	20.57
Histidine (mmol/l)	2341	2.92	2723	3.19	2341	2.90	2723	3.15	2341	2.90	2723	3.15
Isoleucine (mmol/l)	2341	15.92	2723	19.47	2341	15.27	2723	18.35	2341	15.17	2723	17.04
Leucine (mmol/l)	2341	28.74	2723	30.25	2341	28.42	2723	29.59	2341	28.46	2723	29.19
Valine (mmol/l)	2341	18.31	2723	21.83	2341	18.00	2723	21.25	2341	18.03	2723	20.34
Phenylalanine (mmol/l)	2341	4.96	2723	7.15	2341	4.35	2723	6.40	2341	4.36	2723	6.25
Tyrosine (mmol/l)	2341	9.94	2723	13.58	2341	9.13	2723	12.65	2341	8.93	2723	11.73
Acetate (mmol/l)	2340	0.50	2722	1.02	2340	0.45	2722	0.95	2340	0.52	2722	1.04
Acetoacetate (mmol/l)	2341	1.25	2723	0.88	2341	1.09	2723	0.78	2341	1.19	2723	0.75
3-hydroxybutyrate (mmol/l)	2338	3.11	2719	2.95	2338	3.01	2719	2.82	2338	3.08	2719	2.75
Creatinine (mmol/l)	2341	33.01	2723	32.62	2341	32.94	2723	32.55	2341	32.95	2723	32.55
Albumin (signal area)	2342	8.99	2724	9.16	2342	8.96	2724	9.11	2342	9.01	2724	9.18
Glycoprotein acetyls, mainly a1-acid glycoprotein (mmol/l)	2341	9.84	2723	17.15	2341	8.75	2723	15.84	2341	8.47	2723	14.13
C-reactive protein (mg/l)	2419	1.64	2819	3.28	2419	1.45	2819	3.17	2419	1.40	2819	3.10

Online Figure 1 Correlations between repeated measures of total and regional fat indexes at age 10y and 18y among 2840 participants in ALSPAC

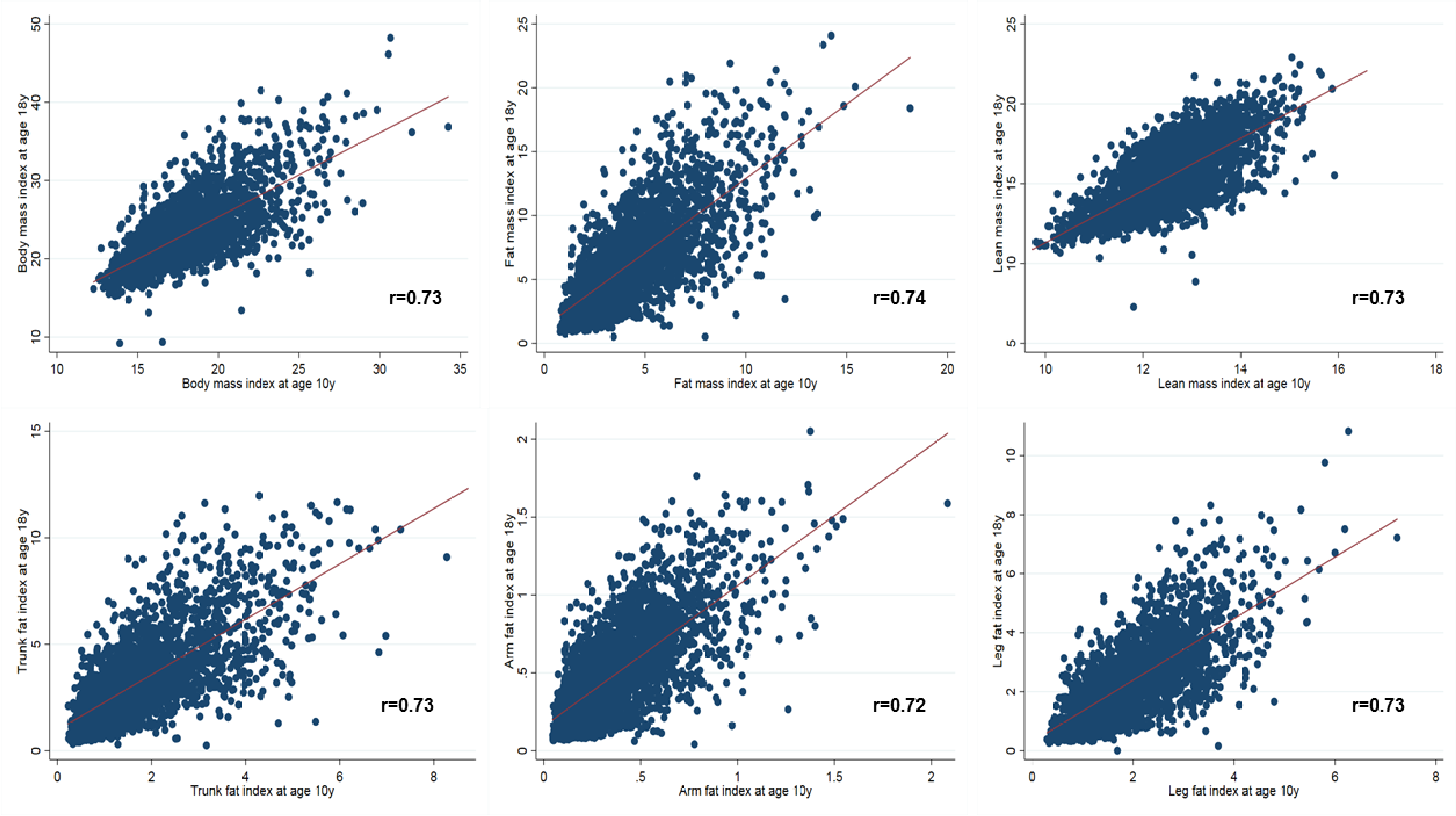

Internal lines are lines of best-fit. All correlation coefficients are P<0.0001.

Online Figure 2 Correlations of BMI with total and regional fat indexes at age 10y among 2840 participants in ALSPAC

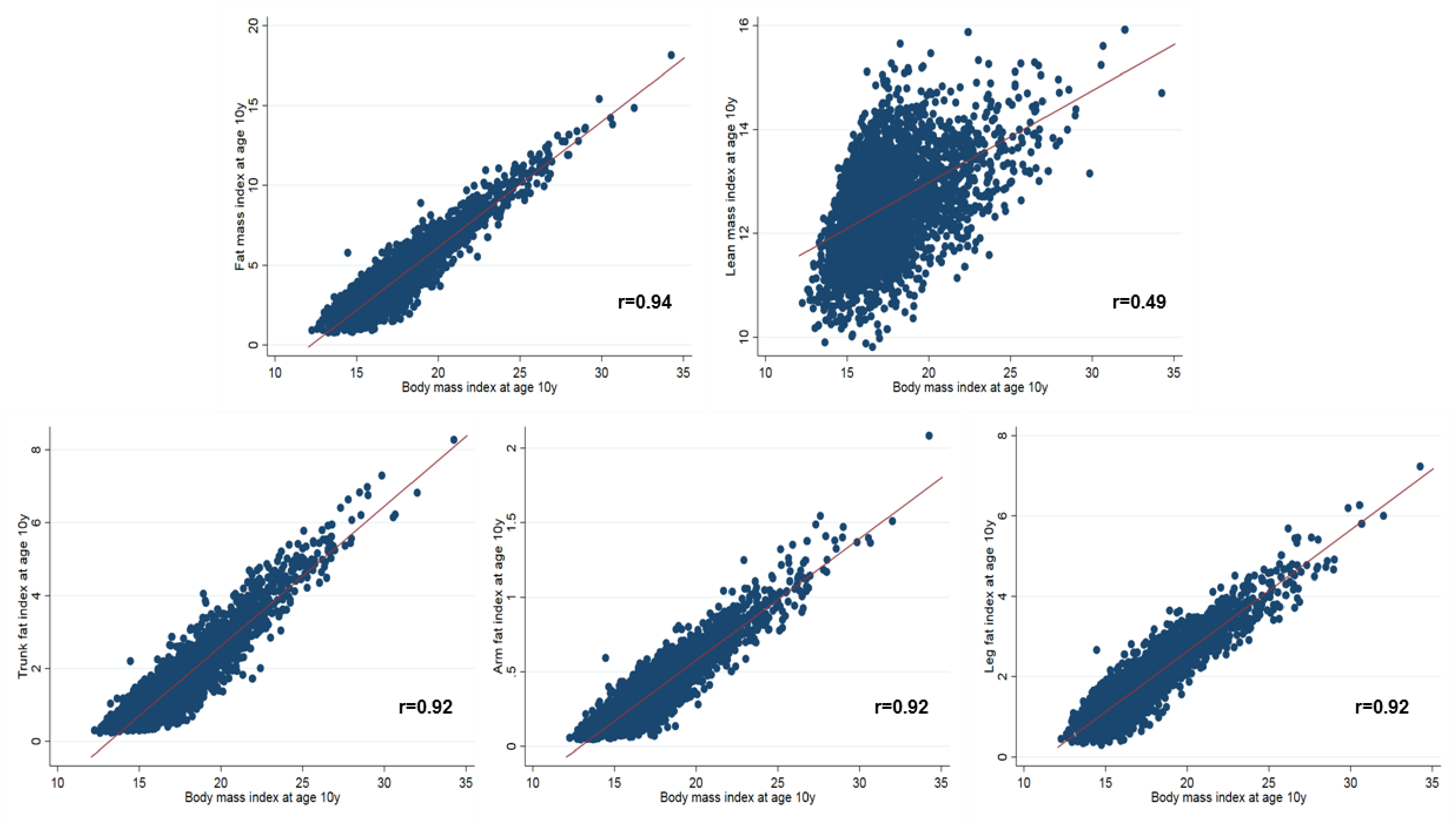

Internal lines are lines of best-fit. All correlation coefficients are P<0.0001.

Online Figure 3 Correlations of BMI with total and regional fat indexes at age 18y among 2840 participants in ALSPAC

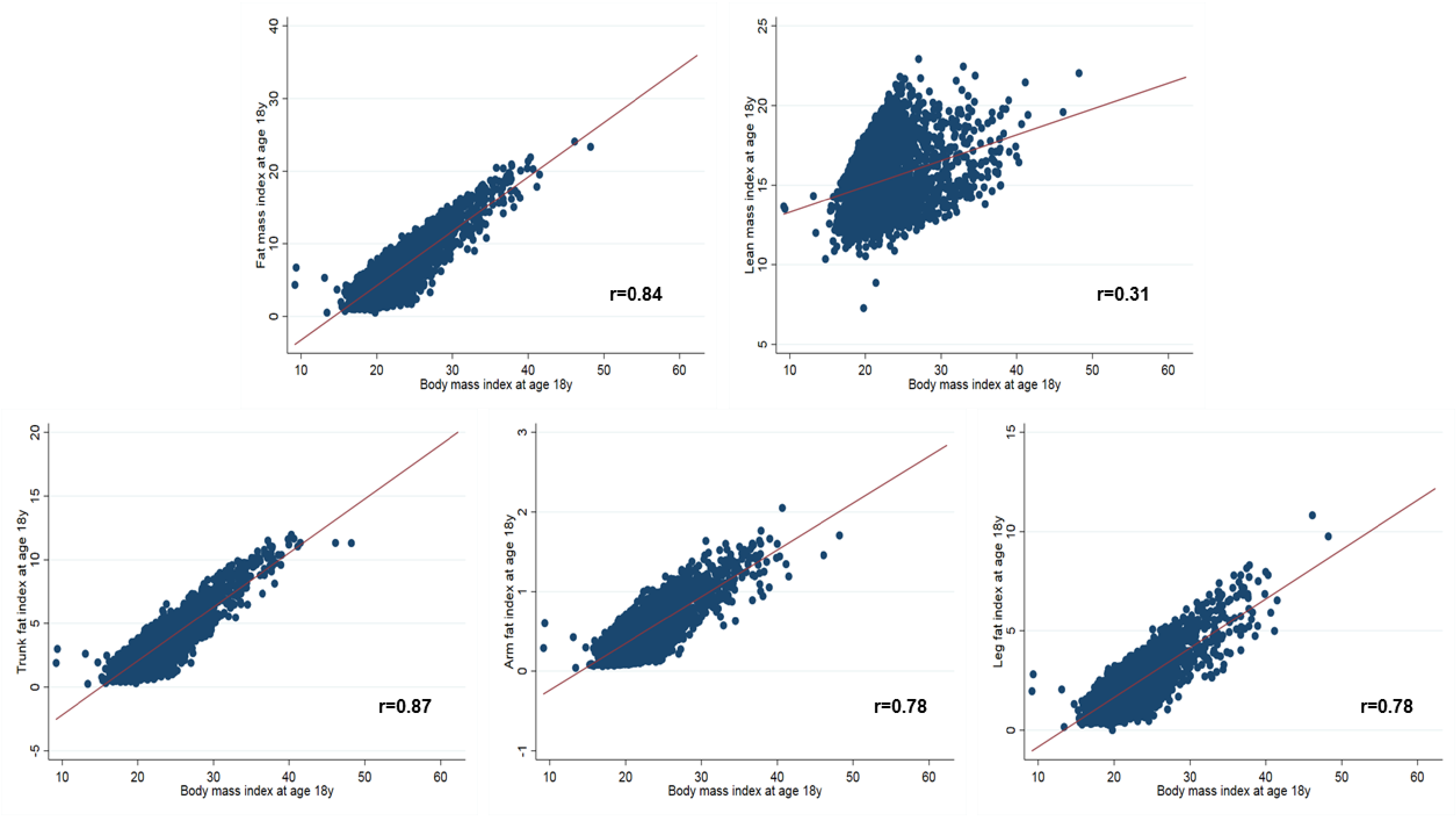

Internal lines are lines of best-fit. All correlation coefficients are P<0.0001.

Online Figure 4 Correlations of change in BMI with change in total and regional fat indexes from age 10-18y among 2840 participants in ALSPAC

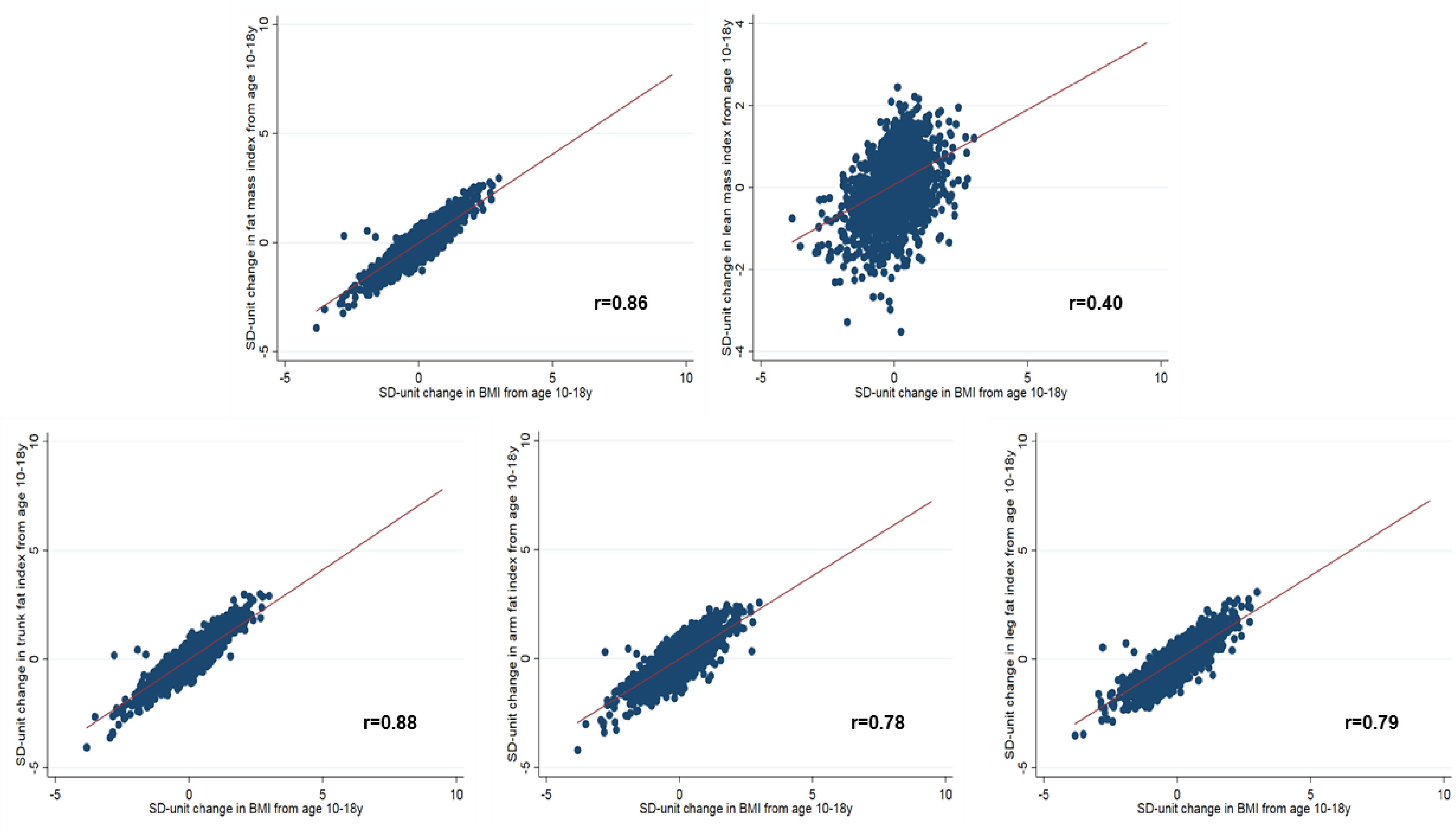

Internal lines are lines of best-fit. All correlation coefficients are P<0.0001.

Online Figure 5 Change in BMI and fat mass index from age 10-18y in relation to lipoprotein particle diameter, apolipoproteins, fatty acids, glycolysis factors, amino acids, ketones, and fluid factors at age 18y in ALSPAC

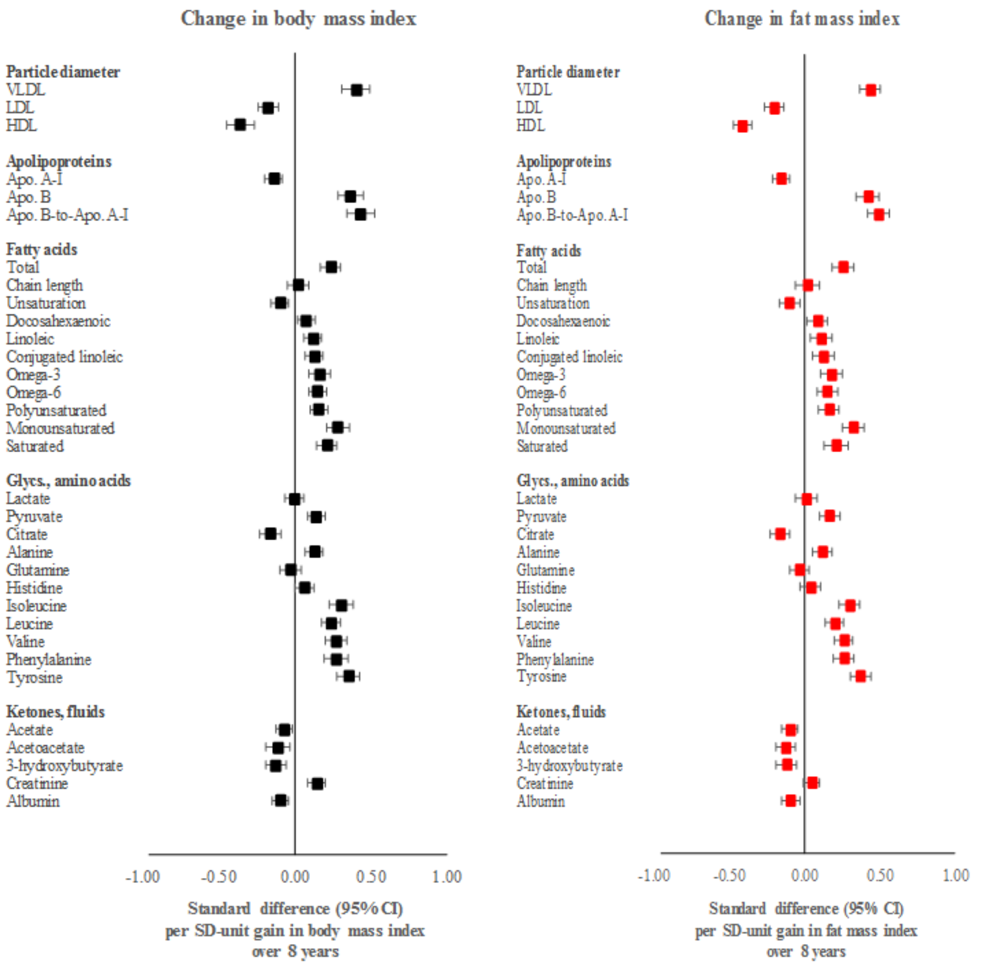

Models are adjusted for age (in months) at age 10y, sex, ethnicity, maternal education, and BMI or fat mass index at age 10y. Estimates are standardized beta coefficients from linear regression models and are interpreted as the number of SDs from the mean of the outcome distribution per SD-unit gain in BMI or fat mass index.

Online Figure 6 Change in regional fat indexes from age 10-18y in relation to lipoprotein particle diameter, apolipoproteins, fatty acids, glycolysis factors, amino acids, ketones, and fluid factors at age 18y in ALSPAC

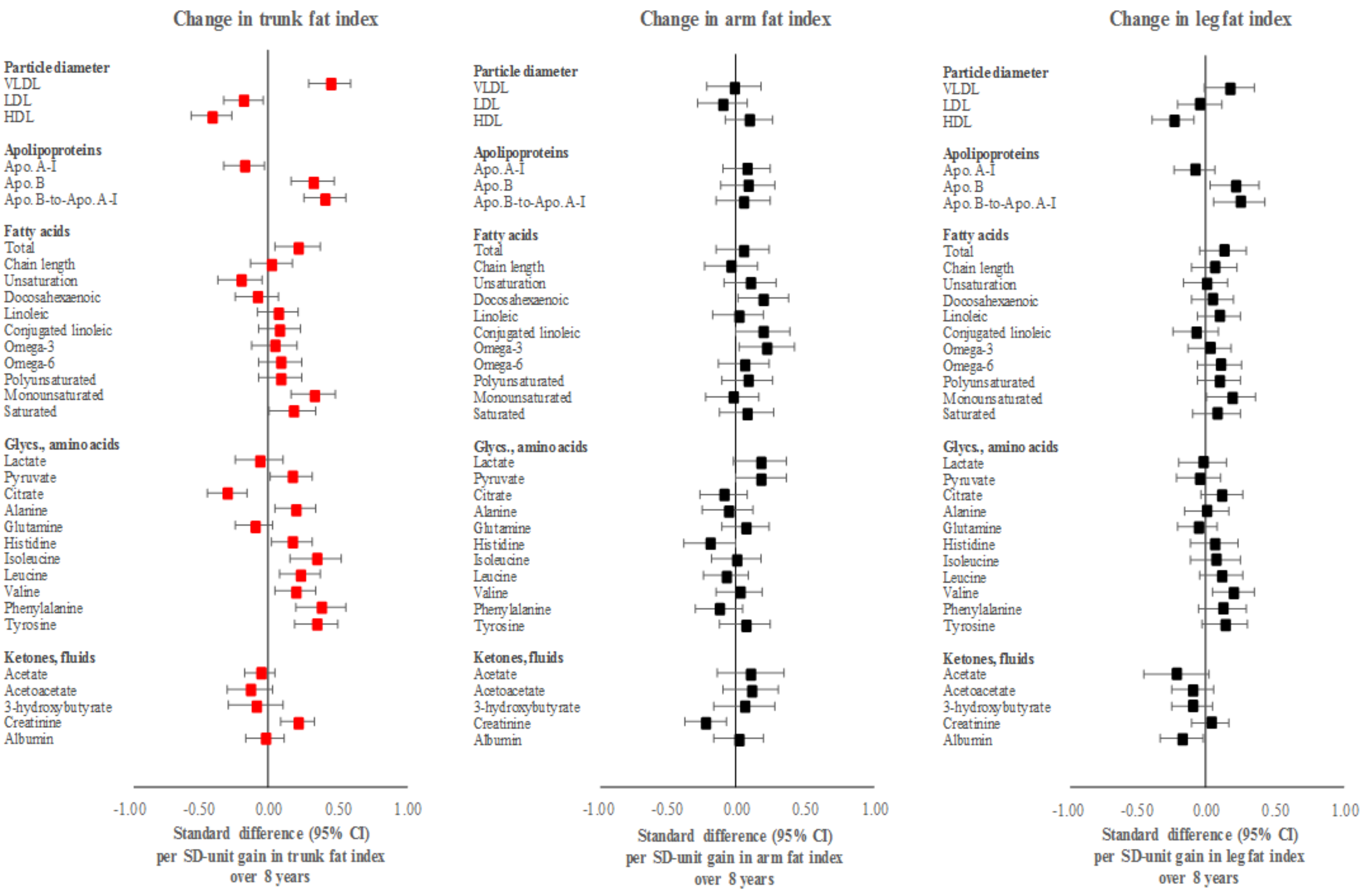

Models are adjusted for age (in months) at age 10y, sex, ethnicity, maternal education, change in alternative regional fat indexes from age 10-18y, and regional fat index exposure at age 10y. Estimates are standardized beta coefficients from linear regression models and are interpreted as the number of SDs from the mean of the outcome distribution per SD-unit gain in regional fat index.
